# Structural-Based Optimizations of the Marine-Originated Meridianin C as Glucose Uptake Agents by Inhibiting GSK-3β

**DOI:** 10.3390/md19030149

**Published:** 2021-03-12

**Authors:** Shuwen Han, Chunlin Zhuang, Wei Zhou, Fener Chen

**Affiliations:** 1 Institutes of Biomedical Sciences, Fudan University, Shanghai 200433, China; a8986330@163.com; 2 Shanghai Engineering Center of Industrial Asymmetric Catalysis for Chiral Drugs, Shanghai 200433, China; zclnathan@163.com; 3 Engineering Center of Catalysis and Synthesis for Chiral Molecules, Department of Chemistry, Fudan University, Shanghai 200433, China; 4 Department of Chemistry, Fudan University, Shanghai 200438, China

**Keywords:** glycogen synthase kinase 3β, inhibitor, structural-based optimization, diabetes, glucose uptake

## Abstract

Glycogen synthase kinase 3β (GSK-3β) is a widely investigated molecular target for numerous diseases, and inhibition of GSK-3β activity has become an attractive approach for the treatment of diabetes. Meridianin C, an indole-based natural product isolated from marine *Aplidium meridianum*, has been reported as a potent GSK-3β inhibitor. In the present study, applying the structural-based optimization strategy, the pyrimidine group of meridianin C was modified by introducing different substituents based on the 2-aminopyrimidines-substituted pyrazolo pyridazine scaffold. Among them, compounds **B29** and **B30** showed a much higher glucose uptake than meridianin C (<5%) and the positive compound 4-benzyl-2-methyl-1,2,4-thiadiazolidine-3,5-dione (TDZD-8, 16%), with no significant toxicity against HepG2 cells at the same time. Furthermore, they displayed good GSK-3β inhibitory activities (IC_50_ = 5.85; 24.4 μM). These results suggest that these meridianin C analogues represent novel lead compounds with therapeutic potential for diabetes.

## 1. Introduction

Diabetes is a chronic metabolic disease characterized by elevated levels of blood glucose, which leads to serious damage to the heart, blood vessels, eyes, kidneys, and nerves [1]. The International Diabetes Foundation (IDF) confirms that diabetes is one of the fastest growing global health emergencies in the 21st century. In 2019, it was estimated that 463 million people had diabetes, which is predicted to increase to 578 million by 2030 and 700 million by 2045 [2]. The major contemporary conventional oral antihyperglycemic agents include biguanides (metformin), second- and third-generation sulfonylureas, α-glucosidase inhibitors, thiazolidinediones, dipeptidyl peptidase-4 inhibitors, sodium-glucose co-transporter 2 inhibitors, and glucagon-like peptide-1 receptor agonists [3]. These agents are either administered as monotherapy or given in combination with other agents to achieve hypoglycemic effects [4]. Along with solubility and permeability problems, lack of target specificity, low therapeutic efficacy, severe hypoglycemia, and weight gain are the major drawbacks associated with the above-mentioned conventional drugs [5]. Thus, there is still an urgent need for new and better antihyperglycemic agents or therapeutics to reduce both the morbidity and mortality caused by diabetes mellitus.

Glycogen synthase kinase 3β (GSK-3β) is an intermediate multifunctional serine/threonine kinase having diverse physiological pathways [6,7] and involved in numerous diseases, like diabetes, inflammation, cancer, Alzheimer’s disease, and bipolar disorder [8,9,10]. Furthermore, GSK-3β functionalized as the regulator of glycogen synthase (GCS), the rate-determining enzyme for glycogen synthesis, might regulate glycogen metabolism [11,12]. Glycogen homeostasis relies on a balance between glycogenesis and glycogenolysis. Phosphorylation of GCS by GSK-3β results in its deactivation, which causes an increase in blood glucose and then obviously reduces the glycogen storage of hepatocytes in type 2 diabetes [13]. The inhibition of GSK-3β activity leads to the activation of glycogen synthase activity and may enhance glucose uptake [14]. Therefore, the inhibition of GSK-3β might provide an important and alternative therapeutic strategy for the treatment of diabetes.

**Figure 1:**
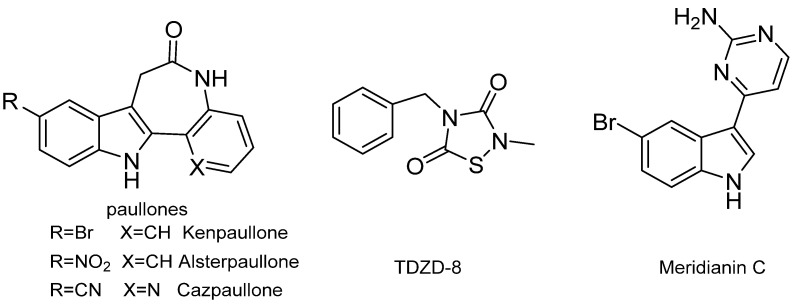
Chemical structures of the representative small-molecule glycogen synthase kinase 3β (GSK-3β) inhibitors.

A number of GSK-3β inhibitors have been reported (Figure 1), including synthetic paullones, thiadiazolidinediones (TDZDs), and natural-origin-derived small molecules [15]. However, the majority of these compounds are considered to be toolkit compounds and do not have the necessary absorption, distribution, metabolism, excretion, and toxicity (ADMET) properties to be advanced as drug candidates into the clinic [16]. Meridianin C is a brominated 3-(2-aminopyrimidine)-indole compound that was initially isolated from *Aplidium meridianum*, an ascidian from the South Georgia Islands, by Palermo and co-workers in 1998 [8,17,18]. Meridianin C was found to be a good inhibitor of GSK-3β and indicated a good antitumor activity [8,19]. However, the activity of meridianin C and its derivatives in glucose metabolism has not been disclosed yet. In the present study, we focused on structural optimizations based on meridianin C in order to obtain analogues with kinase inhibitory activity and antihyperglycemic activity.

**Figure 2:**
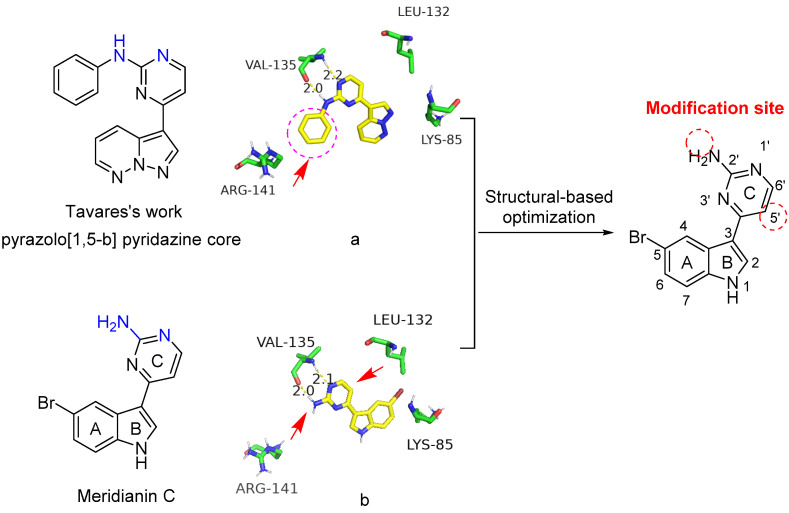
Predicted binding modes of pyrazolo[1,5-b] pyridazine (**a**) and meridianin C (**b**) with GSK-3β and the structural-based optimization strategy of meridianin C (PDB code: 6B8J). The hydrogen bonds are shown by the yellow dashed lines.

It has been reported that the 2-aminopyrimidine-substituted pyrazolo[1,5-b] pyridazine compound (Figure 2), developed by Tavares’s group, possesses excellent inhibitory activity against GSK-3β [11]. Consistent with the previous publication [11], the predicted binding mode of the compound with GSK-3β was reproduced as shown in Figure 2a. Similarly, we generated the docking model of meridianin C with the ATP-binding site of GSK-3β (Figure 2b). The NH and the adjacent nitrogen in the pyrimidine ring of these two compounds (highlighted in blue) have similar hydrogen-bonding interactions with the Val135 of GSK-3β. The aniline of the pyrazolo[1,5-b] pyridazine is located in the front solvent-exposed binding site, which has not been occupied in the meridianin C model. In addition, the back pocket formed by the residue Leu132 appears to be another pocket for optimization.

Thus, based on the chemical structure of meridianin C and the predicted binding mode with GSK-3β, modifications (highlighted arrows in red) at the amino group (2′-position and 5′-position) are proposed according to important substitutions of the pyrazolo[1,5-b] pyridazine scaffold.

## 2. Results and Discussion

### 2.1. Chemistry

**Scheme 1:**
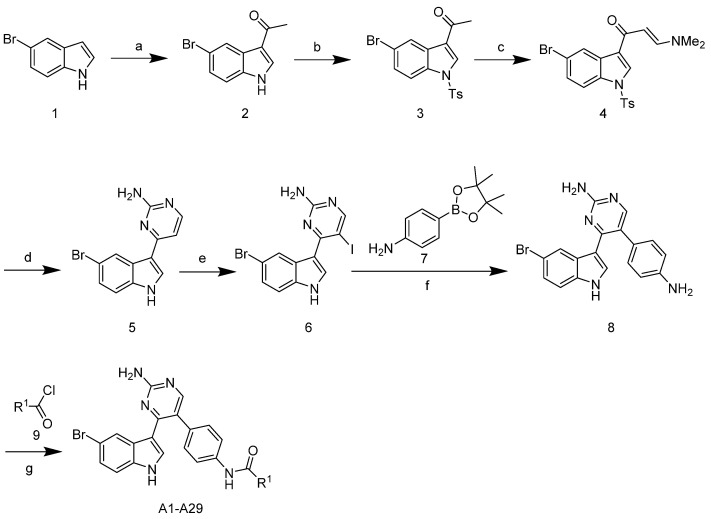
Synthetic route of the compounds **A1**–**29**. Reagents and conditions: (**a**) AcCl, SnCl_4_, toluene, 0 °C, 2 h, 87%; (**b**) TsCl, *N*,*N*-diisopropylethylamine (DIPEA), 4-(dimethylamino)-pyridine (DMAP), DCM, room temperature (rt), 20 h, 70%; (**c**) *N*,*N*-dimethylformamide diethyl acetal (DMF-DMA), DMF, 110 °C, 4 h, 52%; (**d**) guanidine-HCl, 2-methoxyethanol, K_2_CO_3_, reflux, 24 h, 51%; (**e**) *N*-iodosuccinimide (NIS), DMF, rt, 2 h, 87%; (**f**) Pd(dppf)Cl_2_CH_2_Cl_2_, Na_2_CO_3_, DME, H_2_O, 85 °C, 43%; (**g**) RCOCl, pyridine, 0 °C–rt, 15–51%.

The synthetic route of the target compounds **A1–29** is shown in Scheme 1. The commercially available starting material 5-bromoindole **1** was acetylated using acetyl chloride and SnCl_4_ in toluene to give the intermediate **2**. Then, protection of indole was performed by reaction with tosyl chloride (TsCl) in the presence of 4-(dimethylamino)-pyridine (DMAP) and *N,N*-diisopropylethylamine (DIPEA) in methylene chloride, leading to the formation of compound **3**. The reaction of compound **3** with *N,N*-dimethylformamide diethyl acetal (DMF-DMA) in *N,N*-dimethylformamide (DMF) provided enaminone **4** in a 52% yield. Treatment of enaminone **4** with guanidine hydrochloride in 2-methoxyethanol and potassium carbonate led to the formation of meridianin C **5** in a 51% yield. Meridianin C **5** was then iodinated with *N*-iodosuccinimide (NIS) in methylene chloride to give the corresponding compound **6** in an 87% yield. Suzuki cross coupling was then performed using boronate, a (1,1′-bis (diphenylphosphino) ferrocene) dichloropalladium (II) complex with dichloromethane, and sodium carbonate in an H_2_O/DME medium, which led to compound **8** in a 43% isolated yield. The target compounds **A1**–**29** were obtained from the reaction of compound **8** with commercially available substituted acyl halides.

**Scheme 2:**
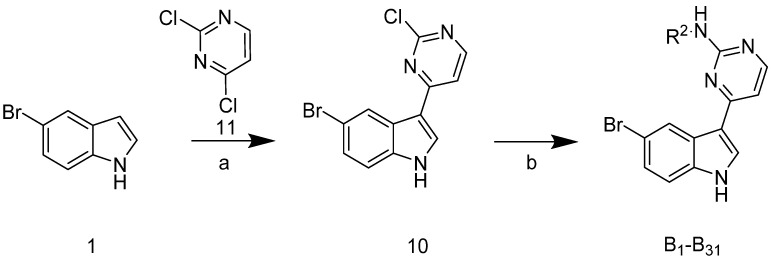
Synthetic route of the compounds **B1**–**31.** Reagents and conditions: (**a**) AlCl_3_, DCE, 85 °C, 3 h, 63%; (**b**) substituted aromatic amine, tosic acid, 2-pentanol, 125 °C, 25–63%.

The synthetic route of the target compounds **B1**–**31** is shown in Scheme 2. Compound **10** was obtained in a 63% yield using 5-bromoindole **1** and 2,4-dichloropyrimidine **11**. Target compounds **B1**–**31** were obtained from the reaction of **10** with commercially available substituted aromatic amines.

### 2.2. Biological Activity

**Figure 3:**
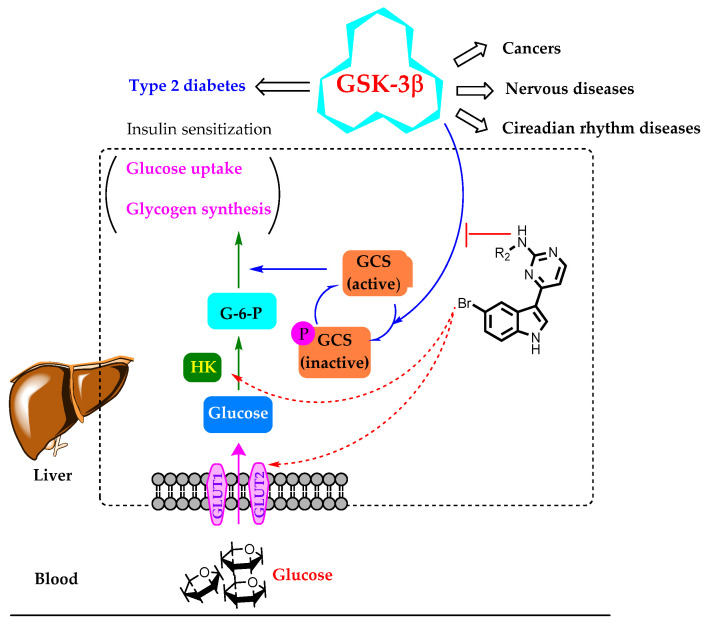
GSK-3β-mediated multicellular responses and the hypothetic mechanism of the compounds in this study for glucose uptake improvement. G-6-P, glucose 6-phosphate; GCS, glycogen synthase; HK, hexokinase.

Regulatory control of glycogen synthase (GCS), the final enzyme in glycogen biosynthesis, is an obvious target for antihyperglycemic control. Overexpression/overactivation of GSK-3β can inhibit the activation of GCS effectively, and pharmacological inhibitors of GSK-3β have been demonstrated to activate glycogen synthase and improve glycogen synthesis, suppress gluconeogenesis, and increase glucose uptake (Figure 3) [12,14,15].

Meridianin C is a marine alkaloid characterized by an indole ring connected to a pyrimidine at the three position and has extensive biological activity in GSK-3β inhibition. Anticancer [19] and antimalarial [20] GSK-3β-inhibition-related activities of meridianin C were also investigated. However, to the best of our knowledge, antihyperglycemic activity of meridianin C and its derivatives has not been disclosed yet. Thus, we hypothesized that meridianin C might influence other upstream proteins at the same time, which leads to insipid activity in glucose uptake. Nevertheless, GSK-3β has a close association with glucose uptake, and meridianin C was confirmed to be a good inhibitor of GSK-3β. Thus, we selected meridianin C as the lead compound for modification to obtain better derivatives for diabetic therapy.

Considering that there are still several key upstream signaling proteins that would influence glucose uptake in the body, such as glucose transporters (GLUT), glycogen synthase (GCS), and glycogen phosphorylase (GP) (Figure 3), in this study, we set up a cell model of glucose uptake in HepG2 cells, instead of a GSK-3β assay, to screen active compounds and investigate the structure–activity relationship in order to evaluate the glucose uptake improving activity more accurately.

Herein, viabilities of the target-compound-treated HepG2 cells were first detected to exclude the influence of cell toxicity on the determination of glucose uptake using an MTT assay. As illustrated in Table 1 and Table 2, most of the tested compounds showed concentration dependence after 48 h of incubation and CC_50_ values were shown to be higher than 5 µM, as seen in Table 1 and Table 2. Comparatively, compounds with 5′-substitutions (series A), overall, had higher cytotoxicity than those with 2′-substitutions (series B). According to previous reports [21], compounds without apparent cytotoxicity at 5 µM were selected for investigation of their cell glucose uptake effects in the following study.

Next, the synthesized meridianin derivatives were evaluated on the basis of their ability to increase glucose transport using HepG2 as the tool cell model to test their possible anti-diabetes effect. As shown in Table 1, most of the derivatives with 5′-substitutions exhibited no obvious effect on the cell glucose uptake at 5 µM, which is similar to or better than that of meridianin C (CC_50_ = 13 µM). However, compound **A13** with an *ortho*-bromo-phenyl substitution exhibited a glucose uptake ability of 27%, which was different from that of the parent meridianin C and better than that of the reference compound TDZD-8 (16%). Compound **A21** with *para*-methylphenyl and compound **A26** with 3,5-dichlorophenyl showed glucose uptake of 24% and 22%, respectively.

As shown in Table 2, the derivatives with substitutions on 2′-position of the pyrimidine ring had significant improvement in terms of the relative hepatocyte glucose uptake. Overall, in this series, introducing substituents at the *ortho*-position of the phenyl ring improved the glucose uptake more than the presence of substituents at the *para*-position and the *meta*-position. For instance, compound **B1** with 2-fluorophenyl showed a 20% glucose uptake, while compound **B3** with 4-fluorophenyl showed a 7.4% uptake and compound **B2** with 3-fluorophenyl showed no change at the same concentration. This phenomenon was also observed in the halogen and alkyl mono-substituted analogues (**B1–22**). At the *ortho*-position, the activity was along the following sequence: bromo > chloro > trifluoromethyl > fluorine > methoxy > methyl > isopropyl. Among the halogen-substituted compounds, compound **B7** with a 2-bromo group showed the best glucose uptake, of 26% at 5 µM. For the di-substituted analogues, they all showed no apparent improvement in glucose uptake, with three exceptions. The 2,6-difluorophenyl compound **B29** and the 2,6-dimethyl compound **B30** showed excellent hepatocyte glucose uptake activity (29% and 38%, respectively), with no significant toxicity against HepG2 cells. Compound **B31** with 2,6-dimethoxyl showed a 16% uptake.

To further confirm the inhibitory activities against GSK-3β of the synthesized meridianin derivatives, compounds with the best activity on cell glucose uptake were selected for a GSK-3β Kinase-Glo™ luminescent assay. As shown in Table 1 and Table 3, compounds with 5′-substitutions (**A13** and **A26**) exhibited the ability of glucose uptake but showed no apparent inhibitory activity against GSK-3β at 30 µM. On the other hand, compounds **B29** and **B30** showed much better potencies, with inhibitory activity (IC_50_) values of 5.85 and 24.4 µM, respectively, which were slightly lower than that of the lead compound meridianin C (IC_50_ = 2 µM; [18]).

**Figure 4:**
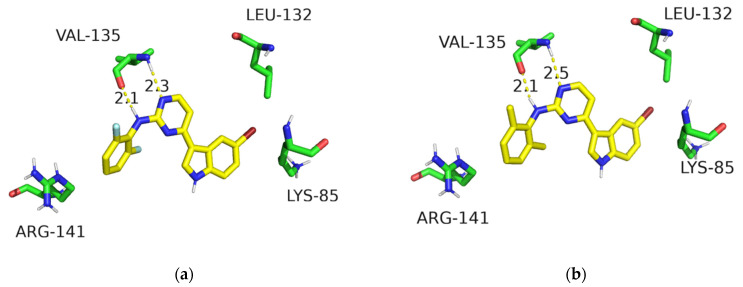
Binding modes of compounds **B29** (**a**) and **B30** (**b**) in the ATP site of GSK-3β (PDB code: 6B8J). The hydrogen bonds are shown by the yellow dashed lines.

Finally, the structural binding mode of the potent 2-aminopyrimidine-modified analogues were predicted to show compounds **B29** and **B30** adopting similar poses within the ATP-binding site of GSK-3β (Figure 4). They maintained the key interactions of meridianin C as shown in Figure 2. Additionally, the aniline group well occupied the front solvent-exposed binding site. However, no new hydrogen-bonding interactions were formed, signifying that there was no improvement in the GSK-3β but a significant increase in the cell glucose uptake.

Conclusively, meridianin C had better GSK-3β inhibition but had no glucose uptake activity, **A13** and **A26** showed better glucose uptake activity but without GSK-3β inhibition, and **B29** and **B30** displayed the best glucose uptake ability with moderate GSK-3β inhibitory activities. Therefore, from our results, there seems no close association between GSK-3β inhibitory activity and glucose uptake efficiency for meridianin C and its analogues, which is consistent with our hypothesis that these novel compounds might interfere in the other signaling proteins (such as GLUT, HK, and GCS) at the same time to influence glucose uptake (Figure 3). The mechanisms of these active compounds need to be clarified in more detail. Anyhow, in this study, two novel hit compounds of **B29** and **B30** were screened and found to have proper glucose uptake improving ability, as well as a definite GSK-3β binding and inhibitory activity. Thus, in further studies, based on the current structures, we will optimize the structures continuously, along with the activities with other involved proteins, to obtain much better anti-diabetes drugs.

## 3. Materials and Methods

### 3.1. Chemistry

#### 3.1.1. General Procedure

Chemical reagents and solvents, purchased from commercial sources, were of analytical grade and were used without further purification. All air-sensitive reactions were run under a nitrogen atmosphere. All the reactions were monitored by TLC on recoated silica gel G plates at 254 nm under a UV lamp using ethyl acetate/*n-hexane* as the eluent. Column chromatography was performed on a glass column packed with silica gel (200–300 mesh) using ethyl acetate/*n-hexane* as the eluent. Melting points were measured on an SGW X-1 microscopic melting point apparatus. Proton nuclear magnetic resonance (^1^H NMR) and carbon nuclear magnetic resonance (^13^C NMR) spectra were recorded in DMSO-*d*_6_, CD_3_OD, or CDCl_3_ on a Bruker AV 400 MHz spectrometer. Chemical shifts were reported in *δ* (ppm) units relative to the internal standard tetramethylsilane (TMS). Mass spectra and HRMS were obtained on a Waters Quattro Micromass instrument and a Bruker Compact instrument, respectively, using electrospray ionization (ESI) techniques. The purities of the target compounds were ≥95%, measured by HPLC, performed on an Agilent 1260 HPLC system with a UV detector and an Agilent Eclipse Plus C18 column (150 × 4.6 mm, 5 μm), eluting with a mixture of solvents acetonitrile and water. (a) 0–12 min, 50–95% ACN; (b) 12–16 min, 95% ACN; (c) 16–16.01 min, 95–50% ACN; (d) 16.01–20 min, 50% ACN. Peaks were detected at λ 254 nm with a flow rate of 1.0 mL/min. The final structures were fully characterized by ^1^H NMR, ^13^C NMR and HRMS (see Supplementary Materials).

#### 3.1.2. Synthesis of 1-(5-Bromo-1*H*-indol-3-yl) Ethanone **2**

To a solution of 5-bromoindole **1** (5.00 g, 25.5 mmol, 1.00 eq) in 25 mL of anhydrous toluene was added (4.00 g, 51.0 mmol, 2.00 eq) acetyl chloride at 0 °C. The resulting mixture was stirred for 15 min at 0 °C, and a solution of SnCl_4_ (13.3 g, 51.0 mmol, 2.00 eq) in 24 mL anhydrous toluene was added. The resulting solution was stirred for 2 h at 0 °C, and 75 mL of 8% NaHCO_3_ was added dropwise. The resulting slurry was diluted with 150 mL of ethyl acetate, dried (Na_2_SO_4_), and filtered. The solvent was removed with a rotary evaporator. The crude product was purified by column chromatography to afford the compound **2**. Yield: 87%, brick red solid, mp 250.4–251.3 °C. ^1^H NMR (400 MHz, CD_3_OD) *δ* 8.38 (d, *J* = 2.0 Hz, 1H), 8.17 (s, 1H), 7.45–7.23 (m, 2H), 2.51 (s, 3H).

#### 3.1.3. Synthesis of 1-(5-Bromo-1-tosyl-1*H*-indol-3-yl) Ethanone **3**

To the solution of 5-bromo-3-acetyl indole **2** (5.00 g, 21.0 mmol, 1.00 eq) in dichloromethane was added DMAP (0.128 g, 1.05 mmol, 0.0500 eq), *p*-toluenesulfonyl choride (TsCl, 4.40 g, 23.1 mmol, 1.10 eq), and *N*,*N*-diisopropylethylamine (DIPEA, 4.07 g, 31.5 mmol, 1.50 eq). The mixture was stirred at room temperature for 20 h. The reaction was then quenched by the addition of 10% HCl. Dichloromethane was added to the reaction mixture. The organic layer was separated, dried over anhydrous sodium sulphate, and concentrated to get a crude compound. The crude product was purified by column chromatography to afford the compound **3**. Yield: 70%, white solid, mp 140.6–141.5 °C. ^1^H NMR (400 MHz, CDCl_3_) *δ* 8.47 (d, *J* = 2.0 Hz, 1H), 8.15 (s, 1H), 7.79 (s, 1H), 7.76 (d, *J* = 7.3 Hz, 2H), 7.44 (d, *J* = 8.9 Hz, 1H), 7.27 (d, *J* = 8.1 Hz, 2H), 2.53 (s, 3H), 2.36 (s, 3H).

#### 3.1.4. Synthesis of 1-(5-Bromo-1-tosyl-1*H*-indol-3-yl)-3-(dimethylamino) prop-2-en-1-one **4**

1-(5-bromo-1-tosyl-1*H*-indol-3-yl) ethanone **3** (4.00 g, 10.2 mmol, 1.00 eq) was taken in anhydrous *N,N*-dimethylformamide (DMF, 40 mL), and a solution of dimethyl formamide-dimethylacetal (DMF-DMA, 1.82 g, 15.3 mmol, 1.50 eq) was added to the same solvent (4 mL). The resultant solution was heated at 110 °C for 4 h under a N_2_ atmosphere. After cooling, the solution was poured into water and then extracted with ethyl acetate. The combined organic layers were dried over anhydrous sodium sulphate and concentrated to dryness under reduced pressure. The crude product was purified by column chromatography to afford the compound **4**. Yield: 52%, pale yellow solid, mp 176.7–178.5 °C. ^1^H NMR (400 MHz, CDCl_3_) *δ* 8.44 (s, 1H), 7.96 (s, 1H), 7.73–7.64 (m, 4H), 7.37–7.29 (m, 1H), 7.16 (d, *J* = 8.1 Hz, 2H), 5.46 (d, *J* = 12.3 Hz, 1H), 3.07 (s, 3H), 2.87 (s, 3H), 2.27 (s, 3H).

#### 3.1.5. Synthesis of Meridianin C **5**

A mixture of 1-(5-bromo-1-tosyl-1*H*-indol-3-yl)-3-(dimethylamino) prop-2-en-1-one **4** (2.00 g, 4.47 mmol, 1.00 eq), guanidine hydrochloride (0.64 g, 6.71 mmol, 1.50 eq), anhydrous K_2_CO_3_ (1.24 g, 8.94 mmol, 2.00 eq), and 2-methoxyethanol (20 mL) was heated at reflux temperature for 24 h under a N_2_ atmosphere. After cooling, the solution was poured into water and then extracted with ethyl acetate. The combined organic layers were dried over anhydrous sodium sulphate and concentrated to dryness under reduced pressure. The crude product was purified by column chromatography to afford the compound **5**. Yield: 51%, pale yellow solid, mp 105.4–106.3 °C. ^1^H NMR (400 MHz, DMSO-*d*_6_) *δ* 11.86 (s, 1H), 8.75 (s, 1H), 8.25 (s, 1H), 8.11 (s, 1H), 7.40 (d, *J* = 8.1 Hz, 1H), 7.29 (d, *J* = 8.8 Hz, 1H), 7.00 (s, 1H), 6.49 (s, 2H).

#### 3.1.6. Synthesis of 4-(5-bromo-1*H*-indol-3-yl)-5-iodopyrimidin-2-amine **6**

*N*-Iodosuccinimide (NIS, 0.490 g, 2.17 mmol, 1.10 eq) was added to a solution of meridianin C **5** (0.570 g, 1.97 mmol, 1.00 eq) in anhydrous *N,N*-dimethylformamide (DMF, 6 mL). The reaction was stirred at room temperature for 1.5 h before concentrating to remove the solvent. The residue was partitioned between ethyl acetate (100 mL) and Na_2_S_2_O_3_ (100 mL). The organics were washed with NaHCO_3_ (80 mL) and brine (10 mL), dried over anhydrous sodium sulphate, and concentrated under reduced pressure. The crude product was purified by column chromatography to afford the compound **6.** Yield: 87%, pale yellow solid, mp 203.5–204.3 °C. ^1^H NMR (400 MHz, DMSO-*d*_6_) *δ* 11.85 (s, 1H), 8.51 (s, 1H), 8.46 (s, 1H), 8.39 (d, *J* = 2.0 Hz, 1H), 7.45 (d, *J* = 8.7 Hz, 1H), 7.30 (dd, *J* = 8.7, 2.1 Hz, 1H), 6.79 (s, 2H).

#### 3.1.7. Synthesis of 5-(4-aminophenyl)-4-(5-bromo-1*H*-indol-3-yl) pyrimidin-2-amine **8**

A mixture of compound **6** (0.500 g, 1.20 mmol, 1.00 eq), 4-aminophenylboronic acid pinacol ester **7** (0.317 g, 1.45 mmol, 1.20 eq), and Na_2_CO_3_ (0.319 g, 3.01 mmol, 2.50 eq) under a nitrogen atmosphere was treated with DME (9 mL), water (3 mL), and Pd (dppf) Cl_2_CH_2_Cl_2_ (0.0500 g, 0.0600 mmol, 0.0500 eq). The mixture was purged with bubbling nitrogen for 2 min and then stirred at 85 °C for 16 h, cooled to room temperature, and partitioned between ethyl acetate and water. The aqueous layer was extracted with ethyl acetate twice, and the combined organic layers were washed with brine, dried over anhydrous sodium sulphate, filtered, and concentrated. The crude product was purified by column chromatography to afford the compound **8.** Yield: 43%, pale yellow solid, mp 217.7–218.3 °C. ^1^H NMR (400 MHz, DMSO-*d*_6_) *δ* 11.49 (s, 1H), 8.65 (s, 1H), 7.94 (s, 1H), 7.32 (d, *J* = 8.5 Hz, 1H), 7.24 (d, *J* = 8.5 Hz, 1H), 6.89 (d, *J* = 7.9 Hz, 2H), 6.74 (s, 1H), 6.61 (d, *J* = 8.0 Hz, 2H), 6.55 (s, 2H), 5.33 (s, 2H). ^13^C NMR (100 MHz, DMSO-*d*_6_) *δ* 161.55, 160.62, 156.92, 148.17, 134.70, 130.90, 130.22, 128.23, 125.40, 125.19, 124.80, 121.82, 114.52, 113.68, 113.45, 112.81. HRMS calcd for C_18_H_15_BrN_5_^+^ [M + H] ^+^ 380.0505, found 380.0504.

#### 3.1.8. Synthesis of 5-bromo-3-(2-chloropyrimidin-4-yl)-1*H*-indole **10**

To the solution of 2,4-dichloropyrimidine (3.04 g, 20.4 mmol, 1.00 eq) in dichloroethane (20 mL) was added aluminum chloride (AlCl_3_, 3.26 g, 24.5 mmol, 1.20 eq) under a N_2_ atmosphere, and the mixture was stirred for 5 min. 5-Bromoindole **1** (4.00 g, 20.4 mmol, 1.00 eq) was added, and the mixture was heated at 85 °C for 3 h. After completion of the reaction, the reaction mixture was cooled at room temperature and poured into ice-cold water (30 mL) with continuous stirring for 20 min. The crude products were isolated by extracting with ethyl acetate. The organic phase was separated and washed with brine, dried over sodium sulphate, and evaporated under vacuum. The crude products obtained were further purified by silica gel column chromatography using an ethyl acetate-hexane mixture. Yield: 63%, pale yellow solid, mp 260.5–261.3 °C. ^1^H NMR (400 MHz, DMSO-*d*_6_) *δ* 12.25 (s, 1H), 8.57 (d, *J* = 2.6 Hz, 2H), 8.55 (d, *J* = 5.6 Hz, 1H), 7.92 (d, *J* = 5.5 Hz, 1H), 7.47 (d, *J* = 8.7 Hz, 1H), 7.36 (dd, *J* = 8.6, 1.9 Hz, 1H). 

##### General Procedure for the Preparation of the Final Compounds **A1**–**29**

To a solution of 5-(4-aminophenyl)-4-(5-bromo-1H-indol-3-yl) pyrimidin-2-amine 8 (200 mg, 0.530 mmol, 1.00 eq) in pyridine (5 mL), the appropriate substituted benzoyl chloride derivative (0.680 mmol, 1.30 eq) was added and the mixture stirred for 1 h. The solution was poured into ice-cold water and then extracted with ethyl acetate. The combined organic layers were dried over anhydrous sodium sulphate and concentrated to dryness under reduced pressure. The crude product was purified by column chromatography to afford the target compounds.

##### General Procedure for the Preparation of the Final Compounds **B1**–**31**

*p*-Toluene sulfonic acid hydrate (203 mg, 1.07 mmol, 1.10 eq) was added in one portion to a mixture of substituted aromatic amine (0.972 mmol, 1.00 eq) and compound **10** (300 mg, 0.972 mmol, 1.00 eq) in 2-pentanol (10 mL). The resulting mixture was then stirred at 125 °C for 18 h. the solution was poured into ice-cold water and then extracted with ethyl acetate. The combined organic layers were dried over anhydrous sodium sulphate and concentrated to dryness under reduced pressure. The crude product was purified by column chromatography to afford the target compounds.

#### 3.1.9. *N-*(4-(2-amino-4-(5-bromo-1*H*-indol-3-yl) pyrimidin-5-yl) phenyl) Propionamide **A1**

Yield: 30%, pale yellow solid, mp 193.6–194.2 °C. ^1^H NMR (400 MHz, DMSO-*d*_6_) *δ* 11.36 (s, 1H), 9.86 (s, 1H), 8.44 (s, 1H), 7.87 (s, 1H), 7.50 (d, *J* = 8.2 Hz, 2H), 7.19 (d, *J* = 8.6 Hz, 1H), 7.11 (d, *J* = 8.6 Hz, 1H), 7.05 (d, *J* = 8.1 Hz, 2H), 6.53 (d, *J* = 17.5 Hz, 3H), 2.21 (q, *J* = 7.5 Hz, 2H), 0.96 (t, *J* = 7.5 Hz, 3H). ^13^C NMR (100 MHz, DMSO-*d*_6_) *δ* 172.51, 162.91, 159.91, 158.66, 139.03, 134.97, 133.45, 130.61, 130.24, 128.37, 125.20, 124.99, 121.22, 119.67, 113.98, 113.55, 112.93, 30.01, 10.16. HRMS calcd for C_21_H_19_BrN_5_O^+^ [M + H] ^+^ 436.0767, found 437.0765. HPLC purity 98.27% (t_R_ = 10.3 min).

#### 3.1.10. *N*-(4-(2-amino-4-(5-bromo-1*H*-indol-3-yl) pyrimidin-5-yl) phenyl) Butyramide **A2**

Yield: 33%, pale yellow solid, mp 187.6–188.3 °C. ^1^H NMR (400 MHz, DMSO-*d*_6_) *δ* 11.44 (s, 1H), 9.94 (s, 1H), 8.55 (s, 1H), 7.99 (s, 1H), 7.63 (d, *J* = 8.1 Hz, 2H), 7.32 (d, *J* = 8.6 Hz, 1H), 7.23 (d, *J* = 8.1 Hz, 1H), 7.17 (d, *J* = 8.1 Hz, 2H), 6.69 (s, 1H), 6.60 (s, 2H), 2.30 (t, *J* = 7.3 Hz, 2H), 1.62 (m, 2H), 0.92 (t, *J* = 7.3 Hz, 3H). ^13^C NMR (100 MHz, DMSO-*d*_6_) *δ* 170.76, 162.02, 159.01, 157.80, 138.08, 134.09, 132.63, 129.72, 129.34, 127.49, 124.30, 124.10, 120.38, 118.84, 113.06, 112.66, 112.09, 37.95, 18.15, 13.23. HRMS calcd for C_22_H_21_BrN_5_O^+^ [M + H] ^+^ 450.0924, found 450.0920. HPLC purity 95.59% (t_R_ = 11.0 min).

#### 3.1.11. *N*-(4-(2-amino-4-(5-bromo-1*H*-indol-3-yl) pyrimidin-5-yl) phenyl) Isobutyramide **A3**

Yield: 33%, pale yellow solid, mp 172.6–173.3 °C. ^1^H NMR (400 MHz, DMSO-*d*_6_) *δ* 11.44 (s, 1H), 9.90 (s, 1H), 8.57 (s, 1H), 8.00 (s, 1H), 7.65 (d, *J* = 8.1 Hz, 2H), 7.33 (d, *J* = 8.6 Hz, 1H), 7.25 (d, *J* = 6.6 Hz, 1H), 7.18 (d, *J* = 8.2 Hz, 2H), 6.71 (s, 1H), 6.61 (s, 2H), 2.61 (m, 1H), 1.12 (d, *J* = 6.8 Hz, 6H). ^13^C NMR (100 MHz, DMSO-*d*_6_) *δ* 174.83, 162.03, 159.01, 157.81, 138.19, 134.09, 132.64, 129.74, 129.32, 127.50, 124.31, 124.11, 120.38, 118.94, 113.08, 112.67, 112.08, 34.54, 19.11. HRMS calcd for C_22_H_21_BrN_5_O^+^ [M + H] ^+^ 450.0924, found 450.0917. HPLC purity 95.64% (t_R_ = 11.0 min).

#### 3.1.12. *N*-(4-(2-amino-4-(5-bromo-1*H*-indol-3-yl) pyrimidin-5-yl) phenyl) Pivalamide **A4**

Yield: 35%, pale yellow solid, mp 139.6–140.3 °C. ^1^H NMR (400 MHz, DMSO-*d*_6_) *δ* 11.43 (s, 1H), 9.26 (s, 1H), 8.57 (s, 1H), 7.97 (s, 1H), 7.67 (d, *J* = 8.3 Hz, 2H), 7.30 (d, *J* = 8.6 Hz, 1H), 7.25–7.20 (m, 1H), 7.15 (d, *J* = 8.2 Hz, 2H), 6.67 (s, 1H), 6.61 (s, 2H), 1.21 (s, 9H). ^13^C NMR (100 MHz, DMSO-*d*_6_) *δ* 176.73, 162.69, 159.66, 158.48, 138.81, 134.75, 133.48, 130.41, 129.76, 128.17, 125.00, 124.79, 121.01, 120.57, 113.75, 113.35, 112.72, 31.39, 27.46. HRMS calcd for C_23_H_23_BrN_5_O^+^ [M + H] ^+^ 464.1080, found 464.1079. HPLC purity 96.11% (t_R_ = 11.1 min).

#### 3.1.13. *N*-(4-(2-amino-4-(5-bromo-1*H*-indol-3-yl) pyrimidin-5-yl) phenyl)-2-phenylacetamide **A5**

Yield: 35%, pale yellow solid, mp 207.6–208.4 °C. ^1^H NMR (400 MHz, DMSO-*d*_6_) *δ* 11.42 (s, 1H), 10.26 (s, 1H), 8.57 (s, 1H), 8.00 (s, 1H), 7.64 (d, *J* = 8.3 Hz, 2H), 7.36 (d, *J* = 2.0 Hz, 2H), 7.33 (d, *J* = 3.6 Hz, 2H), 7.31 (d, *J* = 4.5 Hz, 1H), 7.30–7.20 (m, 2H), 7.20 (d, *J* = 8.2 Hz, 2H), 6.68 (s, 1H), 6.62 (s, 2H), 3.66 (s, 2H). ^13^C NMR (100 MHz, DMSO-*d*_6_) *δ* 169.13, 162.46, 159.44, 158.19, 138.38, 135.98, 134.49, 133.36, 130.16, 129.84, 129.11, 128.32, 127.90, 126.56, 124.72, 124.53, 120.73, 119.35, 113.49, 113.09, 112.47, 43.40. HRMS calcd for C_26_H_21_BrN_5_O^+^ [M + H] ^+^ 498.0924, found 498.0924. HPLC purity 95.64% (t_R_ = 11.8 min).

#### 3.1.14. *N*-(4-(2-amino-4-(5-bromo-1*H*-indol-3-yl) pyrimidin-5-yl) phenyl) Benzamide **A6**

Yield: 43%, pale yellow solid, mp 187.9–188.6 °C. ^1^H NMR (400 MHz, DMSO-*d*_6_) *δ* 11.10 (s, 1H), 10.13 (s, 1H), 9.21 (d, *J* = 2.0 Hz, 1H), 8.52 (s, 1H), 8.47 (d, *J* = 7.5 Hz, 2H), 8.37 (d, *J* = 8.5 Hz, 2H), 8.03 (d, *J* = 7.3 Hz, 1H), 7.97 (t, *J* = 7.5 Hz, 2H), 7.82 (d, *J* = 8.6 Hz, 1H), 7.76 (d, *J* = 8.5 Hz, 2H), 7.71 (dd, *J* = 8.6, 2.0 Hz, 1H), 7.32 (s, 1H), 6.55 (s, 2H). ^13^C NMR (100 MHz, DMSO-*d*_6_) *δ* 175.94, 173.23, 170.46, 168.93, 149.17, 145.75, 145.40, 144.73, 141.90, 140.83, 140.34, 138.92, 138.81, 137.84, 135.79, 135.21, 132.19, 130.81. HRMS calcd for C_25_H_19_BrN_5_O^+^ [M + H] ^+^ 484.0767, found 484.0770. HPLC purity 95.56% (t_R_ = 12.6 min).

#### 3.1.15. *N*-(4-(2-amino-4-(5-bromo-1*H*-indol-3-yl) pyrimidin-5-yl) phenyl)-2-fluorobenzamide **A7**

Yield: 37%, pale yellow solid, mp 159.6–160.4 °C. ^1^H NMR (400 MHz, DMSO-*d*_6_) *δ* 10.51 (s, 1H), 8.57 (s, 1H), 8.01 (s, 1H), 7.77 (d, *J* = 8.1 Hz, 2H), 7.69 (s, 2H), 7.59 (d, *J* = 6.5 Hz, 1H), 7.36 (q, *J* = 8.3, 7.2 Hz, 3H), 7.24 (t, *J* = 8.1 Hz, 3H), 6.77 (s, 1H), 6.61 (s, 2H). ^13^C NMR (100 MHz, DMSO-*d*_6_) *δ* 175.41, 163.29, 162.95, 160.57, 159.97, 158.71, 158.10, 138.52, 135.13, 134.52, 132.97 (d, *J* = 9.2 Hz), 130.81, 130.34, 128.41, 125.51 (d, *J* = 15.3 Hz), 125.10 (d, *J* = 8.8 Hz), 124.97 (d, *J* = 11.1 Hz), 121.11, 120.43, 116.63 (d, *J* = 21.7 Hz), 114.13, 113.53, 112.81. HRMS calcd for C_25_H_18_BrFN_5_O^+^, [M + H] ^+^ 502.0673, found 502.0668. HPLC purity 95.43% (t_R_ = 12.0 min).

#### 3.1.16. *N*-(4-(2-amino-4-(5-bromo-1*H*-indol-3-yl) pyrimidiN-5-yl) phenyl)-3-fluorobenzamide **A8**

Yield: 41%, pale yellow solid, mp 183.6–184.3 °C. ^1^H NMR (400 MHz, DMSO-*d*_6_) *δ* 11.48 (s, 1H), 10.40 (s, 1H), 8.57 (d, *J* = 2.0 Hz, 1H), 8.04 (s, 1H), 7.85–7.76 (m, 4H), 7.64–7.56 (m, 1H), 7.49–7.43 (m, 1H), 7.33 (d, *J* = 8.6 Hz, 1H), 7.29–7.24 (m, 3H), 6.75 (d, *J* = 2.9 Hz, 1H), 6.63 (s, 2H). ^13^C NMR (100 MHz, DMSO-*d*_6_) *δ* 164.23, 163.18, 162.51, 160.74, 159.46, 158.31, 138.07, 134.57, 134.10, 130.65 (d, *J* = 7.9 Hz), 130.16, 129.79, 127.93, 124.73, 124.59, 123.92 (d, *J* = 2.6 Hz), 120.75, 120.64 (d, *J* = 3.1 Hz), 114.63, 114.40, 113.55, 113.14, 112.54. HRMS calcd for C_25_H_18_BrFN_5_O^+^, [M + H] ^+^ 502.0673, found 502.0673. HPLC purity 98.18% (t_R_ = 12.2 min).

#### 3.1.17. *N*-(4-(2-amino-4-(5-bromo-1*H*-indol-3-yl) pyrimidin-5-yl) phenyl)-4-fluorobenzamide **A9**

Yield: 38%, pale yellow solid, mp 178.6–179.4 °C. ^1^H NMR (400 MHz, DMSO-*d*_6_) *δ* 11.47 (s, 1H), 10.35 (s, 1H), 8.58 (d, *J* = 2.0 Hz, 1H), 8.07–8.03 (m, 3H), 7.82 (d, *J* = 8.4 Hz, 2H), 7.38 (t, *J* = 8.8 Hz, 2H), 7.33 (d, *J* = 8.6 Hz, 1H), 7.26 (d, *J* = 8.5 Hz, 3H), 6.74 (d, *J* = 2.9 Hz, 1H), 6.63 (s, 2H). ^13^C NMR (100 MHz, DMSO*-d*_6_) *δ* 165.76, 164.94, 163.29, 162.92, 159.87, 158.72, 138.68, 134.98, 134.32, 131.81 (d, *J* = 3.0 Hz), 130.85 (d, *J* = 9.2 Hz), 130.57, 130.18, 128.35, 125.07 (d, *J* = 14.9 Hz), 121.20, 120.98, 115.80 (d, *J* = 22.0 Hz), 113.96, 113.55, 112.96. HRMS calcd for C_25_H_18_BrFN_5_O^+^ [M + H] ^+^ 502.0673, found 502.0669. HPLC purity 95.97% (t_R_ = 12.1 min).

#### 3.1.18. *N*-(4-(2-amino-4-(5-bromo-1*H*-indol-3-yl) pyrimidin-5-yl) phenyl)-2-chlorobenzamide **A10**

Yield: 43%, pale yellow solid, mp 211.5–212.4 °C. ^1^H NMR (400 MHz, DMSO-*d*_6_) *δ* 11.48 (s, 1H), 10.59 (s, 1H), 8.57 (s, 1H), 8.03 (s, 1H), 7.76 (d, *J* = 8.2 Hz, 2H), 7.62–7.57 (m, 2H), 7.53 (d, *J* = 7.9 Hz, 1H), 7.49 (d, *J* = 5.8 Hz, 1H), 7.46 (d, *J* = 7.3 Hz, 1H), 7.34 (d, *J* = 8.6 Hz, 1H), 7.26 (d, *J* = 8.2 Hz, 2H), 6.77 (d, *J* = 2.9 Hz, 1H), 6.64 (s, 2H). ^13^C NMR (100 MHz, DMSO-*d*_6_) *δ* 164.55, 162.09, 159.02, 157.94, 137.64, 136.55, 134.15, 133.64, 130.73, 129.80, 129.52, 129.49, 129.28, 128.54, 127.52, 126.89, 124.30, 124.17, 120.32, 119.45, 113.14, 112.72, 112.09. HRMS calcd for C_25_H_18_BrFN_5_O^+^ [M + H] ^+^ 518.0378, found 518.0381. HPLC purity 96.64% (t_R_ = 12.1 min).

#### 3.1.19. *N*-(4-(2-amino-4-(5-bromo-1*H*-indol-3-yl) pyrimidin-5-yl) phenyl)-3-chlorobenzamide **A11**

Yield: 36%, pale yellow solid, mp 204.8–205.2 °C. ^1^H NMR (400 MHz, DMSO-*d*_6_) *δ* 11.46 (s, 1H), 10.42 (s, 1H), 8.55 (s, 1H), 8.01 (d, *J* = 7.0 Hz, 2H), 7.92 (d, *J* = 7.7 Hz, 1H), 7.80 (d, *J* = 8.1 Hz, 2H), 7.66 (d, *J* = 7.3 Hz, 1H), 7.57 (t, *J* = 7.8 Hz, 1H), 7.32 (d, *J* = 8.6 Hz, 1H), 7.28–7.23 (m, 3H), 6.73 (d, *J* = 2.8 Hz, 1H), 6.62 (s, 2H). ^13^C NMR (100 MHz, DMSO-*d*_6_) *δ* 164.13, 162.51, 159.44, 158.35, 158.31, 138.06, 136.91, 134.57, 134.11, 133.25, 131.44, 130.46, 130.15, 129.78, 127.43, 126.52, 124.72, 124.58, 120.74, 120.60, 113.54, 113.13, 112.53. HRMS calcd for C₂₅H₁₈BrClN₅O^+^, [M + H] ^+^ 518.0378, found 518.0371. HPLC purity 95.15% (t_R_ = 13.1 min).

#### 3.1.20. *N*-(4-(2-amino-4-(5-bromo-1*H*-indol-3-yl) pyrimidin-5-yl) phenyl)-4-chlorobenzamide **A12**

Yield: 42%, pale yellow solid, mp 261.7–262.5 °C. ^1^H NMR (400 MHz, DMSO-*d*_6_) *δ* 11.47 (s, 1H), 10.40 (s, 1H), 8.58 (d, *J* = 2.2 Hz, 1H), 8.04 (s, 1H), 8.01 (d, *J* = 8.6 Hz, 2H), 7.82 (d, *J* = 8.3 Hz, 2H), 7.62 (d, *J* = 8.7 Hz, 2H), 7.33 (d, *J* = 8.6 Hz, 1H), 7.27 (d, *J* = 8.4 Hz, 3H), 6.74 (s, 1H), 6.64 (s, 2H). ^13^C NMR (100 MHz, DMSO-*d*_6_) *δ* 164.70, 162.71, 159.65, 158.49, 138.36, 136.65, 134.76, 134.21, 133.85, 130.35, 129.97, 129.86, 128.70, 128.13, 124.93, 124.77, 120.96, 120.78, 113.73, 113.33, 112.74. HRMS calcd for C₂₅H₁₈BrClN₅O^+^ [M + H] ^+^ 518.0378, found 518.0385. HPLC purity 96.20% (t_R_ = 12.9 min).

#### 3.1.21. *N*-(4-(2-amino-4-(5-bromo-1*H*-indol-3-yl) pyrimidin-5-yl) phenyl)-2-bromobenzamide **A13**

Yield: 46%, pale yellow solid, mp 147.4–148.3 °C. ^1^H NMR (400 MHz, DMSO-*d*_6_) *δ* 11.49 (s, 1H), 10.58 (s, 1H), 8.59 (s, 1H), 8.04 (s, 1H), 7.80–7.71 (m, 3H), 7.61–7.57 (m, 1H), 7.52 (t, *J* = 7.5 Hz, 1H), 7.44 (td, *J* = 7.7, 1.9 Hz, 1H), 7.35 (d, *J* = 8.7 Hz, 1H), 7.29–7.25 (m, 3H), 6.78 (d, *J* = 2.8 Hz, 1H), 6.65 (s, 2H). ^13^C NMR (100 MHz, DMSO-*d*_6_) *δ* 165.38, 162.07, 158.96, 157.92, 138.68, 137.64, 134.11, 133.60, 132.31, 130.78, 129.75, 129.45, 128.44, 127.50, 127.32, 124.28, 124.13, 120.28, 119.42, 118.57, 113.09, 112.68, 112.06. HRMS calcd for C₂₅H₁₈Br₂N₅O^+^, [M + H] ^+^ 561.9873, found 561.9870. HPLC purity 96.11% (t_R_ = 12.1min).

#### 3.1.22. *N*-(4-(2-amino-4-(5-bromo-1*H*-indol-3-yl) pyrimidin-5-yl) phenyl)-3-bromobenzamide **A14**

Yield: 38%, pale yellow solid, mp 176.6–177.3 °C. ^1^H NMR (400 MHz, DMSO-*d*_6_) *δ* 11.48 (s, 1H), 10.44 (s, 1H), 8.58 (d, *J* = 2.1 Hz, 1H), 8.20–8.13 (m, 1H), 8.04 (s, 1H), 7.98 (d, *J* = 7.8 Hz, 1H), 7.86–7.77 (m, 3H), 7.52 (t, *J* = 7.8 Hz, 1H), 7.34 (d, *J* = 8.6 Hz, 1H), 7.31–7.23 (m, 3H), 6.75 (d, *J* = 2.9 Hz, 1H), 6.64 (s, 2H). ^13^C NMR (100 MHz, DMSO-*d*_6_) *δ* 164.40, 162.91, 159.82, 158.70, 138.45, 137.50, 134.95, 134.73, 134.51, 131.08, 130.66, 130.54, 130.17, 128.32, 127.29, 125.12, 124.96, 122.11, 121.12, 120.98, 113.92, 113.51, 112.93. HRMS calcd for C₂₅H₁₈Br₂N₅O^+^, [M + H] ^+^ 561.9873, found 561.9870. HPLC purity 97.54% (t_R_ = 13.3 min).

#### 3.1.23. *N*-(4-(2-amino-4-(5-bromo-1*H*-indol-3-yl) pyrimidin-5-yl) phenyl)-4-bromobenzamide **A15**

Yield: 48%, pale yellow solid, mp 276.4–277.3 °C. ^1^H NMR (400 MHz, DMSO-*d*_6_) *δ* 11.47 (s, 1H), 10.40 (s, 1H), 8.57 (d, *J* = 2.0 Hz, 1H), 8.03 (s, 1H), 7.93 (d, *J* = 8.5 Hz, 2H), 7.82 (d, *J* = 8.3 Hz, 2H), 7.76 (d, *J* = 8.3 Hz, 2H), 7.33 (d, *J* = 8.5 Hz, 1H), 7.26 (d, *J* = 8.5 Hz, 3H), 6.74 (d, *J* = 2.9 Hz, 1H), 6.63 (s, 2H). ^13^C NMR (100 MHz, DMSO-*d*_6_) *δ* 165.02, 162.91, 159.84, 158.69, 138.55, 134.96, 134.41, 132.53, 131.85, 130.55, 130.24, 130.17, 128.33, 125.78, 125.13, 124.97, 121.15, 120.97, 113.93, 113.52, 112.94. HRMS calcd for C₂₅H₁₈Br₂N₅O^+^ [M + H] ^+^ 561.9873, found 561.9878. HPLC purity 95.23% (t_R_ = 13.1 min).

#### 3.1.24. *N*-(4-(2-amino-4-(5-bromo-1*H*-indol-3-yl) pyrimidin-5-yl) phenyl)-2-(trifluoromethyl) Benzamide **A16**

Yield: 20%, pale yellow solid, mp 163.6–164.3 °C. ^1^H NMR (400 MHz, DMSO-*d*_6_) *δ* 11.60 (s, 1H), 10.70 (s, 1H), 8.58 (d, *J* = 2.0 Hz, 1H), 8.04 (s, 1H), 7.86 (d, *J* = 7.8 Hz, 1H), 7.81 (t, *J* = 7.5 Hz, 1H), 7.77–7.69 (m, 4H), 7.35 (d, *J* = 8.6 Hz, 1H), 7.29–7.25 (m, 3H), 6.79 (s, 1H), 6.68 (s, 2H).^13^C NMR (100 MHz, DMSO-*d*_6_) *δ* 165.69, 162.57, 159.50, 158.45, 138.10, 136.24, 134.65, 134.19, 132.74, 130.30, 130.17, 129.98, 128.62, 126.45 (q, *J* = 4.9 Hz), 125.24(q, *J* = 274.0 Hz),126.05, 125.74, 124.78, 124.64, 120.75, 119.99, 113.65, 113.21, 112.53. HRMS calcd for C₂₆H₁₈BrF₃N₅O^+^ [M + H] ^+^552.0641, found 552.0628. HPLC purity 96.07% (t_R_ = 12.1 min).

#### 3.1.25. *N*-(4-(2-amino-4-(5-bromo-1*H*-indol-3-yl) pyrimidin-5-yl) phenyl)-3-(trifluoromethyl) Benzamide **A17**

Yield: 43%, pale yellow solid, mp 172.6–173.3 °C. ^1^H NMR (400 MHz, DMSO-*d*_6_) *δ* 11.49 (s, 1H), 10.57 (s, 1H), 8.58 (s, 1H), 8.37–8.23 (m, 2H), 8.06 (s, 1H), 7.97 (d, *J* = 7.9 Hz, 1H), 7.84 (d, *J* = 8.5 Hz, 2H), 7.79 (d, *J* = 7.9 Hz, 1H), 7.34 (d, *J* = 8.6 Hz, 1H), 7.29 (d, *J* = 8.3 Hz, 2H), 7.26 (d, *J* = 9.0 Hz, 1H), 6.77 (s, 1H), 6.65 (s, 2H). ^13^C NMR (100 MHz, DMSO-*d*_6_) *δ* 164.09, 162.53, 159.46, 158.32, 137.98, 135.81, 134.58, 134.23, 131.86, 130.15, 129.78 (d, *J* = 4.8 Hz), 129.39, 129.07, 128.75, 128.18 (d, *J* = 3.6 Hz), 127.93, 126.49, 125.35(q, *J* = 273.7 Hz),124.65 (d, *J* = 14.9 Hz), 124.28 (q, *J* = 3.6 Hz), 120.73, 113.53, 113.13, 112.56. HRMS calcd for C₂₆H₁₈BrF₃N₅O^+^ [M + H] ^+^ 552.0641, found 552.0640. HPLC purity 97.48% (t_R_ = 13.2min).

#### 3.1.26. *N*-(4-(2-amino-4-(5-bromo-1*H*-indol-3-yl) pyrimidin-5-yl) phenyl)-4-(trifluoromethyl) Benzamide **A18**

Yield: 43%, pale yellow solid, mp 312.7–313.4 °C. ^1^H NMR (400 MHz, DMSO-*d*_6_) *δ* 11.48 (s, 1H), 10.56 (s, 1H), 8.56 (s, 1H), 8.17 (d, *J* = 8.1 Hz, 2H), 8.04 (s, 1H), 7.93 (d, *J* = 8.2 Hz, 2H), 7.83 (d, *J* = 8.5 Hz, 2H), 7.34 (d, *J* = 8.6 Hz, 1H), 7.29 (s, 1H), 7.27–7.24 (m, 2H), 6.75 (d, *J* = 2.9 Hz, 1H), 6.64 (s, 2H). ^13^C NMR (100 MHz, DMSO-*d*_6_) *δ* 164.89, 162.93, 159.87, 158.72, 139.20, 138.42, 134.99, 134.65, 132.35, 131.97, 131.66, 130.40 (d, *J* = 32.8 Hz), 129.05, 128.34, 125.86 (q, *J* = 3.5 Hz), 125.80 (d, *J* = 279.0 Hz), 123.01(q, *J* = 3.5 Hz), 121.15, 121.04, 113.96, 113.55, 112.96. HRMS calcd for C₂₆H₁₈BrF₃N₅O^+^, [M + H] ^+^ 552.0641, found 552.0636. HPLC purity 97.91% (t_R_ = 12.9 min).

#### 3.1.27. *N*-(4-(2-amino-4-(5-bromo-1*H*-indol-3-yl) pyrimidin-5-yl) phenyl)-2-methylbenzamide **A19**

Yield: 33%, pale yellow solid, mp 170.7–171.6 °C. ^1^H NMR (400 MHz, DMSO-*d*_6_) *δ* 10.34 (s, 1H), 8.48 (s, 1 H), 7.93 (s, 1H), 7.70 (d, *J* = 8.2 Hz, 2H), 7.38 (d, *J* = 7.1 Hz, 1H), 7.31 (d, *J* = 7.8 Hz, 1H), 7.28 (d, *J* = 5.3 Hz, 1H), 7.24 (d, *J* = 7.2 Hz, 2H), 7.20 (d, *J* = 7.1 Hz, 1H), 7.18–7.13 (m, 3H), 6.69 (s, 1H), 6.56 (s, 2H), 2.31 (s, 3H). ^13^C NMR (100 MHz, DMSO-*d*_6_) *δ* 167.57, 162.12, 159.12, 157.98, 138.08, 136.86, 134.87, 134.31, 133.38, 130.20, 130.00, 129.44, 129.32, 127.59, 126.88, 125.34, 124.35, 124.16, 120.40, 119.54, 113.29, 112.75, 112.09, 19.00. HRMS calcd for C₂₆H₂₁BrN₅O^+^ [M + H] ^+^498.0924, found 498.0921. HPLC purity 96.94% (t_R_ = 12.2 min).

#### 3.1.28. *N*-(4-(2-amino-4-(5-bromo-1*H*-indol-3-yl) pyrimidin-5-yl) phenyl)-3-methylbenzamide **A20**

Yield: 42%, pale yellow solid, mp 211.8–212.9 °C. ^1^H NMR (400 MHz, DMSO-*d*_6_) *δ* 11.45 (s, 1H), 10.23 (s, 1H), 8.49 (s, 1H), 7.93 (s, 1H), 7.74 (d, *J* = 8.2 Hz, 2H), 7.67 (d, *J* = 12.5 Hz, 2H), 7.32 (d, *J* = 5.8 Hz, 2H), 7.24 (d, *J* = 8.6 Hz, 1H), 7.15 (d, *J* = 8.3 Hz, 3H), 6.64 (s, 1H), 6.56 (s, 2H), 2.30 (s, 3H). ^13^C NMR (100 MHz, DMSO-*d*_6_) *δ* 166.44, 163.17, 160.15, 158.97, 139.10, 138.43, 135.65, 135.24, 134.44, 132.88, 130.88, 130.41, 129.02, 128.85, 128.61, 125.54, 125.42, 125.26, 121.45, 121.16, 114.25, 113.82, 113.18, 21.68. HRMS calcd for C₂₆H₂₁BrN₅O^+^ [M + H] ^+^498.0924, found 498.0923. HPLC purity 95.11% (t_R_ = 12.4 min).

#### 3.1.29. *N*-(4-(2-amino-4-(5-bromo-1*H*-indol-3-yl) pyrimidin-5-yl) phenyl)-4-methylbenzamide **A21**

Yield: 42%, pale yellow solid, mp 291.7–292.4 °C. ^1^H NMR (400 MHz, DMSO-*d*_6_) *δ* 11.47 (s, 1H), 10.24 (s, 1H), 8.58 (s, 1H), 8.04 (s, 1H), 7.89 (d, *J* = 7.9 Hz, 2H), 7.83 (d, *J* = 8.6 Hz, 2H), 7.37–7.31 (m, 3H), 7.25 (d, *J* = 8.5 Hz, 3H), 6.74 (s, 1H), 6.63 (s, 2H), 2.39 (s, 3H). ^13^C NMR (100 MHz, DMSO-*d*_6_) *δ* 165.82, 162.90, 159.86, 158.67, 142.03, 138.85, 134.95, 134.09, 132.48, 130.56, 130.11, 129.34, 128.34, 128.12, 125.15, 124.97, 121.21, 120.89, 113.93, 113.52, 112.94, 21.43. HRMS calcd for C₂₆H₂₁BrN₅O^+^, [M + H] ^+^ 498.0924, found 498.0930. HPLC purity 96.26% (t_R_ = 12.9 min).

#### 3.1.30. *N*-(4-(2-amino-4-(5-bromo-1*H*-indol-3-yl) pyrimidin-5-yl) phenyl)-2-methoxybenzamide **A22**

Yield: 22%, pale yellow solid, mp 154.9–155.6 °C. ^1^H NMR (400 MHz, DMSO-*d*_6_) *δ* 11.47 (s, 1H), 10.21 (s, 1H), 8.60 (s, 1H), 8.03 (s, 1H), 7.79 (d, *J* = 8.3 Hz, 2H), 7.65 (d, *J* = 5.8 Hz, 1H), 7.51 (t, *J* = 7.8 Hz, 1H), 7.34 (d, *J* = 8.6 Hz, 1H), 7.27–7.23 (m, 3H), 7.19 (d, *J* = 8.4 Hz, 1H), 7.08 (t, *J* = 7.5 Hz, 1H), 6.75 (d, *J* = 2.9 Hz, 1H), 6.63 (s, 2H), 3.91 (s, 3H). ^13^C NMR (100 MHz, DMSO-*d*_6_) *δ* 164.37, 162.24, 159.30, 158.01, 156.28, 138.04, 134.35, 133.47, 131.85, 130.03, 129.63, 129.46, 127.75, 124.79, 124.54, 124.39, 120.59, 120.33, 119.75, 113.34, 112.95, 112.31, 111.82, 55.72. HRMS calcd for C₂₆H₂₁BrN₅O₂^+^ [M + H] ^+^ 514.0873, found 514.0869. HPLC purity 97.46% (t_R_ = 13.1 min).

#### 3.1.31. *N*-(4-(2-amino-4-(5-bromo-1*H*-indol-3-yl) pyrimidin-5-yl) phenyl)-3-methoxybenzamide **A23**

Yield: 43%, pale yellow solid, mp 172.6–173.3 °C. ^1^H NMR (400 MHz, DMSO-*d*_6_) *δ* 11.61 (s, 1H), 10.36 (s, 1H), 8.57 (s, 1H), 8.03 (s, 1H), 7.83 (d, *J* = 8.2 Hz, 2H), 7.56 (d, *J* = 7.7 Hz, 1H), 7.50 (s, 1H), 7.45 (t, *J* = 7.9 Hz, 1H), 7.34 (d, *J* = 8.5 Hz, 1H), 7.25 (d, *J* = 8.3 Hz, 3H), 7.19–7.13 (m, 1H), 6.75 (s, 1H), 6.65 (s, 2H), 3.84 (s, 3H).^13^C NMR (100 MHz, DMSO-*d*_6_) *δ* 165.05, 162.15, 159.16, 158.86, 157.95, 137.96, 136.01, 134.26, 133.54, 129.89, 129.40, 129.30, 127.59, 124.39, 124.24, 120.44, 120.30, 119.58, 117.04, 113.27, 112.81, 112.59, 112.15, 55.03. HRMS calcd for C₂₆H₂₁BrN₅O₂^+^ [M + H] ^+^ 514.0873, found 514.0871. HPLC purity 98.12% (t_R_ = 12.1 min).

#### 3.1.32. *N*-(4-(2-amino-4-(5-bromo-1*H*-indol-3-yl) pyrimidin-5-yl) phenyl)-4-methoxybenzamide **A24**

Yield: 51%, pale yellow solid, mp 184.6–185.3 °C. ^1^H NMR (400 MHz, DMSO-*d*_6_) *δ* 11.47 (s, 1H), 10.18 (s, 1H), 8.58 (d, *J* = 2.0 Hz, 1H), 8.04 (s, 1H), 7.98 (d, *J* = 8.8 Hz, 2H), 7.82 (d, *J* = 8.6 Hz, 2H), 7.33 (d, *J* = 8.6 Hz, 1H), 7.24 (d, *J* = 8.5 Hz, 3H), 7.07 (d, *J* = 8.8 Hz, 2H), 6.74 (d, *J* = 2.9 Hz, 1H), 6.62 (s, 2H), 3.84 (s, 3H). ^13^C NMR (100 MHz, DMSO-*d*_6_) *δ* 164.77, 162.26, 161.73, 159.27, 158.03, 138.35, 134.34, 133.34, 129.96, 129.47, 129.42, 127.73, 126.76, 124.54, 124.36, 120.63, 120.27, 113.43, 113.32, 112.92, 112.33, 55.25. HRMS calcd for C₂₆H₂₁BrN₅O₂^+^, [M + H] ^+^ 514.0873, found 514.0871. HPLC purity 96.66% (t_R_ = 12.1 min).

#### 3.1.33. *N*-(4-(2-amino-4-(5-bromo-1*H*-indol-3-yl) pyrimidin-5-yl) phenyl)-2, 5-difluorobenzamide **A25**

Yield: 36%, pale yellow solid, mp 163.5–164.3 °C. ^1^H NMR (400 MHz, DMSO-*d*_6_) *δ* 11.48 (s, 1H), 10.59 (s, 1H), 8.58 (d, *J* = 2.0 Hz, 1H), 8.03 (s, 1H), 7.76 (d, *J* = 8.2 Hz, 2H), 7.58–7.53 (m, 1H), 7.47–7.40 (m, 2H), 7.34 (d, *J* = 8.6 Hz, 1H), 7.29–7.24 (m, 3H), 6.75 (d, *J* = 2.9 Hz,1H), 6.64 (s, 2H). ^13^C NMR (100 MHz, DMSO-*d*_6_) *δ* 162.94, 161.91, 159.86, 159.44, 158.72, 157.03, 156.71, 154.27, 138.21, 134.97, 134.71, 130.48 (d, *J* = 24.3 Hz), 128.35, 126.68 (dd, *J* = 18.6, 7.4 Hz), 125.07 (d, *J* = 12.5 Hz), 121.10, 120.46, 119.41 (dd, *J* = 24.2, 9.0 Hz), 118.47 (dd, *J* = 25.0, 8.5 Hz), 116.65 (dd, *J* = 25.8, 3.6 Hz), 113.97, 113.56, 112.91. HRMS calcd for C₂₅H₁₇BrF₂N₅O^+^, [M + H] ^+^ 520.0579, found 520.0586. HPLC purity 95.42% (t_R_ = 12.4 min).

#### 3.1.34. *N*-(4-(2-amino-4-(5-bromo-1*H*-indol-3-yl) pyrimidin-5-yl) phenyl)-3, 5-dichlorobenzamide **A26**

Yield: 33%, pale yellow solid, mp 177.7–178.5 °C. ^1^H NMR (400 MHz, DMSO-*d*_6_) *δ* 11.49 (s, 1H), 10.51 (s, 1H), 8.57 (s, 1H), 8.04 (s, 1H), 8.00 (d, *J* = 2.0 Hz, 2H), 7.87 (s, 1H), 7.81 (d, *J* = 8.5 Hz, 2H), 7.34 (d, *J* = 8.6 Hz, 1H), 7.27 (t, *J* = 10.2 Hz, 3H), 6.75 (d, *J* = 3.0 Hz, 1H), 6.65 (s, 2H). ^13^C NMR (100 MHz, DMSO-*d*_6_) *δ* 162.28, 162.09, 158.99, 157.86, 137.66, 137.35, 134.13, 133.90, 130.53, 129.68, 129.38, 127.48, 126.59, 126.09, 124.27, 124.13, 120.25, 120.20, 113.09, 112.68, 112.09. HRMS calcd for C₂₅H₁₇BrCl₂N₅O^+^, [M + H] ^+^ 551.9988, found 551.9988. HPLC purity 97.16% (t_R_ = 13.2 min).

#### 3.1.35. *N*-(4-(2-amino-4-(5-bromo-1*H*-indol-3-yl) pyrimidin-5-yl) phenyl) thiophene-2-carboxamide **A27**

Yield: 15%, pale yellow solid, mp 303.8–304.4 °C. ^1^H NMR (400 MHz, DMSO-*d*_6_) *δ* 11.63 (s, 1H), 10.39 (s, 1H), 8.57 (s, 1H), 8.06 (d, *J* = 15.0 Hz, 2H), 7.87 (d, *J* = 5.0 Hz, 1H), 7.79 (d, *J* = 8.1 Hz, 2H), 7.34 (d, *J* = 8.6 Hz, 1H), 7.26 (d, *J* = 8.4 Hz, 4H), 6.70 (d, *J* = 27.3 Hz, 3H). ^13^C NMR (100 MHz, DMSO-*d*_6_) *δ* 162.93, 160.37, 159.90, 158.72, 140.49, 138.38, 135.03, 134.33, 132.40, 130.66, 130.23, 129.67, 128.57, 128.36, 125.15, 124.97, 121.13, 120.96, 114.03, 113.54, 112.92. HRMS calcd for C₂₃H₁₇BrN₅OS^+^ [M + H] ^+^490.0332, found 490.0332. HPLC purity 95.22 % (t_R_ = 11.7 min).

#### 3.1.36. *N*-(4-(2-amino-4-(5-bromo-1*H*-indol-3-yl) pyrimidin-5-yl) phenyl) isoquinoline-3-carboxamide **A28**

Yield: 43%, pale yellow solid, mp 189.3–190.2 °C. ^1^H NMR (400 MHz, DMSO-*d*_6_) *δ* 11.46 (s, 1H), 10.81 (s, 1H), 8.59 (d, *J* = 8.5 Hz, 1H), 8.52 (d, *J* = 2.0 Hz, 1H), 8.25–8.20 (m, 2H), 8.10–8.05 (m, 1H), 8.02 (s, 1H), 7.99–7.95 (m, 2H), 7.90–7.85 (m, 1H), 7.74–7.69 (m, 1H), 7.30–7.27 (m, 2H), 7.25 (d, *J* = 2.0 Hz, 1H), 7.22–7.18 (m, 1H), 6.71 (d, *J* = 2.8 Hz, 1H), 6.63 (s, 2H). ^13^C NMR (100 MHz, DMSO-*d*_6_) *δ* 162.46, 162.14, 159.10, 157.92, 149.73, 145.54, 137.88, 137.12, 134.19, 133.83, 130.35, 129.81, 129.51, 129.01, 128.58, 128.05, 127.79, 127.53, 124.34, 124.20, 120.34, 120.13, 118.45, 113.19, 112.75, 112.16. HRMS calcd for C₂₈H₂₀BrN₆O^+^ [M + H] ^+^535.0876, found 535.0873. HPLC purity 95.84% (t_R_ = 14.6 min).

#### 3.1.37. *N*-(4-(2-amino-4-(5-bromo-1*H*-indol-3-yl) pyrimidin-5-yl) phenyl)-2-naphthamide **A29**

Yield: 43%, pale yellow solid, mp 189.3–190.2 °C. ^1^H NMR (400 MHz, DMSO-*d*_6_) *δ* 11.51 (s, 1H), 10.59 (s, 1H), 8.60 (d, *J* = 7.1 Hz, 2H), 8.04 (q, *J* = 11.1, 7.4 Hz, 5H), 7.90 (d, *J* = 8.0 Hz, 2H), 7.75–7.58 (m, 2H), 7.35 (d, *J* = 8.7 Hz, 1H), 7.28 (d, *J* = 9.0 Hz, 3H), 6.72 (d, *J* = 43.7 Hz, 3H). ^13^C NMR (100 MHz, DMSO-*d*_6_) *δ* 166.10, 162.82, 159.89, 158.64, 138.78, 134.95, 134.65, 134.19, 132.57, 132.44, 130.58, 130.14, 129.36, 128.44, 128.39, 128.27, 128.05, 127.27, 125.07, 124.96, 124.84, 121.17, 120.95, 113.97, 113.52, 112.86. HRMS calcd for C₂₉H₂₁BrN₅O^+^ [M + H] ^+^534.0924, found 534.0922. HPLC purity 98.46% (t_R_ = 13.4 min).

#### 3.1.38. 4-(5-Bromo-1*H*-indol-3-yl)-*N*-(2-fluorophenyl) pyrimidin-2-amine **B1**

Yield: 25%, pale yellow solid, mp 206.6–207.1 °C. ^1^H NMR (400 MHz, DMSO-*d*_6_) *δ* 11.88 (s, 1H), 8.96 (s, 1H), 8.43 (s, 1H), 8.29 (s, 1H), 8.20 (d, *J* = 5.3 Hz, 1H), 7.70–7.63 (m, 1H), 7.32 (d, *J* = 8.5 Hz, 1H), 7.25–7.08 (m, 5H). ^13^C NMR (100 MHz, DMSO-*d*_6_) *δ* 162.71, 161.10, 157.53, 157.22, 154.78, 136.21, 130.77, 128.25 (d, *J* = 11.4 Hz), 127.32, 126.46, 125.47 (d, *J* = 7.3 Hz), 125.25, 124.87, 124.78 (d, *J* = 4.0 Hz), 116.29 (d, *J* = 19.8 Hz), 114.13 (d, *J* = 29.7 Hz), 113.58, 107.68. HRMS calcd for C₁₈H₁₃BrFN₄^+^ [M + H] ^+^383.0302, found 383.0301. HPLC purity 99.29% (t_R_ = 14.1 min).

#### 3.1.39. 4-(5-Bromo-1*H*-indol-3-yl)-*N*-(3-fluorophenyl) pyrimidin-2-amine **B2**

Yield: 48%, pale yellow solid, mp 211.8–212.4 °C. ^1^H NMR (400 MHz, DMSO-*d*_6_) *δ* 11.93 (s, 1H), 9.64 (s, 1H), 8.67 (s, 1H), 8.29 (s, 2H), 7.72 (d, *J* = 12.4 Hz, 1H), 7.49 (d, *J* = 8.2 Hz, 1H), 7.35 (d, *J* = 8.6 Hz, 1H), 7.29–7.19 (m, 3H), 6.66 (t, *J* = 8.4 Hz, 1H). ^13^C NMR (100 MHz, DMSO-*d*_6_) *δ* 164.12, 162.67, 161.73, 160.28, 157.42, 143.20 (d, *J* = 11.4 Hz), 136.35, 130.99, 130.44 (d, *J* = 9.7 Hz), 127.26, 125.39, 124.75, 115.27, 114.33 (d, *J* = 29.6 Hz), 113.62, 108.46, 107.85 (d, *J* = 21.1 Hz), 106.01 (d, *J* = 26.4 Hz). HRMS calcd for C₁₈H₁₃BrFN₄^+^ [M + H] ^+^383.0302, found 383.0295. HPLC purity 98.07% (t_R_ = 14.2 min).

#### 3.1.40. 4-(5-Bromo-1*H*-indol-3-yl)-*N*-(4-fluorophenyl) pyrimidin-2-amine **B3**

Yield: 41%, pale yellow solid, mp 246.8–247.7 °C. ^1^H NMR (400 MHz, DMSO-*d*_6_) *δ* 12.02 (s, 1H), 9.44 (s, 1H), 8.65 (s, 1H), 8.34 (s, 1H), 8.27 (d, *J* = 5.3 Hz, 1H), 7.75–7.70 (m, 2H), 7.39 (d, *J* = 8.6 Hz, 1H), 7.26 (d, *J* = 9.0 Hz, 1H), 7.23 (d, *J* = 5.3 Hz, 1H), 7.13 (t, *J* = 8.7 Hz, 2H). ^13^C NMR (100 MHz, DMSO-*d*_6_) *δ* 162.53, 161.51, 159.55, 157.77, 156.42, 155.41, 136.45, 135.25, 129.84, 126.20, 123.96 (d, *J* = 46.4 Hz), 120.75 (d, *J* = 7.5 Hz), 114.47 (d, *J* = 22.2 Hz), 113.39, 112.76 (d, *J* = 37.2 Hz), 106.71. HRMS calcd for C₁₈H₁₃BrFN₄^+^ [M + H] ^+^383.0302, found 383.0296 HPLC purity 96.24% (t_R_ = 14.1 min).

#### 3.1.41. 4-(5-Bromo-1*H*-indol-3-yl)-*N*-(2-chlorophenyl) pyrimidin-2-amine **B4**

Yield: 41%, pale yellow solid, mp 173.8–174.6 °C. ^1^H NMR (400 MHz, DMSO-*d*_6_) *δ* 11.94 (s, 1H), 8.74 (s, 1H), 8.41 (s, 1H), 8.36 (d, *J* = 2.7 Hz, 1H), 8.29 (d, *J* = 5.4 Hz, 1H), 7.83 (d, *J* = 8.3 Hz, 1H), 7.55 (d, *J* = 8.0 Hz, 1H), 7.43 (d, *J* = 7.8 Hz, 1H), 7.39 (d, *J* = 8.3 Hz, 1H), 7.26 (t, *J* = 6.8 Hz, 2H), 7.21 (d, *J* = 7.6 Hz, 1H). ^13^C NMR (100 MHz, DMSO-*d*_6_) *δ* 162.65, 161.04, 157.64, 137.31, 136.19, 130.82, 130.15, 128.30, 128.06, 127.30, 126.98, 125.99, 125.24, 124.85, 114.29, 113.96, 113.52, 107.76. HRMS calcd for C₁₈H₁₃BrFN₄^+^ [M + H] ^+^399.0007, found 399. 0002.HPLC purity 98.97% (t_R_ = 15.7 min).

#### 3.1.42. 4-(5-Bromo-1*H*-indol-3-yl)-*N*-(3-chlorophenyl) pyrimidin-2-amine **B5**

Yield: 38%, pale yellow solid, mp 210.3–211.4 °C. ^1^H NMR (400 MHz, DMSO-*d*_6_) *δ* 12.23 (s, 1H), 9.69 (s, 1H), 8.73 (s, 1H), 8.40 (s, 1H), 8.38 (d, *J* = 5.4 Hz, 1H), 8.10 (s, 1H), 7.83 (d, *J* = 8.1 Hz, 1H), 7.45 (d, *J* = 8.6 Hz, 1H), 7.35–7.30 (m, 2H), 7.28 (d, *J* = 8.1 Hz, 1H), 7.13 (d, *J* = 7.9 Hz, 1H). ^13^C NMR (100 MHz, DMSO-*d*_6_) *δ* 161.83, 159.32, 156.51, 142.18, 135.47, 130.14, 130.03, 126.40, 124.53, 123.87, 123.14, 121.21, 120.66, 117.35, 113.59, 113.33, 112.70, 107.64. HRMS calcd for C₁₈H₁₃BrClN₄^+^ [M + H] ^+^399.0007, found 399.0000. HPLC purity 96.15% (t_R_ = 14.4 min).

#### 3.1.43. 4-(5-Bromo-1*H*-indol-3-yl)-*N*-(4-chlorophenyl) pyrimidin-2-amine **B6**

Yield: 43%, pale yellow solid, mp 273.5–274.8 °C. ^1^H NMR (400 MHz, DMSO-*d*_6_) *δ* 12.28 (s, 1H), 9.58 (s, 1H), 8.66 (s, 1H), 8.36 (s, 1H), 8.28 (d, *J* = 5.3 Hz, 1H), 7.77 (d, *J* = 8.8 Hz, 2H), 7.40 (d, *J* = 8.6 Hz, 1H), 7.31 (d, *J* = 8.8 Hz, 2H), 7.28–7.23 (m, 2H). ^13^C NMR (100 MHz, DMSO-*d*_6_) *δ* 162.15, 159.93, 157.03, 139.74, 135.88, 130.59, 128.42, 126.81, 124.87, 124.80, 124.33, 120.94, 114.03, 113.62, 113.15, 107.69. HRMS calcd for C₁₈H₁₃BrClN₄^+^, [M + H] ^+^399.0007, found 399.0005. HPLC purity 95.20% (t_R_ = 15.4 min).

#### 3.1.44. 4-(5-Bromo-1*H*-indol-3-yl)-*N*-(2-bromophenyl) pyrimidin-2-amine **B7**

Yield: 37%, pale yellow solid, mp 177.8–178.4 °C. ^1^H NMR (400 MHz, DMSO-*d*_6_) *δ* 11.96 (s, 1H), 8.71 (s, 1H), 8.39–8.36 (m, 2H), 8.28 (d, *J* = 5.3 Hz, 1H), 7.80 (d, *J* = 8.0 Hz, 1H), 7.72 (d, *J* = 8.0 Hz, 1H), 7.46 (t, *J* = 7.6 Hz, 1H), 7.39 (d, *J* = 8.6 Hz, 1H), 7.29–7.23 (m, 2H), 7.15 (t, *J* = 7.7 Hz, 1H). ^13^C NMR (100 MHz, DMSO-*d*_6_) *δ* 162.63, 161.06, 157.65, 138.64, 136.18, 133.31, 130.88, 130.80, 128.71, 127.30, 126.53, 125.22, 124.86, 119.55, 114.27, 113.94, 113.52, 107.70. HRMS calcd for C₁₈H₁₃Br₂N₄^+^ [M + H] ^+^442.9501, found 442.9504. HPLC purity 98.68% (t_R_ = 15.9 min).

#### 3.1.45. 4-(5-Bromo-1*H*-indol-3-yl)-*N*-(3-bromophenyl) pyrimidin-2-amine **B8**

Yield: 48%, pale yellow solid, mp 214.8–215.6 °C. ^1^H NMR (400 MHz, DMSO-*d*_6_) *δ* 12.04 (s, 1H), 9.71 (s, 1H), 8.73 (d, *J* = 2.0 Hz, 1H), 8.40 (d, *J* = 3.9 Hz, 1H), 8.38 (s, 1H), 7.99 (s, 1H), 7.79–7.71 (m, 1H), 7.45 (d, *J* = 8.6 Hz, 1H), 7.36 (d, *J* = 8.4 Hz, 1H), 7.34–7.30 (m, 2H), 7.02–6.96 (m, 1H).^13^C NMR (100 MHz, DMSO-*d*_6_) *δ* 161.82, 159.34, 156.53, 142.01, 135.46, 132.64, 130.15, 129.71, 126.38, 124.52, 123.85, 120.24, 117.80, 116.97, 113.59, 113.30, 112.70, 107.64. HRMS calcd for C₁₈H₁₃Br₂N₄^+^ [M + H] ^+^442.9501, found 442.9498. HPLC purity 98.57% (t_R_ = 15.1 min).

#### 3.1.46. 4-(5-Bromo-1*H*-indol-3-yl)-*N*-(4-bromophenyl) pyrimidin-2-amine **B9**

Yield: 36%, pale yellow solid, mp 276.7–277.5 °C. ^1^H NMR (400 MHz, DMSO-*d*_6_) *δ* 12.22 (s, 1H), 9.55 (s, 1H), 8.63 (s, 1H), 8.32 (s, 1H), 8.25 (d, *J* = 5.4 Hz, 1H), 7.69 (d, *J* = 8.5 Hz, 2H), 7.44–7.33 (m, 3H), 7.24–7.20 (m, 2H).^13^C NMR (100 MHz, DMSO-*d*_6_) *δ* 161.57, 159.28, 156.39, 139.56, 135.31, 130.68, 129.99, 126.18, 124.18, 123.70, 120.78, 113.48, 112.98, 112.42, 112.07, 107.12. HRMS calcd for C₁₈H₁₃Br₂N₄^+^, [M + H] ^+^ 442.9501, found 442.9502. HPLC purity 95.11% (t_R_ = 15.7 min).

#### 3.1.47. 4-(5-Bromo-1*H*-indol-3-yl)-*N*-(2-(trifluoromethyl) phenyl) pyrimidin-2-amine **B10**

Yield: 40%, pale yellow solid, mp 150.5–151.3 °C. ^1^H NMR (400 MHz, DMSO-*d*_6_) *δ* 12.10 (s, 1H), 8.68 (s, 1H), 8.34 (s, 1H), 8.26 (d, *J* = 5.3 Hz, 1H), 8.21 (s, 1H), 7.78 (d, *J* = 8.2 Hz, 3H), 7.47 (d, *J* = 7.6 Hz, 1H), 7.39 (d, *J* = 8.6 Hz, 1H), 7.28–7.21 (m, 2H). ^13^C NMR (100 MHz, DMSO-*d*_6_) *δ* 162.51, 161.89, 157.49, 138.26, 136.06, 133.55, 130.58, 130.00, 127.18, 126.75 (q, *J* = 5.3 Hz), 126.07, 125.74 (q, *J* = 272 Hz), 125.04, 124.70 (d, *J* = 5.7 Hz), 124.44, 114.15, 113.76, 113.29, 107.41. HRMS calcd for C₁₉H₁₃BrF₃N₄^+^, [M + H] ^+^433.0270, found 433.0271. HPLC purity 95.40% (t_R_ = 14.7 min).

#### 3.1.48. 4-(5-Bromo-1*H*-indol-3-yl)-*N*-(3-(trifluoromethyl) phenyl) pyrimidin-2-amine **B11**

Yield: 45%, pale yellow solid, mp 171.8–172.3 °C. ^1^H NMR (400 MHz, DMSO-*d*_6_) *δ* 12.02 (s, 1H), 9.82 (s, 1H), 8.72 (s, 1H), 8.41 (d, *J* = 5.2 Hz, 2H), 8.22 (s, 1H), 8.15 (d, *J* = 8.4 Hz, 1H), 7.56 (t, *J* = 8.0 Hz, 1H), 7.45 (d, *J* = 8.9 Hz, 1H), 7.35 (t, *J* = 4.9 Hz, 2H), 7.32-7.27 (m, 1H). ^13^C NMR (100 MHz, DMSO-*d*_6_) *δ* 162.31, 159.80, 156.97, 141.74, 135.93, 130.58, 129.93 (q, *J* = 29.3 Hz), 129.00, 126.83, 125.79 (q, *J* = 272.7 Hz) 124.97, 124.27, 122.45, 117.22, 114.90, 114.04, 113.76, 113.12, 108.24. HRMS calcd for C₁₉H₁₃BrF₃N₄^+^, [M + H] ^+^433.0270, found 433.0278. HPLC purity 99.39% (t_R_ = 15.1 min).

#### 3.1.49. 4-(5-Bromo-1*H*-indol-3-yl)-*N*-(4-(trifluoromethyl) phenyl) pyrimidin-2-amine **B12**

Yield: 34%, pale yellow solid, mp 279.5–280.3 °C. ^1^H NMR (400 MHz, DMSO-*d*_6_) *δ* 12.02 (s, 1H), 9.91 (s, 1H), 8.76 (s, 1H), 8.43 (d, *J* = 3.0 Hz, 1H), 8.41 (d, *J* = 5.4 Hz, 1H), 8.02 (d, *J* = 8.5 Hz, 2H), 7.68 (d, *J* = 8.5 Hz, 2H), 7.45 (d, *J* = 8.6 Hz, 1H), 7.38 (d, *J* = 5.4 Hz, 1H), 7.35–7.31 (m, 1H). ^13^C NMR (100 MHz, DMSO-*d*_6_) *δ* 162.25, 159.72, 157.06, 144.51, 135.94, 130.76, 126.81, 125.87 (d, *J* = 4.1 Hz), 124.92, 124.32, 123.47 (q, *J* = 272.7 Hz), 120.95 (q, *J* = 31.1 Hz), 118.75, 114.08, 113.66, 113.09, 108.36. HRMS calcd for C₁₉H₁₃BrF₃N₄^+^ [M + H] ^+^433.0270, found 433.0273. HPLC purity 97.41% (t_R_ = 15.3 min).

#### 3.1.50. 4-(5-Bromo-1*H*-indol-3-yl)-*N*-(o-tolyl) pyrimidin-2-amine **B13**

Yield: 39%, pale yellow solid, mp 187.6–188.4 °C. ^1^H NMR (400 MHz, DMSO-*d*_6_) *δ* 11.86 (s, 1H), 8.65 (s, 1H), 8.39–8.06 (m, 3H), 7.38 (d, *J* = 57.8 Hz, 2H), 7.12 (d, *J* = 59.2 Hz, 5H), 2.20 (s, 3H). ^13^C NMR (100 MHz, DMSO-*d*_6_) *δ* 162.58, 161.66, 157.61, 138.73, 136.16, 134.54, 132.91, 131.01, 130.56, 127.34, 126.65, 125.85, 125.21, 124.93, 114.22, 113.94, 113.72, 106.80, 18.80. HRMS calcd for C₁₉H₁₆BrN₄^+^ [M + H] ^+^379.0553, found 379.0561. HPLC purity 95.08% (t_R_ = 14.4 min).

#### 3.1.51. 4-(5-Bromo-1*H*-indol-3-yl)-*N*-(m-tolyl) pyrimidin-2-amine **B14**

Yield: 52%, pale yellow solid, mp 141.5–142.3 °C. ^1^H NMR (400 MHz, DMSO-*d*_6_) *δ* 12.00 (s, 1H), 9.42 (s, 1H), 8.76 (s, 1H), 8.39 (s, 1H), 8.34 (d, *J* = 5.3 Hz, 1H), 7.63 (d, *J* = 7.1 Hz, 2H), 7.44 (d, *J* = 8.6 Hz, 1H), 7.34–7.30 (m, 1H), 7.27 (d, *J* = 5.4 Hz, 1H), 7.25–7.19 (m, 1H), 6.80 (d, *J* = 7.5 Hz, 1H), 2.31 (s, 3H).^13^C NMR (100 MHz, DMSO-*d*_6_) *δ* 162.47, 160.58, 157.42, 141.10, 138.02, 136.28, 130.76, 128.84, 127.27, 125.28, 124.77, 122.49, 120.16, 116.97, 114.38, 114.00, 113.76, 107.69, 21.90. HRMS calcd for C₁₉H₁₆BrN₄^+^ [M + H] ^+^ 379.0553, found 379.0547. HPLC purity 97.81% (t_R_ = 14.7 min).

#### 3.1.52. 4-(5-Bromo-1*H*-indol-3-yl)-*N*-(p-tolyl) pyrimidin-2-amine **B15**

Yield: 41%, pale yellow solid, mp 227.8–228.6 °C. ^1^H NMR (400 MHz, DMSO-*d*_6_) *δ* 12.06 (s, 1H), 9.36 (s, 1H), 8.74 (s, 1H), 8.34 (d, *J* = 30.0 Hz, 2H), 7.71–7.59 (m, 2H), 7.42 (s, 1H), 7.28 (d, *J* = 29.2 Hz, 2H), 7.21–7.11 (m, 2H), 2.28 (s, 3H) ^13^C NMR (100 MHz, DMSO-*d*_6_) *δ* 162.47, 160.73, 157.43, 138.52, 136.28, 130.78, 130.63, 129.46, 127.24, 125.22, 124.84, 120.24, 114.38, 113.96, 113.71, 107.41, 20.97. HRMS calcd for C₁₉H₁₆BrN₄^+^ [M + H] ^+^379.0553, found 379.0552. HPLC purity 96.65% (t_R_ = 14.8 min).

#### 3.1.53. 4-(5-Bromo-1*H*-indol-3-yl)-*N*-(2-methoxyphenyl) pyrimidin-2-amine **B16**

Yield: 36%, pale yellow solid, mp 207.9–208.3 °C. ^1^H NMR (400 MHz, DMSO-*d*_6_) *δ* 11.97 (s, 1H), 8.58 (s, 1H), 8.36 (s, 1H), 8.29 (d, *J* = 5.4 Hz, 1H), 8.10–8.05 (m, 2H), 7.40 (d, *J* = 8.5 Hz, 1H), 7.31–7.24 (m, 2H), 7.12–6.98 (m, 3H), 3.84 (s, 3H). ^13^C NMR (100 MHz, DMSO-*d*_6_) *δ* 162.77, 160.89, 157.80, 150.46, 136.45, 131.11, 129.35, 127.42, 125.41, 124.83, 123.74, 122.18, 121.13, 114.61, 114.15, 113.79, 111.72, 107.96, 56.31. HRMS calcd for C₁₉H₁₆BrN₄O^+^ [M + H] ^+^395.0502, found 395.0502. HPLC purity 98.24% (t_R_ = 15.0 min).

#### 3.1.54. 4-(5-Bromo-1*H*-indol-3-yl)-*N*-(3-methoxyphenyl) pyrimidin-2-amine **B17**

Yield: 49%, pale yellow solid, mp 208.7–209.3 °C. ^1^H NMR (400 MHz, DMSO-*d*_6_) *δ* 11.96 (s, 1H), 9.44 (s, 1H), 8.73 (s, 1H), 8.34 (s, 1H), 8.30 (d, *J* = 5.3 Hz, 1H), 7.39 (d, *J* = 8.7 Hz, 3H), 7.27 (d, *J* = 8.6 Hz, 1H), 7.23 (d, *J* = 5.4 Hz, 1H), 7.19 (t, *J* = 8.1 Hz, 1H), 6.51 (d, *J* = 8.0 Hz, 1H), 3.68 (s, 3H). ^13^C NMR (100 MHz, DMSO-*d*_6_) *δ* 161.69, 159.67, 159.22, 156.56, 141.57, 135.45, 129.97, 128.87, 126.40, 124.47, 124.01, 113.54, 113.26, 112.88, 111.32, 107.07, 106.16, 104.64, 54.45. HRMS calcd for C₁₉H₁₆BrN₄O^+^ [M + H] ^+^395.0502, found 395.0498. HPLC purity 97.43% (t_R_ = 13.6 min).

#### 3.1.55. 4-(5-Bromo-1*H*-indol-3-yl)-*N*-(4-methoxyphenyl) pyrimidin-2-amine **B18**

Yield: 47%, pale yellow solid, mp 245.7–246.3 °C.^1^H NMR (400 MHz, DMSO-*d*_6_) *δ* 11.96 (s, 1H), 9.24 (s, 1H), 8.72 (s, 1H), 8.38 (d, *J* = 2.8 Hz, 1H), 8.30 (d, *J* = 5.3 Hz, 1H), 7.66 (d, *J* = 8.6 Hz, 2H), 7.44 (d, *J* = 8.5 Hz, 1H), 7.32 (d, *J* = 8.7 Hz, 1H), 7.23 (d, *J* = 5.3 Hz, 1H), 6.96 (d, *J* = 8.6 Hz, 2H), 3.77 (s, 3H). ^13^C NMR (100 MHz, DMSO-*d*_6_) *δ* 162.41, 160.86, 157.46, 154.73, 136.24, 134.11, 130.68, 127.25, 125.18, 124.84, 122.00, 114.32, 114.26, 113.91, 113.77, 107.09, 55.60. HRMS calcd for C₁₉H₁₆BrN₄O^+^ [M + H] ^+^395.0502, found 395.0497. HPLC purity 95.61% (t_R_ = 13.4 min).

#### 3.1.56. 4-(5-Bromo-1*H*-indol-3-yl)-*N*-(2-isopropylphenyl) pyrimidin-2-amine **B19**

Yield: 41%, pale yellow solid, mp 110.5–111.3 °C. ^1^H NMR (400 MHz, DMSO-*d*_6_) *δ* 11.88 (s, 1H), 8.70 (d, *J* = 3.8 Hz, 1H), 8.29 (s, 1H), 8.21 (d, *J* = 17.1 Hz, 2H), 7.41–7.30 (m, 3H), 7.29–7.20 (m, 3H), 7.13 (t, *J* = 4.6 Hz, 1H), 3.26 (q, *J* = 6.4, 5.7 Hz, 1H), 1.12 (d, *J* = 3.1 Hz, 6H). ^13^C NMR (100 MHz, DMSO-*d*_6_) *δ* 162.08, 162.02, 157.01, 144.29, 136.77, 135.53, 129.74, 127.55, 126.76, 125.92, 125.88, 125.61, 124.62, 124.55, 113.53, 113.27, 113.15, 105.81, 27.28, 23.10. HRMS calcd for C₂₁H₂₀BrN₄^+^ [M + H] ^+^407.0866, found 407.0863. HPLC purity 98.51% (t_R_ = 15.1 min).

#### 3.1.57. 4-(5-Bromo-1*H*-indol-3-yl)-*N*-(3-isopropylphenyl) pyrimidin-2-amine **B20**

Yield: 60%, pale yellow solid, mp 152.6–153.4 °C.^1^H NMR (400 MHz, DMSO-*d*_6_) *δ* 11.92 (s, 1H), 9.33 (s, 1H), 8.68 (d, *J* = 4.6 Hz, 1H), 8.32–8.23 (m, 2H), 7.70 (t, *J* = 6.1 Hz, 1H), 7.45 (d, *J* = 4.6 Hz, 1H), 7.39–7.34 (m, 1H), 7.25–7.15 (m, 3H), 6.77 (t, *J* = 6.2 Hz, 1H), 2.77 (q, *J* = 6.8 Hz, 1H), 1.13 (d, *J* = 6.1 Hz, 6H). ^13^C NMR (100 MHz, DMSO-*d*_6_) *δ* 162.33, 160.45, 157.22, 148.95, 140.94, 136.11, 130.56, 128.71, 127.07, 125.11, 124.58, 119.58, 117.56, 117.19, 114.21, 113.86, 113.58, 107.54, 33.85, 24.25. HRMS calcd for C₂₁H₂₀BrN₄^+^ [M + H] ^+^407.0866, found 407.0866. HPLC purity 98.50% (t_R_ = 15.8 min).

#### 3.1.58. 4-(5-Bromo-1*H*-indol-3-yl)-*N*-(4-isopropylphenyl) pyrimidin-2-amine **B21**

Yield: 41%, pale yellow solid, mp 218.7–219.4 °C. ^1^H NMR (400 MHz, DMSO-*d*_6_) *δ* 11.88 (s, 1H), 9.26 (s, 1H), 8.67 (s, 1H), 8.27 (s, 1H), 8.19 (d, *J* = 5.3 Hz, 1H), 7.56 (d, *J* = 8.2 Hz, 2H), 7.33 (d, *J* = 8.6 Hz, 1H), 7.20 (d, *J* = 8.6 Hz, 1H), 7.15–7.10 (m, 3H), 2.78–2.72 (m, 1H), 1.10 (d, *J* = 6.9 Hz, 6H). ^13^C NMR (100 MHz, DMSO-*d*_6_) *δ* 161.88, 160.12, 156.93, 141.25, 138.17, 135.72, 130.22, 126.67, 126.22, 124.66, 124.28, 119.63, 113.83, 113.38, 113.22, 106.85, 32.78, 23.99. HRMS calcd for C₂₁H₂₀BrN₄^+^ [M + H] ^+^407.0866, found 407.0870. HPLC purity 98.97% (t_R_ = 16.3 min).

#### 3.1.59. 4-(5-Bromo-1*H*-indol-3-yl)-*N*-phenylpyrimidin-2-amine **B22**

Yield: 46%, pale yellow solid, mp 197.6–198.7 °C. ^1^H NMR (400 MHz, DMSO-*d*_6_) *δ* 12.06 (s, 1H), 9.46 (s, 1H), 8.77 (s, 1H), 8.37 (s, 1H), 8.33 (d, *J* = 5.3 Hz, 1H), 7.79 (d, *J* = 8.0 Hz, 2H), 7.45 (d, *J* = 8.6 Hz, 1H), 7.33 (q, *J* = 9.4, 8.5 Hz, 3H), 7.27 (d, *J* = 5.3 Hz, 1H), 6.98 (t, *J* = 7.3 Hz, 1H). ^13^C NMR (100 MHz, DMSO-*d*_6_) *δ* 162.36, 160.38, 157.23, 140.98, 136.21, 130.81, 128.82, 127.06, 124.97, 124.55, 121.53, 119.60, 114.31, 113.78, 113.42, 107.56. HRMS calcd for C₁₈H₁₄BrN₄^+^ [M + H] ^+^365.0396, found 365.0397. HPLC purity 98.81% (t_R_ = 10.5 min).

#### 3.1.60. 4-(5-Bromo-1*H*-indol-3-yl)-*N*-(3,5-difluorophenyl) pyrimidin-2-amine **B23**

Yield: 52%, pale yellow solid, mp 251.3–252.2 °C. ^1^H NMR (400 MHz, DMSO-*d*_6_) *δ* 12.06 (s, 1H), 9.92 (s, 1H), 8.73 (d, *J* = 2.0 Hz, 1H), 8.52–8.24 (m, 2H), 7.63–7.58 (m, 2H), 7.46 (d, *J* = 8.6 Hz, 1H), 7.38 (d, *J* = 5.4 Hz, 1H), 7.35–7.32 (m, 1H), 6.74 (t, *J* = 9.3 Hz, 1H).^13^C NMR (100 MHz, DMSO-*d*_6_) *δ* 163.86 (d, *J* = 16.1 Hz), 162.34, 161.47 (d, *J* = 16.0 Hz), 159.54, 156.97, 143.53 (d, *J* = 14.6 Hz), 135.92, 130.73, 126.78, 125.01, 124.17, 114.10, 113.79, 113.02, 108.62, 101.42 (d, *J* = 8.1 Hz). HRMS calcd for C₁₈H₁₂BrF₂N₄^+^ [M + H] ^+^ 401.0208, found 401.0201 HPLC purity 96.39% (t_R_ = 14.7 min).

#### 3.1.61. 4-(5-Bromo-1*H*-indol-3-yl)-*N*-(3,5-dichlorophenyl) pyrimidin-2-amine **B24**

Yield: 63%, pale yellow solid, mp 223.6–224.3 °C. ^1^H NMR (400 MHz, DMSO-*d*_6_) *δ* 12.02 (s, 1H), 9.84 (s, 1H), 8.66 (d, *J* = 2.0 Hz, 1H), 8.39 (s, 1H), 8.37 (d, *J* = 3.0 Hz, 1H), 7.91 (d, *J* = 1.9 Hz, 2H), 7.41 (d, *J* = 8.6 Hz, 1H), 7.34 (d, *J* = 5.4 Hz, 1H), 7.31–7.27 (m, 1H), 7.06 (t, *J* = 1.8 Hz, 1H).^13^C NMR (100 MHz, DMSO-*d*_6_) *δ* 162.82, 159.84, 157.34, 143.86, 136.33, 134.40, 131.13, 127.23, 125.49, 124.60, 120.30, 117.01, 114.50, 114.27, 113.39, 109.14. HRMS calcd for C₁₈H₁₂BrCl₂N₄^+^ [M + H] ^+^432.9617, found 432. 9611. HPLC purity 97.08% (t_R_ = 16.7 min).

#### 3.1.62. 4-(5-Bromo-1*H*-indol-3-yl)-*N*-(3,5-dibromophenyl) pyrimidin-2-amine **B25**

Yield: 48%, pale yellow solid, mp 193.4–193.7 °C. ^1^H NMR (400 MHz, DMSO-*d*_6_) *δ* 11.91 (s, 1H), 9.68 (s, 1H), 8.54 (s, 1H), 8.29–8.23 (m, 2H), 7.98–7.94 (m, 2H), 7.30 (d, *J* = 8.5 Hz, 1H), 7.21 (d, *J* = 5.4 Hz, 1H), 7.18 (d, *J* = 4.9 Hz, 2H). ^13^C NMR (100 MHz, DMSO-*d*_6_) *δ* 162.28, 159.25, 156.82, 143.68, 135.80, 130.53, 126.69, 125.08, 125.00, 124.09, 122.23, 119.67, 113.99, 113.79, 112.85, 108.61. HRMS calcd for C₁₈H₁₄Br₃N₄^+^ [M + H] ^+^522.8763, found 522.8587. HPLC purity 97.05% (t_R_ = 17.3 min).

#### 3.1.63. *N*-(3,5-bis(trifluoromethyl)phenyl)-4-(5-bromo-1*H*-indol-3-yl) pyrimidin-2-amine **B26**

Yield: 59%, pale yellow solid, mp 220.9–221.3 °C.^1^H NMR (400 MHz, DMSO-*d*_6_) *δ* 12.06 (s, 1H), 10.17 (s, 1H), 8.68 (s, 1H), 8.55 (s, 2H), 8.45 (d, *J* = 5.4 Hz, 1H), 8.40 (s, 1H), 7.56 (s, 1H), 7.42 (t, *J* = 7.8 Hz, 2H), 7.31 (d, *J* = 7.6 Hz, 1H). ^13^C NMR (100 MHz, DMSO-*d*_6_) *δ* 162.54, 159.37, 156.90, 142.96, 135.93, 130.93 (d, *J* = 34.2 Hz), 130.30 (d, *J* = 32.3 Hz), 126.79, 125.08, 124.94 (q, *J* = 273.7 Hz),124.12, 118.04, 114.09, 113.84, 113.17, 112.88, 109.08. HRMS calcd for C₂₀H₁₂BrF₆N₄^+^, [M + H] ^+^501.0144, found 501.0138. HPLC purity 97.89% (t_R_ = 16.1 min).

#### 3.1.64. 4-(5-Bromo-1*H*-indol-3-yl)-*N*-(3, 5-dimethylphenyl) pyrimidin-2-amine **B27**

Yield: 42%, pale yellow solid, mp 184.6–185.3 °C. ^1^H NMR (400 MHz, DMSO-*d*_6_) *δ* 11.94 (s, 1H), 9.29 (s, 1H), 8.66 (s, 1H), 8.30 (d, *J* = 16.5 Hz, 2H), 7.38 (s, 3H), 7.23 (d, *J* = 30.4 Hz, 2H), 6.55 (s, 1H), 2.21 (s, 6H). ^13^C NMR (100 MHz, DMSO-*d*_6_) *δ* 161.84, 159.96, 156.81, 140.44, 137.19, 135.67, 130.06, 126.70, 124.71, 124.15, 122.78, 116.73, 113.77, 113.37, 113.20, 107.03, 21.21. HRMS calcd for C₂₀H₁₈BrN₄^+^ [M + H] ^+^393.0709, found 393.0712 HPLC purity 97.05% (t_R_ = 15.3 min).

#### 3.1.65. 4-(5-Bromo-1*H*-indol-3-yl)-*N*-(3, 5-dimethoxyphenyl) pyrimidin-2-amine **B28**

Yield: 48%, pale yellow solid, mp 255.6–256.8 °C. ^1^H NMR (400 MHz, DMSO-*d*_6_) *δ* 11.96 (s, 1H), 9.40 (s, 1H), 8.71 (s, 1H), 8.33 (d, *J* = 2.5 Hz, 1H), 8.30 (d, *J* = 5.3 Hz, 1H), 7.39 (d, *J* = 8.6 Hz, 1H), 7.29–7.25 (m, 1H), 7.23 (d, *J* = 5.4 Hz, 1H), 7.04 (d, *J* = 2.2 Hz, 2H), 6.09 (s, 1H), 3.67 (s, 6H).^13^C NMR (100 MHz, DMSO-*d*_6_) *δ* 162.53, 160.96, 160.46, 157.37, 142.89, 136.29, 130.76, 127.25, 125.37, 124.87, 114.39, 114.16, 113.70, 108.04, 97.92, 93.74, 55.40. HRMS calcd for C₂₀H₁₈BrN₄O₂^+^ [M + H] ^+^425.0608, found 425.0601. HPLC purity 96.76% (t_R_ = 13.3 min).

#### 3.1.66. 4-(5-Bromo-1*H*-indol-3-yl)-*N*-(2,6-difluorophenyl) pyrimidin-2-amine **B29**

Yield: 40%, pale yellow solid, mp 263.3–264.2 °C. ^1^H NMR (400 MHz, DMSO-*d*_6_) *δ* 11.87 (s, 1H), 8.91 (s, 1H), 8.23 (d, *J* = 40.8 Hz, 3H), 7.53–7.01 (m, 7H). ^13^C NMR (100 MHz, DMSO-*d*_6_) *δ* 162.85, 161.48, 160.29 (d, *J* = 5.2 Hz), 157.83 (d, *J* = 5.1 Hz), 157.57, 136.15, 130.69, 127.36, 125.23, 124.72, 114.23, 113.91, 113.41, 112.50 (d, *J* = 5.9 Hz), 112.32 (d, *J* = 5.2 Hz), 107.43. HRMS calcd for C₁₈H₁₂BrF₂N₄^+^, [M + H] ^+^401.0208, found 401.0201. HPLC purity 97.49% (t_R_ = 12.4 min).

#### 3.1.67. 4-(5-Bromo-1*H*-indol-3-yl)-*N*-(2,6-dimethylphenyl) pyrimidin-2-amine **B30**

Yield: 34%, pale yellow solid, mp 228.5–229.4 °C.^1^H NMR (400 MHz, DMSO-*d*_6_) *δ* 11.85 (s, 1H), 8.54 (s, 1H), 8.24 (d, *J* = 38.1 Hz, 2H), 7.82 (s, 1H), 7.33 (s, 1H), 7.25–6.98 (m, 5H), 2.20 (s, 6H). ^13^C NMR (100 MHz, DMSO-*d*_6_) *δ* 162.27, 161.60, 157.27, 148.03, 137.14, 135.62, 129.80, 128.05, 126.99, 126.08, 124.73, 124.44, 113.64, 113.40, 113.32, 105.52, 18.58. HRMS calcd for C₂₀H₁₈BrN₄^+^ [M + H] ^+^393.0709, found 393.0715. HPLC purity 96.56% (t_R_ = 14.2 min).

#### 3.1.68. 4-(5-Bromo-1*H*-indol-3-yl)-*N*-(2,6-dimethoxyphenyl) pyrimidin-2-amine **B31**

Yield: 62%, pale yellow solid, mp 244.3–245.6 °C. ^1^H NMR (400 MHz, DMSO-*d*_6_) *δ* 11.74 (s, 1H), 8.15 (s, 1H), 8.01 (d, *J* = 5.3 Hz, 1H), 7.92 (s, 1H), 7.25 (d, *J* = 8.5 Hz, 1H), 7.15–7.09 (m, 2H), 6.95 (d, *J* = 5.3 Hz, 1H), 6.65 (d, *J* = 8.4 Hz, 2H), 3.60 (s, 6H). ^13^C NMR (100 MHz, DMSO-*d*_6_) *δ* 162.50, 162.37, 157.25, 156.70, 136.12, 130.09, 127.49, 127.38, 125.20, 125.04, 117.15, 114.14, 113.93, 113.82, 106.08, 105.16, 56.00. HRMS calcd for C₂₀H₁₈BrN₄O₂^+^ [M + H] ^+^425.0608, found 425.0600. HPLC purity 97.85% (t_R_ = 12.2 min).

### 3.2. Biological Evaluation

#### 3.2.1. General Procedure

The hepatocyte cell line HepG2 cells obtained from the Type Culture Collection of the Chinese Academy of Sciences (Shanghai, China) were maintained in high-glucose DMEM (Gibco) supplemented with 10% fetal bovine serum (FBS) and 1% penicillin-streptomycin in an atmosphere of 5% CO_2_ at 37 °C.

#### 3.2.2. MTT Assay for Cell Viability

Into a 96-well plate, 1 × 10^3^ HepG2 cells per well were seeded. After 24 h, stock solutions of test compounds in DMSO were diluted in DMEM to ≤0.1% final DMSO concentration. Cells were treated with various concentrations (2.5–25 µM) of tested compounds for 48 h, and the MTT solution (0.5 mg/mL) was added. After 4 h of incubation at 37 °C for MTT-formazan formation, the supernatant was discarded and 100 µL of DMSO was applied into each well to dissolve formazan crystals. Absorbance at 490 nm was determined spectrophotometrically by using a microplate reader (Epoch, BioTek Instruments, Inc., Winooski, VT, USA).

#### 3.2.3. Inhibition of GSK-3β

The activity of recombinant GSK-3β (Invitrogen) was measured in a Z-lyte assay of Invitrogen. In this assay, 100 nL of the compound solution in DMSO was transferred to a 384-well assay plate (the compound got threefold serial dilution, and the top concentration of the compound was 30 μM in the final reaction mixture). Then 5 μL of an enzyme (2 nM) and Ser/Thr 9 peptide (4 μM) mixture was added. After the enzyme and peptide pre-incubation with the compound for 30 min at 23 °C, 5 μL of ATP (12 μM) solution was added. The plate was incubated for 60 min at 23 °C. The reaction was stopped with 5 μL of development reagent. Fluorescence signals were detected with Envision (PerkinElmer, America) after 30 min of incubation, and the emission ratio (RFU 460 nm/RFU 535 nm) was used to analyze the data. The relative IC_50_ was reported using four-parameter logistic fit of IDBS Xlfit software. The IC_50_ values were calculated using the following equation:

$$\%\mathrm{Phosphorylation}\;=\;1\;-\;\frac{\left({\mathrm{Emission}\;\mathrm{Ratio}\;\times \;F}_{100\%}\right){\;-\;C}_{100\%}}{\left(C_{0\%}{\;-\;C}_{100\%}\right)\;+\;[\mathrm{Emission}\;\mathrm{Ratio}\;\times \;\left(F_{100\%}{\;-\;F}_{0\%}\right)]}$$

where

Emission ratio = Coumarin/Fluorescein ratio of sample wells

C_100%_ = Average coumarin emission signal of the 100% phos. control

C_0%_ = Average coumarin emission signal of the 0% phos. control

F_100%_ = Average fluorescein emission signal of the 100% phos. control

F_0%_ = Average fluorescein emission signal of the 0% phos. control

$$\%\mathrm{Inhibition}\;=\;\left\{1\;-\;\frac{{\%\mathrm{Phos}\;}_{\mathrm{Sample}}}{{\%\mathrm{Phos}\;}_{0\%\mathrm{Inhibition}\;\mathrm{Ctl}}}\right\}*100$$

#### 3.2.4. Cell Glucose Uptake Assay

Into a 96-well plate, 5 × 10^3^ HepG2 cells per well were seeded. After 48 h, phenol red-free DMEM (Gibco) with 1% FBS was transferred to deprive the cells overnight, and then the tested compounds dissolved in a glucose-HEPES buffer (glucose concentration was 100 µg/mL) at a concentration of 5 µM were added to the culture system. Three hours later, the culture supernatants were collected and the glucose concentrations were detected using the Glucose (GO) Assay Kit (Sigma-Aldrich Corp.). Compared with the original glucose concentration of the buffer, the relative glucose uptake was calculated.

#### 3.2.5. Assay Molecular Modeling

The modeling study was carried out using Schrödinger Maestro 11.4. GSK-3β protein models (PDB entry 6B8J) were minimized by Schrödinger BioLuminate. The docking study was performed using Glide. The parameters chosen were the default ones, without any constraints. The molecular docking result was generated using PyMol (http://pymol.sourceforge.net/ accessed on 10 January 2021).

## 4. Conclusions

In summary, a series of 2-aminopyrimidine- and 5-aminophenyl-modified marine-originated meridianin C analogues were designed and synthesized by a structural-based optimization strategy. Among the analogues, **B29** and **B30** with 2-aminopyrimidine substitutions could significantly increase hepatocyte glucose uptake in HepG2 cells, with good inhibitory activity against GSK-3β, showing potential for the treatment of diabetes. They represent potential lead compounds for further development of GSK-3β and anti-diabetic drugs.

## Figures and Tables

**Table 1:** Chemical structures of compounds **A1**–**29** and their relative glucose uptake and cytotoxicity in a HepG2 cell model.

Compounds	R^1^	Cell Glucose Uptake (%) ^a^	Cytotoxicity (CC_50_, μM)	Compounds	R^1^	Cell Glucose Uptake (%) ^a^	Cytotoxicity (CC_50_, μM)
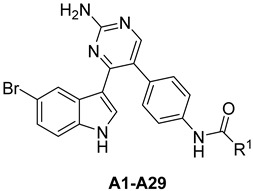							
**A1**	CH_2_CH_3_	na ^b^	16.7	**A16**	2-CF_3_-Ph	na	9.9
**A2**	CH_2_CH_2_CH_3_	na	>25	**A17**	3-CF_3_-Ph	na	14.3
**A3**	CH(CH_3_)_2_	na	10.8	**A18**	4-CF_3_-Ph	na	4.2
**A4**	C(CH_3_)_3_	na	11.9	**A19**	2-Me-Ph	na	10.3
**A5**	Bn	na	20.9	**A20**	3-Me-Ph	na	13.2
**A6**	Ph	na	12.5	**A21**	4-Me-Ph	24	6.9
**A7**	2-F-Ph	na	18.1	**A22**	2-MeO-Ph	na	>25
**A8**	3-F-Ph	na	6.8	**A23**	3-MeO-Ph	na	14.7
**A9**	4-F-Ph	na	6.0	**A24**	4-MeO-Ph	na	4.3
**A10**	2-Cl-Ph	na	18.4	**A25**	2,5-DiF-Ph	na	8.9
**A11**	3-Cl-Ph	na	18.5	**A26**	3,5-DiCl-Ph	22	14.3
**A12**	4-Cl-Ph	na	7.5	**A27**	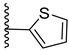	na	10.0
**A13**	2-Br-Ph	27	>25	**A28**	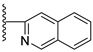	na	>25
**A14**	3-Br-Ph	na	>25	**A29**	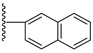	na	>25
**A15**	4-Br-Ph	na	7.1	meridianin C		na	13
				TDZD-8		16	>25

^a^ The relative glucose uptake (%) was calculated at 5 µM compared with the original glucose concentration of the culture medium before drug administration. ^b^ na = not active at 5 µM.

**Table 2:** Chemical structures of compounds **B1**–**31** and their relative glucose uptake and cytotoxicity in a HepG2 cell model.

Compounds	R^2^	Cell Glucose Uptake (%) ^a^	Cytotoxicity (CC_50_, μM)	Compounds	R^2^	Cell Glucose Uptake (%) ^a^	Cytotoxicity (CC_50_, μM)
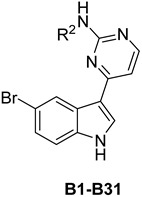							
**B1**	2-F-Ph	20	10.5	**B16**	2-MeO-Ph	17	21.4
**B2**	3-F-Ph	na ^b^	10.7	**B17**	3-MeO-Ph	na	10.6
**B3**	4-F-Ph	7.4	12.8	**B18**	4-MeO-Ph	6.1	6.0
**B4**	2-Cl-Ph	22	>25	**B19**	2-iPr-Ph	13	>25
**B5**	3-Cl-Ph	na	>25	**B20**	3-iPr-Ph	na	12.5
**B6**	4-Cl-Ph	14.4	21.1	**B21**	4-iPr-Ph	na	13.0
**B7**	2-Br-Ph	26	>25	**B22**	Ph	na	10.4
**B8**	3-Br-Ph	na	18.7	**B23**	3,5-DiF-Ph	na	16.1
**B9**	4-Br-Ph	12.6	20.3	**B24**	3,5-DiCl-Ph	na	10.5
**B10**	2-CF_3_-Ph	20.2	>25	**B25**	3,5-DiBr-Ph	na	>25
**B11**	3-CF_3_-Ph	na	17.6	**B26**	3,5-DiCF_3_-Ph	na	11.8
**B12**	4-CF_3_-Ph	na	>25	**B27**	3,5-DiMe-Ph	na	6.0
**B13**	2-Me-Ph	14.9	>25	**B28**	3,5-DiMeO-Ph	na	10.0
**B14**	3-Me-Ph	na	8.6	**B29**	2,6-DiF-Ph	29	22.4
**B15**	4-Me-Ph	na	15.5	**B30**	2,6-DiMe-Ph	38	18.1
meridianin C		na	13.0	**B31**	2,6-DiMeO-Ph	16	19.2
TDZD-8		16	>25				

^a^ The relative glucose uptake (%) was calculated at 5 µM compared with the original glucose concentration of the culture medium before drug administration. ^b^ na = not active at 5 µM.

**Table 3:** Inhibitory activity (IC_50_) against human GSK-3β.

Compounds	IC_50_ (μM) GSK-3β
A13	>30
A26	>30
B29	5.85
B30	24.4
meridianin C	2 [18]

## Supplementary Materials

The following are available online at https://www.mdpi.com/1660-3397/19/3/149/s1: NMR, HRMS, HPLC purity.

## Abbreviations

GSK-3β, glycogen synthase kinase 3β; GCS, glycogen synthase; HepG2, human hepatocellular carcinoma; FBS, fetal bovine serum; PBS, phosphate buffered saline; HPLC, high-performance liquid chromatography; NMR, nuclear magnetic resonance; DMF, dimethylformamide; DMAP, 4-(dimethylamino)-pyridine; DIPEA, *N*,*N*-diisopropylethylamine; NIS, *N*-iodosuccinimide; TsCl, tosyl chloride; ADMET, (absorption, distribution, metabolism, excretion, and toxicity); TDZD-8, 4-benzyl-2-methyl-1,2,4-thiadiazolidine-3,5-dione.
