# Supplementary material for: Structural-Based Optimizations of the Marine-Originated Meridianin C as Glucose Uptake Agents by Inhibiting GSK-3β
Source: Mar Drugs. 2021 Mar 12;19(3):149. doi: 10.3390/md19030149 (PMC7998309; doi:10.3390/md19030149)
Supplement: Supplementary file 1 [file marinedrugs-19-00149-s001.pdf]

Supporting Information for

Structural-Based Optimizations of the Marine-Originated Meridianin C as Glucose Uptake Agents by Inhibiting GSK-3β

Shuwen Han [1,2], Chunlin Zhuang [2,3], Wei Zhou [4,*], Fener Chen[1,2,3,*]

[1] Institutes of Biomedical Sciences, Fudan University, Shanghai 200433, China; a8986330@163.com (S. H.)
[2] Shanghai Engineering Center of Industrial Asymmetric Catalysis for Chiral Drugs, Shanghai 200433, China; zclnathan@163.com (C. Z.); rfchen@fudan.edu.cn (F. C.)
[3] Engineering Center of Catalysis and Synthesis for Chiral Molecules, Department of Chemistry, Fudan University, Shanghai 200433, China
[4] Department of Chemistry, Shanghai Key Laboratory of Molecular Catalysis and Innovative Materials, Fudan University, Shanghai 200438, China; zhouw@fudan.edu.cn (W. Z.)
* Correspondence: zhouw@fudan.edu.cn; rfchen@fudan.edu.cn

Table of Contents

NMR of intermediate compounds 2-10……………..........................S2-S5

NMR, HRMS and HPLC purity spectra of final compounds………........S6

Dose-response curves of the GSK3β assays…………………..……........S186

^{1}H NMR spectra of compound 2

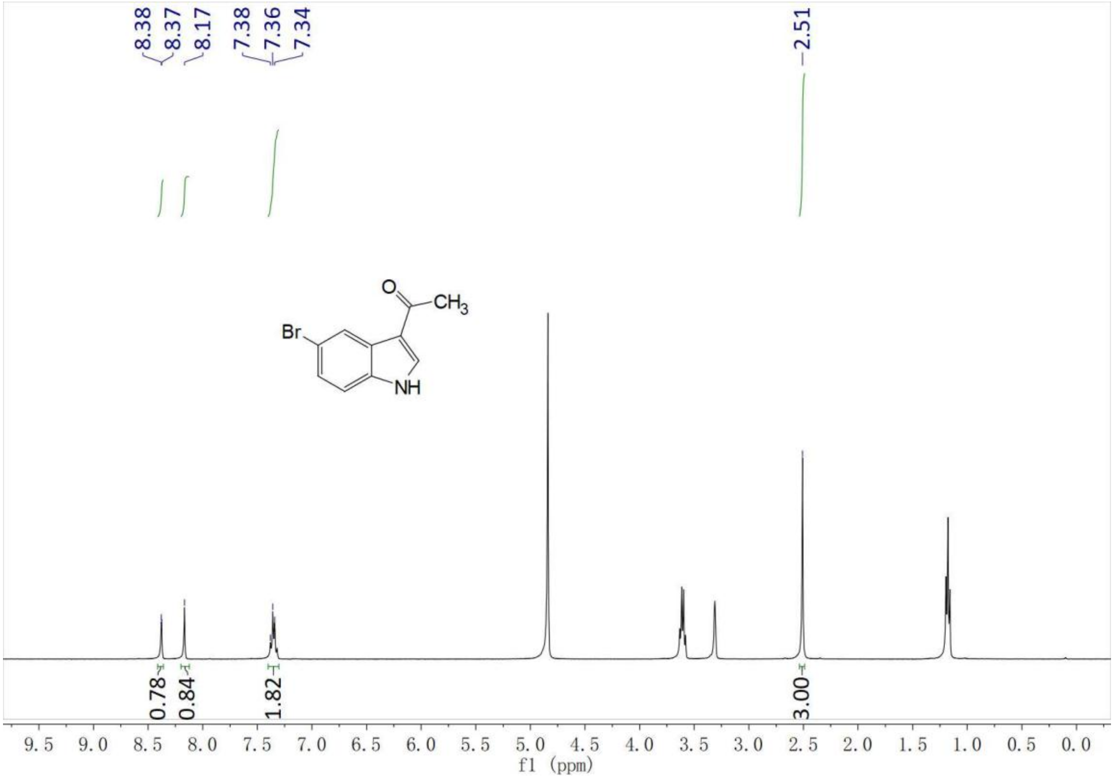

^{1}H NMR spectra of compound 3

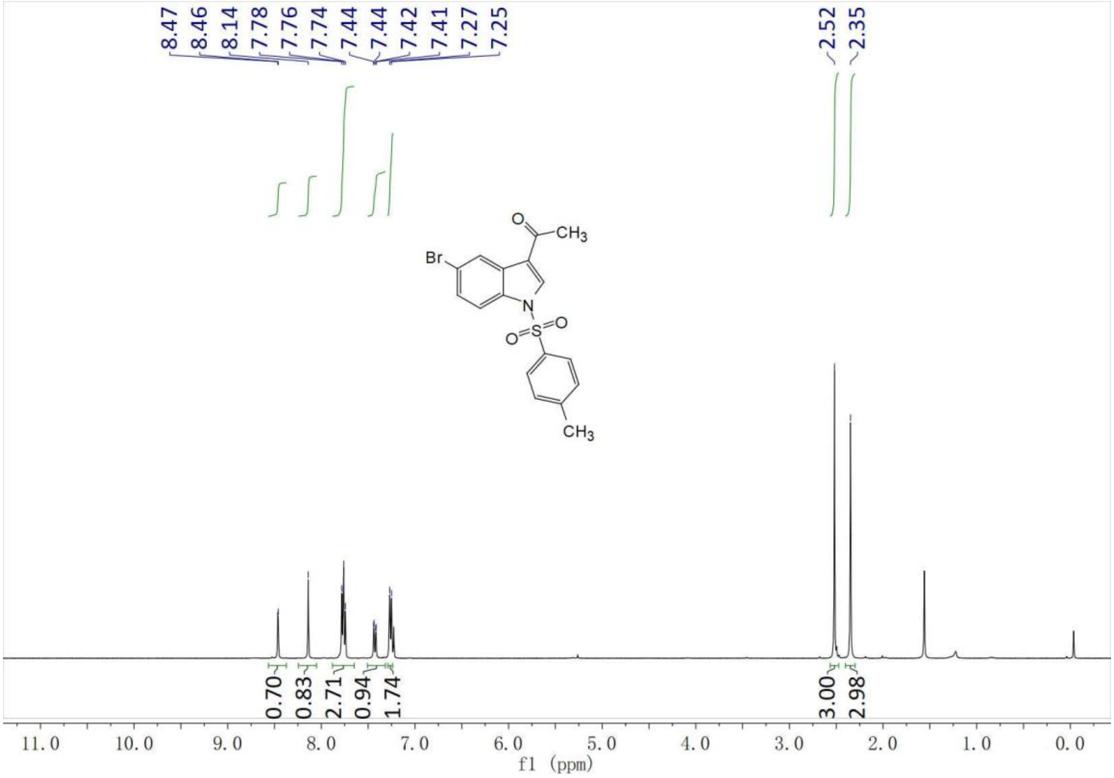

^{1}**H NMR spectra of compound 4**

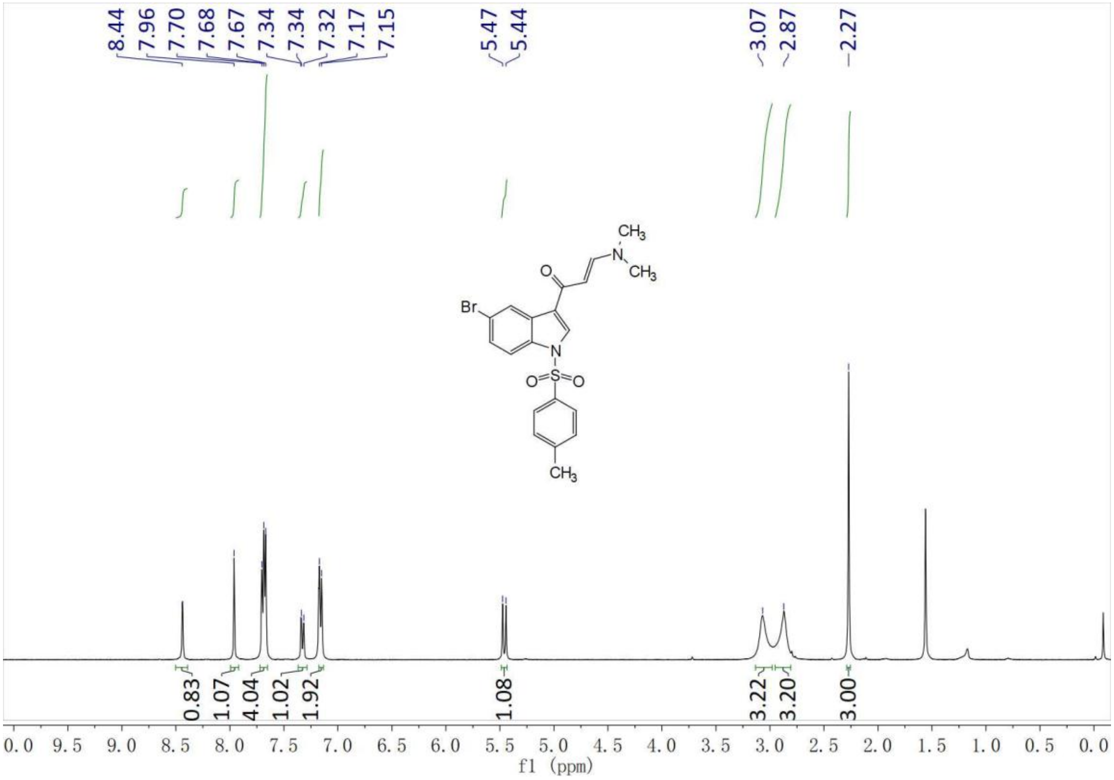

^{1}**H NMR spectra of compound 5**

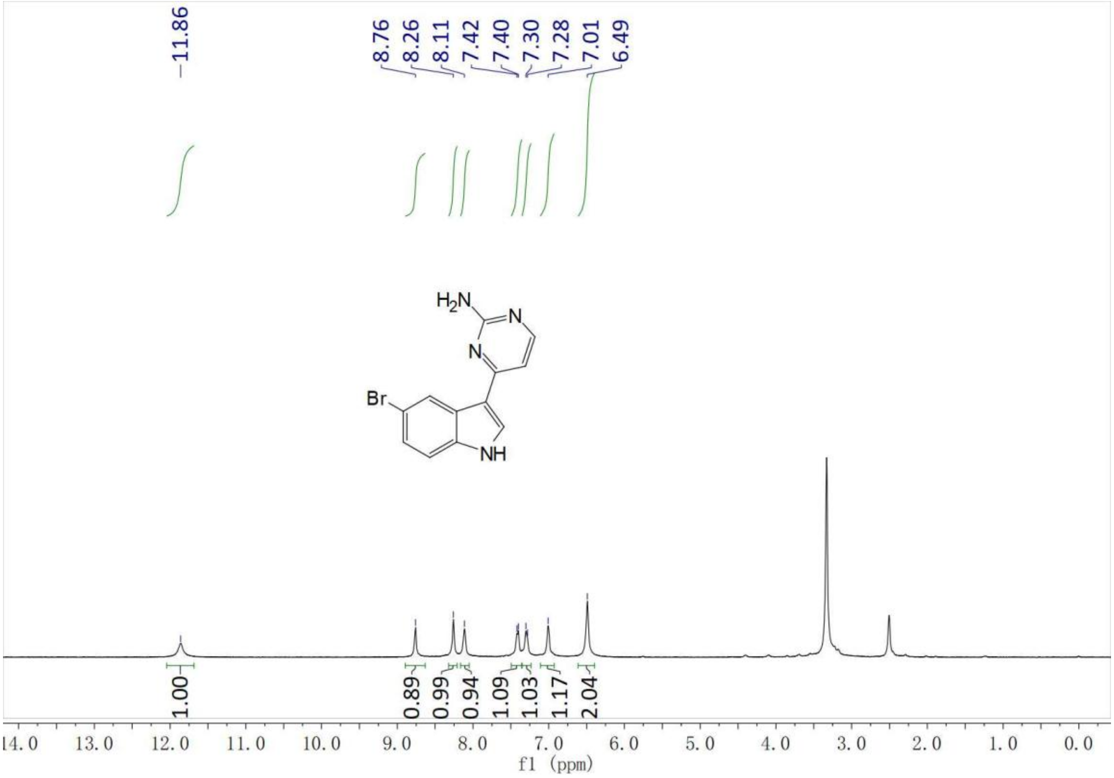

^{1}H NMR spectra of compound 6

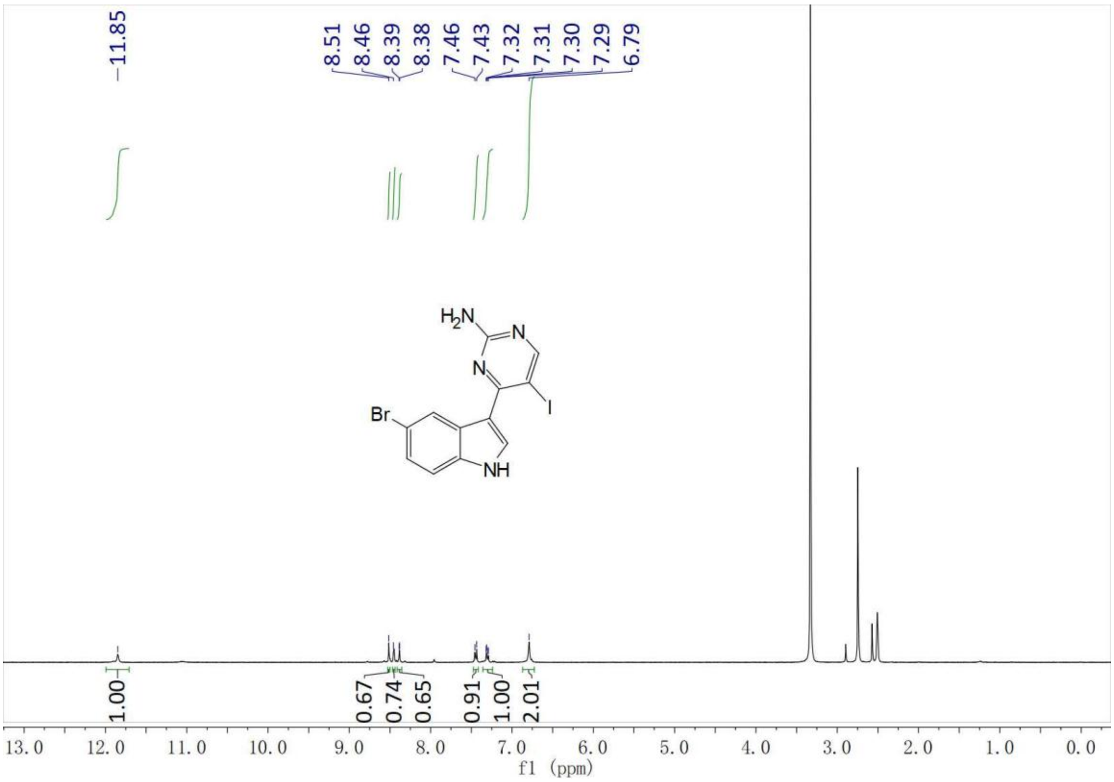

^{1}H NMR spectra of compound 8

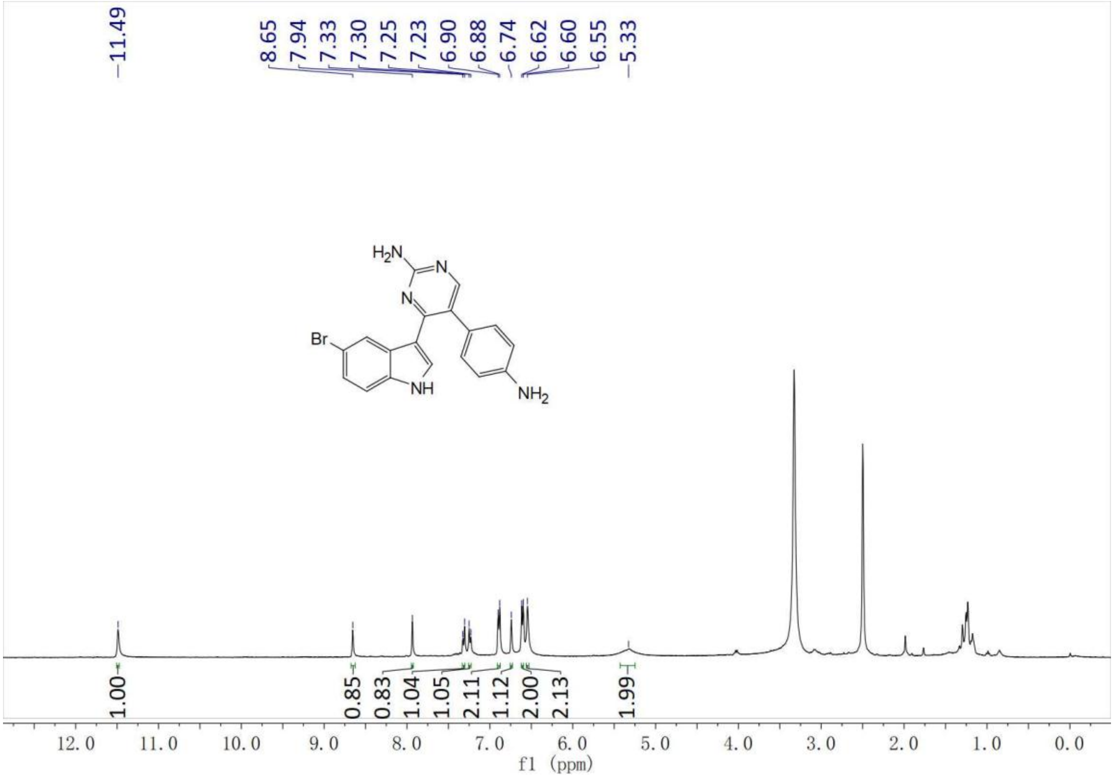

^{1}H NMR spectra of compound 10

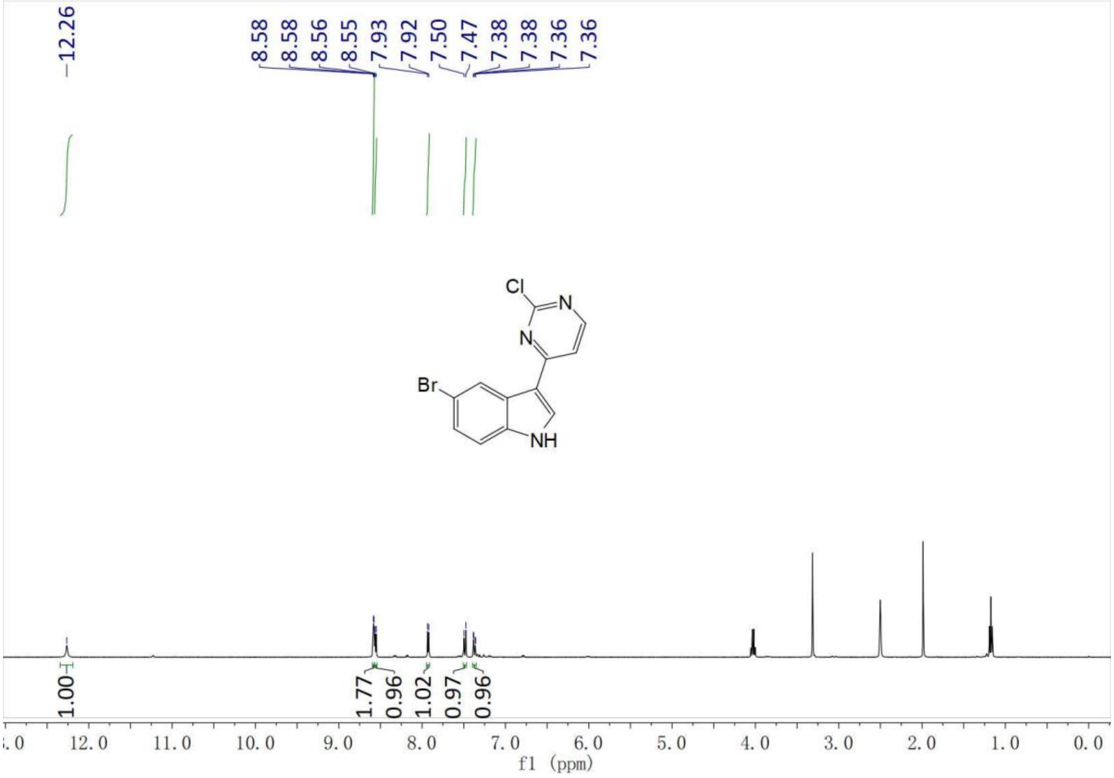

^{1}H NMR, ^{13}C NMR, HRMS, and HPLC of compound A1

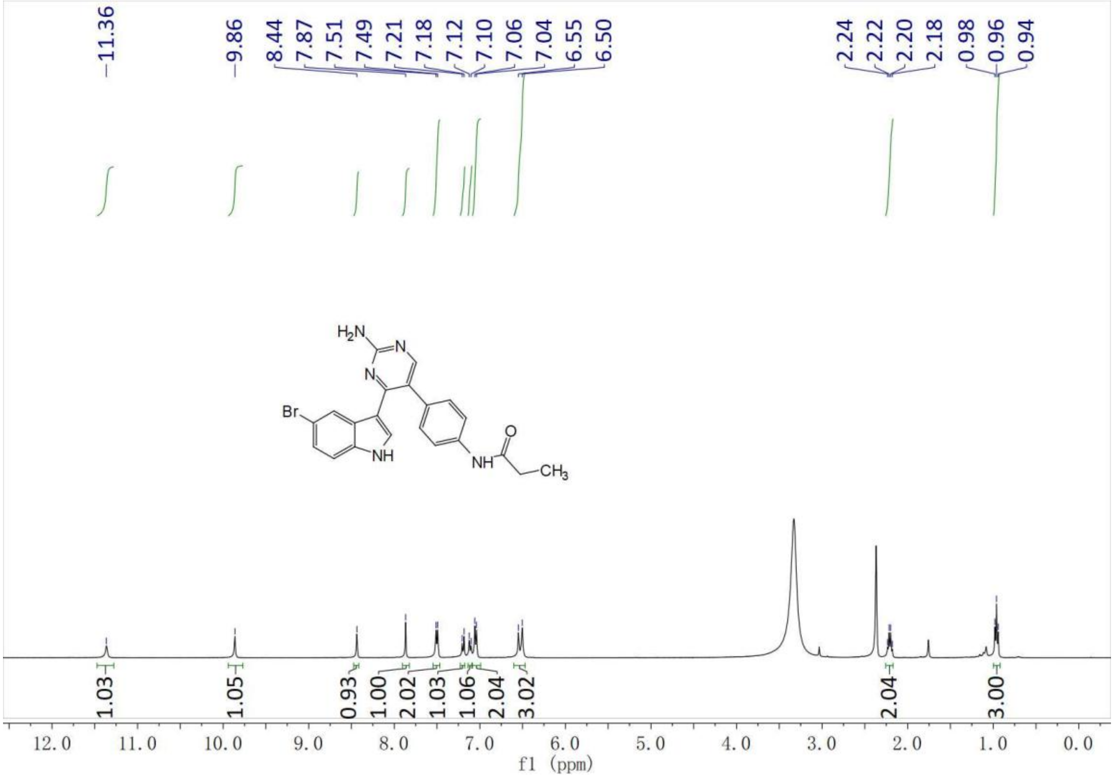

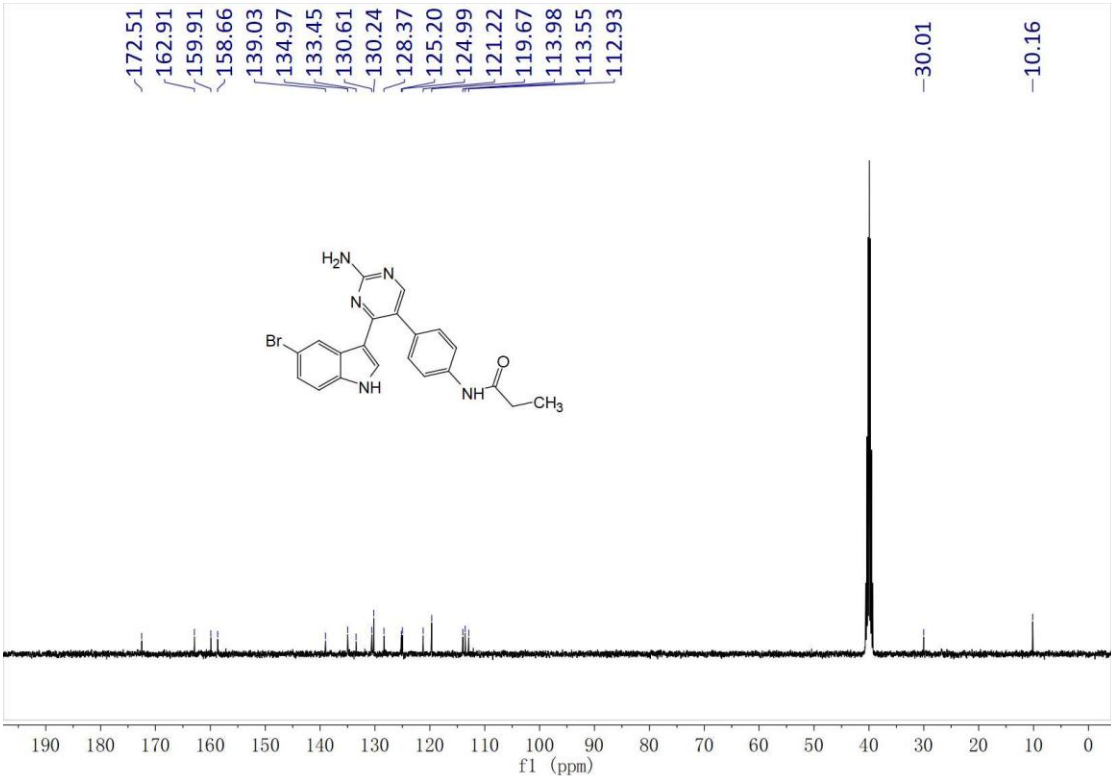

172.51
162.91
159.91
158.66
139.03
134.97
133.45
130.61
130.24
128.37
125.20
124.99
121.22
119.67
113.98
113.55
112.93
30.01
10.16
H2N
N
N
Br
NH
NH
O
CH3
190 180 170 160 150 140 130 120 110 100 90 80 70 60 50 40 30 20 10 0
f1 (ppm)

Display Report

Analysis Info

Analysis Name	D:\Data\data\2020\TC23_RC3_01_4200.d	Acquisition Date	10/10/2020 15:54:11 PM
Method	MS-2MIN-POS.m	Operator	BDAL@DE
Sample Name	TC23	Instrument	compact 8255754.20127
Comment			

Acquisition Parameter

Source Type	ESI	Ion Polarity	Positive	Set Nebulizer	2.0 Bar
Focus	Active	Set Capillary	4500 V	Set Dry Heater	200 °C
Scan Begin	50 m/z	Set End Plate Offset	-500 V	Set Dry Gas	8.0 l/min
Scan End	3000 m/z	Set Charging Voltage	2000 V	Set Divert Valve	Waste
		Set Corona	0 nA	Set APCI Heater	0 °C

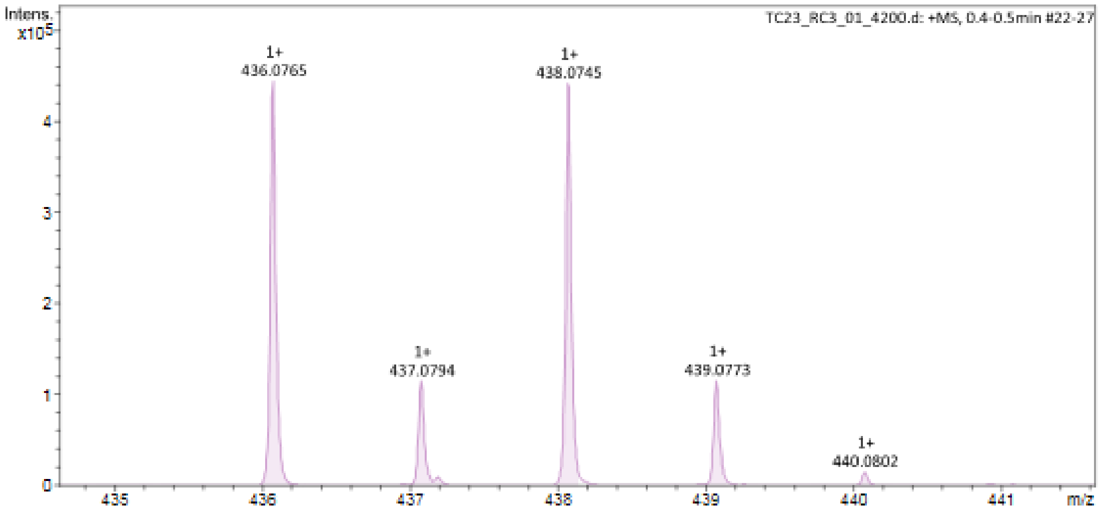

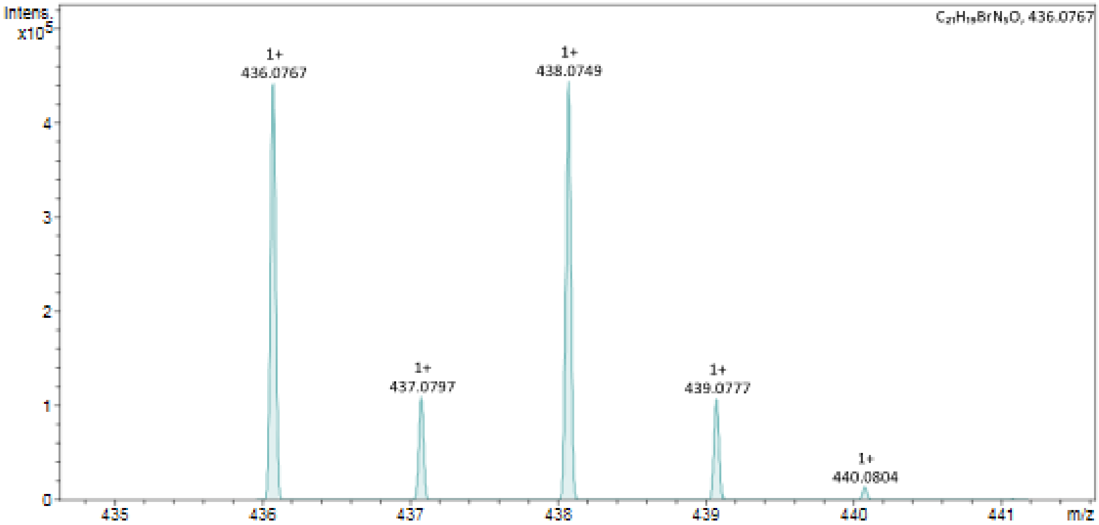

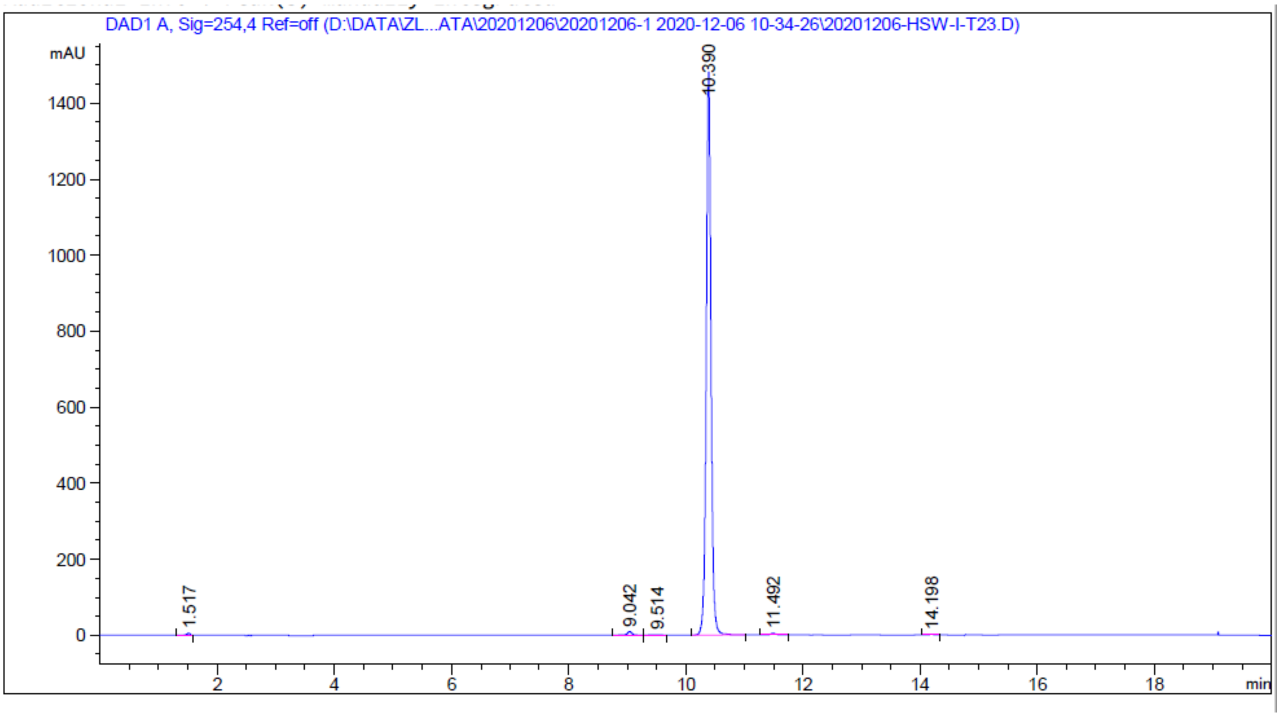

Signal 1: DAD1 A, Sig=254,4 Ref=off

Peak #	RetTime [min]	Type	Width [min]	Area [mAU*s]	Height [mAU]	Area %
1	1.517	BB	0.0573	21.93911	5.90471	0.2600
2	9.042	BB	0.0920	61.66922	9.85872	0.7307
3	9.514	BB	0.0987	10.80172	1.32342	0.1280

1260R 12/6/2020 2:42:36 PM BY

Data File D:\DATA\ZLM\DATA\20201206\20201206-1 2020-12-06 10-34-26\20201206-HSW-I-T23.D
Sample Name: 20201206-HSW-I-T23

Peak #	RetTime [min]	Type	Width [min]	Area [mAU*s]	Height [mAU]	Area %
4	10.390	BV R	0.0843	8294.30469	1482.21606	98.2791
5	11.492	BB	0.1078	36.35308	4.49604	0.4307
6	14.198	BB	0.0774	14.47206	2.48841	0.1715

Totals : 8439.53989 1506.28737

^{1}H NMR, ^{13}C NMR, HRMS, and HPLC of compound A2

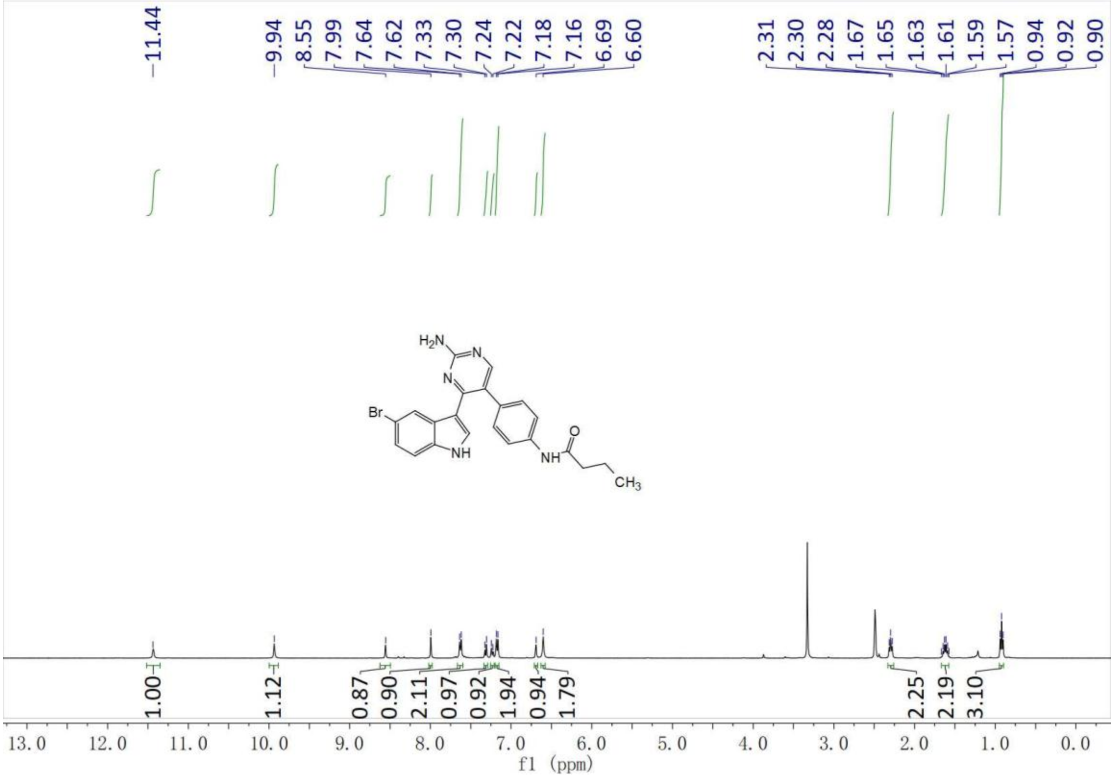

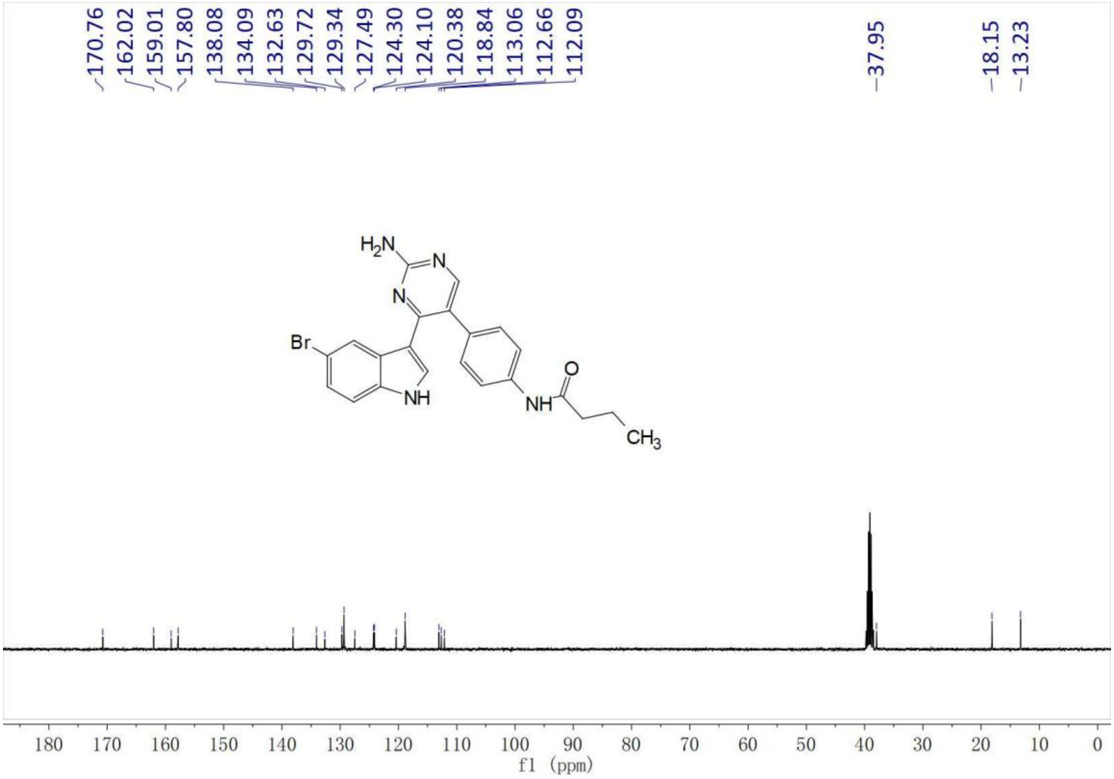

Display Report

Analysis Info

Analysis Name	D:\Data\data\2020\TC24_RC4_01_4201.d	Acquisition Date	10/10/2020 15:56:57 PM
Method	MS-2MIN-POS.m	Operator	BDAL@DE
Sample Name	TC24	Instrument	compact 8255754.20127
Comment			

Acquisition Parameter

Source Type	ESI	Ion Polarity	Positive	Set Nebulizer	2.0 Bar
Focus	Active	Set Capillary	4500 V	Set Dry Heater	200 °C
Scan Begin	50 m/z	Set End Plate Offset	-500 V	Set Dry Gas	8.0 l/min
Scan End	3000 m/z	Set Charging Voltage	2000 V	Set Divert Valve	Waste
		Set Corona	0 nA	Set APCI Heater	0 °C

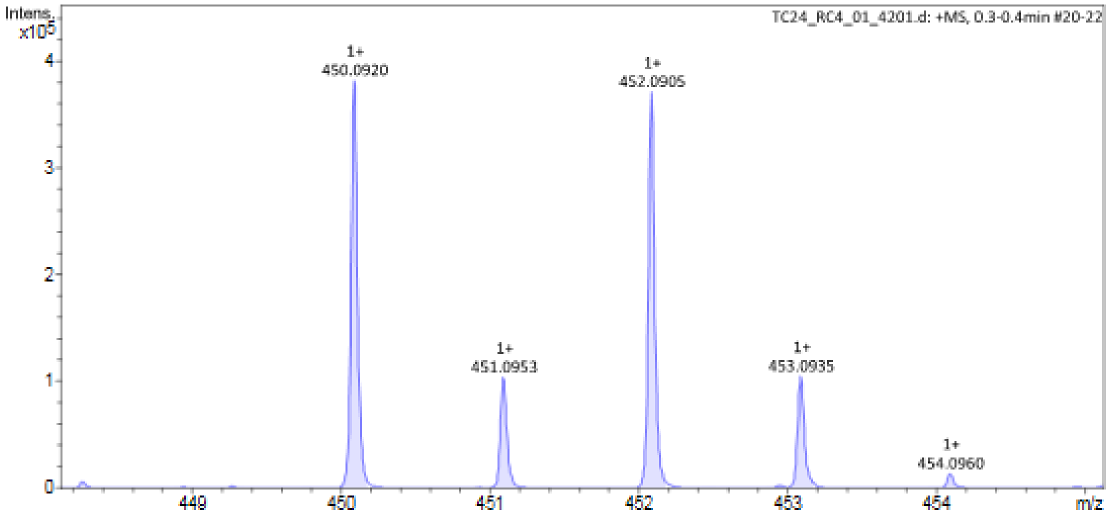

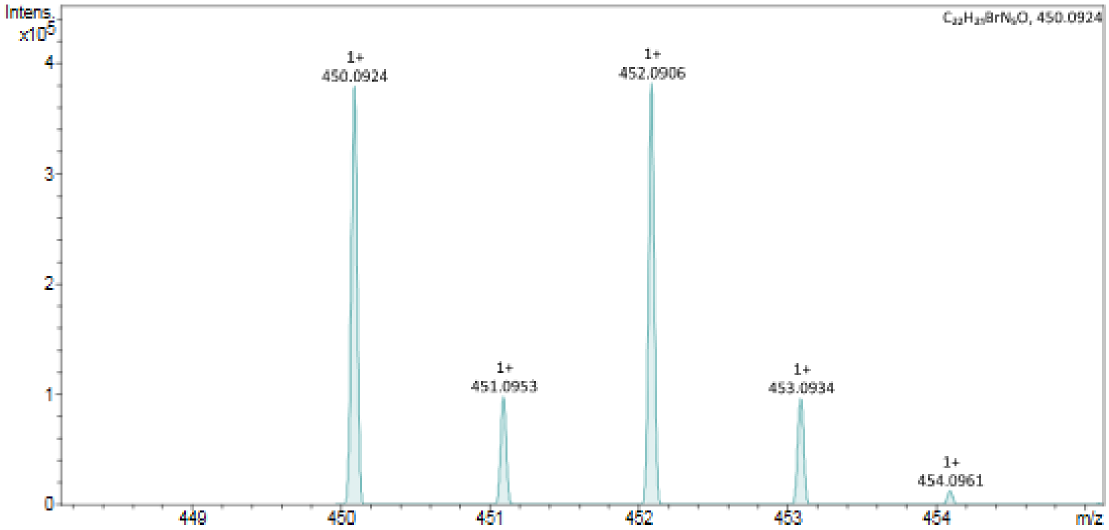

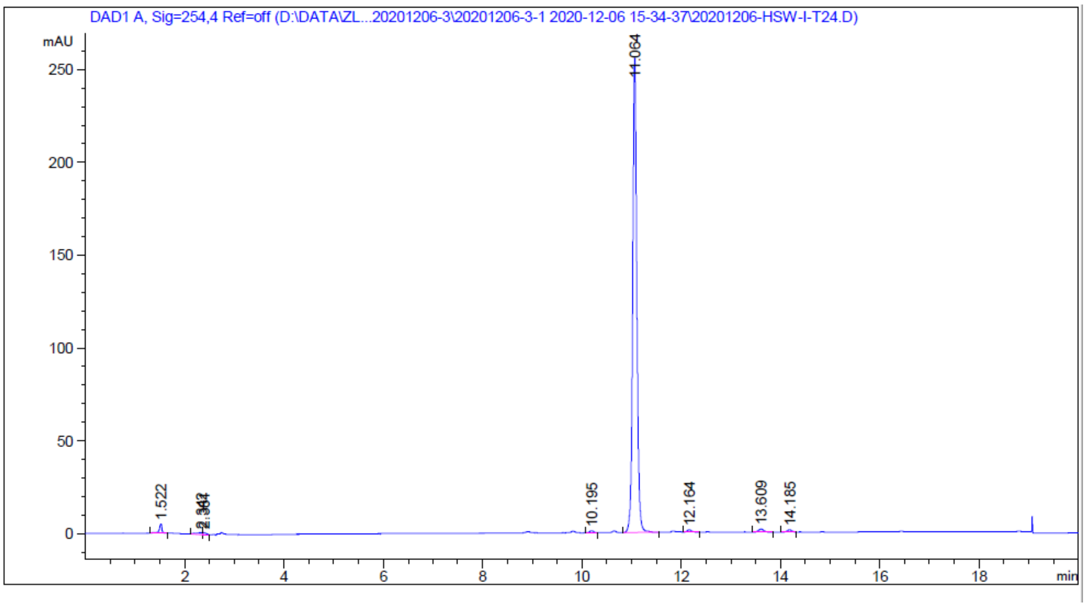

Signal 1: DAD1 A, Sig=254,4 Ref=off

Peak #	RetTime [min]	Type	Width [min]	Area [mAU*s]	Height [mAU]	Area %
1	1.522	BB	0.0609	20.16367	4.96293	1.3050
2	2.342	BV	0.0958	8.74167	1.09849	0.5658
3	2.381	VB	0.0578	5.48754	1.16891	0.3552

1260R 12/6/2020 7:29:21 PM BY

Data File D:\DATA\ZL...TA\20201206-3\20201206-3-1 2020-12-06 15-34-37\20201206
Sample Name: 20201206-HSW-I-T24

Peak #	RetTime [min]	Type	Width [min]	Area [mAU*s]	Height [mAU]	Area %
4	10.195	BB	0.0672	5.87567	1.12096	0.3803
5	11.064	BB	0.0878	1476.96924	256.21567	95.5931
6	12.164	BB	0.0820	7.33410	1.18834	0.4747
7	13.609	BB	0.1001	12.87626	1.67745	0.8334
8	14.185	BB	0.0779	7.60989	1.27077	0.4925

^{1}H NMR, ^{13}C NMR, HRMS, and HPLC of compound A3

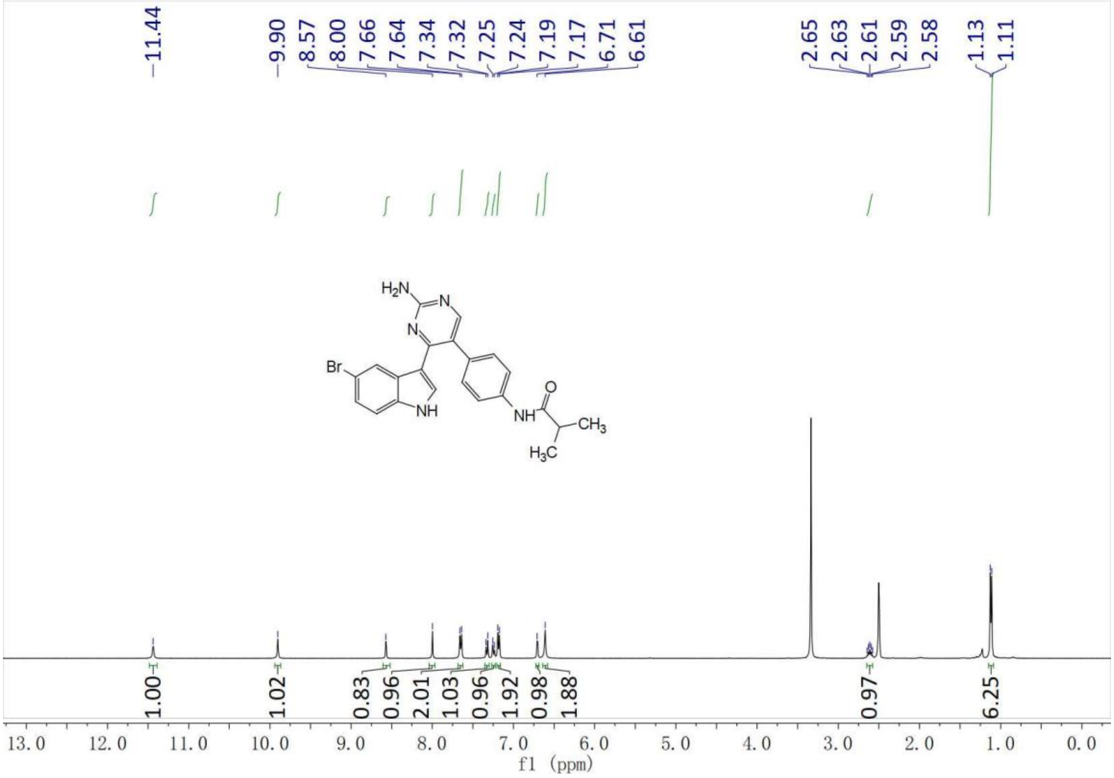

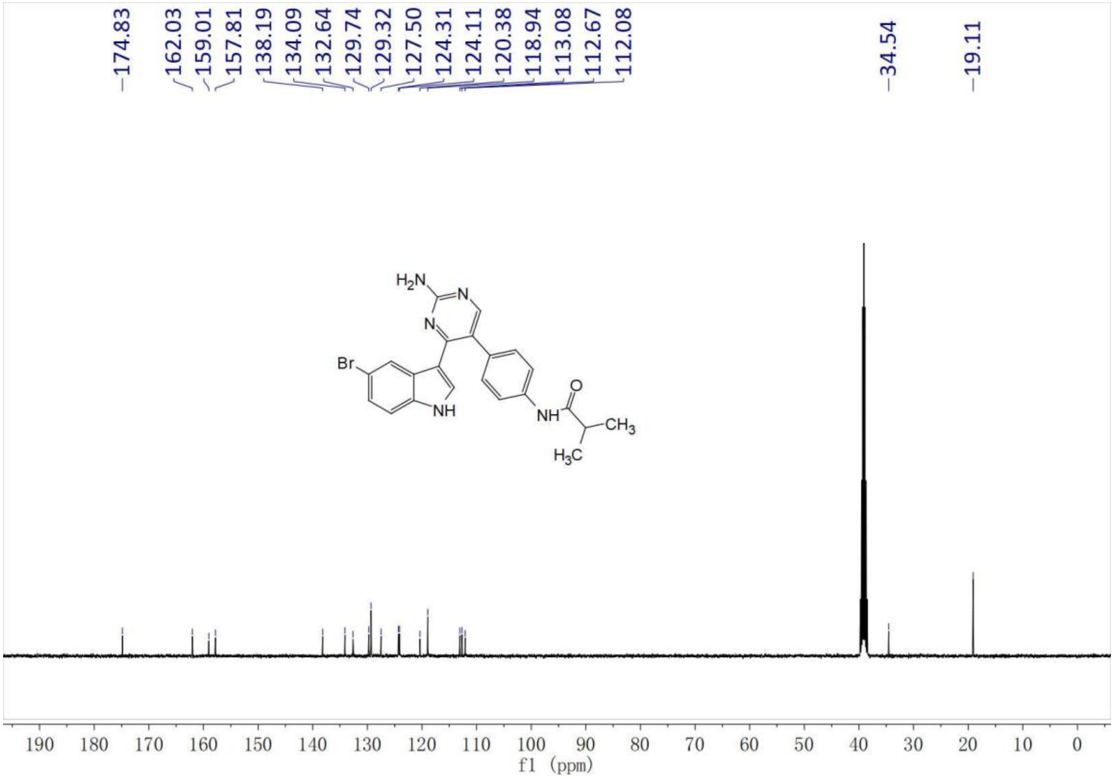

Display Report

Analysis Info

Analysis Name	D:\Data\data\2020\TC25_RC5_01_4202.d	Acquisition Date	10/10/2020 15:59:43 PM
Method	MS-2MIN-POS.m	Operator	BDAL@DE
Sample Name	TC25	Instrument	compact 8255754.20127
Comment			

Acquisition Parameter

Source Type	ESI	Ion Polarity	Positive	Set Nebulizer	2.0 Bar
Focus	Active	Set Capillary	4500 V	Set Dry Heater	200 °C
Scan Begin	50 m/z	Set End Plate Offset	-500 V	Set Dry Gas	8.0 l/min
Scan End	3000 m/z	Set Charging Voltage	2000 V	Set Divert Valve	Waste
		Set Corona	0 nA	Set APCI Heater	0 °C

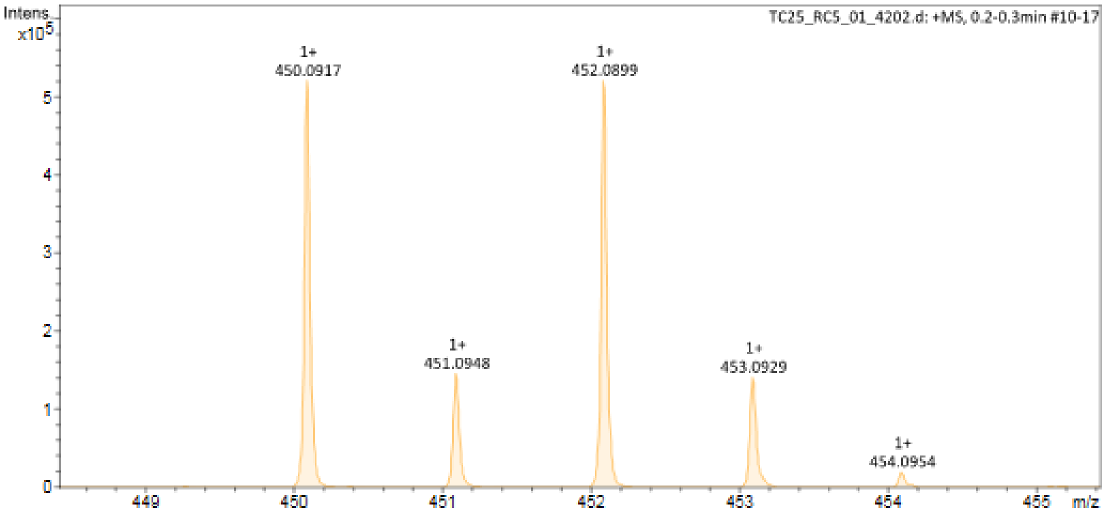

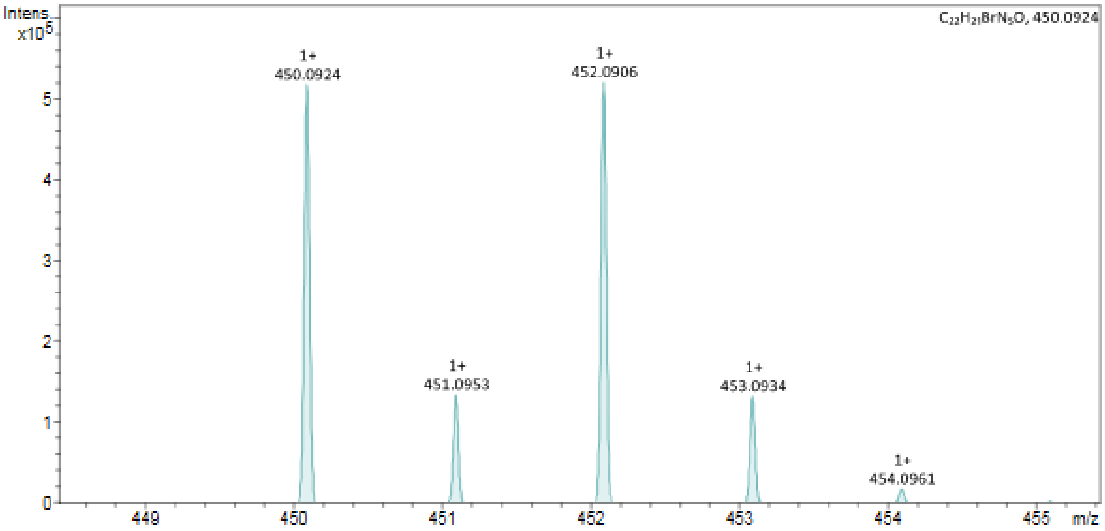

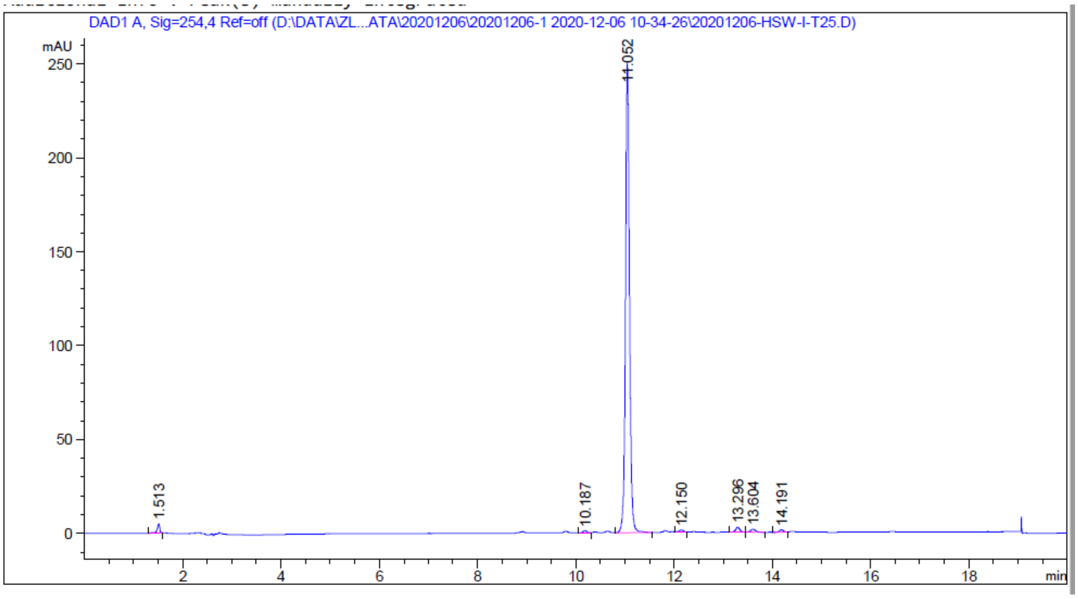

Signal 1: DAD1 A, Sig=254,4 Ref=off

Peak #	RetTime [min]	Type	Width [min]	Area [mAU*s]	Height [mAU]	Area %
1	1.513	BB	0.0578	17.89703	4.71478	1.2101
2	10.187	BB	0.0681	5.91592	1.13920	0.4000
3	11.052	BB	0.0849	1414.57910	250.56532	95.6425

1260R 12/6/2020 7:32:14 PM BY

Data File D:\DATA\ZLM\DATA\20201206\20201206-1 2020-12-06 10-34-26\20201206-HSW-I-T25.D
Sample Name: 20201206-HSW-I-T25

Peak #	RetTime [min]	Type	Width [min]	Area [mAU*s]	Height [mAU]	Area %
4	12.150	BB	0.0708	6.08525	1.03950	0.4114
5	13.296	BB	0.0829	15.67165	2.61401	1.0596
6	13.604	BB	0.1072	11.86040	1.60473	0.8019
7	14.191	BB	0.0804	7.01777	1.23296	0.4745

Totals : 1479.02712 262.91049

^{1}H NMR, ^{13}C NMR, HRMS, and HPLC of compound A4

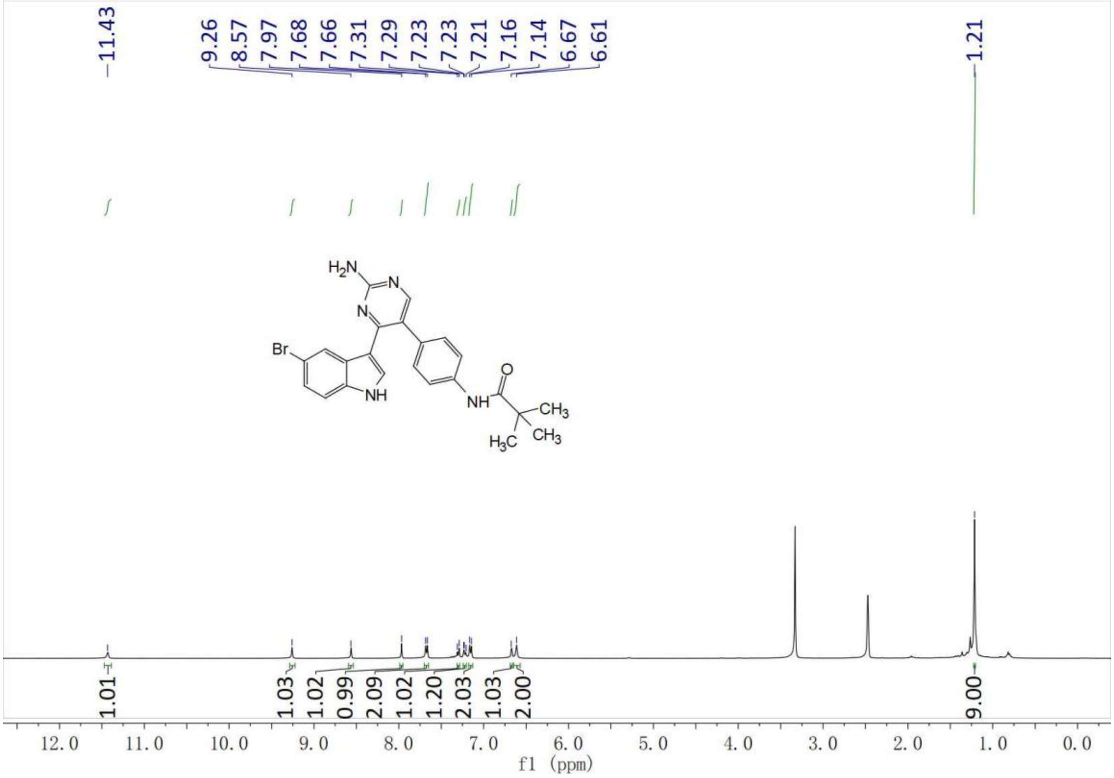

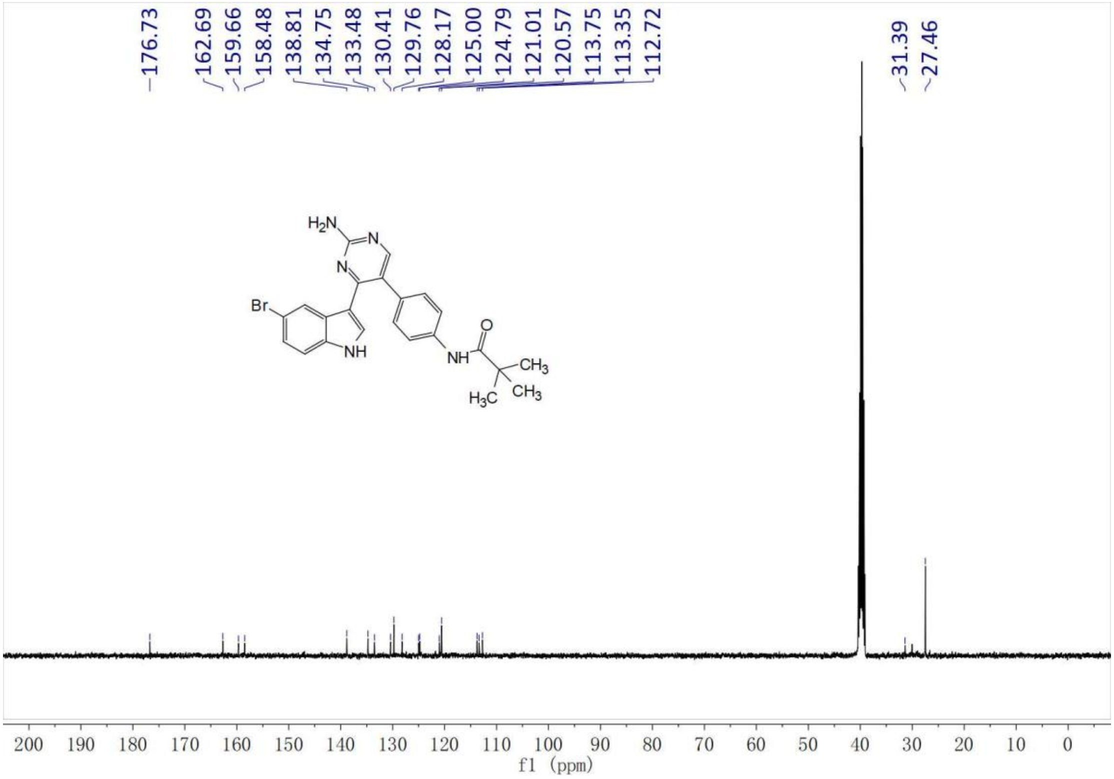

Display Report

Analysis Info

Analysis Name	D:\Data\data\2020\TC26_RC6_01_4203.d	Acquisition Date	10/10/2020 16:02:29 PM
Method	MS-2MIN-POS.m	Operator	BDAL@DE
Sample Name	TC26	Instrument	compact 8255754.20127
Comment			

Acquisition Parameter

Source Type	ESI	Ion Polarity	Positive	Set Nebulizer	2.0 Bar
Focus	Active	Set Capillary	4500 V	Set Dry Heater	200 °C
Scan Begin	50 m/z	Set End Plate Offset	-500 V	Set Dry Gas	8.0 l/min
Scan End	3000 m/z	Set Charging Voltage	2000 V	Set Divert Valve	Waste
		Set Corona	0 nA	Set APCI Heater	0 °C

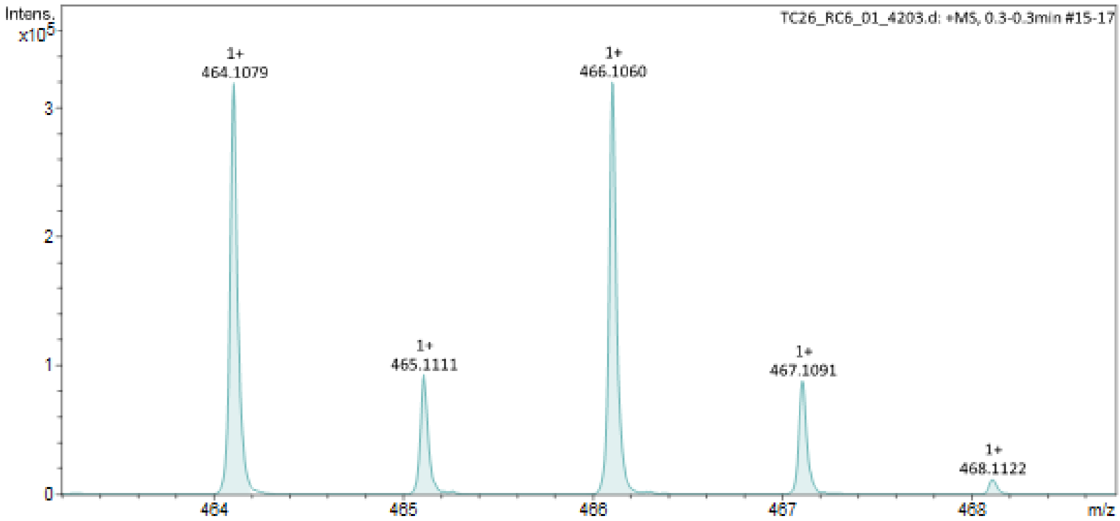

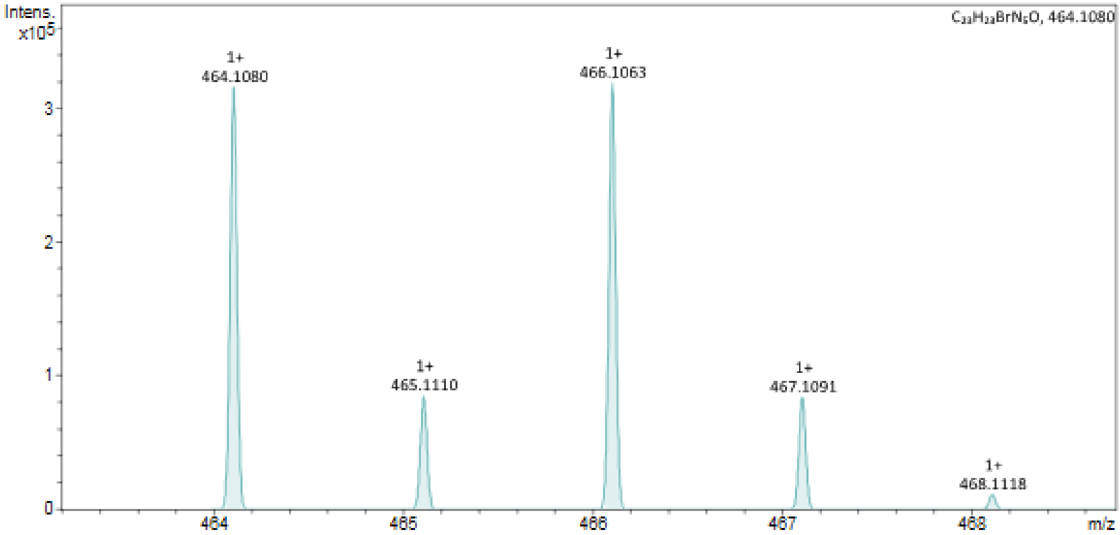

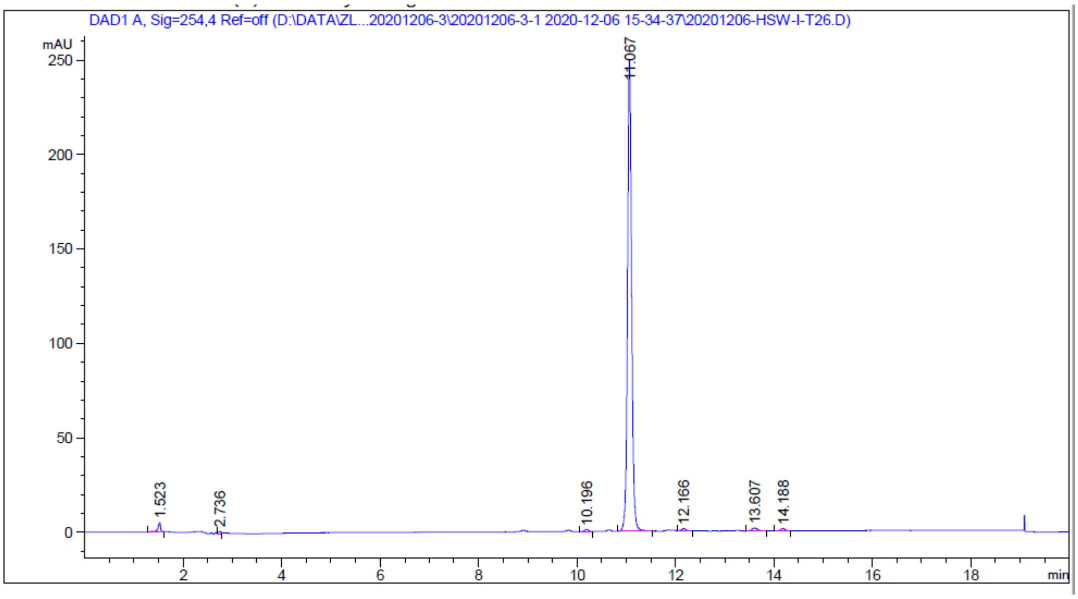

Signal 1: DAD1 A, Sig=254,4 Ref=off

Peak #	RetTime [min]	Type	Width [min]	Area [mAU*s]	Height [mAU]	Area %
1	1.523	BB	0.0611	18.97841	4.75006	1.2635
2	2.736	VV	0.0493	5.99407	1.52676	0.3991
3	10.196	BB	0.0646	5.84239	1.10739	0.3890

1260R 12/6/2020 7:31:05 PM BY

Data File D:\DATA\ZL...TA\20201206-3\20201206-3-1 2020-12-06 15-34-37\2020120[illegible]
Sample Name: 20201206-HSW-I-T26

Peak #	RetTime [min]	Type	Width [min]	Area [mAU*s]	Height [mAU]	Area %
4	11.067	BB	0.0880	1443.76782	249.66632	96.1198
5	12.166	BB	0.0772	7.68430	1.24226	0.5116
6	13.607	BB	0.0909	12.36686	1.63242	0.8233
7	14.188	BB	0.0754	7.41651	1.23827	0.4938
Totals :				1502.05037	261.16349	

^{1}H NMR, ^{13}C NMR, HRMS, and HPLC of compound A5

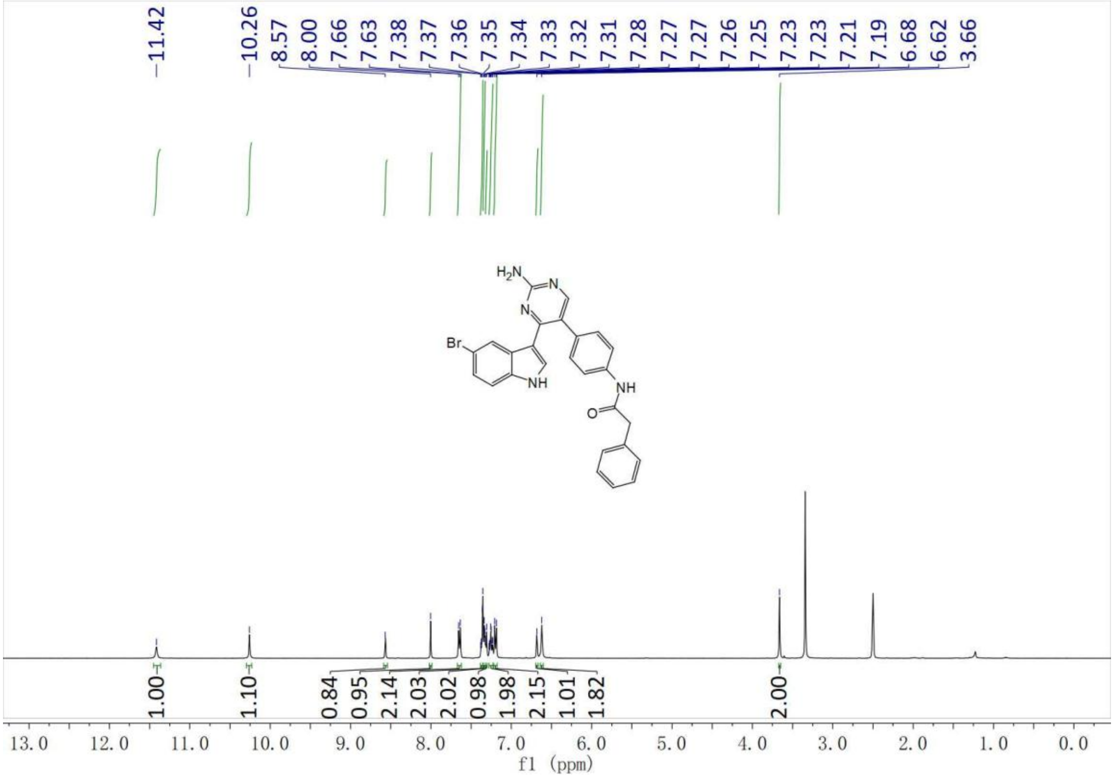

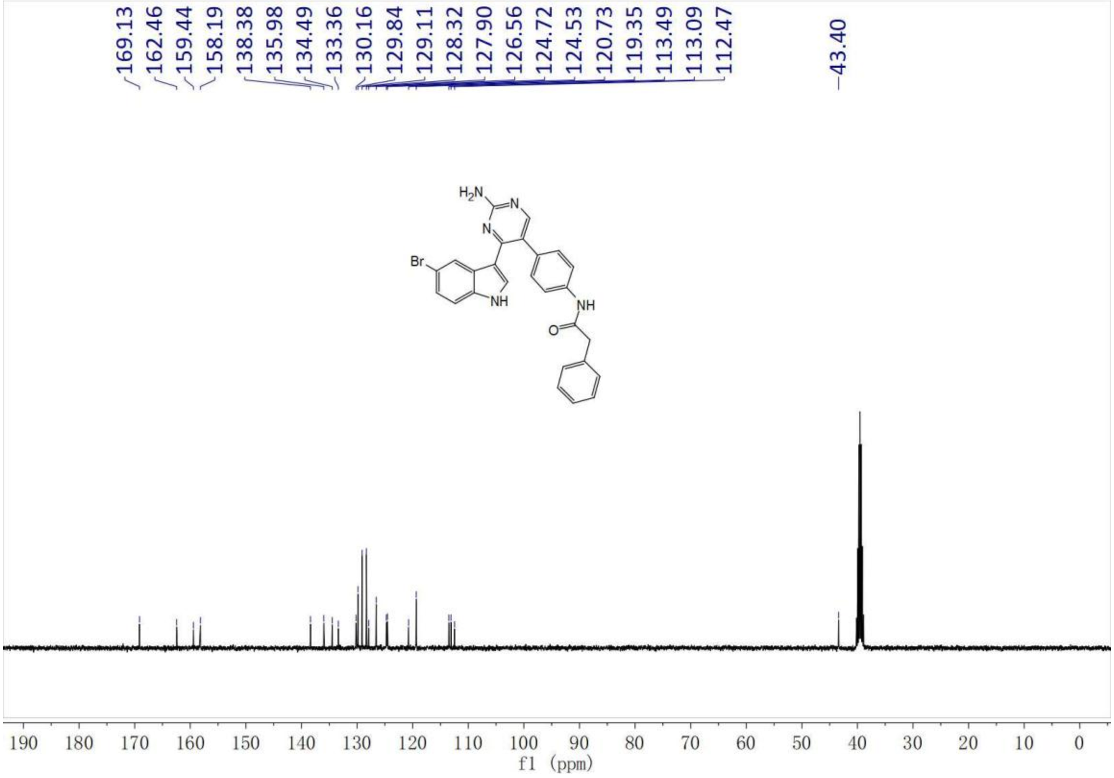

Display Report

Analysis Info

Analysis Name	D:\Data\data\2020\TC21_RC2_01_4199.d	Acquisition Date	10/10/2020 15:51:25 PM	
Method	MS-2MIN-POS.m	Operator	BDAL@DE	
Sample Name	TC21	Instrument	compact	8255754.20127
Comment				

Acquisition Parameter

Source Type	ESI	Ion Polarity	Positive	Set Nebulizer	2.0 Bar
Focus	Active	Set Capillary	4500 V	Set Dry Heater	200 °C
Scan Begin	50 m/z	Set End Plate Offset	-500 V	Set Dry Gas	8.0 l/min
Scan End	3000 m/z	Set Charging Voltage	2000 V	Set Divert Valve	Waste
		Set Corona	0 nA	Set APCI Heater	0 °C

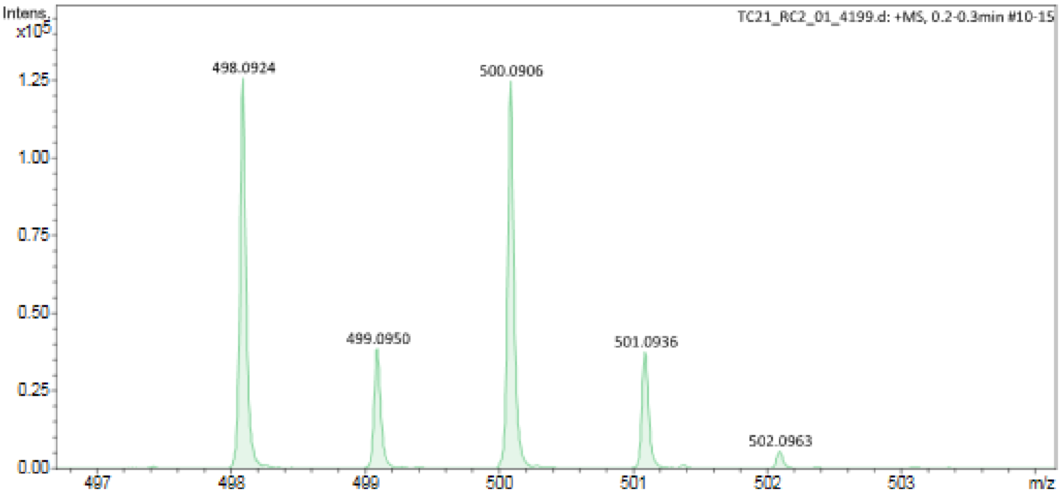

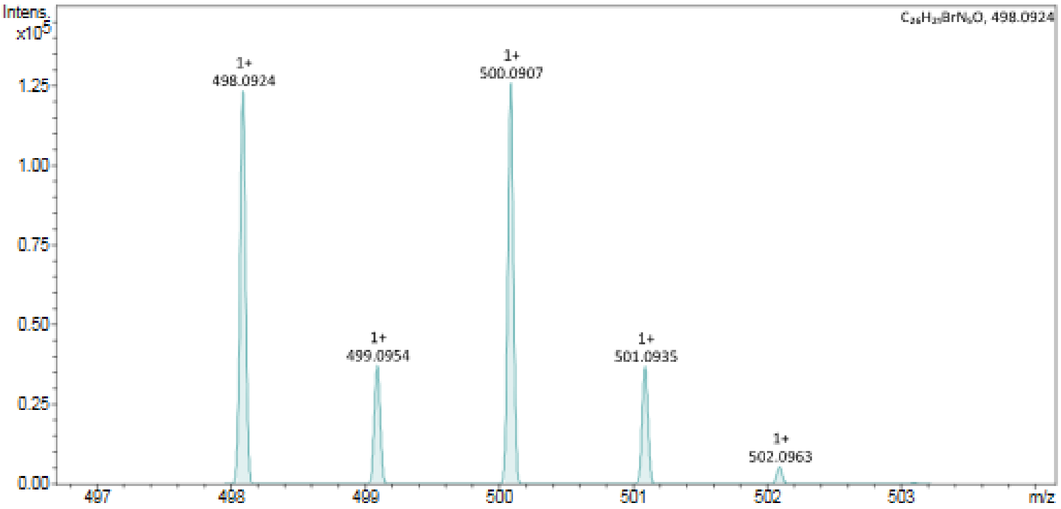

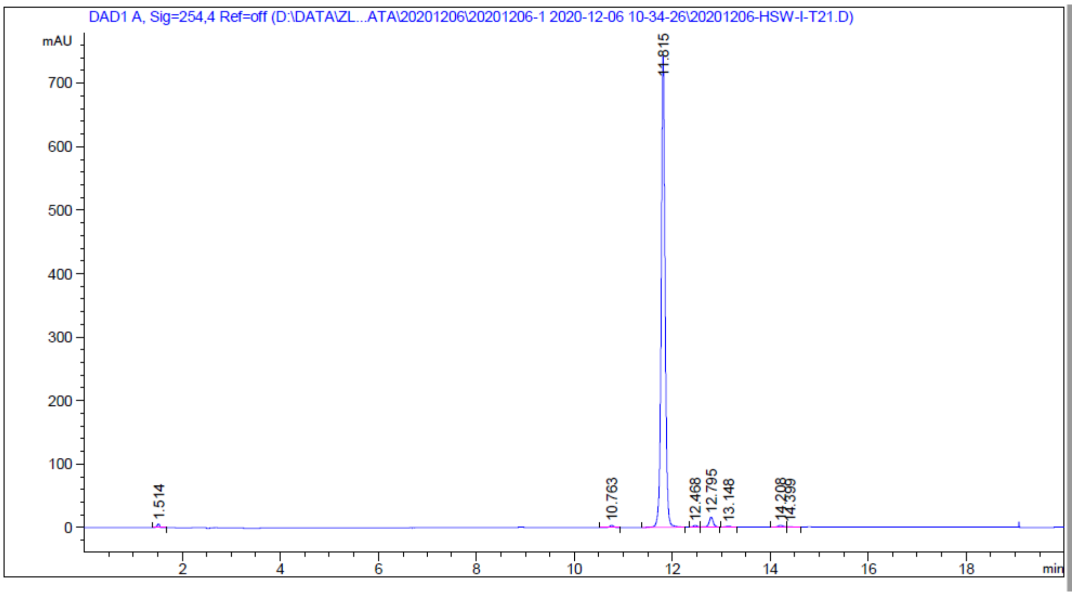

Signal 1: DAD1 A, Sig=254,4 Ref=off

Peak #	RetTime [min]	Type	Width [min]	Area [mAU*s]	Height [mAU]	Area %
1	1.514	BB	0.0561	20.95823	5.81246	0.4928
2	10.763	BB	0.0781	18.56174	3.43252	0.4364
3	11.815	BB	0.0825	4068.03491	741.20227	95.6449

1260R 12/6/2020 2:41:40 PM BY

Data File D:\DATA\ZLM\DATA\20201206\20201206-1 2020-12-06 10-34-26\20201206-HSW-I-T21.D
Sample Name: 20201206-HSW-I-T21

Peak #	RetTime [min]	Type	Width [min]	Area [mAU*s]	Height [mAU]	Area %
4	12.468	BB	0.0781	12.95952	2.35741	0.3047
5	12.795	BB	0.0878	91.43913	15.62631	2.1499
6	13.148	BB	0.0868	10.88776	1.65318	0.2560
7	14.208	BV	0.0954	22.72798	3.04982	0.5344
8	14.399	VB	0.0896	7.70011	1.01989	0.1810
Totals :				4253.26938	774.15386	

^{1}H NMR, ^{13}C NMR, HRMS, and HPLC of compound A6

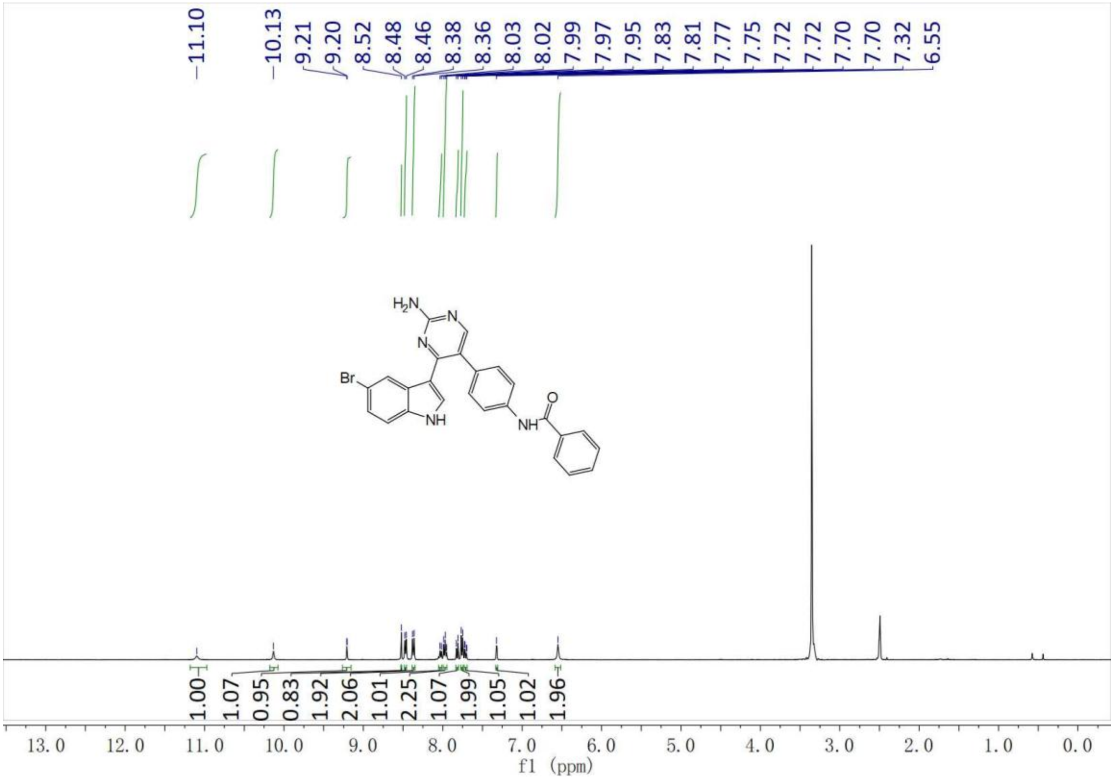

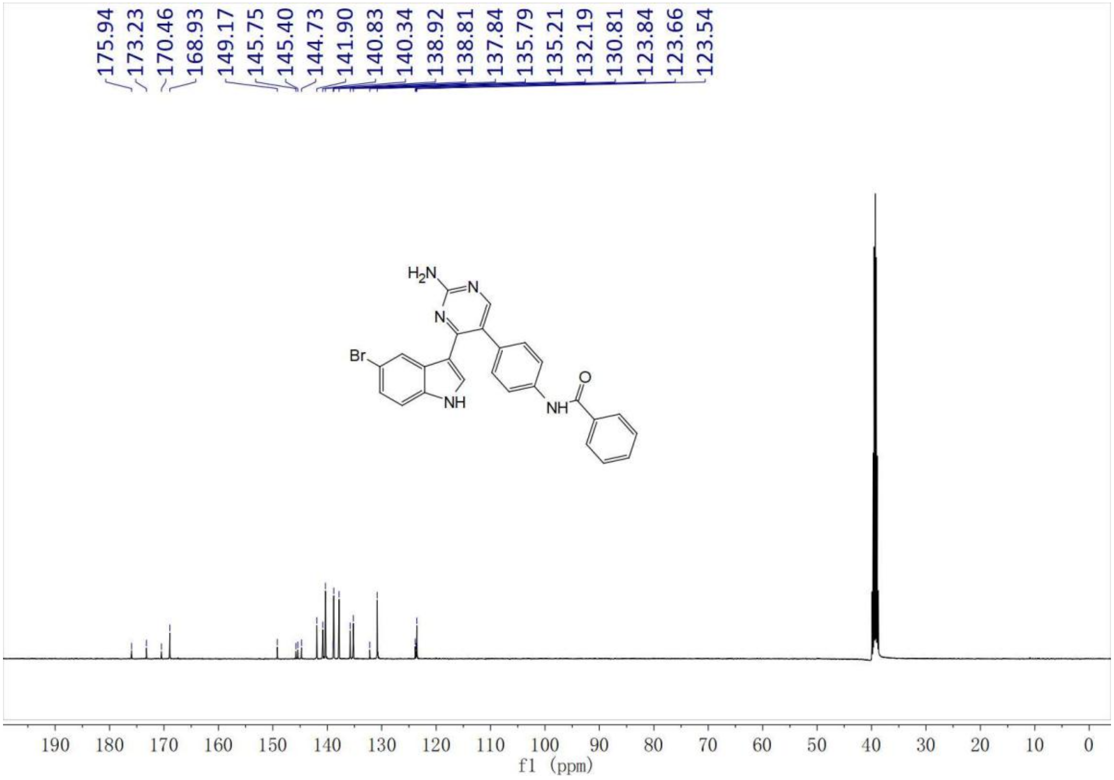

Display Report

Analysis Info

Analysis Name	D:\Data\data\2020\TC2_RA1_01_4183.d	Acquisition Date	10/10/2020 15:07:14 PM
Method	MS-2MIN-POS.m	Operator	BDAL@DE
Sample Name	TC2	Instrument	compact 8255754.20127
Comment			

Acquisition Parameter

Source Type	ESI	Ion Polarity	Positive	Set Nebulizer	2.0 Bar
Focus	Active	Set Capillary	4500 V	Set Dry Heater	200 °C
Scan Begin	50 m/z	Set End Plate Offset	-500 V	Set Dry Gas	8.0 l/min
Scan End	3000 m/z	Set Charging Voltage	2000 V	Set Divert Valve	Waste
		Set Corona	0 nA	Set APCI Heater	0 °C

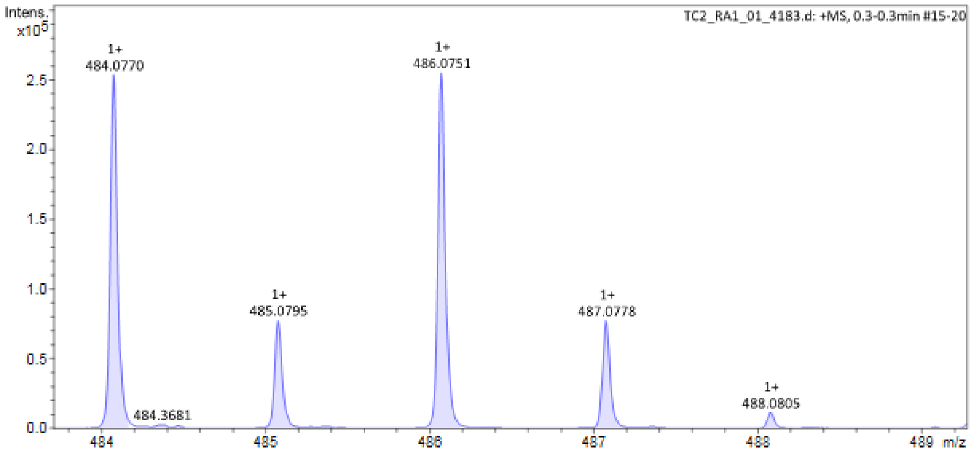

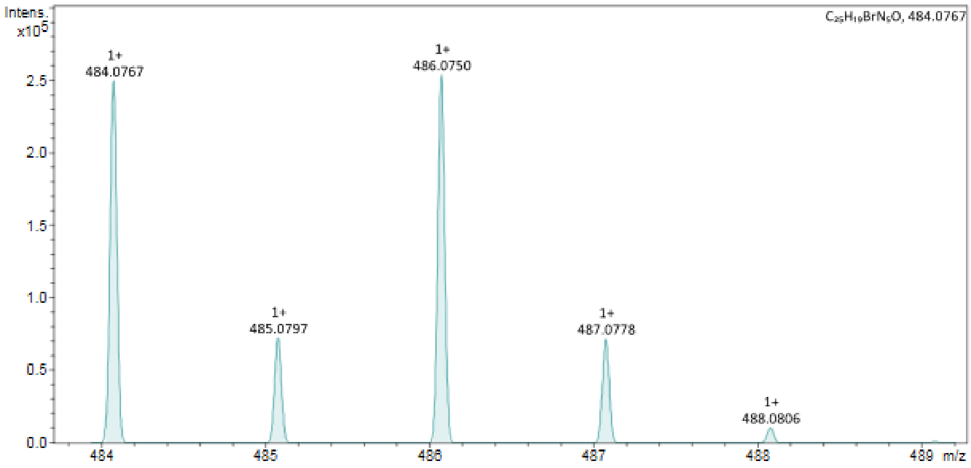

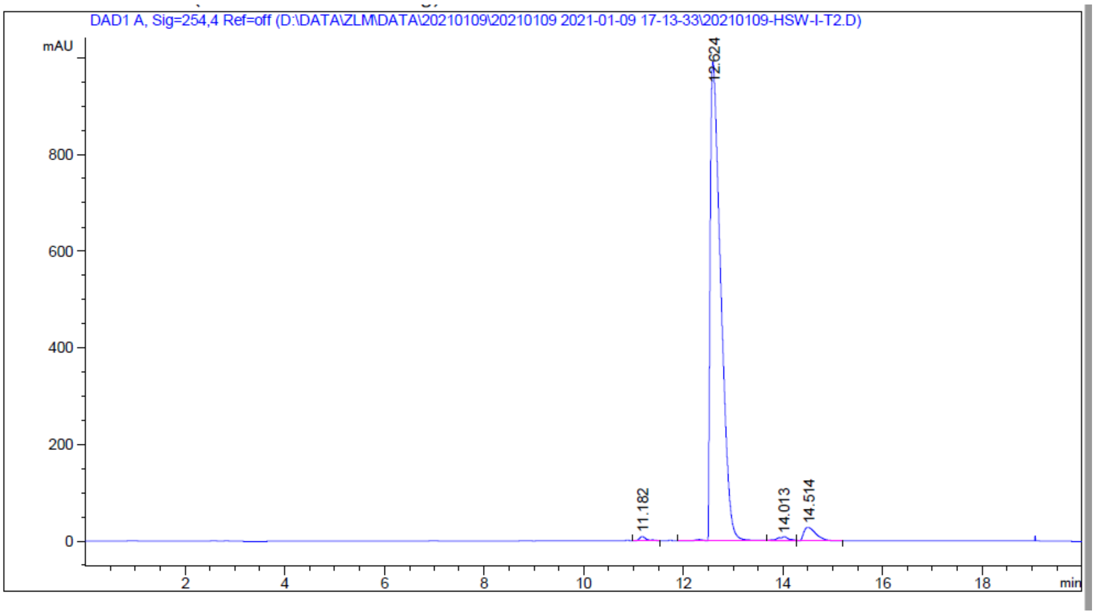

Signal 1: DAD1 A, Sig=254,4 Ref=off

Peak #	RetTime [min]	Type	Width [min]	Area [mAU*s]	Height [mAU]	Area %
1	11.182	BB	0.1513	80.83986	8.47520	0.5320
2	12.624	BV R	0.2356	1.45218e4	943.08911	95.5691
3	14.013	VV E	0.2607	123.24249	7.61800	0.8111
4	14.514	VB	0.2713	469.19122	27.45978	3.0878

Totals : 1.51950e4 986.64209

^{1}H NMR, ^{13}C NMR, HRMS, and HPLC of compound A7

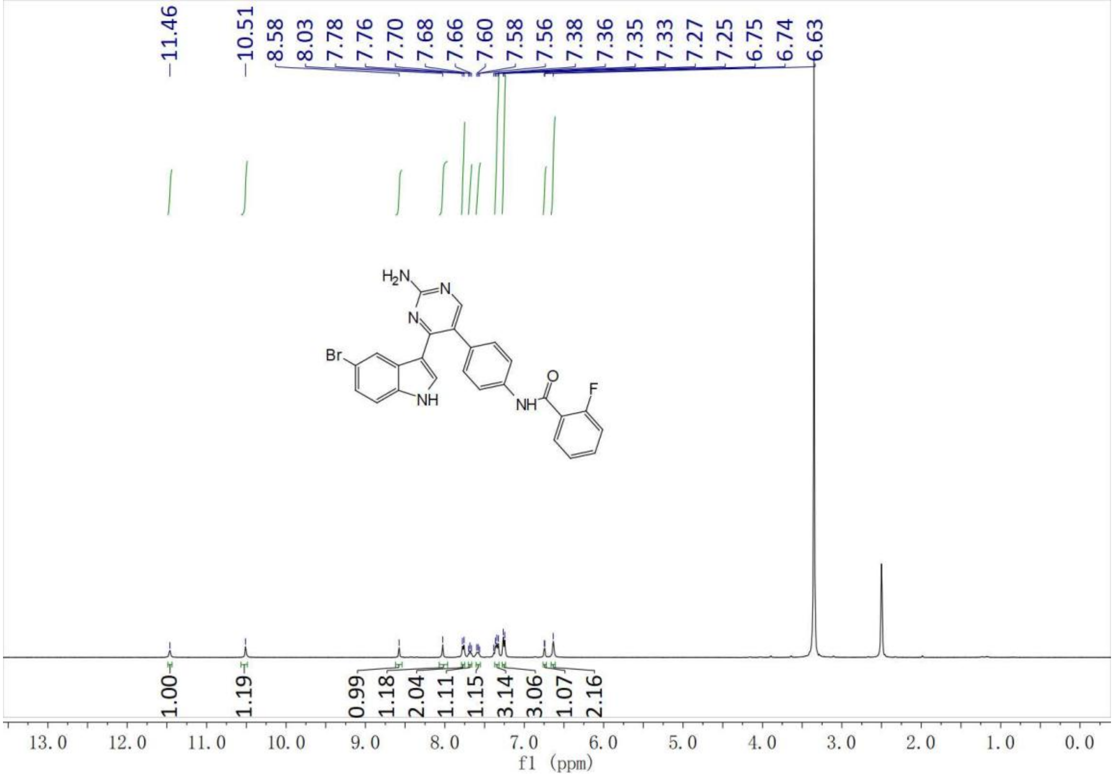

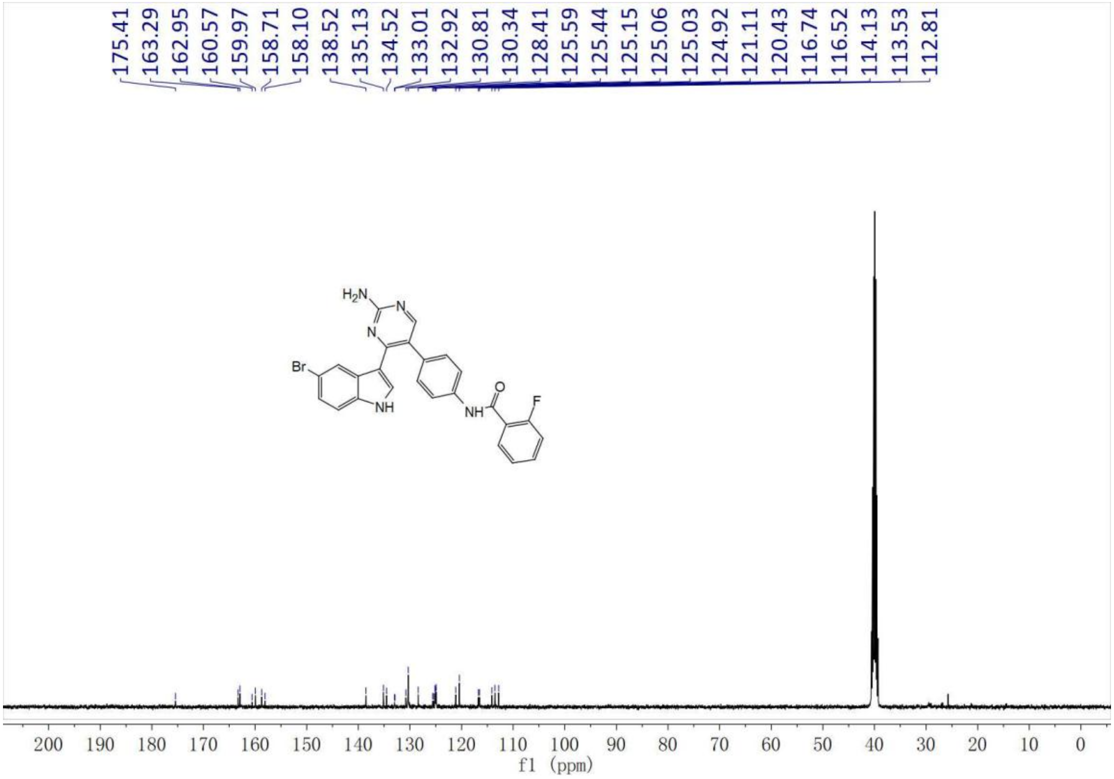

Display Report

Analysis Info

Analysis Name	D:\Data\data\2020\TC10_RB1_01_4191.d	Acquisition Date	10/10/2020 15:29:20 PM
Method	MS-2MIN-POS.m	Operator	BDAL@DE
Sample Name	TC10	Instrument	compact 8255754.20127
Comment			

Acquisition Parameter

Source Type	ESI	Ion Polarity	Positive	Set Nebulizer	2.0 Bar
Focus	Active	Set Capillary	4500 V	Set Dry Heater	200 °C
Scan Begin	50 m/z	Set End Plate Offset	-500 V	Set Dry Gas	8.0 l/min
Scan End	3000 m/z	Set Charging Voltage	2000 V	Set Divert Valve	Waste
		Set Corona	0 nA	Set APCI Heater	0 °C

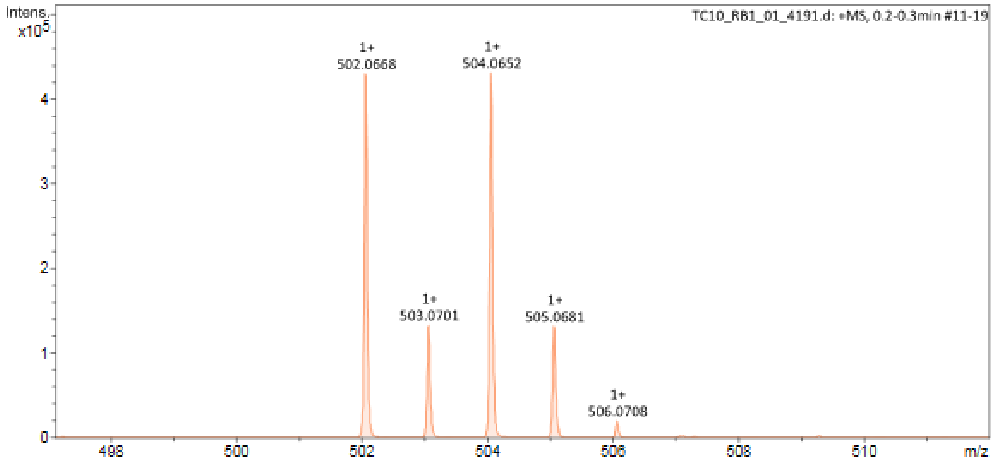

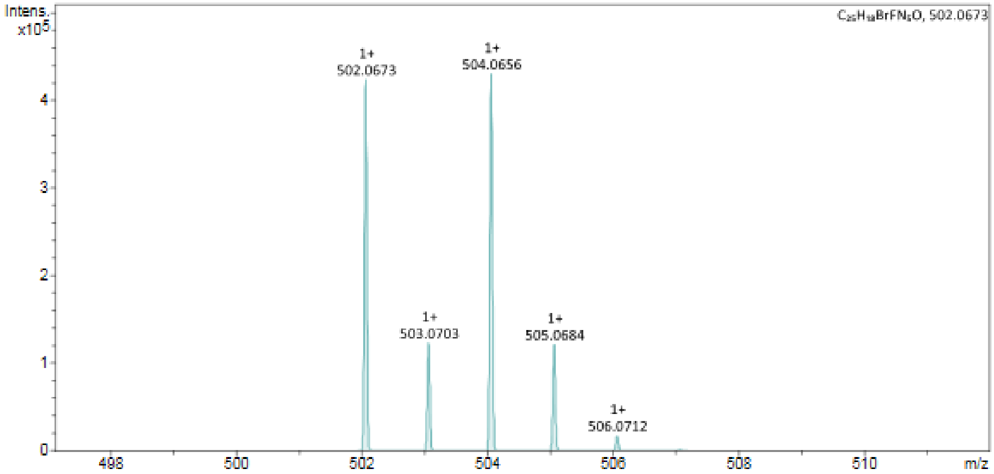

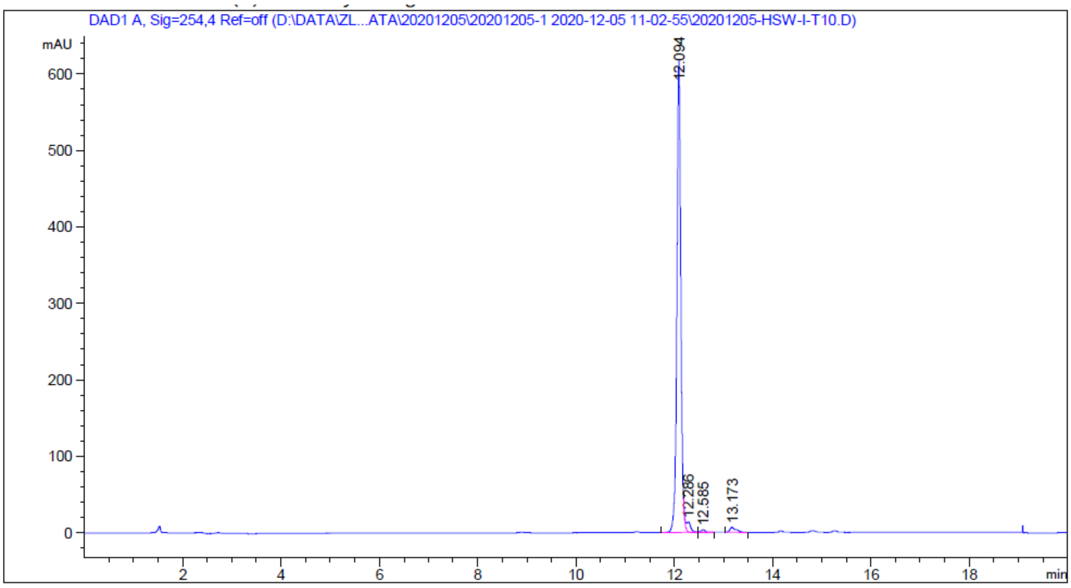

Signal 1: DAD1 A, Sig=254,4 Ref=off

Peak #	RetTime [min]	Type	Width [min]	Area [mAU*s]	Height [mAU]	Area %
1	12.094	BV R	0.0844	3458.89429	617.49146	95.4310
2	12.286	VV E	0.0948	86.72348	13.18068	2.3927
3	12.585	VB E	0.0868	22.22200	3.46552	0.6131

1260R 12/5/2020 2:03:46 PM BY

Data File D:\DATA\ZLM\DATA\20201205\20201205-1 2020-12-05 11-02-55\20201205-HSW-

Sample Name: 20201205-HSW-I-T10

Peak #	RetTime [min]	Type	Width [min]	Area [mAU*s]	Height [mAU]	Area %
4	13.173	BB	0.1175	56.65709	6.38742	1.5632

^{1}H NMR, ^{13}C NMR, HRMS, and HPLC of compound A8

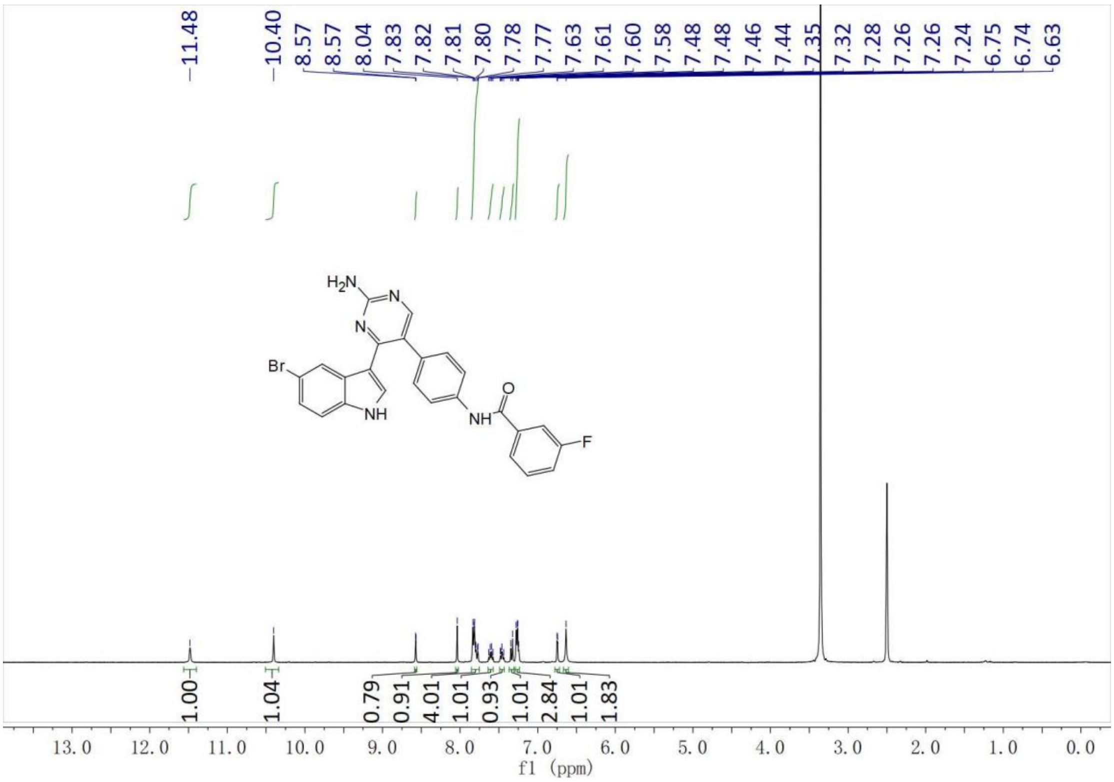

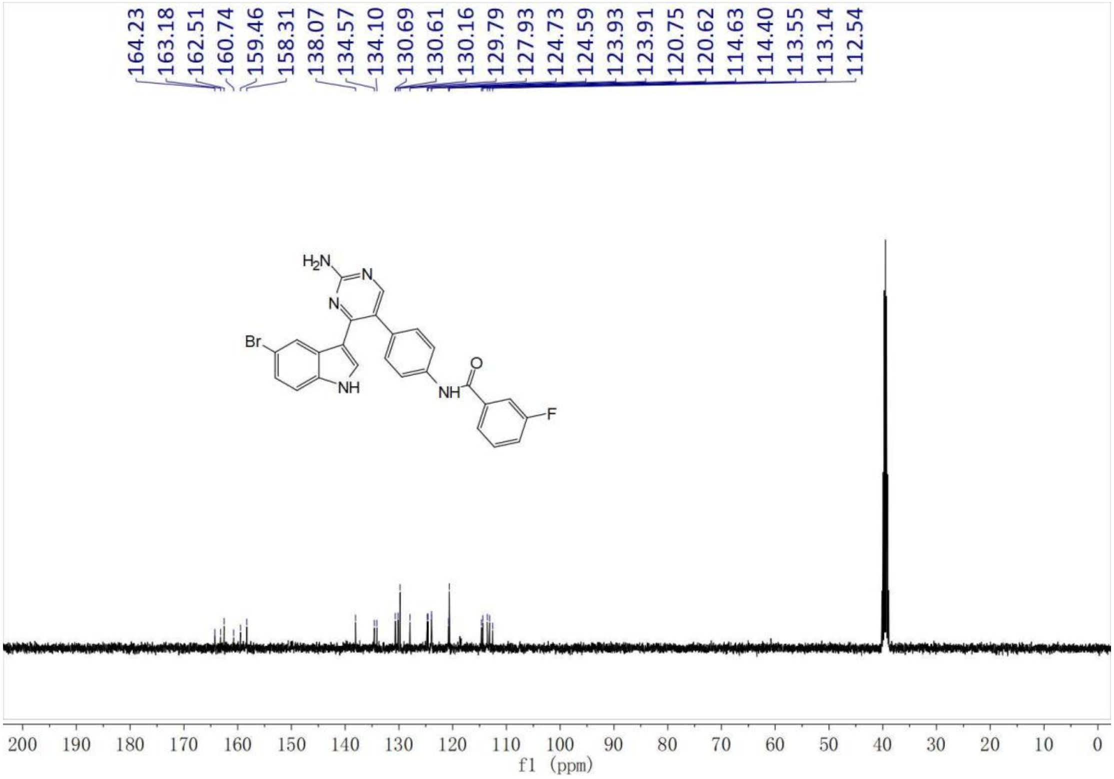

Display Report

Analysis Info

Analysis Name	D:\Data\data\2020\TC9_RA8_01_4190.d	Acquisition Date	10/10/2020 15:26:34 PM
Method	MS-2MIN-POS.m	Operator	BDAL@DE
Sample Name	TC9	Instrument	compact 8255754.20127
Comment			

Acquisition Parameter

Source Type	ESI	Ion Polarity	Positive	Set Nebulizer	2.0 Bar
Focus	Active	Set Capillary	4500 V	Set Dry Heater	200 °C
Scan Begin	50 m/z	Set End Plate Offset	-500 V	Set Dry Gas	8.0 l/min
Scan End	3000 m/z	Set Charging Voltage	2000 V	Set Divert Valve	Waste
		Set Corona	0 nA	Set APCI Heater	0 °C

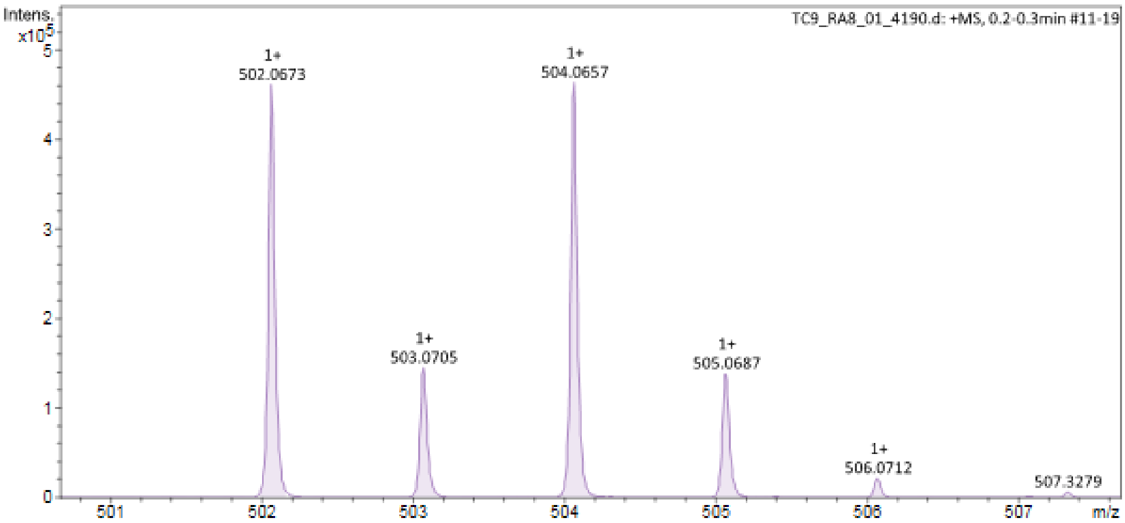

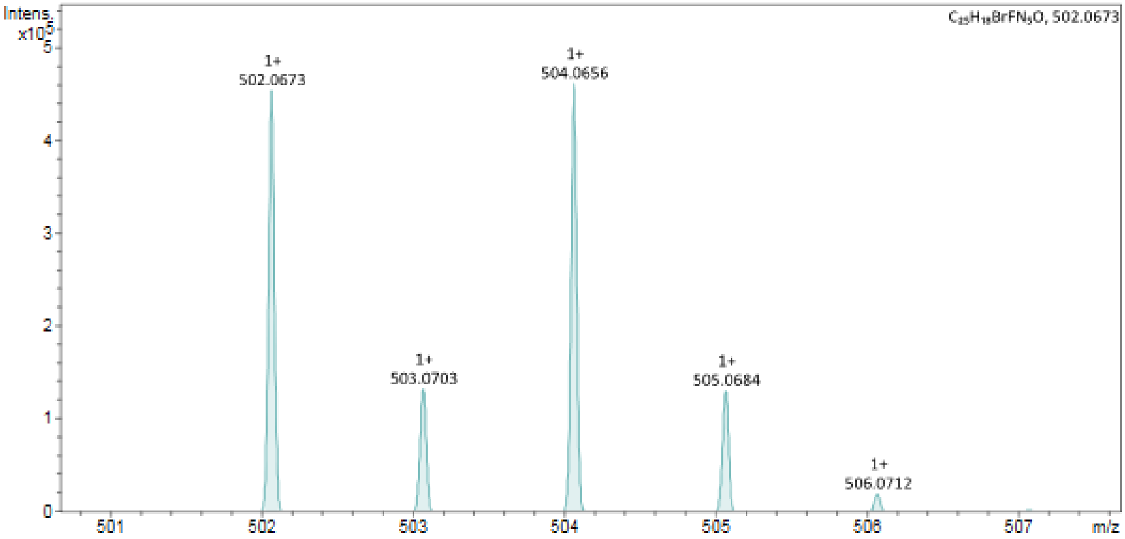

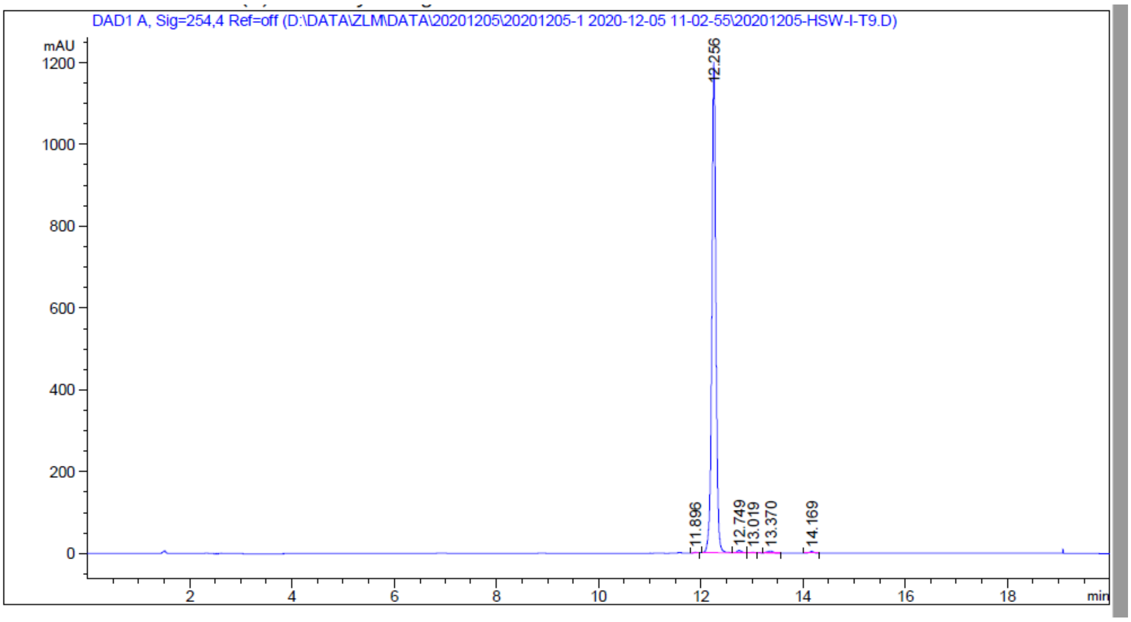

Signal 1: DAD1 A, Sig=254,4 Ref=off

Peak #	RetTime [min]	Type	Width [min]	Area [mAU*s]	Height [mAU]	Area %
1	11.896	BB	0.0598	7.30479	1.72726	0.1075
2	12.256	BV R	0.0834	6671.84082	1199.86926	98.1857
3	12.749	VV E	0.0885	37.35518	6.13798	0.5497

1260R 12/5/2020 9:16:42 PM BY

Data File D:\DATA\ZLM\DATA\20201205\20201205-1 2020-12-05 11-02-55\2020120[illegible]
Sample Name: 20201205-HSW-I-T9

Peak #	RetTime [min]	Type	Width [min]	Area [mAU*s]	Height [mAU]	Area %
4	13.019	VV E	0.0816	12.48004	1.91310	0.1837
5	13.370	VB E	0.1156	40.53680	4.77001	0.5966
6	14.169	BB	0.0809	25.61051	4.40070	0.3769

Totals : 6795.12814 1218.81830

^{1}H NMR, ^{13}C NMR, HRMS, and HPLC of compound A9

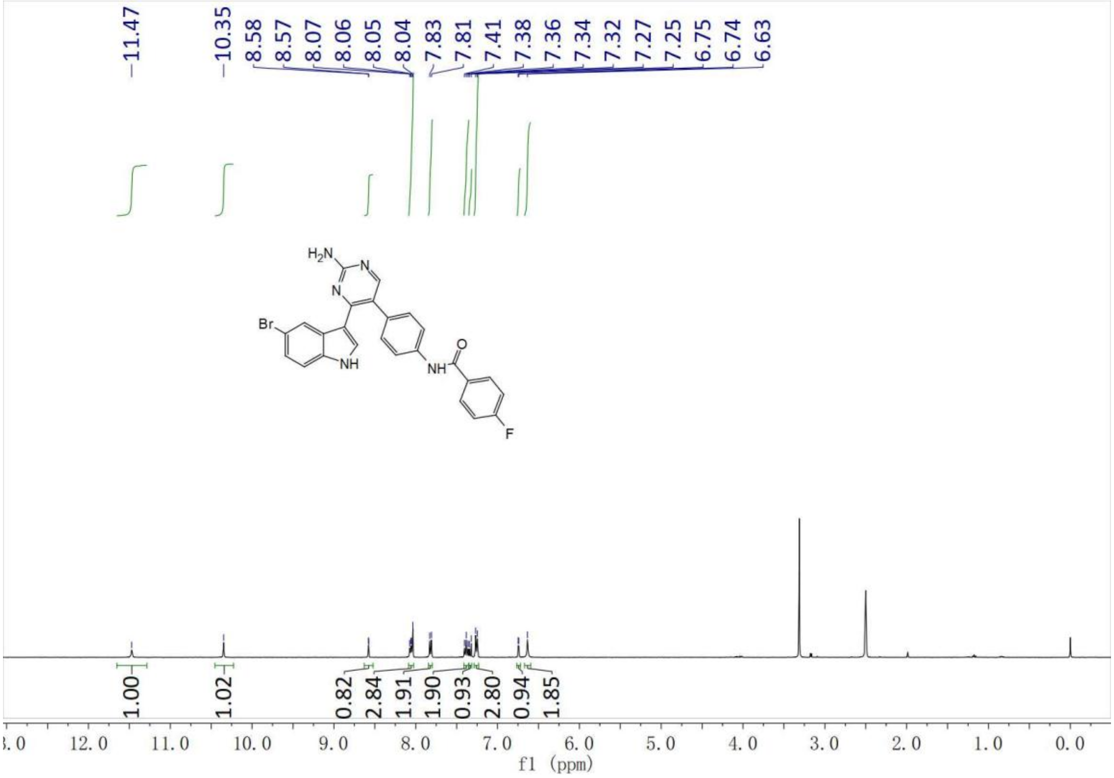

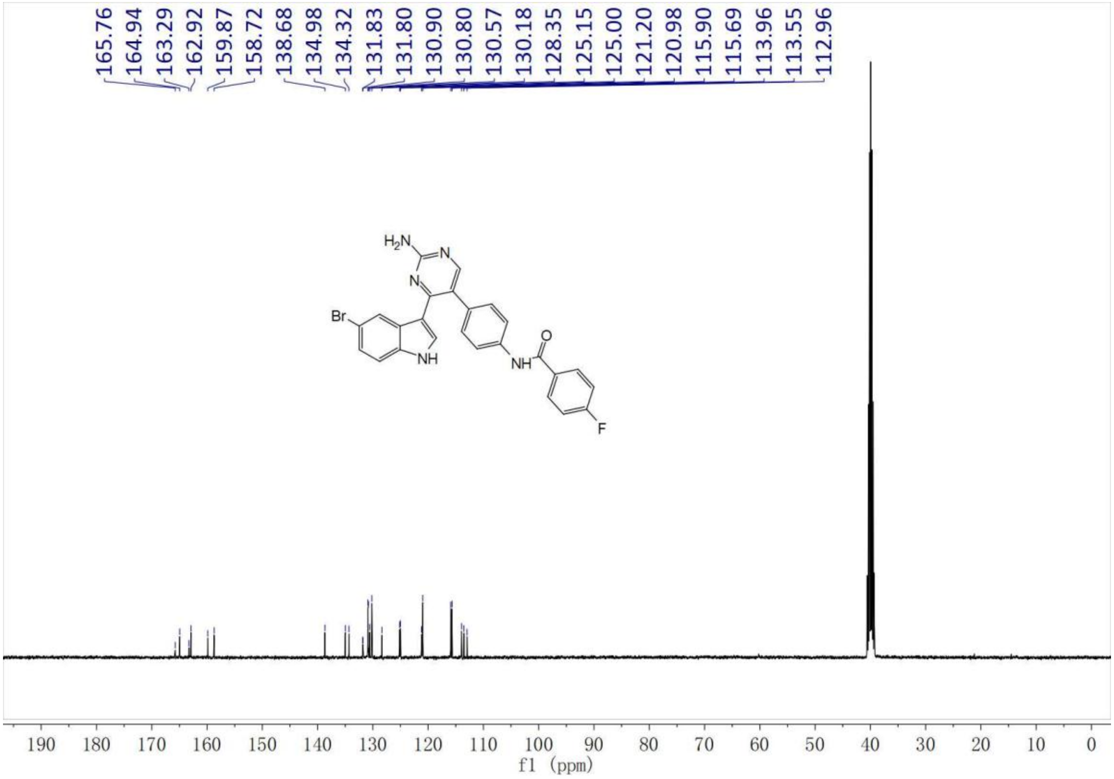

Display Report

Analysis Info

Analysis Name	D:\Data\data\2020\TC3_RA2_01_4184.d	Acquisition Date	10/10/2020 15:10:01 PM
Method	MS-2MIN-POS.m	Operator	BDAL@DE
Sample Name	TC3	Instrument	compact 8255754.20127
Comment			

Acquisition Parameter

Source Type	ESI	Ion Polarity	Positive	Set Nebulizer	2.0 Bar
Focus	Active	Set Capillary	4500 V	Set Dry Heater	200 °C
Scan Begin	50 m/z	Set End Plate Offset	-500 V	Set Dry Gas	8.0 l/min
Scan End	3000 m/z	Set Charging Voltage	2000 V	Set Divert Valve	Waste
		Set Corona	0 nA	Set APCI Heater	0 °C

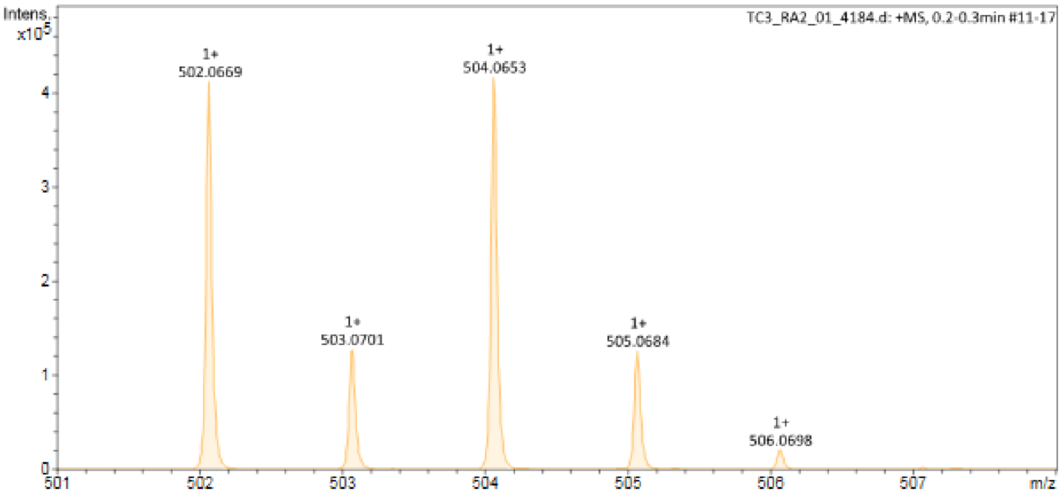

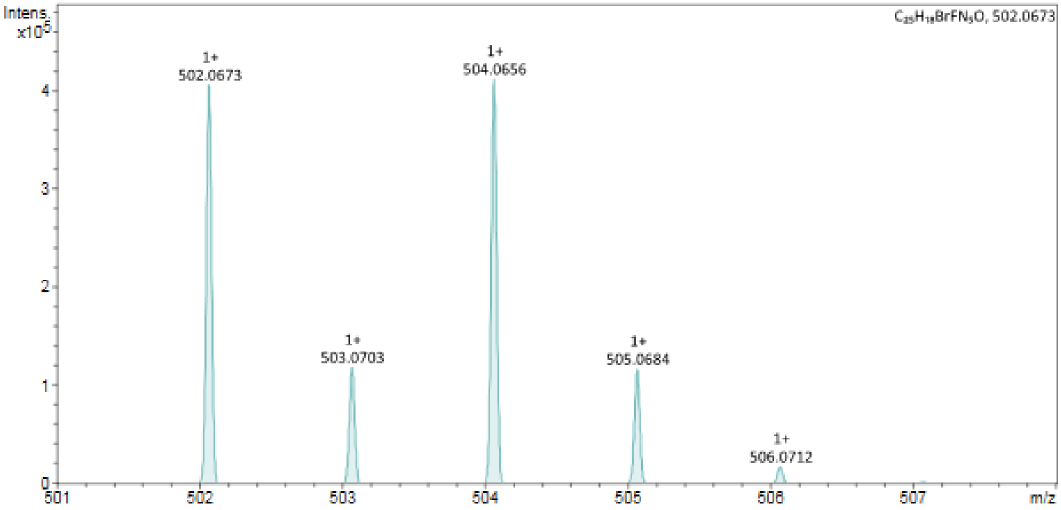

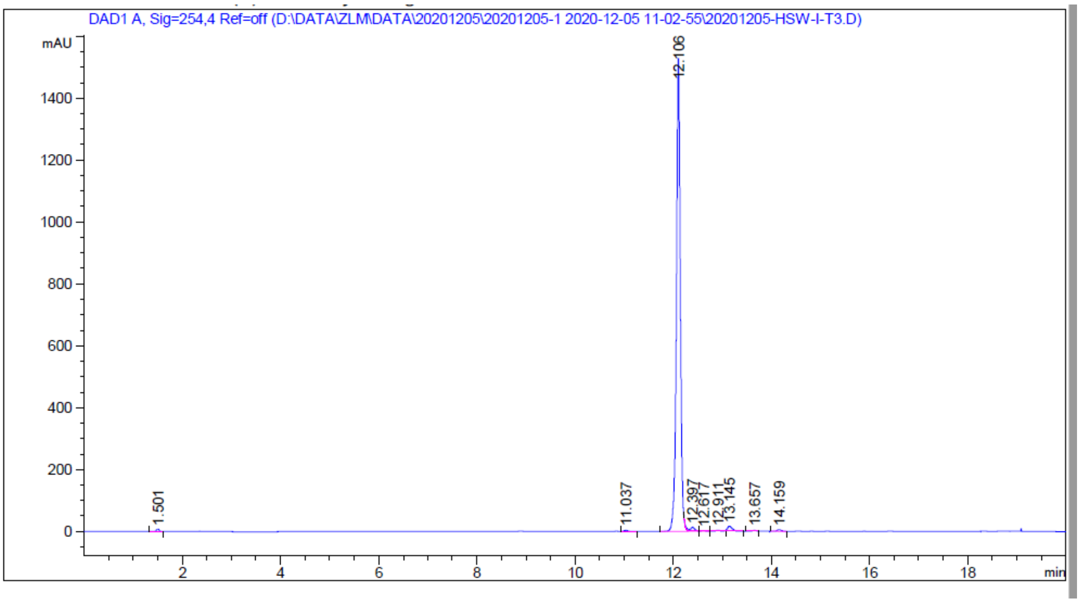

Signal 1: DAD1 A, Sig=254,4 Ref=off

Peak #	RetTime [min]	Type	Width [min]	Area [mAU*s]	Height [mAU]	Area %
1	1.501	BB	0.0639	28.51104	7.24421	0.3212
2	11.037	VB	0.0832	21.22701	3.74207	0.2392
3	12.106	BV R	0.0841	8518.53906	1526.94849	95.9736

1260R 12/5/2020 9:14:12 PM BY

Data File D:\DATA\ZLM\DATA\20201205\20201205-1 2020-12-05 11-02-55\20
Sample Name: 20201205-HSW-I-T3

Peak #	RetTime [min]	Type	Width [min]	Area [mAU*s]	Height [mAU]	Area %
4	12.397	VV E	0.1061	101.76255	13.10434	1.1465
5	12.617	VB E	0.0803	14.52352	2.26483	0.1636
6	12.911	BV E	0.0816	18.66226	3.26769	0.2103
7	13.145	VB R	0.1027	119.04343	16.60013	1.3412
8	13.657	BV	0.0808	19.63878	3.16800	0.2213
9	14.159	BB	0.0860	34.00940	5.83180	0.3832
Totals :				8875.91706	1582.17157	

^{1}H NMR, ^{13}C NMR, HRMS, and HPLC of compound A10

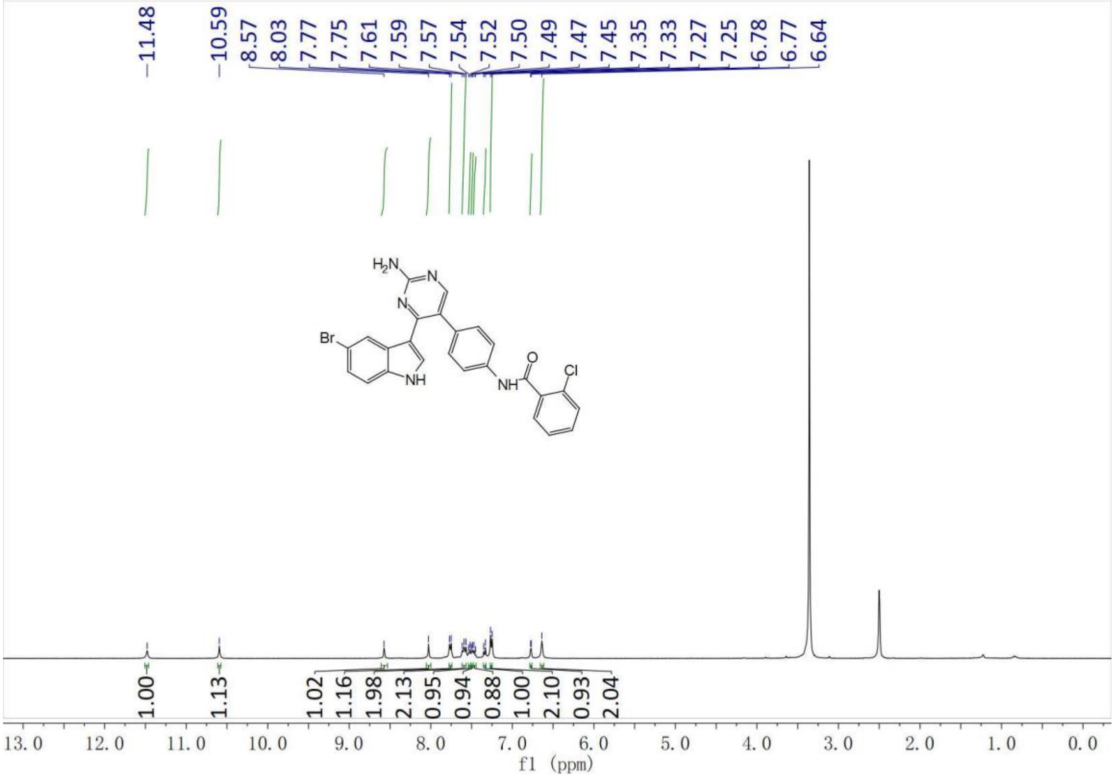

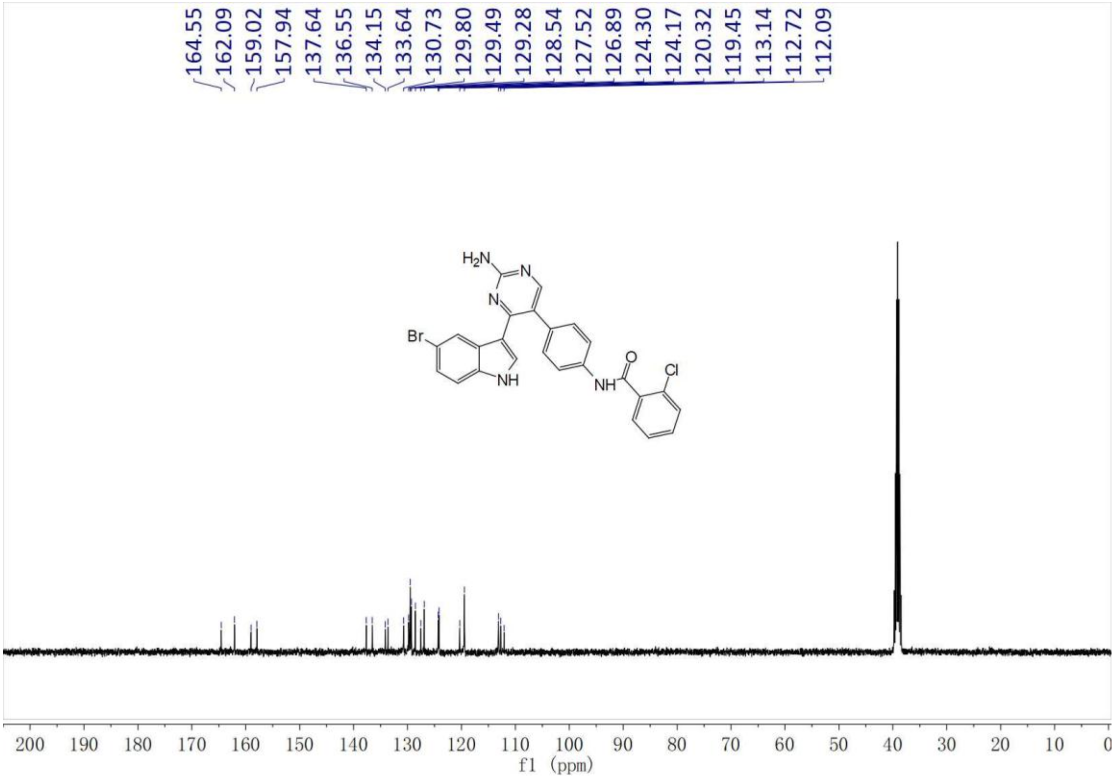

Display Report

Analysis Info

Analysis Name	D:\Data\data\2020\TC11_RB2_01_4192.d	Acquisition Date	10/10/2020 15:32:07 PM
Method	MS-2MIN-POS.m	Operator	BDAL@DE
Sample Name	TC11	Instrument	compact 8255754.20127
Comment			

Acquisition Parameter

Source Type	ESI	Ion Polarity	Positive	Set Nebulizer	2.0 Bar
Focus	Active	Set Capillary	4500 V	Set Dry Heater	200 °C
Scan Begin	50 m/z	Set End Plate Offset	-500 V	Set Dry Gas	8.0 l/min
Scan End	3000 m/z	Set Charging Voltage	2000 V	Set Divert Valve	Waste
		Set Corona	0 nA	Set APCI Heater	0 °C

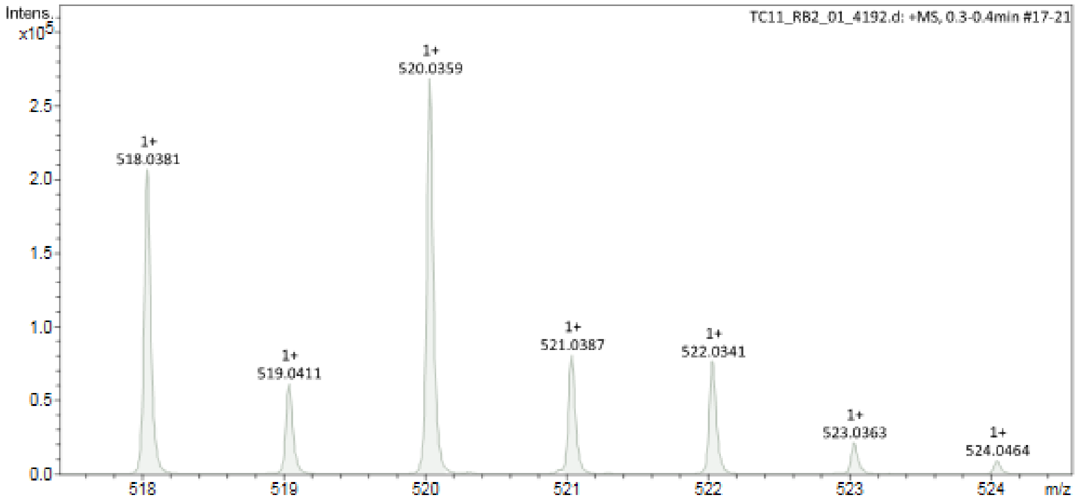

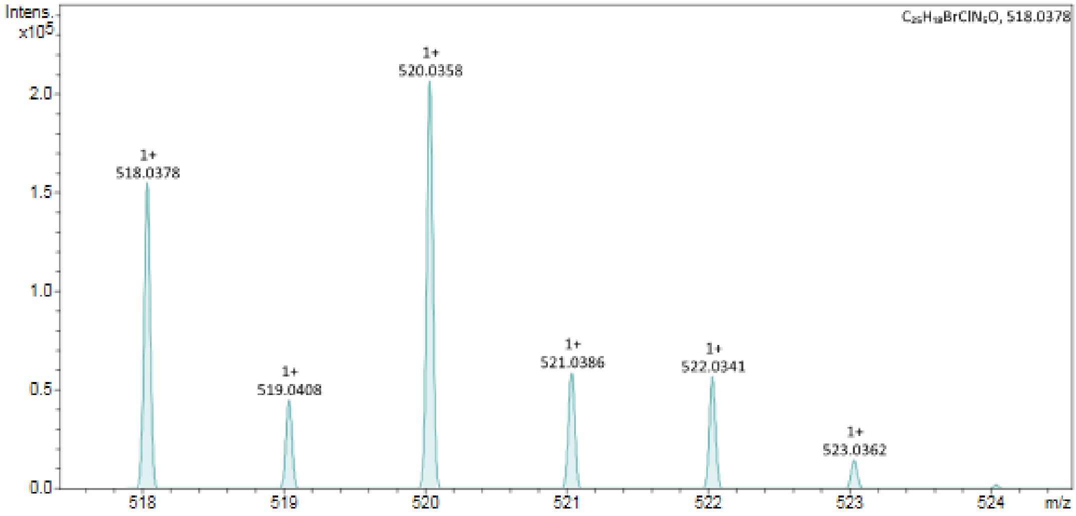

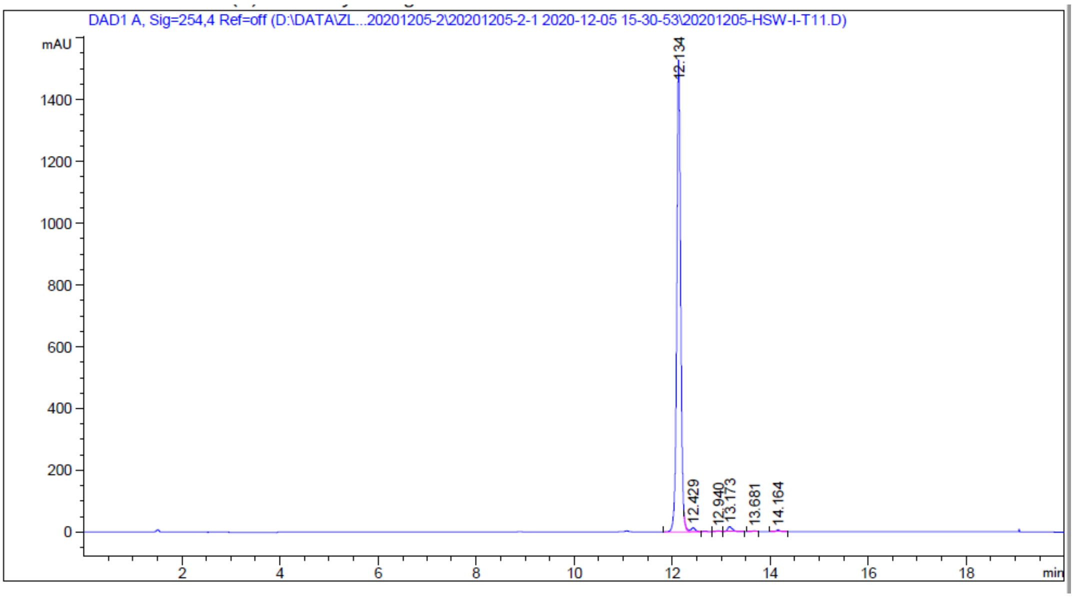

Signal 1: DAD1 A, Sig=254,4 Ref=off

Peak #	RetTime [min]	Type	Width [min]	Area [mAU*s]	Height [mAU]	Area %
1	12.134	BV R	0.0837	8494.28418	1528.83093	96.6402
2	12.429	VV E	0.1096	103.30863	13.09152	1.1754
3	12.940	BV	0.0843	19.79194	3.35801	0.2252

1260R 12/5/2020 9:07:07 PM BY

Data File D:\DATA\ZL...TA\20201205-2\20201205-2-1 2020-12-05 15-30-53\202(
Sample Name: 20201205-HSW-I-T11

Peak #	RetTime [min]	Type	Width [min]	Area [mAU*s]	Height [mAU]	Area %
4	13.173	VB	0.1004	118.62800	16.58551	1.3496
5	13.681	BV	0.0849	19.60647	3.17963	0.2231
6	14.164	BB	0.0877	33.97756	5.80934	0.3866

Totals : 8789.59678 1570.85495

^{1}H NMR, ^{13}C NMR, HRMS, and HPLC of compound A11

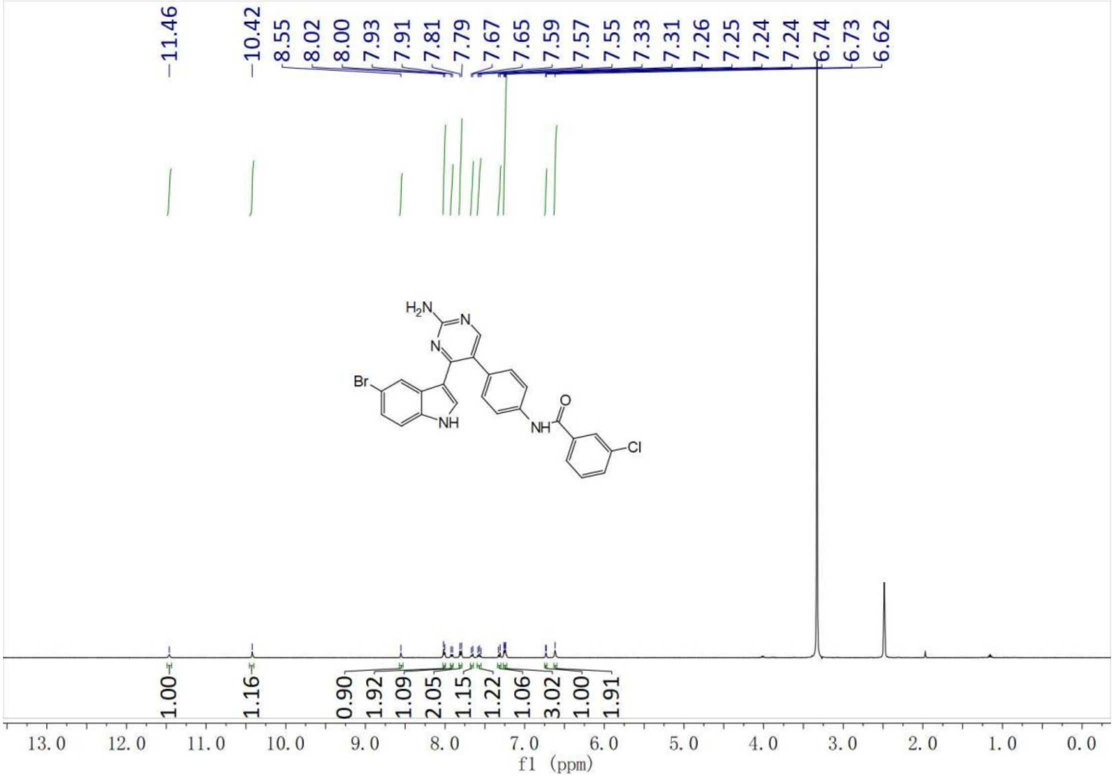

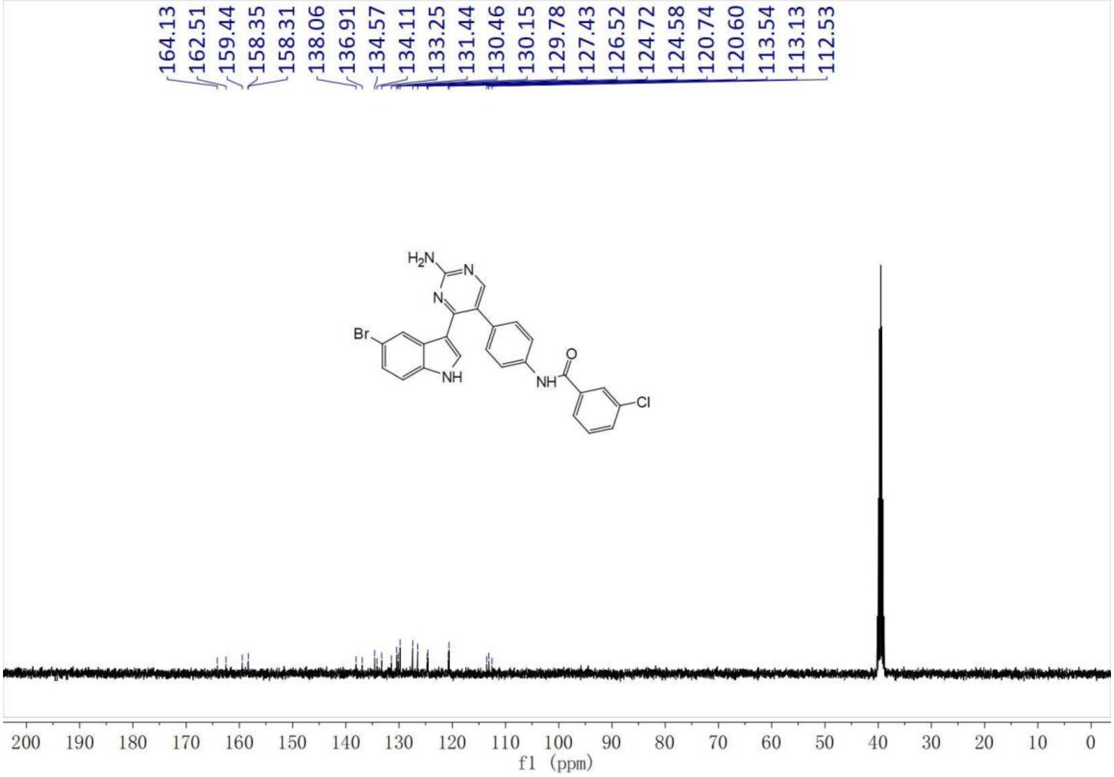

Display Report

Analysis Info

Analysis Name	D:\Data\data\2020\TC12_RB3_01_4193.d	Acquisition Date	10/10/2020 15:34:51 PM
Method	MS-2MIN-POS.m	Operator	BDAL@DE
Sample Name	TC12	Instrument	compact 8255754.20127
Comment			

Acquisition Parameter

Source Type	ESI	Ion Polarity	Positive	Set Nebulizer	2.0 Bar
Focus	Active	Set Capillary	4500 V	Set Dry Heater	200 °C
Scan Begin	50 m/z	Set End Plate Offset	-500 V	Set Dry Gas	8.0 l/min
Scan End	3000 m/z	Set Charging Voltage	2000 V	Set Divert Valve	Waste
		Set Corona	0 nA	Set APCI Heater	0 °C

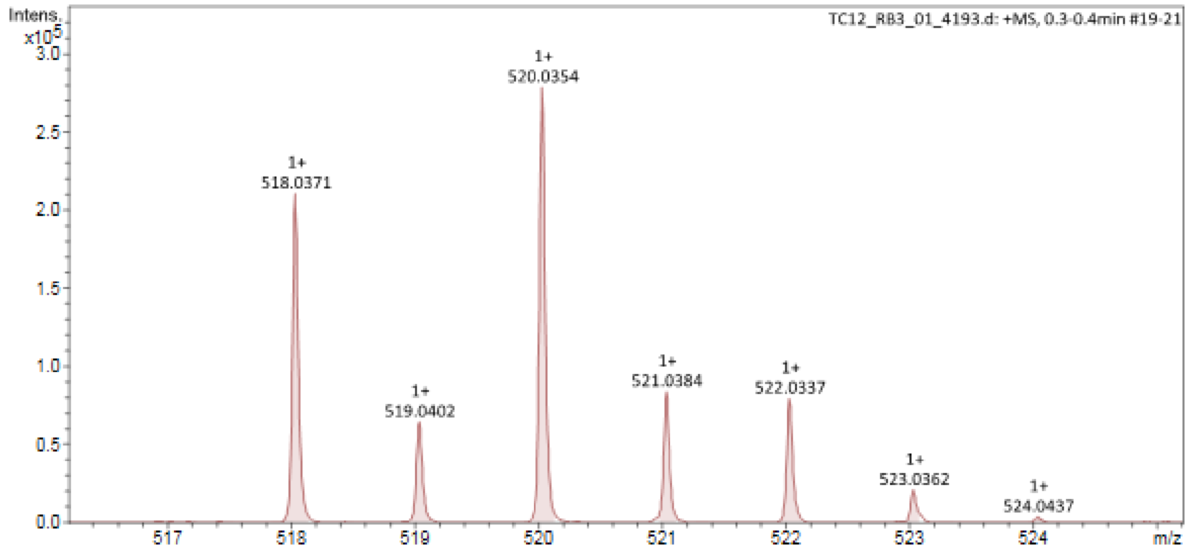

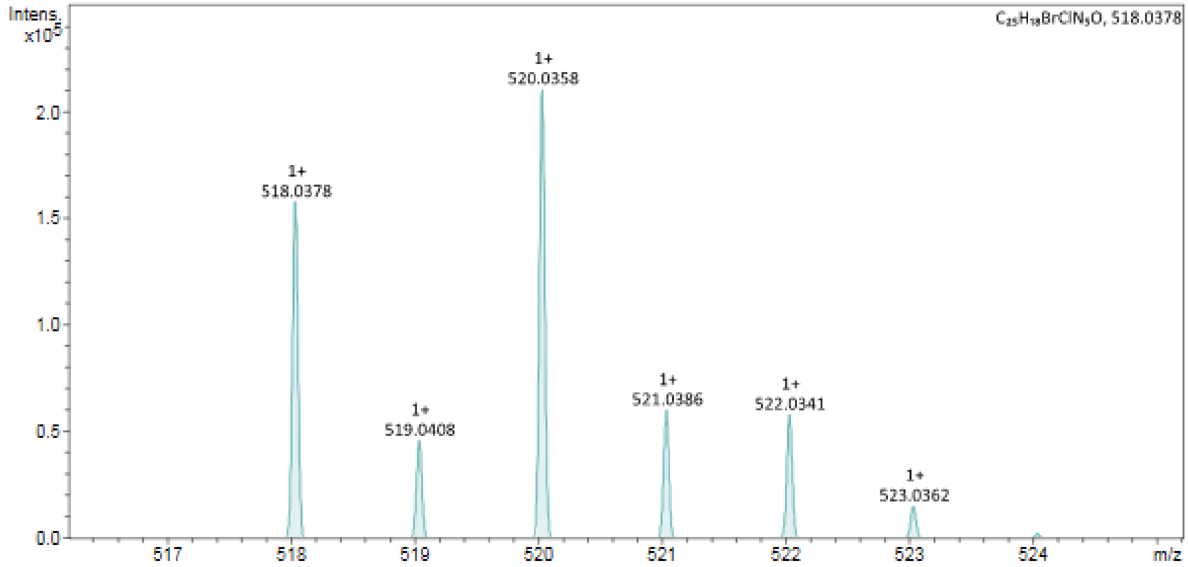

[illegible]

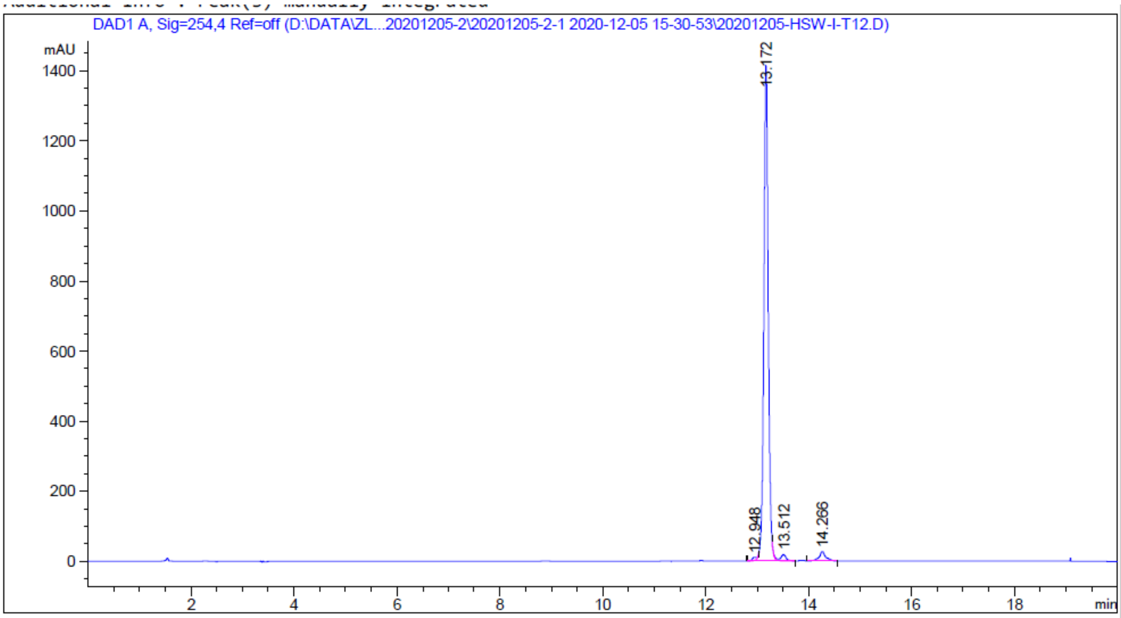

Signal 1: DAD1 A, Sig=254,4 Ref=off

Peak #	RetTime [min]	Type	Width [min]	Area [mAU*s]	Height [mAU]	Area %
1	12.948	BV E	0.0771	51.27475	9.87548	0.5739
2	13.172	VV R	0.0907	8501.55273	1412.72144	95.1580
3	13.512	VB E	0.1167	146.37138	18.03785	1.6383

1260R 12/5/2020 9:03:30 PM BY

Data File D:\DATA\ZL...TA\20201205-2\20201205-2-1 2020-12-05 15-30-53\20[illegible]
Sample Name: 20201205-HSW-I-T12

Peak #	RetTime [min]	Type	Width [min]	Area [mAU*s]	Height [mAU]	Area %
4	14.266	BB	0.1218	234.94702	27.18323	2.6298

Totals :				8934.14589	1467.81800	

^{1}H NMR, ^{13}C NMR, HRMS, and HPLC of compound A12

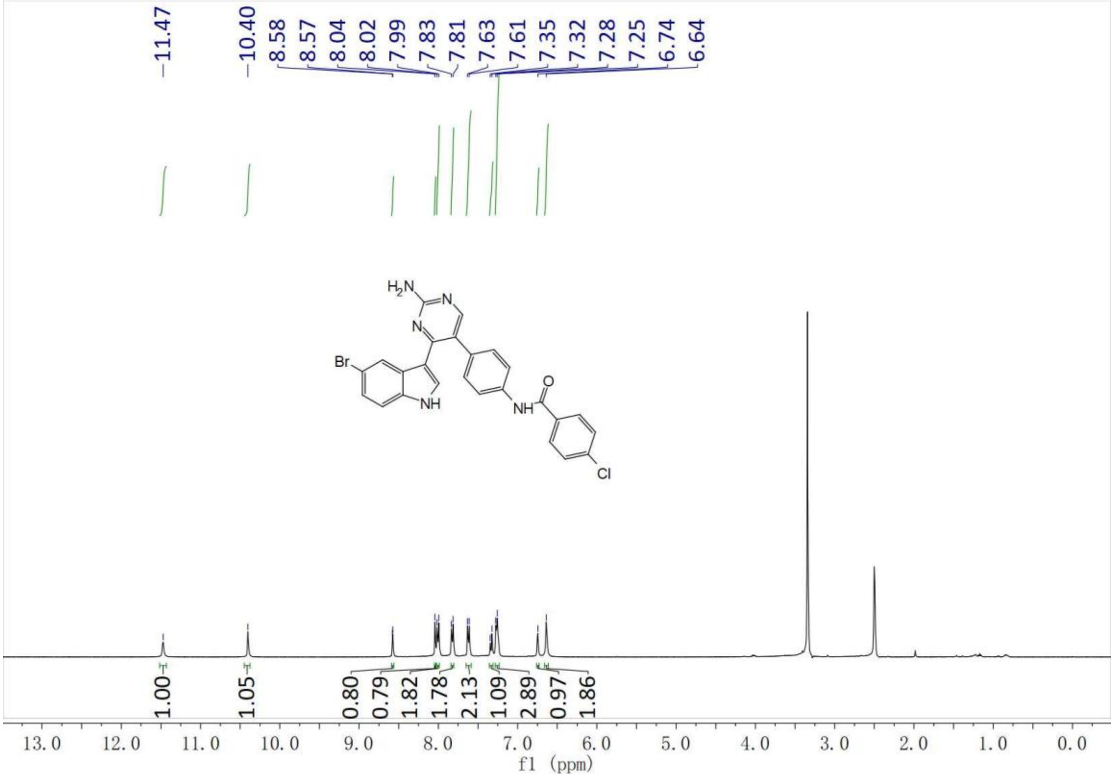

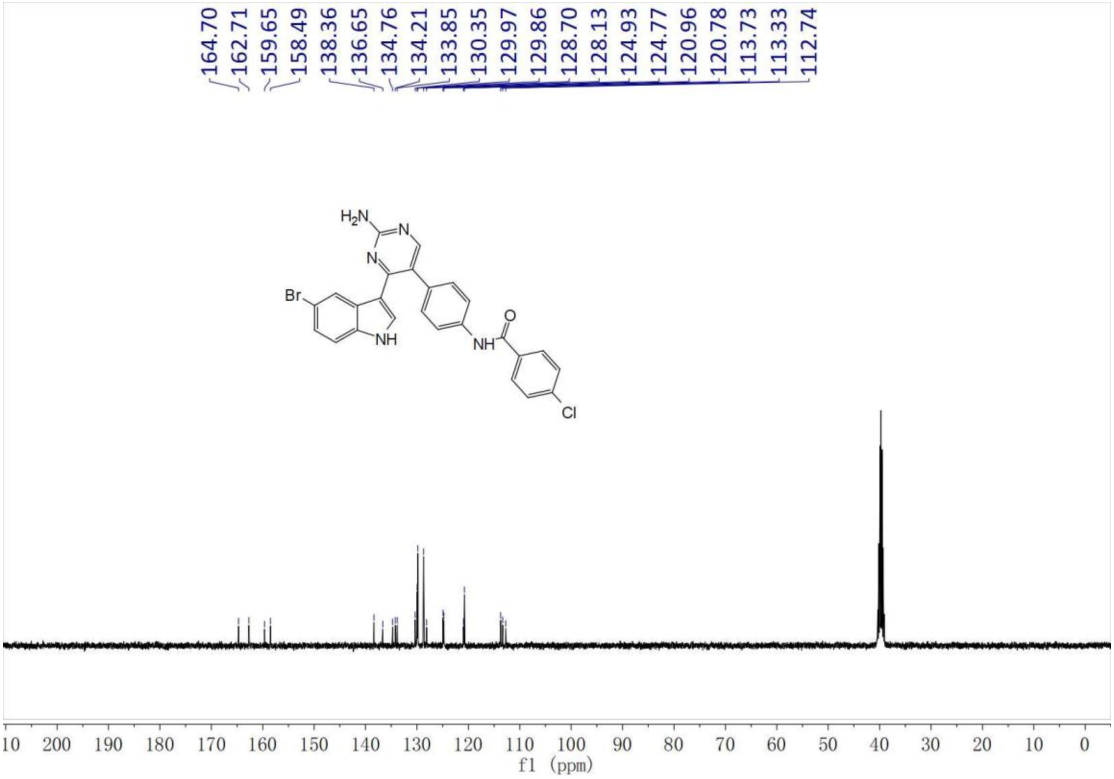

Display Report

Analysis Info

Analysis Name	D:\Data\data\2020\TC4_RA3_01_4185.d	Acquisition Date	10/10/2020 15:12:45 PM
Method	MS-2MIN-POS.m	Operator	BDAL@DE
Sample Name	TC4	Instrument	compact 8255754.20127
Comment			

Acquisition Parameter

Source Type	ESI	Ion Polarity	Positive	Set Nebulizer	2.0 Bar
Focus	Active	Set Capillary	4500 V	Set Dry Heater	200 °C
Scan Begin	50 m/z	Set End Plate Offset	-500 V	Set Dry Gas	8.0 l/min
Scan End	3000 m/z	Set Charging Voltage	2000 V	Set Divert Valve	Waste
		Set Corona	0 nA	Set APCI Heater	0 °C

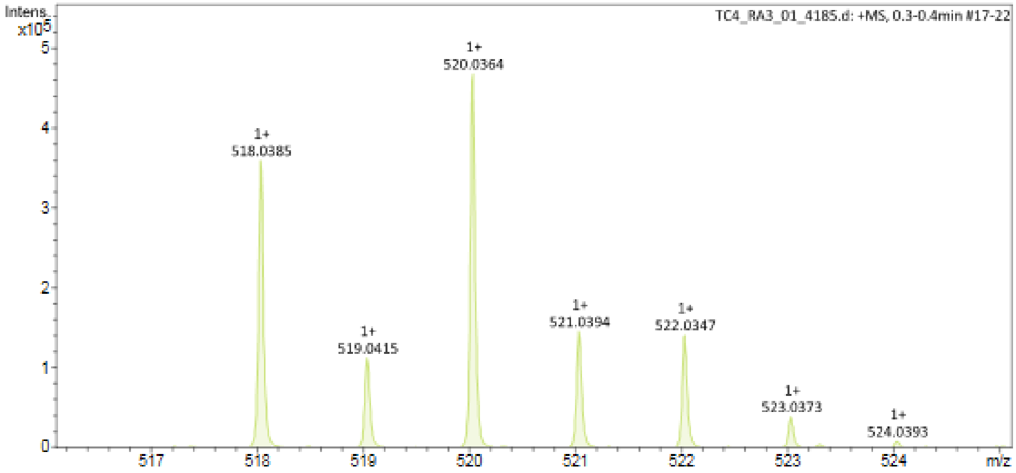

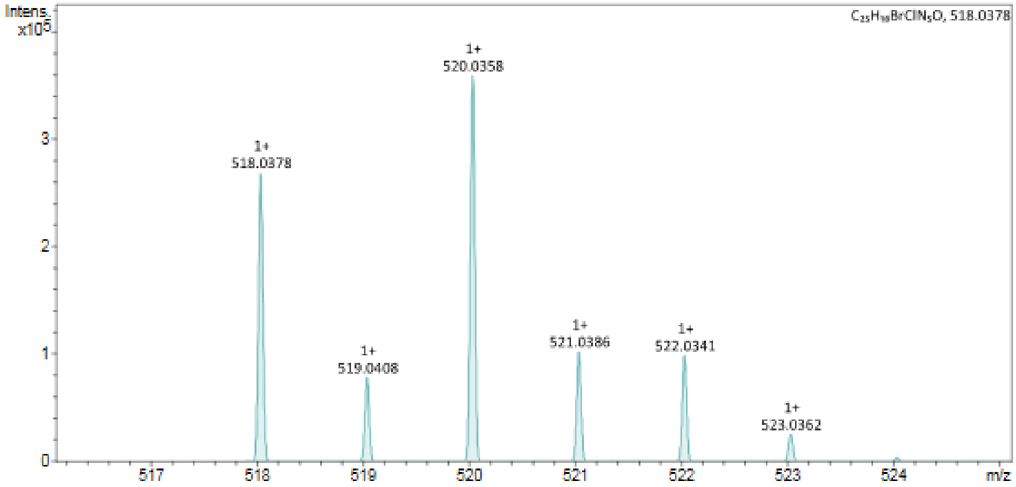

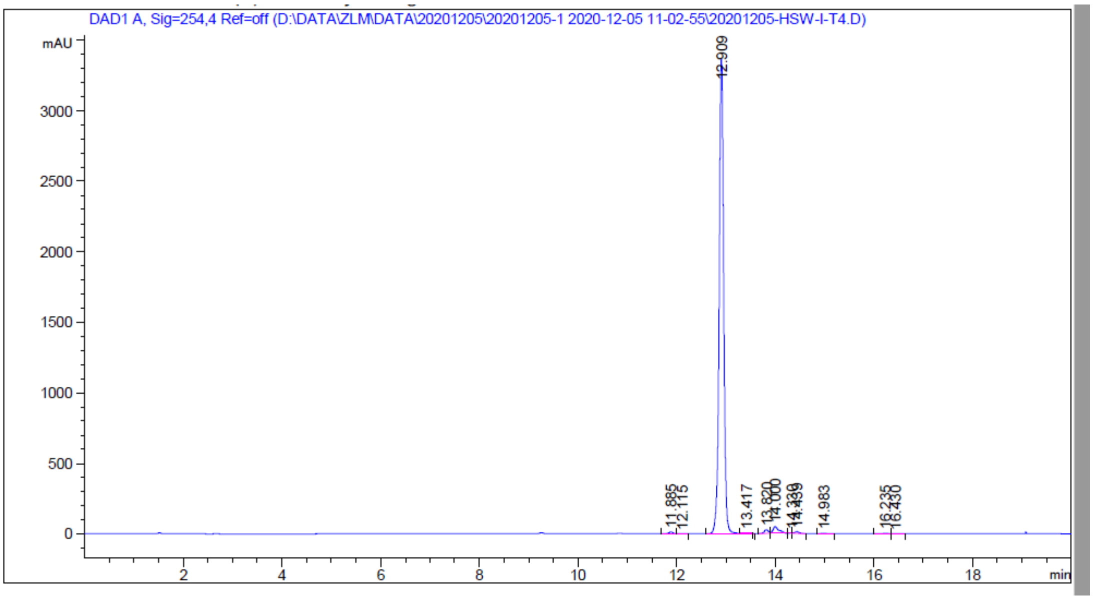

Signal 1: DAD1 A, Sig=254,4 Ref=off

Peak #	RetTime [min]	Type	Width [min]	Area [mAU*s]	Height [mAU]	Area %
1	11.885	BV R	0.0834	77.39366	13.91382	0.3485
2	12.115	VB E	0.0716	7.07631	1.22057	0.0319
3	12.909	BV R	0.0960	2.13651e4	3366.70801	96.2081

1260R 12/5/2020 9:15:14 PM BY

Data File D:\DATA\ZLM\DATA\20201205\20201205-1 2020-12-05 11-02
Sample Name: 20201205-HSW-I-T4

Peak #	RetTime [min]	Type	Width [min]	Area [mAU*s]	Height [mAU]	Area %
4	13.417	VB E	0.1005	25.83179	3.18865	0.1163
5	13.820	BV	0.0896	160.12170	26.63392	0.7210
6	14.000	VB	0.1180	413.76163	49.01915	1.8632
7	14.330	BV	0.0439	12.57234	3.45583	0.0566
8	14.439	VB	0.0965	87.85233	13.13737	0.3956
9	14.983	VB	0.0976	21.34725	2.82673	0.0961
10	16.235	BV	0.1017	22.49681	2.64625	0.1013
11	16.430	VB	0.0974	13.61478	1.66473	0.0613

^{1}H NMR, ^{13}C NMR, HRMS, and HPLC of compound A13

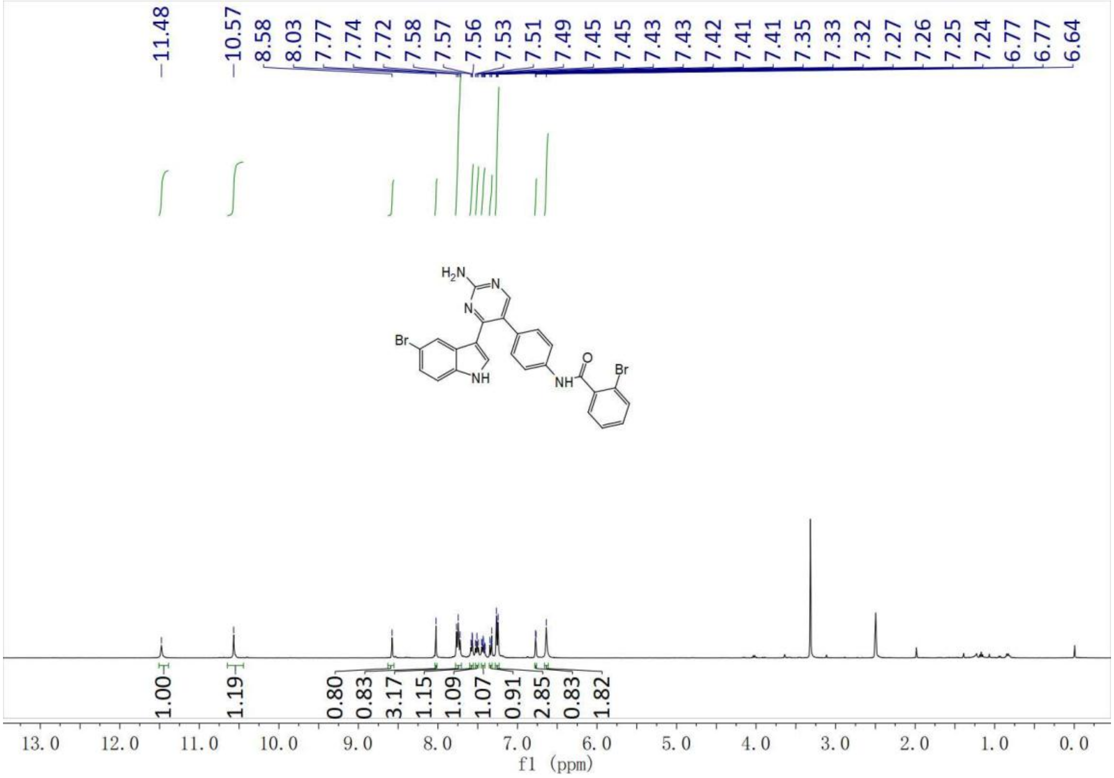

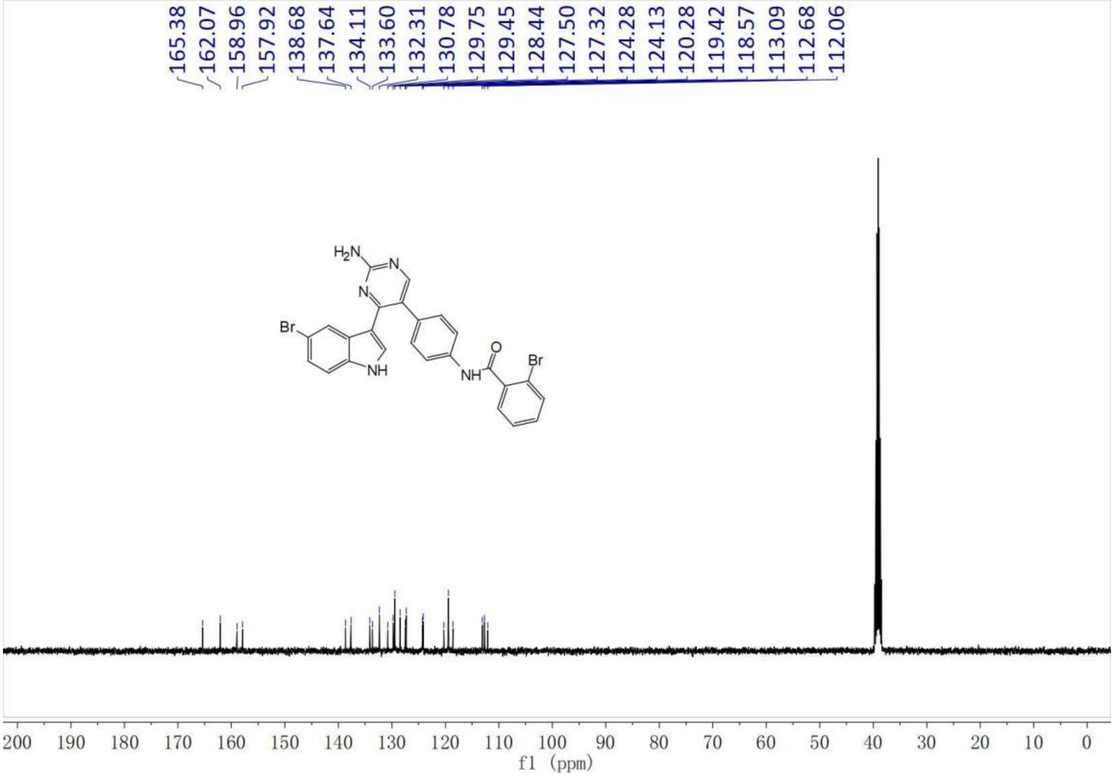

Display Report

Analysis Info

Analysis Name	D:\Data\data\2020\TC13_RB4_01_4194.d	Acquisition Date	10/10/2020 15:37:37 PM
Method	MS-2MIN-POS.m	Operator	BDAL@DE
Sample Name	TC13	Instrument	compact 8255754.20127
Comment			

Acquisition Parameter

Source Type	ESI	Ion Polarity	Positive	Set Nebulizer	2.0 Bar
Focus	Active	Set Capillary	4500 V	Set Dry Heater	200 °C
Scan Begin	50 m/z	Set End Plate Offset	-500 V	Set Dry Gas	8.0 l/min
Scan End	3000 m/z	Set Charging Voltage	2000 V	Set Divert Valve	Waste
		Set Corona	0 nA	Set APCI Heater	0 °C

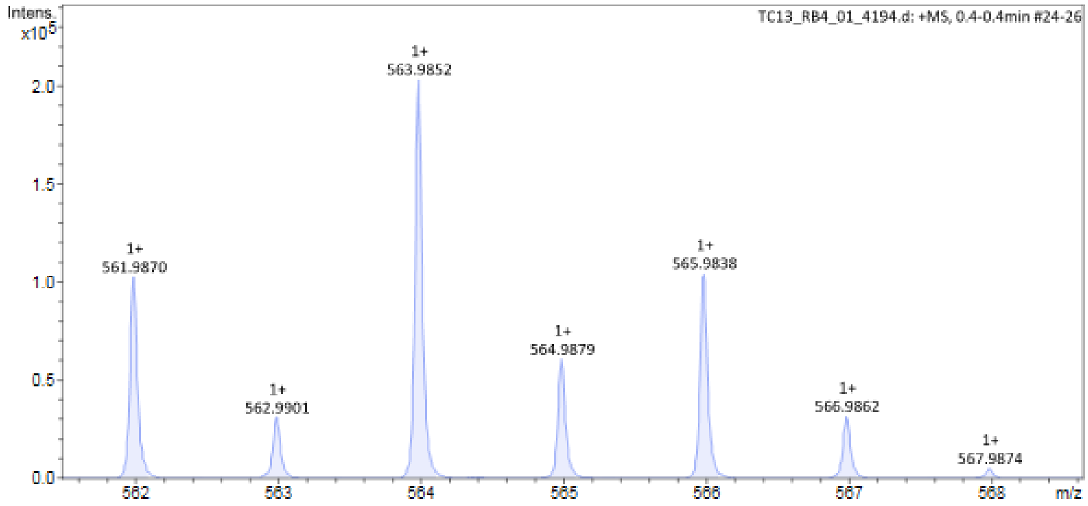

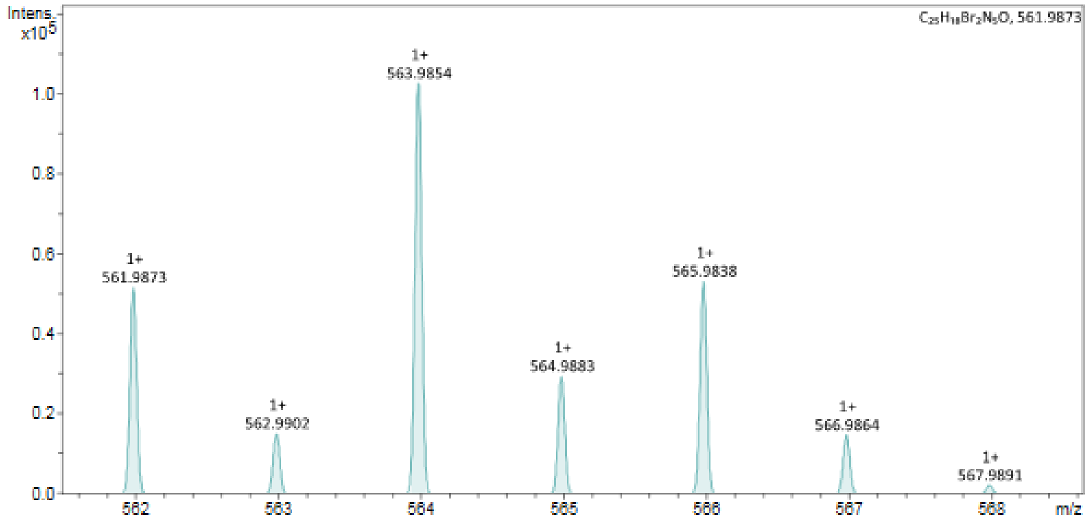

[illegible]

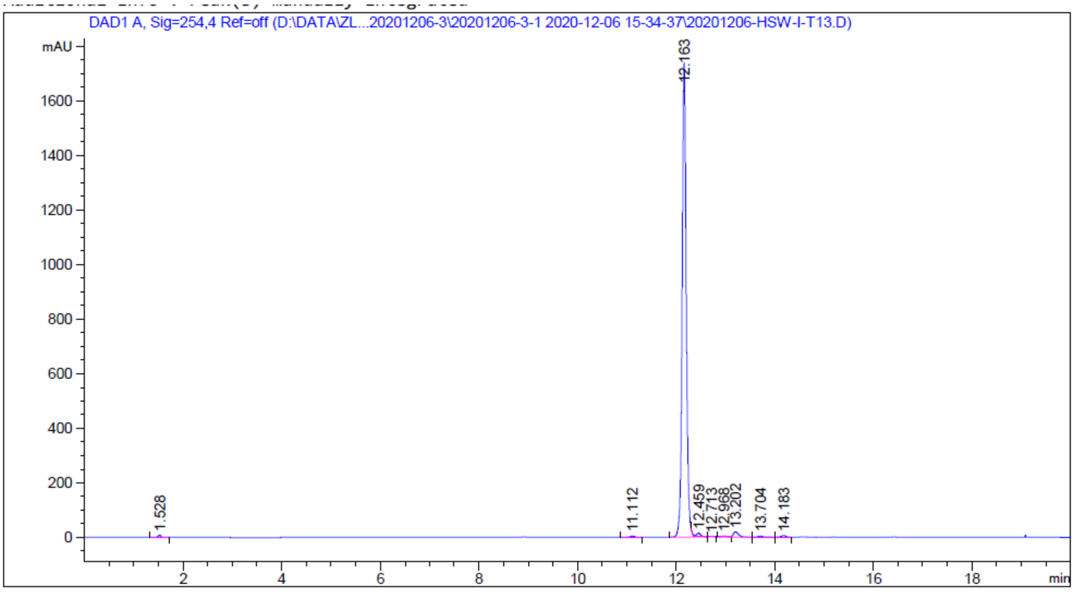

Signal 1: DAD1 A, Sig=254,4 Ref=off

Peak #	RetTime [min]	Type	Width [min]	Area [mAU*s]	Height [mAU]	Area %
1	1.528	BB	0.0687	35.10431	7.92701	0.3385
2	11.112	BB	0.0937	30.44456	4.43103	0.2935
3	12.163	BV R	0.0869	9969.26953	1739.82629	96.1186

1260R 12/6/2020 7:24:47 PM BY

Data File D:\DATA\ZL...TA\20201206-3\20201206-3-1 2020-12-06 15-34-37\[illegible]
Sample Name: 20201206-HSW-I-T13

Peak #	RetTime [min]	Type	Width [min]	Area [mAU*s]	Height [mAU]	Area %
4	12.459	VB E	0.0945	93.65110	14.37158	0.9029
5	12.713	BB	0.0795	9.61318	1.88434	0.0927
6	12.968	BV E	0.0889	22.53334	3.78806	0.2173
7	13.202	VB R	0.1072	141.75975	19.30497	1.3668
8	13.704	BB	0.1165	29.91514	3.67547	0.2884
9	14.183	BB	0.0887	39.55201	6.61921	0.3813

Totals : 1.03718e4 1801.82795

^{1}H NMR, ^{13}C NMR, HRMS, and HPLC of compound A14

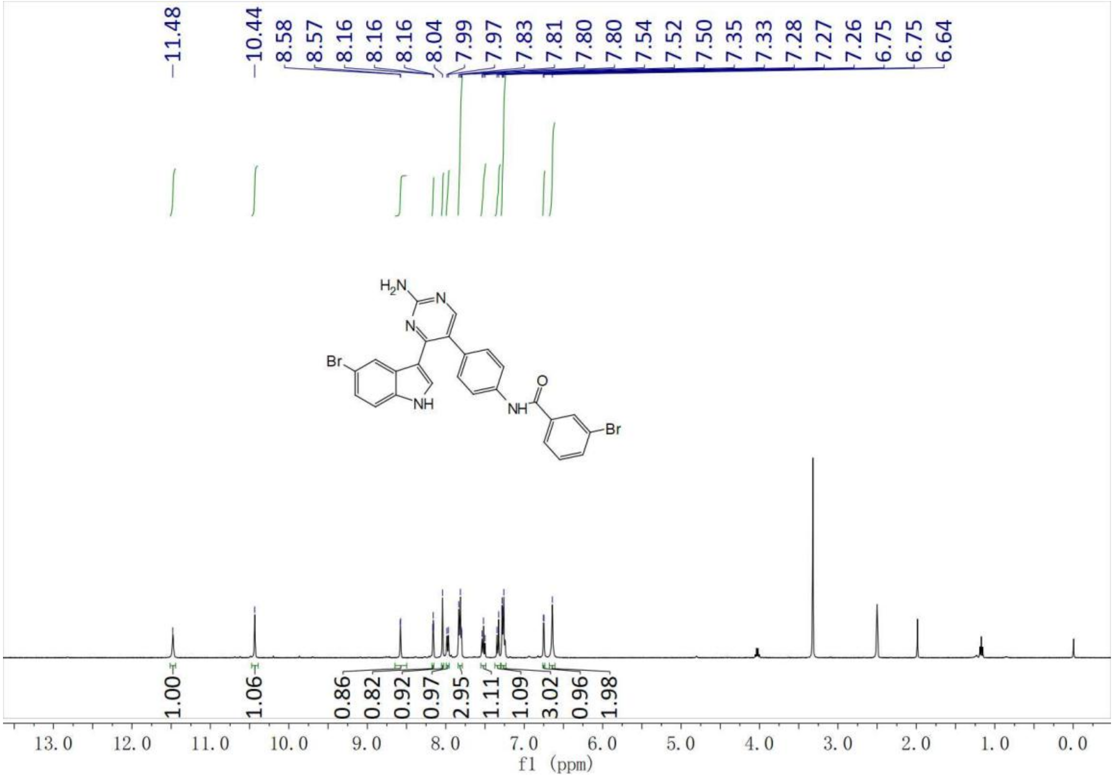

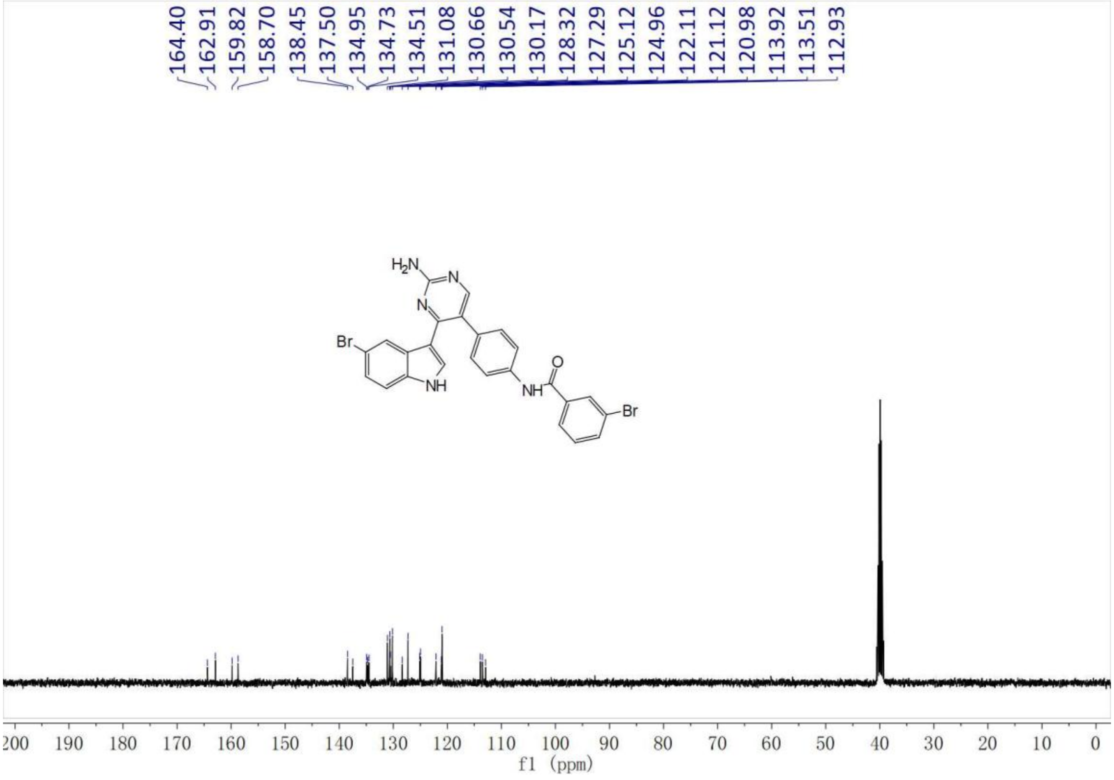

Display Report

Analysis Info

Analysis Name	D:\Data\data\2020\TC14_RB5_01_4195.d	Acquisition Date	10/10/2020 15:40:23 PM
Method	MS-2MIN-POS.m	Operator	BDAL@DE
Sample Name	TC14	Instrument	compact 8255754.20127
Comment			

Acquisition Parameter

Source Type	ESI	Ion Polarity	Positive	Set Nebulizer	2.0 Bar
Focus	Active	Set Capillary	4500 V	Set Dry Heater	200 °C
Scan Begin	50 m/z	Set End Plate Offset	-500 V	Set Dry Gas	8.0 l/min
Scan End	3000 m/z	Set Charging Voltage	2000 V	Set Divert Valve	Waste
		Set Corona	0 nA	Set APCI Heater	0 °C

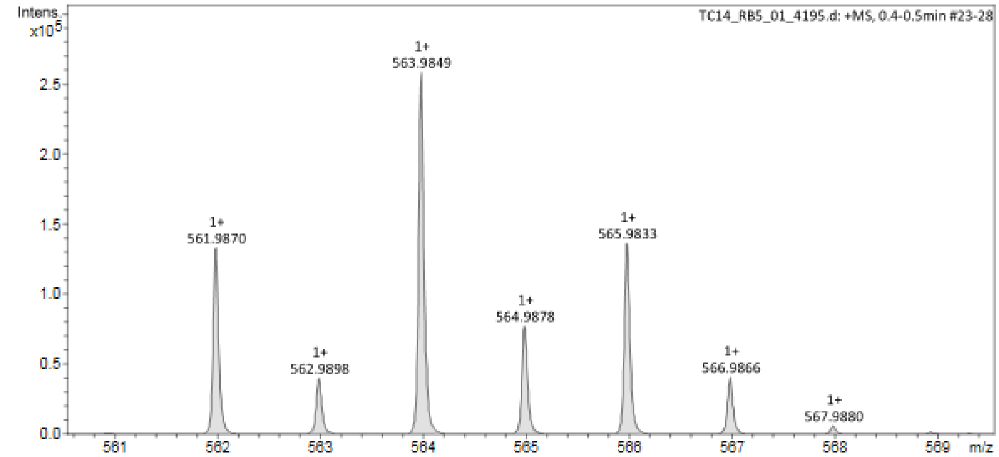

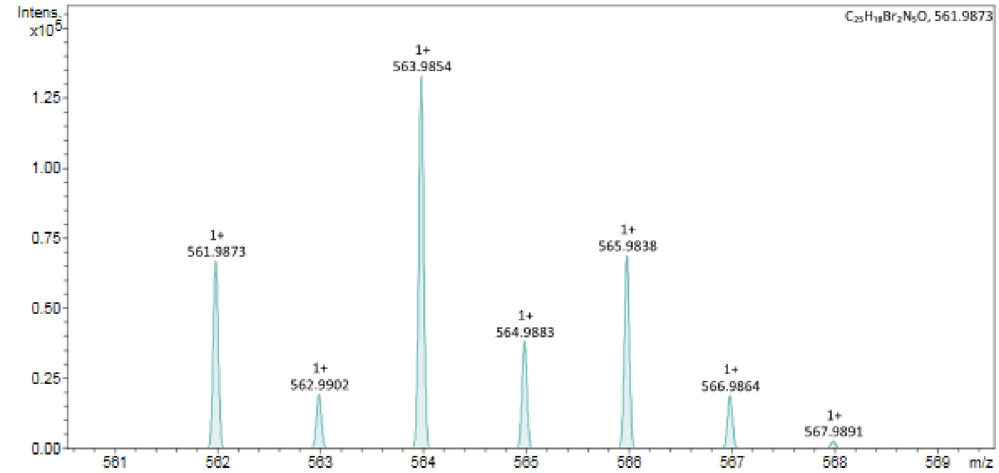

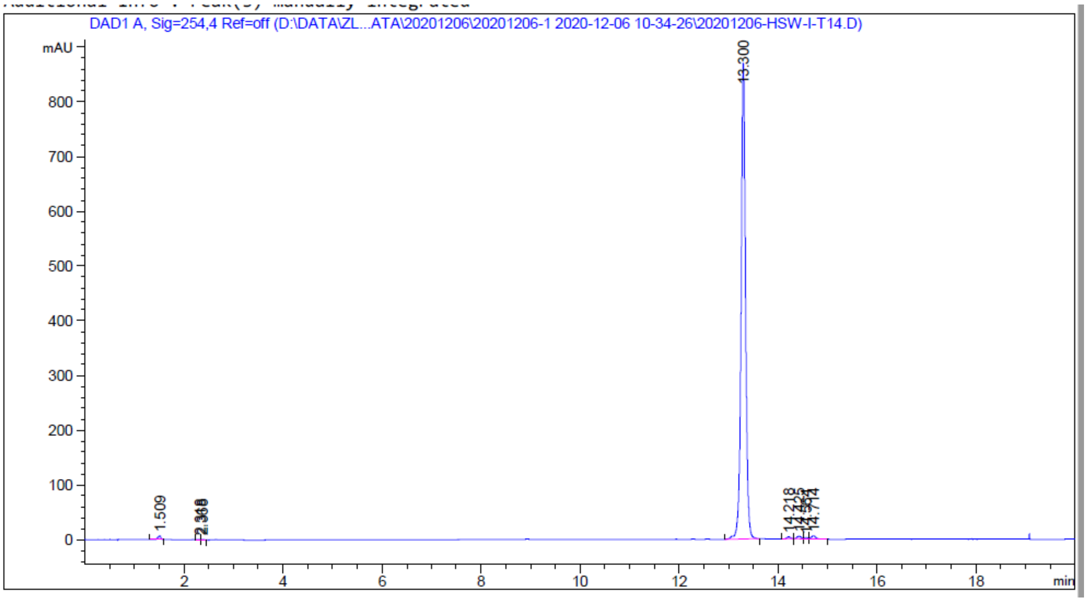

Signal 1: DAD1 A, Sig=254,4 Ref=off

Peak #	RetTime [min]	Type	Width [min]	Area [mAU*s]	Height [mAU]	Area %
1	1.509	BB	0.0662	28.65543	6.79342	0.5172
2	2.318	VV	0.0596	5.58147	1.13965	0.1007
3	2.368	VV	0.0597	5.32699	1.12557	0.0961

1260R 12/6/2020 2:37:55 PM BY

Data File D:\DATA\ZLM\DATA\20201206\20201206-1 2020-12-06 10-34-26\20201206-HSW-

Sample Name: 20201206-HSW-I-T14

Peak #	RetTime [min]	Type	Width [min]	Area [mAU*s]	Height [mAU]	Area %
4	13.300	BB	0.0931	5404.95264	868.47980	97.5444
5	14.218	BB	0.0721	17.60998	3.47581	0.3178
6	14.425	BV	0.0859	27.26084	4.64977	0.4920
7	14.564	VV	0.0775	14.40650	2.50826	0.2600
8	14.714	VB	0.0905	37.22372	5.94690	0.6718
Totals :				5541.01757	894.11918	

^{1}H NMR, ^{13}C NMR, HRMS, and HPLC of compound A15

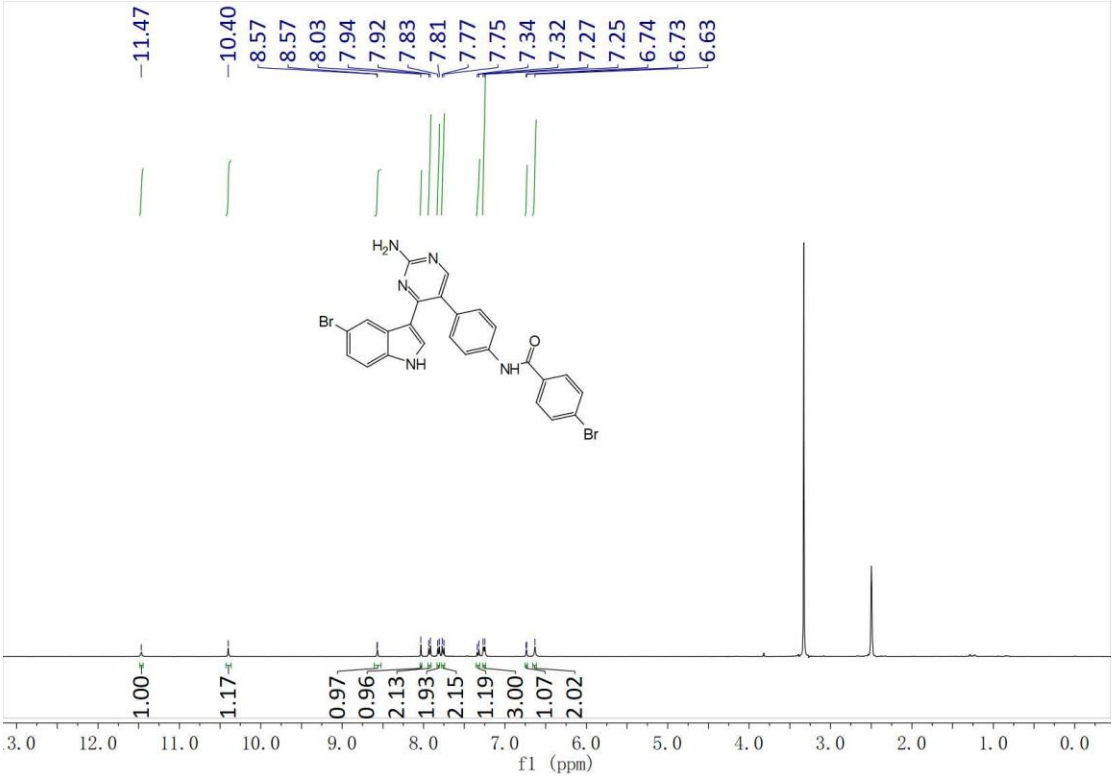

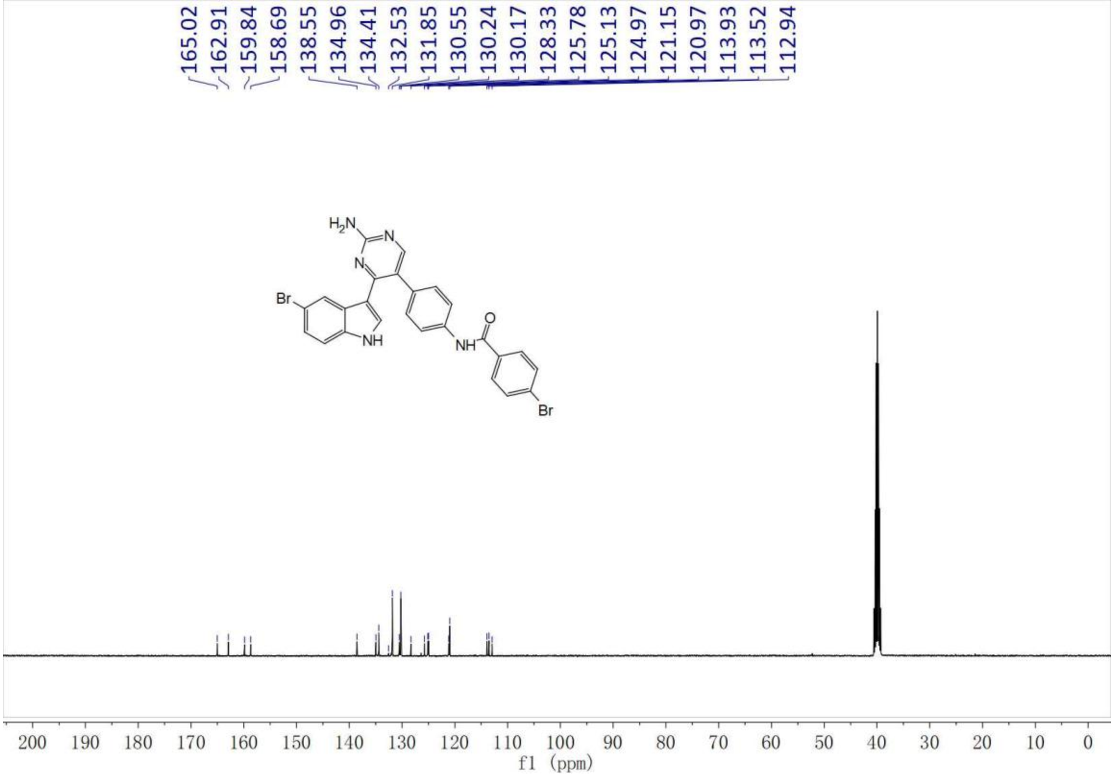

Display Report

Analysis Info

Analysis Name	D:\Data\data\2020\TC5_RA4_01_4186.d	Acquisition Date	10/10/2020 15:15:31 PM
Method	MS-2MIN-POS.m	Operator	BDAL@DE
Sample Name	TC5	Instrument	compact 8255754.20127
Comment			

Acquisition Parameter

Source Type	ESI	Ion Polarity	Positive	Set Nebulizer	2.0 Bar
Focus	Active	Set Capillary	4500 V	Set Dry Heater	200 °C
Scan Begin	50 m/z	Set End Plate Offset	-500 V	Set Dry Gas	8.0 l/min
Scan End	3000 m/z	Set Charging Voltage	2000 V	Set Divert Valve	Waste
		Set Corona	0 nA	Set APCI Heater	0 °C

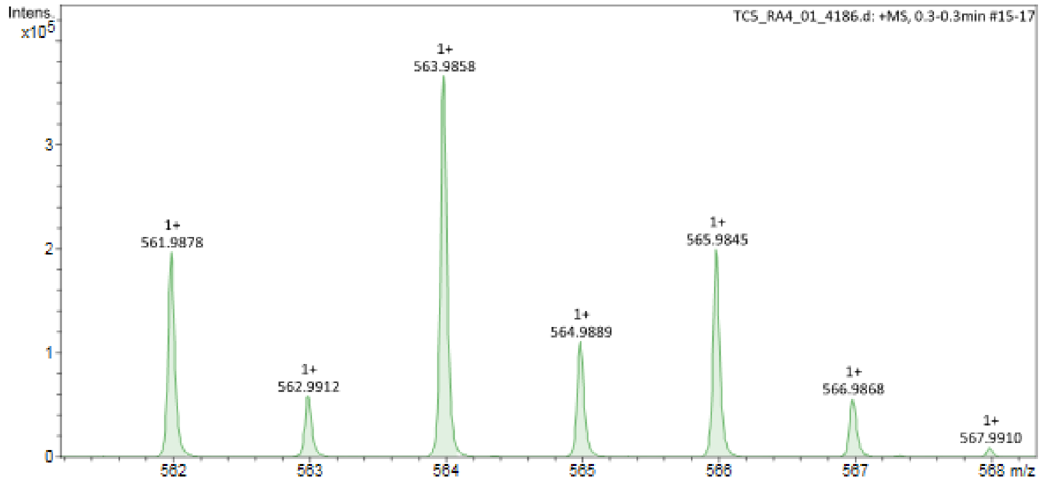

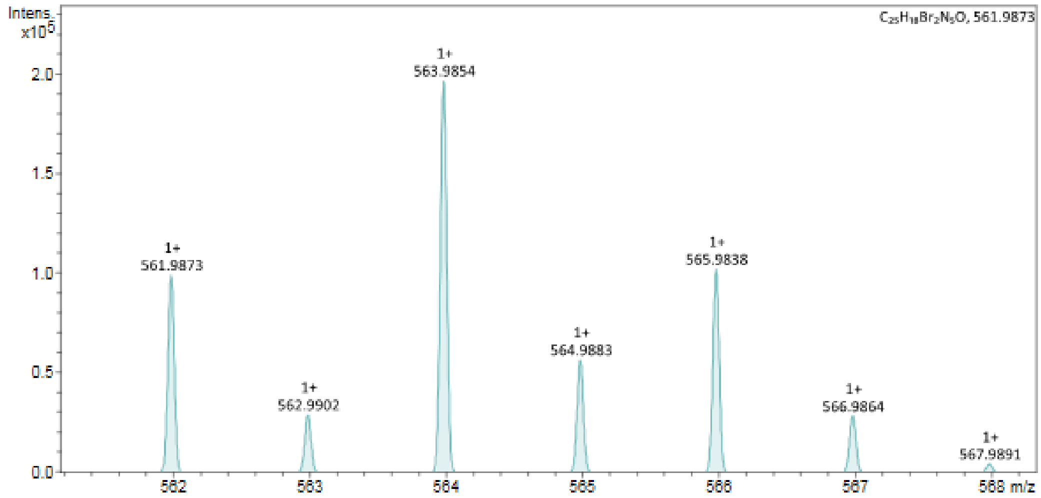

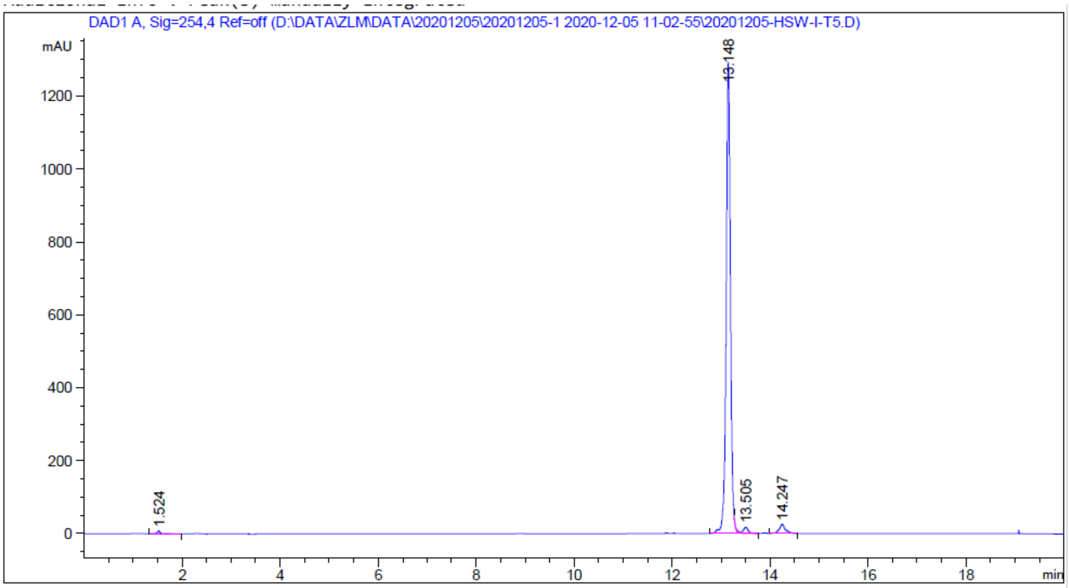

Signal 1: DAD1 A, Sig=254,4 Ref=off

Peak #	RetTime [min]	Type	Width [min]	Area [mAU*s]	Height [mAU]	Area %
1	1.524	BB	0.0724	39.39222	7.54857	0.4801
2	13.148	VV R	0.0903	7814.19287	1292.43201	95.2371
3	13.505	VB E	0.1176	137.57645	16.36361	1.6767

1260R 12/5/2020 1:45:08 PM BY

Data File D:\DATA\ZLM\DATA\20201205\20201205-1 2020-12-05 11-02-55\20201205-HSW-I-T
Sample Name: 20201205-HSW-I-T5

Peak #	RetTime [min]	Type	Width [min]	Area [mAU*s]	Height [mAU]	Area %
4	14.247	BB	0.1191	213.82581	25.04178	2.6060
Totals :				8204.98734	1341.38597	

^{1}H NMR, ^{13}C NMR, HRMS, and HPLC of compound A16

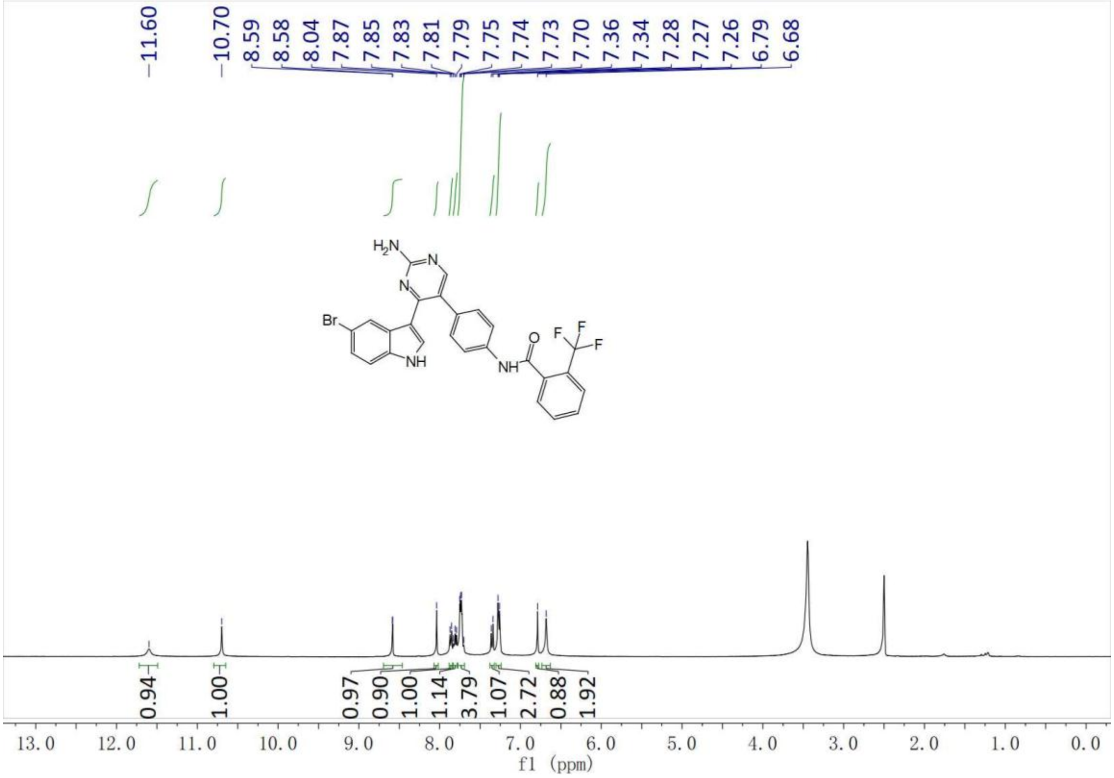

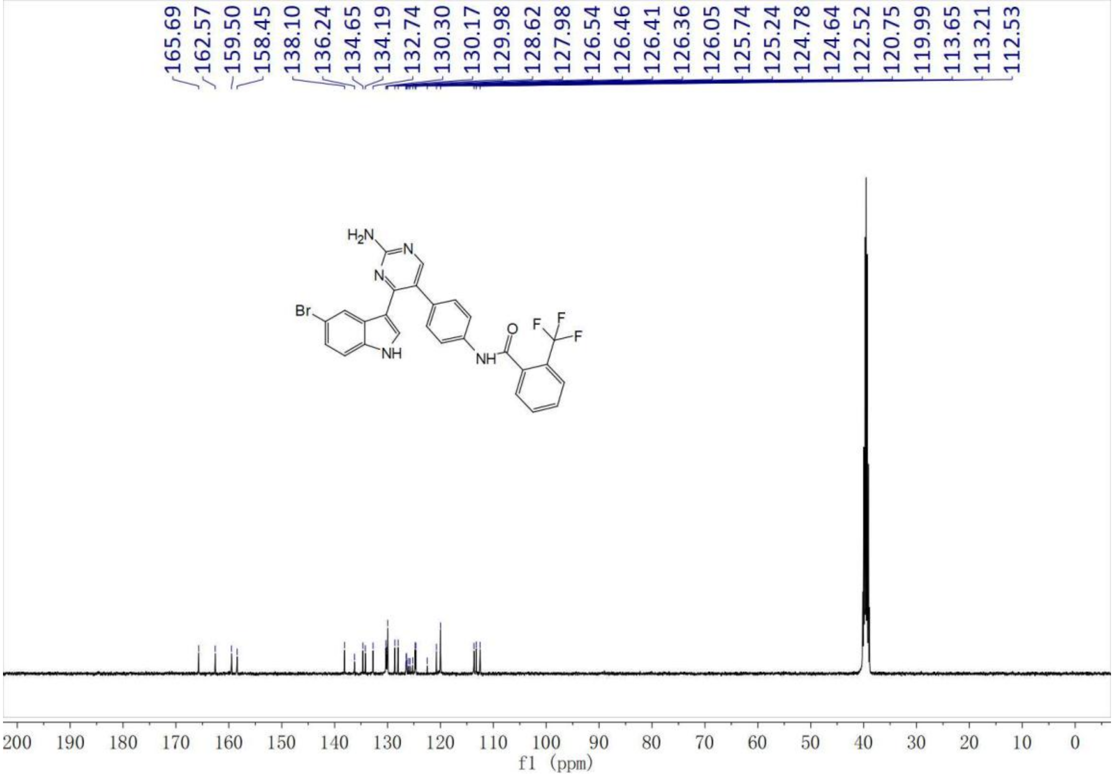

Display Report

**Analysis Info**		Acquisition Date	10/10/2020 16:16:18 PM
Analysis Name	D:\Data\data\2020\TC34_RD3_01_4208.d		
Method	MS-2MIN-POS.m	Operator	BDAL@DE
Sample Name	TC34	Instrument	compact 8255754.20127
Comment			

Acquisition Parameter

Source Type	ESI	Ion Polarity	Positive	Set Nebulizer	2.0 Bar
Focus	Active	Set Capillary	4500 V	Set Dry Heater	200 °C
Scan Begin	50 m/z	Set End Plate Offset	-500 V	Set Dry Gas	8.0 l/min
Scan End	3000 m/z	Set Charging Voltage	2000 V	Set Divert Valve	Waste
		Set Corona	0 nA	Set APCI Heater	0 °C

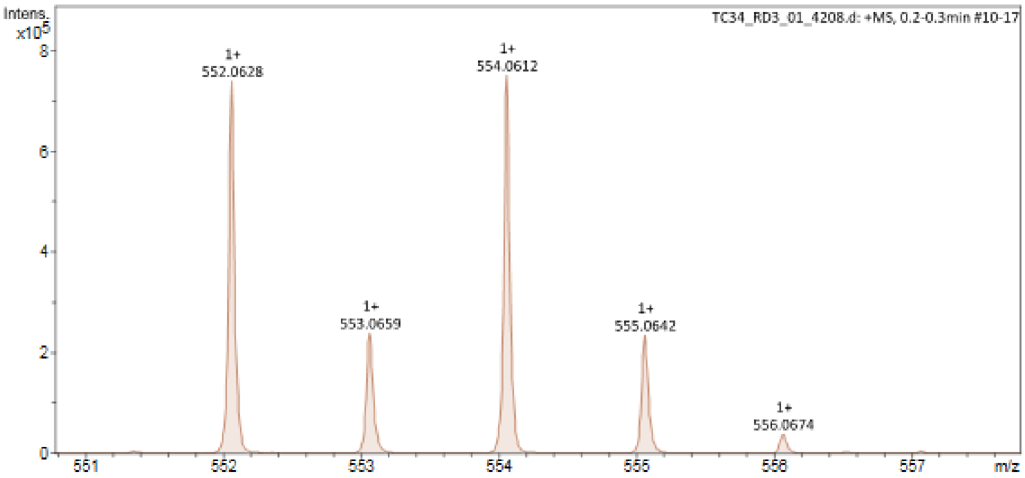

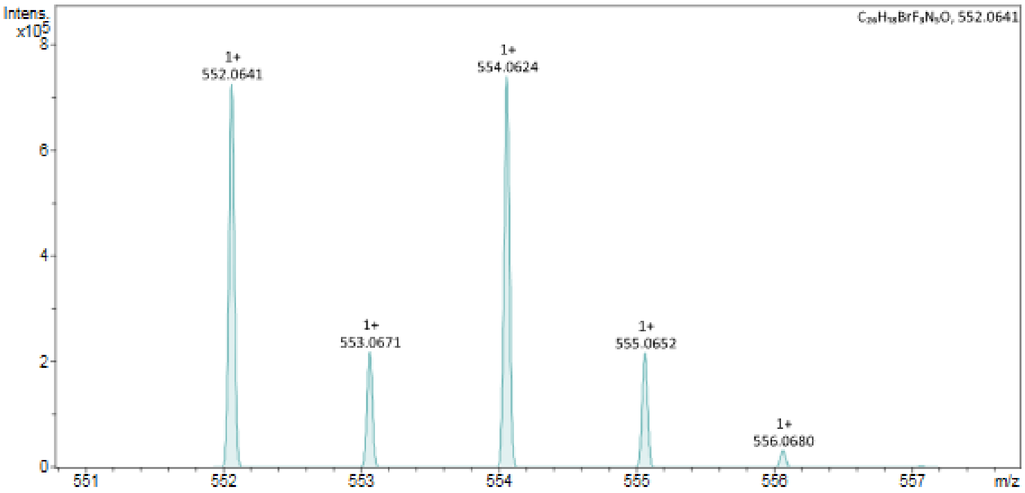

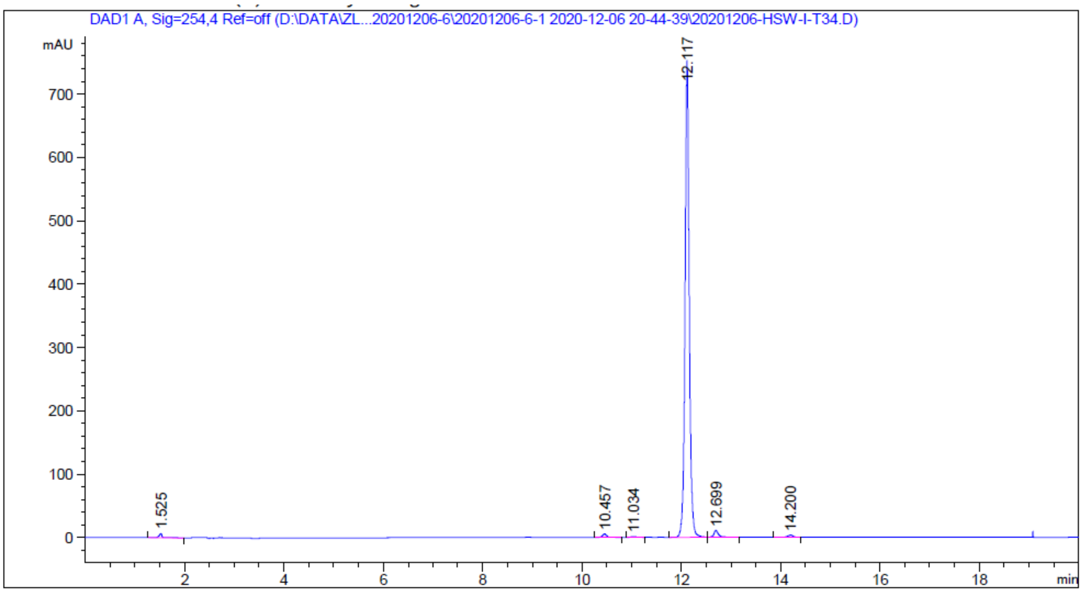

Signal 1: DAD1 A, Sig=254,4 Ref=off

Peak #	RetTime [min]	Type	Width [min]	Area [mAU*s]	Height [mAU]	Area %
1	1.525	VV R	0.0708	31.88255	6.48621	0.6912
2	10.457	BV R	0.0894	36.28636	5.68941	0.7866
3	11.034	BV	0.1054	12.17280	1.38676	0.2639
4	12.117	VV R	0.0877	4431.74023	752.79211	96.0749

1260R 12/7/2020 8:29:57 AM BY

Data File D:\DATA\ZL...TA\20201206-6\20201206-6-1 2020-12-06 20-44-39\20201206-H
Sample Name: 20201206-HSW-I-T34

Peak #	RetTime [min]	Type	Width [min]	Area [mAU*s]	Height [mAU]	Area %
5	12.699	VV R	0.0969	73.27375	10.83844	1.5885
6	14.200	VB R	0.1038	27.44322	3.64793	0.5949

Totals : 4612.79891 780.84087

^{1}H NMR, ^{13}C NMR, HRMS, and HPLC of compound A17

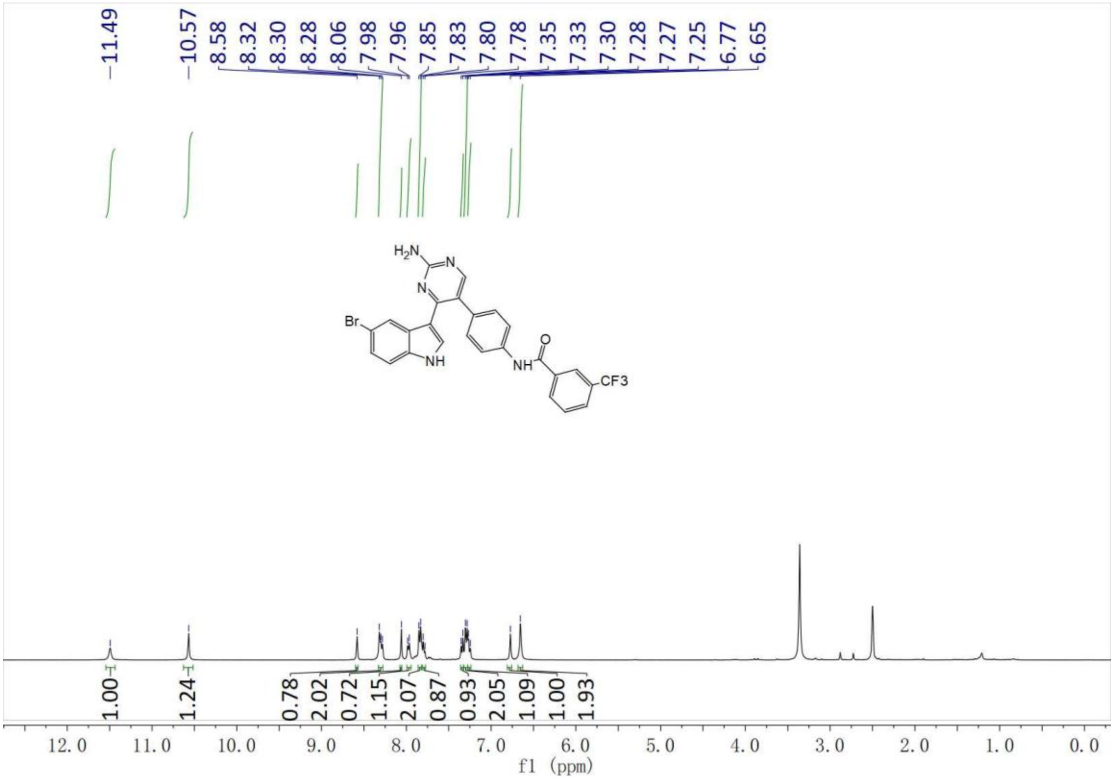

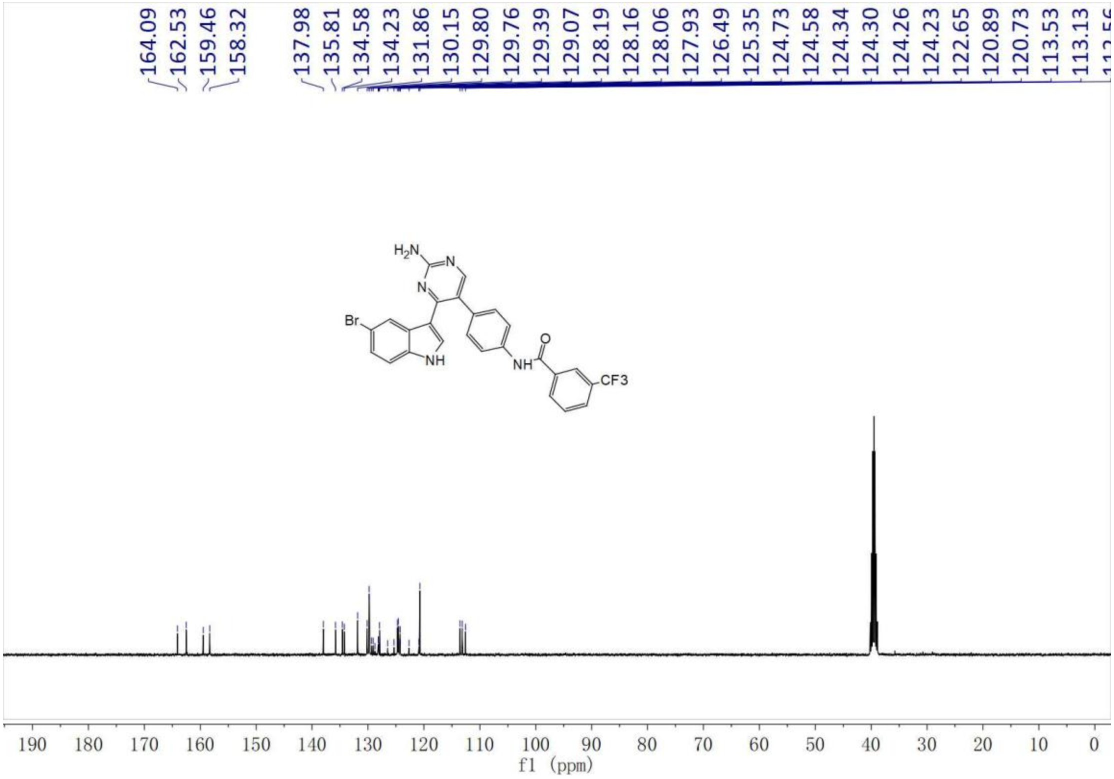

Display Report

Analysis Info

Analysis Name	D:\Data\data\2020\TC29_RC7_01_4204.d	Acquisition Date	10/10/2020 16:05:15 PM
Method	MS-2MIN-POS.m	Operator	BDAL@DE
Sample Name	TC29	Instrument	compact 8255754.20127
Comment			

Acquisition Parameter

Source Type	ESI	Ion Polarity	Positive	Set Nebulizer	2.0 Bar
Focus	Active	Set Capillary	4500 V	Set Dry Heater	200 °C
Scan Begin	50 m/z	Set End Plate Offset	-500 V	Set Dry Gas	8.0 l/min
Scan End	3000 m/z	Set Charging Voltage	2000 V	Set Divert Valve	Waste
		Set Corona	0 nA	Set APCI Heater	0 °C

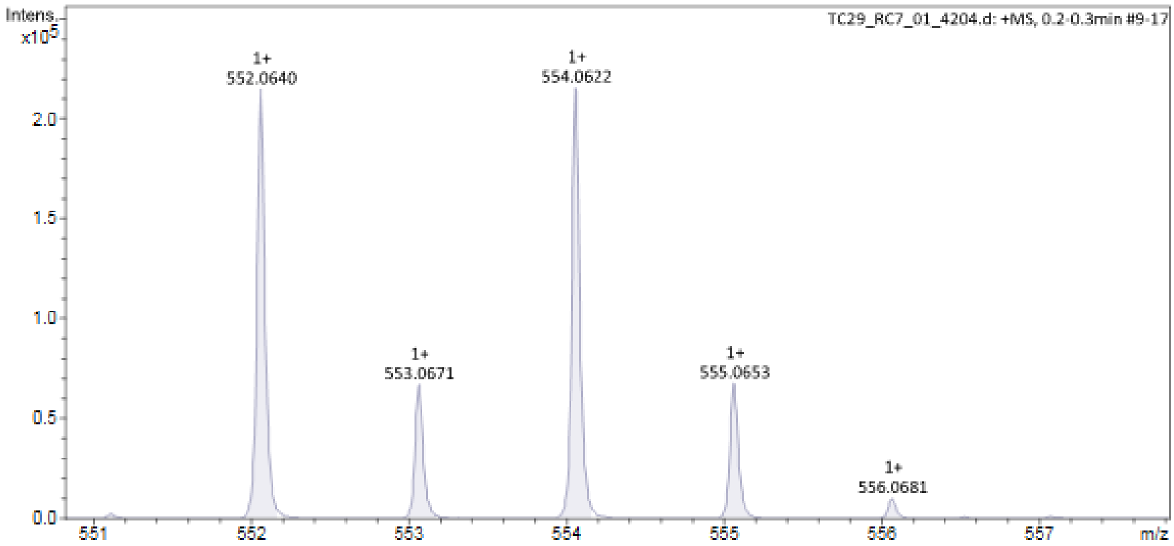

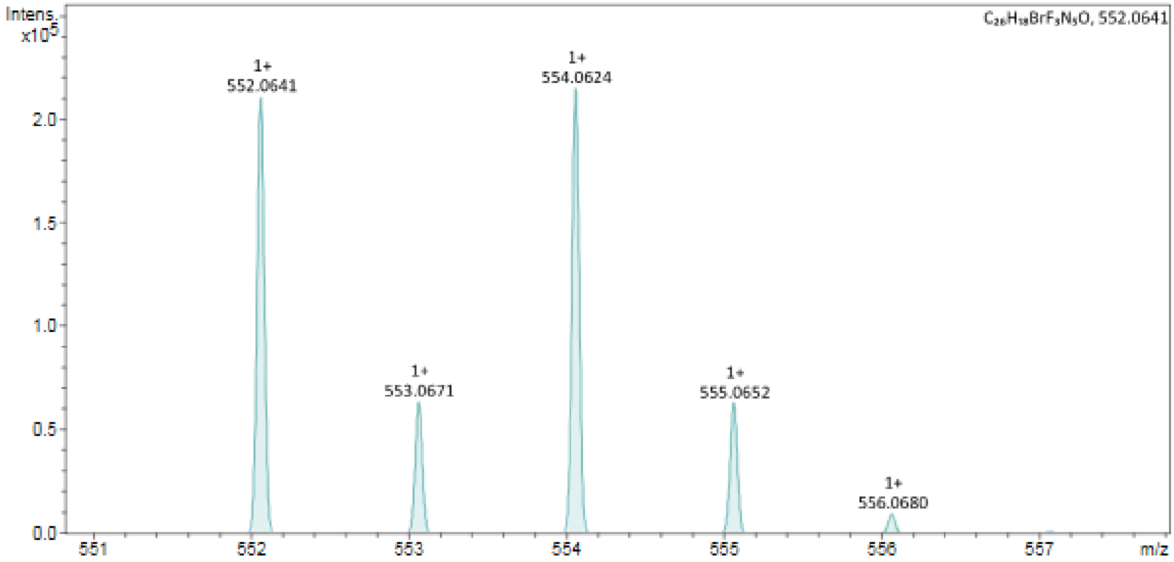

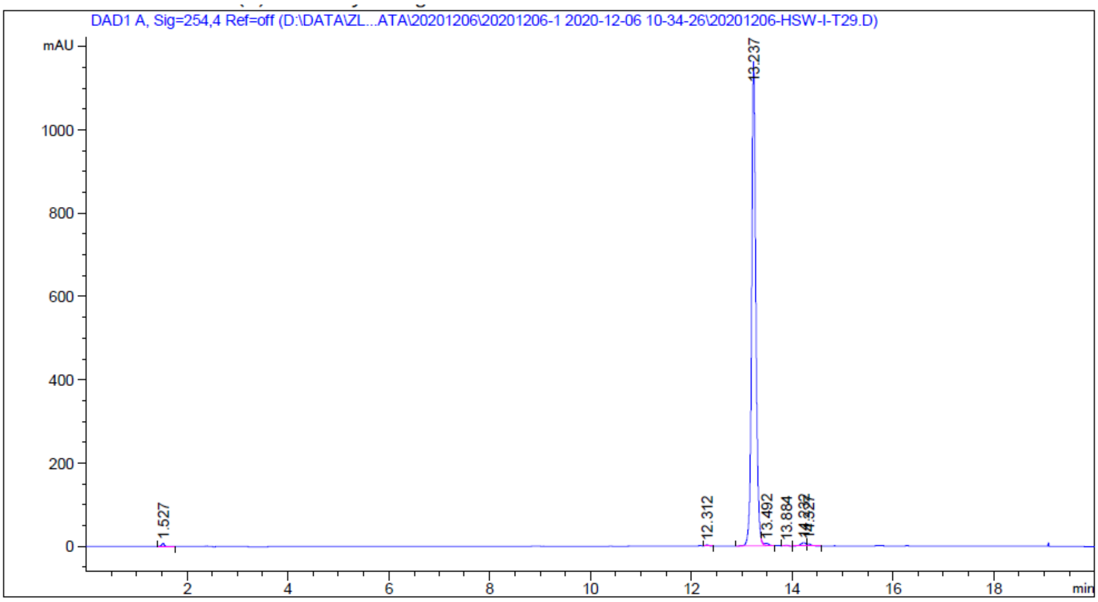

Signal 1: DAD1 A, Sig=254,4 Ref=off

Peak #	RetTime [min]	Type	Width [min]	Area [mAU*s]	Height [mAU]	Area %
1	1.527	BB	0.0572	25.81744	6.96871	0.3712
2	12.312	VV	0.0806	17.04130	3.05224	0.2450
3	13.237	BV R	0.0875	6781.24902	1163.45325	97.4878

1260R 12/6/2020 7:35:21 PM BY

Data File D:\DATA\ZLM\DATA\20201206\20201206-1 2020-12-06 10-34-26\2020120
Sample Name: 20201206-HSW-I-T29

Peak #	RetTime [min]	Type	Width [min]	Area [mAU*s]	Height [mAU]	Area %
4	13.492	VB E	0.0891	37.46646	5.78184	0.5386
5	13.884	BB	0.0726	7.65210	1.51114	0.1100
6	14.232	BV	0.1043	59.66588	8.07040	0.8578
7	14.327	VB	0.0770	27.10612	4.97653	0.3897

Totals : 6955.99833 1193.81410

^{1}H NMR, ^{13}C NMR, HRMS, and HPLC of compound A18

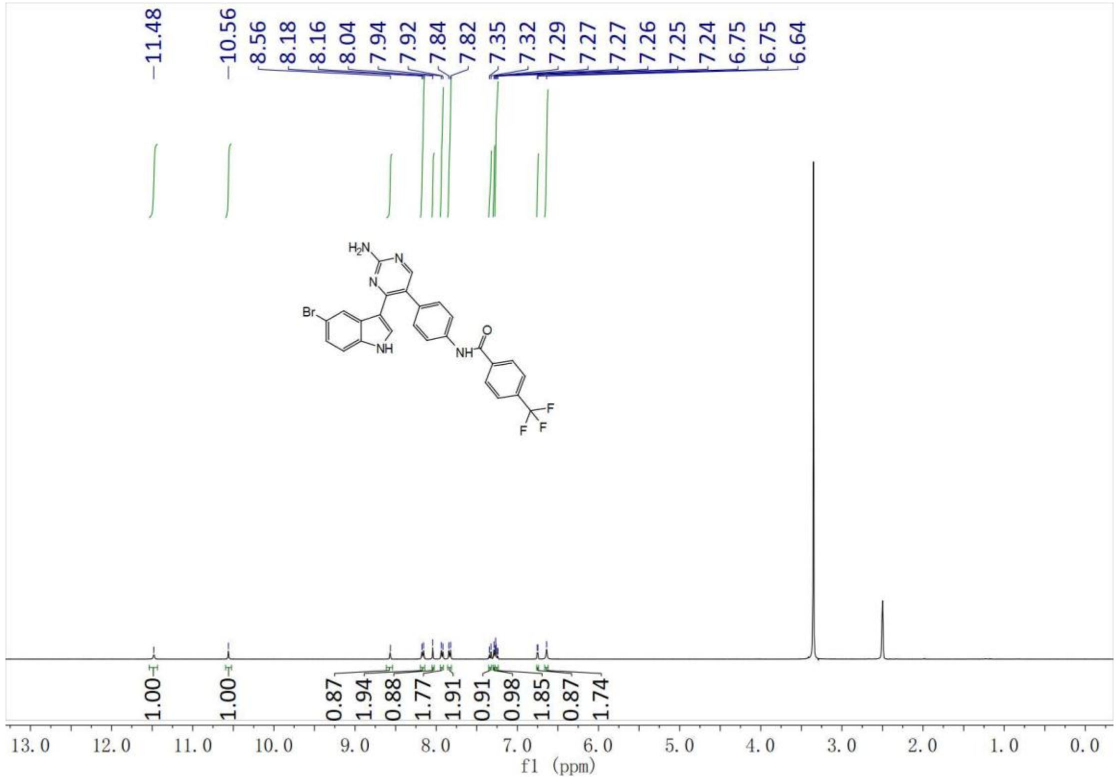

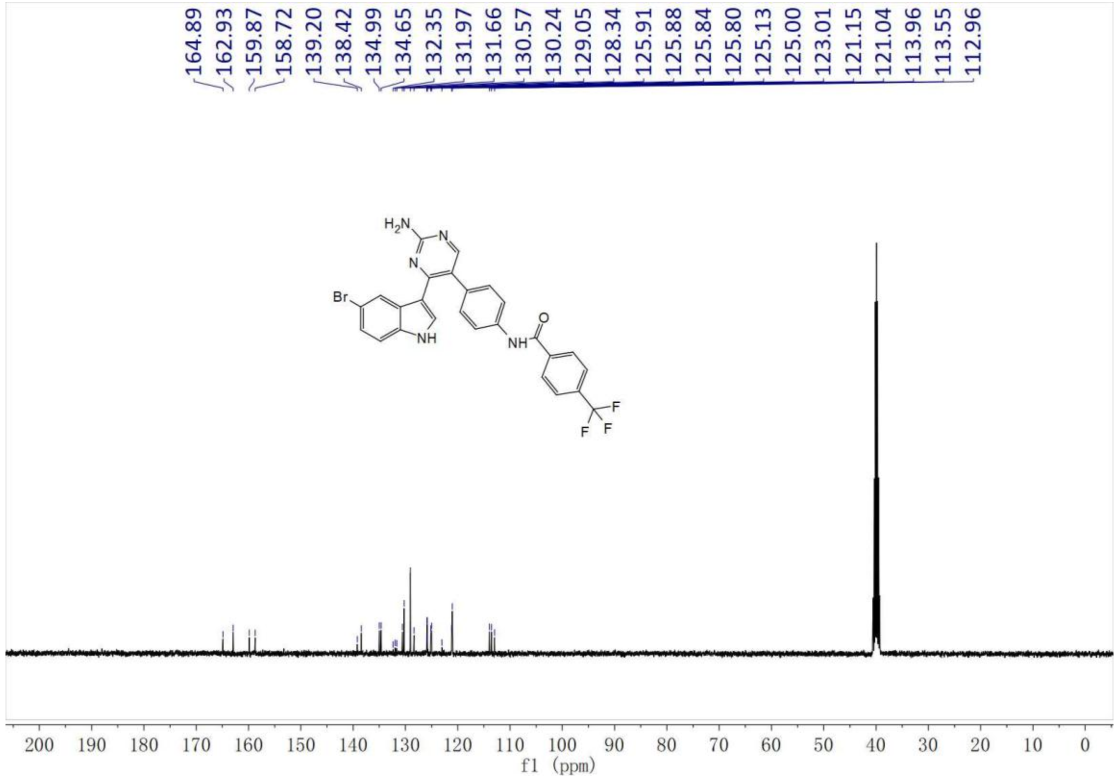

Display Report

Analysis Info

Analysis Name	D:\Data\data\2020\TC8_RA7_01_4189.d	Acquisition Date	10/10/2020 15:23:49 PM
Method	MS-2MIN-POS.m	Operator	BDAL@DE
Sample Name	TC8	Instrument	compact 8255754.20127
Comment			

Acquisition Parameter

Source Type	ESI	Ion Polarity	Positive	Set Nebulizer	2.0 Bar
Focus	Active	Set Capillary	4500 V	Set Dry Heater	200 °C
Scan Begin	50 m/z	Set End Plate Offset	-500 V	Set Dry Gas	8.0 l/min
Scan End	3000 m/z	Set Charging Voltage	2000 V	Set Divert Valve	Waste
		Set Corona	0 nA	Set APCI Heater	0 °C

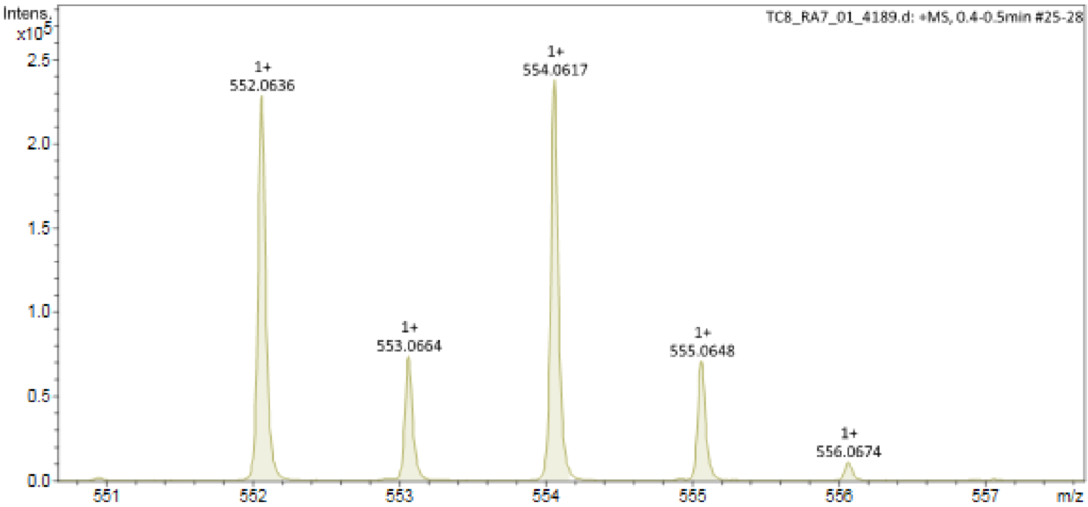

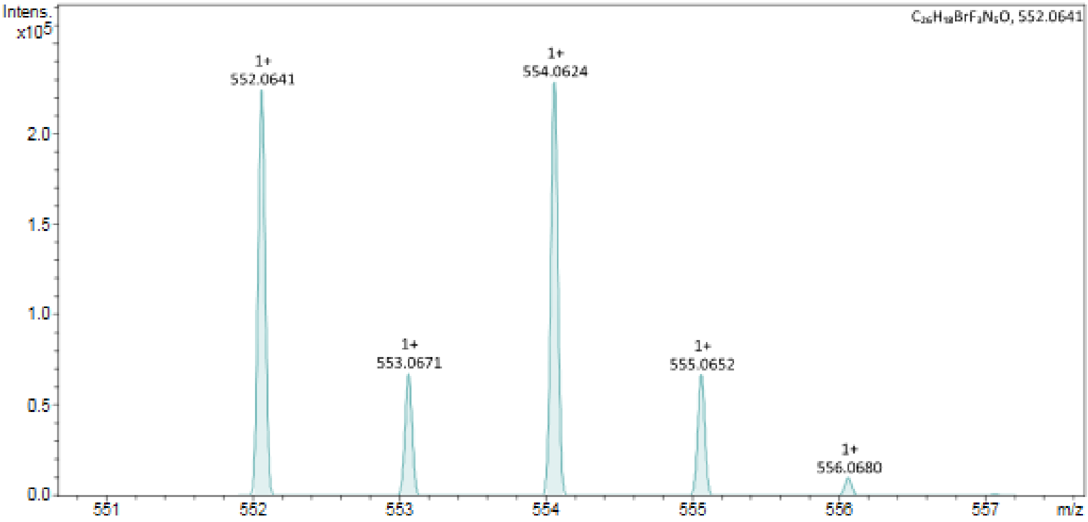

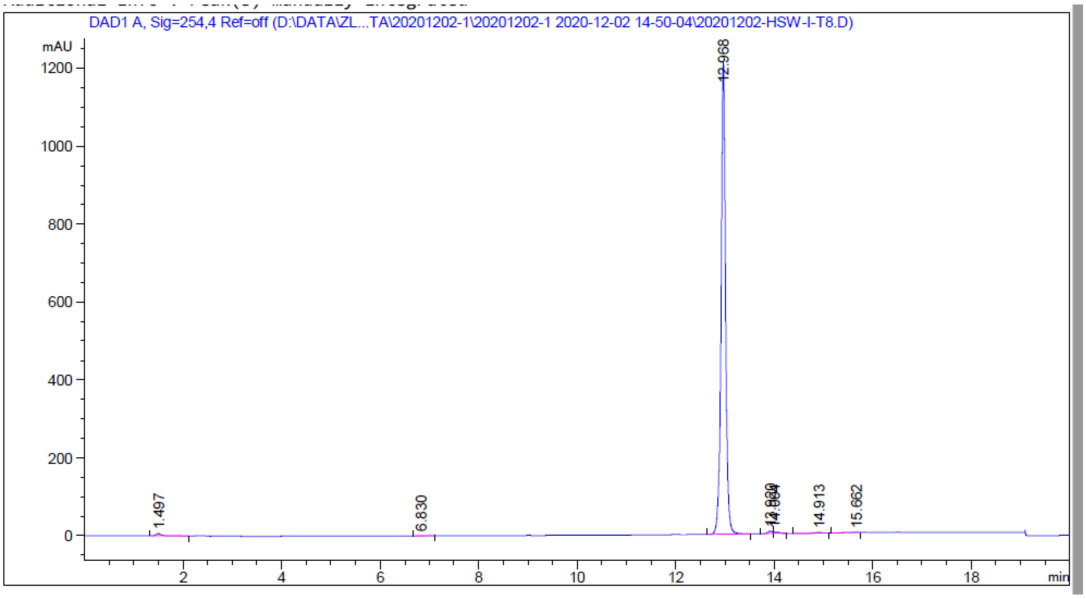

Signal 1: DAD1 A, Sig=254,4 Ref=off

Peak #	RetTime [min]	Type	Width [min]	Area [mAU*s]	Height [mAU]	Area %
1	1.497	BV R	0.0940	41.61324	5.99476	0.5319
2	6.830	BB	0.1173	12.54380	1.27524	0.1603
3	12.968	BB	0.0937	7660.33789	1212.56128	97.9128

1260R 12/6/2020 7:34:14 PM BY

Data File D:\DATA\ZLM\DATA\20201202-1\20201202-1 2020-12-02 14-50-04\
Sample Name: 20201202-HSW-I-T8

Peak #	RetTime [min]	Type	Width [min]	Area [mAU*s]	Height [mAU]	Area %
4	13.920	BV	0.0884	38.88698	6.22630	0.4970
5	14.004	VB	0.1017	37.70924	4.57296	0.4820
6	14.913	BB	0.1290	20.26473	1.87684	0.2590
7	15.662	BB	0.1213	12.27695	1.29282	0.1569

Totals : 7823.63282 1233.80020

^{1}H NMR, ^{13}C NMR, HRMS, and HPLC of compound A19

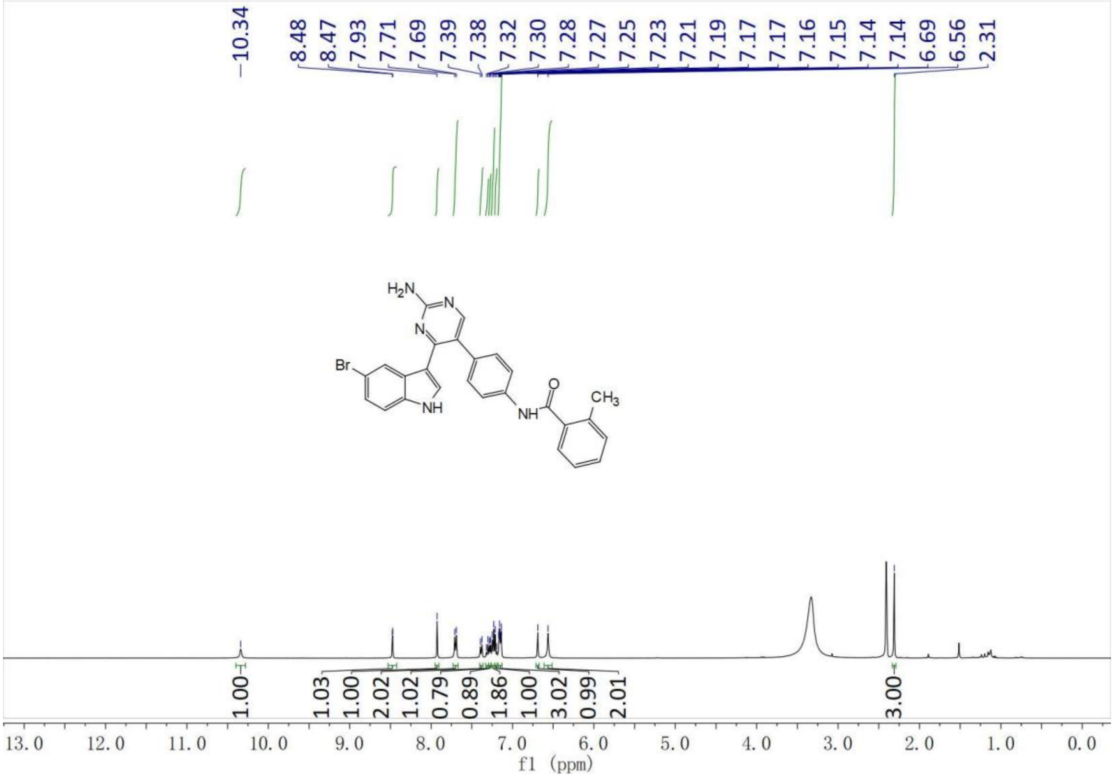

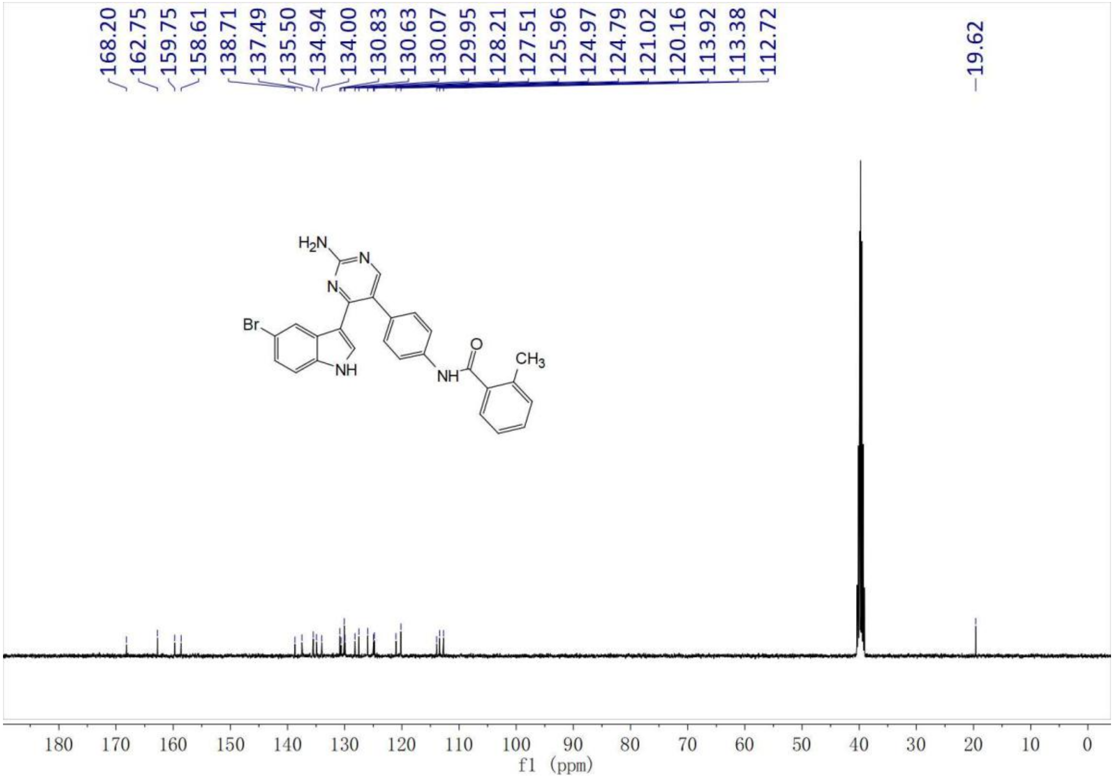

Display Report

Analysis Info

Analysis Name	D:\Data\data\2020\TC35_RD4_01_4209.d	Acquisition Date	10/10/2020 16:19:03 PM
Method	MS-2MIN-POS.m	Operator	BDAL@DE
Sample Name	TC35	Instrument	compact 8255754.20127
Comment			

Acquisition Parameter

Source Type	ESI	Ion Polarity	Positive	Set Nebulizer	2.0 Bar
Focus	Active	Set Capillary	4500 V	Set Dry Heater	200 °C
Scan Begin	50 m/z	Set End Plate Offset	-500 V	Set Dry Gas	8.0 l/min
Scan End	3000 m/z	Set Charging Voltage	2000 V	Set Divert Valve	Waste
		Set Corona	0 nA	Set APCI Heater	0 °C

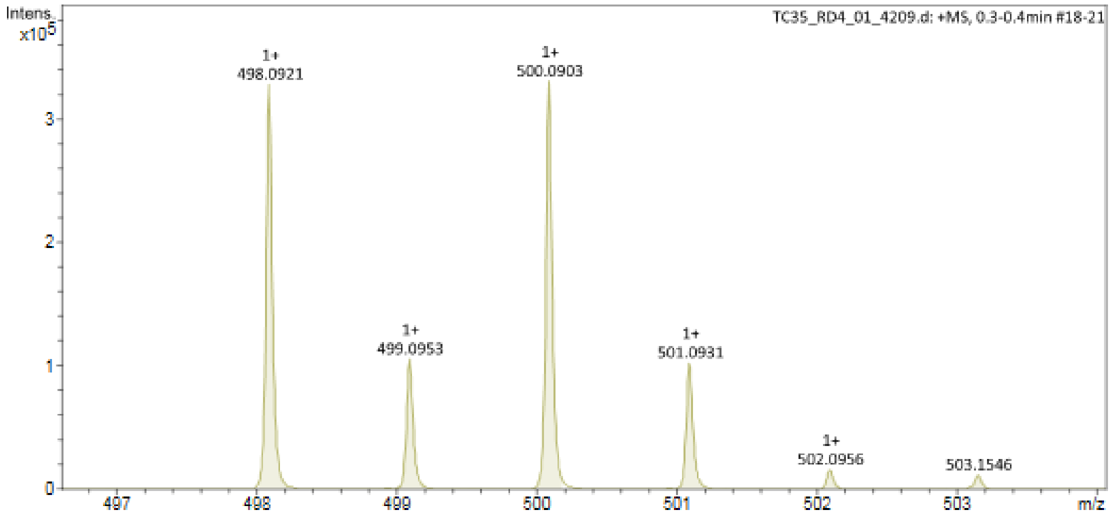

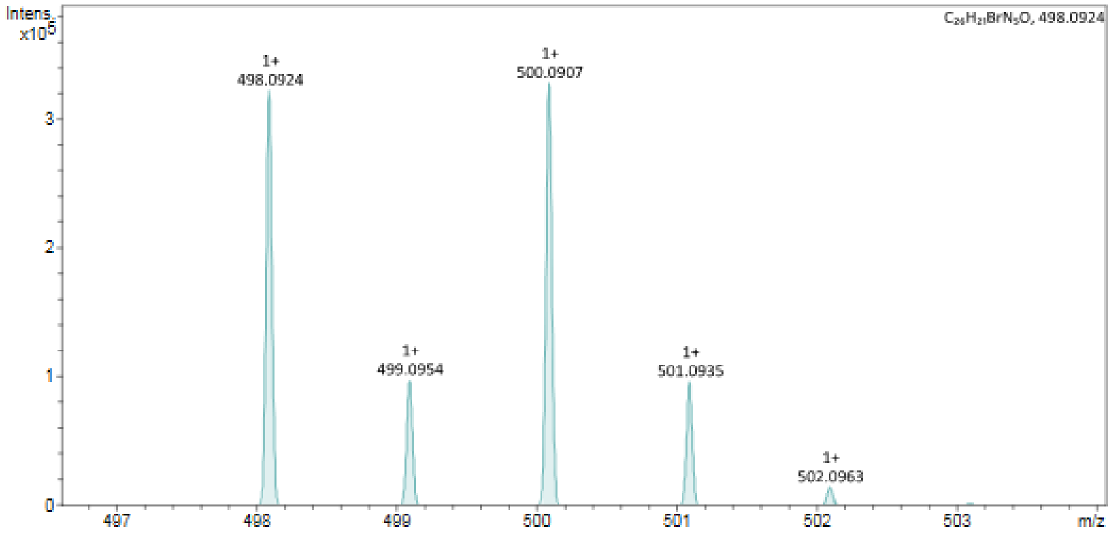

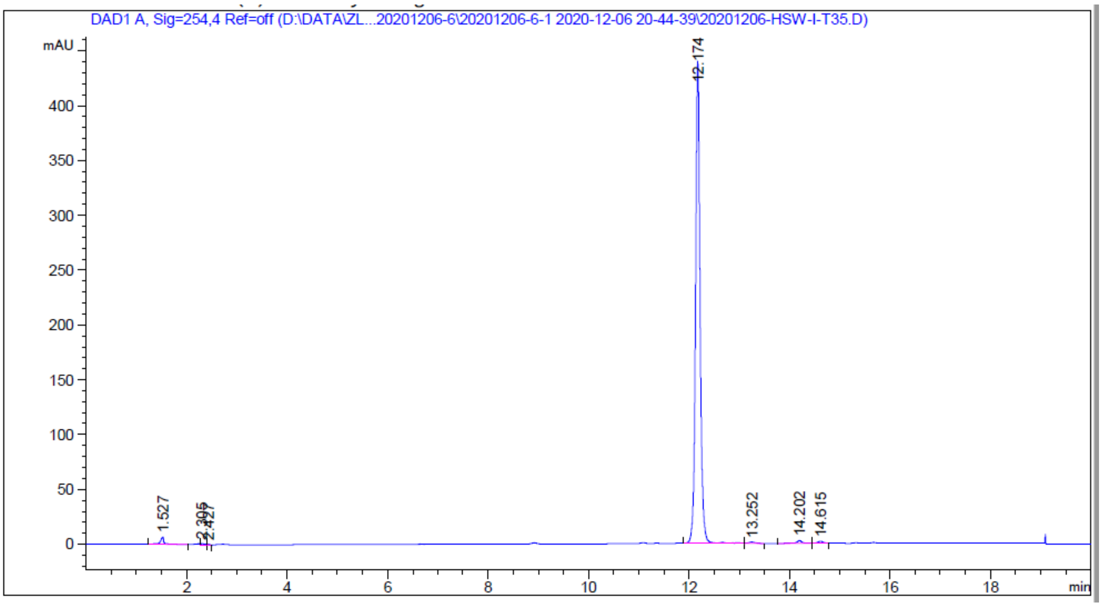

Signal 1: DAD1 A, Sig=254,4 Ref=off

Peak #	RetTime [min]	Type	Width [min]	Area [mAU*s]	Height [mAU]	Area %
1	1.527	VB R	0.0717	34.54093	6.74751	1.2206
2	2.305	VV	0.0755	7.14499	1.14134	0.2525
3	2.427	VB	0.0351	3.05350	1.09283	0.1079
4	12.174	BV R	0.0933	2743.27734	439.87961	96.9428

1260R 12/7/2020 8:31:09 AM BY

Data File D:\DATA\ZL...TA\20201206-6\20201206-6-1 2020-12-06 20-44-39\20
Sample Name: 20201206-HSW-I-T35

Peak #	RetTime [min]	Type	Width [min]	Area [mAU*s]	Height [mAU]	Area %
5	13.252	BB	0.0893	9.42099	1.26584	0.3329
6	14.202	BV R	0.0897	17.94266	2.59298	0.6341
7	14.615	VB	0.0917	14.40896	1.87388	0.5092

Totals : 2829.78938 454.59399

^{1}H NMR, ^{13}C NMR, HRMS, and HPLC of compound A20

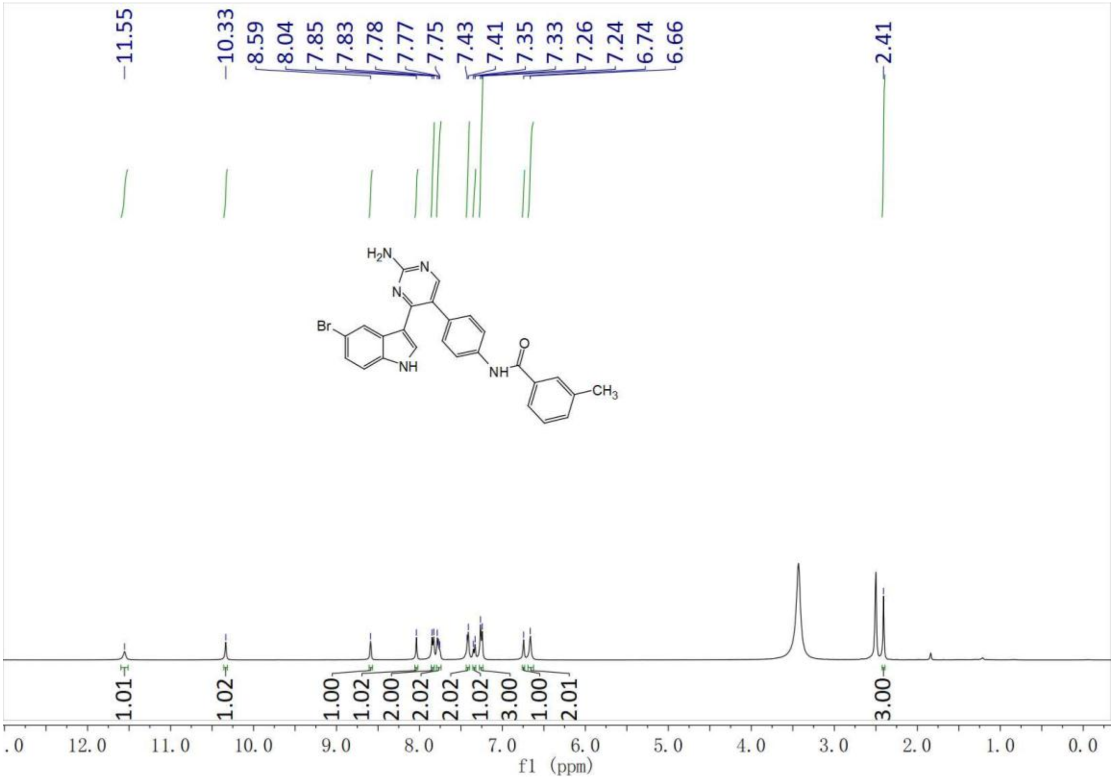

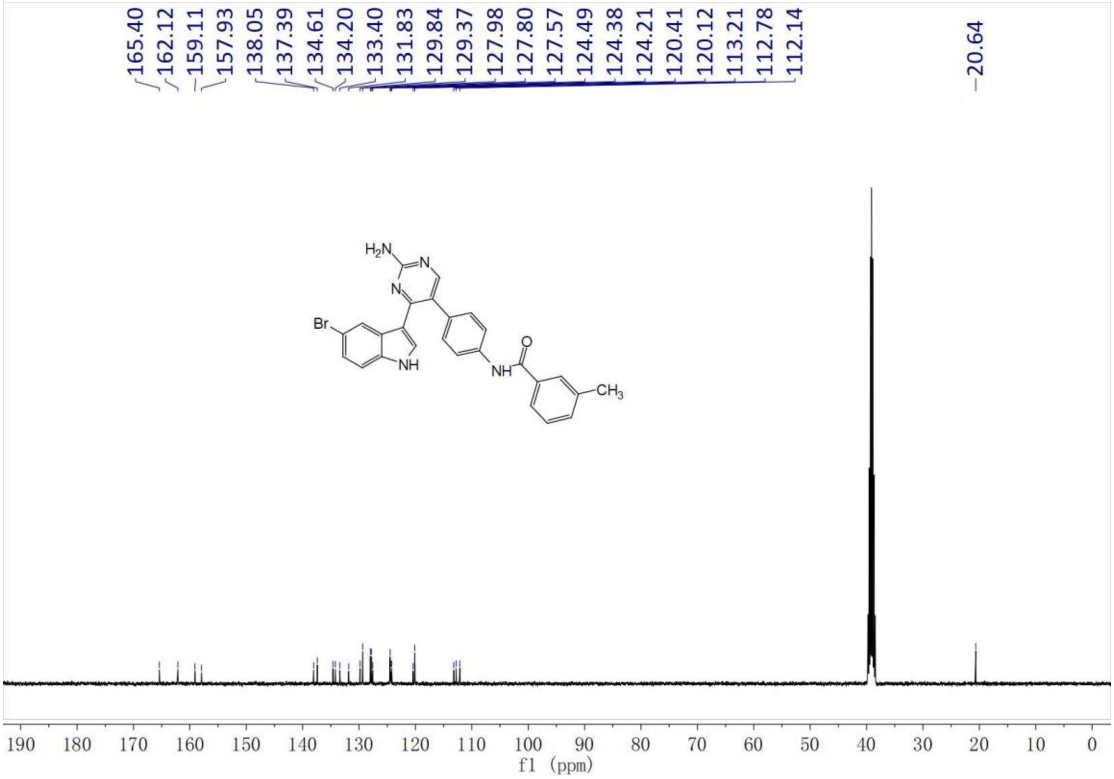

Display Report

Analysis Info

Analysis Name	D:\Data\data\2020\TC36_RD5_01_4210.d	Acquisition Date	10/10/2020 16:21:50 PM
Method	MS-2MIN-POS.m	Operator	BDAL@DE
Sample Name	TC36	Instrument	compact 8255754.20127
Comment			

Acquisition Parameter

Source Type	ESI	Ion Polarity	Positive	Set Nebulizer	2.0 Bar
Focus	Active	Set Capillary	4500 V	Set Dry Heater	200 °C
Scan Begin	50 m/z	Set End Plate Offset	-500 V	Set Dry Gas	8.0 l/min
Scan End	3000 m/z	Set Charging Voltage	2000 V	Set Divert Valve	Waste
		Set Corona	0 nA	Set APCI Heater	0 °C

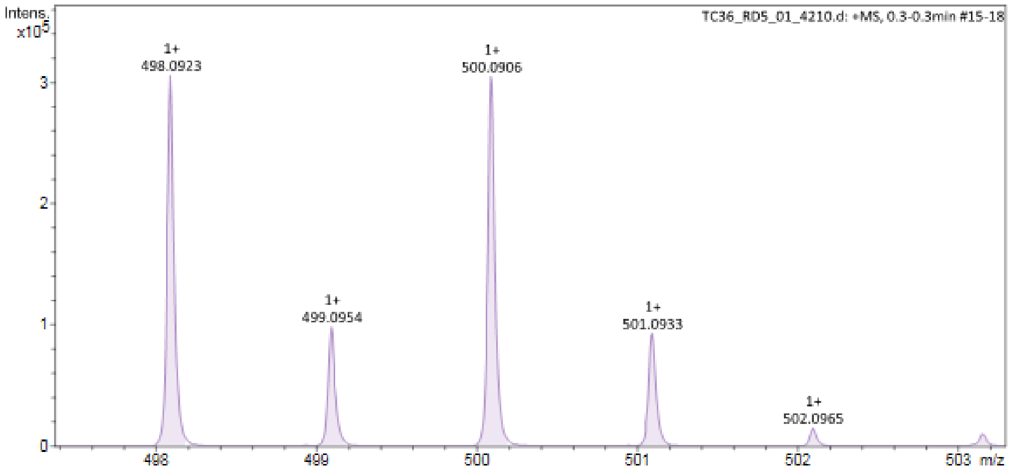

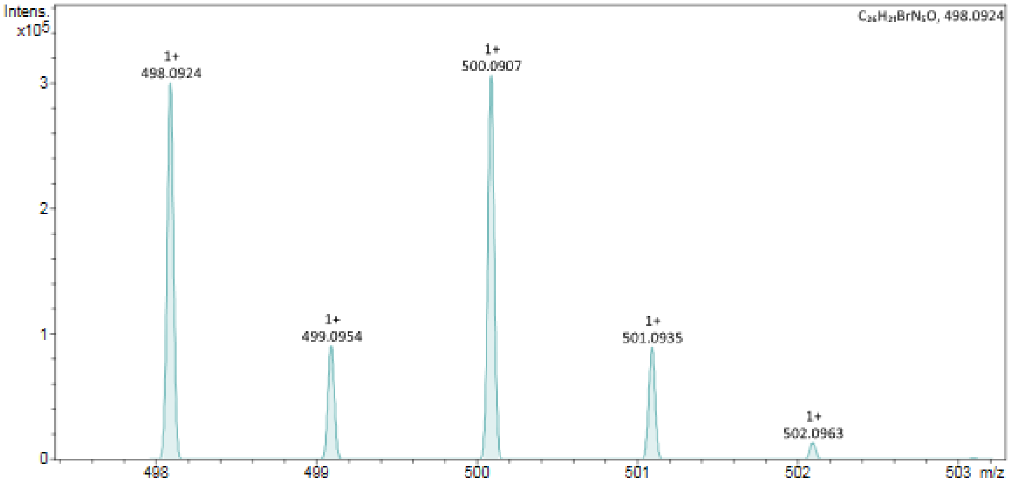

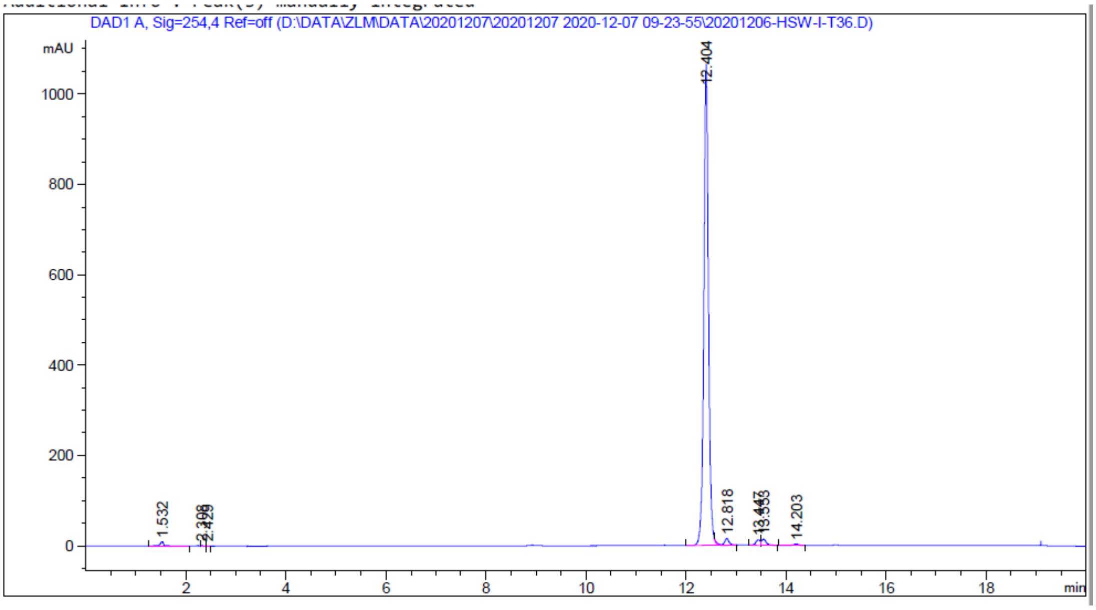

Signal 1: DAD1 A, Sig=254,4 Ref=off

Peak #	RetTime [min]	Type	Width [min]	Area [mAU*s]	Height [mAU]	Area %
1	1.532	BB	0.0720	45.09895	9.23377	0.6313
2	2.308	VV	0.0706	6.12933	1.07295	0.0858
3	2.429	VB	0.0364	3.25009	1.11742	0.0455
4	12.404	BV R	0.0954	6794.58301	1065.76050	95.1108
5	12.818	VB E	0.1044	110.07350	15.21477	1.5408

1260R 12/7/2020 1:29:37 PM BY

Data File D:\DATA\ZLM\DATA\20201207\20201207 2020-12-07 09-23-55\20201206-HSW-I
Sample Name: 20201206-HSW-I-T36

Peak #	RetTime [min]	Type	Width [min]	Area [mAU*s]	Height [mAU]	Area %
6	13.447	BV	0.0880	71.25574	11.96134	0.9974
7	13.553	VB	0.0976	90.60166	13.53109	1.2682
8	14.203	VB R	0.0924	22.87018	3.36207	0.3201

Totals : 7143.86246 1121.25390

^{1}H NMR, ^{13}C NMR, HRMS, and HPLC of compound A21

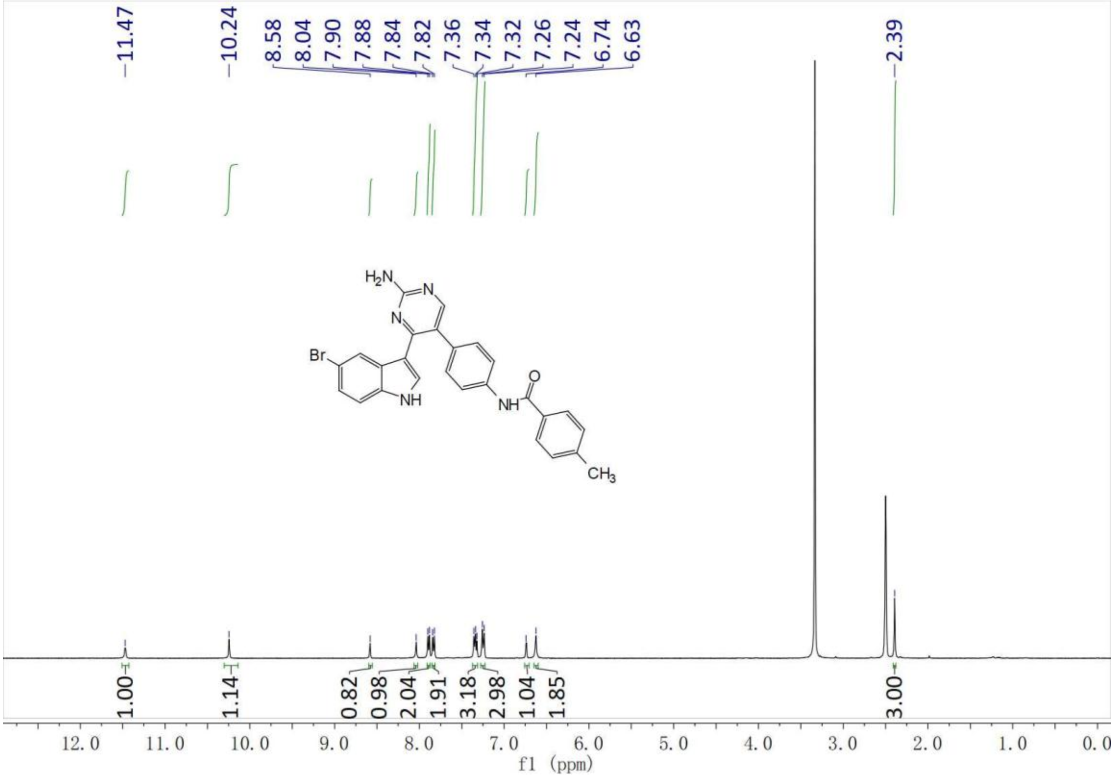

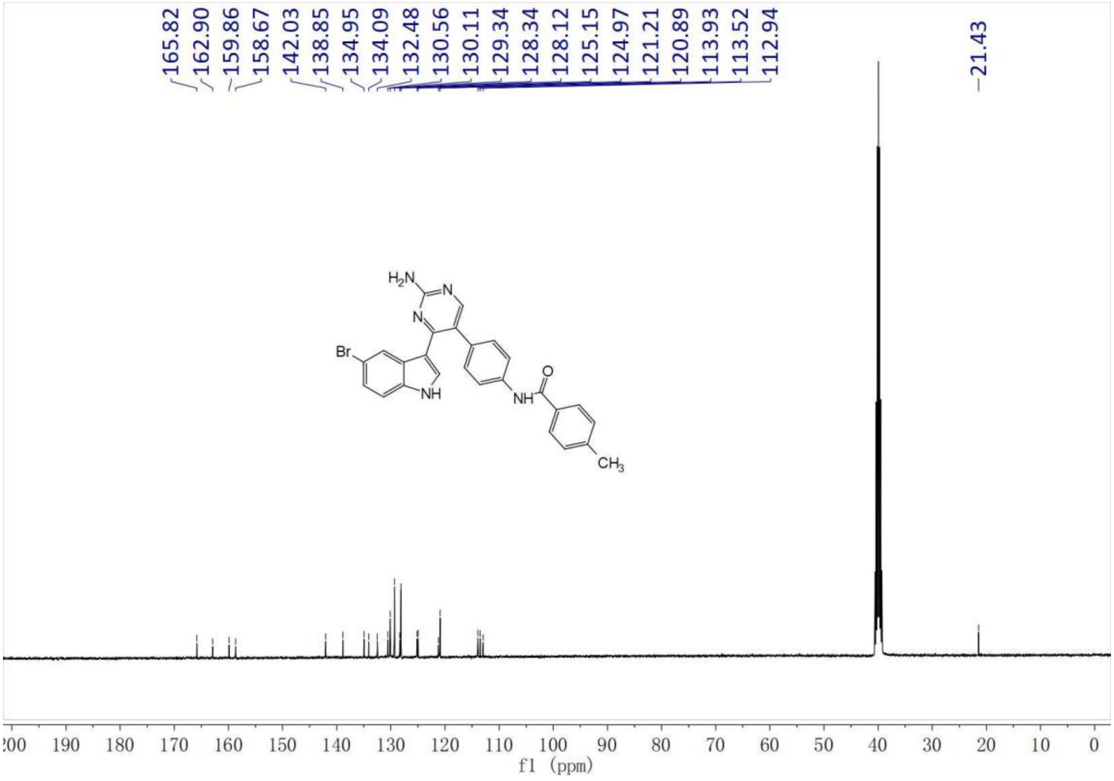

Display Report

Analysis Info

Analysis Name	D:\Data\data\2020\TC6_RA5_01_4187.d	Acquisition Date	10/10/2020 15:18:17 PM
Method	MS-2MIN-POS.m	Operator	BDAL@DE
Sample Name	TC6	Instrument	compact 8255754.20127
Comment			

Acquisition Parameter

Source Type	ESI	Ion Polarity	Positive	Set Nebulizer	2.0 Bar
Focus	Active	Set Capillary	4500 V	Set Dry Heater	200 °C
Scan Begin	50 m/z	Set End Plate Offset	-500 V	Set Dry Gas	8.0 l/min
Scan End	3000 m/z	Set Charging Voltage	2000 V	Set Divert Valve	Waste
		Set Corona	0 nA	Set APCI Heater	0 °C

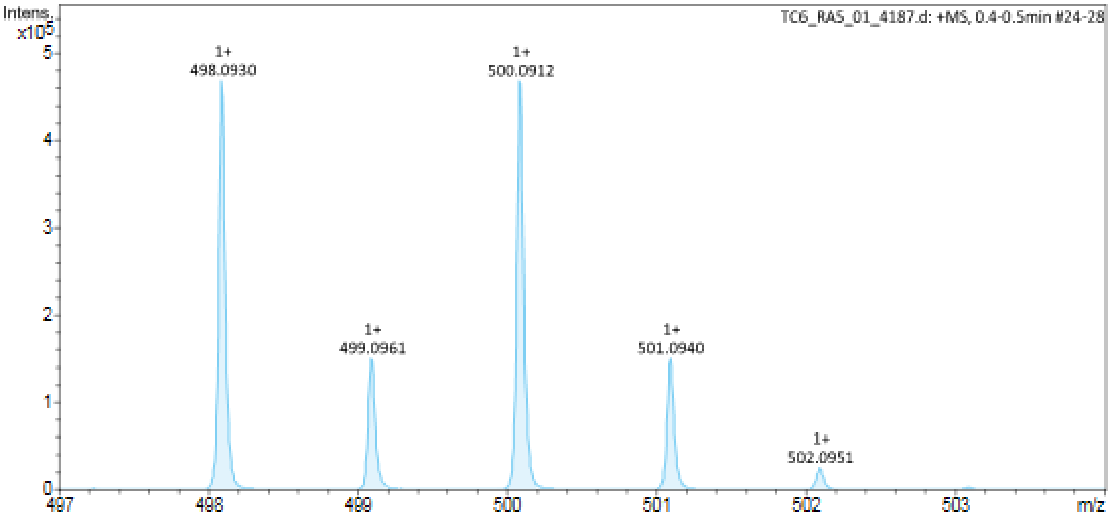

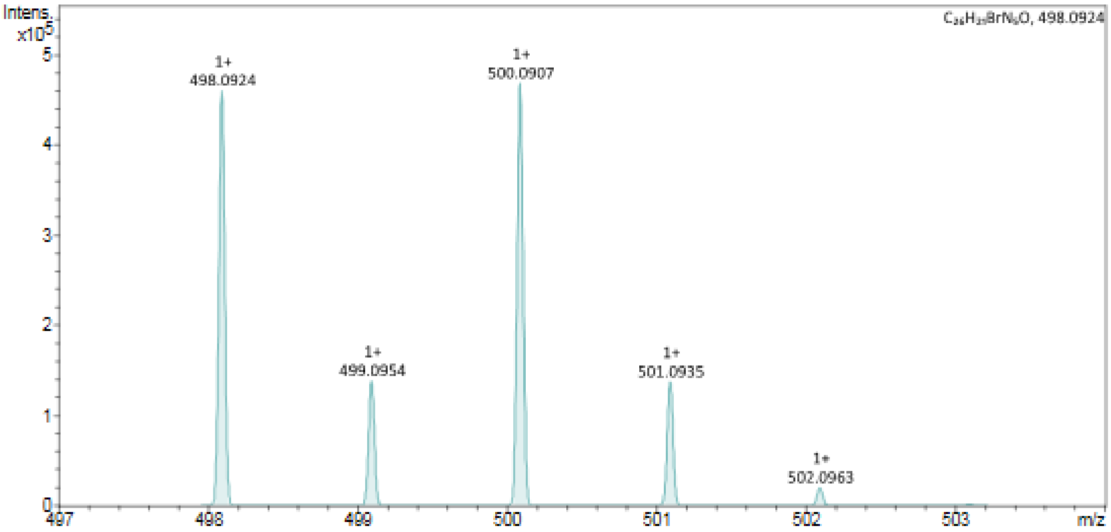

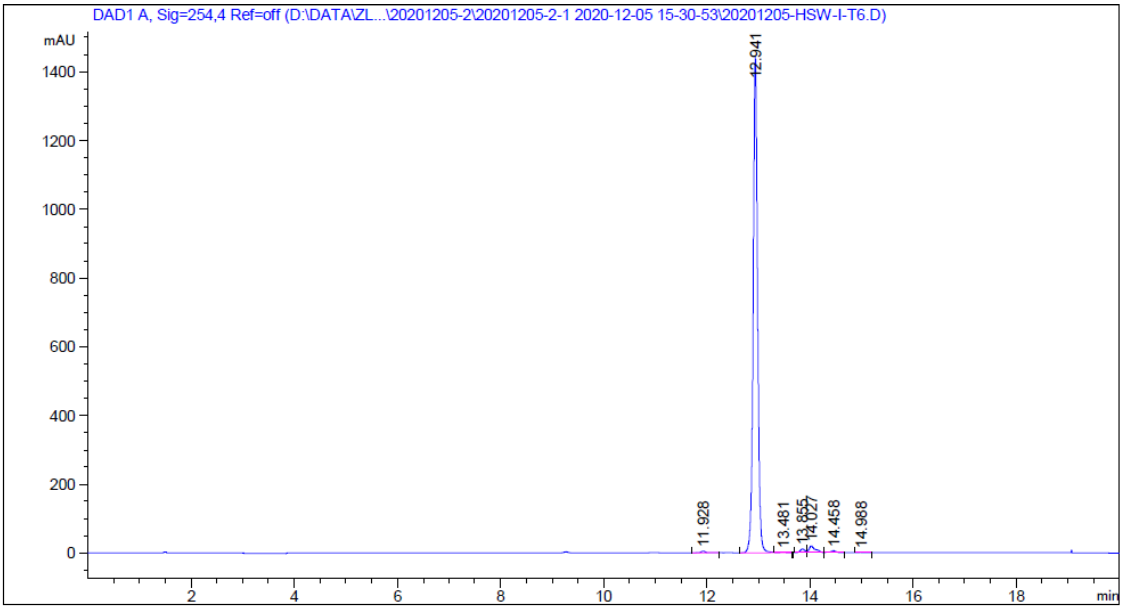

Signal 1: DAD1 A, Sig=254,4 Ref=off

Peak #	RetTime [min]	Type	Width [min]	Area [mAU*s]	Height [mAU]	Area %
1	11.928	BV R	0.0929	33.54079	5.19167	0.3778
2	12.941	BV R	0.0891	8545.58496	1443.16187	96.2623
3	13.481	VB E	0.0907	11.11762	1.53180	0.1252

1260R 12/5/2020 9:09:02 PM BY

Data File D:\DATA\ZLM\DATA\20201205-2\20201205-2-1 2020-12-05 15-30-53\20201205-HSW

Sample Name: 20201205-HSW-I-T6

Peak #	RetTime [min]	Type	Width [min]	Area [mAU*s]	Height [mAU]	Area %
4	13.855	BV	0.0909	62.93402	10.43630	0.7089
5	14.027	VV	0.1207	169.43388	19.52343	1.9086
6	14.458	VB	0.1182	46.95582	5.49611	0.5289
7	14.988	BB	0.0987	7.83022	1.11070	0.0882

Totals : 8877.39733 1486.45187

^{1}H NMR, ^{13}C NMR, HRMS, and HPLC of compound A22

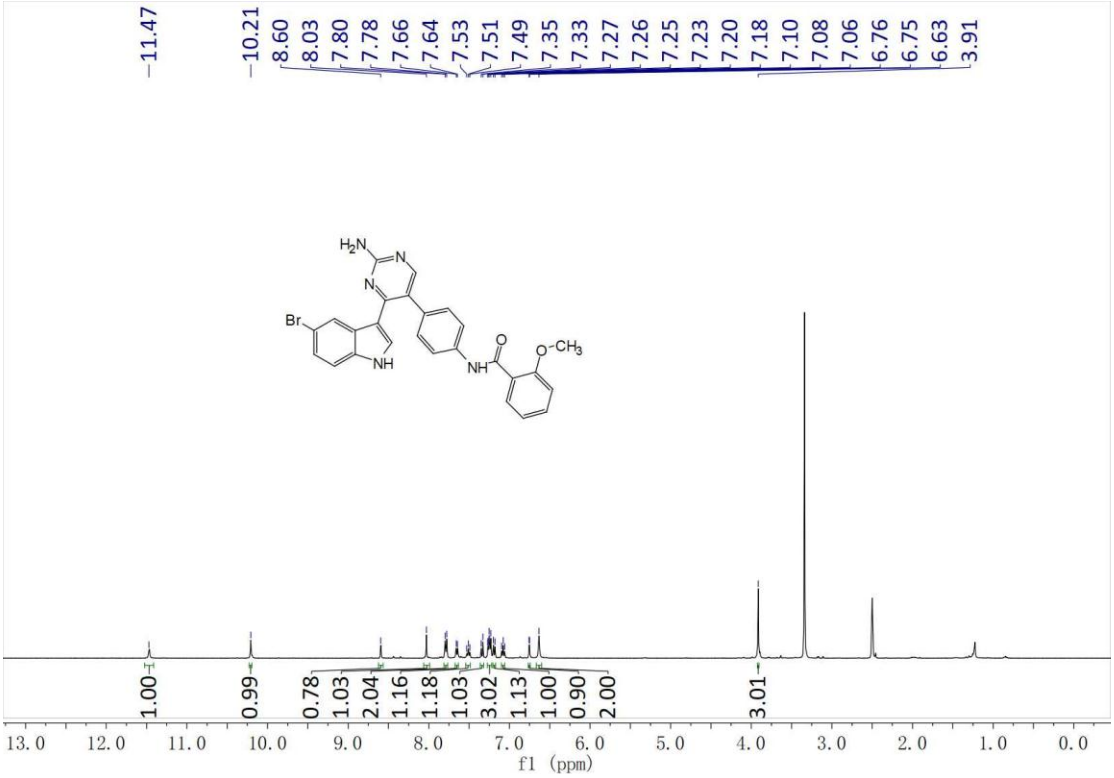

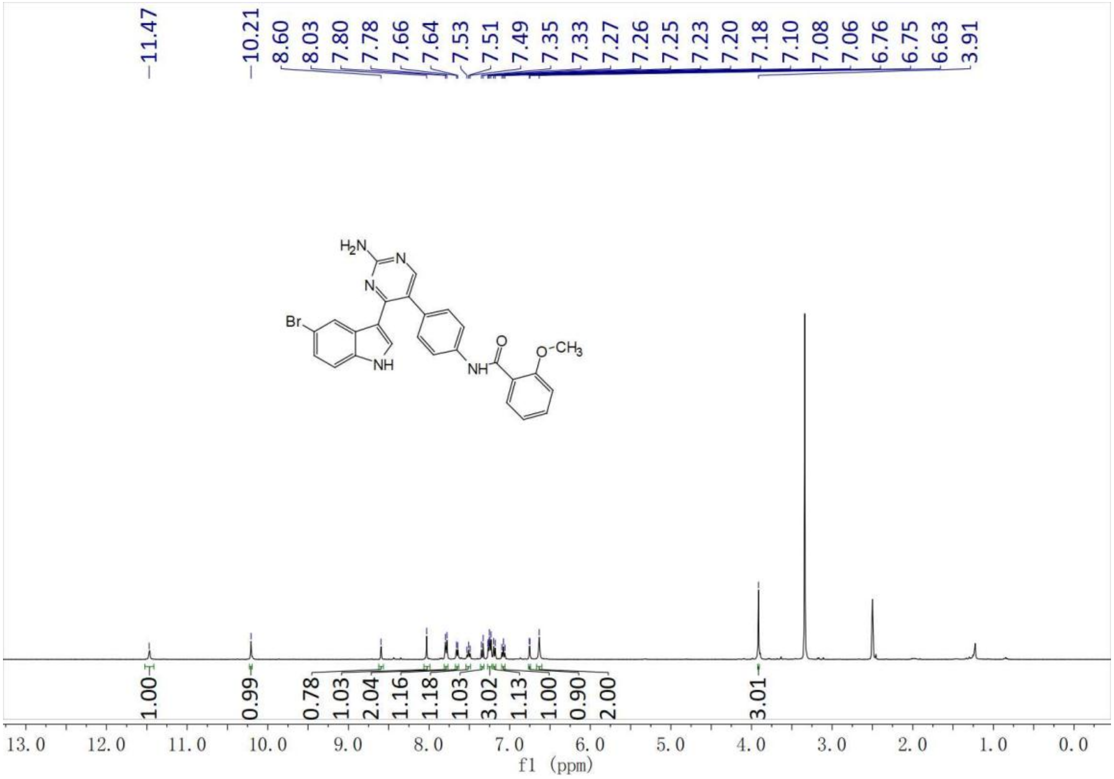

Display Report

Analysis Info

Analysis Name	D:\Data\data\2020\TC32_RD1_01_4206.d	Acquisition Date	10/10/2020 16:10:46 PM
Method	MS-2MIN-POS.m	Operator	BDAL@DE
Sample Name	TC32	Instrument	compact 8255754.20127
Comment			

Acquisition Parameter

Source Type	ESI	Ion Polarity	Positive	Set Nebulizer	2.0 Bar
Focus	Active	Set Capillary	4500 V	Set Dry Heater	200 °C
Scan Begin	50 m/z	Set End Plate Offset	-500 V	Set Dry Gas	8.0 l/min
Scan End	3000 m/z	Set Charging Voltage	2000 V	Set Divert Valve	Waste
		Set Corona	0 nA	Set APCI Heater	0 °C

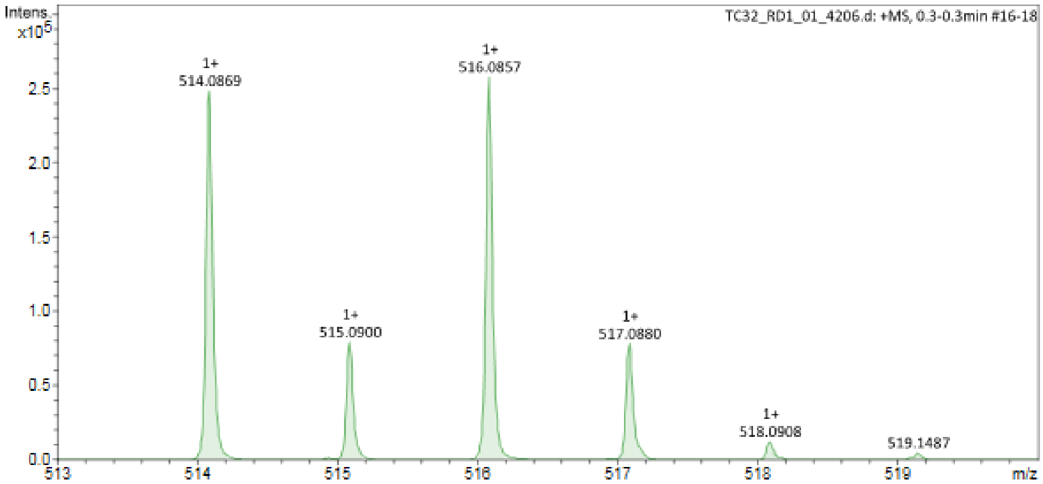

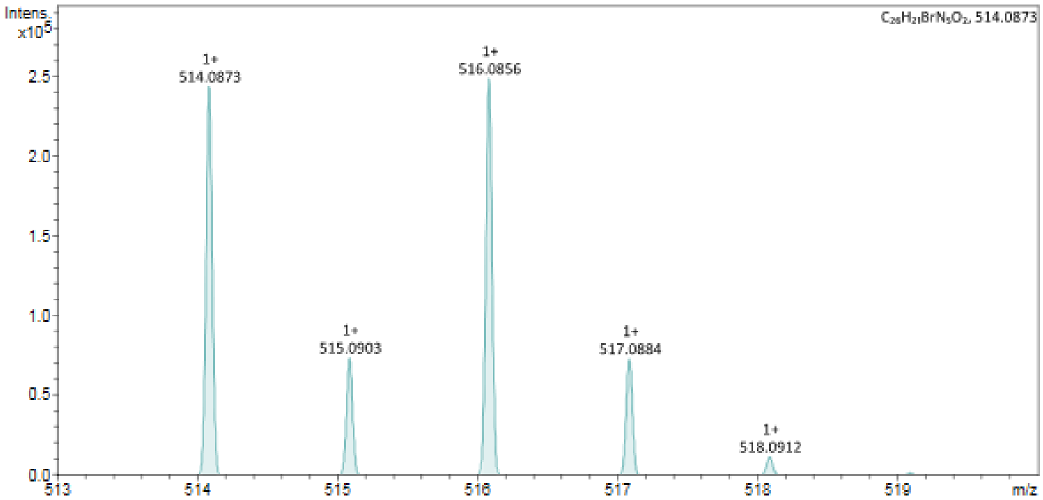

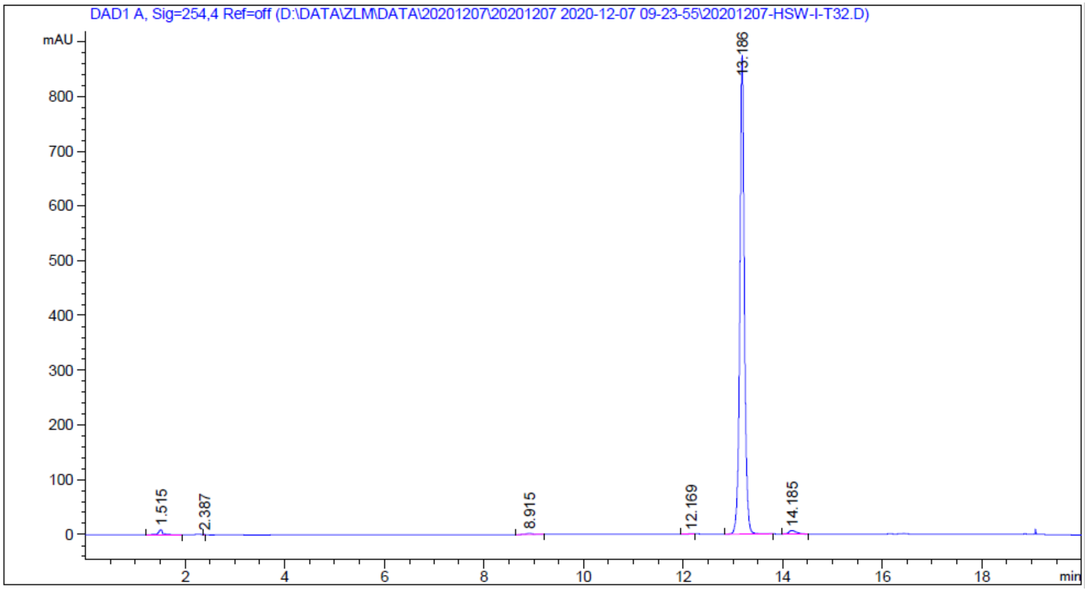

Signal 1: DAD1 A, Sig=254,4 Ref=off

Peak #	RetTime [min]	Type	Width [min]	Area [mAU*s]	Height [mAU]	Area %
1	1.515	VV R	0.0869	50.14472	9.01766	0.8693
2	2.387	VV	0.0267	2.17587	1.07112	0.0377
3	8.915	BV R	0.1076	17.83625	2.11060	0.3092
4	12.169	BV	0.0810	7.59235	1.15784	0.1316
5	13.186	BB	0.0960	5622.59766	874.97559	97.4695

1260R 12/7/2020 1:45:50 PM BY

Data File D:\DATA\ZLM\DATA\20201207\20201207 2020-12-07 09-23-55\20201207
Sample Name: 20201207-HSW-I-T32

Peak #	RetTime [min]	Type	Width [min]	Area [mAU*s]	Height [mAU]	Area %
6	14.185	BB	0.1312	68.22305	6.45946	1.1827

Totals : 5768.56989 894.79226

^{1}H NMR, ^{13}C NMR, HRMS, and HPLC of compound A23

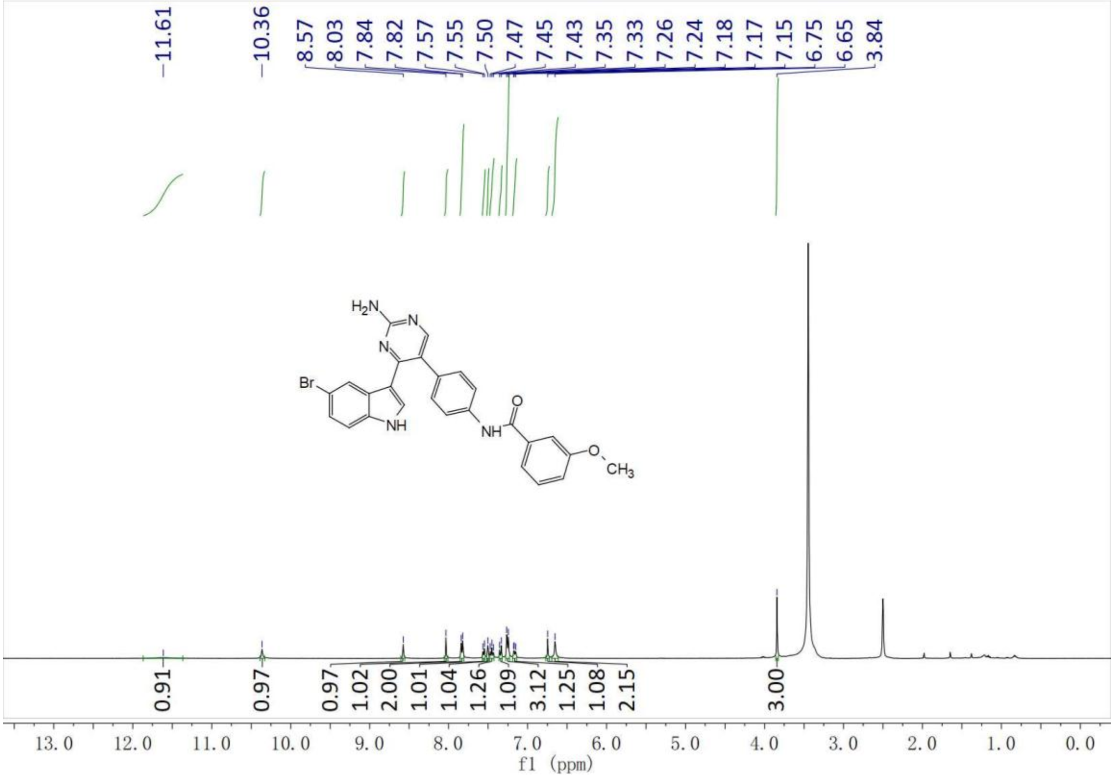

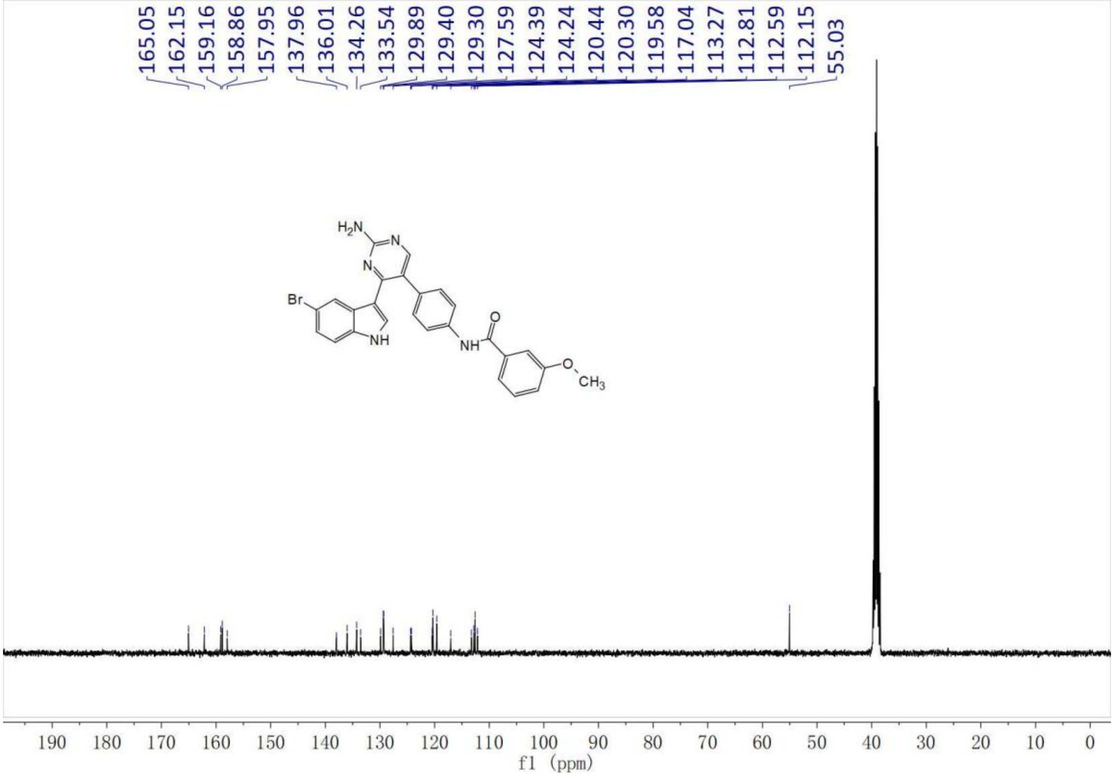

Display Report

Analysis Info

Analysis Name	D:\Data\data\2020\TC33_RD2_01_4207.d	Acquisition Date	10/10/2020 16:13:32 PM
Method	MS-2MIN-POS.m	Operator	BDAL@DE
Sample Name	TC33	Instrument	compact 8255754.20127
Comment			

Acquisition Parameter

Source Type	ESI	Ion Polarity	Positive	Set Nebulizer	2.0 Bar
Focus	Active	Set Capillary	4500 V	Set Dry Heater	200 °C
Scan Begin	50 m/z	Set End Plate Offset	-500 V	Set Dry Gas	8.0 l/min
Scan End	3000 m/z	Set Charging Voltage	2000 V	Set Divert Valve	Waste
		Set Corona	0 nA	Set APCI Heater	0 °C

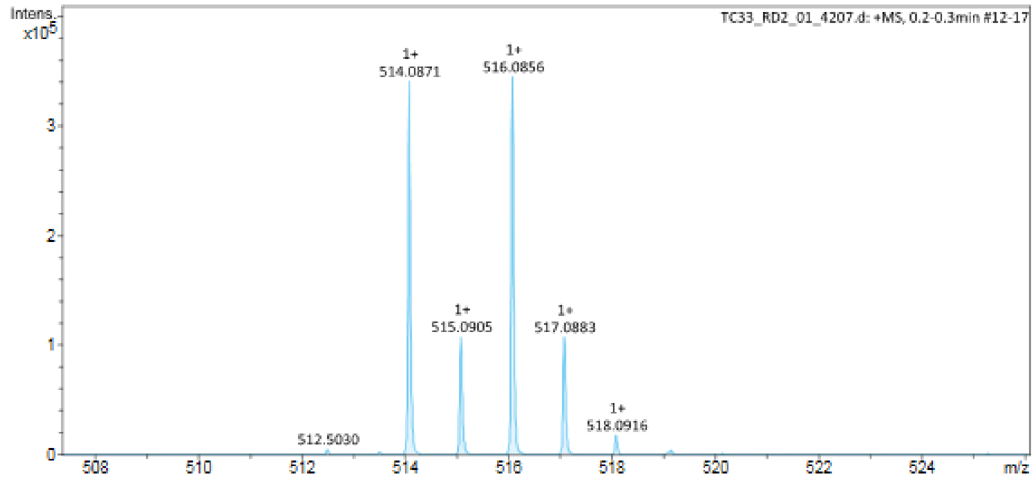

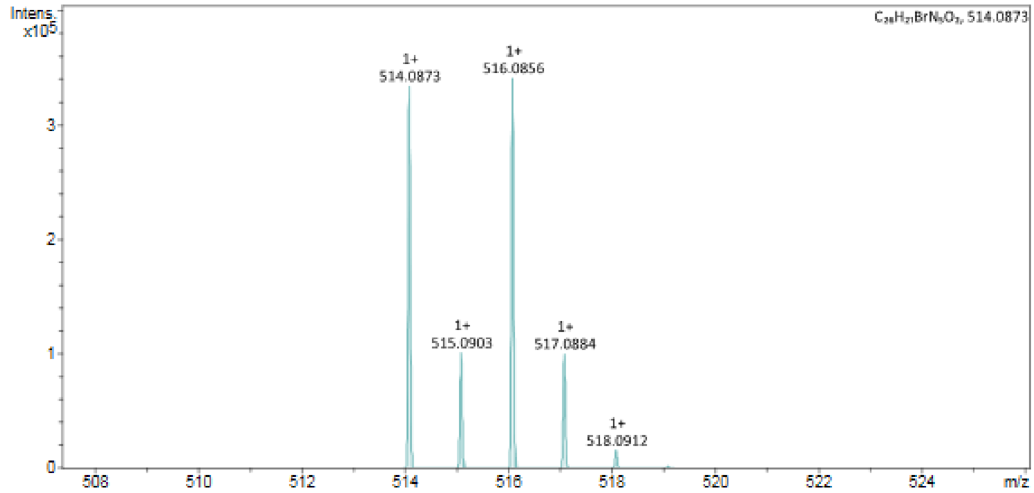

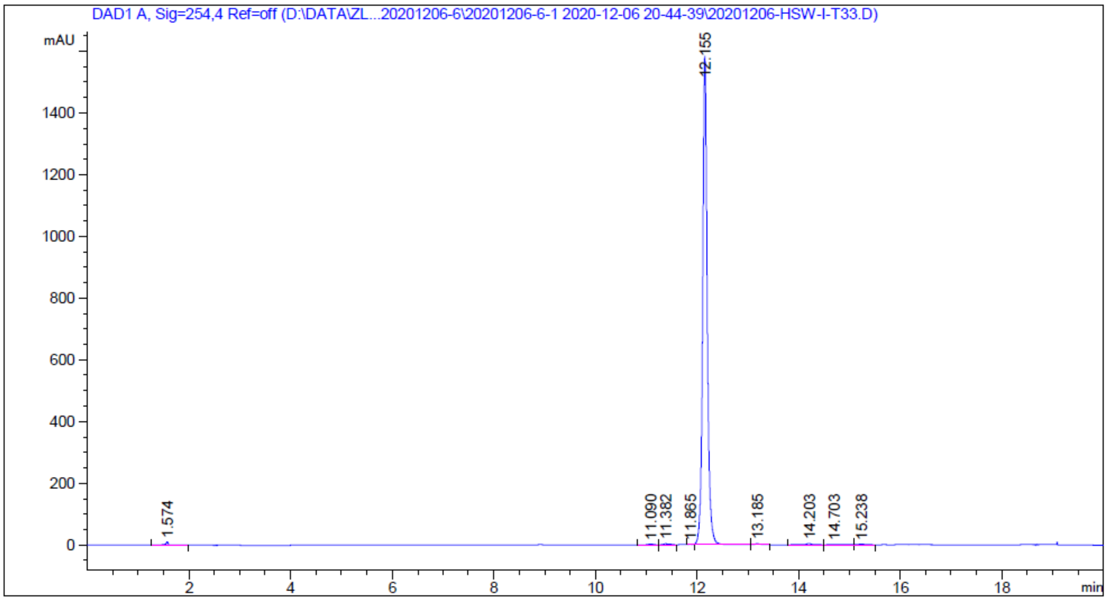

Signal 1: DAD1 A, Sig=254,4 Ref=off

Peak #	RetTime [min]	Type	Width [min]	Area [mAU*s]	Height [mAU]	Area %
1	1.574	BB	0.0528	43.49477	11.33242	0.4299
2	11.090	BV	0.0929	20.23910	3.03051	0.2001
3	11.382	VB	0.1200	31.06946	3.45268	0.3071
4	11.865	BV E	0.0540	4.82987	1.10474	0.0477

1260R 12/7/2020 8:28:56 AM BY

Data File D:\DATA\ZL...TA\20201206-6\20201206-6-1 2020-12-06 20-44-39\20201206-H
Sample Name: 20201206-HSW-I-T33

Peak #	RetTime [min]	Type	Width [min]	Area [mAU*s]	Height [mAU]	Area %
5	12.155	VV R	0.0942	9927.51953	1582.28809	98.1298
6	13.185	BB	0.1038	24.67190	3.22286	0.2439
7	14.203	VB R	0.0968	29.90219	4.09042	0.2956
8	14.703	BV R	0.1240	15.56209	1.51886	0.1538
9	15.238	VB	0.0931	19.43809	2.77889	0.1921

Totals : 1.01167e4 1612.81946

^{1}H NMR, ^{13}C NMR, HRMS, and HPLC of compound A24

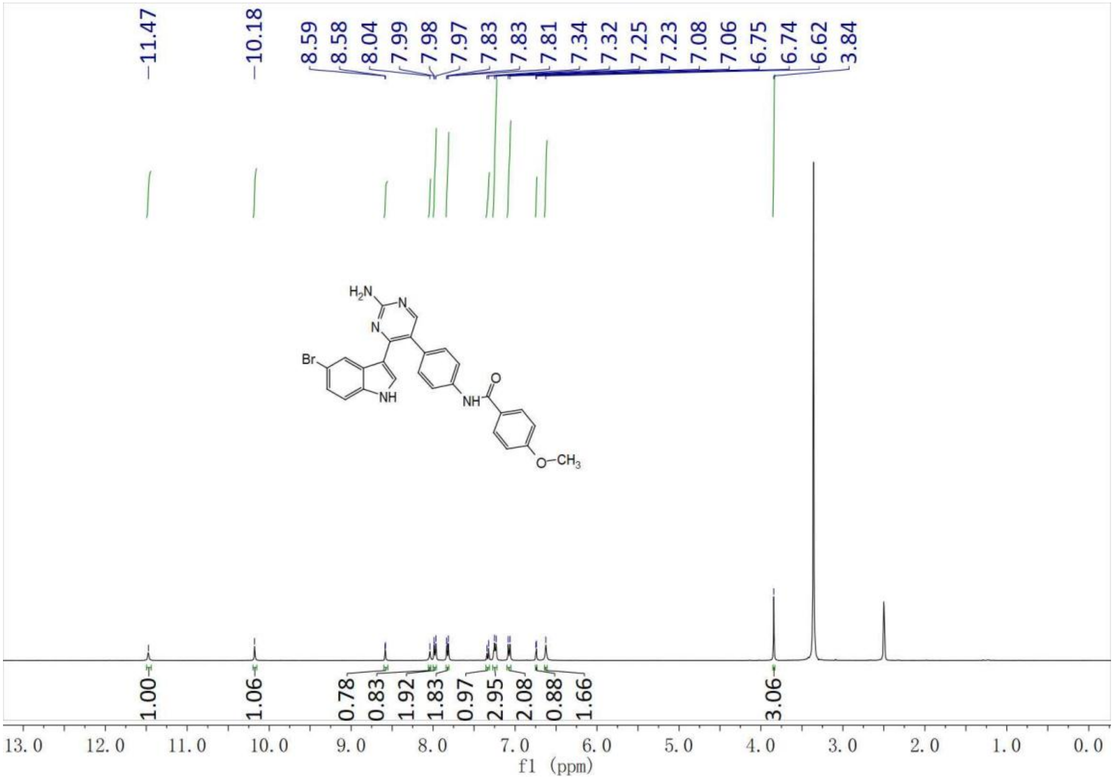

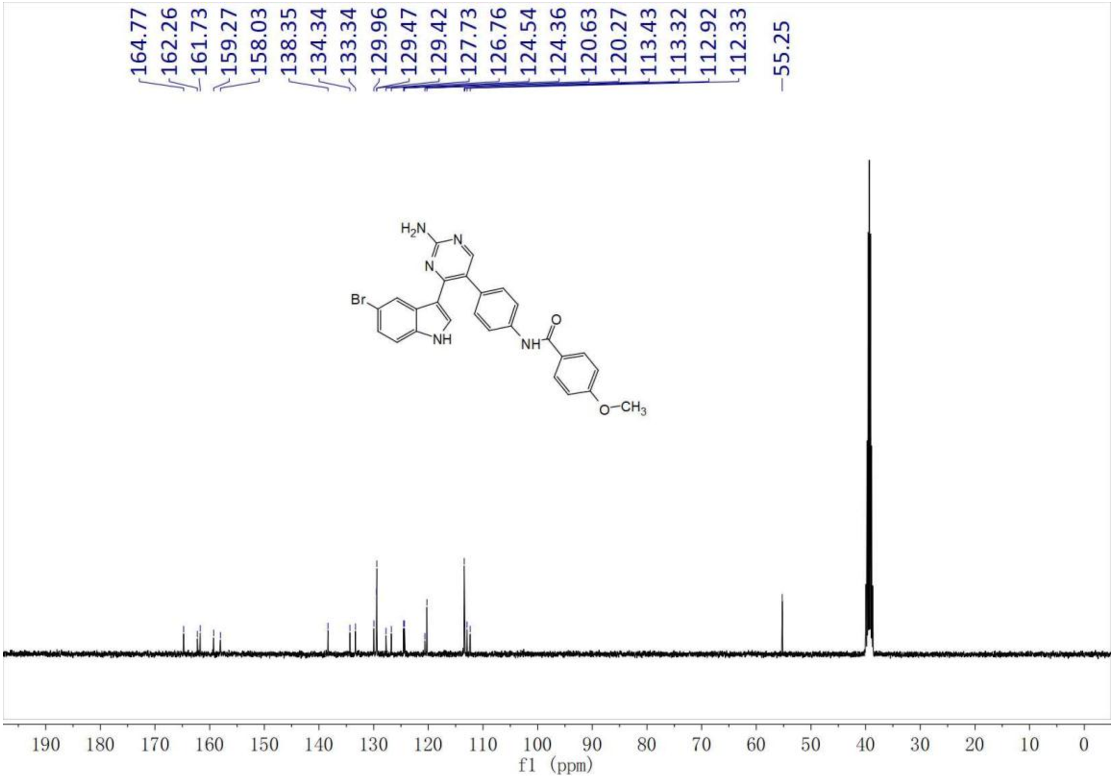

Display Report

Analysis Info

Analysis Name	D:\Data\data\2020\TC7_RA6_01_4188.d	Acquisition Date	10/10/2020 15:21:02 PM
Method	MS-2MIN-POS.m	Operator	BDAL@DE
Sample Name	TC7	Instrument	compact 8255754.20127
Comment			

Acquisition Parameter

Source Type	ESI	Ion Polarity	Positive	Set Nebulizer	2.0 Bar
Focus	Active	Set Capillary	4500 V	Set Dry Heater	200 °C
Scan Begin	50 m/z	Set End Plate Offset	-500 V	Set Dry Gas	8.0 l/min
Scan End	3000 m/z	Set Charging Voltage	2000 V	Set Divert Valve	Waste
		Set Corona	0 nA	Set APCI Heater	0 °C

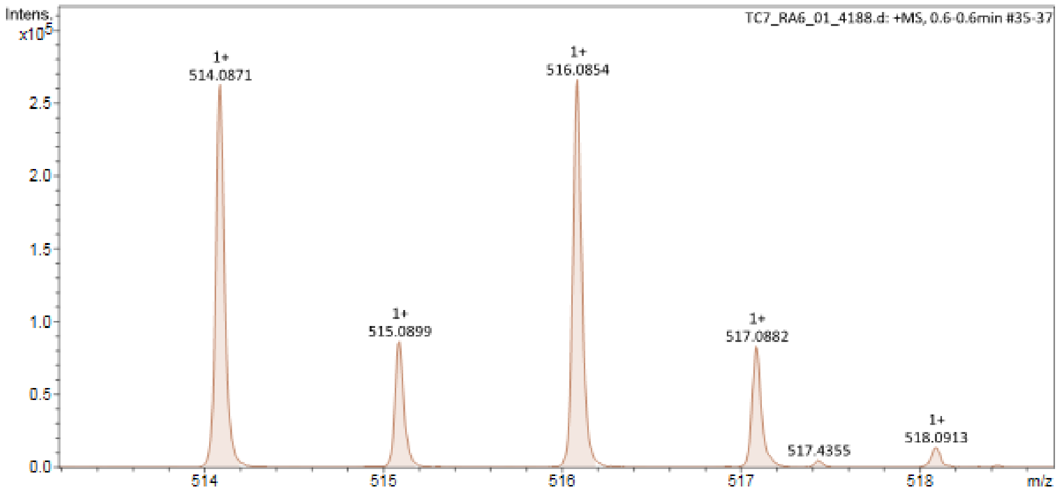

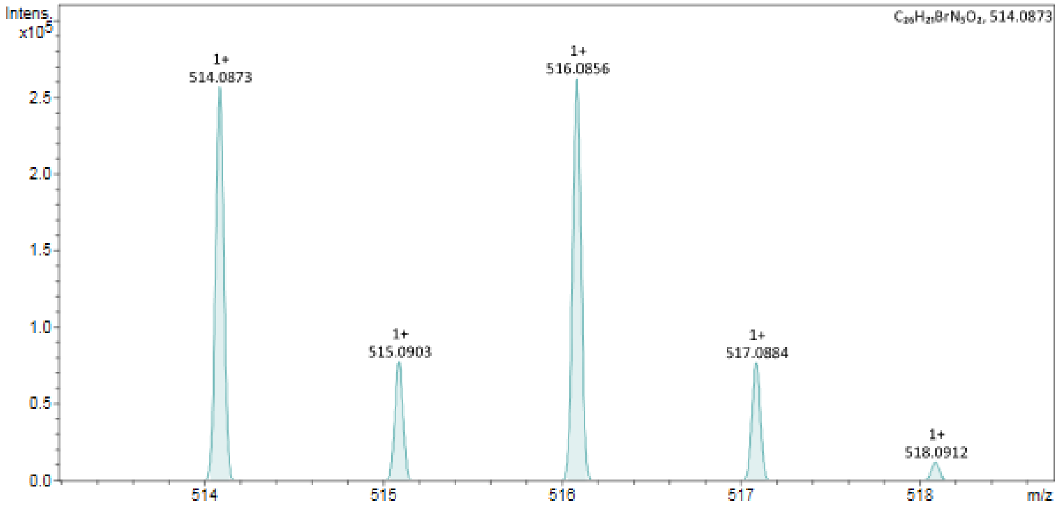

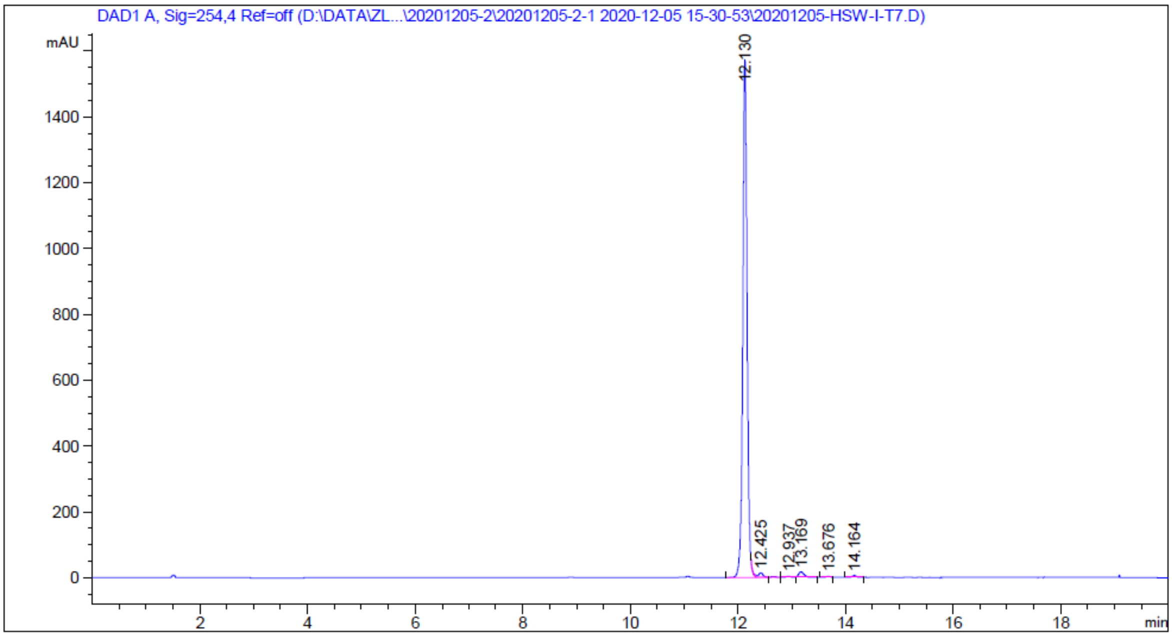

Signal 1: DAD1 A, Sig=254,4 Ref=off

Peak #	RetTime [min]	Type	Width [min]	Area [mAU*s]	Height [mAU]	Area %
1	12.130	BV R	0.0838	8754.78320	1573.76550	96.6668
2	12.425	VV E	0.1093	105.35155	13.46010	1.1633
3	12.937	BV E	0.0849	19.55104	3.38237	0.2159

1260R 12/5/2020 9:11:14 PM BY

Data File D:\DATA\ZLM\DATA\20201205-2\20201205-2-1 2020-12-05 15-30-53\20
Sample Name: 20201205-HSW-I-T7

Peak #	RetTime [min]	Type	Width [min]	Area [mAU*s]	Height [mAU]	Area %
4	13.169	VB R	0.1031	121.59300	17.06179	1.3426
5	13.676	BV	0.0863	20.31712	3.27779	0.2243
6	14.164	BB	0.0843	35.05997	5.98745	0.3871

Totals : 9056.65589 1616.93500

^{1}H NMR, ^{13}C NMR, HRMS, and HPLC of compound A25

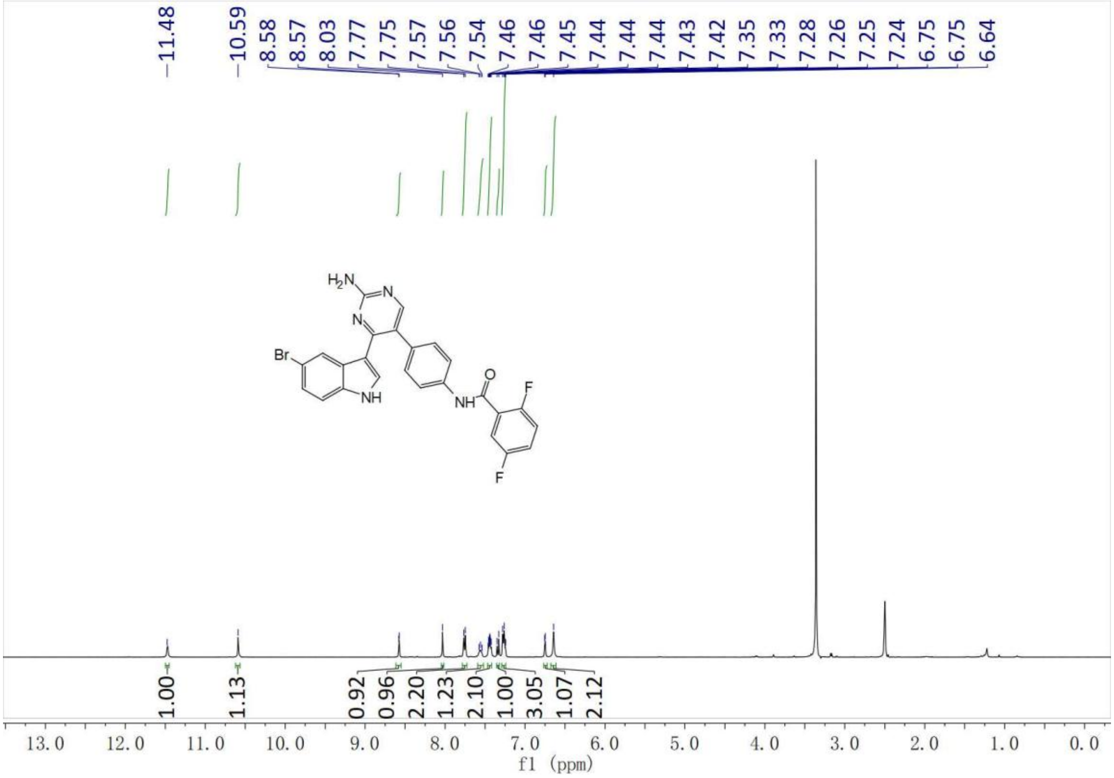

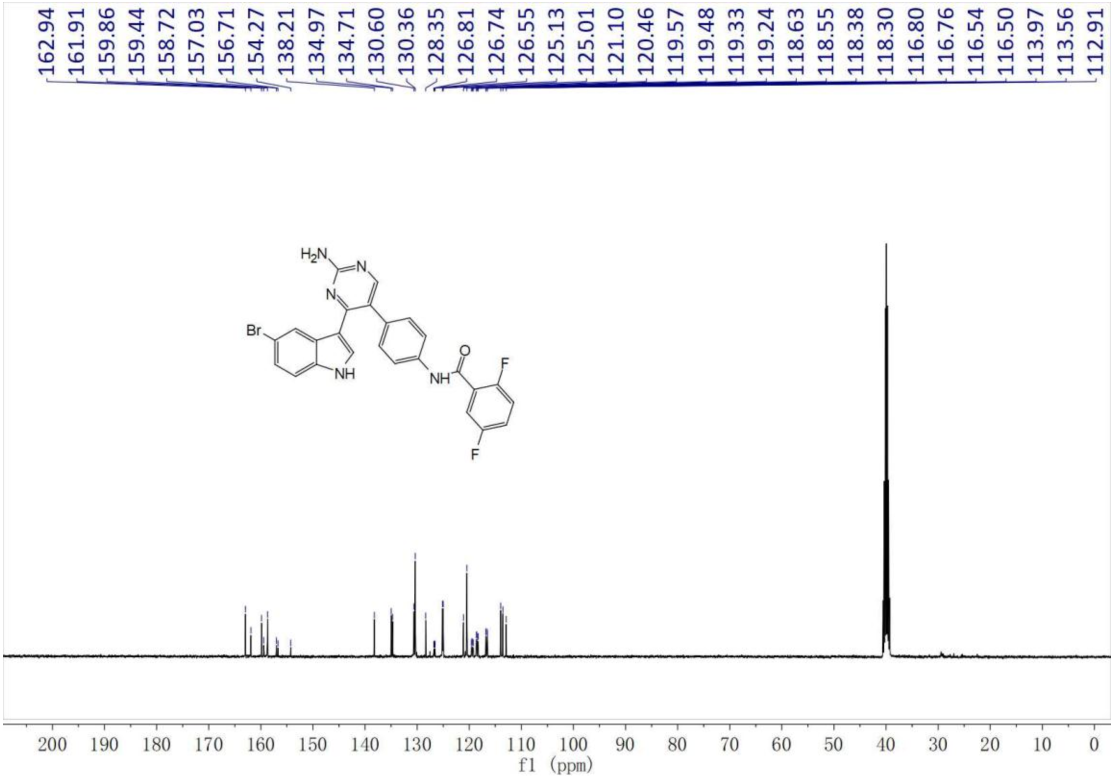

Display Report

Analysis Info

Analysis Name	D:\Data\data\2020\TC16_RB7_01_4196.d	Acquisition Date	10/10/2020 15:43:08 PM
Method	MS-2MIN-POS.m	Operator	BDAL@DE
Sample Name	TC16	Instrument	compact 8255754.20127
Comment			

Acquisition Parameter

Source Type	ESI	Ion Polarity	Positive	Set Nebulizer	2.0 Bar
Focus	Active	Set Capillary	4500 V	Set Dry Heater	200 °C
Scan Begin	50 m/z	Set End Plate Offset	-500 V	Set Dry Gas	8.0 l/min
Scan End	3000 m/z	Set Charging Voltage	2000 V	Set Divert Valve	Waste
		Set Corona	0 nA	Set APCI Heater	0 °C

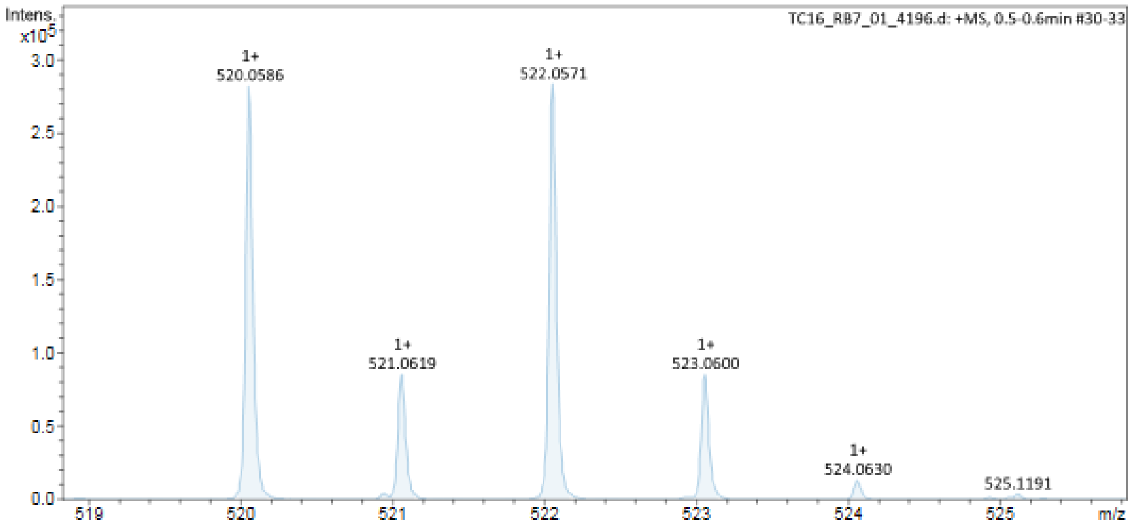

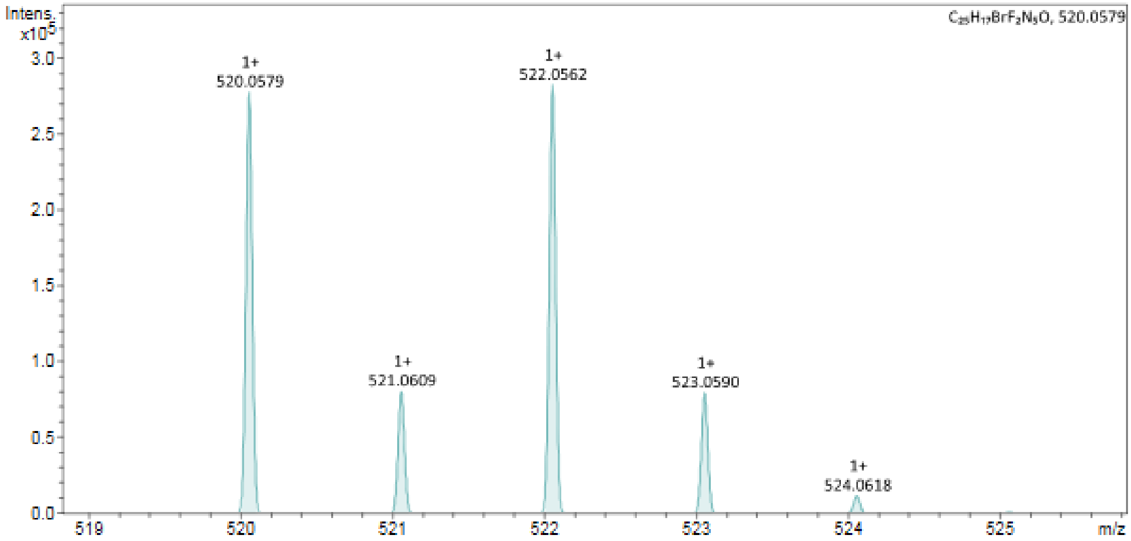

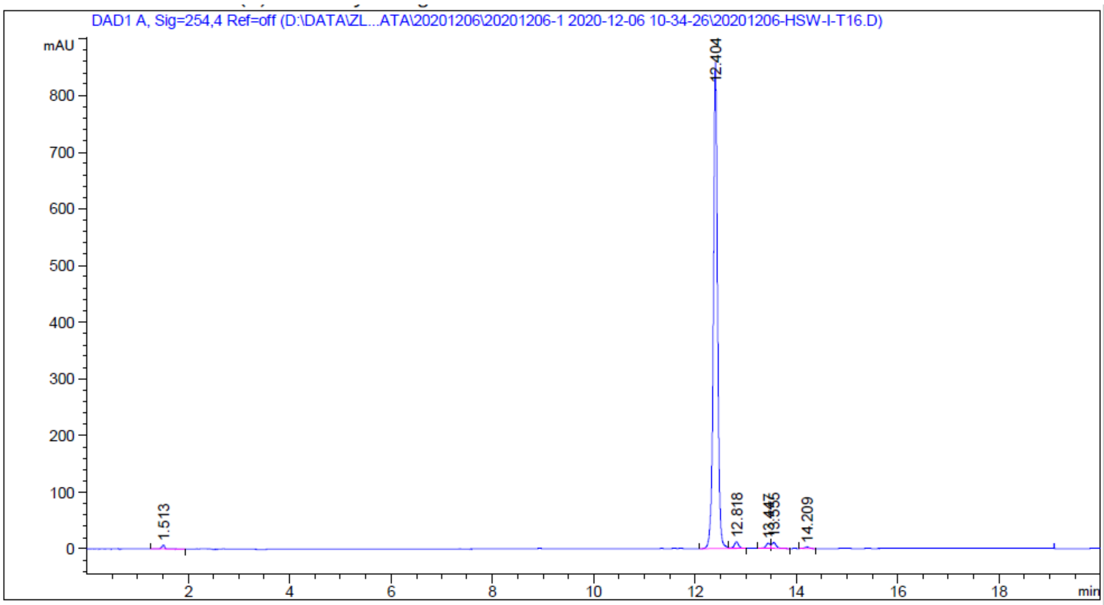

Signal 1: DAD1 A, Sig=254,4 Ref=off

Peak #	RetTime [min]	Type	Width [min]	Area [mAU*s]	Height [mAU]	Area %
1	1.513	BB	0.0728	34.16001	6.95728	0.6602
2	12.404	BV R	0.0856	4937.53271	859.11450	95.4257
3	12.818	VB E	0.0861	67.05705	11.66411	1.2960

1260R 12/6/2020 2:37:10 PM BY

Data File D:\DATA\ZLM\DATA\20201206\20201206-1 2020-12-06 10-34-26\202

Sample Name: 20201206-HSW-I-T16

Peak #	RetTime [min]	Type	Width [min]	Area [mAU*s]	Height [mAU]	Area %
4	13.447	BV	0.0842	54.02493	9.37237	1.0441
5	13.555	VB	0.0910	65.61750	10.55920	1.2682
6	14.209	BB	0.0757	15.82607	2.68750	0.3059

Totals : 5174.21828 900.35495

^{1}H NMR, ^{13}C NMR, HRMS, and HPLC of compound A26

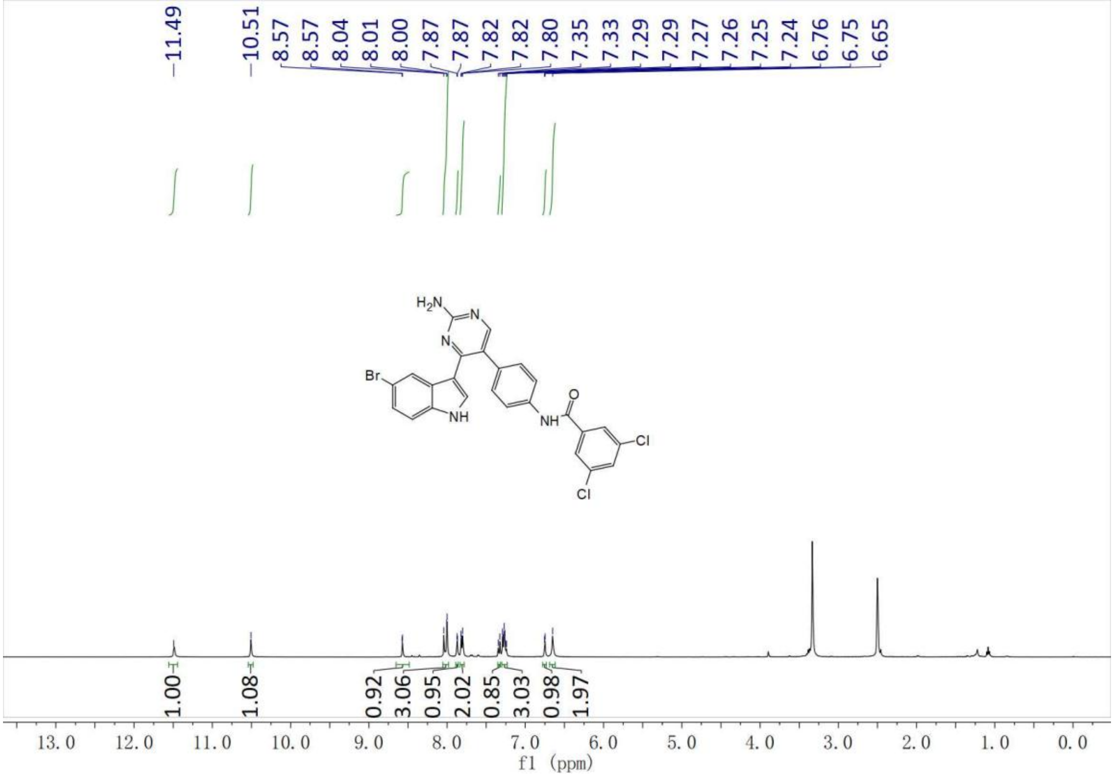

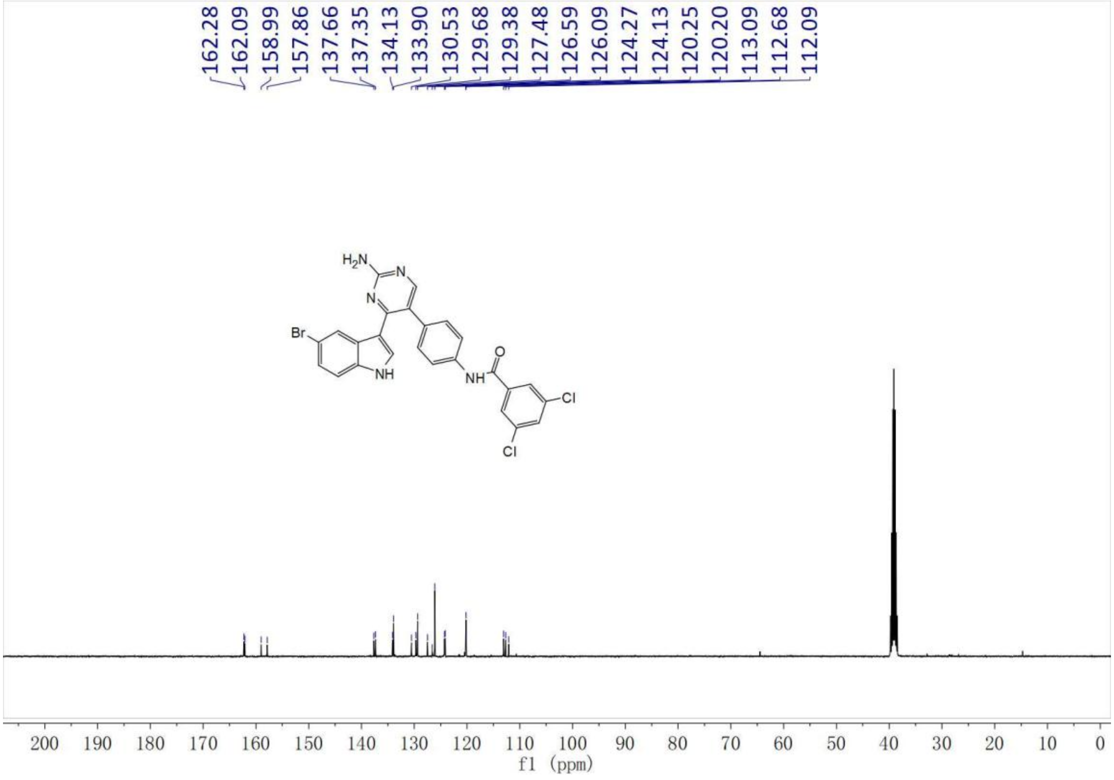

Display Report

Analysis Info

Analysis Name	D:\Data\data\2020\TC19_RC1_01_4198.d	Acquisition Date	10/10/2020 15:48:40 PM
Method	MS-2MIN-POS.m	Operator	BDAL@DE
Sample Name	TC19	Instrument	compact 8255754.20127
Comment			

Acquisition Parameter

Source Type	ESI	Ion Polarity	Positive	Set Nebulizer	2.0 Bar
Focus	Active	Set Capillary	4500 V	Set Dry Heater	200 °C
Scan Begin	50 m/z	Set End Plate Offset	-500 V	Set Dry Gas	8.0 l/min
Scan End	3000 m/z	Set Charging Voltage	2000 V	Set Divert Valve	Waste
		Set Corona	0 nA	Set APCI Heater	0 °C

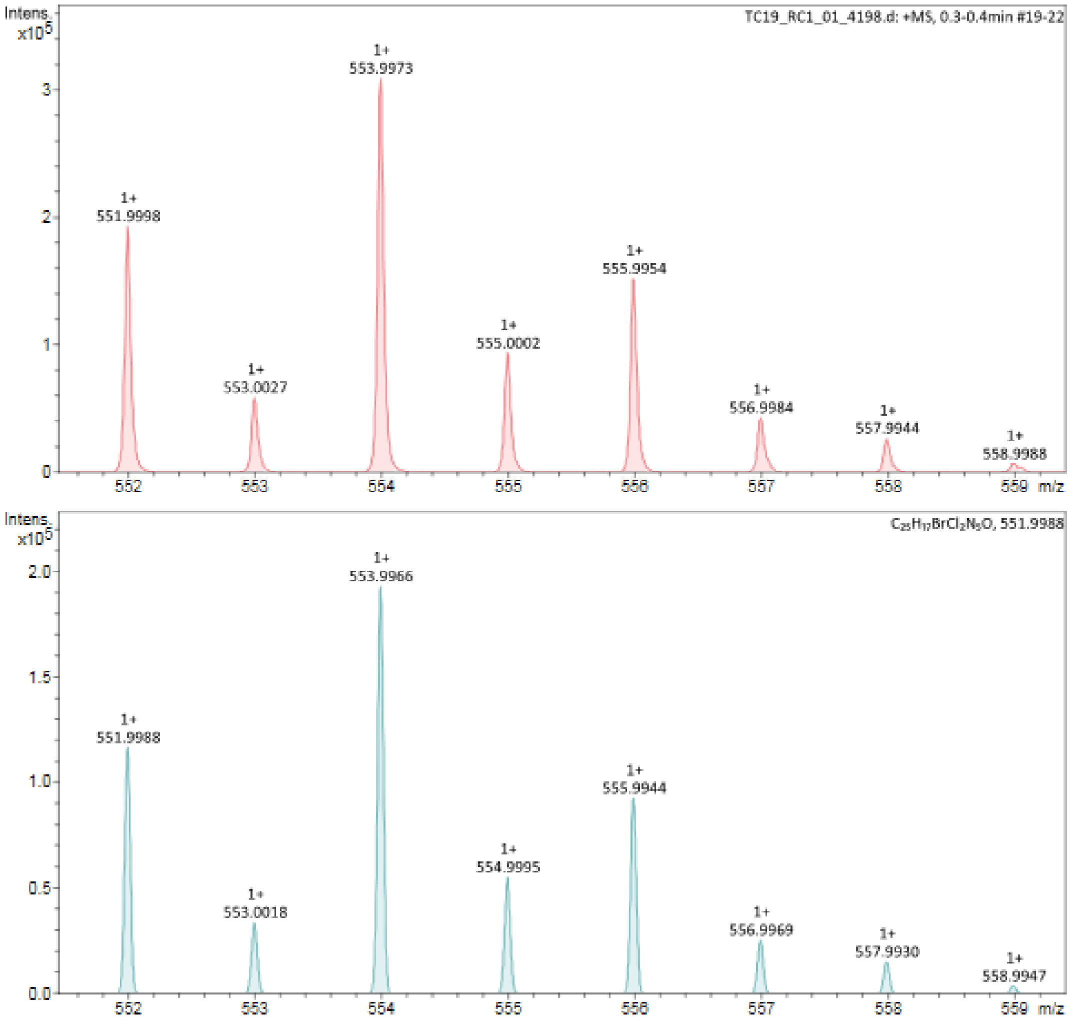

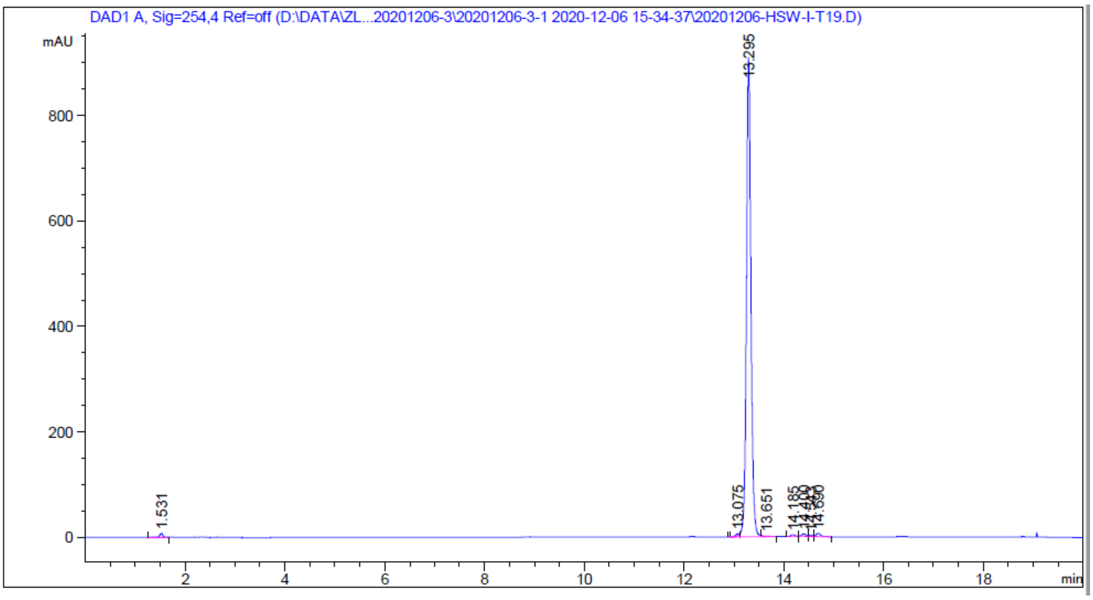

Signal 1: DAD1 A, Sig=254,4 Ref=off

Peak #	RetTime [min]	Type	Width [min]	Area [mAU*s]	Height [mAU]	Area %
1	1.531	BB	0.0685	32.15469	7.28910	0.5508
2	13.075	BV E	0.0764	20.70352	4.03016	0.3547
3	13.295	VV R	0.0938	5672.12402	909.13550	97.1682

1260R 12/6/2020 7:27:42 PM BY

Data File D:\DATA\ZL...TA\20201206-3\20201206-3-1 2020-12-06 15-34-37\20201
Sample Name: 20201206-HSW-I-T19

Peak #	RetTime [min]	Type	Width [min]	Area [mAU*s]	Height [mAU]	Area %
4	13.651	VB E	0.0882	8.75169	1.17136	0.1499
5	14.185	BB	0.0741	18.74560	3.64014	0.3211
6	14.400	BV	0.0876	28.96431	4.68481	0.4962
7	14.543	VV	0.0707	15.57397	2.72413	0.2668
8	14.690	VB	0.0926	40.40864	6.35913	0.6922

Totals : 5837.42645 939.03431

^{1}H NMR, ^{13}C NMR, HRMS, and HPLC of compound A27

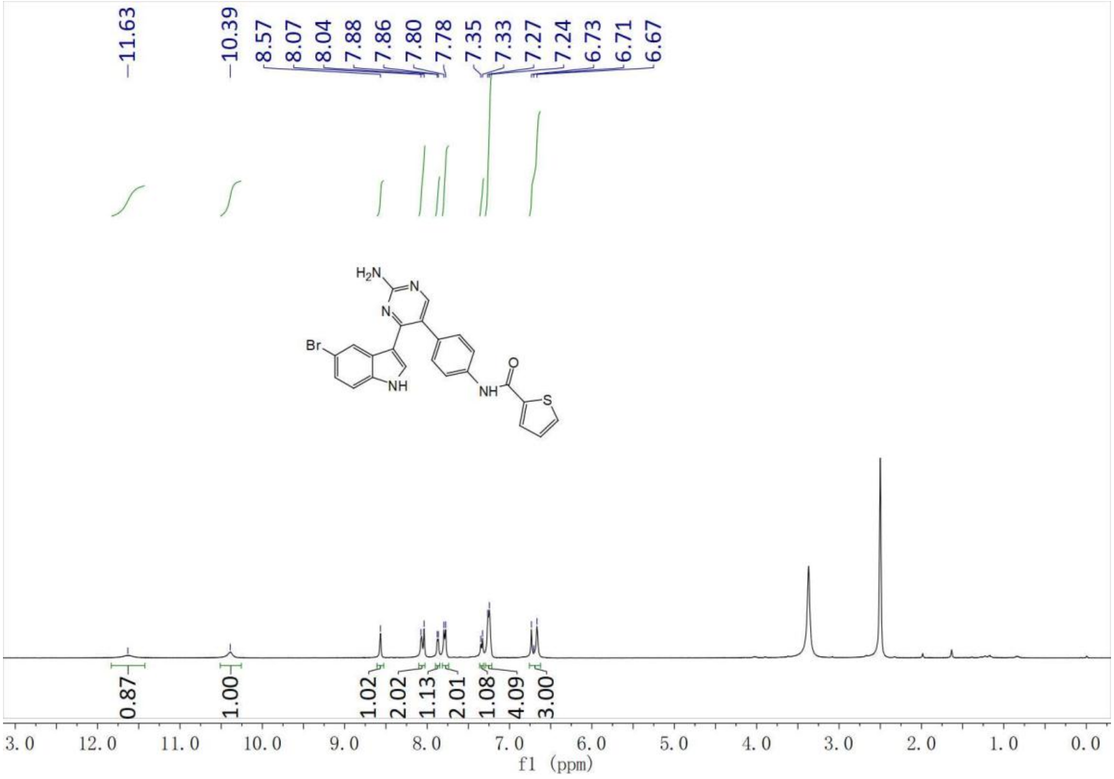

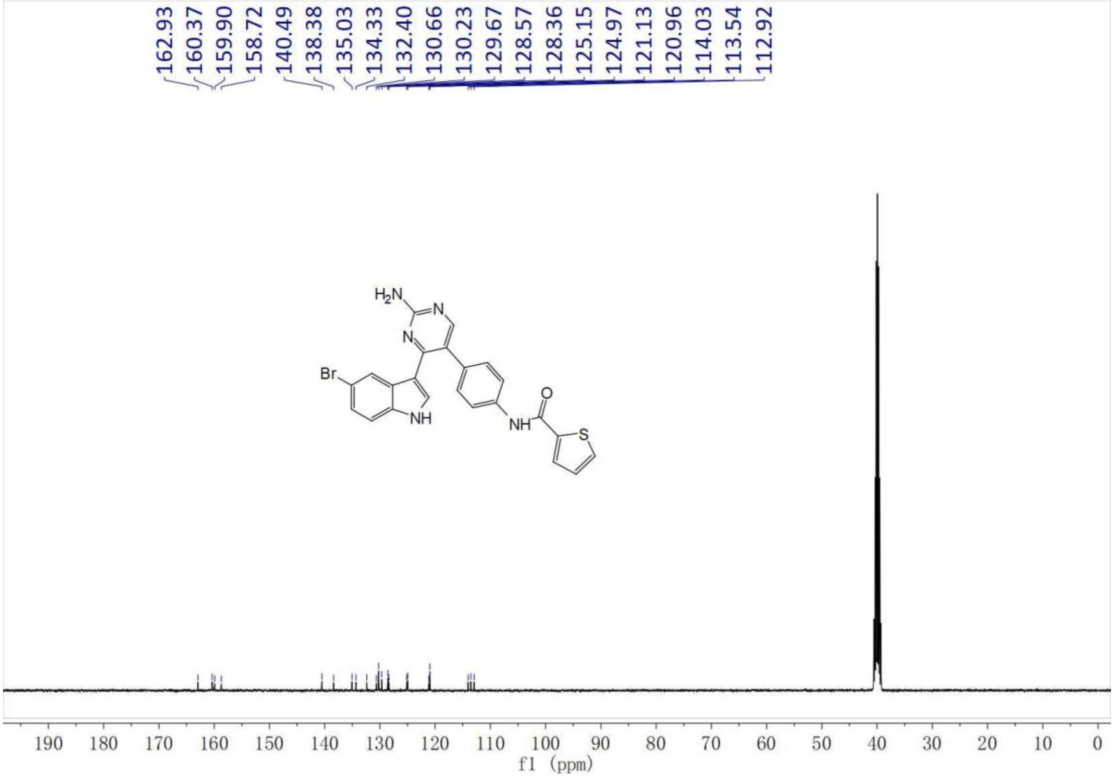

Display Report

Analysis Info

Analysis Name	D:\Data\data\2020\TC37_RD6_01_4211.d	Acquisition Date	10/10/2020 16:24:35 PM
Method	MS-2MIN-POS.m	Operator	BDAL@DE
Sample Name	TC37	Instrument	compact 8255754.20127
Comment			

Acquisition Parameter

Source Type	ESI	Ion Polarity	Positive	Set Nebulizer	2.0 Bar
Focus	Active	Set Capillary	4500 V	Set Dry Heater	200 °C
Scan Begin	50 m/z	Set End Plate Offset	-500 V	Set Dry Gas	8.0 l/min
Scan End	3000 m/z	Set Charging Voltage	2000 V	Set Divert Valve	Waste
		Set Corona	0 nA	Set APCI Heater	0 °C

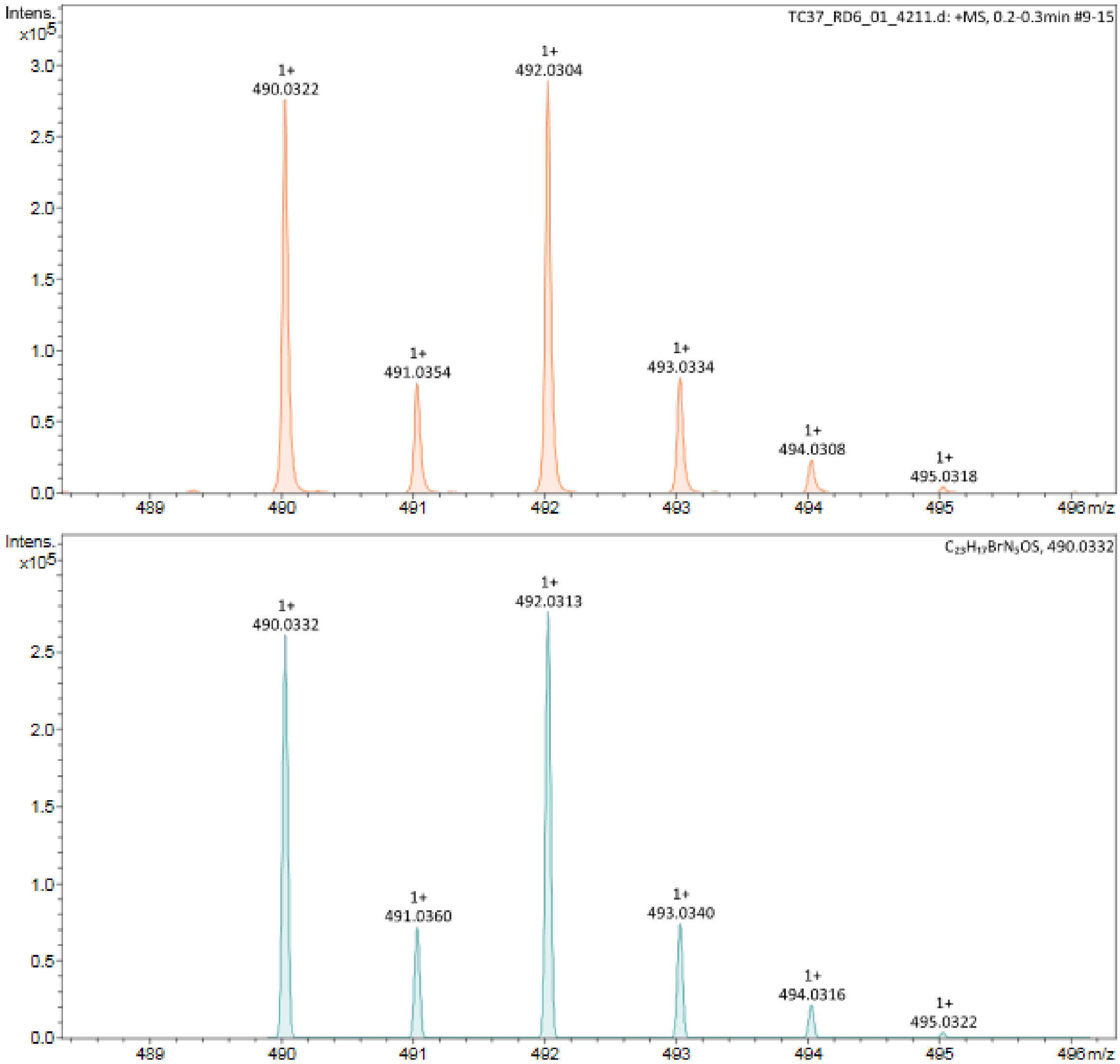

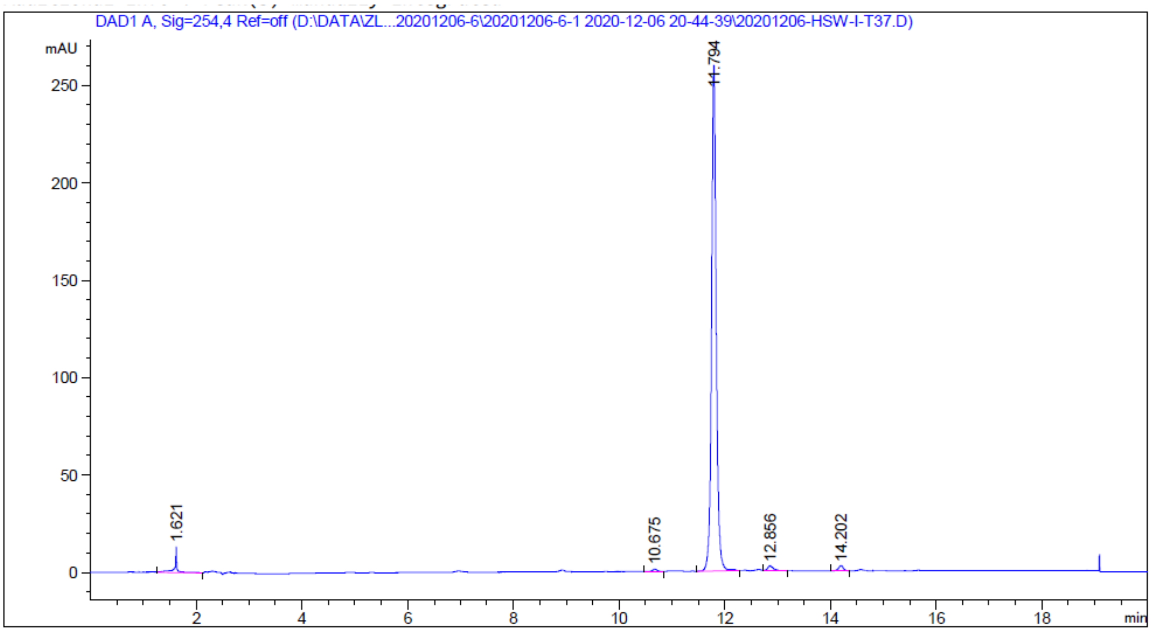

Signal 1: DAD1 A, Sig=254,4 Ref=off

Peak #	RetTime [min]	Type	Width [min]	Area [mAU*s]	Height [mAU]	Area %
1	1.621	VB R	0.0346	34.94706	13.31781	2.0844
2	10.675	BV	0.0743	7.61604	1.25432	0.4543
3	11.794	VV R	0.0922	1596.45264	259.86557	95.2215
4	12.856	VV R	0.0968	20.32247	2.55419	1.2121

1260R 12/7/2020 8:32:57 AM BY

Data File D:\DATA\ZL...TA\20201206-6\20201206-6-1 2020-12-06 20-44-39\2€
Sample Name: 20201206-HSW-I-T37

Peak #	RetTime [min]	Type	Width [min]	Area [mAU*s]	Height [mAU]	Area %
5	14.202	BB	0.0919	17.22996	2.73820	1.0277

Totals : 1676.56818 279.73009

^{1}H NMR, ^{13}C NMR, HRMS, and HPLC of compound A28

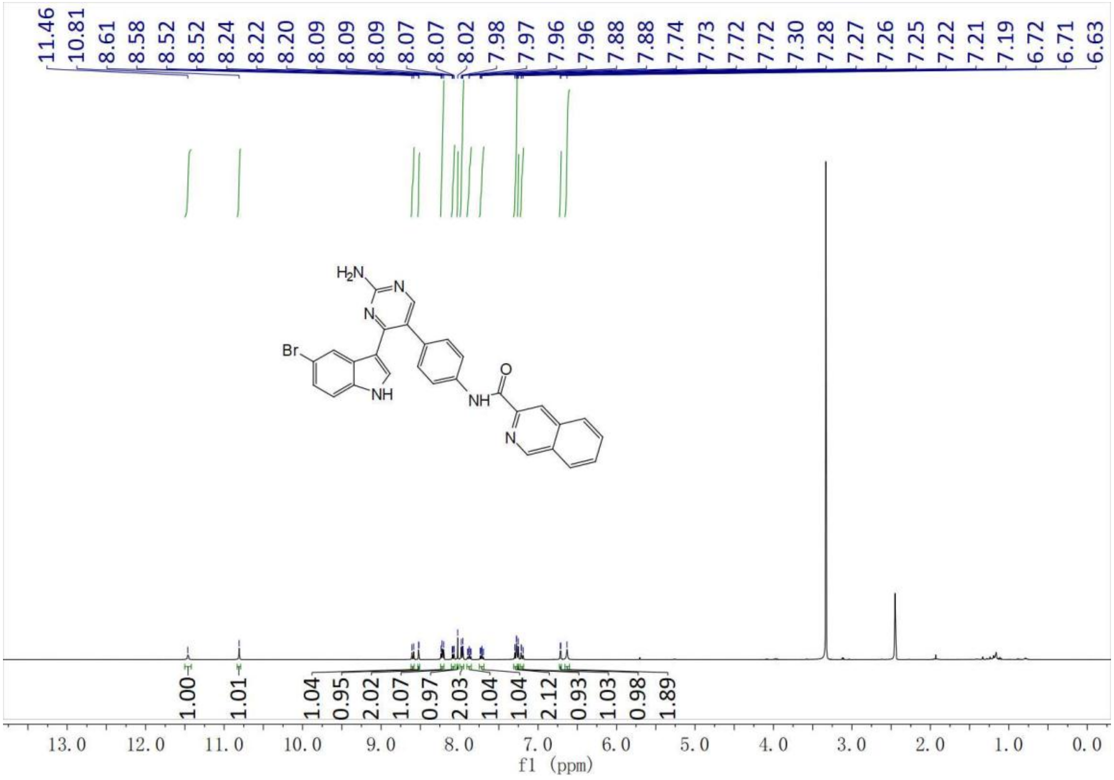

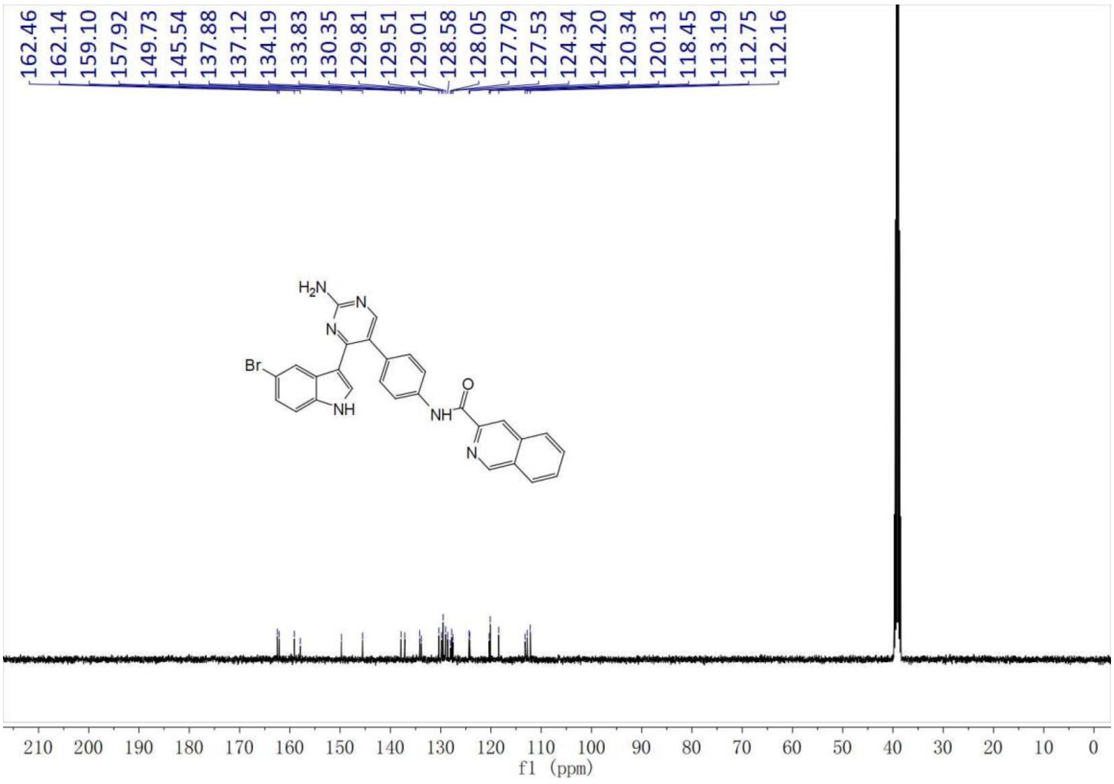

Display Report

**Analysis Info**		Acquisition Date	10/10/2020 16:32:52 PM
Analysis Name	D:\Data\data\2020\TC44_RE1_01_4214.d		
Method	MS-2MIN-POS.m	Operator	BDAL@DE
Sample Name	TC44	Instrument	compact 8255754.20127
Comment			

Acquisition Parameter

Source Type	ESI	Ion Polarity	Positive	Set Nebulizer	2.0 Bar
Focus	Active	Set Capillary	4500 V	Set Dry Heater	200 °C
Scan Begin	50 m/z	Set End Plate Offset	-500 V	Set Dry Gas	8.0 l/min
Scan End	3000 m/z	Set Charging Voltage	2000 V	Set Divert Valve	Waste
		Set Corona	0 nA	Set APCI Heater	0 °C

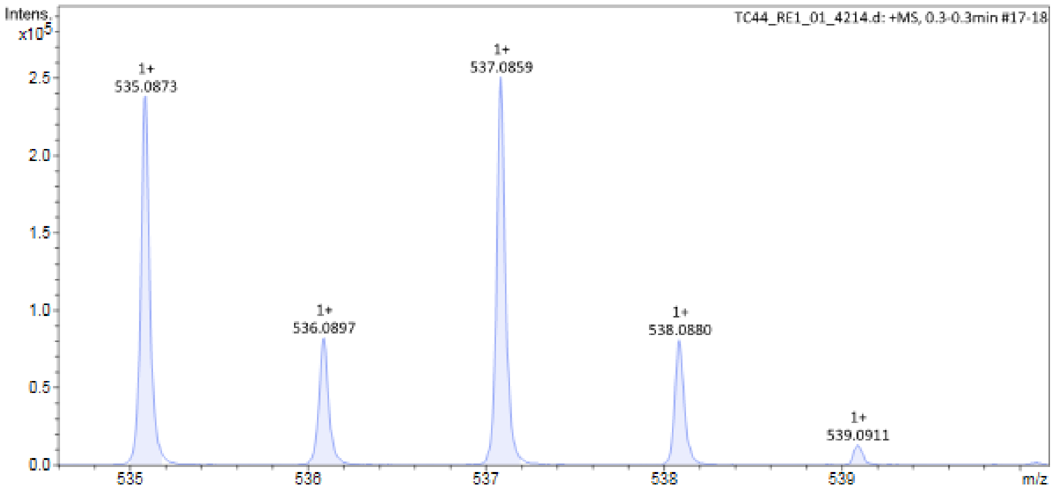

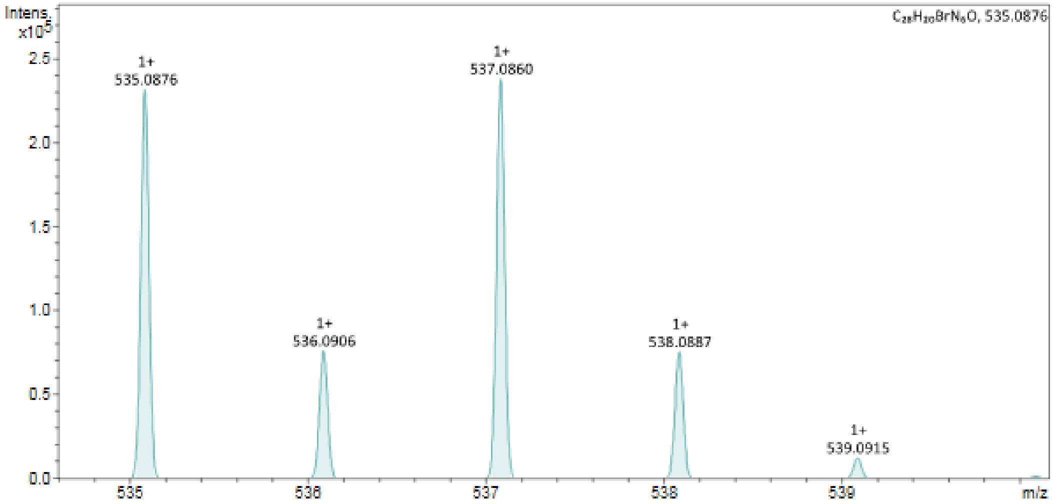

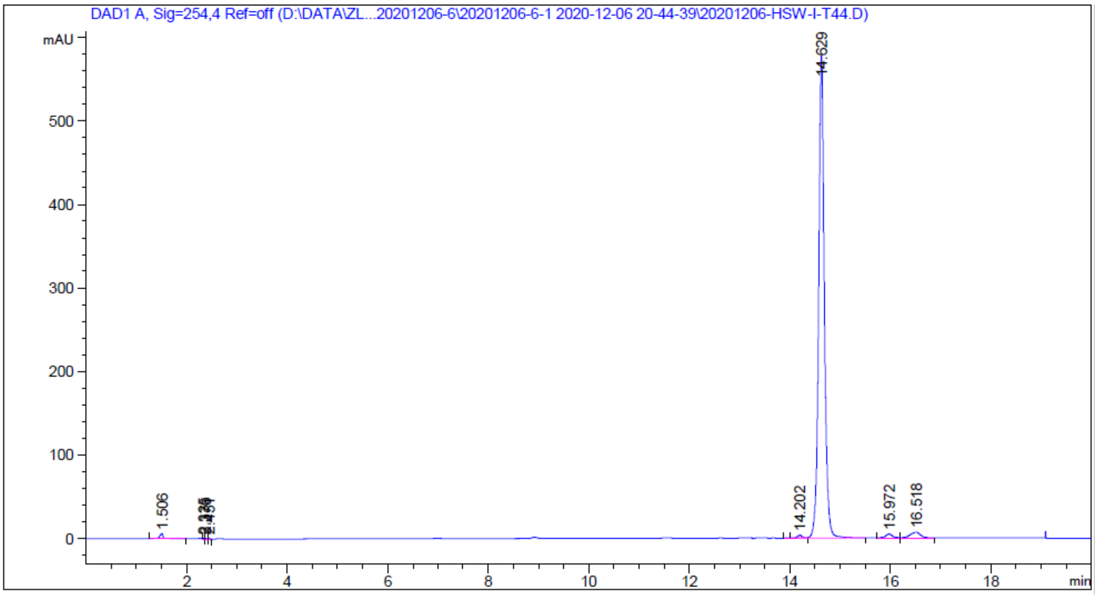

Signal 1: DAD1 A, Sig=254,4 Ref=off

Peak #	RetTime [min]	Type	Width [min]	Area [mAU*s]	Height [mAU]	Area %
1	1.506	VV R	0.0791	30.80748	6.02932	0.6462
2	2.335	VV	0.0379	3.51953	1.14280	0.0738
3	2.376	VV	0.0392	3.30792	1.05047	0.0694
4	2.451	VB	0.0289	2.67496	1.18179	0.0561

1260R 12/7/2020 8:37:39 AM BY

Data File D:\DATA\ZL...TA\20201206-6\20201206-6-1 2020-12-06 20-44-39
Sample Name: 20201206-HSW-I-T44

Peak #	RetTime [min]	Type	Width [min]	Area [mAU*s]	Height [mAU]	Area %
5	14.202	VV E	0.0919	22.16912	3.45250	0.4650
6	14.629	VV R	0.1183	4569.20313	577.66956	95.8434
7	15.972	BV	0.1133	42.67441	4.70379	0.8951
8	16.518	VV R	0.1672	93.00564	6.83928	1.9509

Totals : 4767.36218 602.06950

^{1}H NMR, ^{13}C NMR, HRMS, and HPLC of compound A29

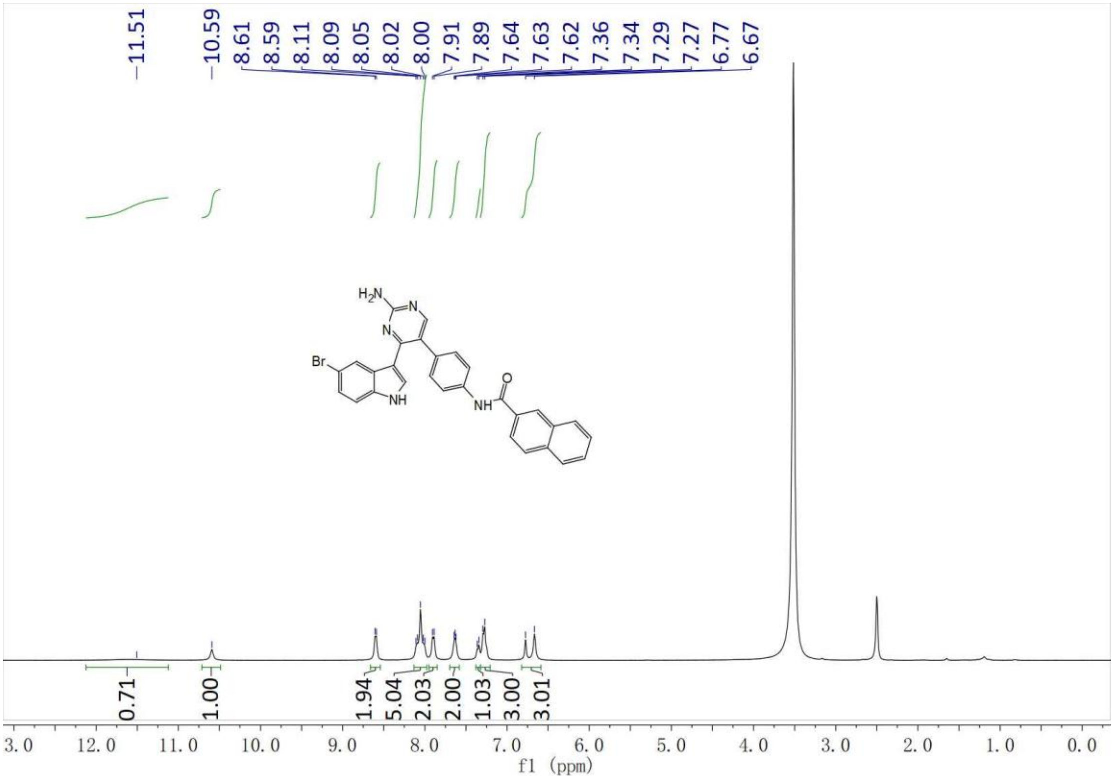

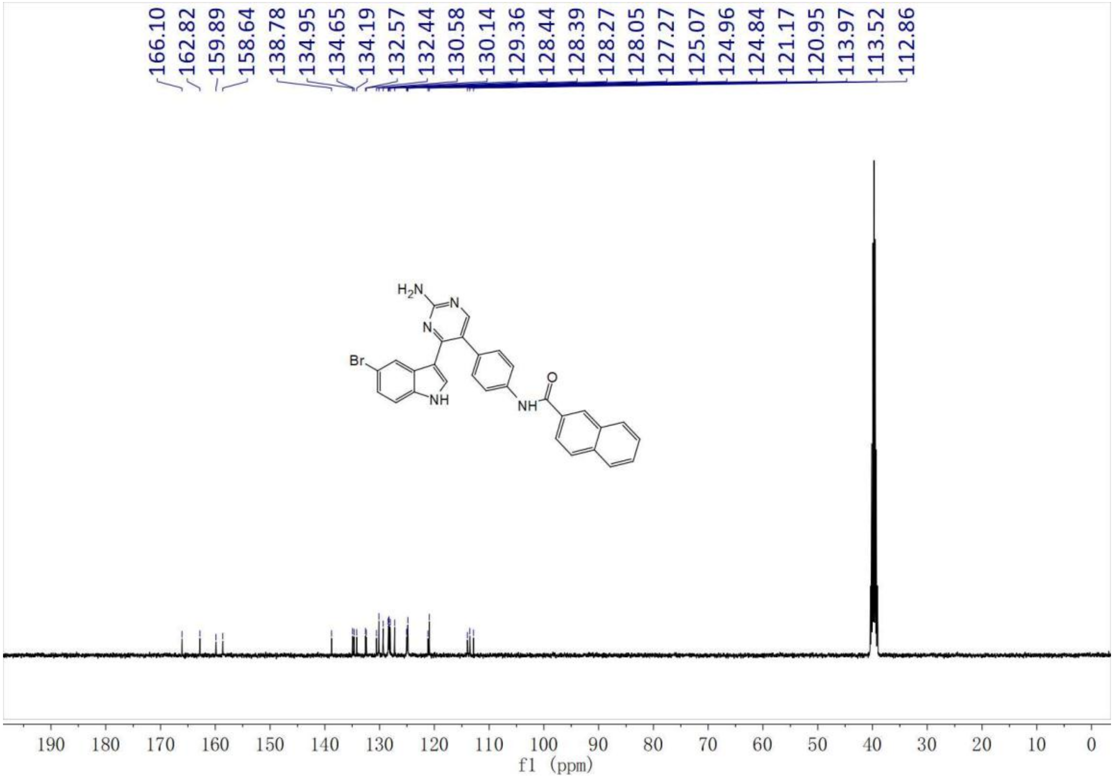

Display Report

Analysis Info

Analysis Name	D:\Data\data\2020\TC47_RE2_01_4215.d	Acquisition Date	10/10/2020 16:35:38 PM
Method	MS-2MIN-POS.m	Operator	BDAL@DE
Sample Name	TC47	Instrument	compact 8255754.20127
Comment			

Acquisition Parameter

Source Type	ESI	Ion Polarity	Positive	Set Nebulizer	2.0 Bar
Focus	Active	Set Capillary	4500 V	Set Dry Heater	200 °C
Scan Begin	50 m/z	Set End Plate Offset	-500 V	Set Dry Gas	8.0 l/min
Scan End	3000 m/z	Set Charging Voltage	2000 V	Set Divert Valve	Waste
		Set Corona	0 nA	Set APCI Heater	0 °C

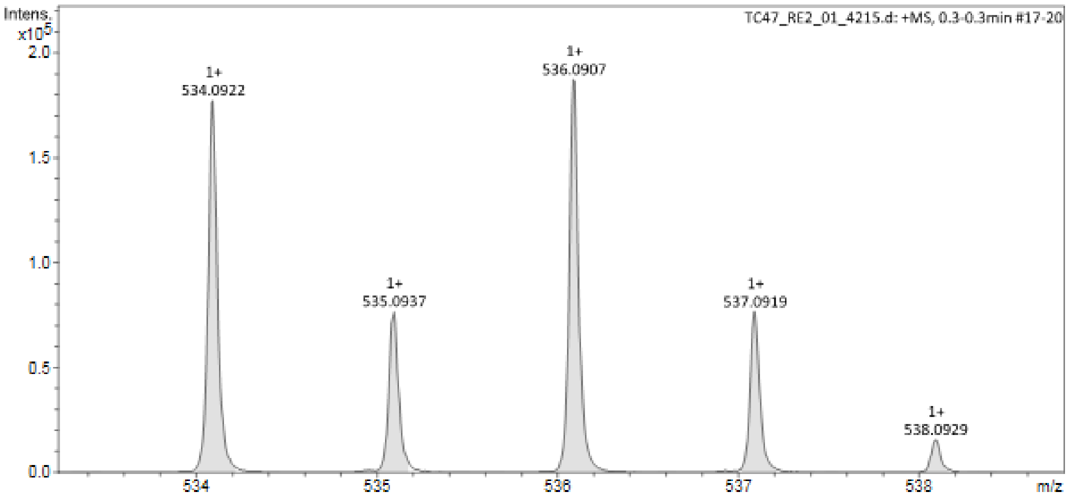

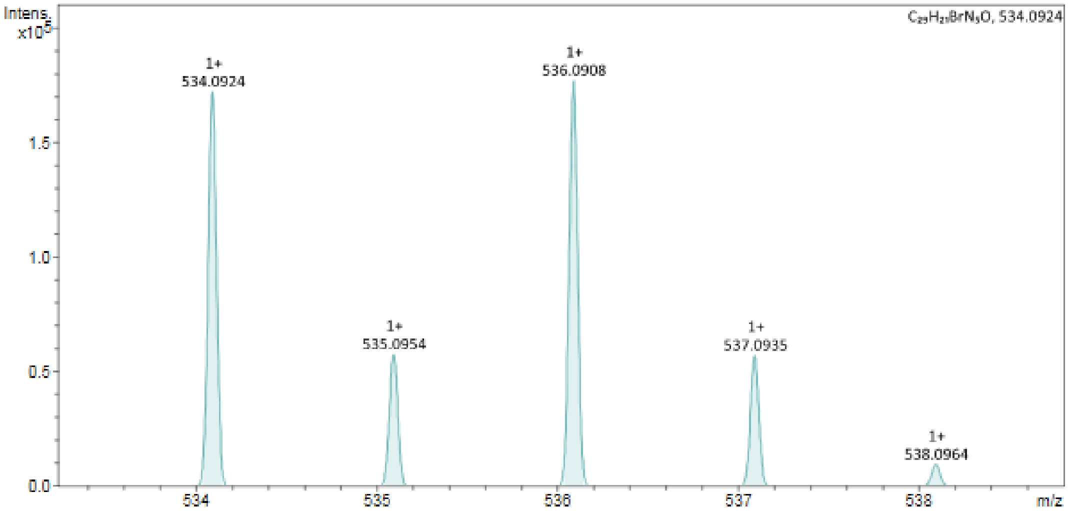

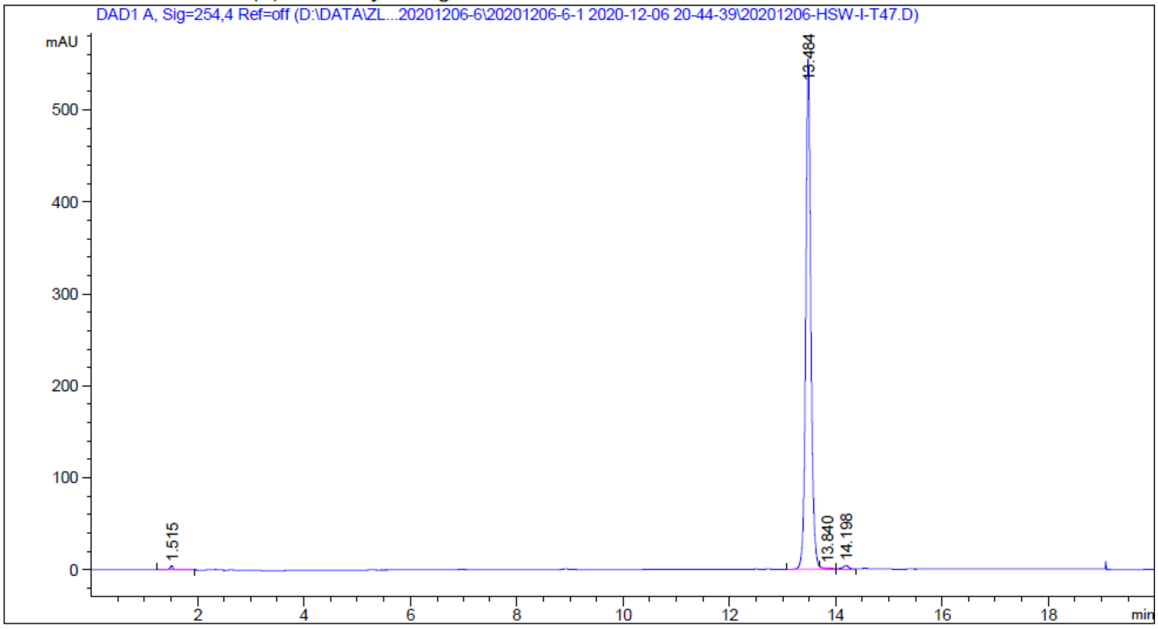

Signal 1: DAD1 A, Sig=254,4 Ref=off

Peak #	RetTime [min]	Type	Width [min]	Area [mAU*s]	Height [mAU]	Area %
1	1.515	VV R	0.0676	21.91526	4.55727	0.5826
2	13.484	BV R	0.0999	3704.17041	554.93536	98.4667
3	13.840	VB E	0.1042	9.83725	1.14483	0.2615
4	14.198	BB	0.0960	25.92748	3.78273	0.6892

1260R 12/7/2020 8:39:28 AM BY

Data File D:\DATA\ZL...TA\20201206-6\20201206-6-1 2020-12-06 20-44-39\202012(
Sample Name: 20201206-HSW-I-T47

Peak #	RetTime [min]	Type	Width [min]	Area [mAU*s]	Height [mAU]	Area %
Totals :				3761.85040	564.42020	

^{1}H NMR, ^{13}C NMR, HRMS, and HPLC of compound B1

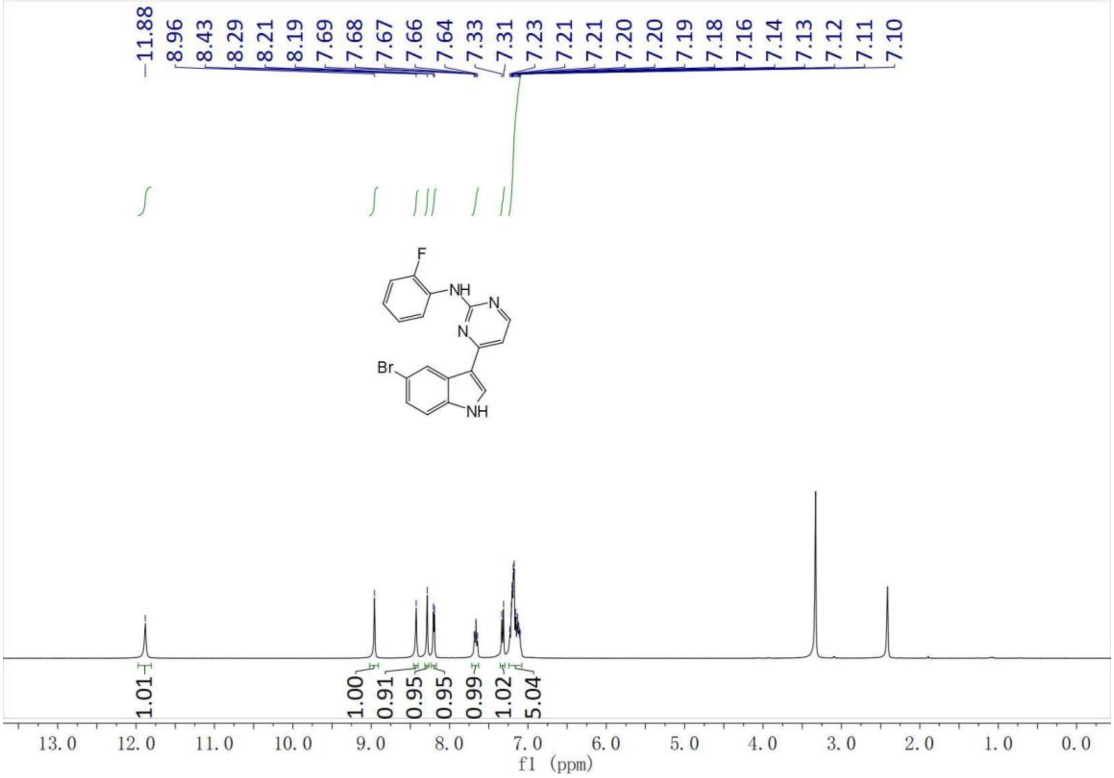

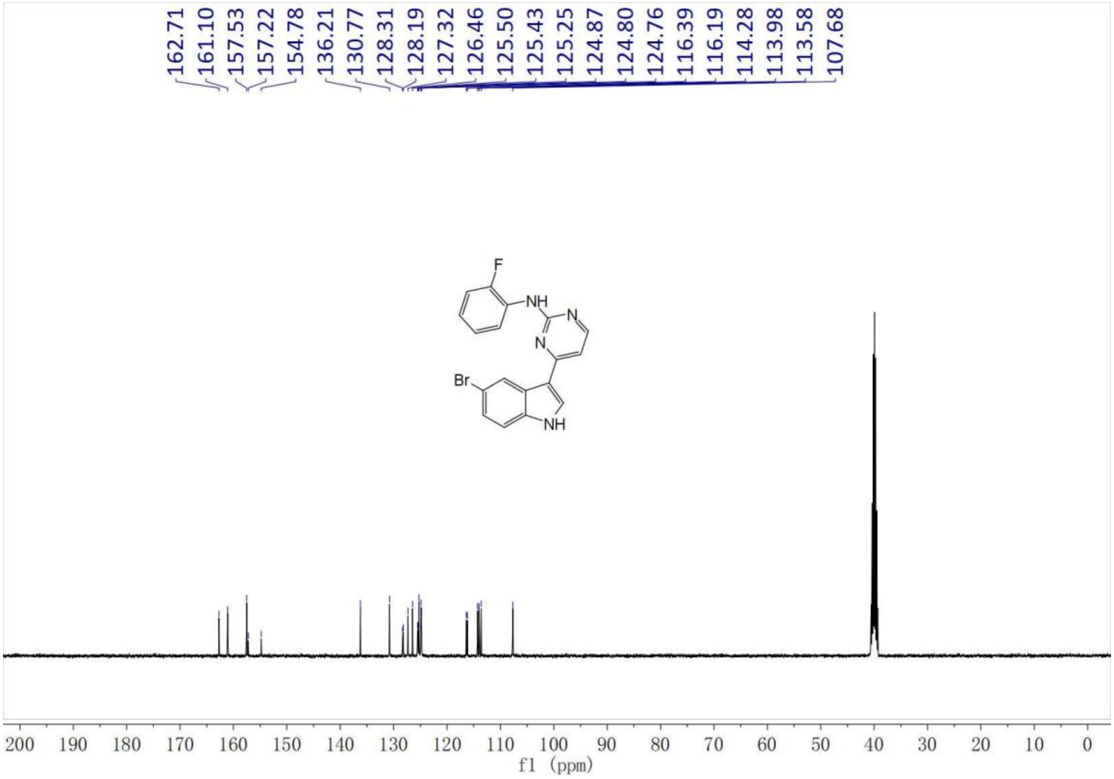

Display Report

Analysis Info

Acquisition Date 12/10/2020 16:59:05 PM

Sample Name TC12
Comment

Acquisition Parameter

Source Type	ESI	Ion Polarity	Positive	Set Nebulizer	2.0 Bar
Focus	Active	Set Capillary	4500 V	Set Dry Heater	200 °C
Scan Begin	50 m/z	Set End Plate Offset	-500 V	Set Dry Gas	8.0 l/min
Scan End	3000 m/z	Set Charging Voltage	2000 V	Set Divert Valve	Waste
		Set Corona	0 nA	Set APCI Heater	0 °C

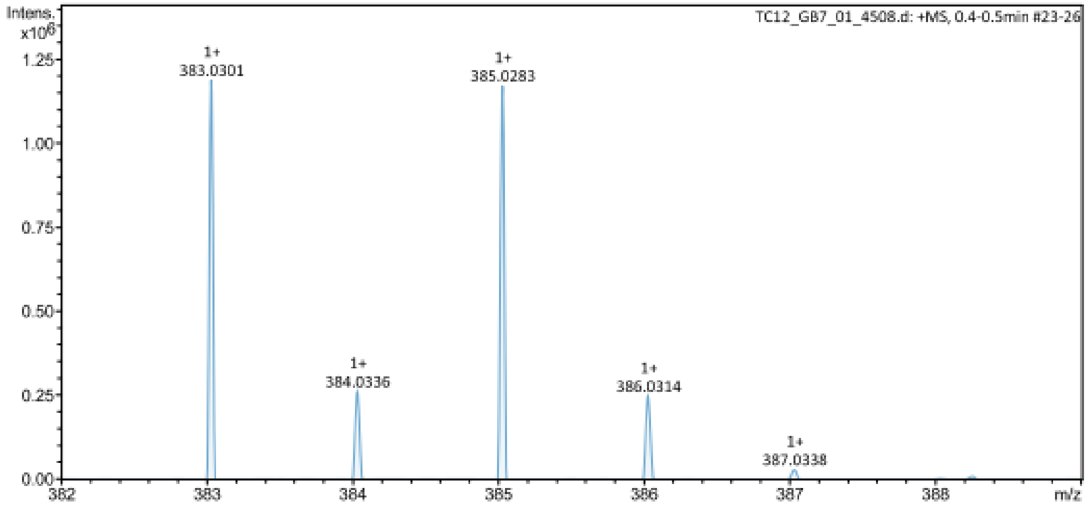

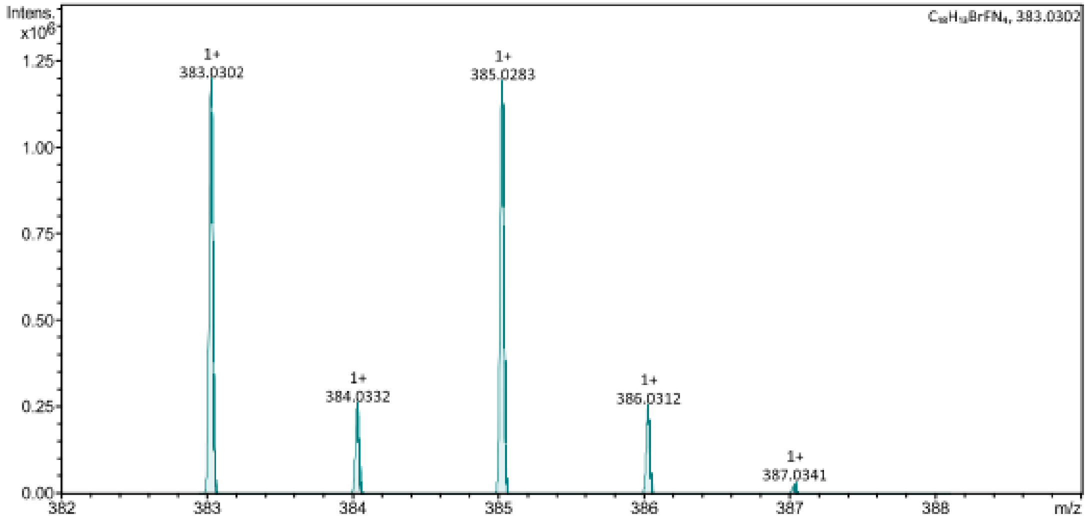

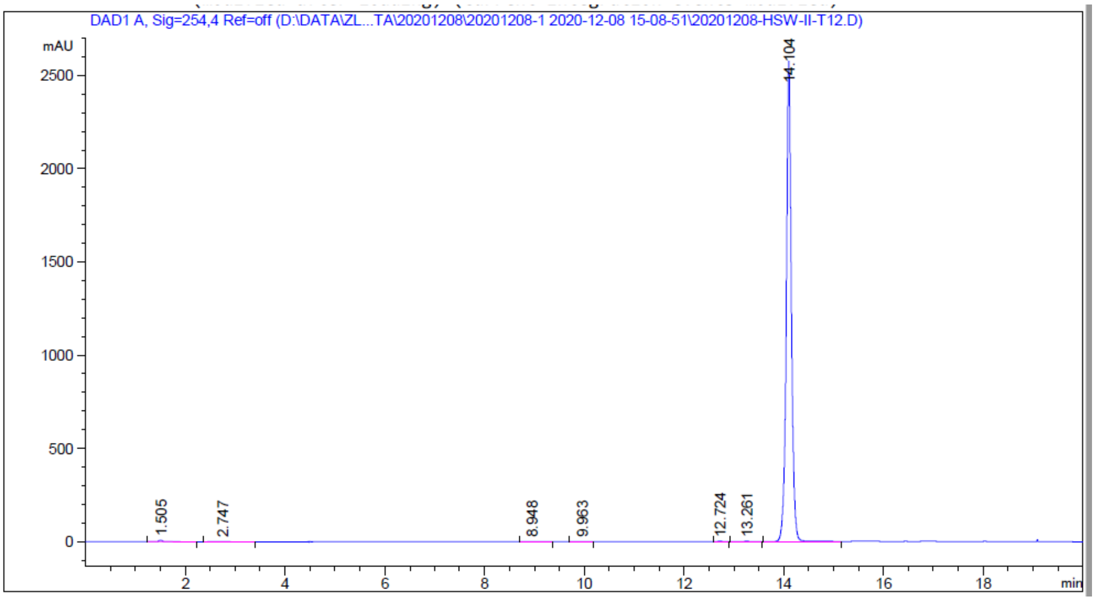

Signal 1: DAD1 A, Sig=254,4 Ref=off

Peak #	RetTime [min]	Type	Width [min]	Area [mAU*s]	Height [mAU]	Area %
1	1.505	BB	0.1069	44.30836	7.72276	0.2412
2	2.747	BB	0.2974	34.69524	1.62331	0.1888
3	8.948	BB	0.1393	13.57609	1.59468	0.0739
4	9.963	BB	0.1166	10.38807	1.58139	0.0565
5	12.724	BB	0.1093	12.40058	2.08750	0.0675

1260R 12/8/2020 8:59:36 PM BY

Data File D:\DATA\ZLM\DATA\20201208\20201208-1 2020-12-08 15-08-51\2020
Sample Name: 20201208-HSW-II-T12

Peak #	RetTime [min]	Type	Width [min]	Area [mAU*s]	Height [mAU]	Area %
6	13.261	BB	0.1429	14.82494	1.68138	0.0807
7	14.104	BB	0.1251	1.82422e4	2501.68799	99.2914

Totals : 1.83723e4 2517.97900

^{1}H NMR, ^{13}C NMR, HRMS, and HPLC of compound B2

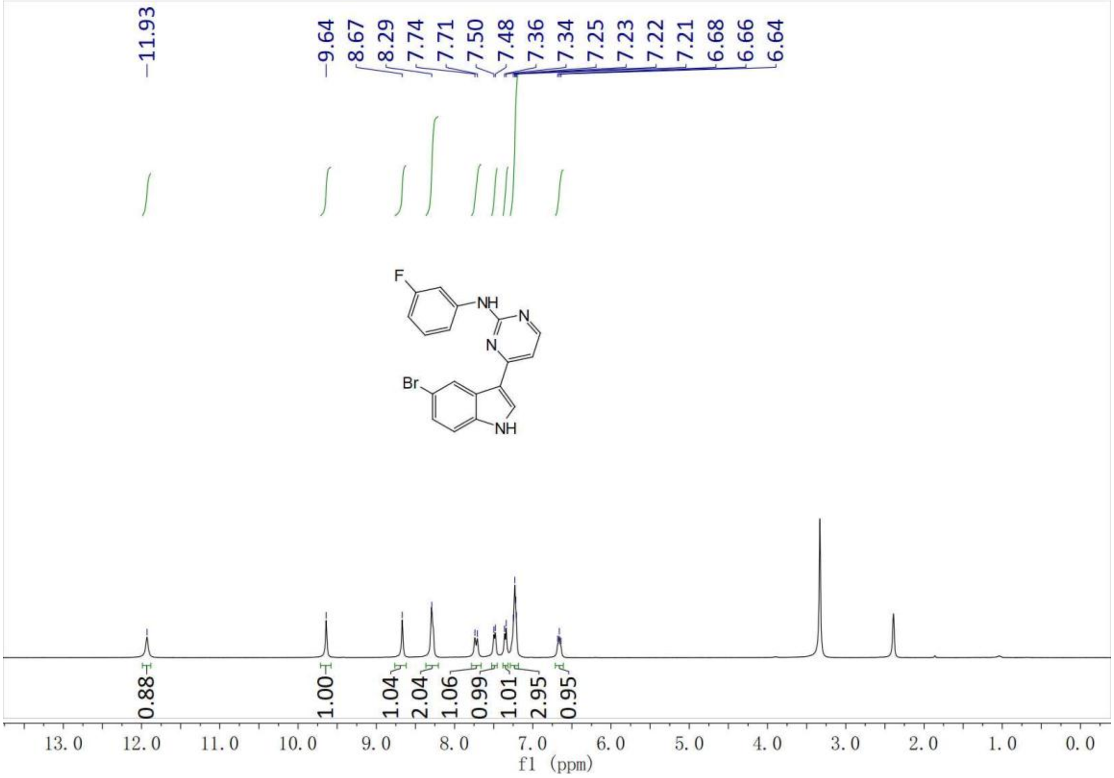

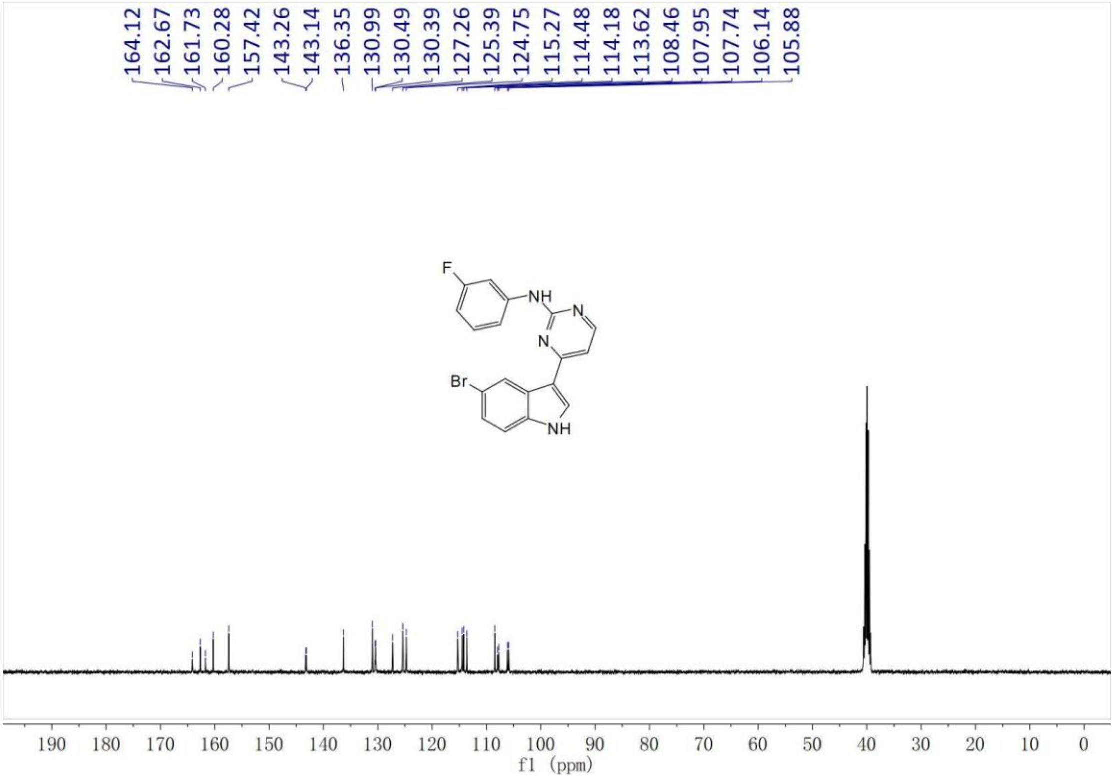

Display Report

Analysis Info | Acquisition Date 12/10/2020 17:07:46 PM

Sample Name TC15
Comment

Acquisition Parameter

Source Type	ESI	Ion Polarity	Positive	Set Nebulizer	2.0 Bar
Focus	Active	Set Capillary	4500 V	Set Dry Heater	200 °C
Scan Begin	50 m/z	Set End Plate Offset	-500 V	Set Dry Gas	8.0 l/min
Scan End	3000 m/z	Set Charging Voltage	2000 V	Set Divert Valve	Waste
		Set Corona	0 nA	Set APCI Heater	0 °C

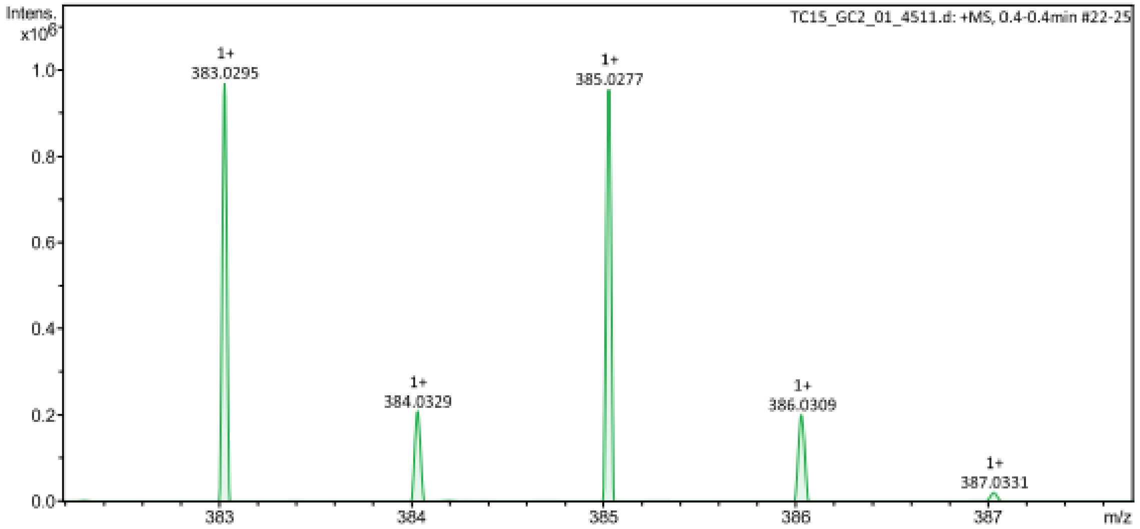

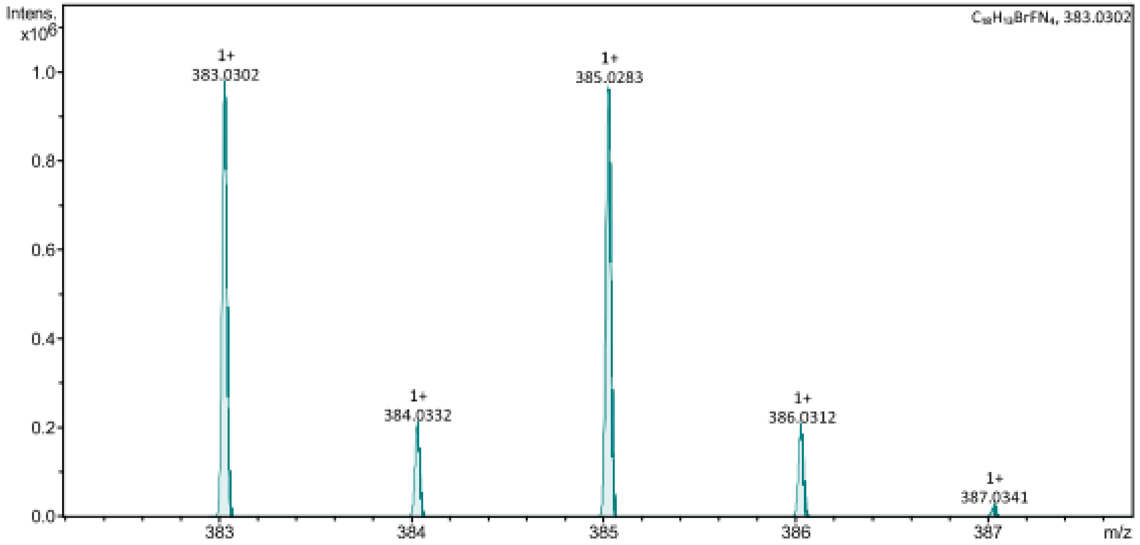

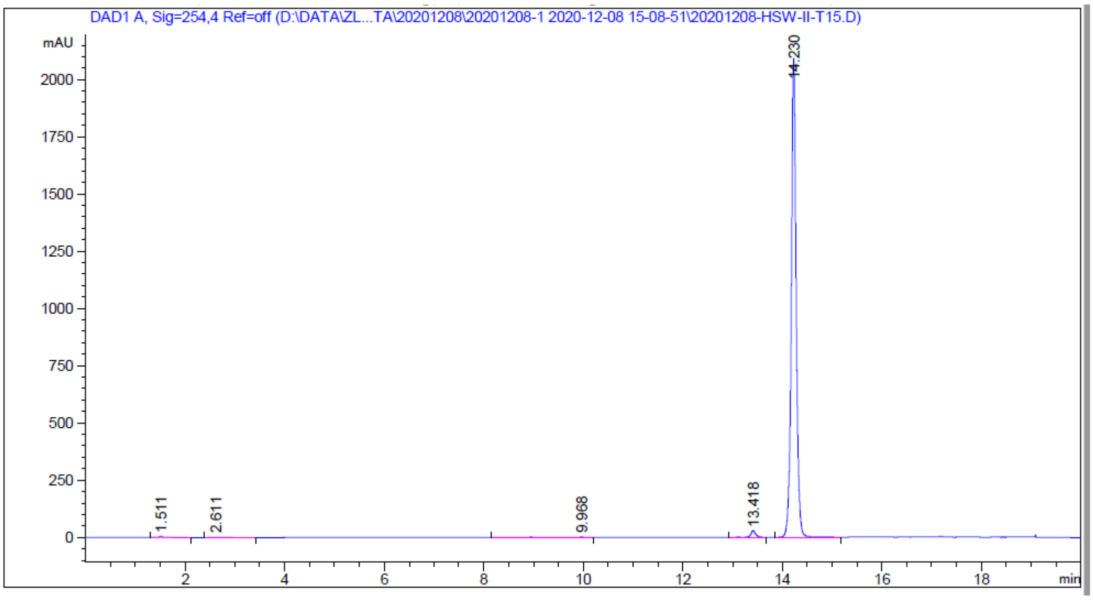

Signal 1: DAD1 A, Sig=254,4 Ref=off

Peak #	RetTime [min]	Type	Width [min]	Area [mAU*s]	Height [mAU]	Area %
1	1.511	BB	0.1025	26.13337	4.87886	0.1813
2	2.611	BB	0.3268	31.95665	1.37689	0.2216
3	9.968	BB	0.2261	25.68444	1.61010	0.1781
4	13.418	BB	0.1220	193.73387	27.57437	1.3437
5	14.230	BB	0.1203	1.41409e4	2053.53345	98.0753

1260R 12/8/2020 9:05:01 PM BY

Data File D:\DATA\ZLM\DATA\20201208\20201208-1 2020-12-08 15-08-51\2020120
Sample Name: 20201208-HSW-II-T15

Peak #	RetTime [min]	Type	Width [min]	Area [mAU*s]	Height [mAU]	Area %
Totals :				1.44184e4	2088.97366	

^{1}H NMR, ^{13}C NMR, HRMS, and HPLC of compound B3

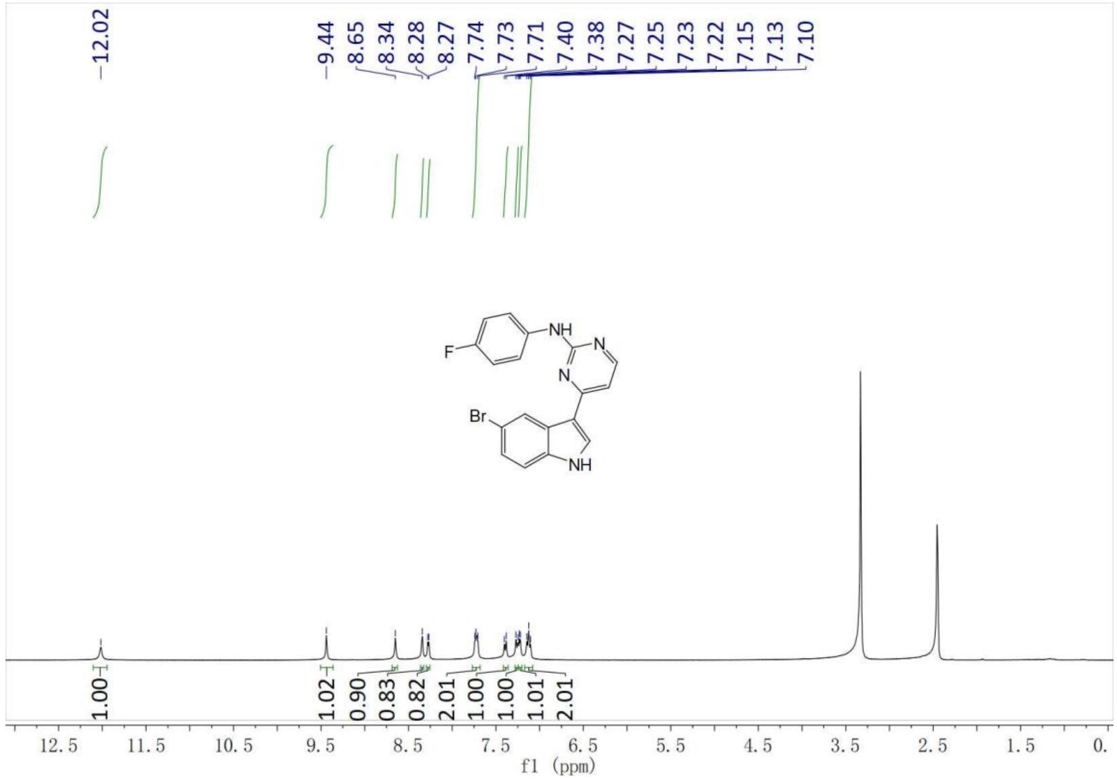

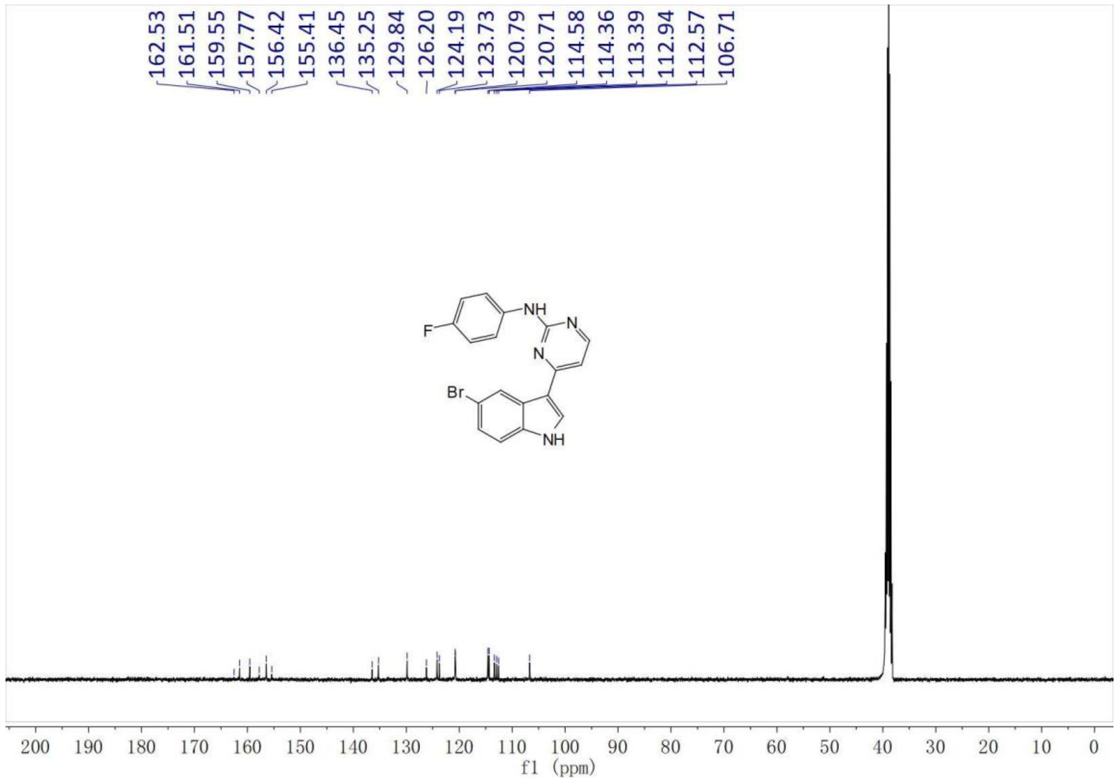

Display Report

Analysis Info

Acquisition Date 12/10/2020 16:42:30 PM

Sample Name TC6
Comment

Acquisition Parameter

Source Type	ESI	Ion Polarity	Positive	Set Nebulizer	2.0 Bar
Focus	Active	Set Capillary	4500 V	Set Dry Heater	200 °C
Scan Begin	50 m/z	Set End Plate Offset	-500 V	Set Dry Gas	8.0 l/min
Scan End	3000 m/z	Set Charging Voltage	2000 V	Set Divert Valve	Waste
		Set Corona	0 nA	Set APCI Heater	0 °C

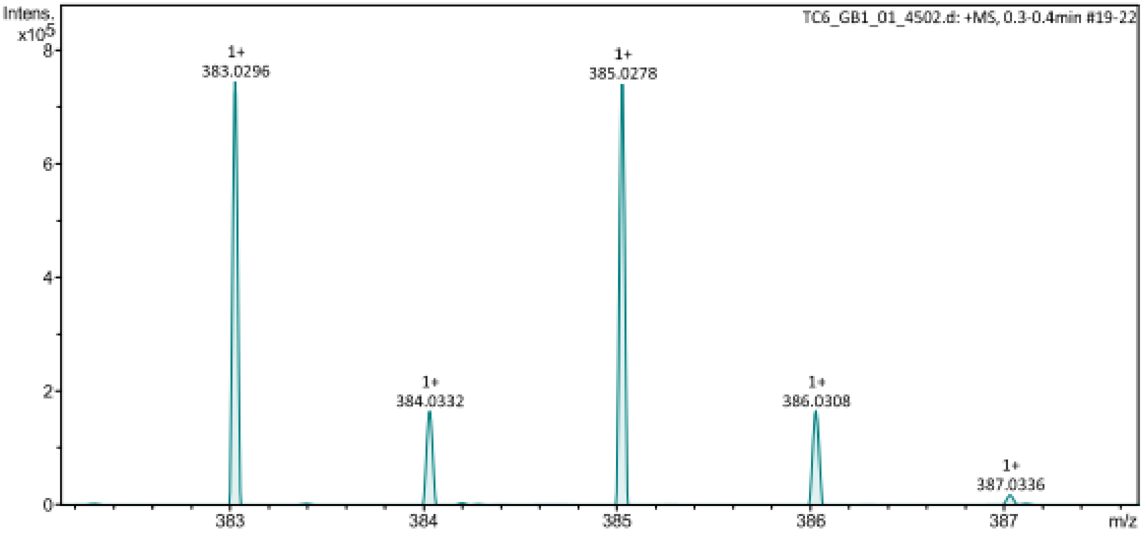

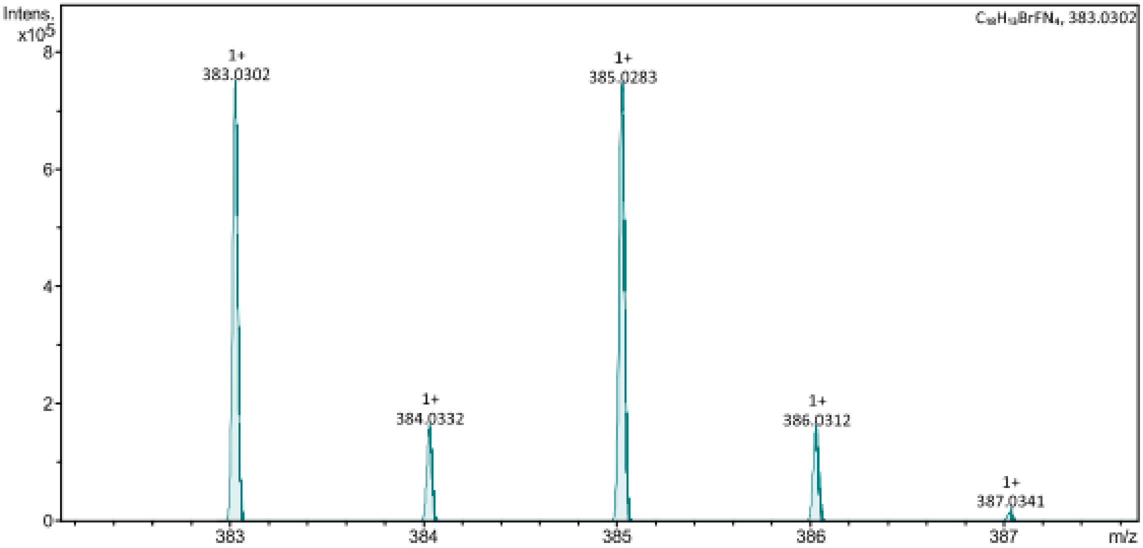

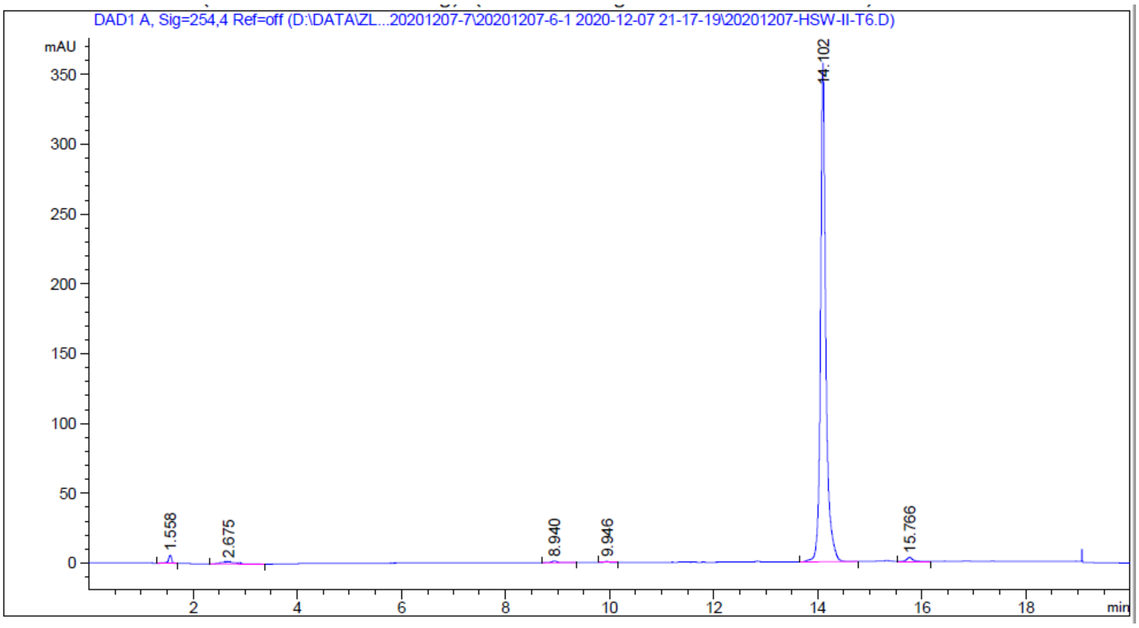

Signal 1: DAD1 A, Sig=254,4 Ref=off

Peak #	RetTime [min]	Type	Width [min]	Area [mAU*s]	Height [mAU]	Area %
1	1.558	BB	0.0737	25.87304	5.61989	0.9786
2	2.675	BB	0.3025	35.42937	1.59927	1.3400
3	8.940	BB	0.1292	9.80566	1.13045	0.3709
4	9.946	BB	0.0973	4.27235	6.80241e-1	0.1616
5	14.102	BB	0.1113	2544.69653	356.18967	96.2469

1260R 12/8/2020 2:17:30 PM BY

Data File D:\DATA\ZL...TA\20201207-7\20201207-6-1 2020-12-07 21-17-19\20
Sample Name: 20201207-HSW-II-T6

Peak #	RetTime [min]	Type	Width [min]	Area [mAU*s]	Height [mAU]	Area %
6	15.766	BB	0.1222	23.84744	2.95228	0.9020

Totals : 2643.92439 368.17180

^{1}H NMR, ^{13}C NMR, HRMS, and HPLC of compound B4

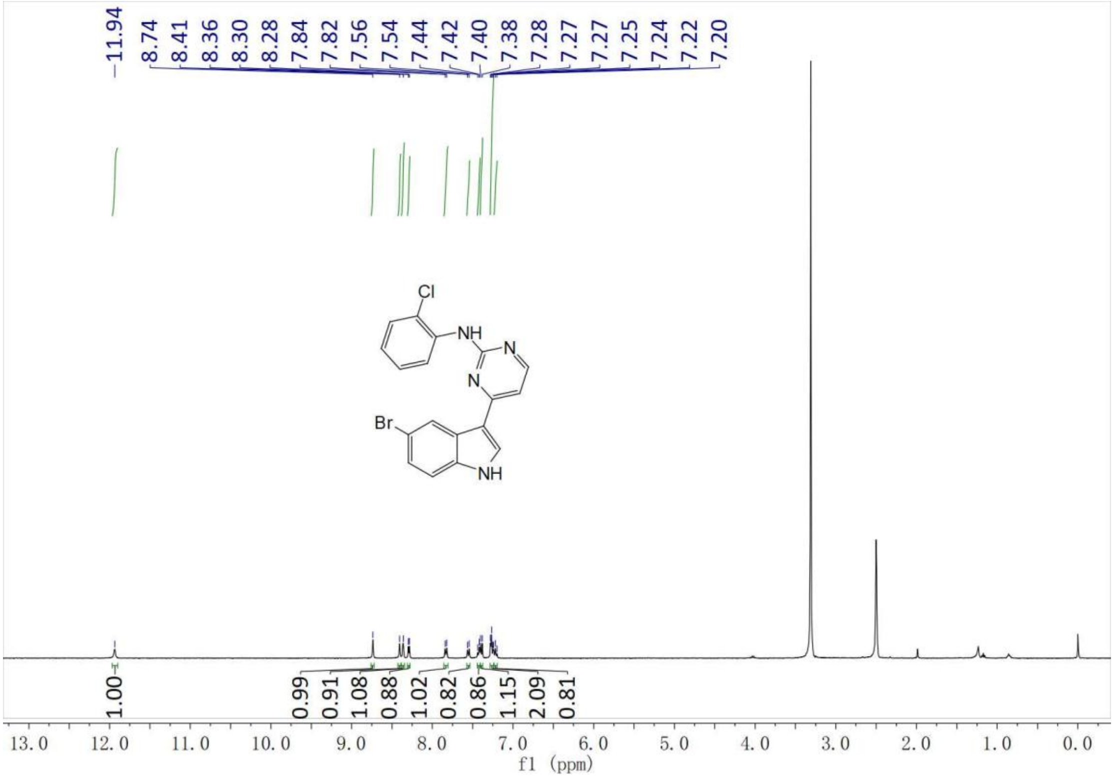

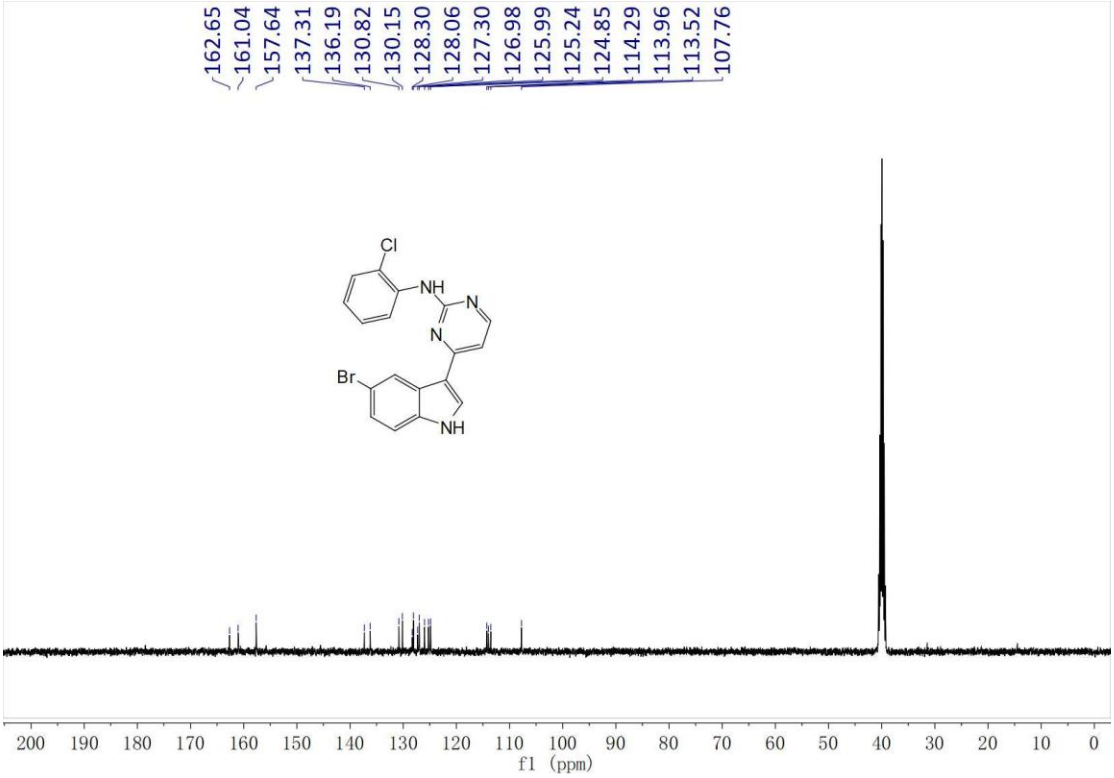

Display Report

Analysis Info

Acquisition Date 12/10/2020 17:02:15 PM

Sample Name TC13
Comment

Acquisition Parameter

Source Type	ESI	Ion Polarity	Positive	Set Nebulizer	2.0 Bar
Focus	Active	Set Capillary	4500 V	Set Dry Heater	200 °C
Scan Begin	50 m/z	Set End Plate Offset	-500 V	Set Dry Gas	8.0 l/min
Scan End	3000 m/z	Set Charging Voltage	2000 V	Set Divert Valve	Waste
		Set Corona	0 nA	Set APCI Heater	0 °C

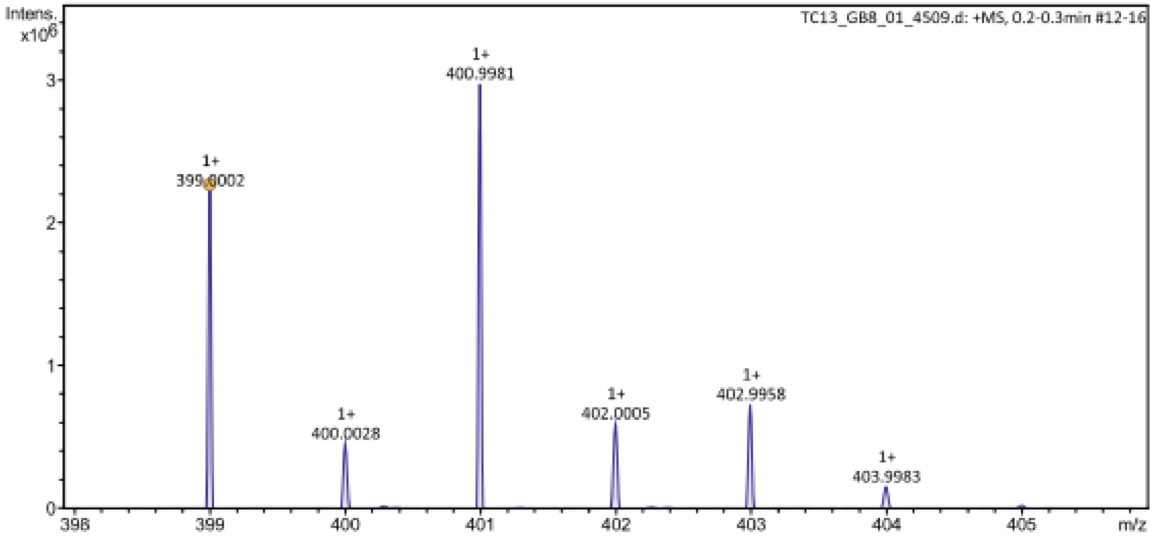

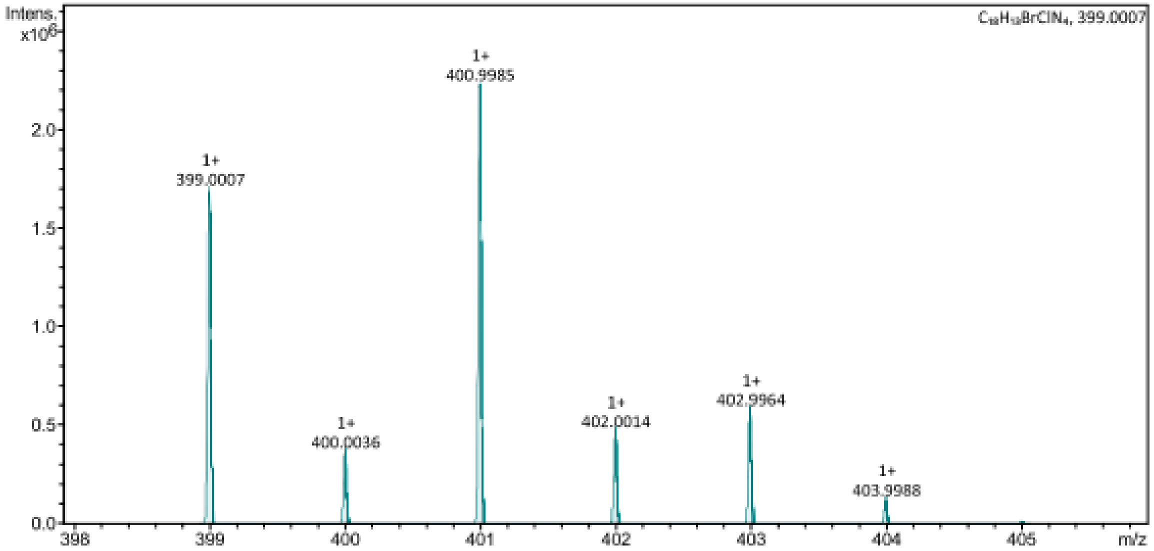

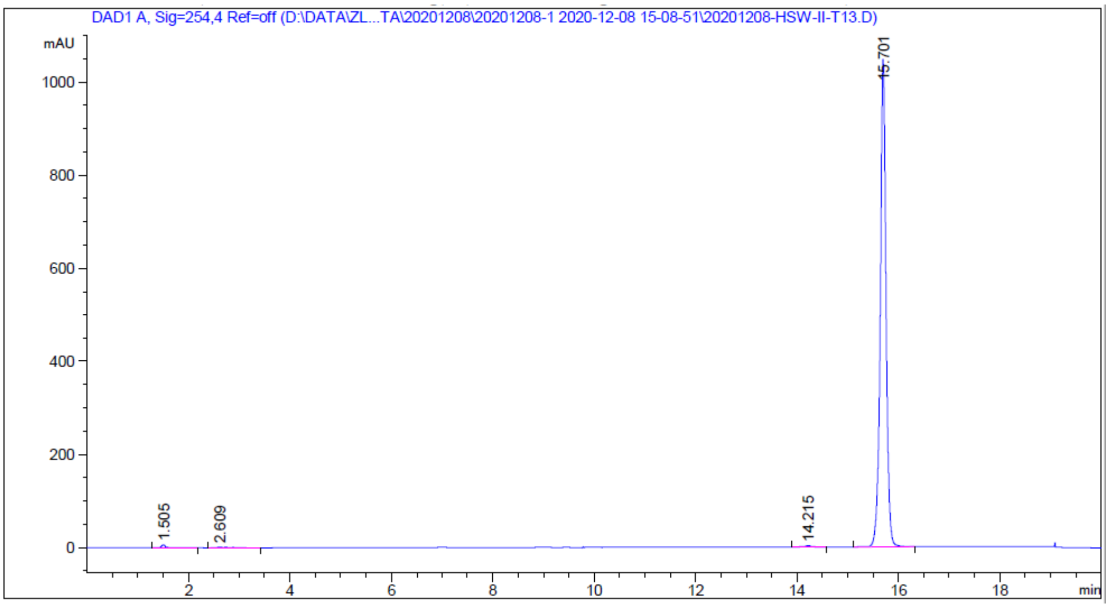

Signal 1: DAD1 A, Sig=254,4 Ref=off

Peak #	RetTime [min]	Type	Width [min]	Area [mAU*s]	Height [mAU]	Area %
1	1.505	BB	0.1013	28.62362	5.44754	0.3432
2	2.609	BB	0.3424	31.23956	1.27248	0.3745
3	14.215	BB	0.1377	25.78764	3.07913	0.3092
4	15.701	BB	0.1337	8255.31348	1027.77759	98.9731

1260R 12/8/2020 9:02:00 PM BY

Data File D:\DATA\ZLM\DATA\20201208\20201208-1 2020-12-08 15-08-51\20
Sample Name: 20201208-HSW-II-T13

Totals : 8340.96430 1037.57674

^{1}H NMR, ^{13}C NMR, HRMS, and HPLC of compound B5

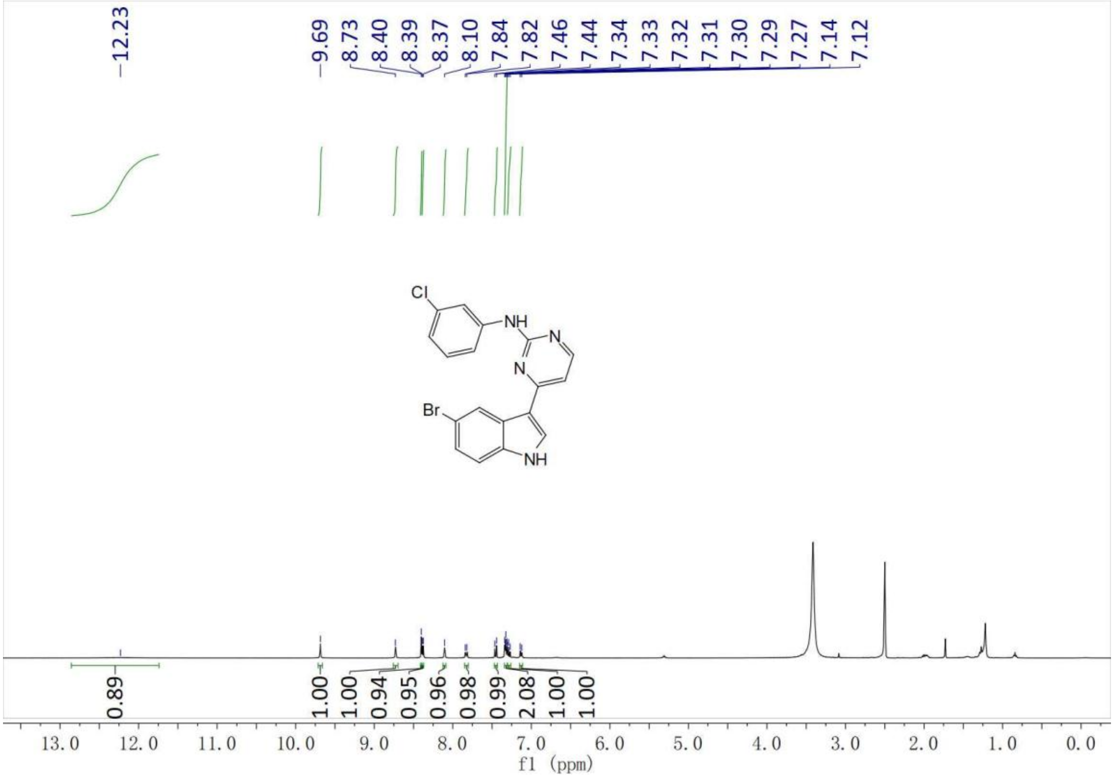

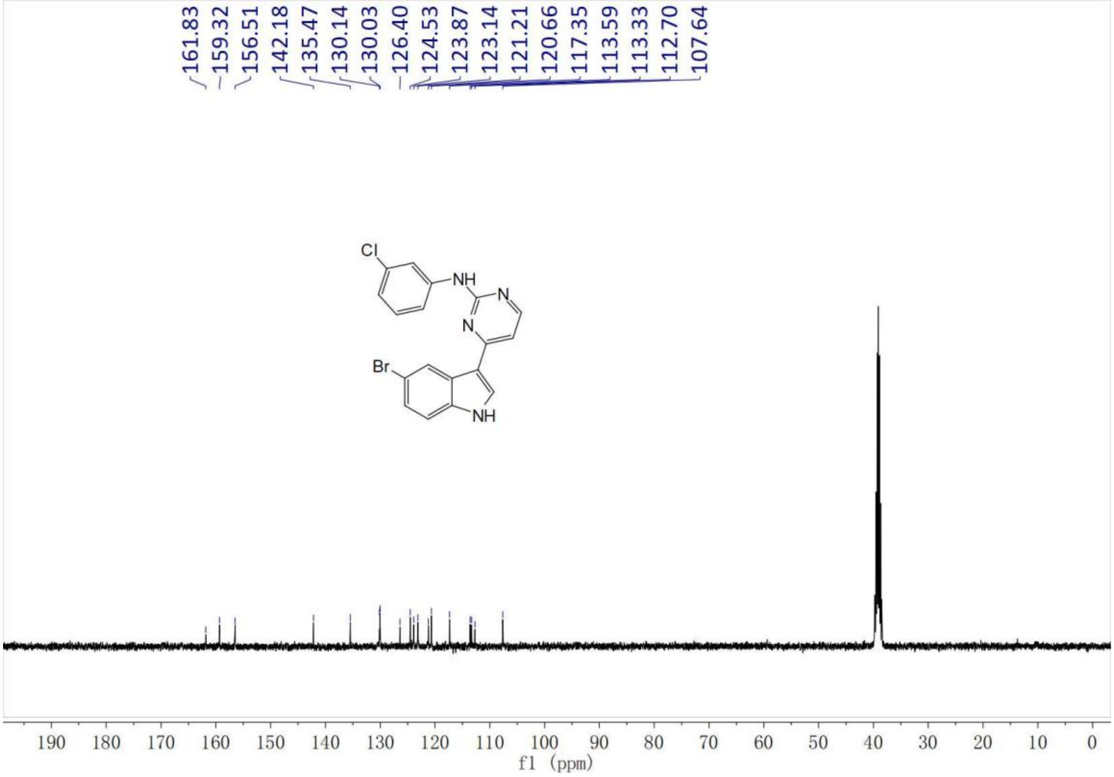

Display Report

Analysis Info

Analysis Name	D:\Data\data\2019\20190711-HSW-TC17_GA3_01_2056.d	Acquisition Date	7/11/2019 17:46:09 PM
Method	MS-2MIN-POS.m	Operator	BDAL@DE
Sample Name	20190711-HSW-TC17	Instrument	compact 8255754.20127
Comment			

Acquisition Parameter

Source Type	ESI	Ion Polarity	Positive	Set Nebulizer	2.0 Bar
Focus	Active	Set Capillary	4500 V	Set Dry Heater	200 °C
Scan Begin	50 m/z	Set End Plate Offset	-500 V	Set Dry Gas	8.0 l/min
Scan End	3000 m/z	Set Charging Voltage	2000 V	Set Divert Valve	Waste
		Set Corona	0 nA	Set APCI Heater	0 °C

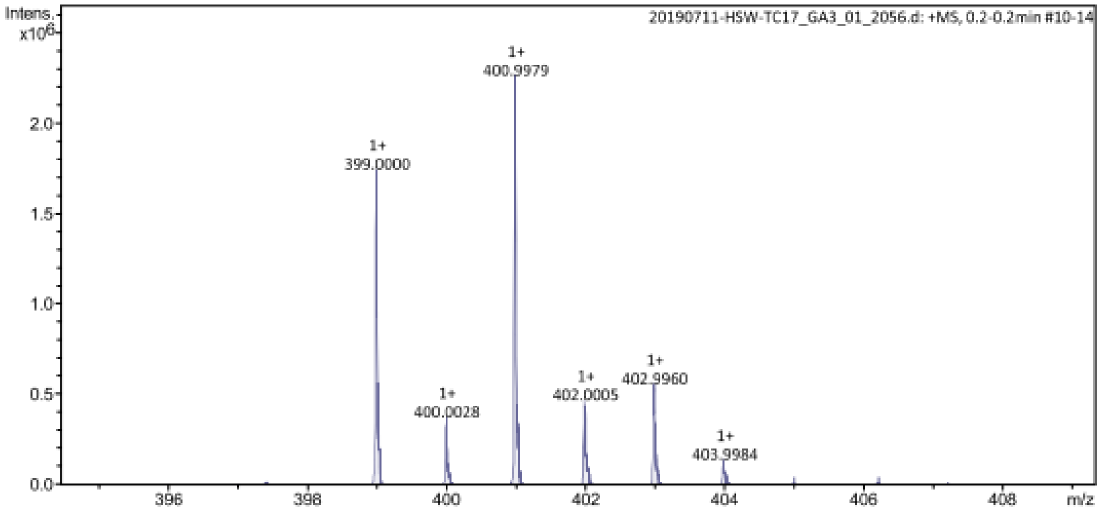

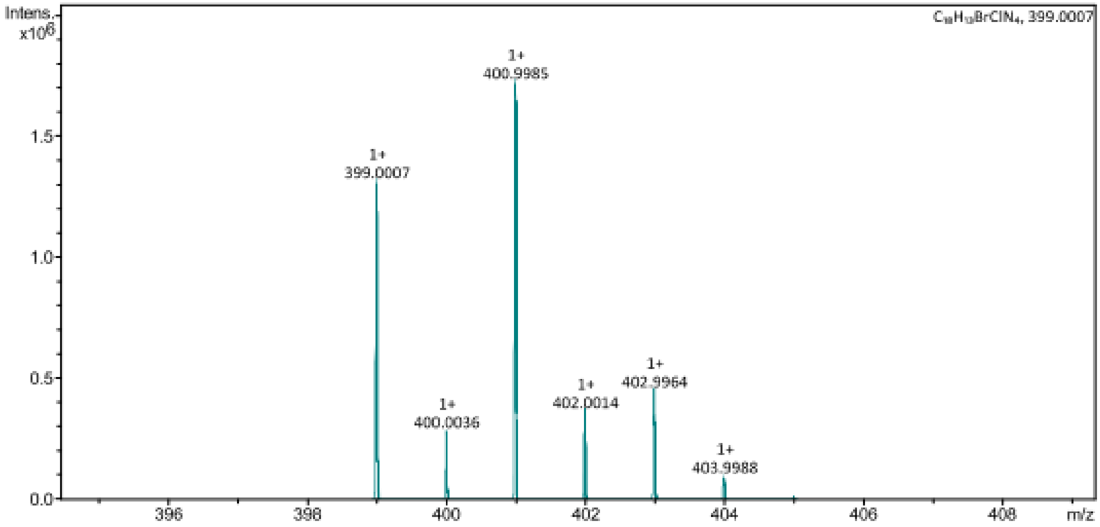

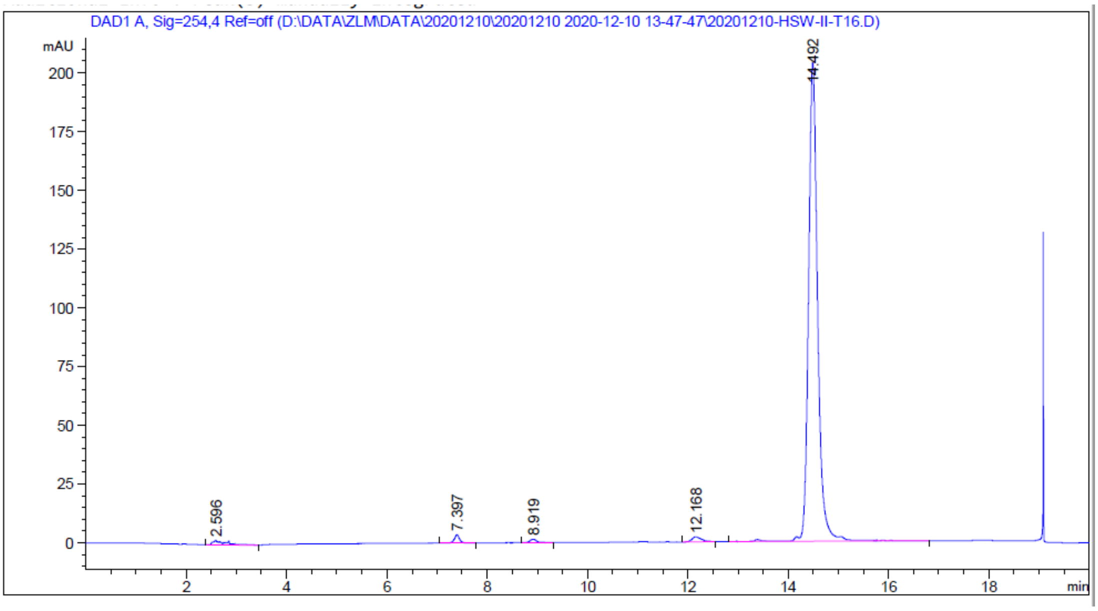

Signal 1: DAD1 A, Sig=254,4 Ref=off

Peak #	RetTime [min]	Type	Width [min]	Area [mAU*s]	Height [mAU]	Area %
1	2.596	BB	0.2900	35.51533	1.71196	1.3270
2	7.397	BB	0.1264	25.53414	3.44676	0.9541
3	8.919	BB	0.1538	14.63989	1.50139	0.5470

1260R 12/23/2020 10:49:05 AM BY

Data File D:\DATA\ZLM\DATA\20201210\20201210 2020-12-10 13-47-47\20201210-HSW-I
Sample Name: 20201210-HSW-II-T16

Peak #	RetTime [min]	Type	Width [min]	Area [mAU*s]	Height [mAU]	Area %
4	12.168	BB	0.2137	27.32267	2.02111	1.0209
5	14.492	BB	0.2042	2573.29150	202.60779	96.1510

Totals : 2676.30353 211.28901

^{1}H NMR, ^{13}C NMR, HRMS, and HPLC of compound B6

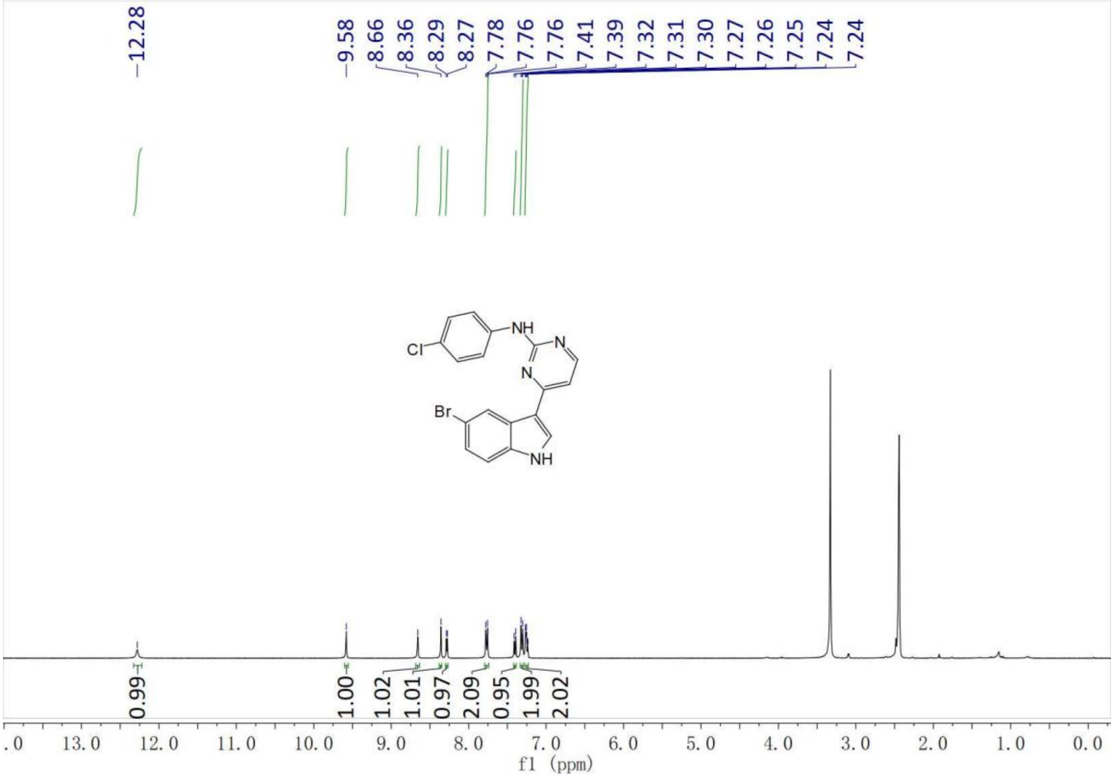

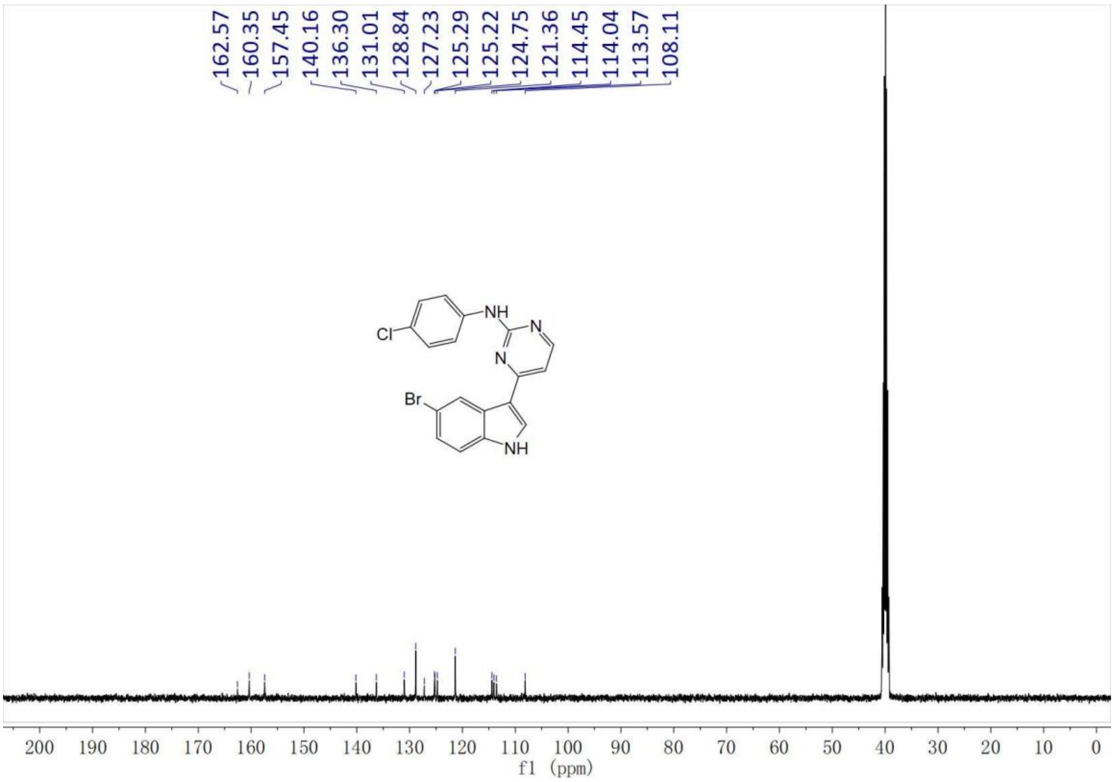

Display Report

Analysis Info

Acquisition Date 12/10/2020 16:31:28 PM

Sample Name TC2
Comment

Acquisition Parameter

Source Type	ESI	Ion Polarity	Positive	Set Nebulizer	2.0 Bar
Focus	Active	Set Capillary	4500 V	Set Dry Heater	200 °C
Scan Begin	50 m/z	Set End Plate Offset	-500 V	Set Dry Gas	8.0 l/min
Scan End	3000 m/z	Set Charging Voltage	2000 V	Set Divert Valve	Waste
		Set Corona	0 nA	Set APCI Heater	0 °C

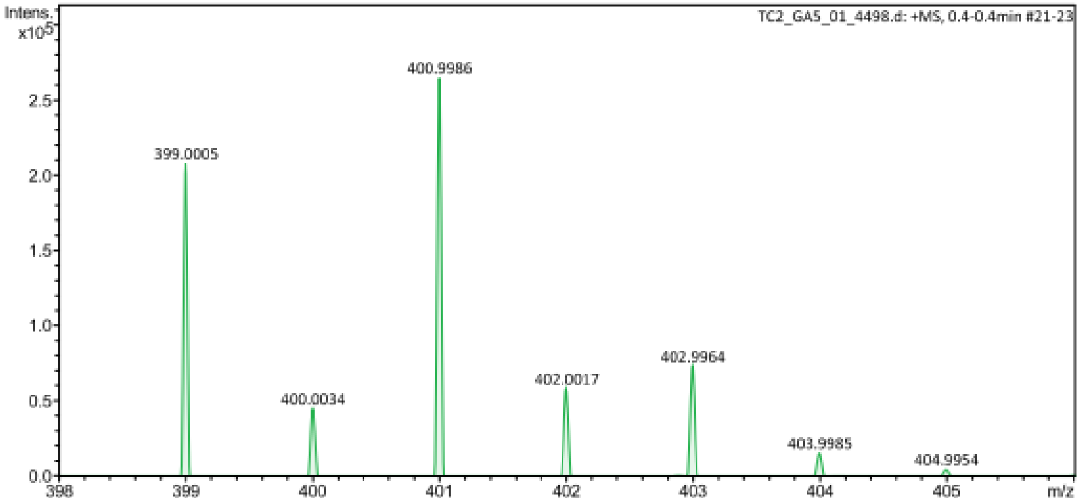

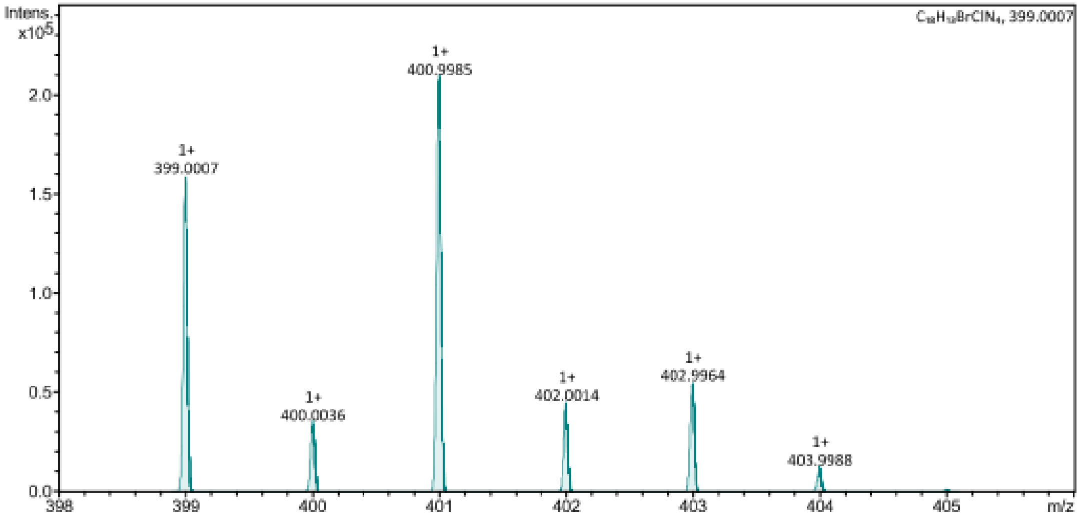

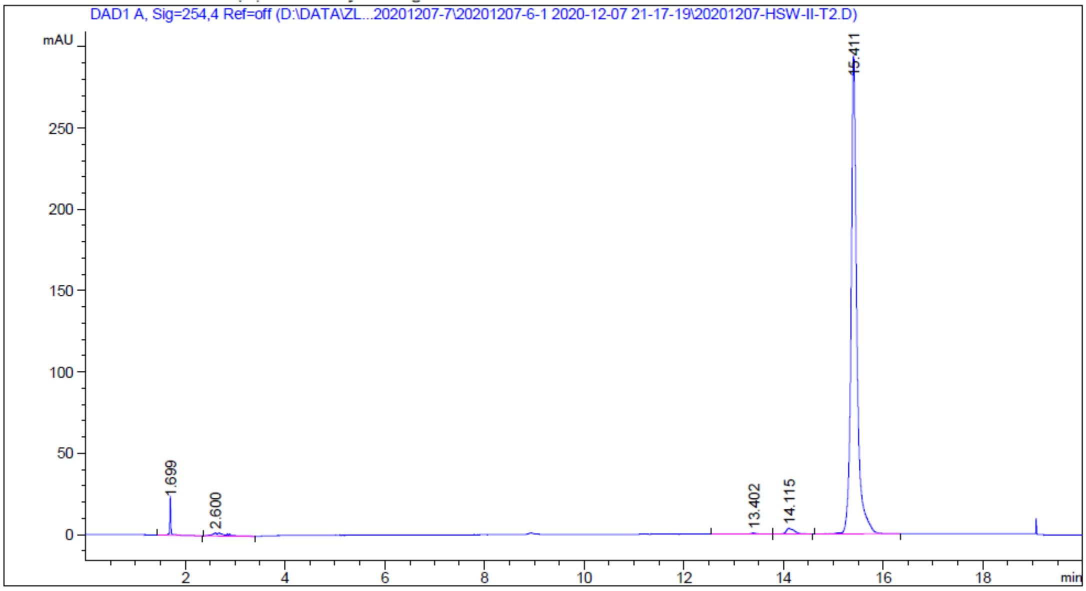

Signal 1: DAD1 A, Sig=254,4 Ref=off

Peak #	RetTime [min]	Type	Width [min]	Area [mAU*s]	Height [mAU]	Area %
1	1.699	BB	0.0406	37.30733	21.09986	1.4899
2	2.600	BB	0.2325	33.44753	1.83616	1.3358
3	13.402	BV	0.1523	8.32623	7.57294e-1	0.3325
4	14.115	VB	0.1569	41.06194	3.60468	1.6399

1260R 12/8/2020 1:47:08 PM BY

Data File D:\DATA\ZL...TA\20201207-7\20201207-6-1 2020-12-07 21-17-19\202
Sample Name: 20201207-HSW-II-T2

Peak #	RetTime [min]	Type	Width [min]	Area [mAU*s]	Height [mAU]	Area %
5	15.411	BB	0.1228	2383.84009	293.40536	95.2019

Totals : 2503.98311 320.70336

^{1}H NMR, ^{13}C NMR, HRMS, and HPLC of compound B7

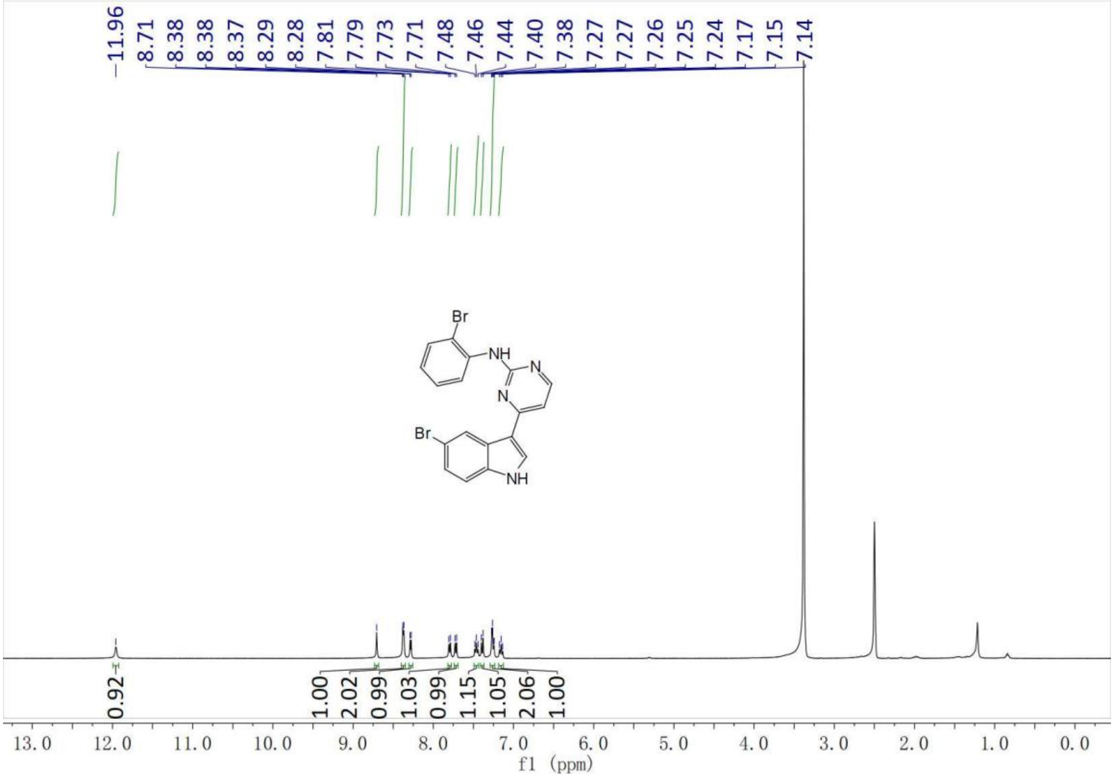

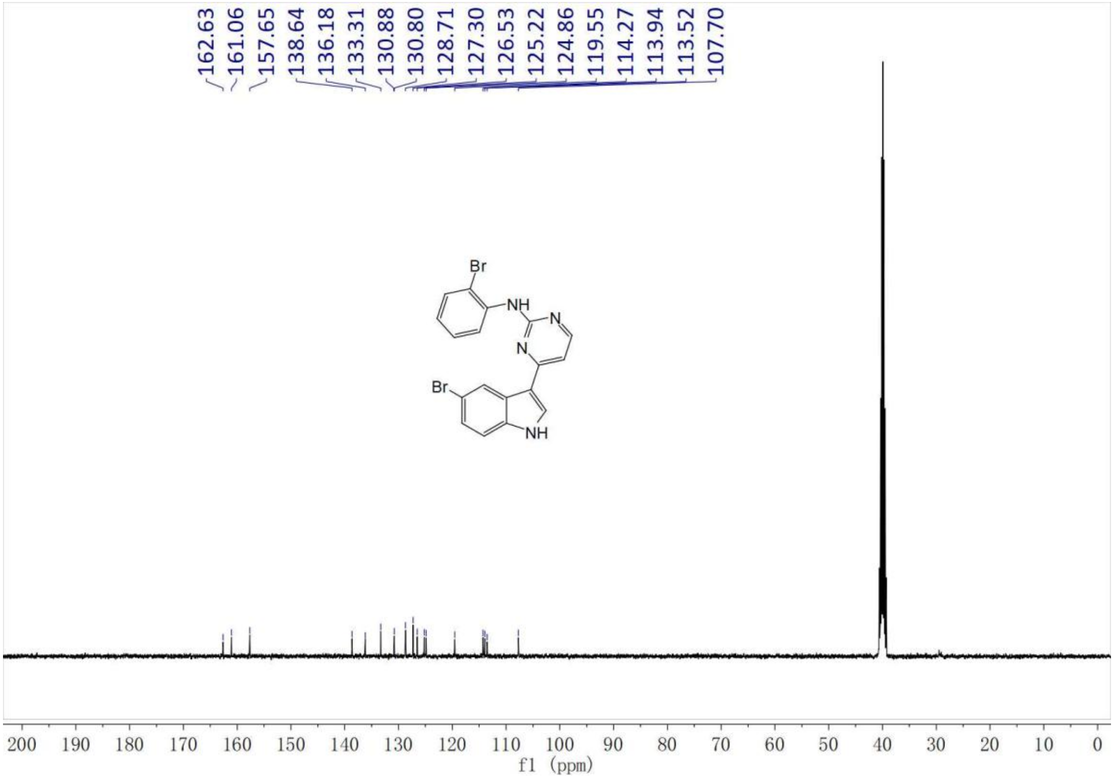

Display Report

Analysis Info Acquisition Date 12/10/2020 17:05:01 PM

Sample Name TC14
Comment

Acquisition Parameter

Source Type	ESI	Ion Polarity	Positive	Set Nebulizer	2.0 Bar
Focus	Active	Set Capillary	4500 V	Set Dry Heater	200 °C
Scan Begin	50 m/z	Set End Plate Offset	-500 V	Set Dry Gas	8.0 l/min
Scan End	3000 m/z	Set Charging Voltage	2000 V	Set Divert Valve	Waste
		Set Corona	0 nA	Set APCI Heater	0 °C

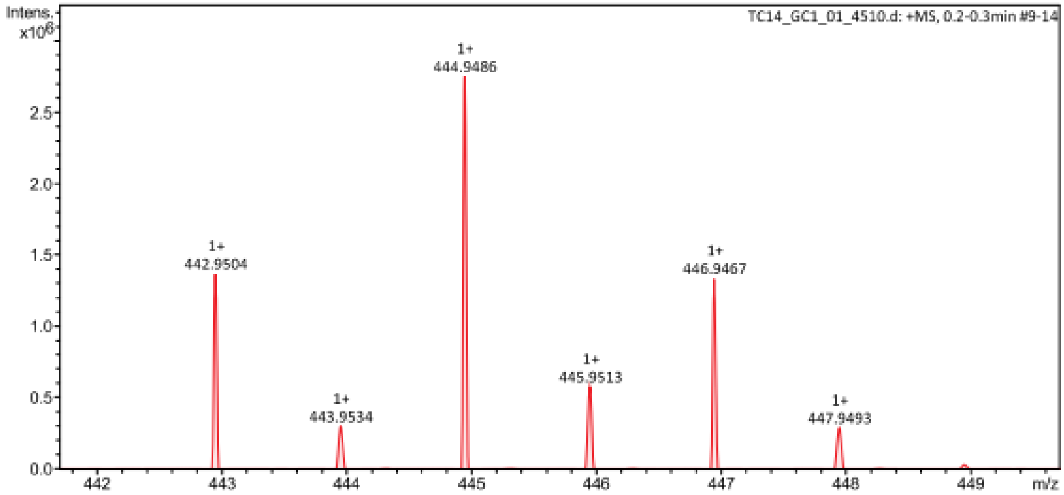

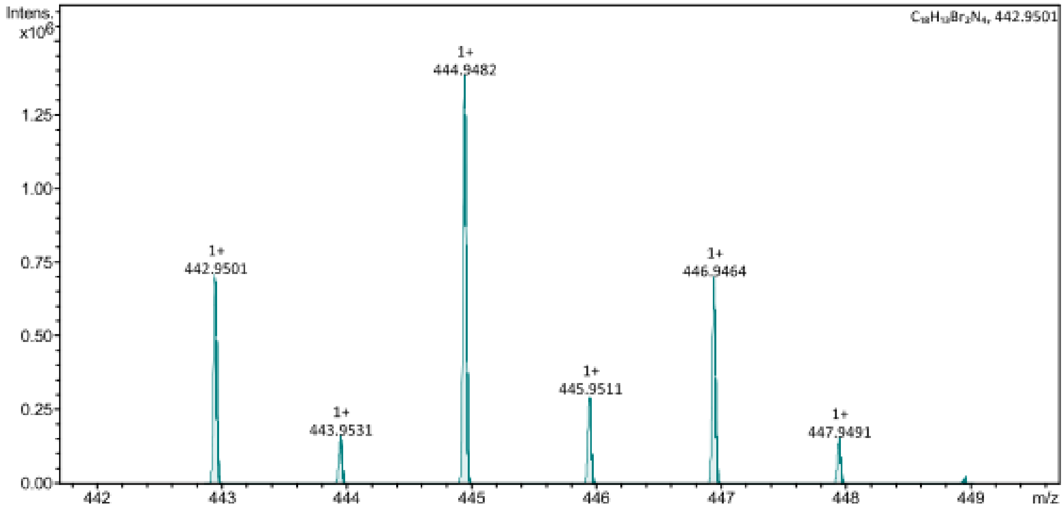

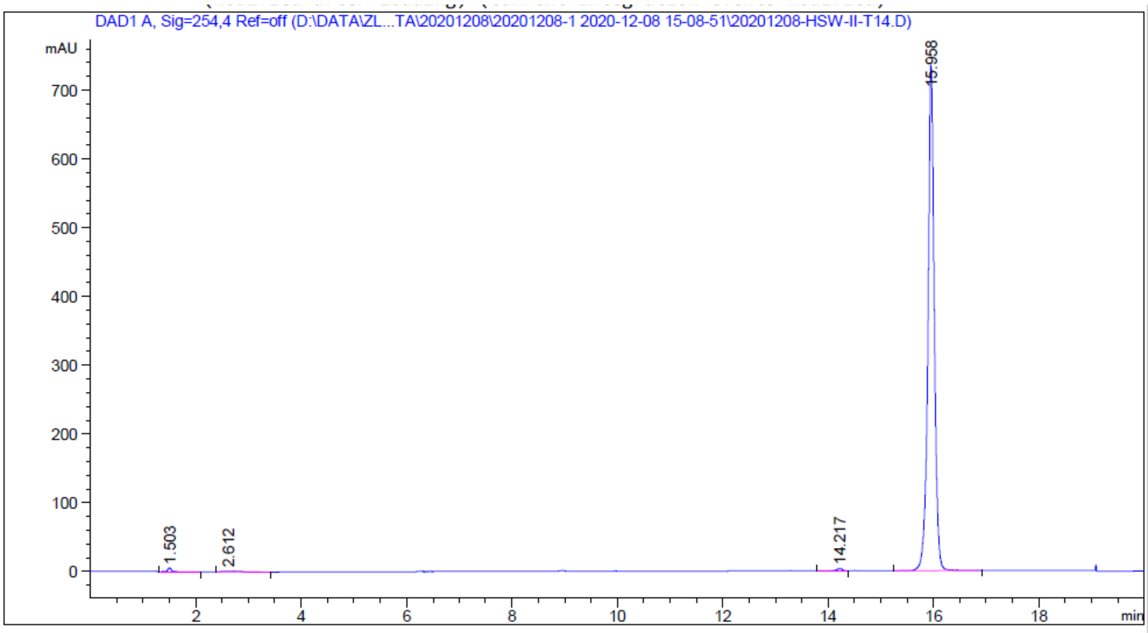

Signal 1: DAD1 A, Sig=254,4 Ref=off

Peak #	RetTime [min]	Type	Width [min]	Area [mAU*s]	Height [mAU]	Area %
1	1.503	BB	0.1000	25.79908	5.01536	0.4132
2	2.612	BB	0.3366	30.30110	1.25975	0.4853
3	14.217	BB	0.1266	25.84705	3.48303	0.4139
4	15.958	BB	0.1392	6162.29248	724.47101	98.6876

1260R 12/8/2020 9:03:26 PM BY

Data File D:\DATA\ZLM\DATA\20201208\20201208-1 2020-12-08 15-08-51\202012
Sample Name: 20201208-HSW-II-T14

Totals : 6244.23971 734.22915

^{1}H NMR, ^{13}C NMR, HRMS, and HPLC of compound B8

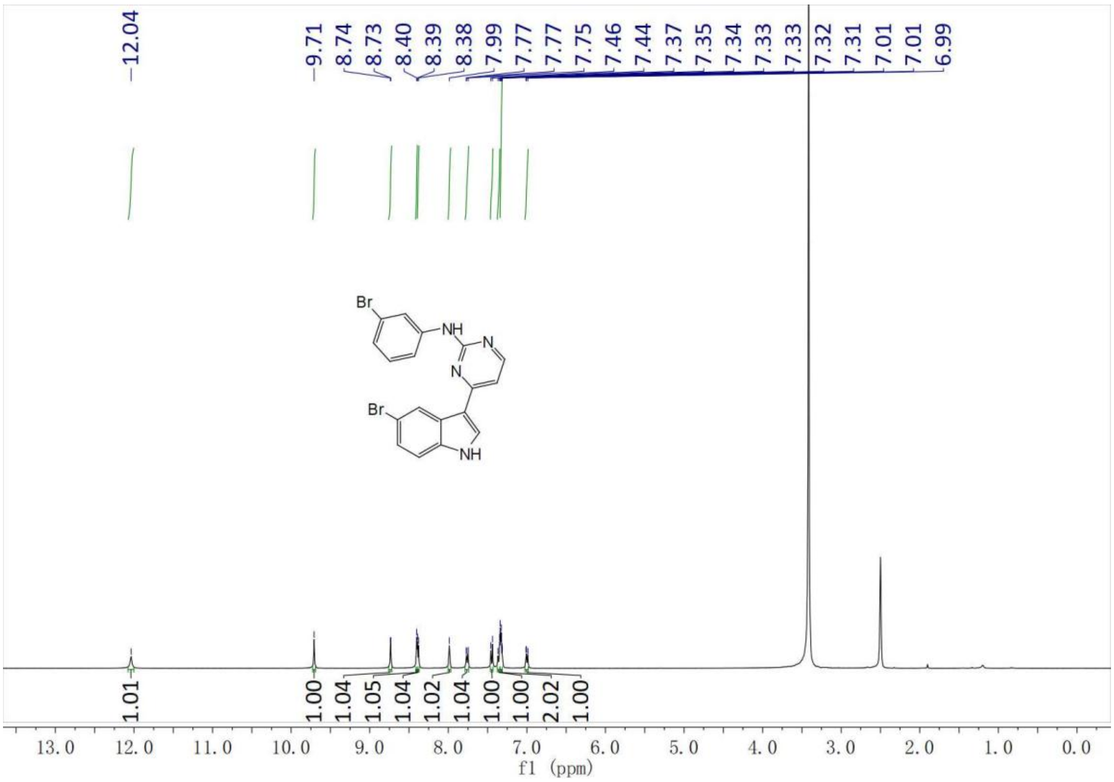

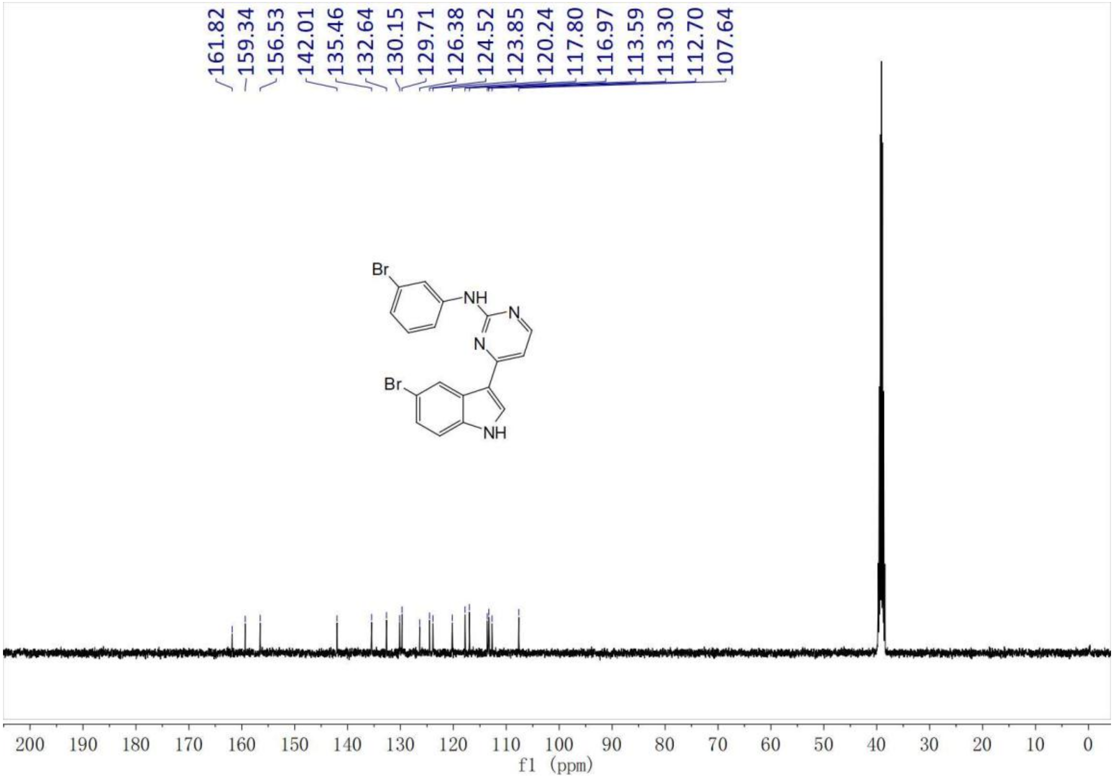

Display Report

Analysis Info

Analysis Name	D:\Data\data\2019\20190711-HSW-TC16_GA4_01_2057.d	Acquisition Date	7/11/2019 17:48:55 PM
Method	MS-2MIN-POS.m	Operator	BDAL@DE
Sample Name	20190711-HSW-TC16	Instrument	compact 8255754.20127
Comment			

Acquisition Parameter

Source Type	ESI	Ion Polarity	Positive	Set Nebulizer	2.0 Bar
Focus	Active	Set Capillary	4500 V	Set Dry Heater	200 °C
Scan Begin	50 m/z	Set End Plate Offset	-500 V	Set Dry Gas	8.0 l/min
Scan End	3000 m/z	Set Charging Voltage	2000 V	Set Divert Valve	Waste
		Set Corona	0 nA	Set APCI Heater	0 °C

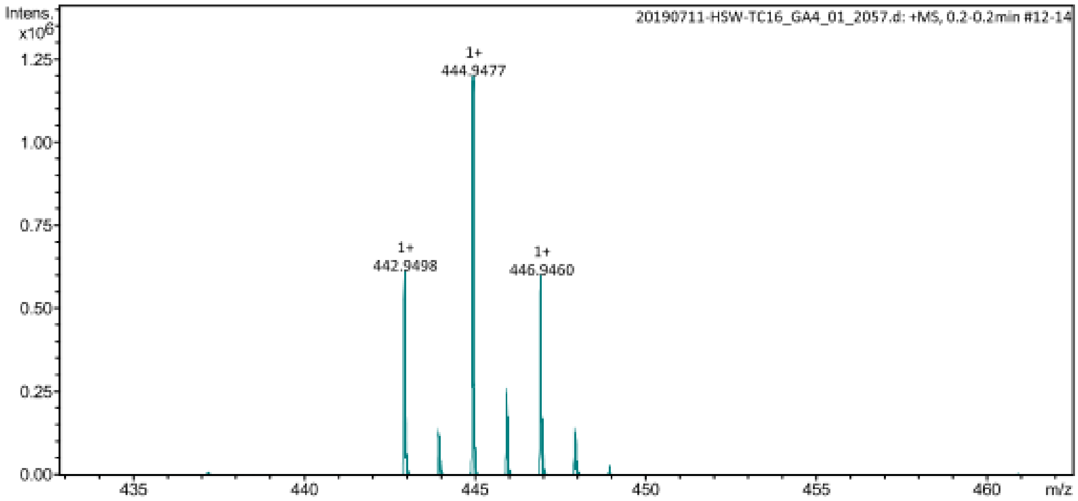

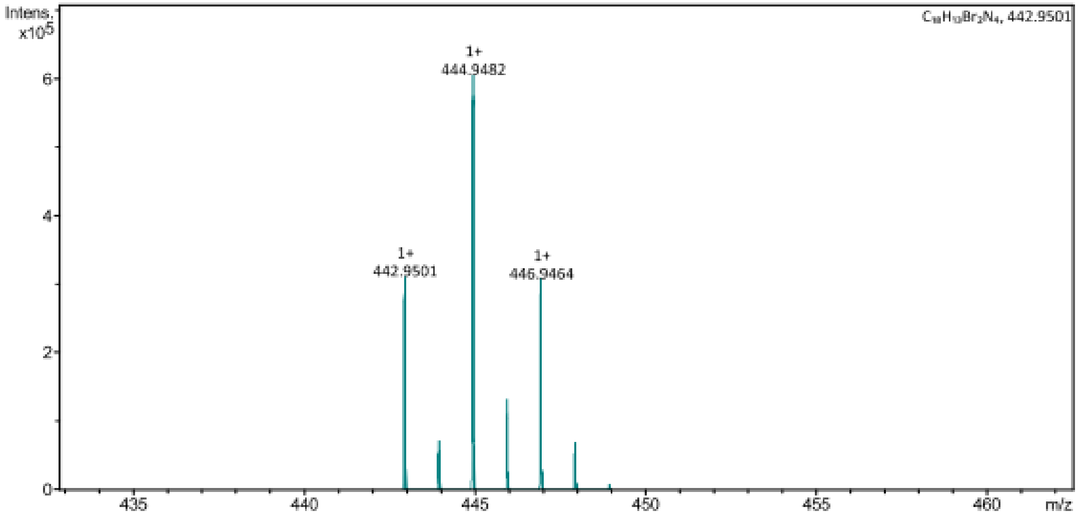

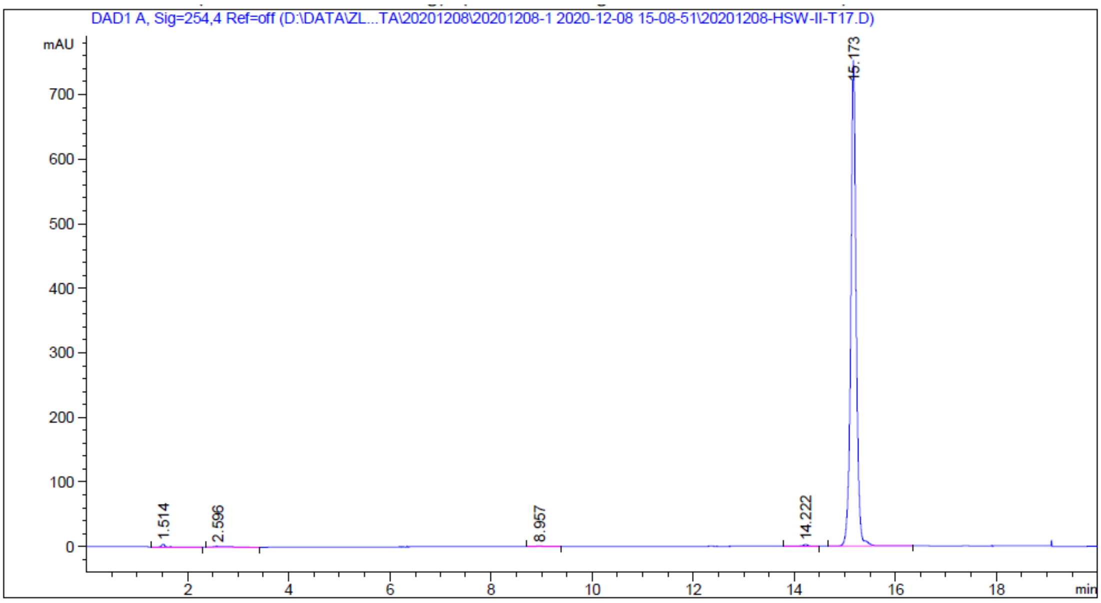

Signal 1: DAD1 A, Sig=254,4 Ref=off

Peak #	RetTime [min]	Type	Width [min]	Area [mAU*s]	Height [mAU]	Area %
1	1.514	BB	0.0924	18.85104	3.64267	0.3355
2	2.596	BB	0.2945	30.89582	1.41609	0.5498
3	8.957	BB	0.1427	9.59806	1.09031	0.1708
4	14.222	BB	0.1380	20.78163	2.47424	0.3698
5	15.173	BB	0.1373	5539.01563	723.59308	98.5740

1260R 12/8/2020 9:11:07 PM BY

Data File D:\DATA\ZLM\DATA\20201208\20201208-1 2020-12-08 15-08-51\20201208-H
Sample Name: 20201208-HSW-II-T17

Peak #	RetTime [min]	Type	Width [min]	Area [mAU*s]	Height [mAU]	Area %
Totals :				5619.14217	732.21639	

^{1}H NMR, ^{13}C NMR, HRMS, and HPLC of compound B9

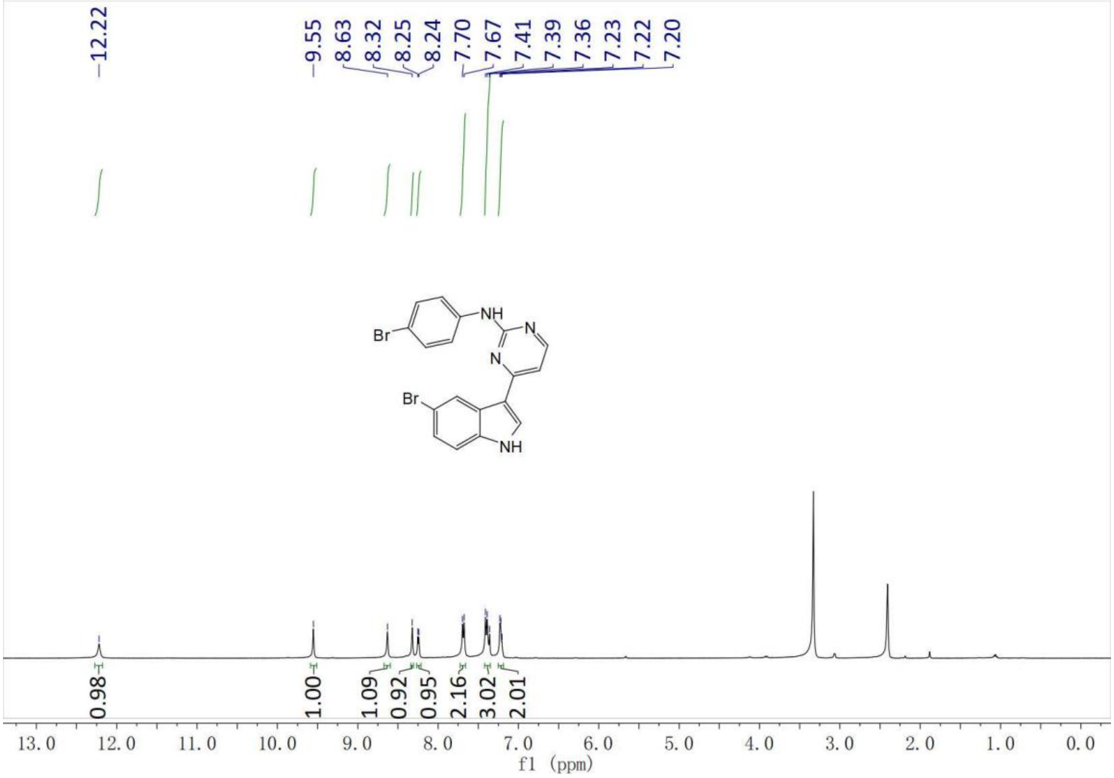

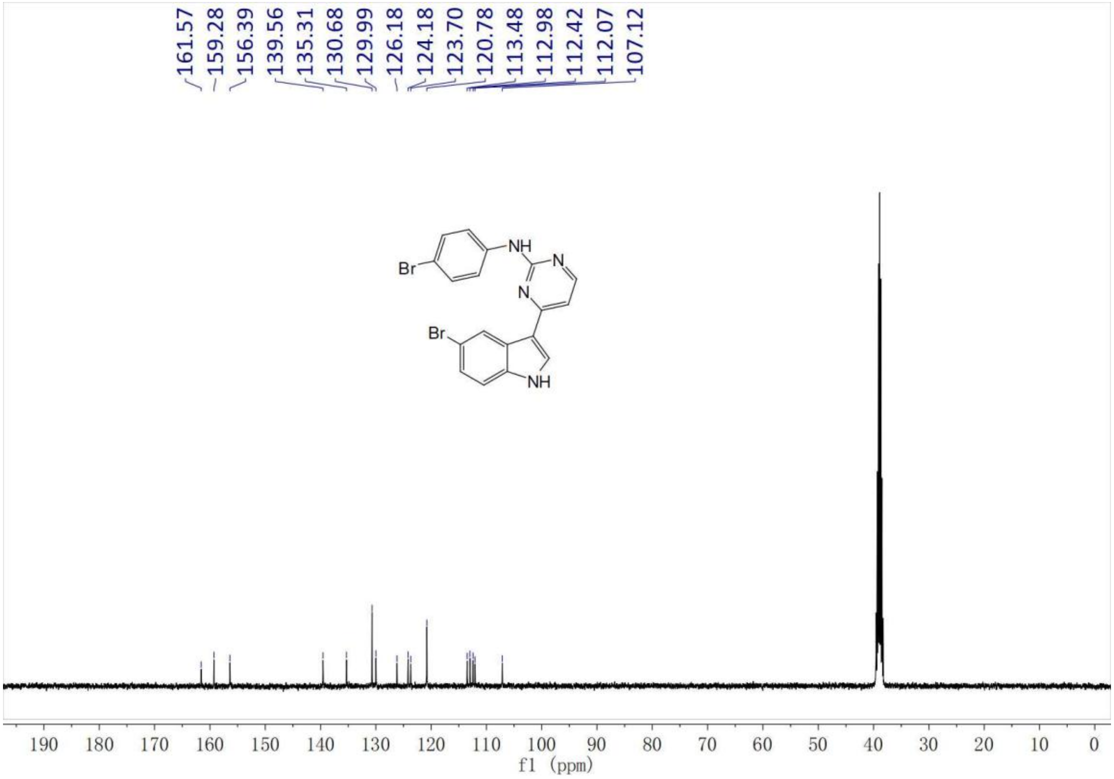

Display Report

Analysis Info | Acquisition Date 12/10/2020 16:45:16 PM

Sample Name TC7
Comment

Acquisition Parameter

Source Type	ESI	Ion Polarity	Positive	Set Nebulizer	2.0 Bar
Focus	Active	Set Capillary	4500 V	Set Dry Heater	200 °C
Scan Begin	50 m/z	Set End Plate Offset	-500 V	Set Dry Gas	8.0 l/min
Scan End	3000 m/z	Set Charging Voltage	2000 V	Set Divert Valve	Waste
		Set Corona	0 nA	Set APCI Heater	0 °C

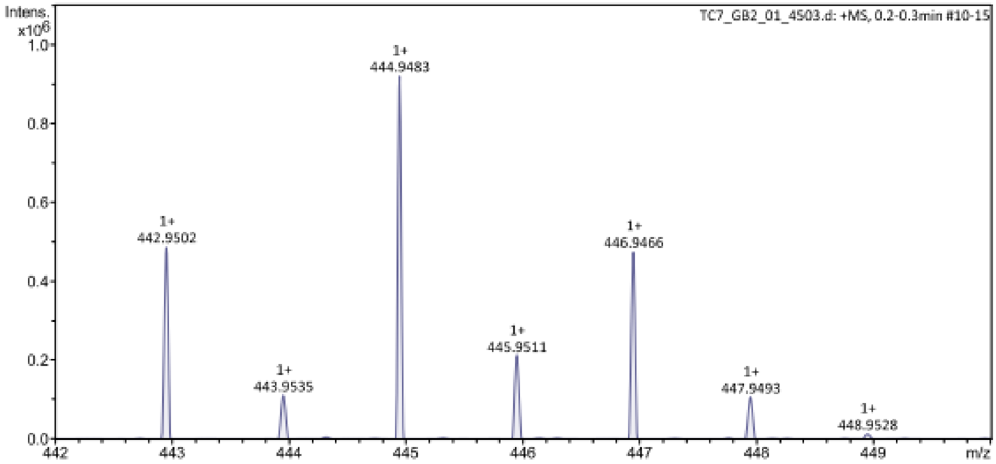

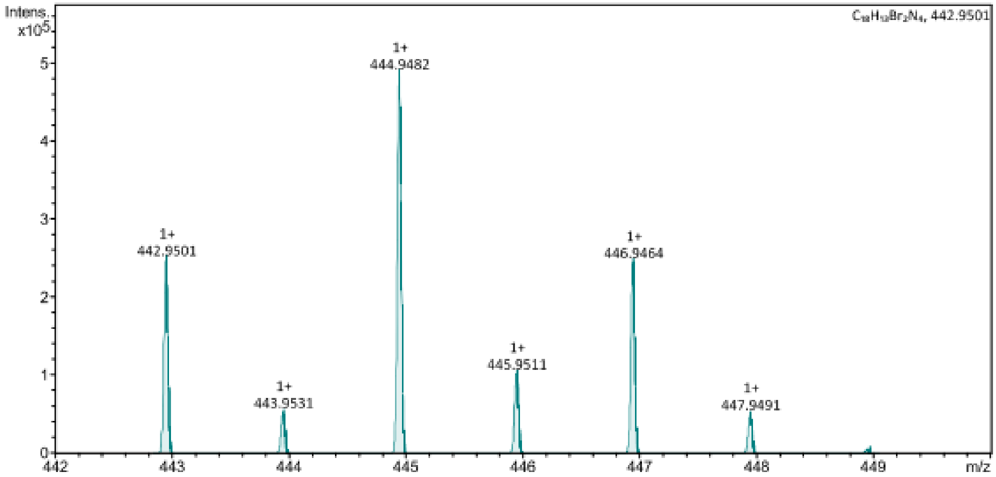

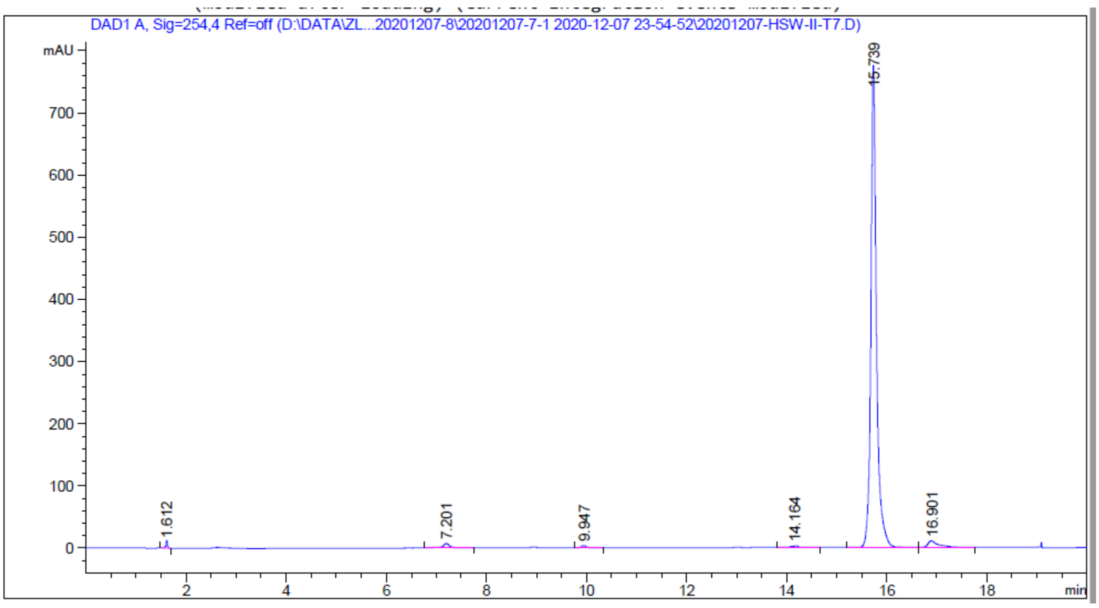

Signal 1: DAD1 A, Sig=254,4 Ref=off

Peak #	RetTime [min]	Type	Width [min]	Area [mAU*s]	Height [mAU]	Area %
1	1.612	BB	0.0517	24.13286	10.15053	0.3788
2	7.201	BB	0.1698	74.08088	7.09796	1.1627
3	9.947	BB	0.1142	23.03635	3.26932	0.3615
4	14.164	BB	0.1847	29.84029	2.54709	0.4683
5	15.739	BV	0.1363	6060.05859	733.97455	95.1109

1260R 12/8/2020 2:19:33 PM BY

Data File D:\DATA\ZL...TA\20201207-8\20201207-7-1 2020-12-07 23-54-52\20201207-H

Sample Name: 20201207-HSW-II-T7

Peak #	RetTime [min]	Type	Width [min]	Area [mAU*s]	Height [mAU]	Area %
6	16.901	VB	0.2145	160.42046	10.72761	2.5178

Totals :				6371.56943	767.76707	

^{1}H NMR, ^{13}C NMR, HRMS, and HPLC of compound B10

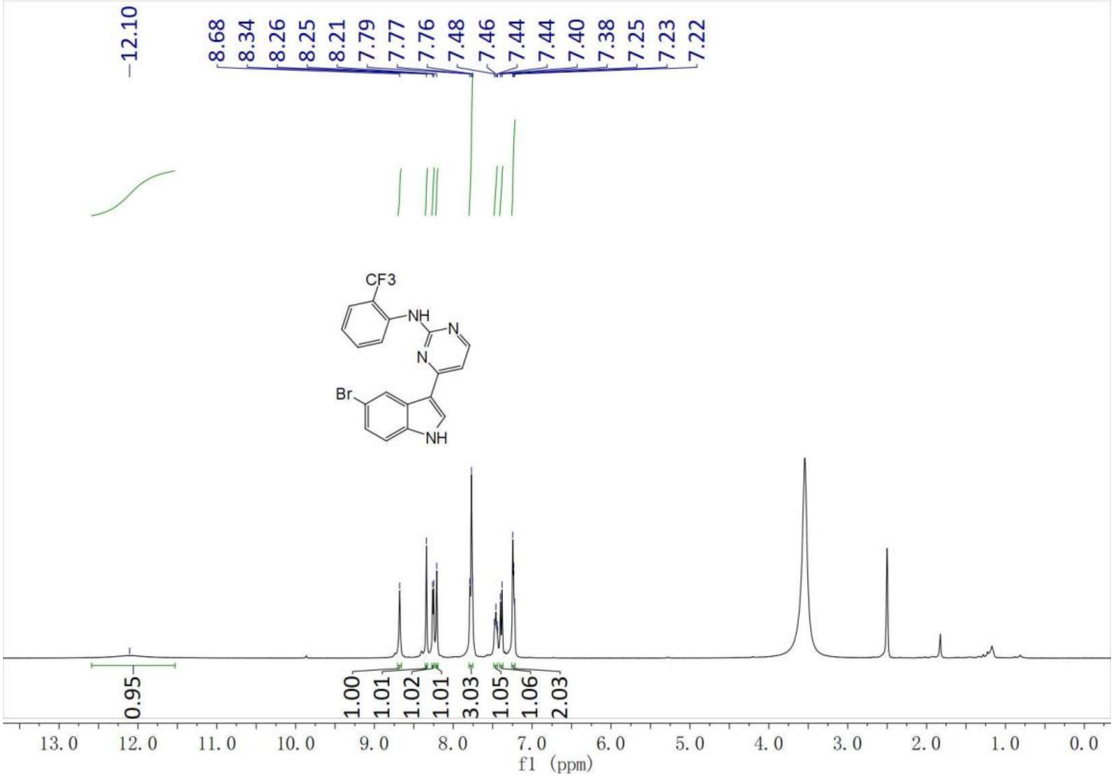

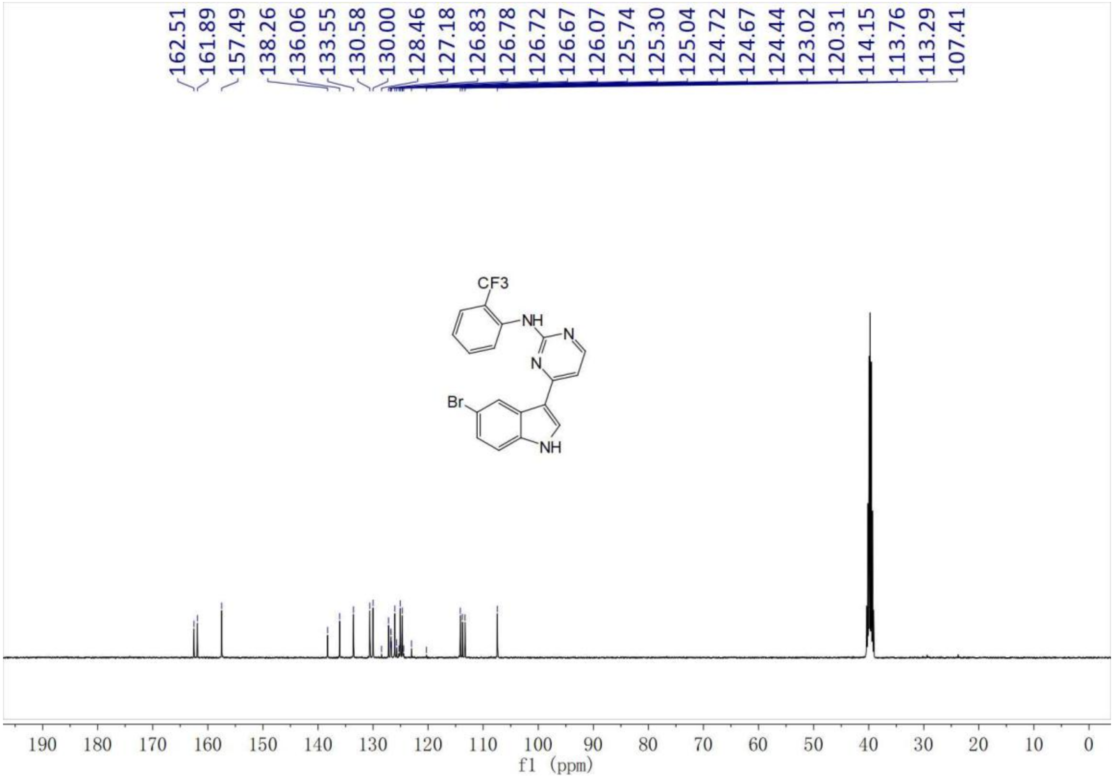

Display Report

Analysis Info

Acquisition Date 12/10/2020 17:50:23 PM

Sample Name TC32
Comment

Acquisition Parameter

Source Type	ESI	Ion Polarity	Positive	Set Nebulizer	2.0 Bar
Focus	Active	Set Capillary	4500 V	Set Dry Heater	200 °C
Scan Begin	50 m/z	Set End Plate Offset	-500 V	Set Dry Gas	8.0 l/min
Scan End	3000 m/z	Set Charging Voltage	2000 V	Set Divert Valve	Waste
		Set Corona	0 nA	Set APCI Heater	0 °C

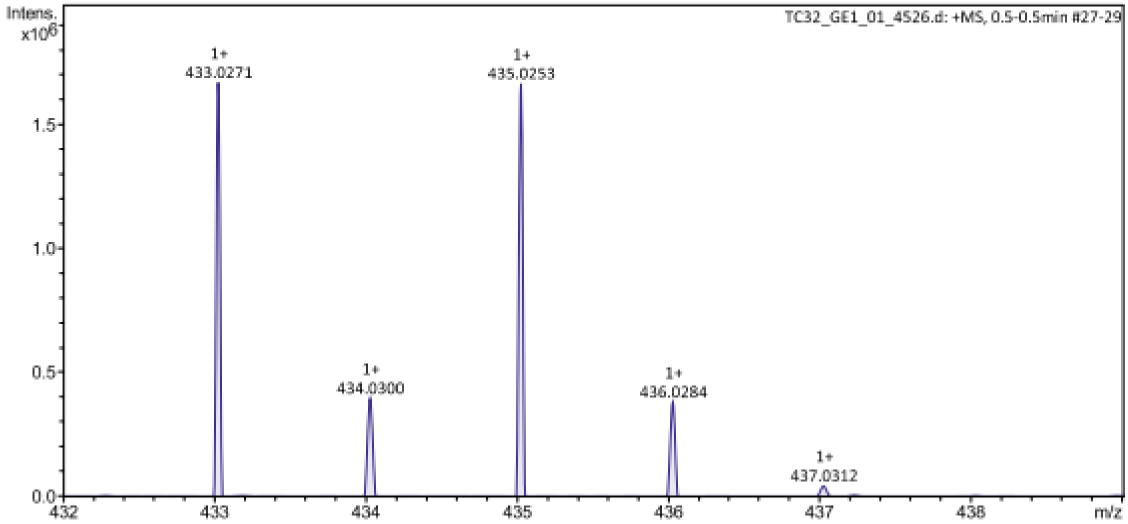

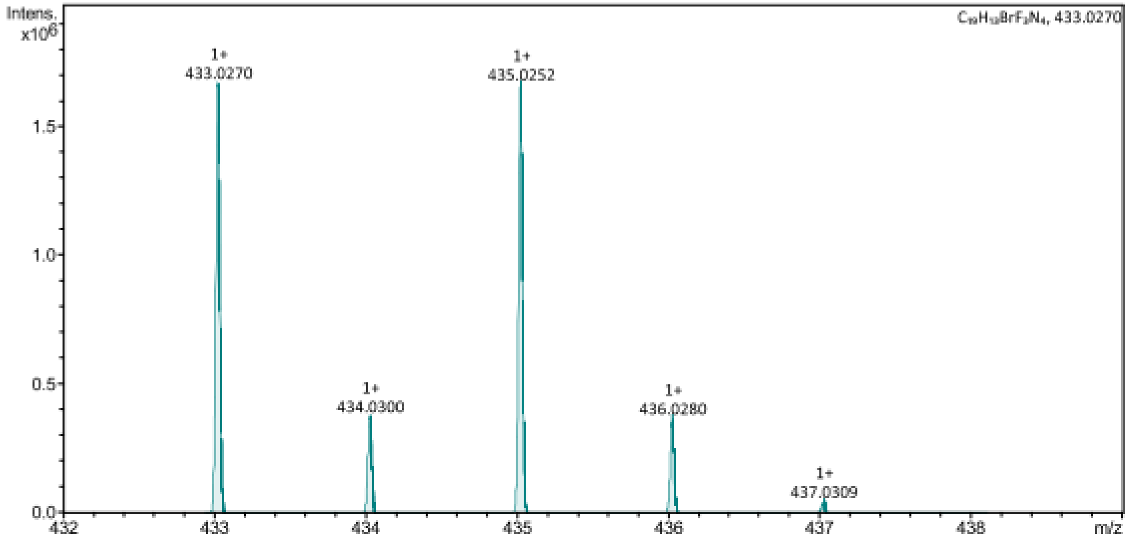

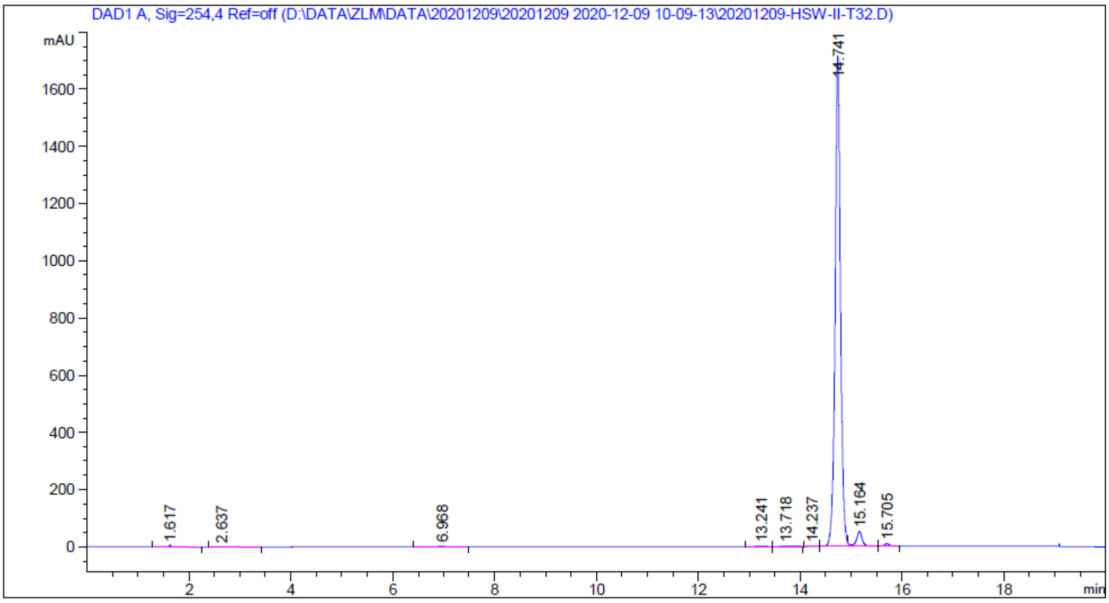

Signal 1: DAD1 A, Sig=254,4 Ref=off

Peak #	RetTime [min]	Type	Width [min]	Area [mAU*s]	Height [mAU]	Area %
1	1.617	BB	0.1050	20.90951	3.34278	0.1602
2	2.637	BB	0.3931	36.64771	1.40118	0.2807
3	6.968	BB	0.2012	18.22401	1.38816	0.1396
4	13.241	BV	0.1966	16.68127	1.38372	0.1278

1260R 12/9/2020 6:02:00 PM BY

Data File D:\DATA\ZLM\DATA\20201209\20201209 2020-12-09 10-09-13\20201209-H
Sample Name: 20201209-HSW-II-T32

Peak #	RetTime [min]	Type	Width [min]	Area [mAU*s]	Height [mAU]	Area %
5	13.718	VB	0.2206	21.78139	1.40723	0.1669
6	14.237	BB	0.1144	9.74391	1.52694	0.0746
7	14.741	BV R	0.1267	1.24547e4	1675.80933	95.4074
8	15.164	VV E	0.1336	412.41901	51.39032	3.1593
9	15.705	VB	0.1104	63.11677	9.39640	0.4835
Totals :				1.30542e4	1747.04605	

^{1}H NMR, ^{13}C NMR, HRMS, and HPLC of compound B11

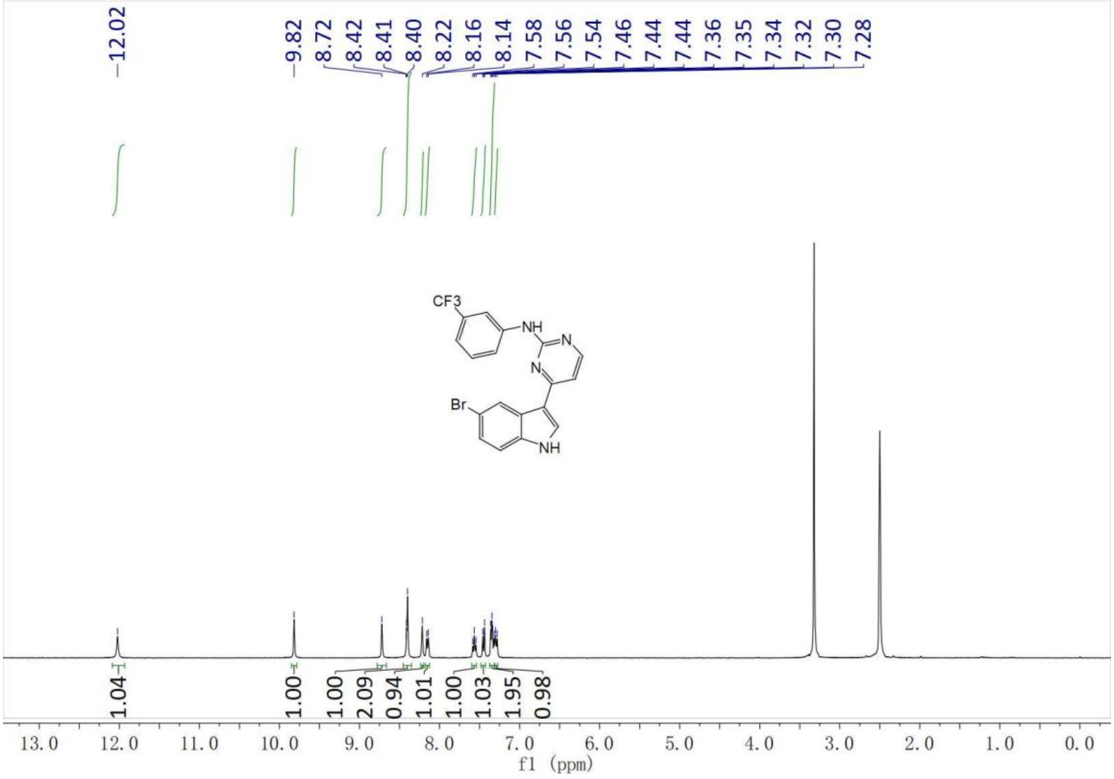

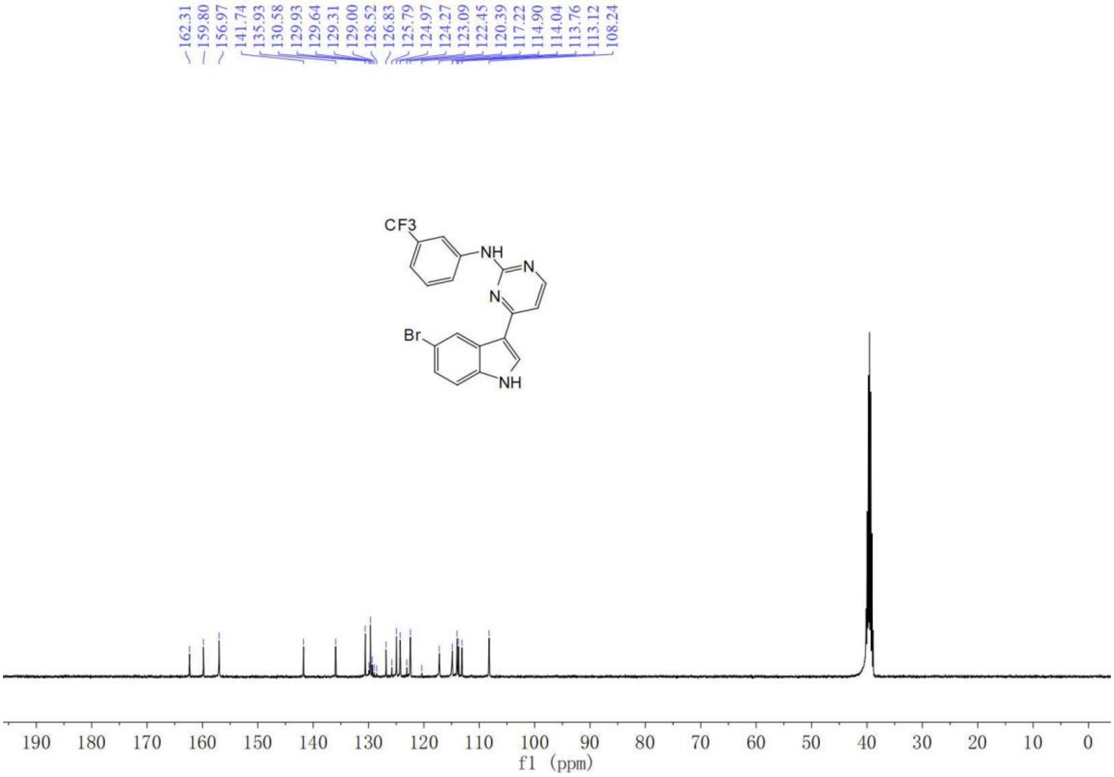

Display Report

Analysis Info

Acquisition Date 12/10/2020 17:19:13 PM

Sample Name TC19
Comment

Acquisition Parameter

Source Type	ESI	Ion Polarity	Positive	Set Nebulizer	2.0 Bar
Focus	Active	Set Capillary	4500 V	Set Dry Heater	200 °C
Scan Begin	50 m/z	Set End Plate Offset	-500 V	Set Dry Gas	8.0 l/min
Scan End	3000 m/z	Set Charging Voltage	2000 V	Set Divert Valve	Waste
		Set Corona	0 nA	Set APCI Heater	0 °C

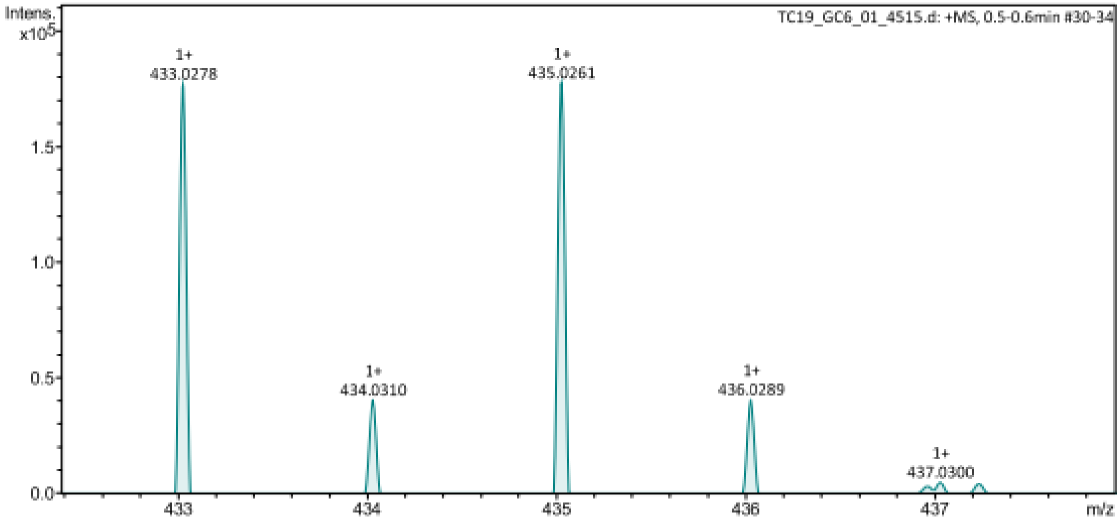

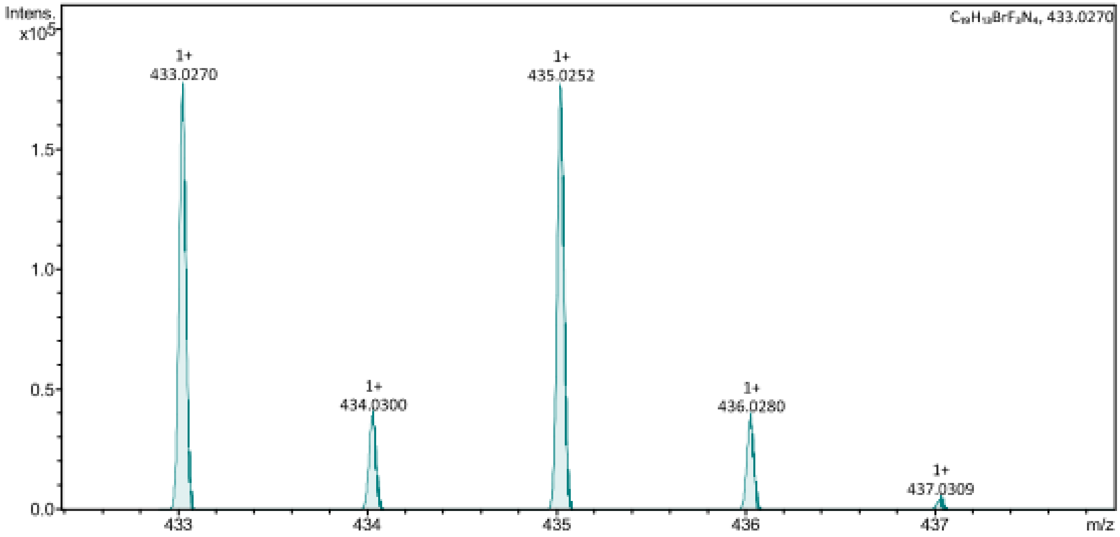

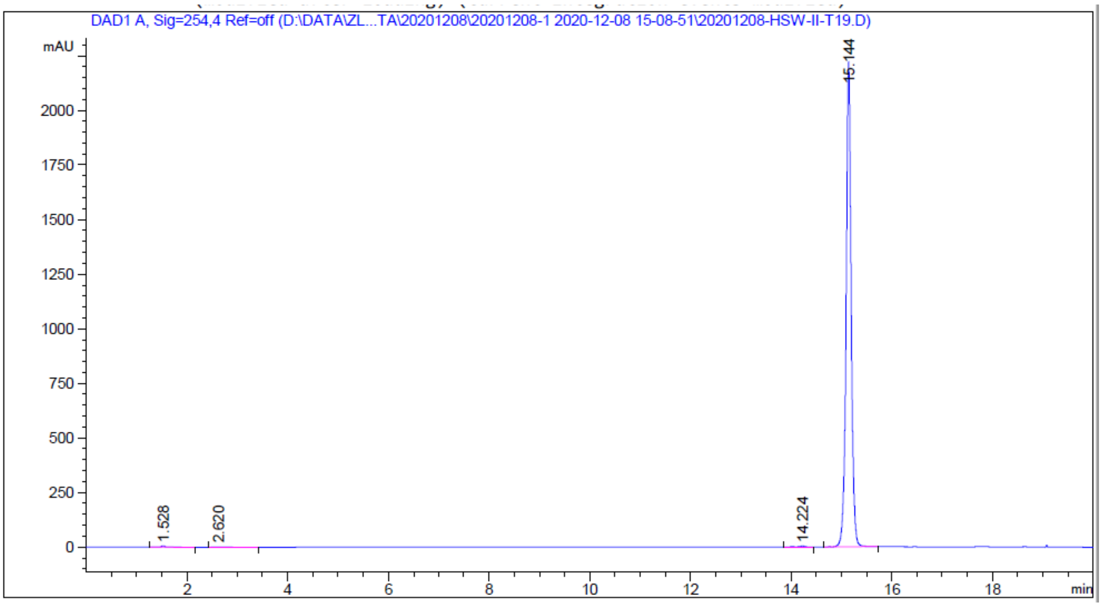

Signal 1: DAD1 A, Sig=254,4 Ref=off

Peak #	RetTime [min]	Type	Width [min]	Area [mAU*s]	Height [mAU]	Area %
1	1.528	BB	0.0940	28.39580	5.34351	0.1821
2	2.620	BB	0.3018	33.23596	1.52808	0.2131
3	14.224	BB	0.1388	32.03264	3.78117	0.2054
4	15.144	BB	0.1241	1.55031e4	2150.96069	99.3995

1260R 12/8/2020 9:16:11 PM BY

Data File D:\DATA\ZLM\DATA\20201208\20201208-1 2020-12-08 15-08-51\20201208-HSW

Sample Name: 20201208-HSW-II-T19

Totals : 1.55967e4 2161.61346

^{1}H NMR, ^{13}C NMR, HRMS, and HPLC of compound B12

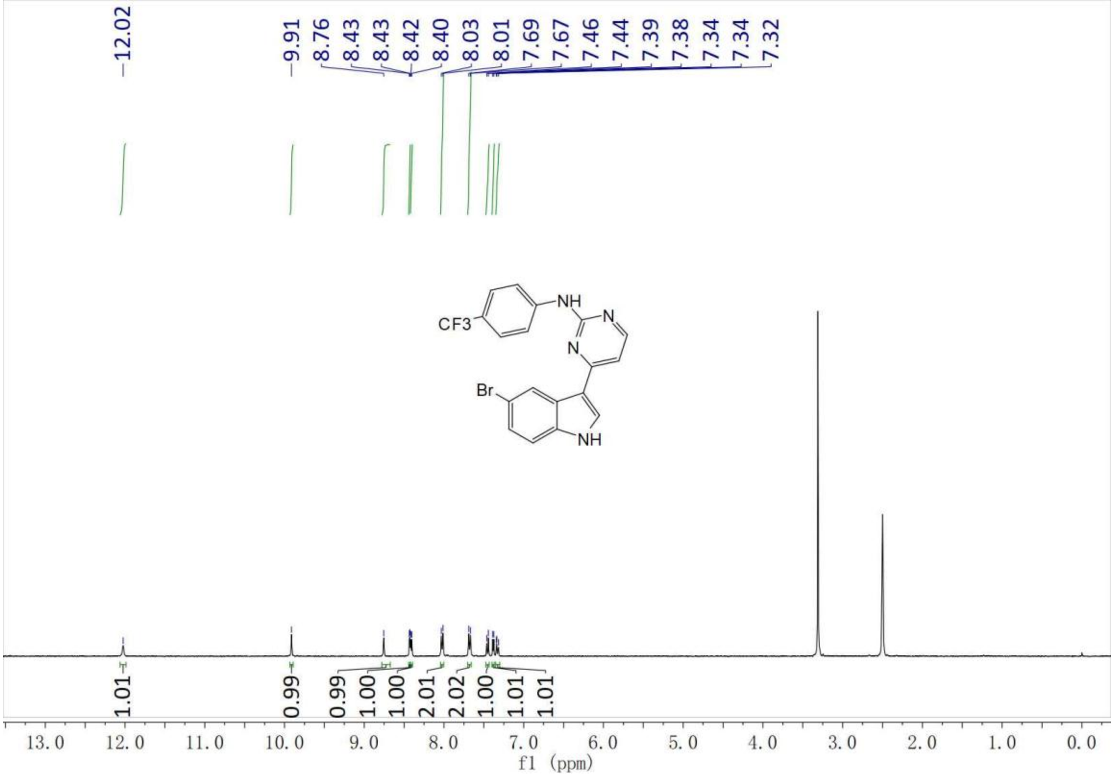

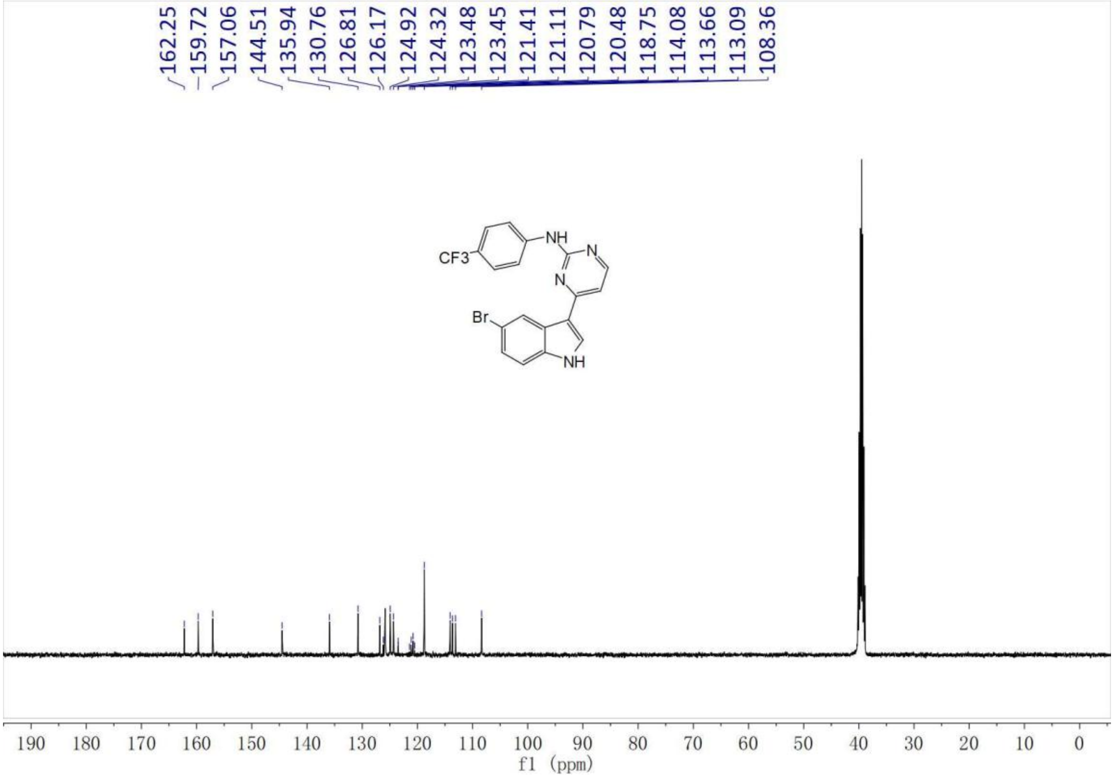

Display Report

Analysis Info

Acquisition Date 12/10/2020 16:39:46 PM

Sample Name TC5
Comment

Acquisition Parameter

Source Type	ESI	Ion Polarity	Positive	Set Nebulizer	2.0 Bar
Focus	Active	Set Capillary	4500 V	Set Dry Heater	200 °C
Scan Begin	50 m/z	Set End Plate Offset	-500 V	Set Dry Gas	8.0 l/min
Scan End	3000 m/z	Set Charging Voltage	2000 V	Set Divert Valve	Waste
		Set Corona	0 nA	Set APCI Heater	0 °C

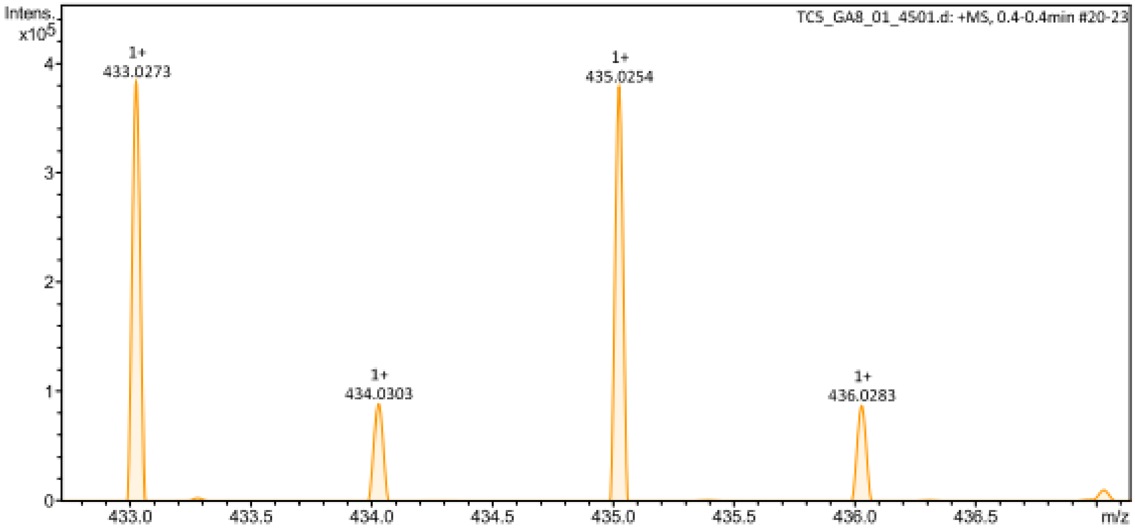

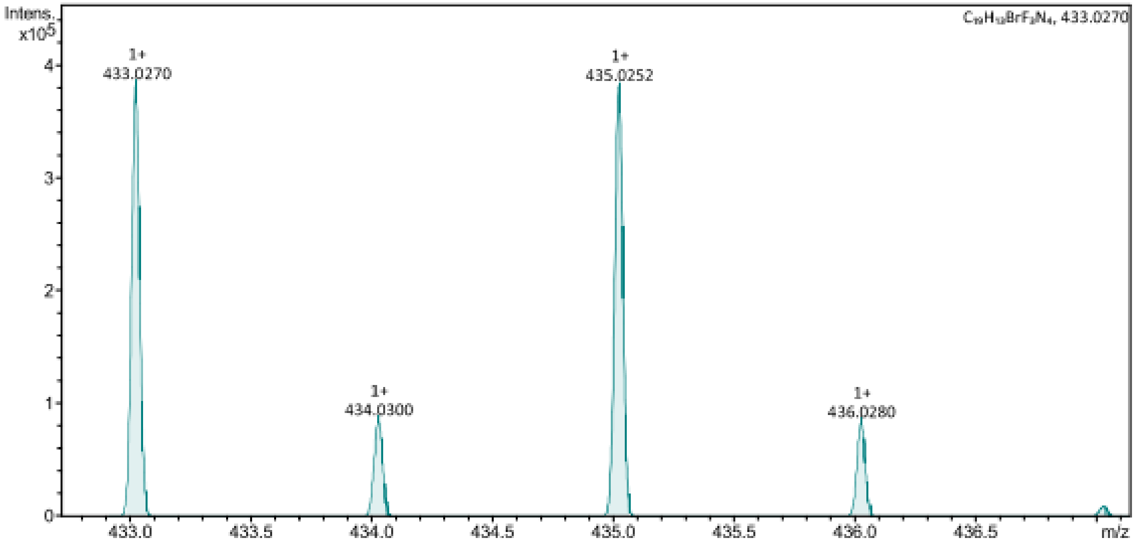

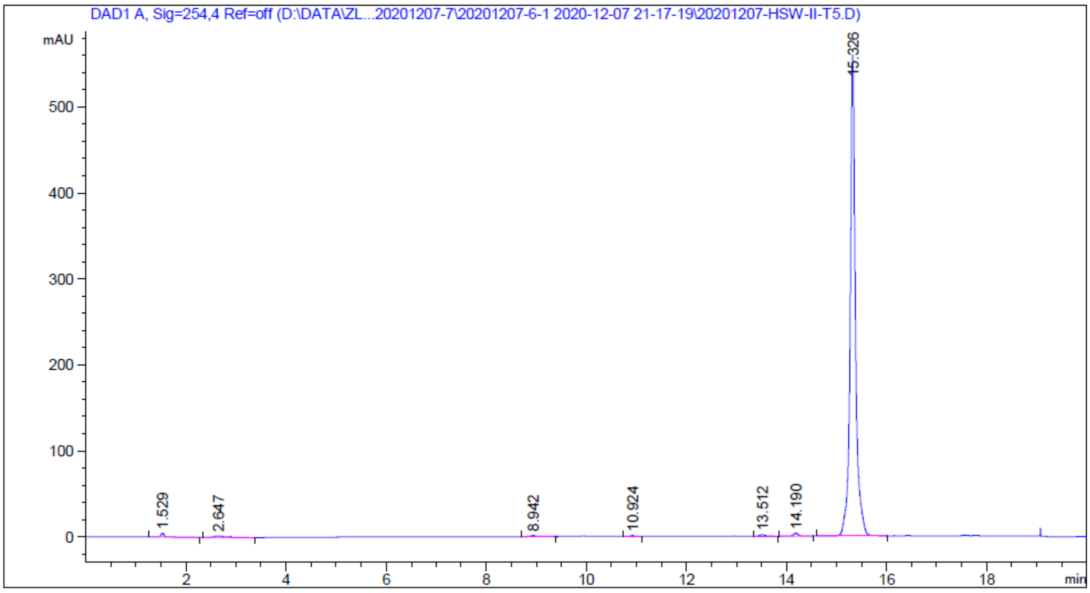

Signal 1: DAD1 A, Sig=254,4 Ref=off

Peak #	RetTime [min]	Type	Width [min]	Area [mAU*s]	Height [mAU]	Area %
1	1.529	BB	0.0927	22.40524	4.30622	0.5299
2	2.647	BB	0.2697	31.50411	1.65791	0.7451
3	8.942	BB	0.1451	11.20659	1.24438	0.2651
4	10.924	BB	0.1106	6.39390	1.05703	0.1512
5	13.512	BB	0.1338	13.65506	1.69795	0.3230

1260R 12/8/2020 2:14:24 PM BY

Data File D:\DATA\ZL...TA\20201207-7\20201207-6-1 2020-12-07 21-17-19\
Sample Name: 20201207-HSW-II-T5

Peak #	RetTime [min]	Type	Width [min]	Area [mAU*s]	Height [mAU]	Area %
6	14.190	BB	0.1259	24.21241	3.28958	0.5727
7	15.326	BB	0.1278	4118.70410	547.45581	97.4131

Totals : 4228.08140 560.70888

^{1}H NMR, ^{13}C NMR, HRMS, and HPLC of compound B13

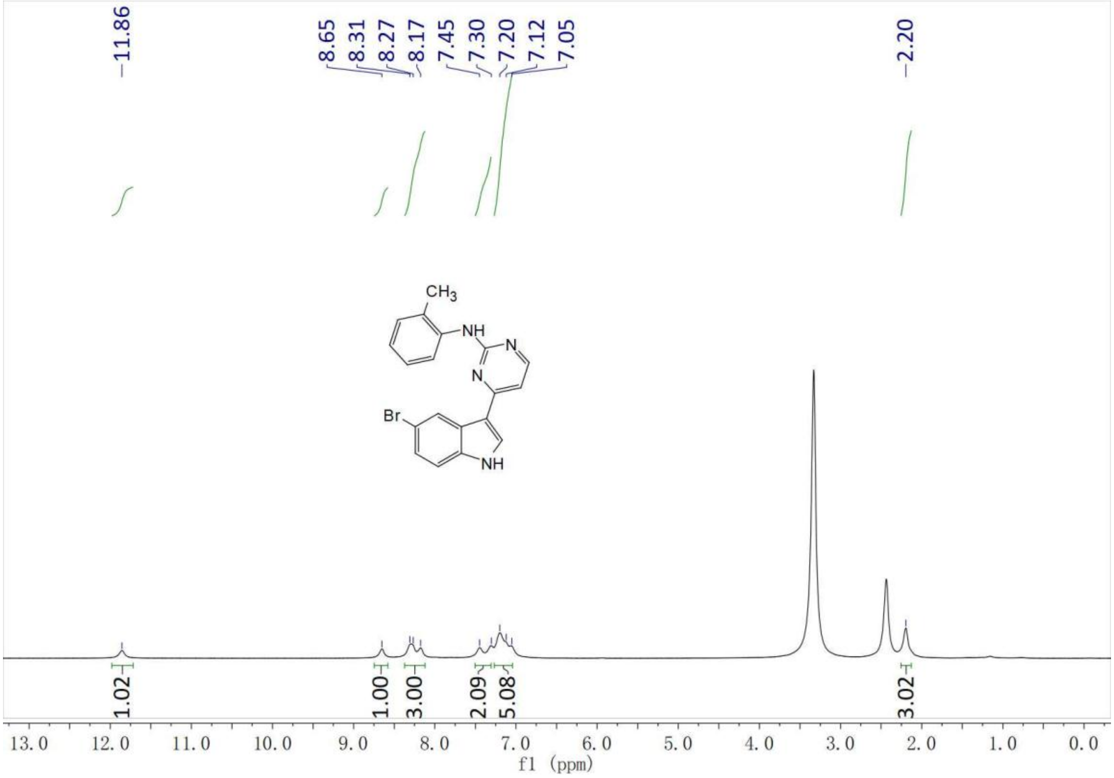

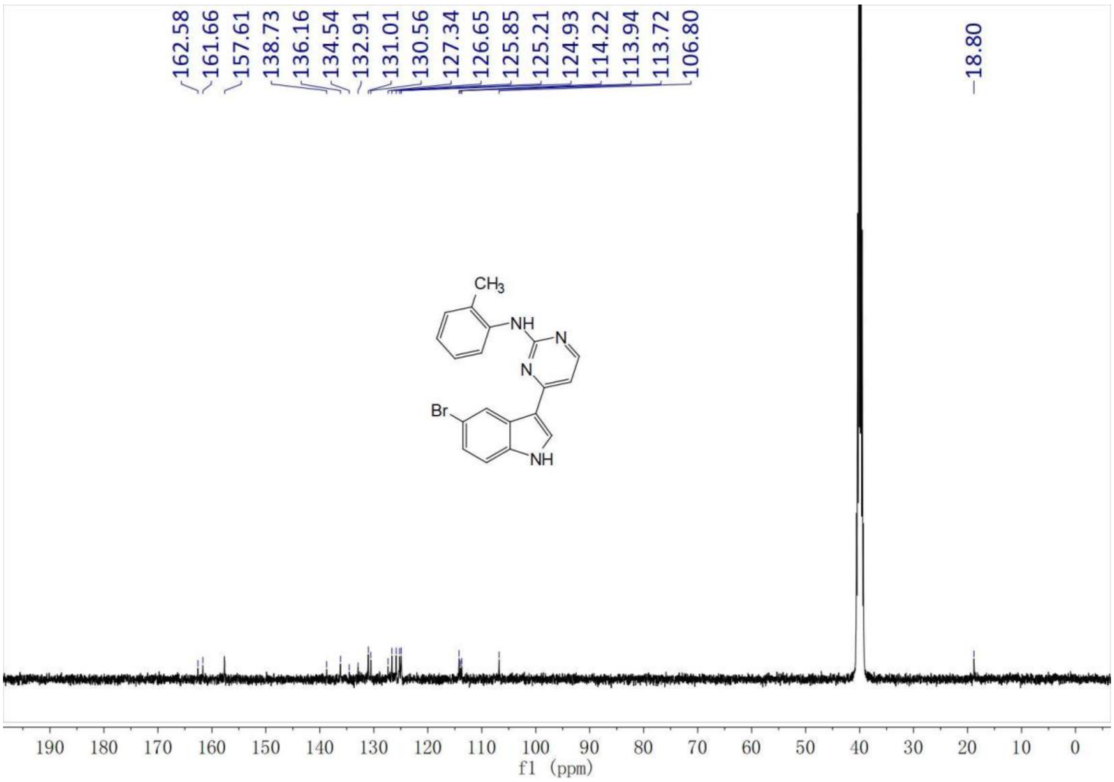

Display Report

Analysis Info

Acquisition Date 12/10/2020 17:44:28 PM

Sample Name TC30
Comment

Acquisition Parameter

Source Type	ESI	Ion Polarity	Positive	Set Nebulizer	2.0 Bar
Focus	Active	Set Capillary	4500 V	Set Dry Heater	200 °C
Scan Begin	50 m/z	Set End Plate Offset	-500 V	Set Dry Gas	8.0 l/min
Scan End	3000 m/z	Set Charging Voltage	2000 V	Set Divert Valve	Waste
		Set Corona	0 nA	Set APCI Heater	0 °C

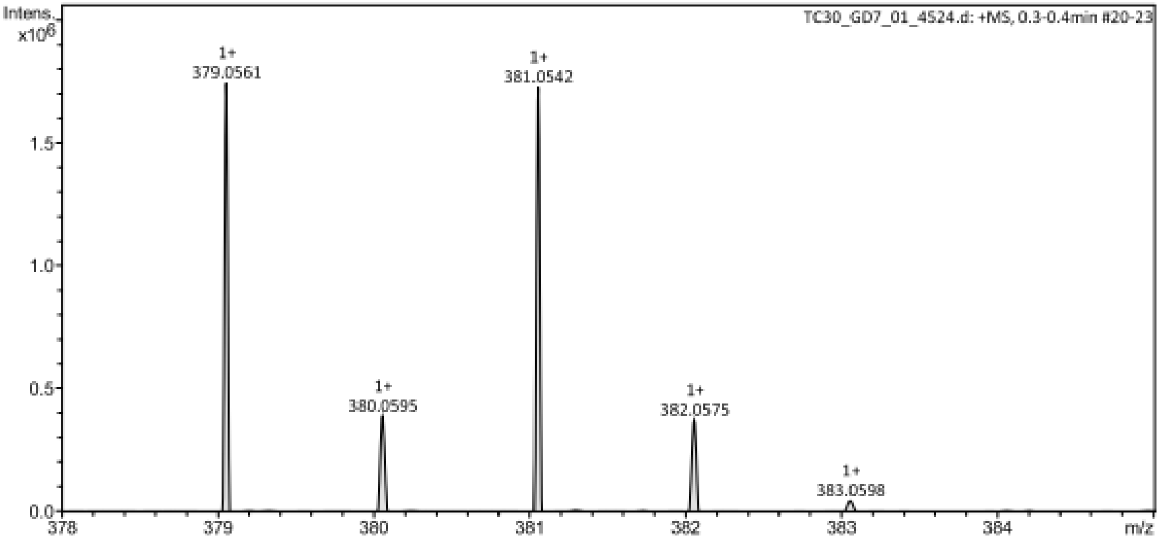

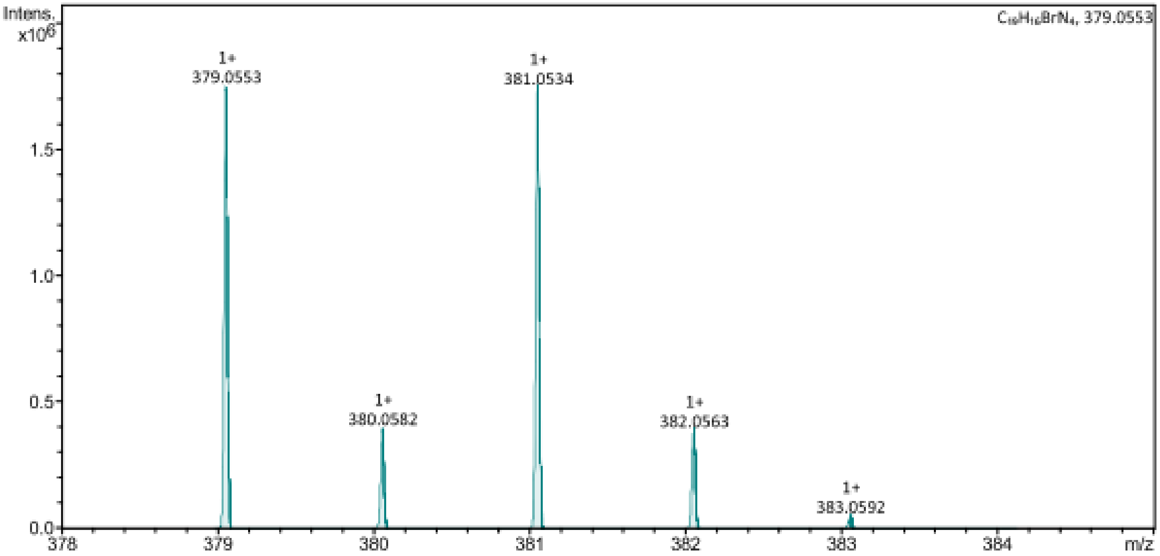

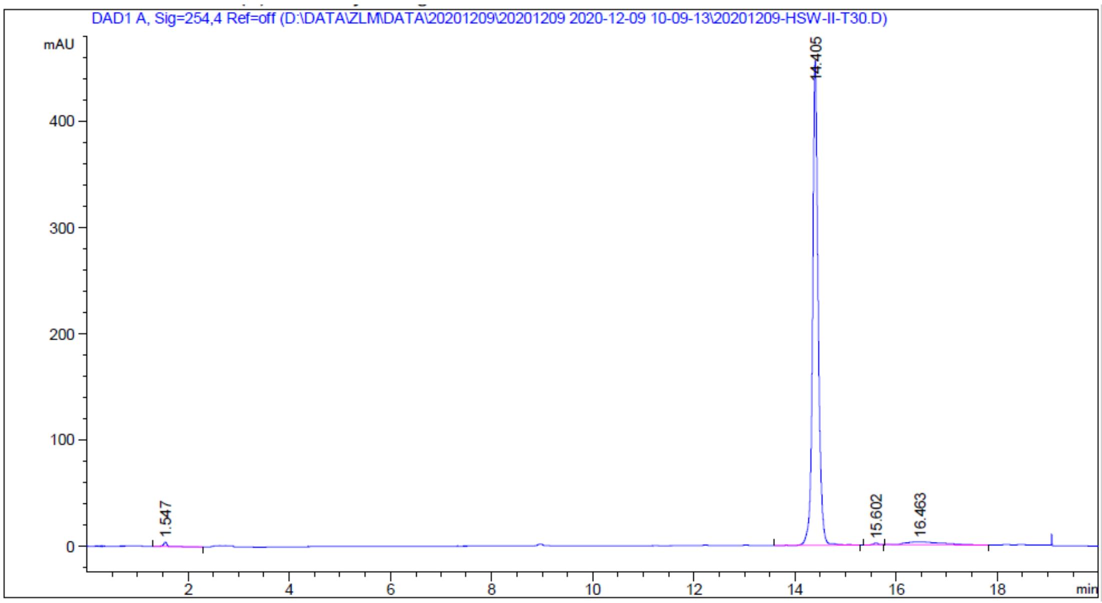

Signal 1: DAD1 A, Sig=254,4 Ref=off

Peak #	RetTime [min]	Type	Width [min]	Area [mAU*s]	Height [mAU]	Area %
1	1.547	BB	0.0994	23.38626	4.04676	0.6388
2	14.405	BB	0.1340	3480.86621	432.06189	95.0843
3	15.602	BB	0.1162	11.45730	1.58812	0.3130
4	16.463	BB	0.7461	145.11282	2.92878	3.9639

1260R 12/9/2020 1:45:53 PM BY

Data File D:\DATA\ZLM\DATA\20201209\20201209 2020-12-09 10-09-13\20201209-HSI
Sample Name: 20201209-HSW-II-T30

Totals : 3660.82259 440.62555

^{1}H NMR, ^{13}C NMR, HRMS, and HPLC of compound B14

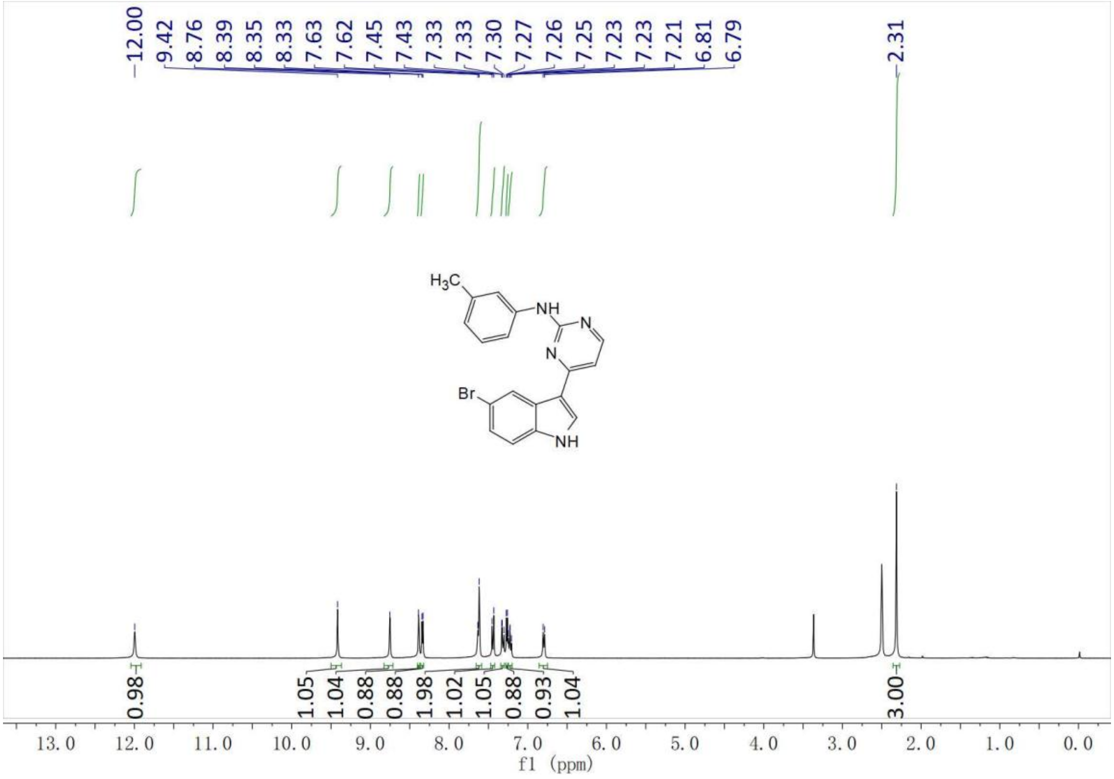

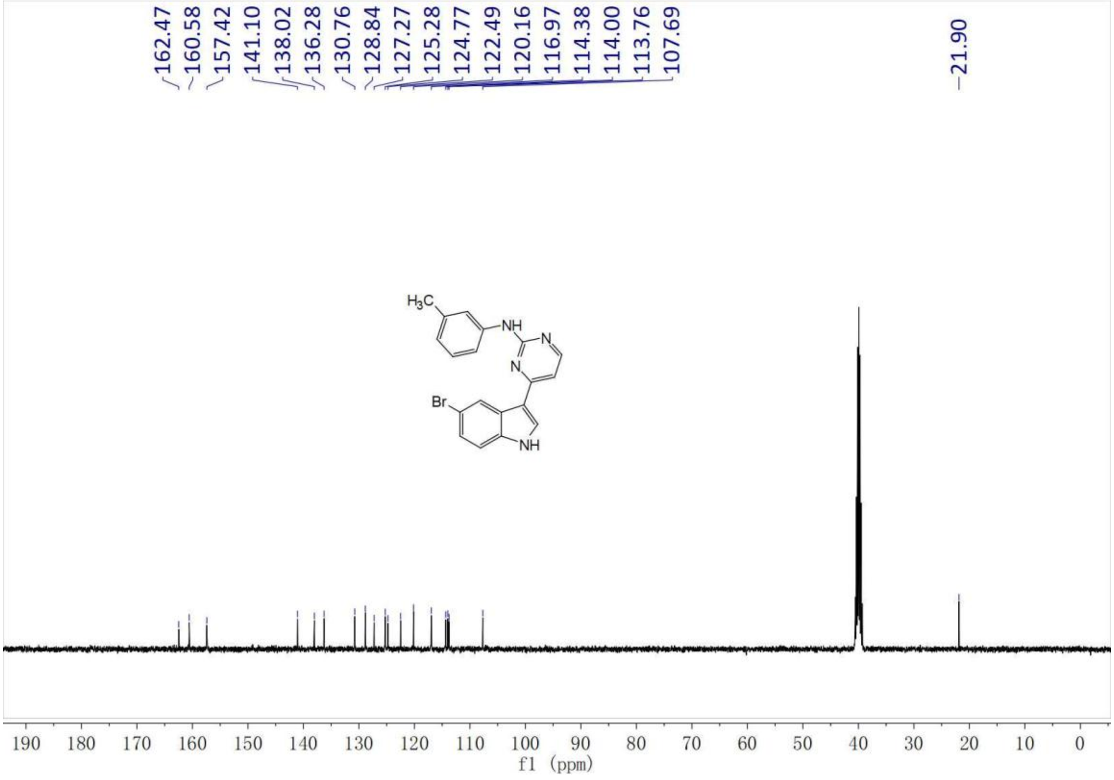

Display Report

Analysis Info Acquisition Date 12/10/2020 17:16:27 PM

Sample Name TC18
Comment

Acquisition Parameter

Source Type	ESI	Ion Polarity	Positive	Set Nebulizer	2.0 Bar
Focus	Active	Set Capillary	4500 V	Set Dry Heater	200 °C
Scan Begin	50 m/z	Set End Plate Offset	-500 V	Set Dry Gas	8.0 l/min
Scan End	3000 m/z	Set Charging Voltage	2000 V	Set Divert Valve	Waste
		Set Corona	0 nA	Set APCI Heater	0 °C

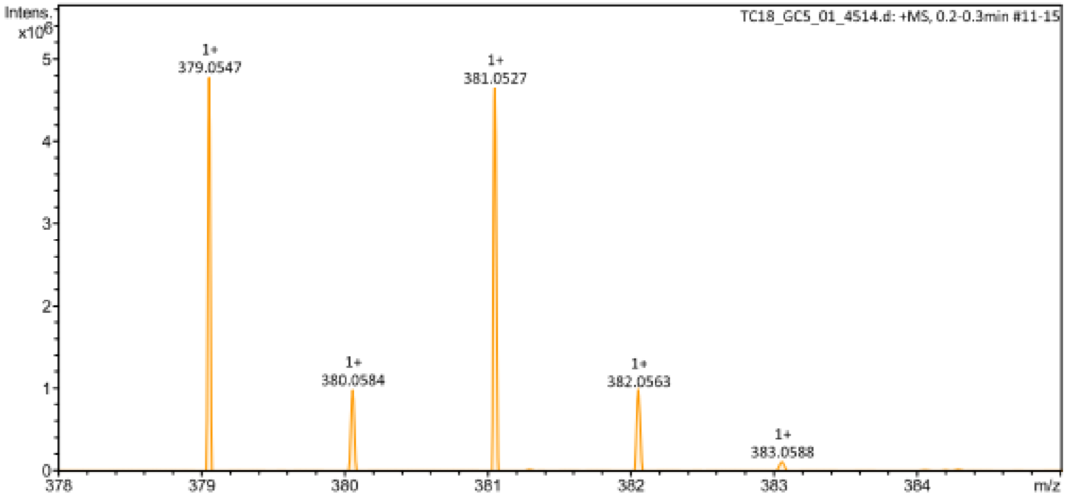

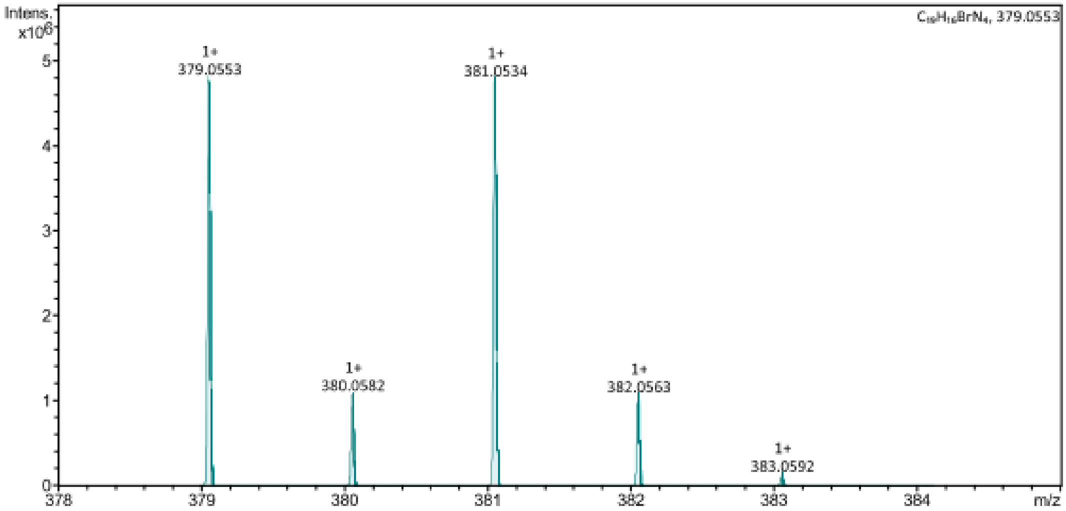

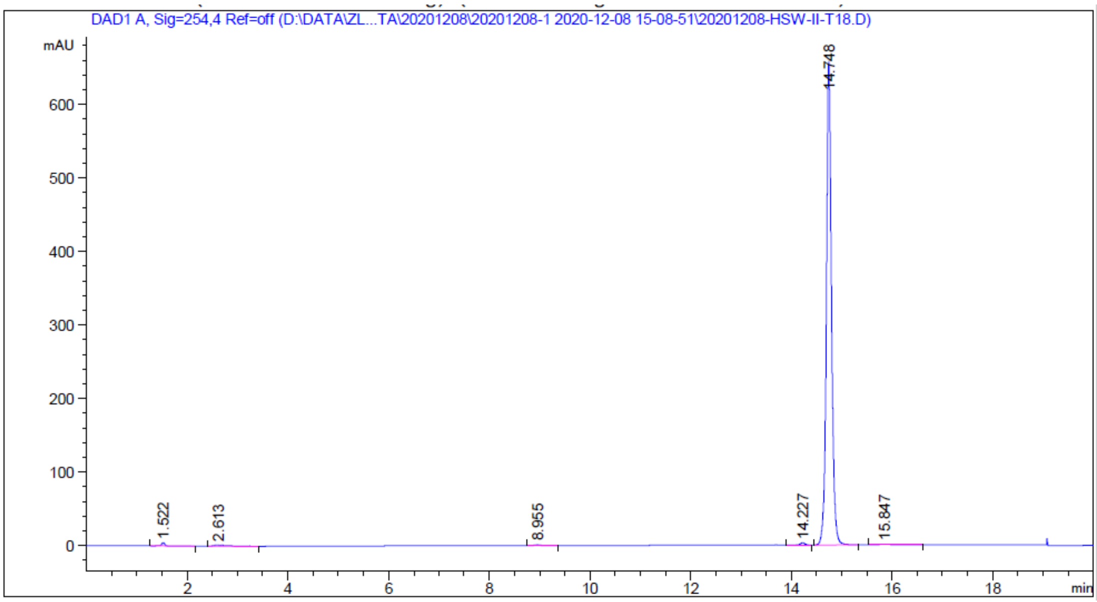

Signal 1: DAD1 A, Sig=254,4 Ref=off

Peak #	RetTime [min]	Type	Width [min]	Area [mAU*s]	Height [mAU]	Area %
1	1.522	BB	0.0944	21.35485	3.99437	0.4528
2	2.613	BB	0.2989	32.55546	1.46704	0.6903
3	8.955	BB	0.1446	8.86327	9.88915e-1	0.1879
4	14.227	BB	0.1176	20.53433	3.08887	0.4354
5	14.748	BB	0.1278	4613.32324	612.95648	97.8151

1260R 12/8/2020 9:11:52 PM BY

Data File D:\DATA\ZLM\DATA\20201208\20201208-1 2020-12-08 15-08-51\20201208-
Sample Name: 20201208-HSW-II-T18

Peak #	RetTime [min]	Type	Width [min]	Area [mAU*s]	Height [mAU]	Area %
6	15.847	BB	0.2736	19.73997	1.02069	0.4185
Totals :				4716.37113	623.51636	

^{1}H NMR, ^{13}C NMR, HRMS, and HPLC of compound B15

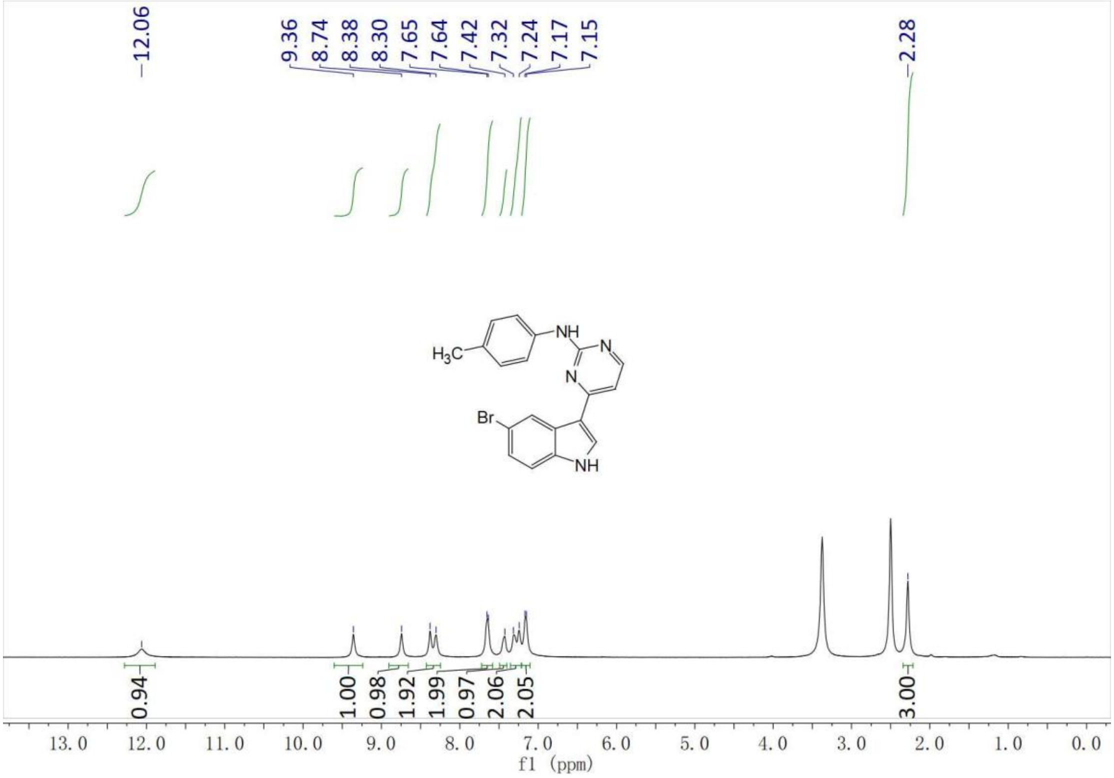

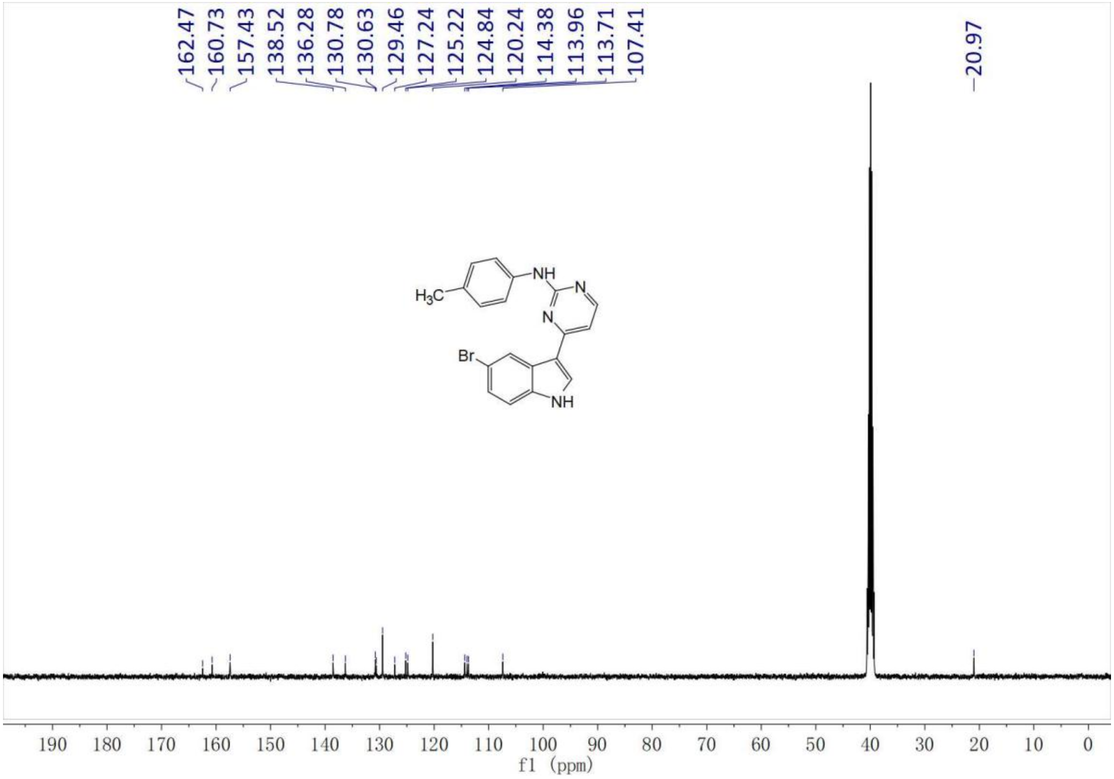

Display Report

Analysis Info | Acquisition Date 12/10/2020 16:34:14 PM

Sample Name TC3
Comment

Acquisition Parameter

Source Type	ESI	Ion Polarity	Positive	Set Nebulizer	2.0 Bar
Focus	Active	Set Capillary	4500 V	Set Dry Heater	200 °C
Scan Begin	50 m/z	Set End Plate Offset	-500 V	Set Dry Gas	8.0 l/min
Scan End	3000 m/z	Set Charging Voltage	2000 V	Set Divert Valve	Waste
		Set Corona	0 nA	Set APCI Heater	0 °C

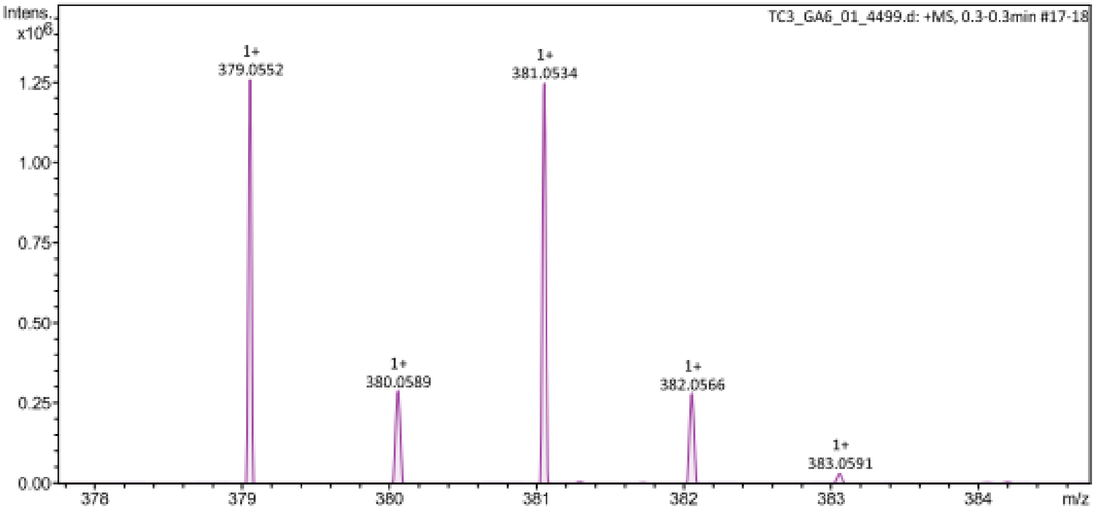

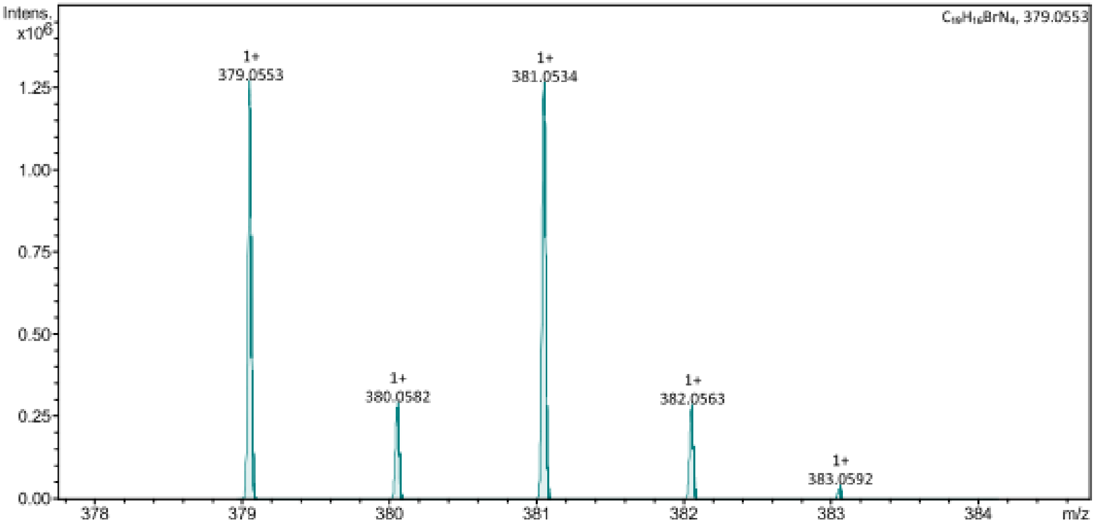

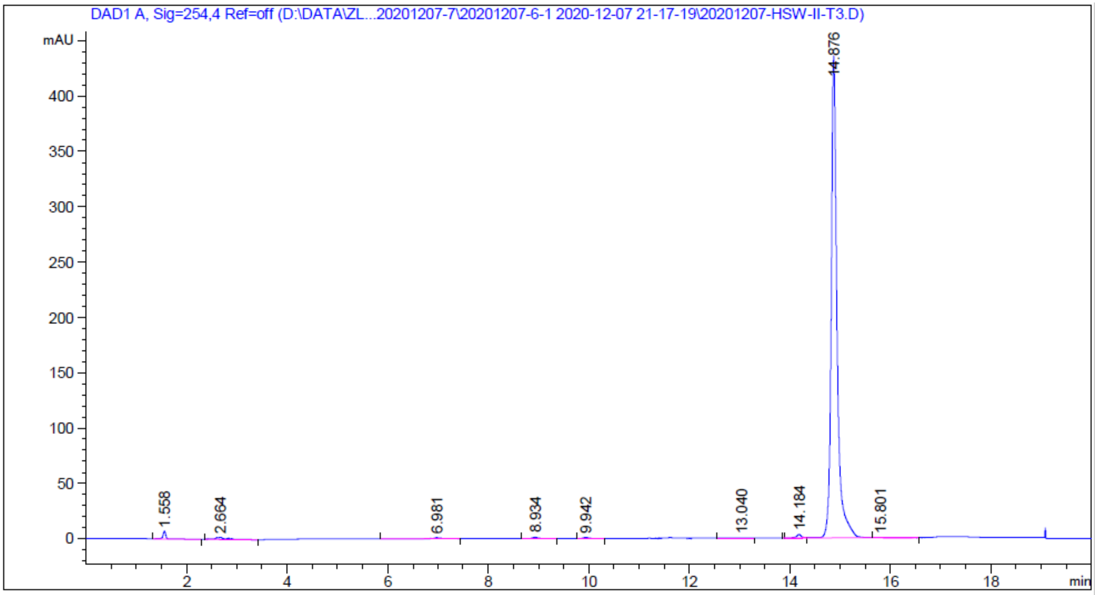

Peak #	RetTime [min]	Type	Width [min]	Area [mAU*s]	Height [mAU]	Area %
1	1.558	BB	0.0745	31.25010	6.68371	0.8569
2	2.664	BB	0.2701	32.42651	1.64433	0.8891
3	6.981	BB	0.1892	11.98472	9.12380e-1	0.3286
4	8.934	BB	0.1271	9.79710	1.10913	0.2686

1260R 12/8/2020 1:49:57 PM BY

Data File D:\DATA\ZL...TA\20201207-7\20201207-6-1 2020-12-07 21-17-19\20201207-HS
Sample Name: 20201207-HSW-II-T3

Peak #	RetTime [min]	Type	Width [min]	Area [mAU*s]	Height [mAU]	Area %
5	9.942	BB	0.1171	6.59760	8.63425e-1	0.1809
6	13.040	BB	0.1860	3.71901	2.74684e-1	0.1020
7	14.184	BV E	0.0985	19.69197	3.08508	0.5400
8	14.876	VV R	0.1226	3524.91284	434.79910	96.6541
9	15.801	VB E	0.3321	6.55638	2.51518e-1	0.1798
Totals :				3646.93623	449.62336	

^{1}H NMR, ^{13}C NMR, HRMS, and HPLC of compound B16

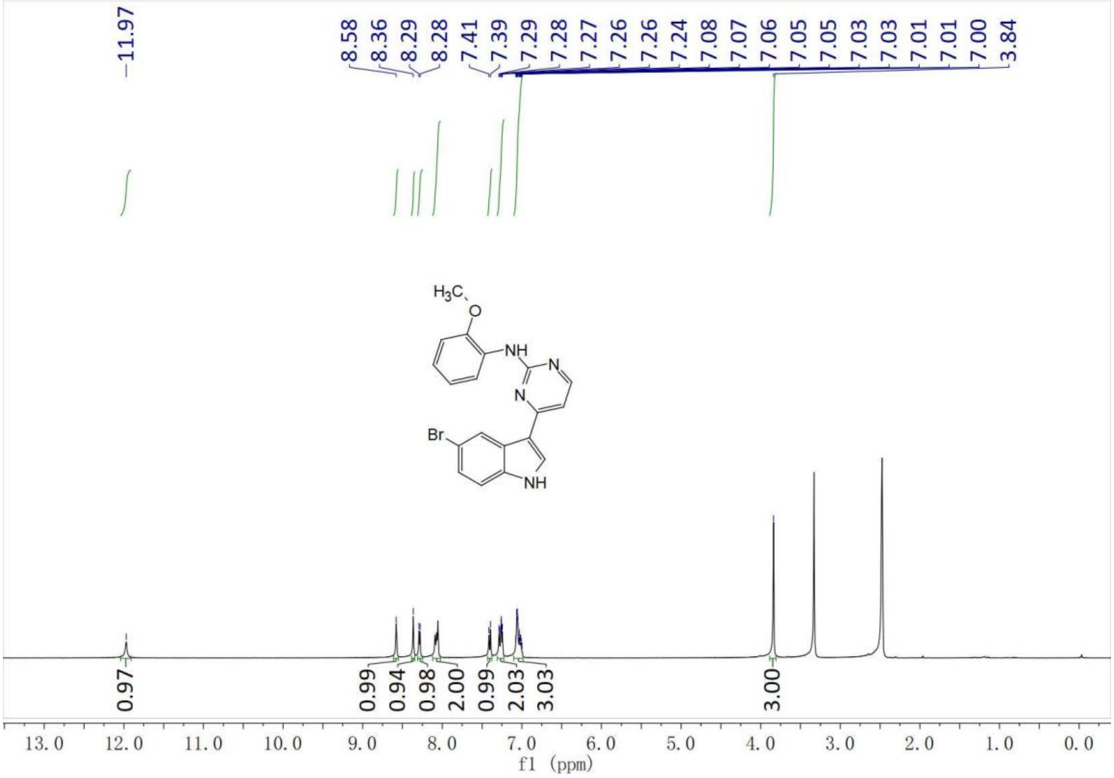

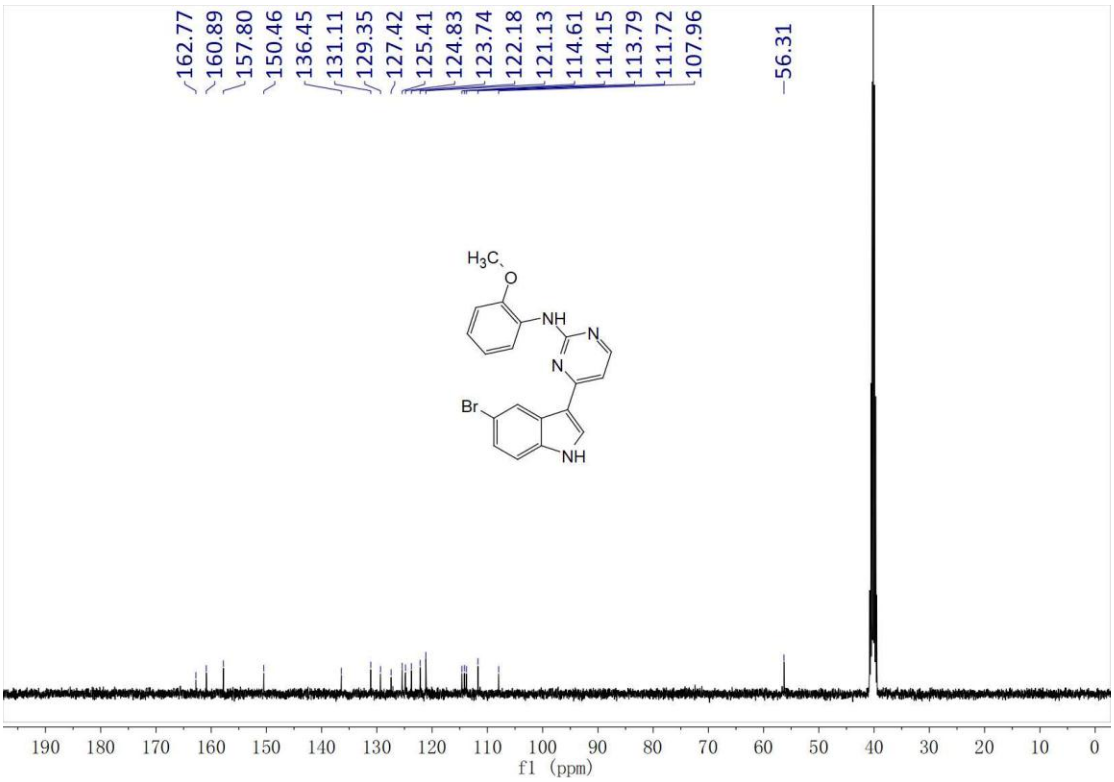

Display Report

Analysis Info Acquisition Date 12/10/2020 17:47:37 PM

Sample Name TC31
Comment

Acquisition Parameter

Source Type	ESI	Ion Polarity	Positive	Set Nebulizer	2.0 Bar
Focus	Active	Set Capillary	4500 V	Set Dry Heater	200 °C
Scan Begin	50 m/z	Set End Plate Offset	-500 V	Set Dry Gas	8.0 l/min
Scan End	3000 m/z	Set Charging Voltage	2000 V	Set Divert Valve	Waste
		Set Corona	0 nA	Set APCI Heater	0 °C

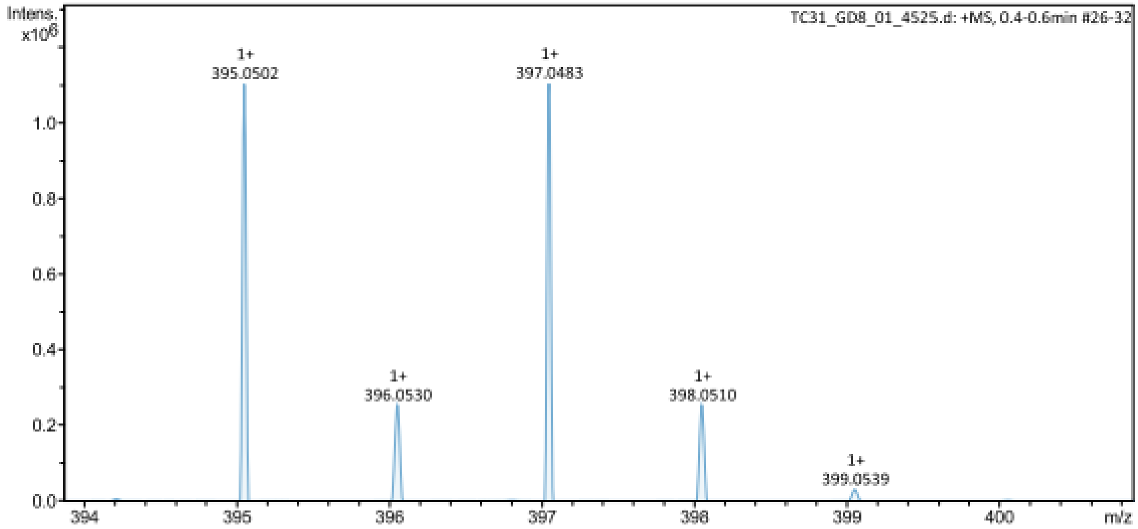

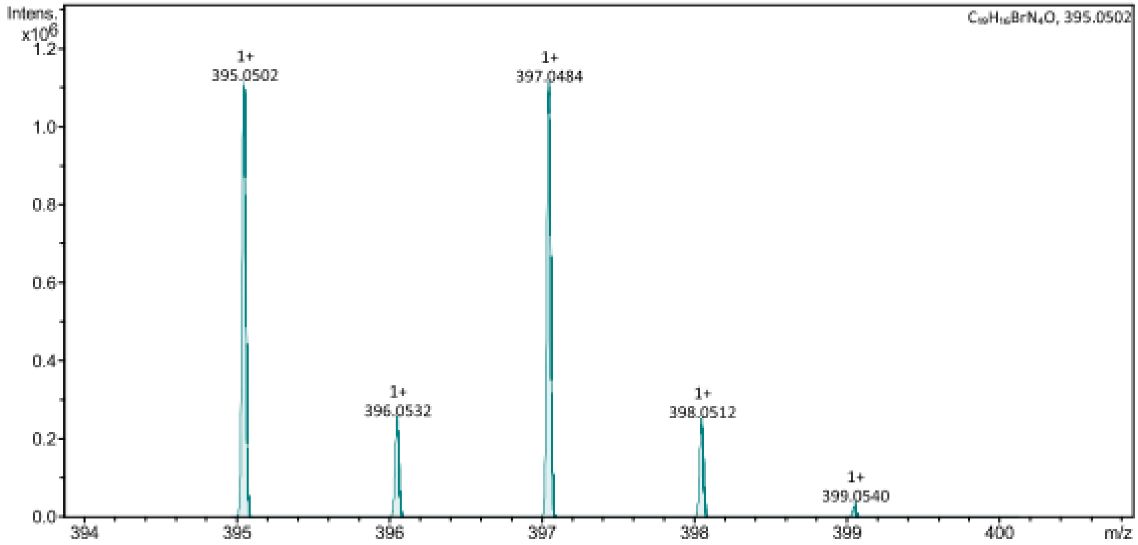

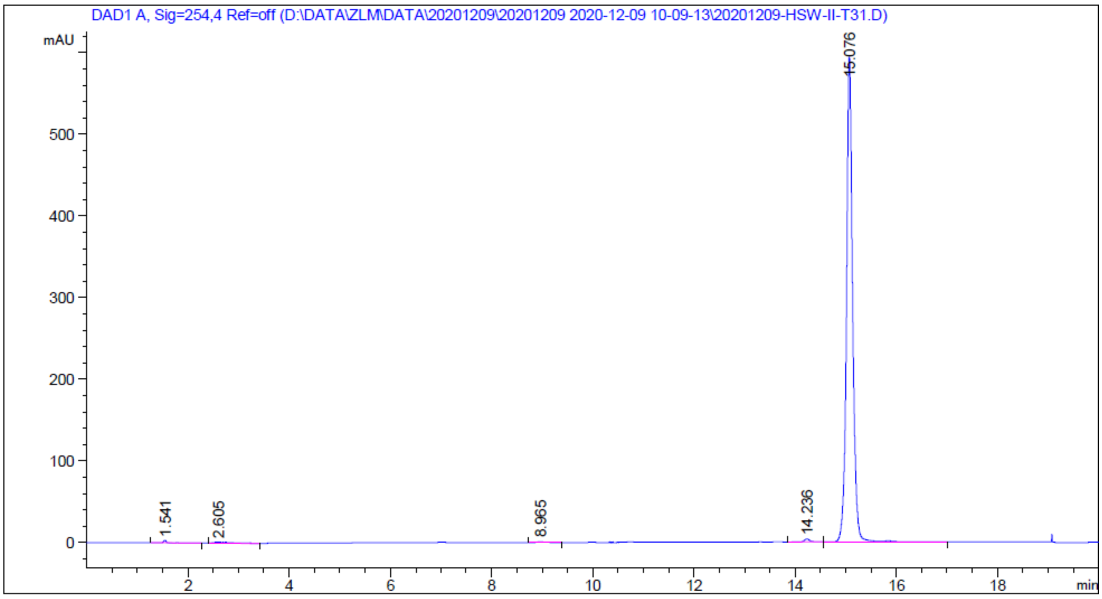

Signal 1: DAD1 A, Sig=254,4 Ref=off

Peak #	RetTime [min]	Type	Width [min]	Area [mAU*s]	Height [mAU]	Area %
1	1.541	BB	0.0982	15.33867	2.70330	0.3020
2	2.605	BB	0.3473	32.69539	1.27441	0.6437
3	8.965	BB	0.1427	10.10086	1.14785	0.1989

1260R 12/9/2020 5:59:22 PM BY

Data File D:\DATA\ZLM\DATA\20201209\20201209 2020-12-09 10-09-13\20201209-H
Sample Name: 20201209-HSW-II-T31

Peak #	RetTime [min]	Type	Width [min]	Area [mAU*s]	Height [mAU]	Area %
4	14.236	BV	0.1340	30.99158	3.84817	0.6101
5	15.076	VV R	0.1402	4990.44678	580.80328	98.2454

Totals : 5079.57328 589.77701

^{1}H NMR, ^{13}C NMR, HRMS, and HPLC of compound B17

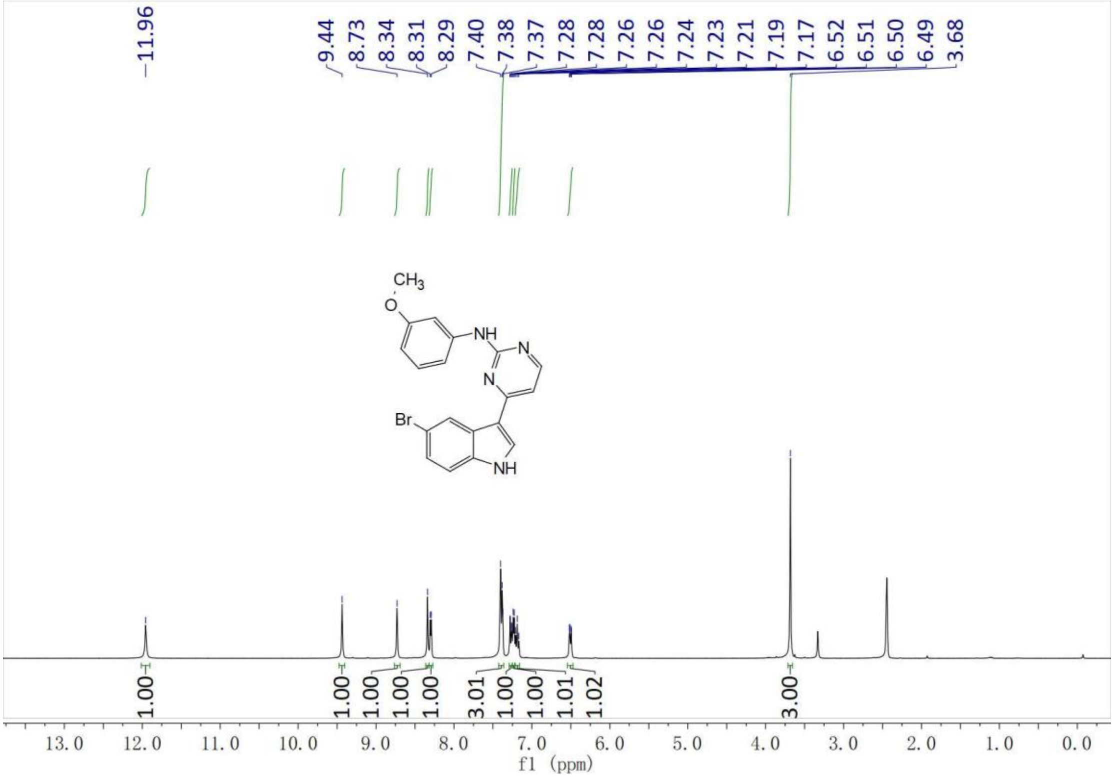

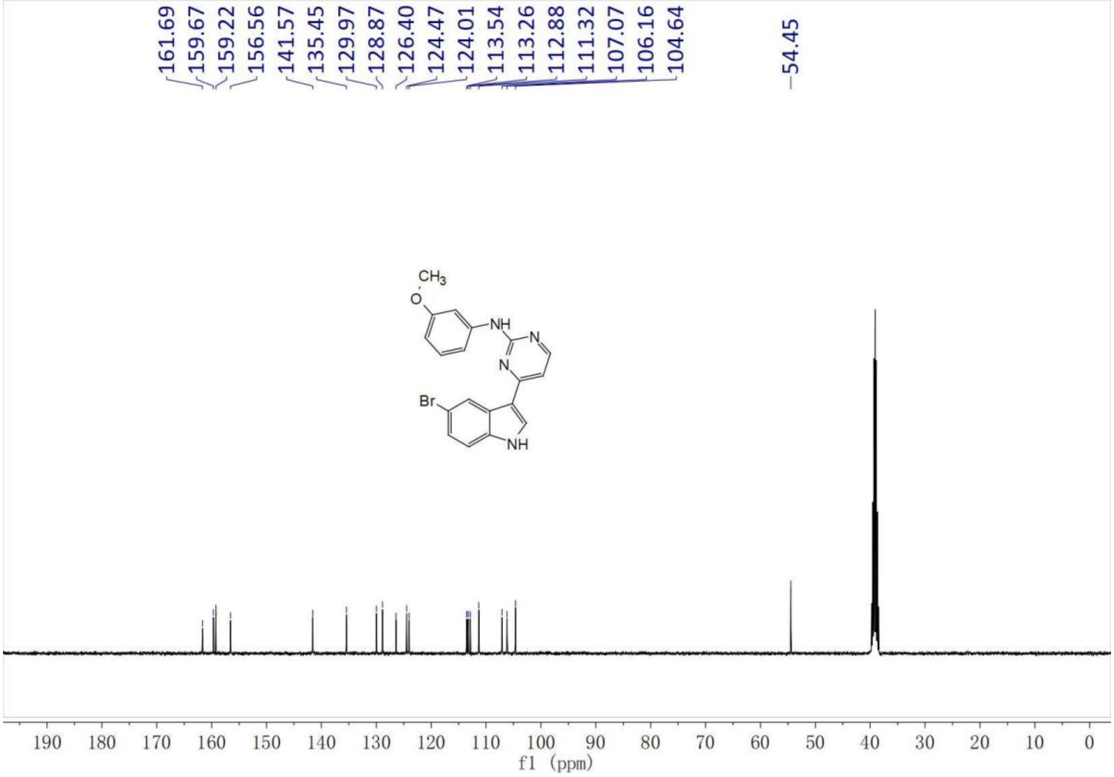

Display Report

Analysis Info

Acquisition Date 12/10/2020 17:21:59 PM

Sample Name TC20
Comment

Acquisition Parameter

Source Type	ESI	Ion Polarity	Positive	Set Nebulizer	2.0 Bar
Focus	Active	Set Capillary	4500 V	Set Dry Heater	200 °C
Scan Begin	50 m/z	Set End Plate Offset	-500 V	Set Dry Gas	8.0 l/min
Scan End	3000 m/z	Set Charging Voltage	2000 V	Set Divert Valve	Waste
		Set Corona	0 nA	Set APCI Heater	0 °C

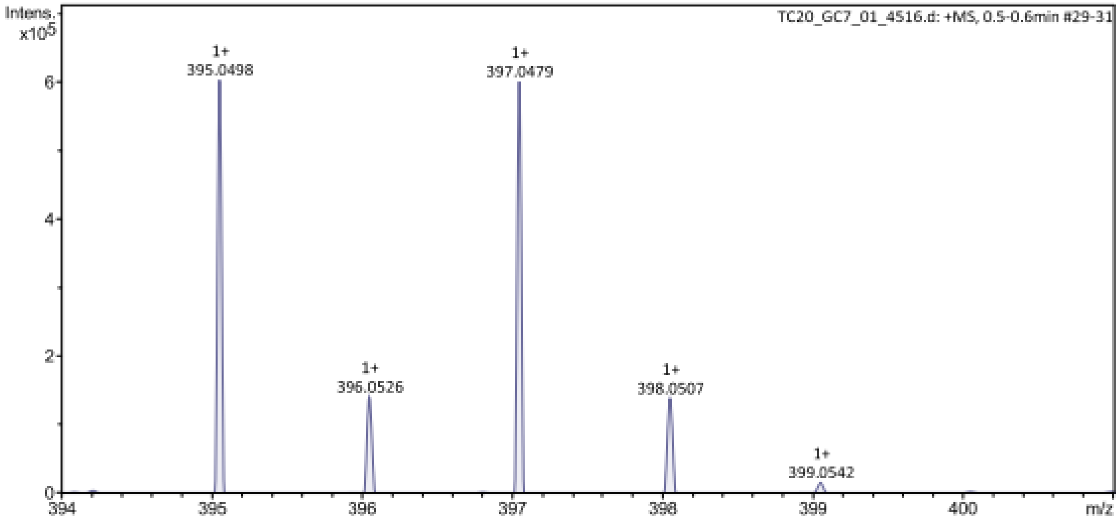

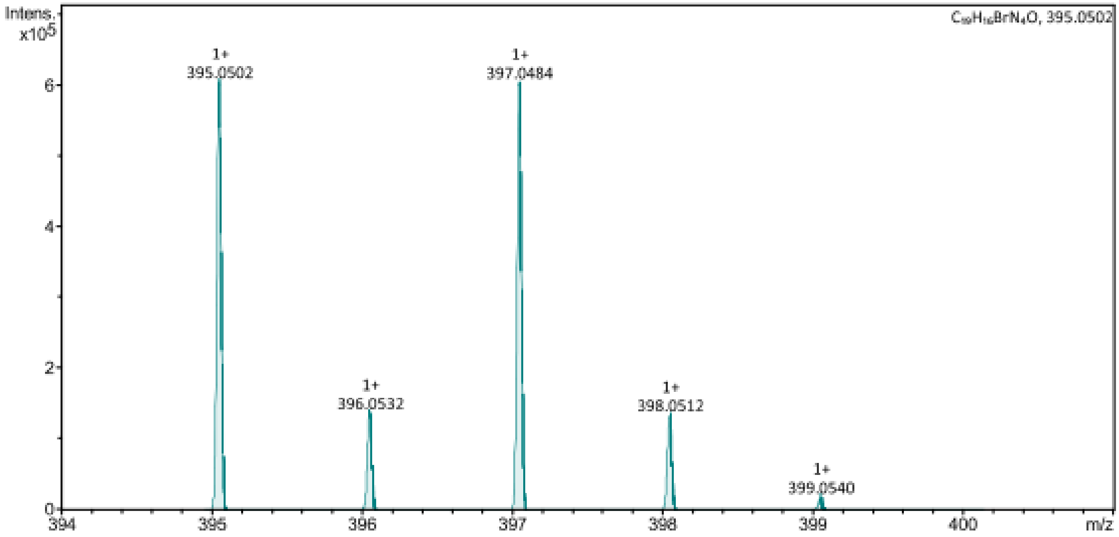

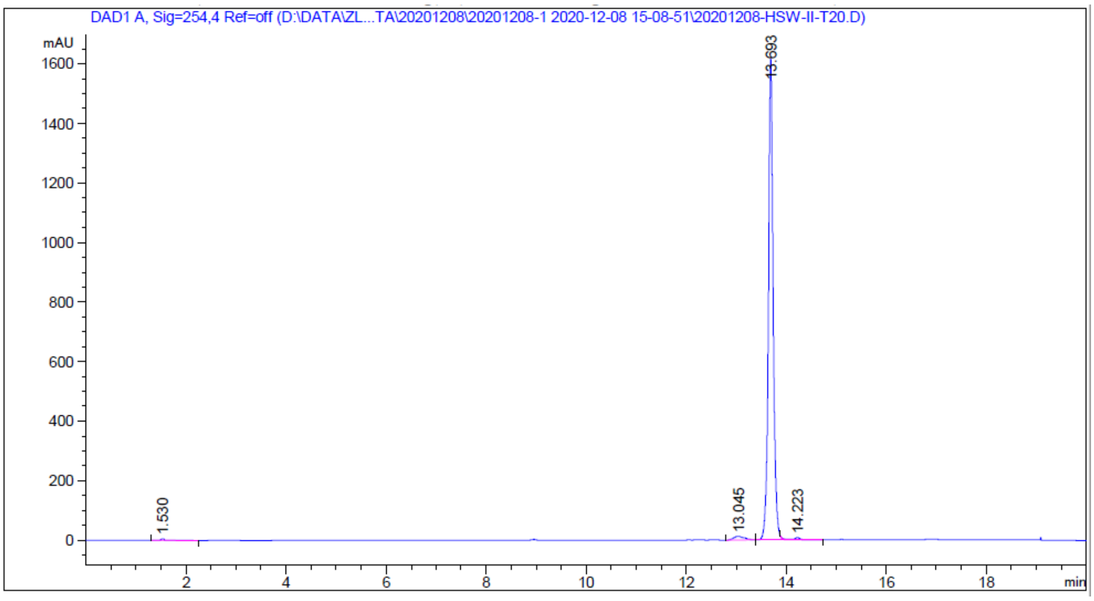

Signal 1: DAD1 A, Sig=254,4 Ref=off

Peak #	RetTime [min]	Type	Width [min]	Area [mAU*s]	Height [mAU]	Area %
1	1.530	BB	0.0954	26.40211	4.85923	0.2398
2	13.045	BV	0.2456	176.52936	11.86654	1.6033
3	13.693	VV R	0.1194	1.07282e4	1576.44006	97.4386
4	14.223	VB E	0.1479	79.08117	7.96004	0.7183

1260R 12/8/2020 9:24:31 PM BY

Data File D:\DATA\ZLM\DATA\20201208\20201208-1 2020-12-08 15-08-51\202[illegible]
Sample Name: 20201208-HSW-II-T20

Totals : 1.10102e4 1601.12587

^{1}H NMR, ^{13}C NMR, HRMS, and HPLC of compound B18

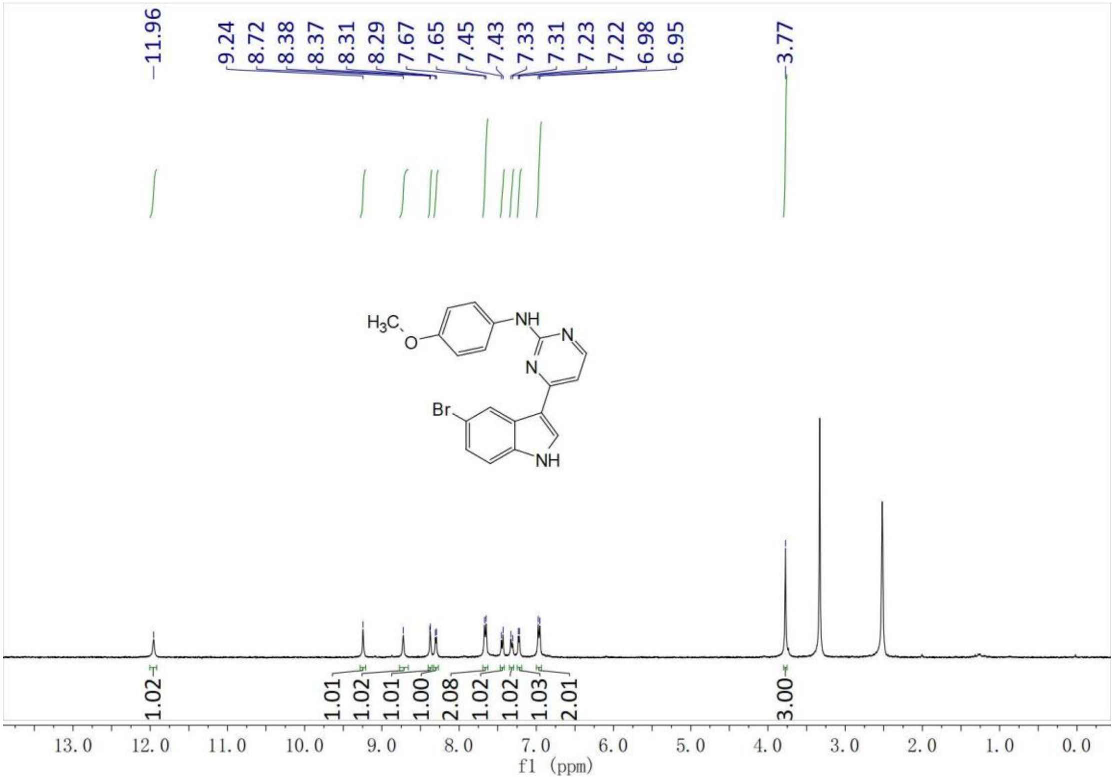

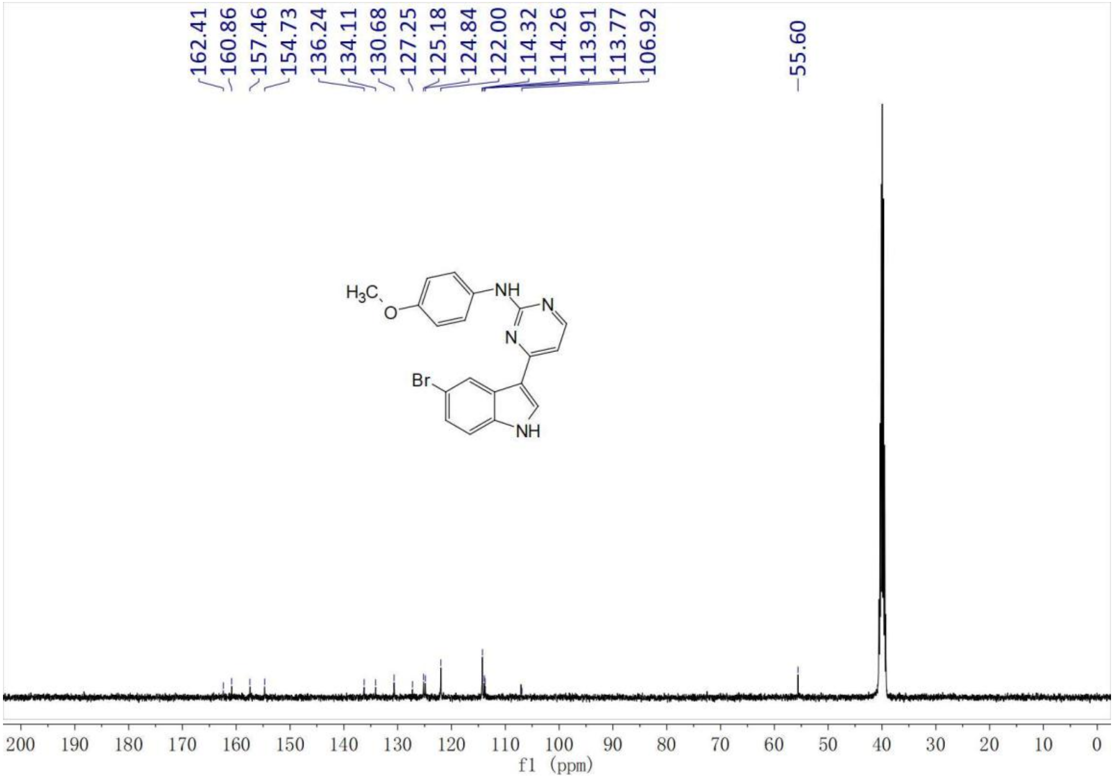

Display Report

Analysis Info Acquisition Date 12/10/2020 16:36:59 PM

Sample Name TC4
Comment

Acquisition Parameter

Source Type	ESI	Ion Polarity	Positive	Set Nebulizer	2.0 Bar
Focus	Active	Set Capillary	4500 V	Set Dry Heater	200 °C
Scan Begin	50 m/z	Set End Plate Offset	-500 V	Set Dry Gas	8.0 l/min
Scan End	3000 m/z	Set Charging Voltage	2000 V	Set Divert Valve	Waste
		Set Corona	0 nA	Set APCI Heater	0 °C

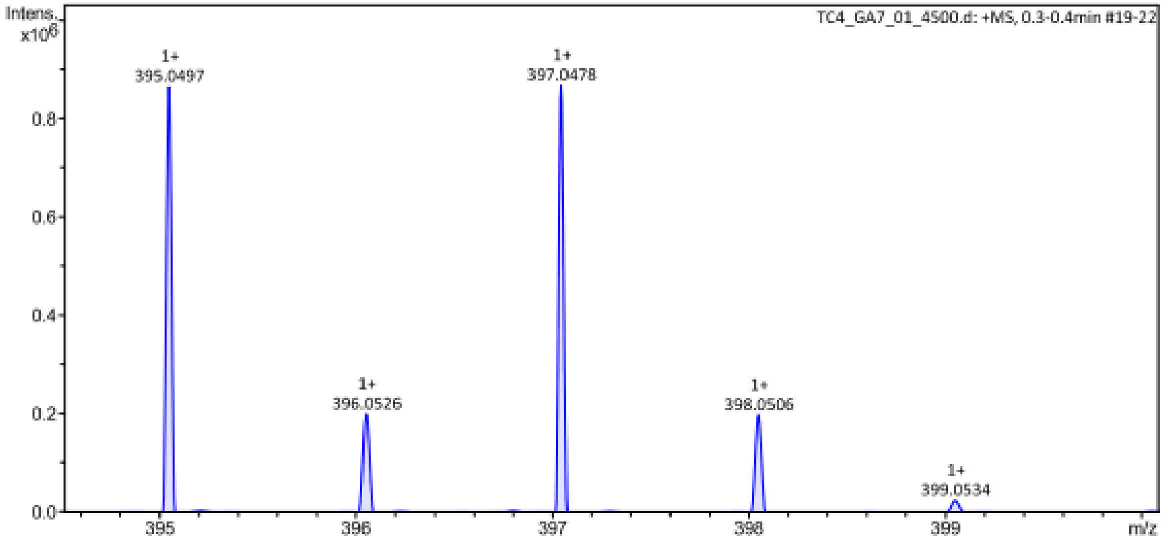

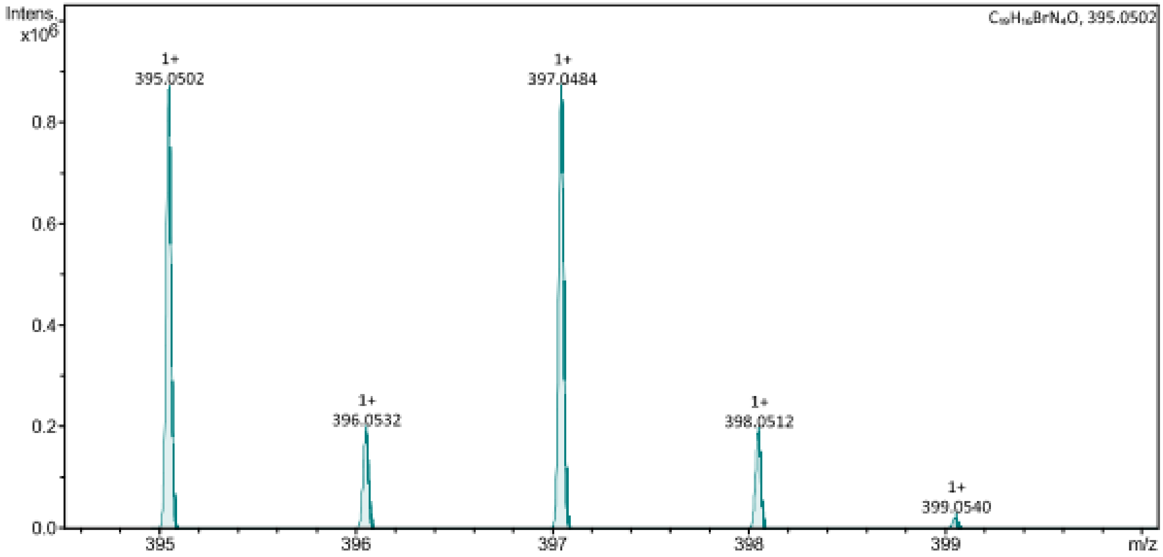

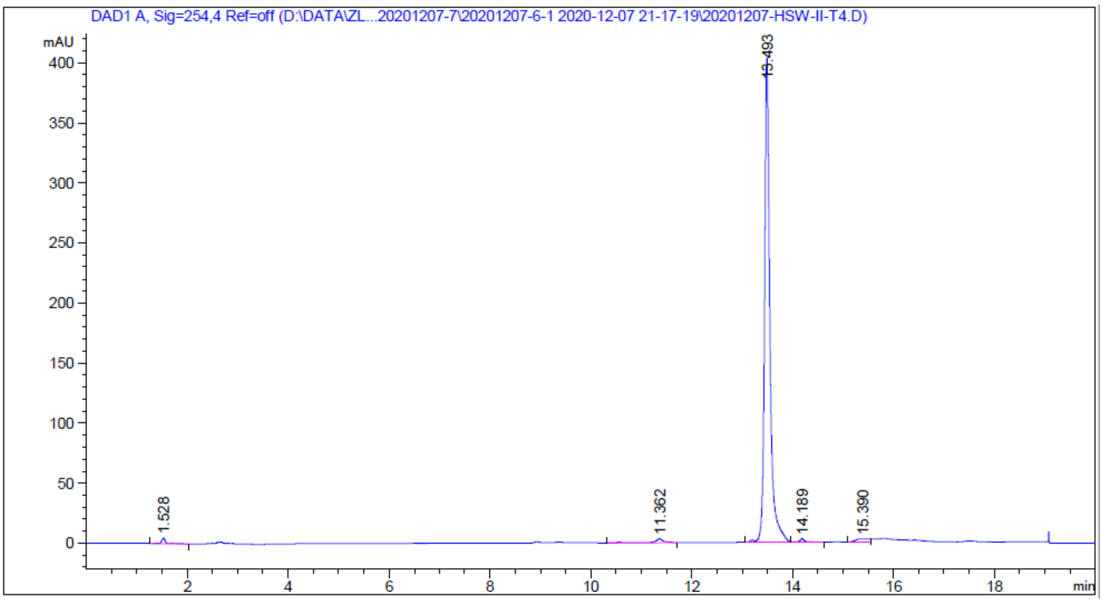

Signal 1: DAD1 A, Sig=254,4 Ref=off

Peak #	RetTime [min]	Type	Width [min]	Area [mAU*s]	Height [mAU]	Area %
1	1.528	BB	0.0806	23.27741	4.47883	0.7483
2	11.362	VB R	0.1621	41.41161	3.30476	1.3313
3	13.493	BV R	0.1101	2974.28345	402.80151	95.6179
4	14.189	VB E	0.1094	21.91028	2.99455	0.7044

1260R 12/8/2020 2:01:34 PM BY

Data File D:\DATA\ZL...TA\20201207-7\20201207-6-1 2020-12-07 21-17-19
Sample Name: 20201207-HSW-II-T4

Peak #	RetTime [min]	Type	Width [min]	Area [mAU*s]	Height [mAU]	Area %
5	15.390	BV	0.2768	49.71017	2.72491	1.5981

Totals : 3110.59292 416.30457

^{1}**H NMR,** ^{13}**C NMR, HRMS, and HPLC of compound B19**

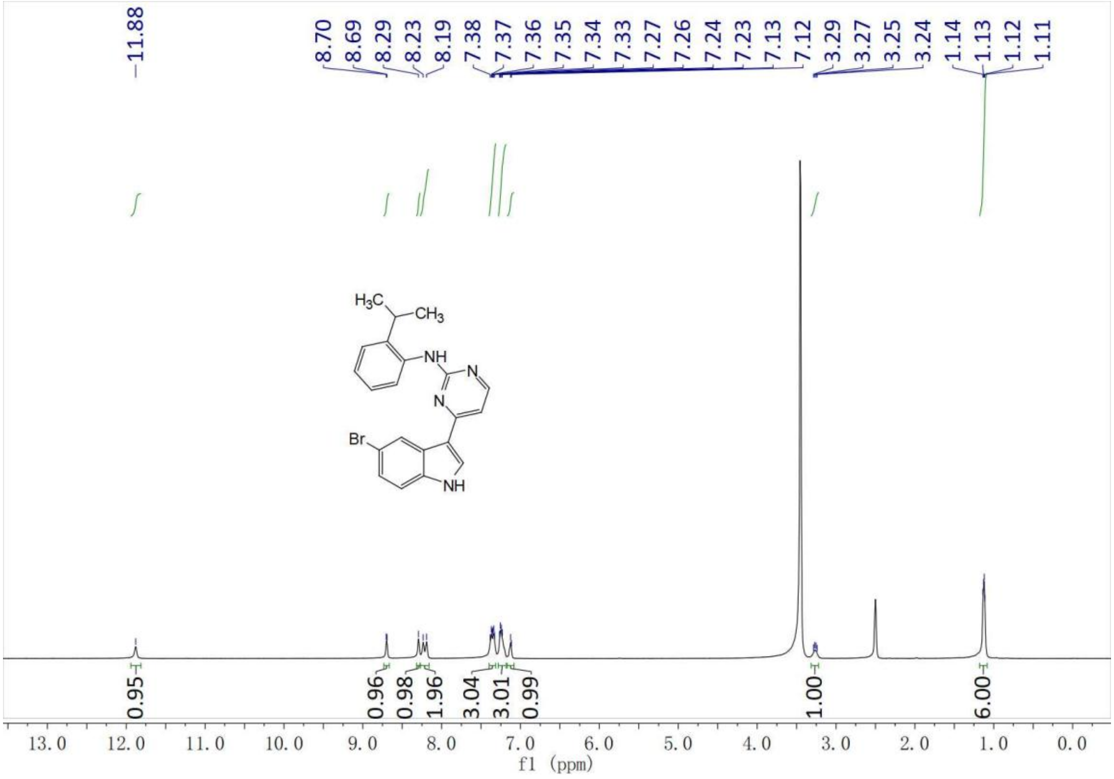

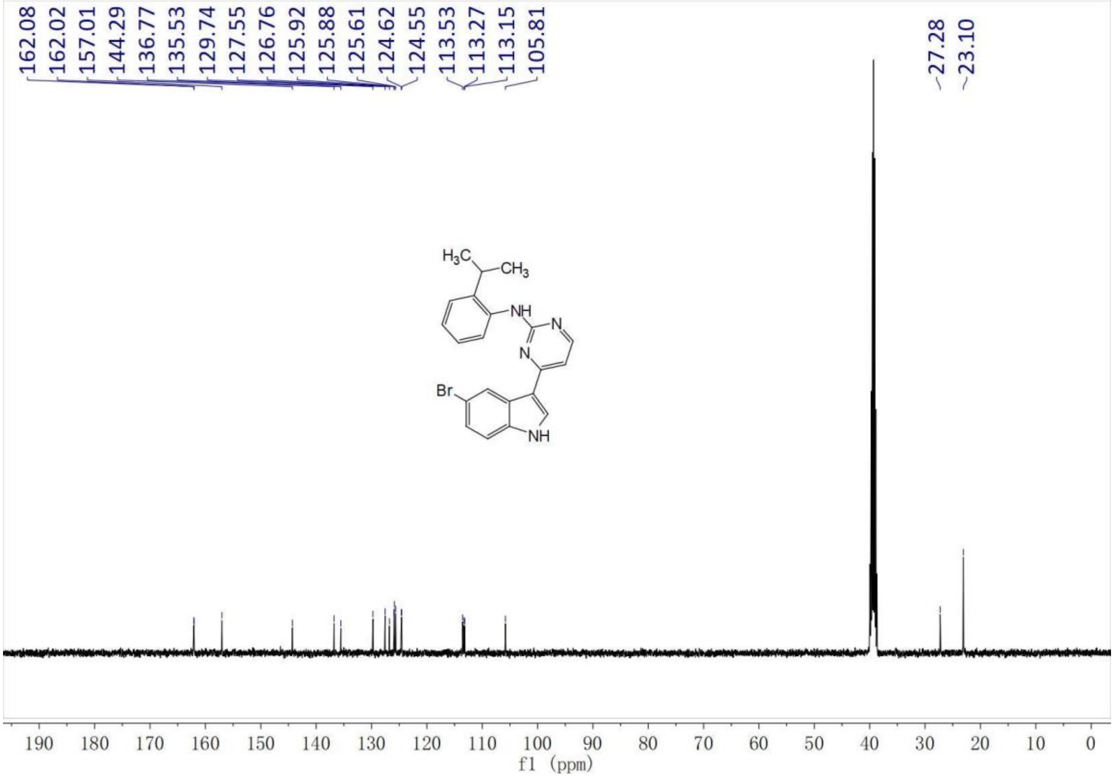

Display Report

Analysis Info

Acquisition Date 12/10/2020 17:33:26 PM

Sample Name TC26
Comment

Acquisition Parameter

Source Type	ESI	Ion Polarity	Positive	Set Nebulizer	2.0 Bar
Focus	Active	Set Capillary	4500 V	Set Dry Heater	200 °C
Scan Begin	50 m/z	Set End Plate Offset	-500 V	Set Dry Gas	8.0 l/min
Scan End	3000 m/z	Set Charging Voltage	2000 V	Set Divert Valve	Waste
		Set Corona	0 nA	Set APCI Heater	0 °C

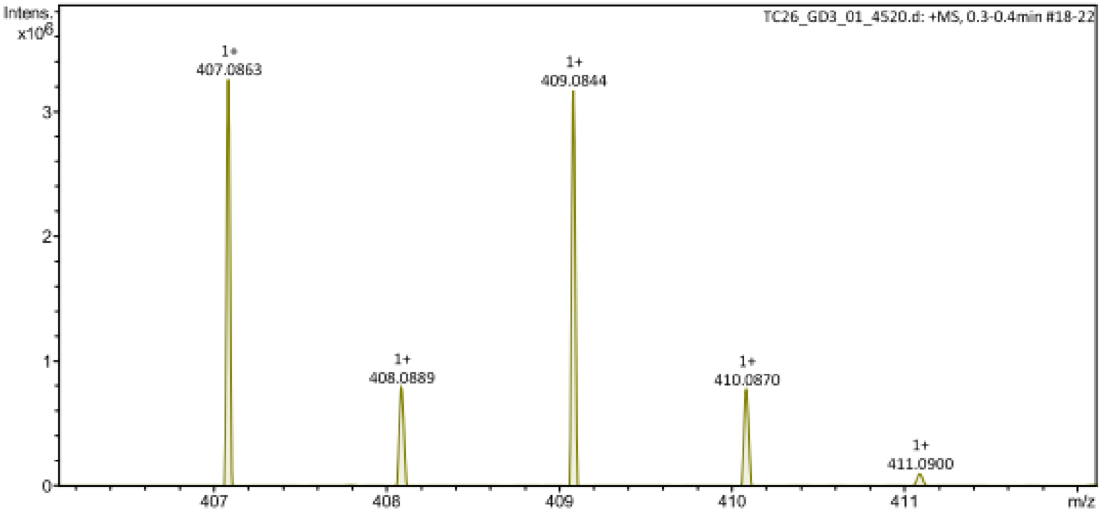

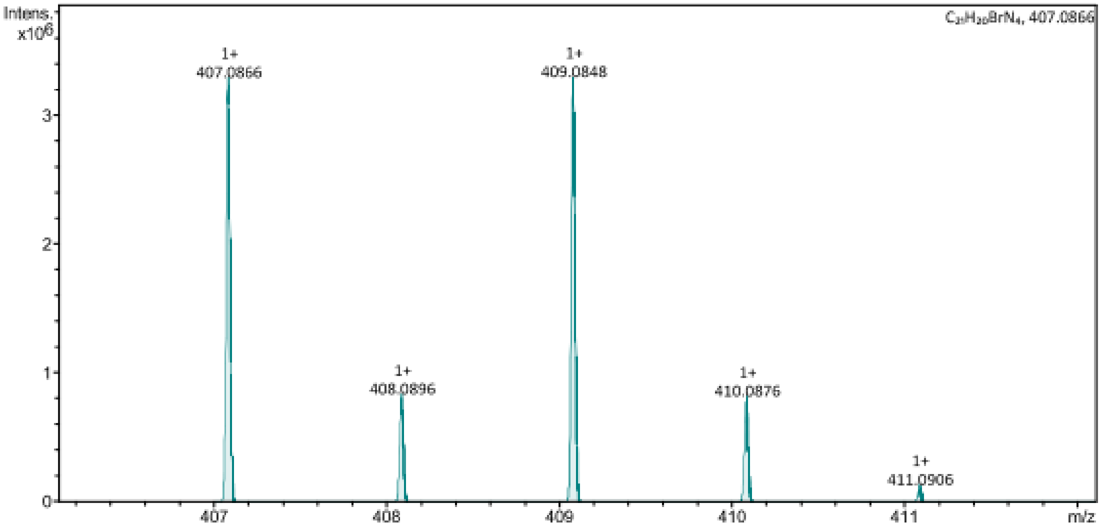

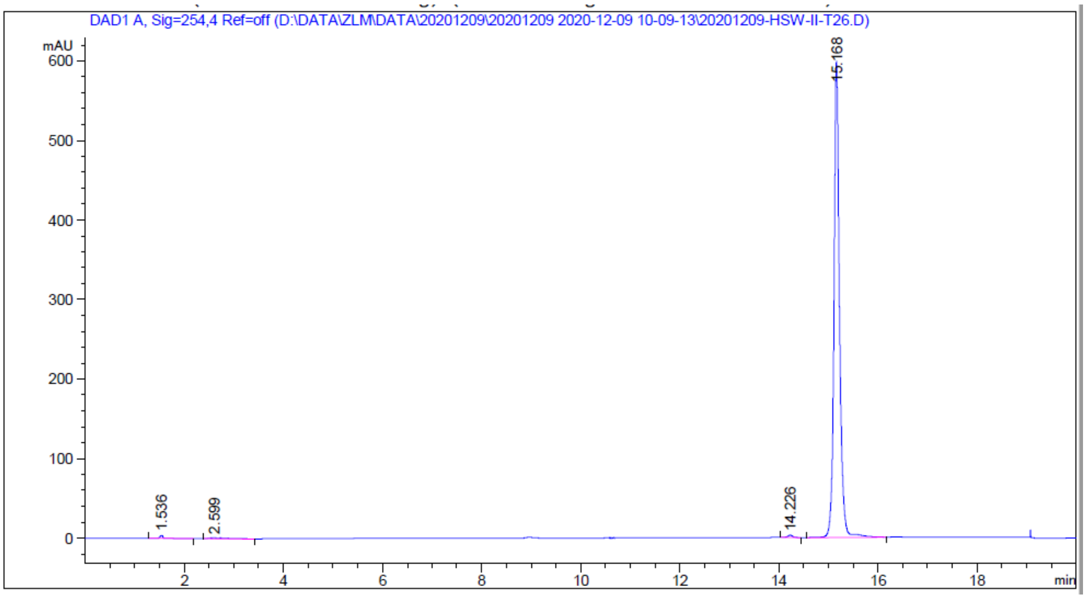

Signal 1: DAD1 A, Sig=254,4 Ref=off

Peak #	RetTime [min]	Type	Width [min]	Area [mAU*s]	Height [mAU]	Area %
1	1.536	BB	0.1038	18.56627	3.39757	0.3952
2	2.599	BB	0.3517	30.34419	1.16552	0.6459
3	14.226	BB	0.1229	20.65141	2.90624	0.4396
4	15.168	BB	0.1314	4628.65918	590.98102	98.5194

Totals : 4698.22106 598.45035

^{1}H NMR, ^{13}C NMR, HRMS, and HPLC of compound B20

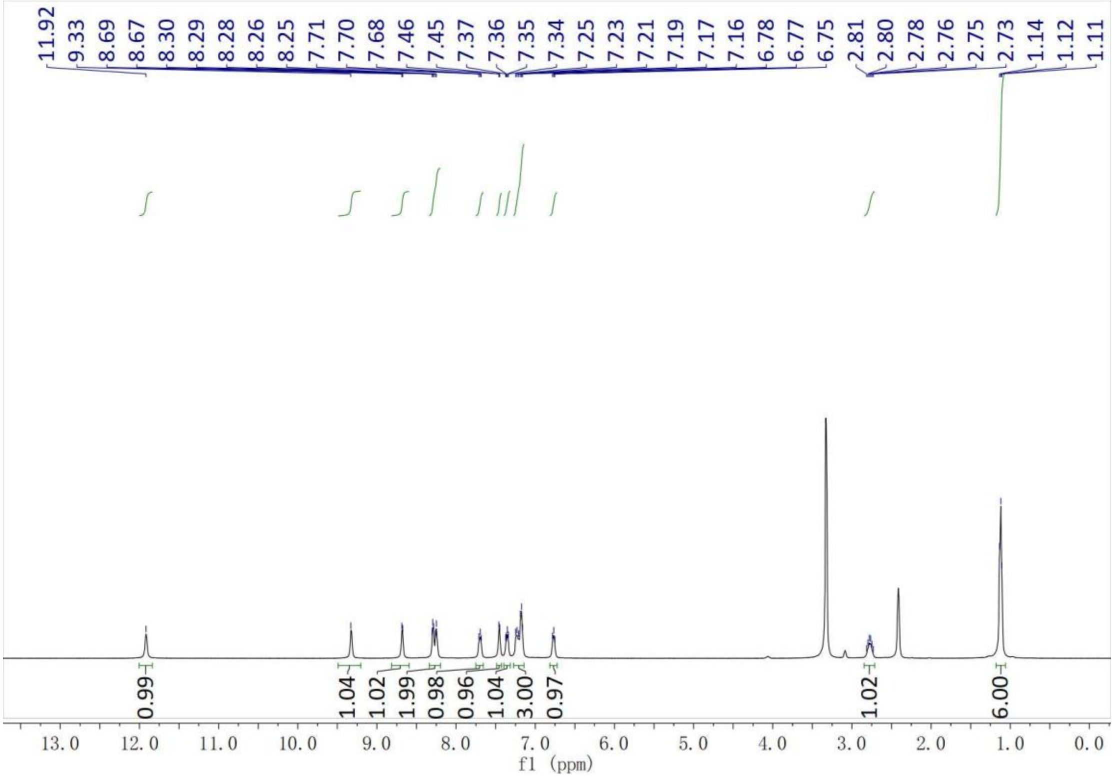

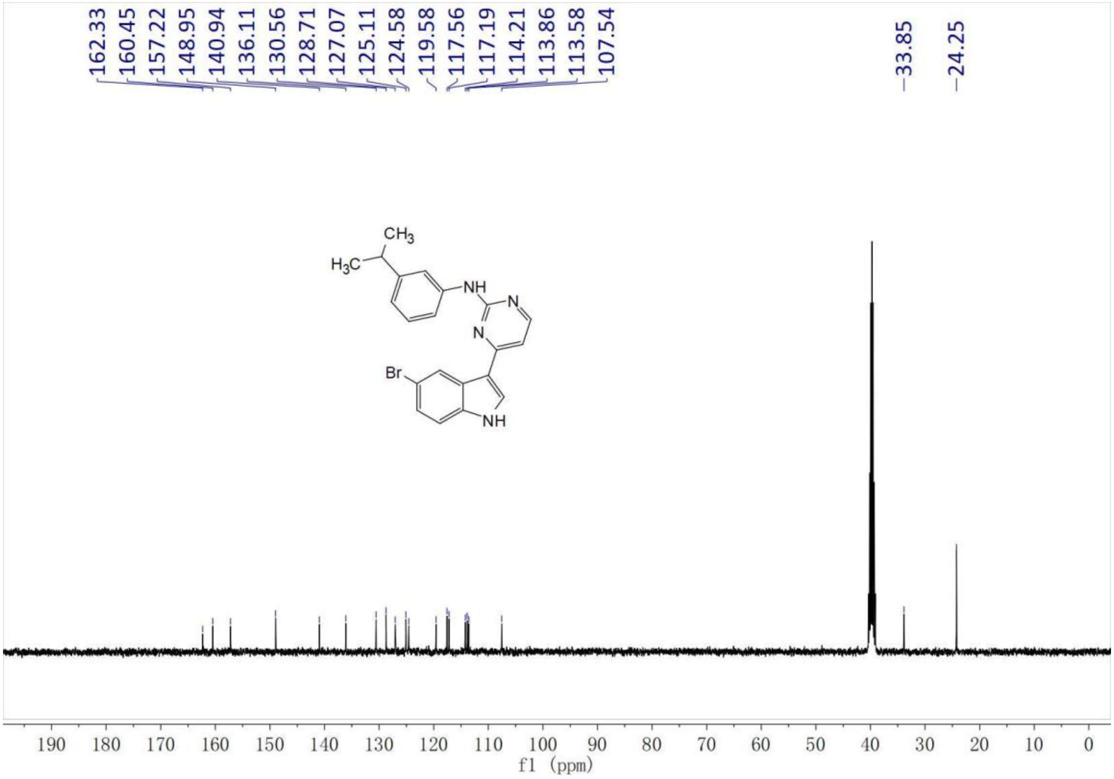

Display Report

Analysis Info Acquisition Date 12/10/2020 17:41:42 PM

Sample Name TC29
Comment

Acquisition Parameter

Source Type	ESI	Ion Polarity	Positive	Set Nebulizer	2.0 Bar
Focus	Active	Set Capillary	4500 V	Set Dry Heater	200 °C
Scan Begin	50 m/z	Set End Plate Offset	-500 V	Set Dry Gas	8.0 l/min
Scan End	3000 m/z	Set Charging Voltage	2000 V	Set Divert Valve	Waste
		Set Corona	0 nA	Set APCI Heater	0 °C

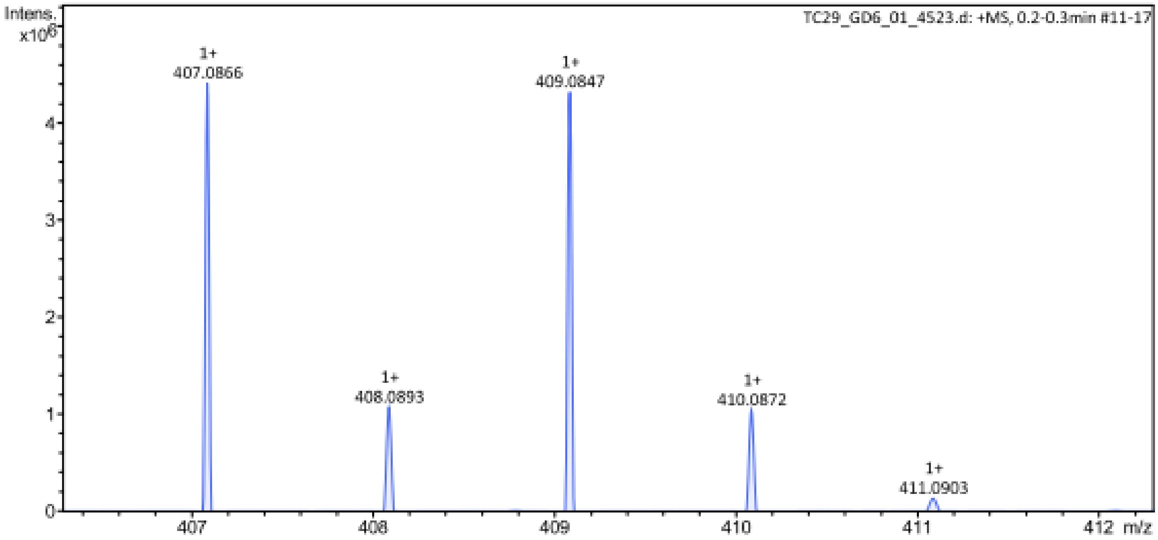

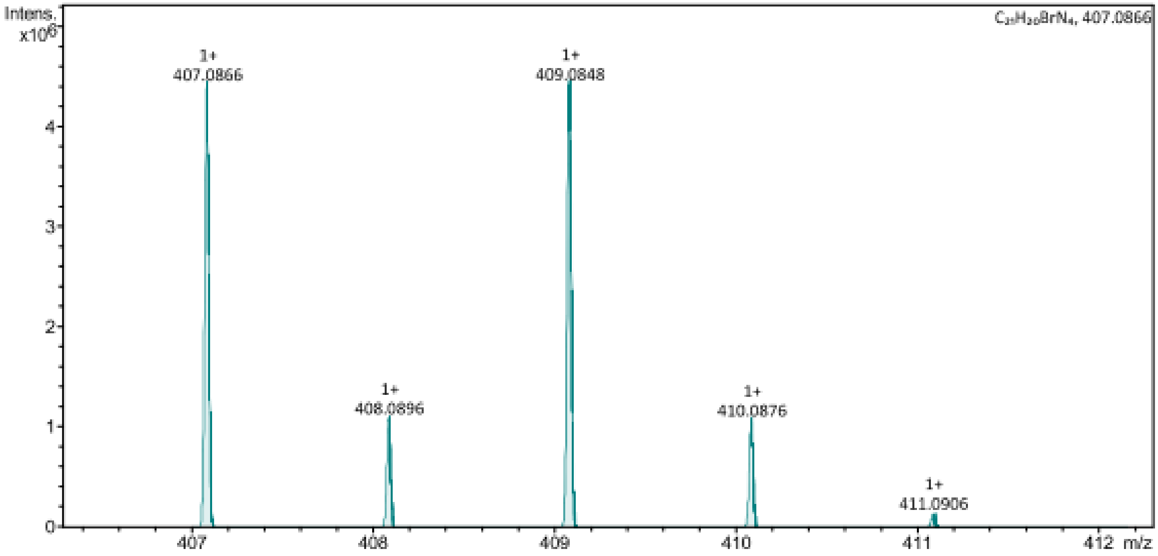

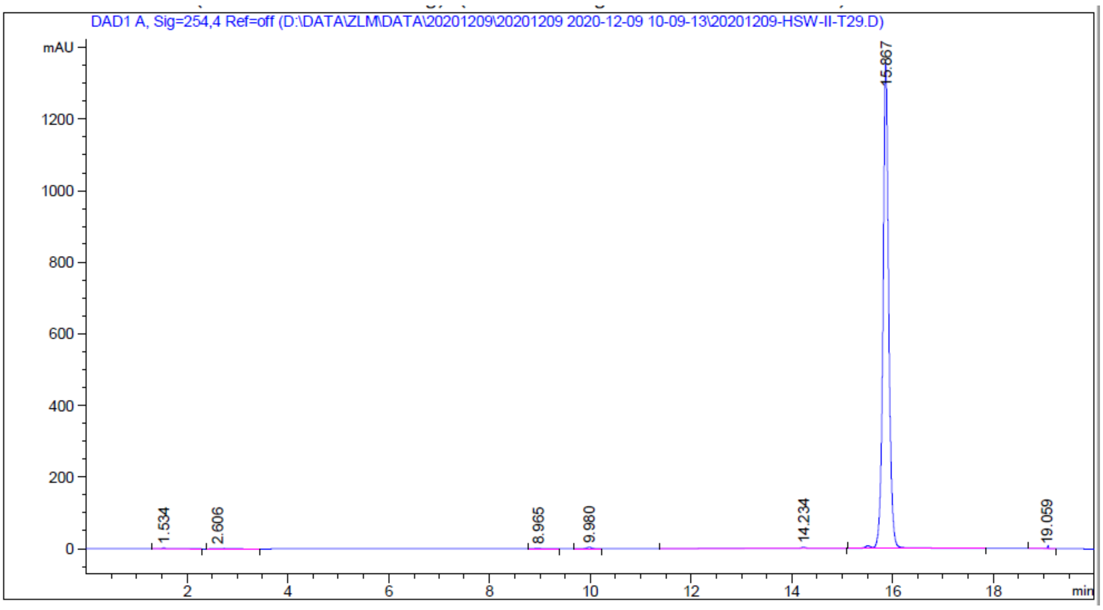

Signal 1: DAD1 A, Sig=254,4 Ref=off

Peak #	RetTime [min]	Type	Width [min]	Area [mAU*s]	Height [mAU]	Area %
1	1.534	BB	0.1058	12.46664	2.20905	0.1147
2	2.606	BB	0.3497	31.47375	1.21722	0.2897
3	8.965	BB	0.1387	9.20721	1.08818	0.0847
4	9.980	BB	0.1175	23.24305	3.49888	0.2139
5	14.234	BB	0.2615	53.08180	2.79594	0.4885
6	15.867	BB	0.1458	1.07035e4	1277.04614	98.5032

1260R 12/9/2020 1:41:09 PM BY

Data File D:\DATA\ZLM\DATA\20201209\20201209 2020-12-09 10-09-13\202012(
Sample Name: 20201209-HSW-II-T29

Peak #	RetTime [min]	Type	Width [min]	Area [mAU*s]	Height [mAU]	Area %
7	19.059	VBA	0.2983	33.17587	1.45327	0.3053
Totals :				1.08661e4	1289.30868	

^{1}H NMR, ^{13}C NMR, HRMS, and HPLC of compound B21

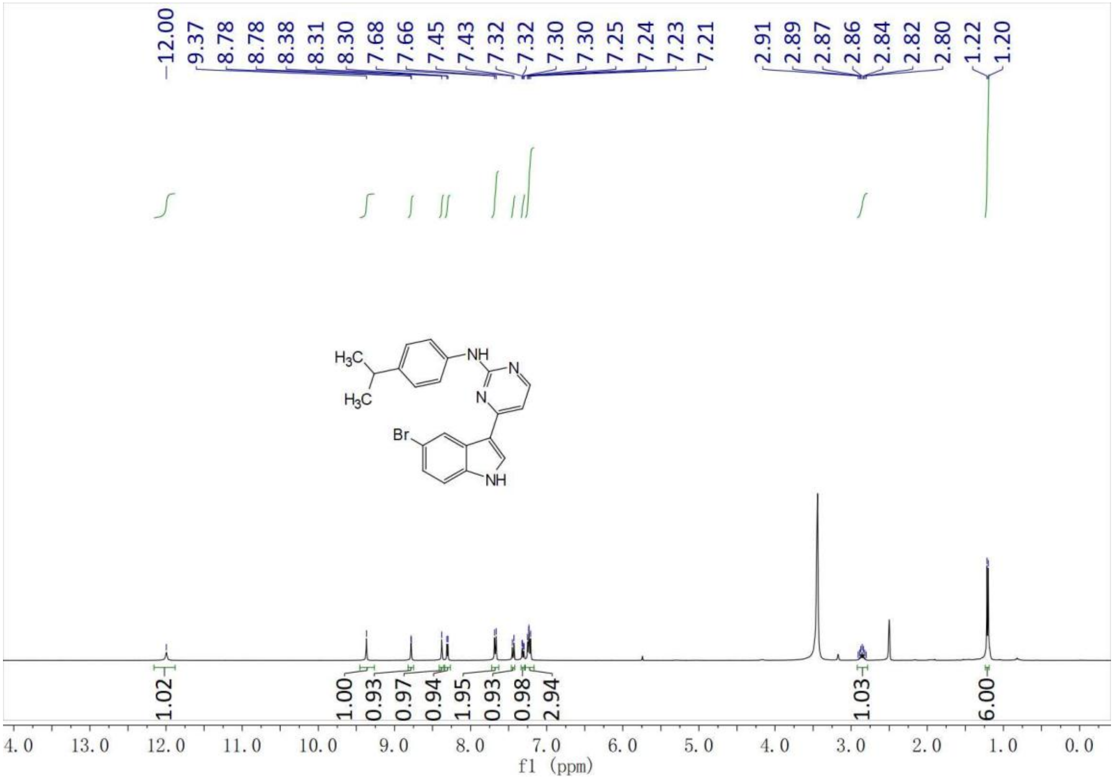

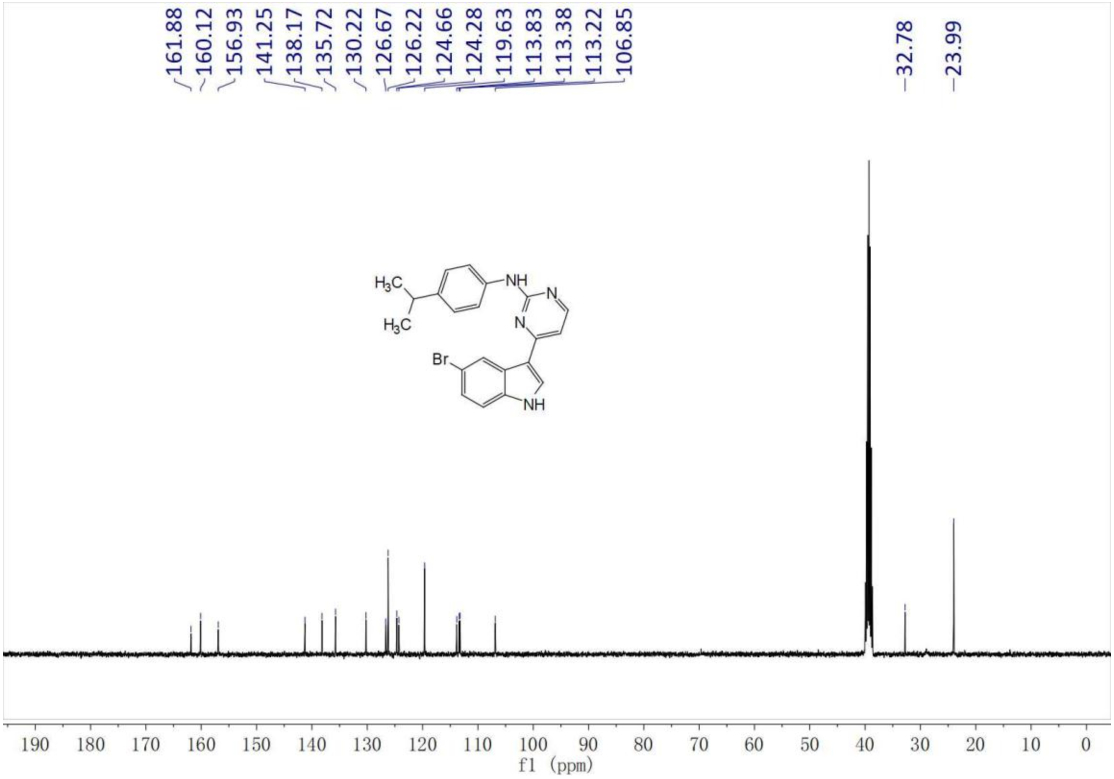

Display Report

Analysis Info

Acquisition Date 12/10/2020 17:30:39 PM

Sample Name TC25
Comment

Acquisition Parameter

Source Type	ESI	Ion Polarity	Positive	Set Nebulizer	2.0 Bar
Focus	Active	Set Capillary	4500 V	Set Dry Heater	200 °C
Scan Begin	50 m/z	Set End Plate Offset	-500 V	Set Dry Gas	8.0 l/min
Scan End	3000 m/z	Set Charging Voltage	2000 V	Set Divert Valve	Waste
		Set Corona	0 nA	Set APCI Heater	0 °C

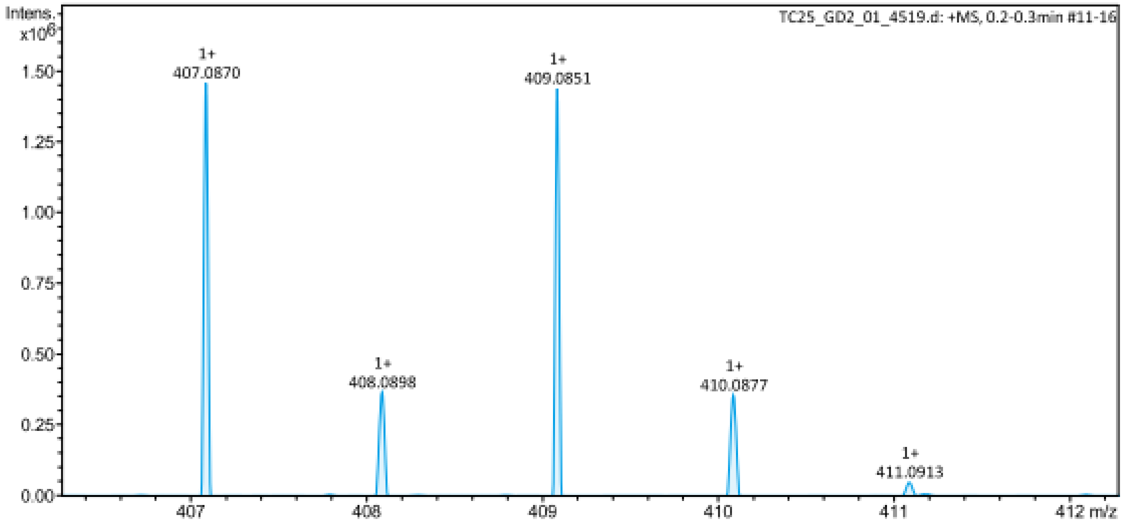

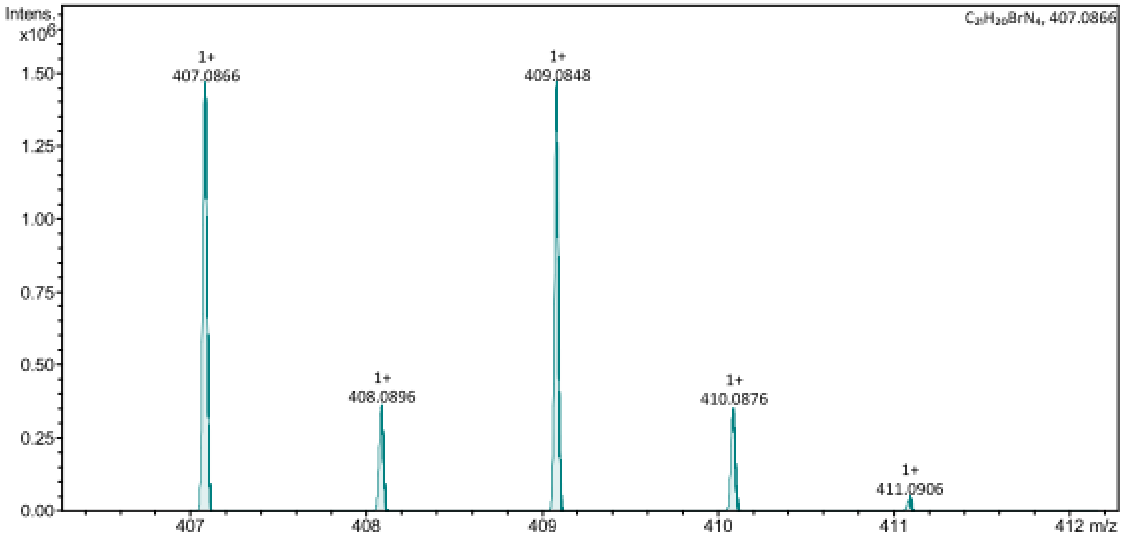

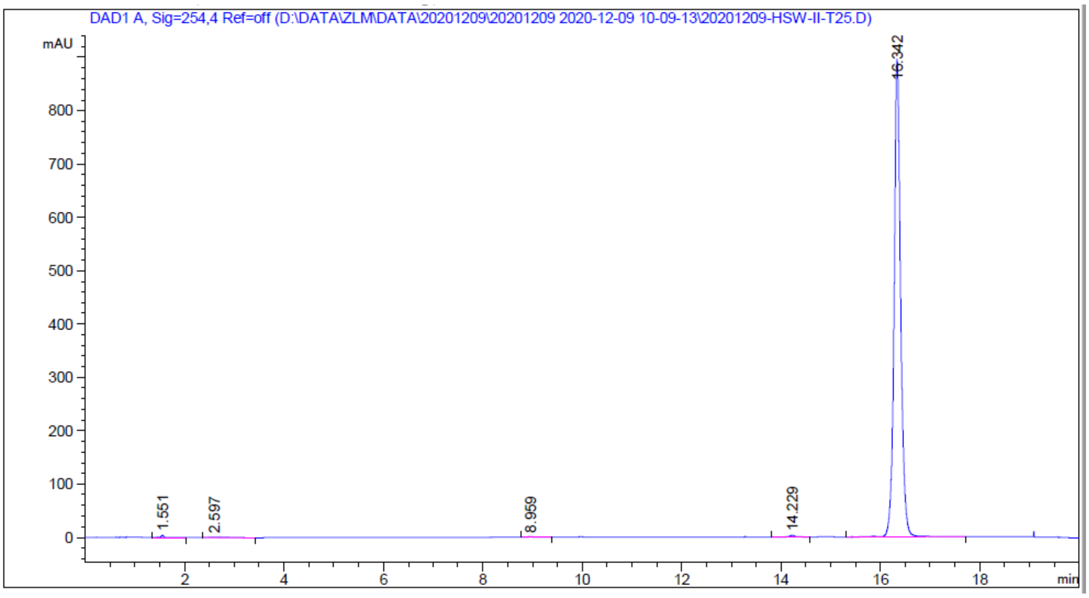

Signal 1: DAD1 A, Sig=254,4 Ref=off

Peak #	RetTime [min]	Type	Width [min]	Area [mAU*s]	Height [mAU]	Area %
1	1.551	BB	0.0871	18.73281	3.96411	0.2265
2	2.597	BB	0.3402	30.93503	1.23508	0.3740
3	8.959	BB	0.1457	10.67117	1.17815	0.1290
4	14.229	BB	0.1386	24.86176	2.94254	0.3005
5	16.342	BB	0.1557	8187.17871	886.78552	98.9701

1260R 12/9/2020 1:28:17 PM BY

Data File D:\DATA\ZLM\DATA\20201209\20201209 2020-12-09 10-09-13\20[illegible]
Sample Name: 20201209-HSW-II-T25

Totals : 8272.37948 896.10540

^{1}H NMR, ^{13}C NMR, HRMS, and HPLC of compound B22

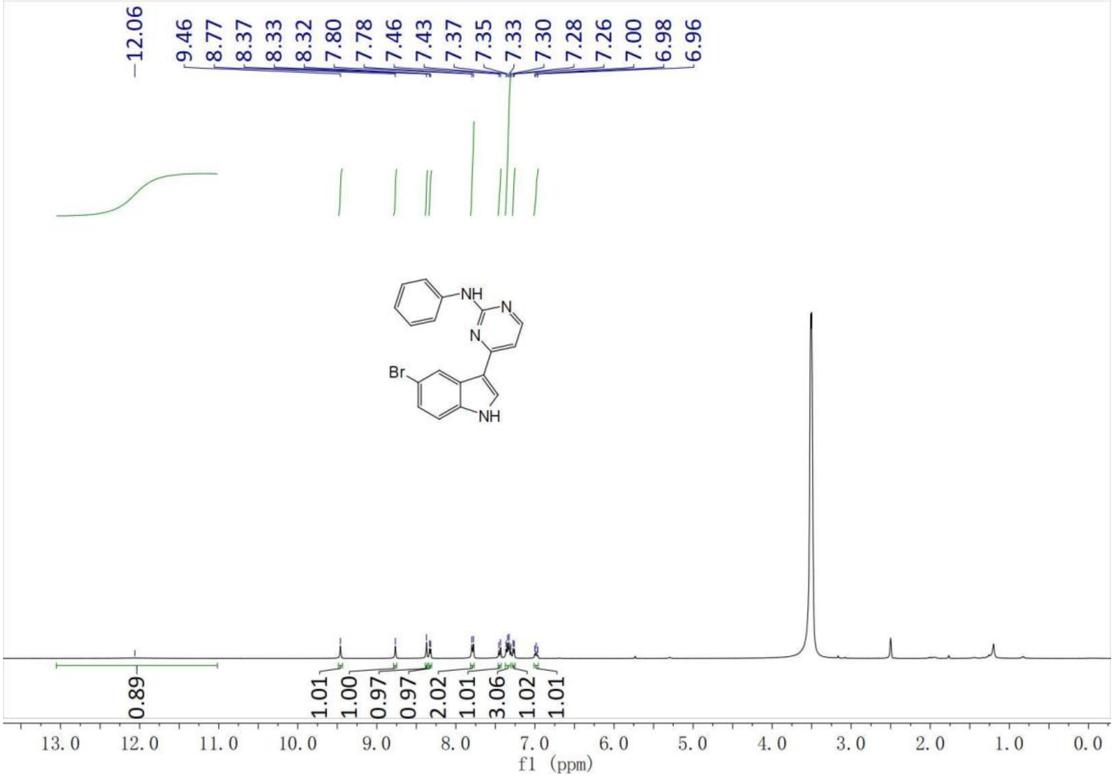

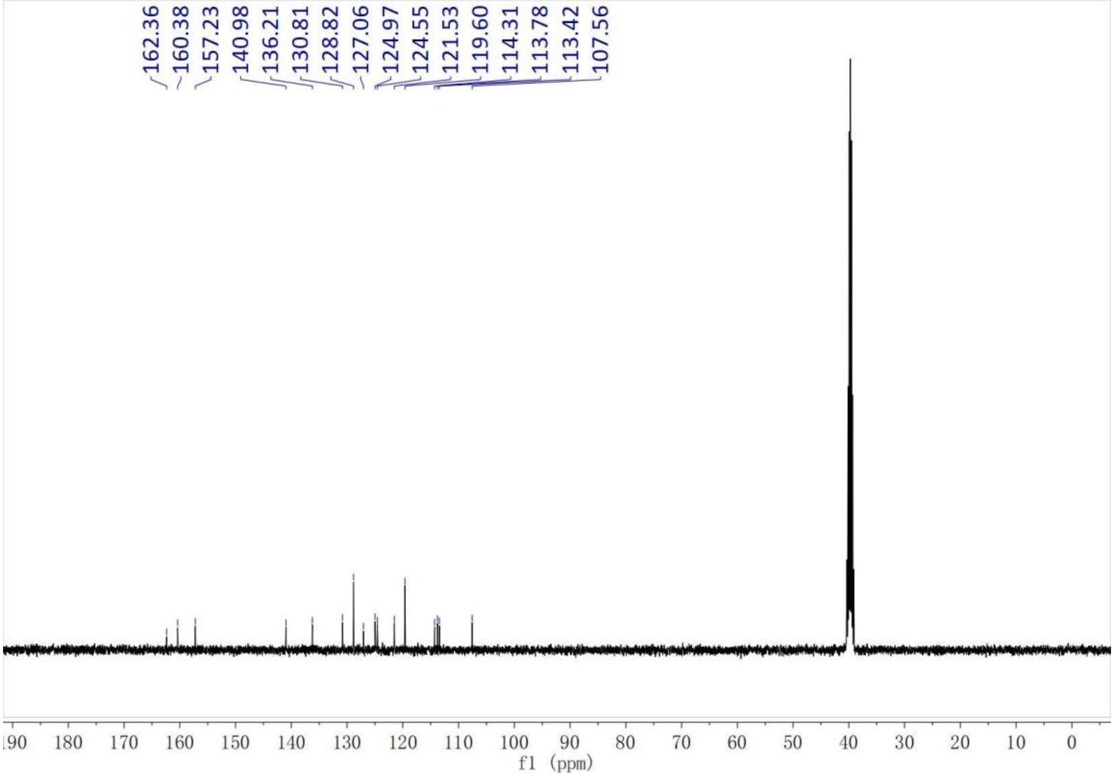

Display Report

Analysis Info

Acquisition Date 12/10/2020 16:28:41 PM

Sample Name TC1
Comment

Acquisition Parameter

Source Type	ESI	Ion Polarity	Positive	Set Nebulizer	2.0 Bar
Focus	Active	Set Capillary	4500 V	Set Dry Heater	200 °C
Scan Begin	50 m/z	Set End Plate Offset	-500 V	Set Dry Gas	8.0 l/min
Scan End	3000 m/z	Set Charging Voltage	2000 V	Set Divert Valve	Waste
		Set Corona	0 nA	Set APCI Heater	0 °C

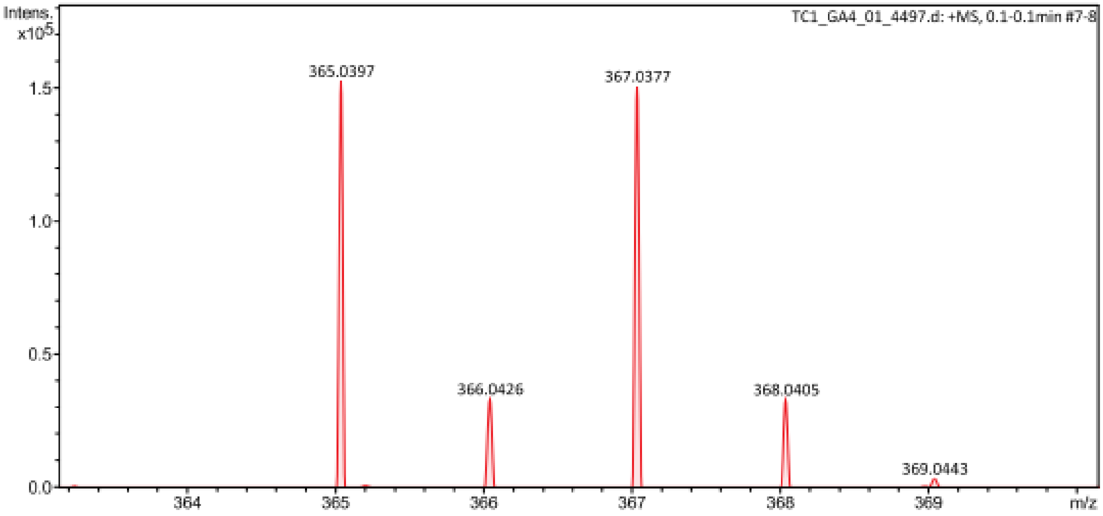

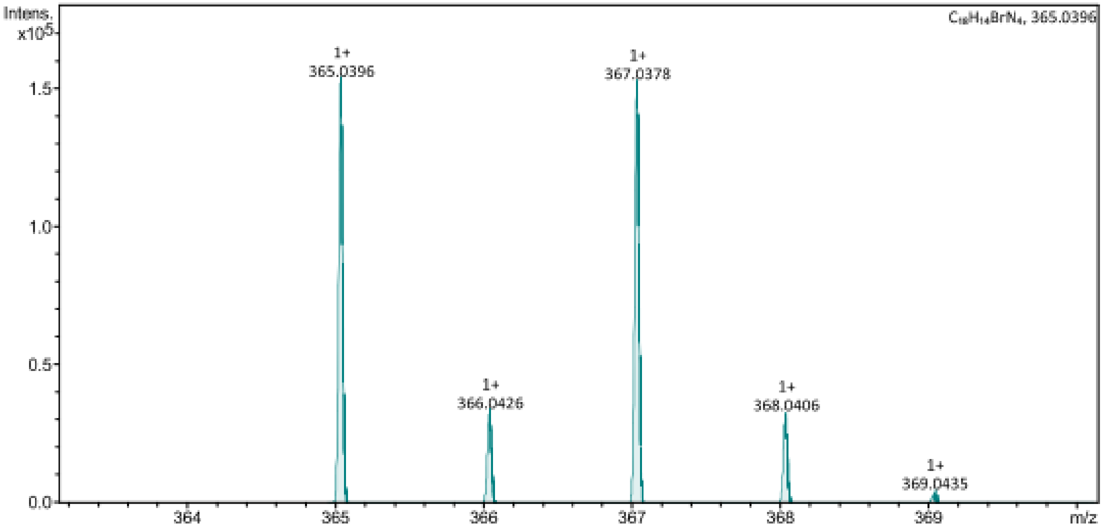

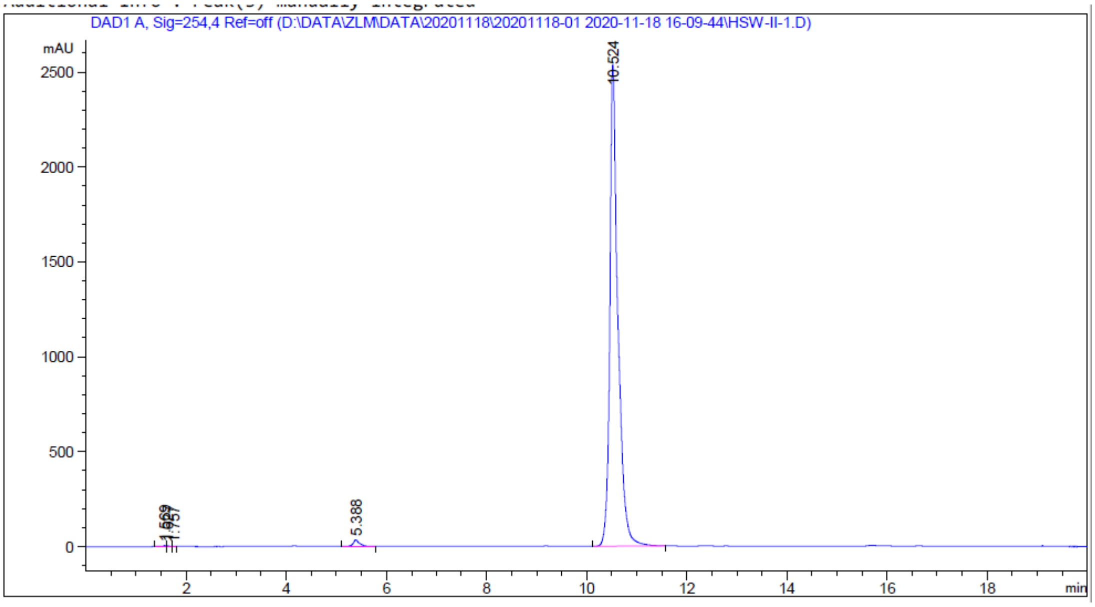

Signal 1: DAD1 A, Sig=254,4 Ref=off

Peak #	RetTime [min]	Type	Width [min]	Area [mAU*s]	Height [mAU]	Area %
1	1.569	BV	0.0333	13.84397	5.42704	0.0478
2	1.627	VV	0.0594	13.15711	2.77062	0.0454
3	1.757	VB	0.0471	6.62905	1.79471	0.0229
4	5.388	BB	0.1217	310.11169	35.73909	1.0709

1260R 12/7/2020 3:18:16 PM BY

Data File D:\DATA\ZLM\DATA\20201118\20201118-01 2020-11-18 16-09-44\
Sample Name: HSW-II-1

Peak #	RetTime [min]	Type	Width [min]	Area [mAU*s]	Height [mAU]	Area %
5	10.524	BB	0.1589	2.86152e4	2540.38354	98.8130

Totals : 2.89589e4 2586.11500

^{1}H NMR, ^{13}C NMR, HRMS, and HPLC of compound B23

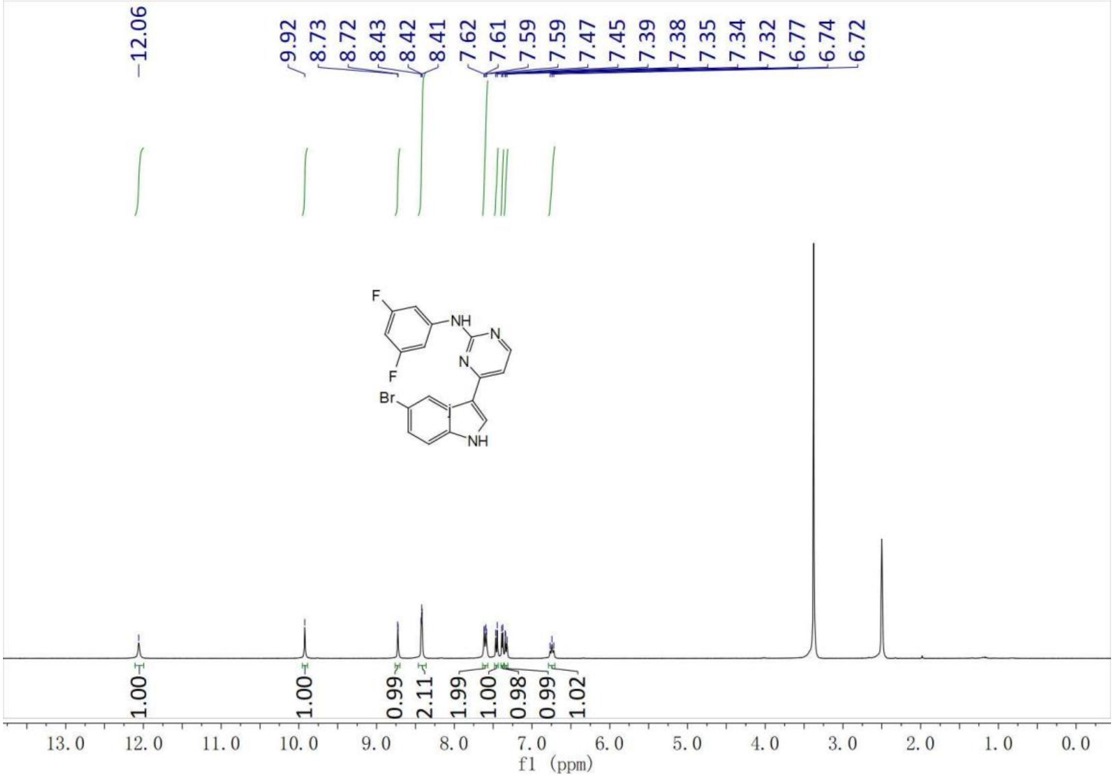

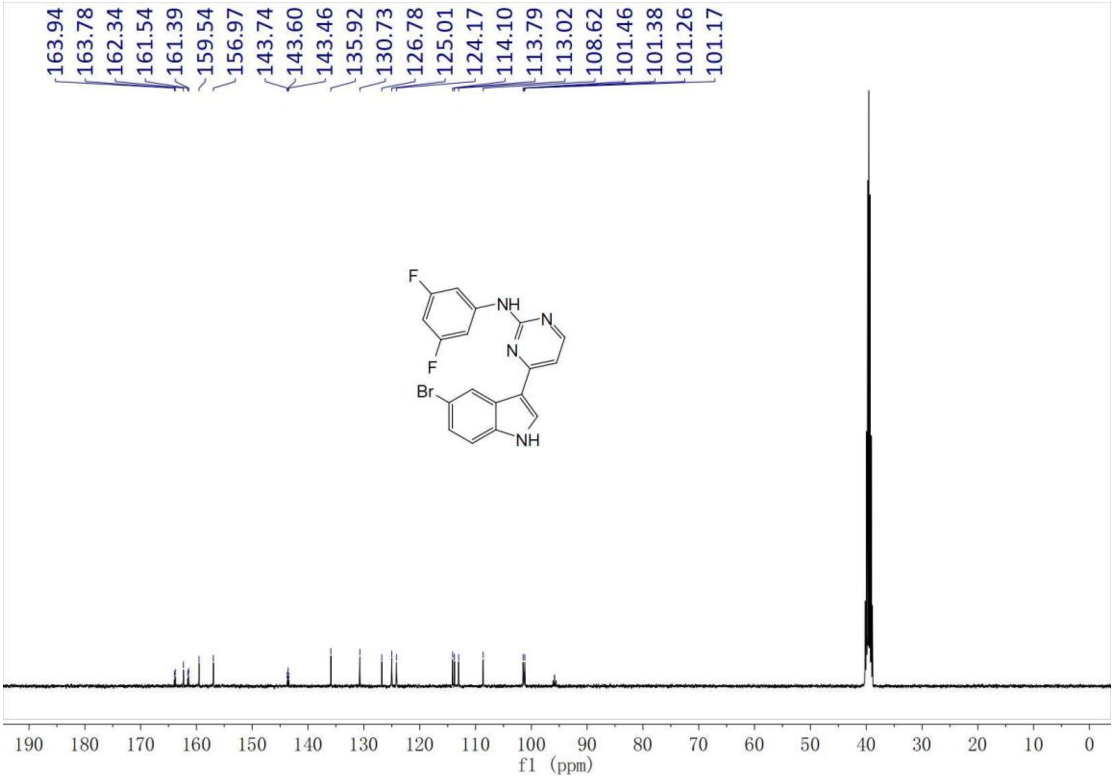

Display Report

Analysis Info

Acquisition Date 12/10/2020 16:53:34 PM

Sample Name TC10
Comment

Acquisition Parameter

Source Type	ESI	Ion Polarity	Positive	Set Nebulizer	2.0 Bar
Focus	Active	Set Capillary	4500 V	Set Dry Heater	200 °C
Scan Begin	50 m/z	Set End Plate Offset	-500 V	Set Dry Gas	8.0 l/min
Scan End	3000 m/z	Set Charging Voltage	2000 V	Set Divert Valve	Waste
		Set Corona	0 nA	Set APCI Heater	0 °C

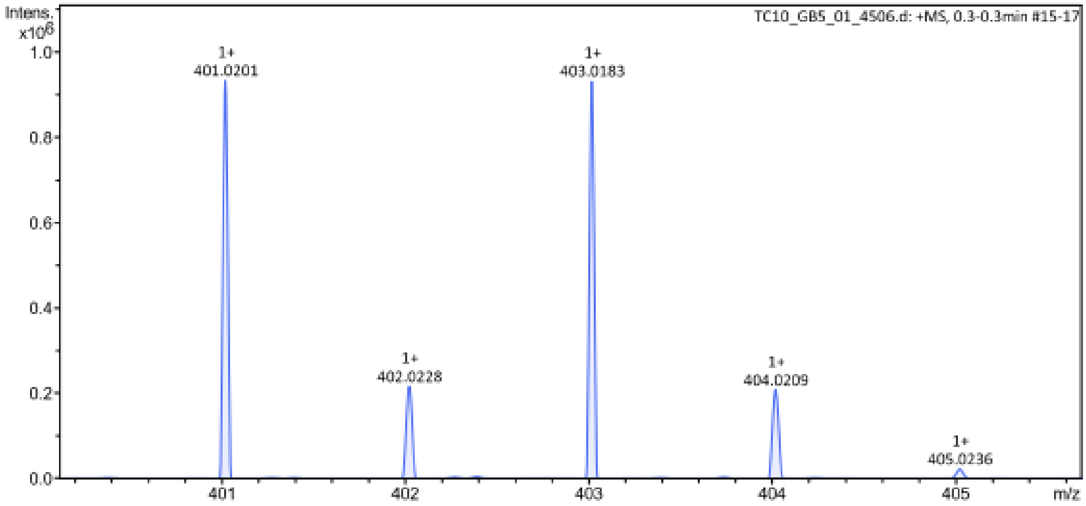

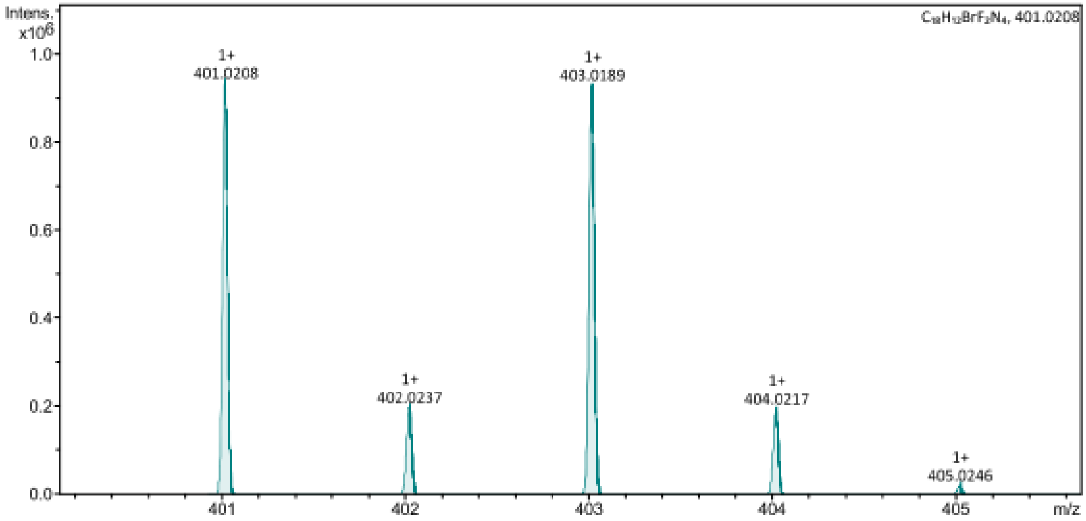

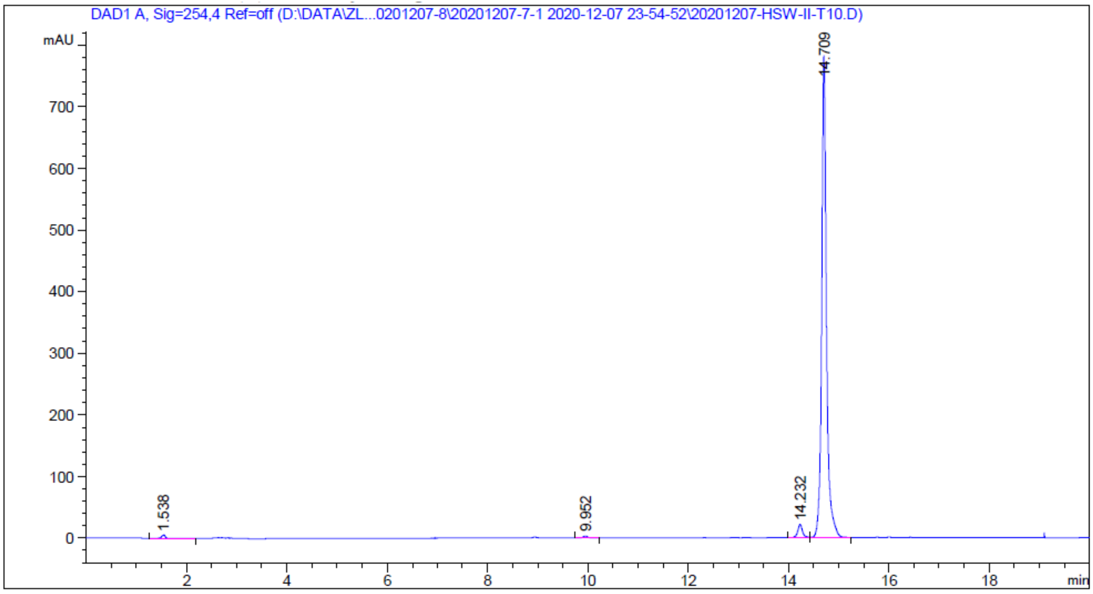

Signal 1: DAD1 A, Sig=254,4 Ref=off

Peak #	RetTime [min]	Type	Width [min]	Area [mAU*s]	Height [mAU]	Area %
1	1.538	BB	0.1087	30.40622	5.16693	0.5646
2	9.952	BB	0.1151	20.20178	3.13976	0.3751
3	14.232	BV	0.1195	143.35297	21.03214	2.6619
4	14.709	VB	0.1192	5191.47168	764.34351	96.3984

1260R 12/8/2020 2:26:30 PM BY

Data File D:\DATA\ZL...A\20201207-8\20201207-7-1 2020-12-07 23-54-52\202
Sample Name: 20201207-HSW-II-T10

Peak #	RetTime [min]	Type	Width [min]	Area [mAU*s]	Height [mAU]	Area %
Totals :				5385.43265	793.68234	

^{1}H NMR, ^{13}C NMR, HRMS, and HPLC of compound B24

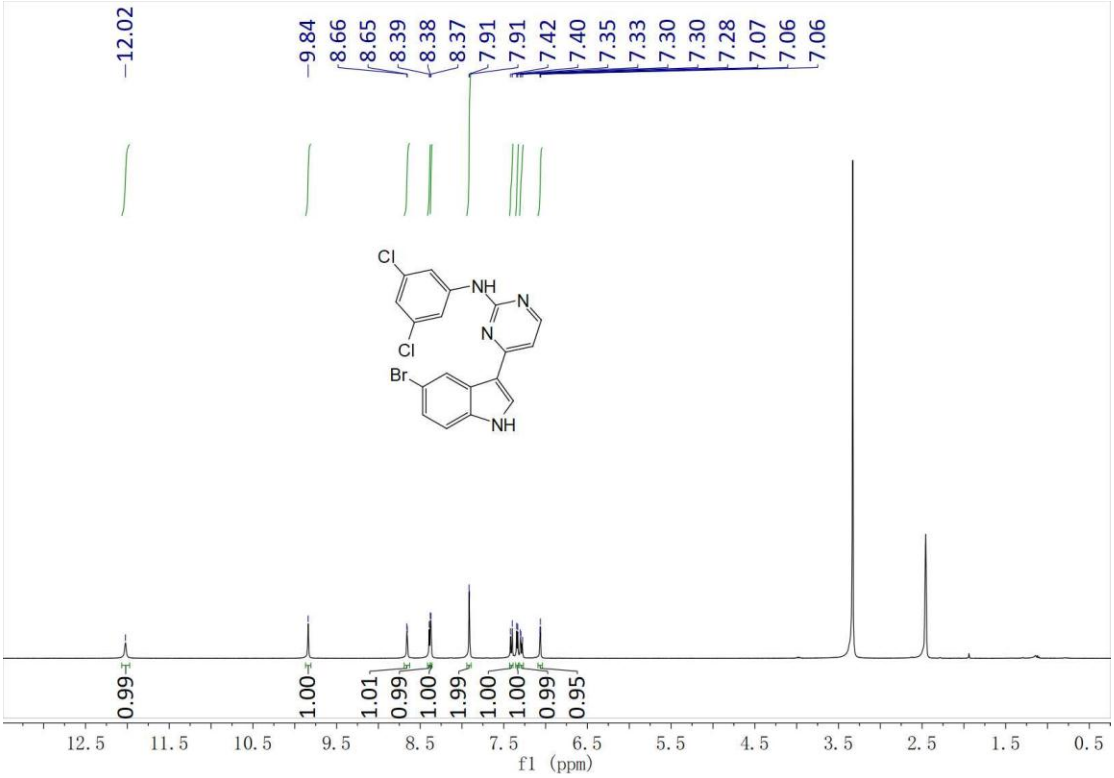

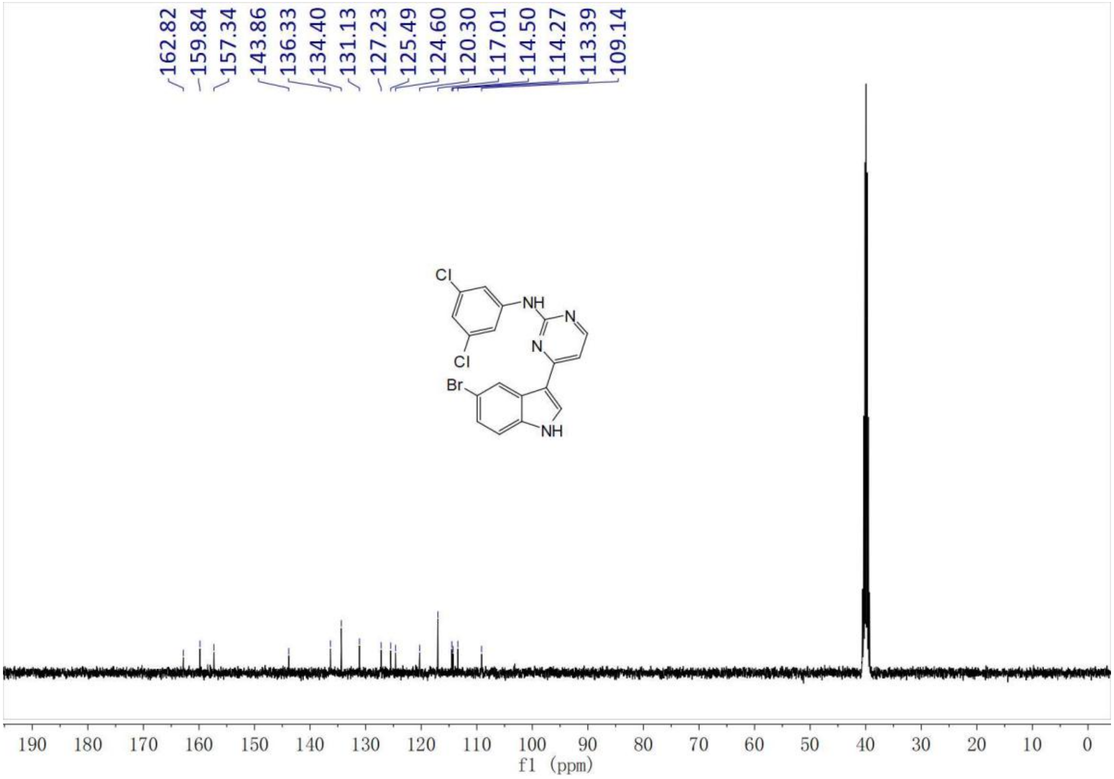

Display Report

Analysis Info

Acquisition Date 12/10/2020 16:56:20 PM

Sample Name TC11
Comment

Acquisition Parameter

Source Type	ESI	Ion Polarity	Positive	Set Nebulizer	2.0 Bar
Focus	Active	Set Capillary	4500 V	Set Dry Heater	200 °C
Scan Begin	50 m/z	Set End Plate Offset	-500 V	Set Dry Gas	8.0 l/min
Scan End	3000 m/z	Set Charging Voltage	2000 V	Set Divert Valve	Waste
		Set Corona	0 nA	Set APCI Heater	0 °C

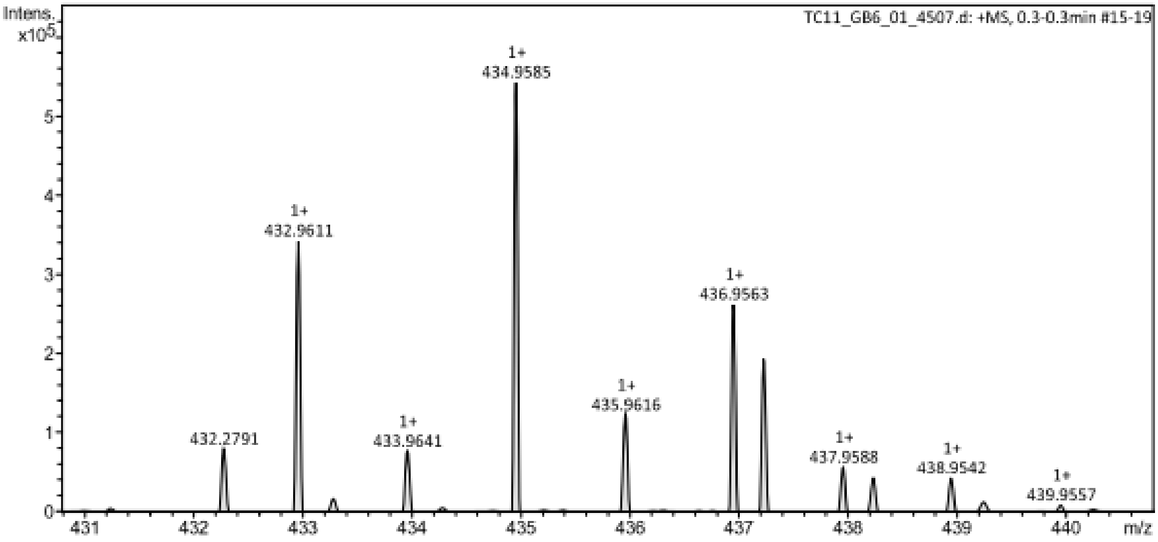

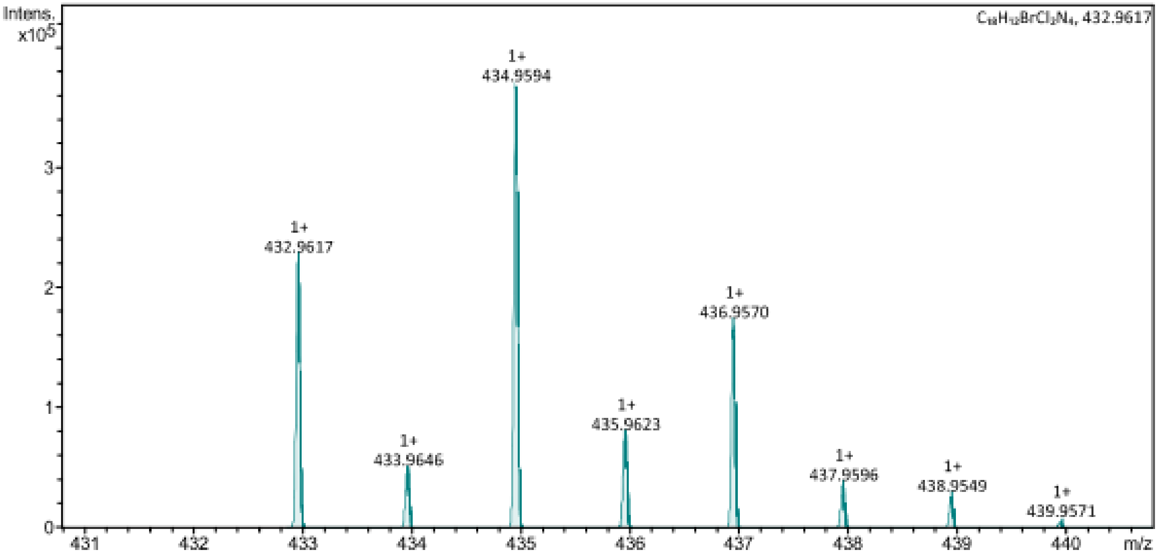

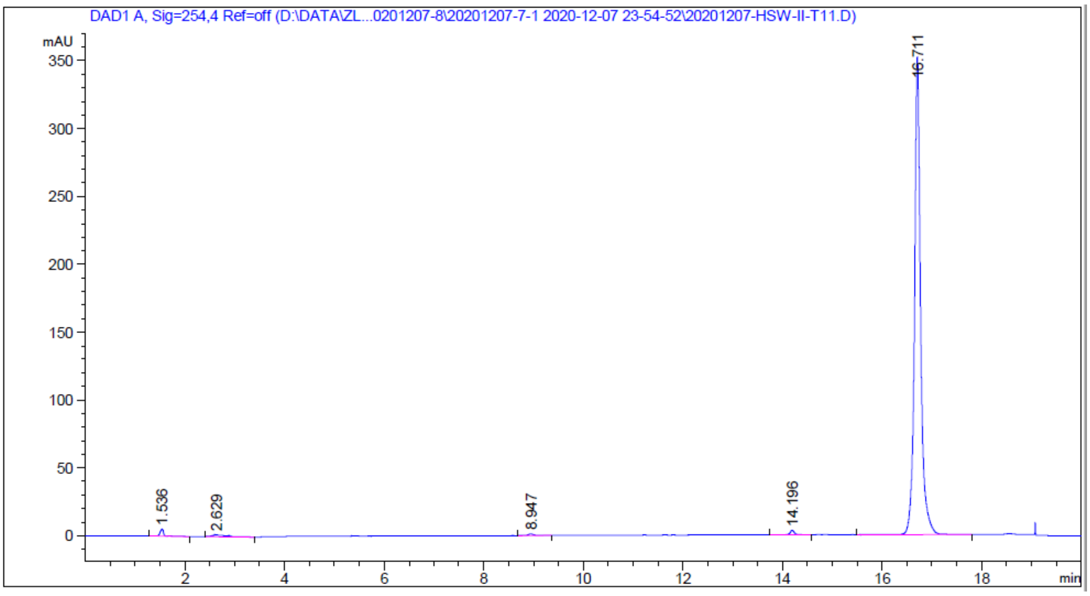

Signal 1: DAD1 A, Sig=254,4 Ref=off

Peak #	RetTime [min]	Type	Width [min]	Area [mAU*s]	Height [mAU]	Area %
1	1.536	BB	0.0838	26.62742	5.19514	0.8564
2	2.629	BB	0.2807	31.14146	1.43799	1.0015
3	8.947	BB	0.1251	10.41939	1.20183	0.3351
4	14.196	BB	0.0997	22.47720	3.46493	0.7229

1260R 12/8/2020 9:31:27 PM BY

Data File D:\DATA\ZL...A\20201207-8\20201207-7-1 2020-12-07 23-54-52\2(
Sample Name: 20201207-HSW-II-T11

Peak #	RetTime [min]	Type	Width [min]	Area [mAU*s]	Height [mAU]	Area %
5	16.711	BB	0.1286	3018.73804	349.95999	97.0842

Totals : 3109.40352 361.25987

^{1}H NMR, ^{13}C NMR, HRMS, and HPLC of compound B25

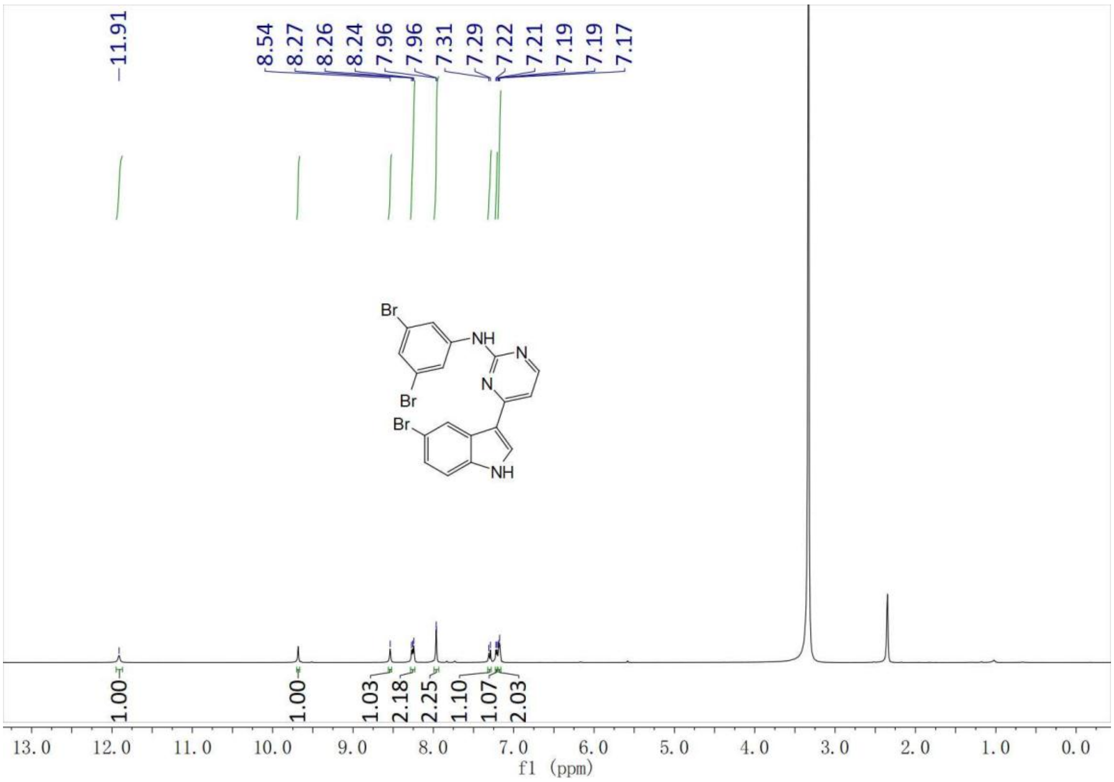

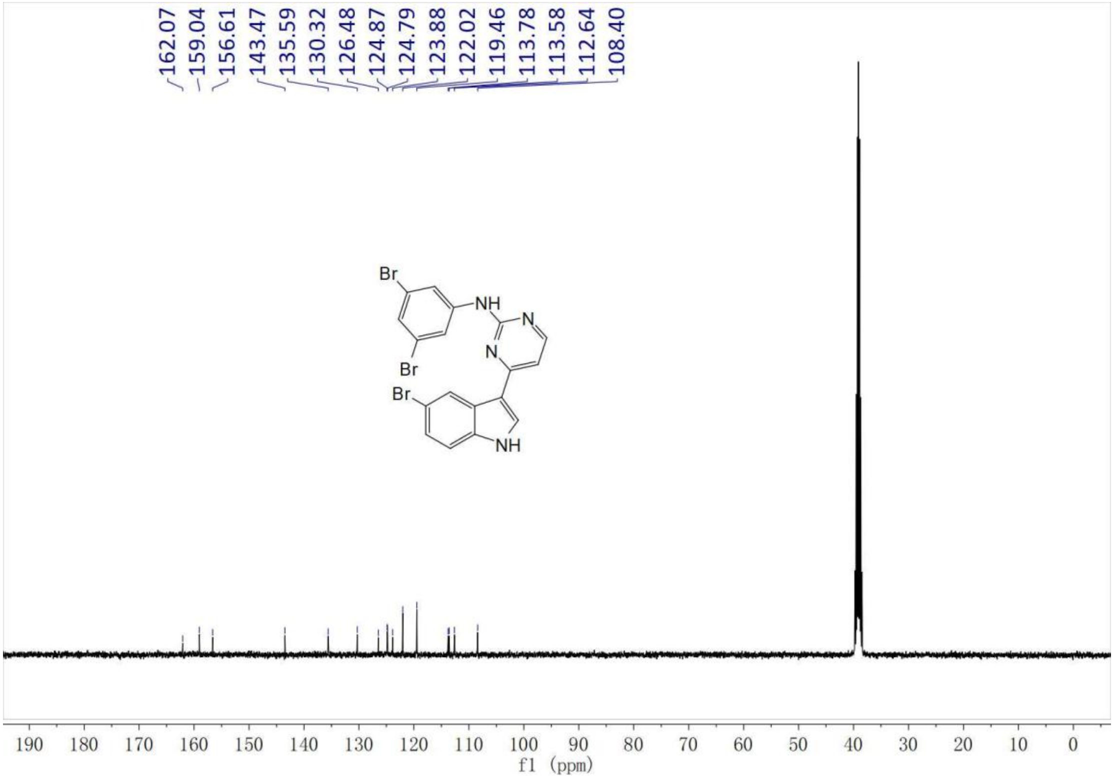

Display Report

Analysis Info

Acquisition Date 12/10/2020 17:36:11 PM

Sample Name TC27
Comment

Acquisition Parameter

Source Type	ESI	Ion Polarity	Positive	Set Nebulizer	2.0 Bar
Focus	Active	Set Capillary	4500 V	Set Dry Heater	200 °C
Scan Begin	50 m/z	Set End Plate Offset	-500 V	Set Dry Gas	8.0 l/min
Scan End	3000 m/z	Set Charging Voltage	2000 V	Set Divert Valve	Waste
		Set Corona	0 nA	Set APCI Heater	0 °C

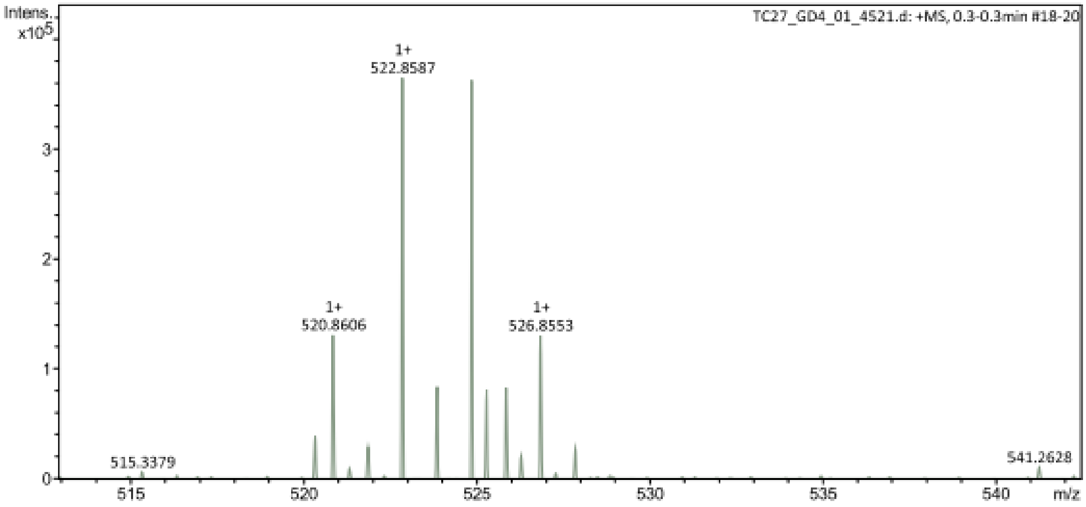

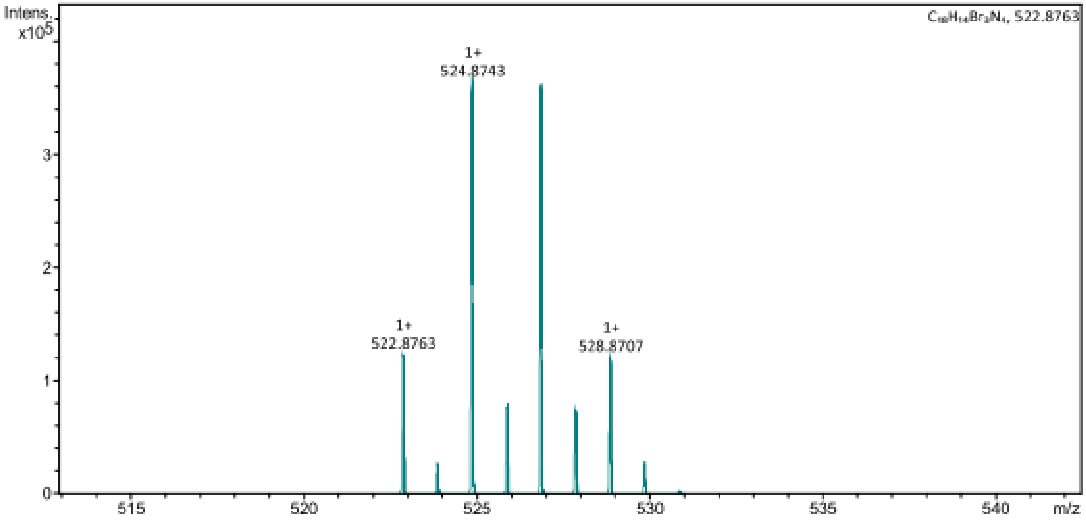

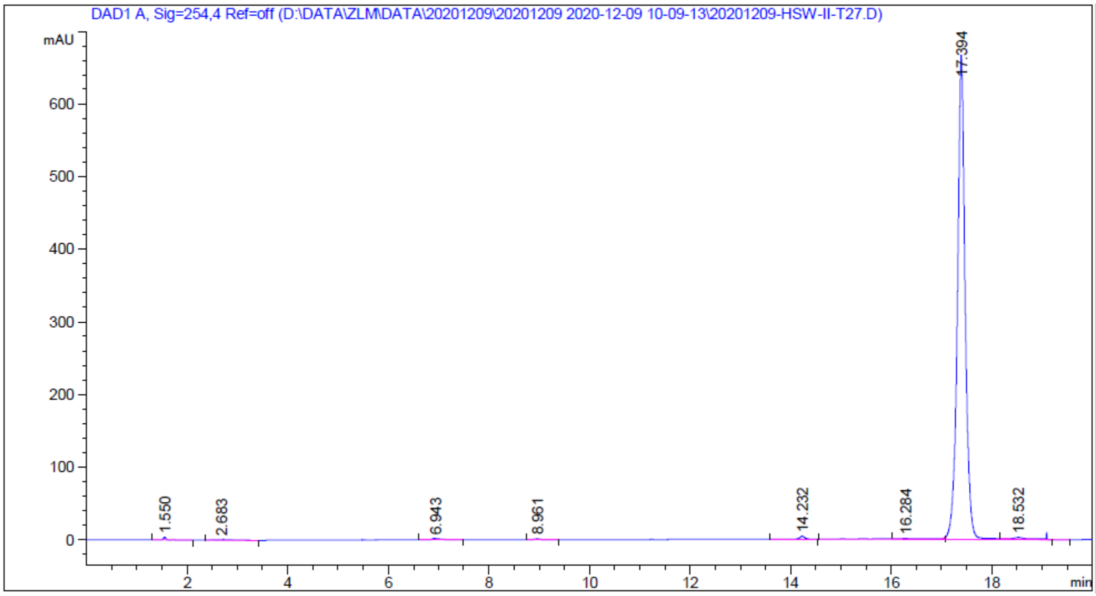

Signal 1: DAD1 A, Sig=254,4 Ref=off

Peak #	RetTime [min]	Type	Width [min]	Area [mAU*s]	Height [mAU]	Area %
1	1.550	BB	0.0858	16.23676	3.52382	0.2181
2	2.683	BB	0.3925	30.89738	1.02266	0.4151
3	6.943	BB	0.2088	22.69437	1.64669	0.3049
4	8.961	BB	0.1485	10.04453	1.07995	0.1349
5	14.232	BB	0.1290	30.91909	4.05429	0.4154
6	16.284	VV E	0.5266	49.97078	1.23886	0.6714

1260R 12/9/2020 1:32:06 PM BY

Data File D:\DATA\ZLM\DATA\20201209\20201209 2020-12-09 10-09-13\202C
Sample Name: 20201209-HSW-II-T27

Peak #	RetTime [min]	Type	Width [min]	Area [mAU*s]	Height [mAU]	Area %
7	17.394	VV R	0.1760	7223.75146	658.37195	97.0508
8	18.532	VBAE	0.3507	58.75708	2.26488	0.7894

Totals :	7443.27146	673.20311

^{1}H NMR, ^{13}C NMR, HRMS, and HPLC of compound B26

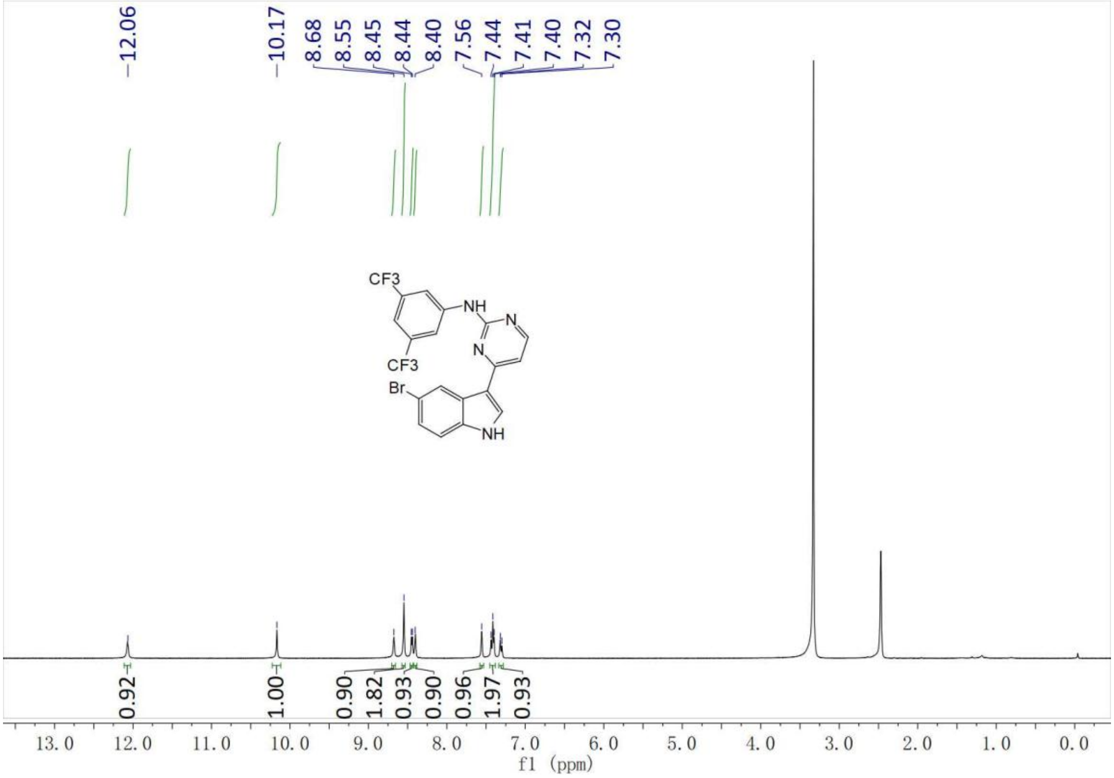

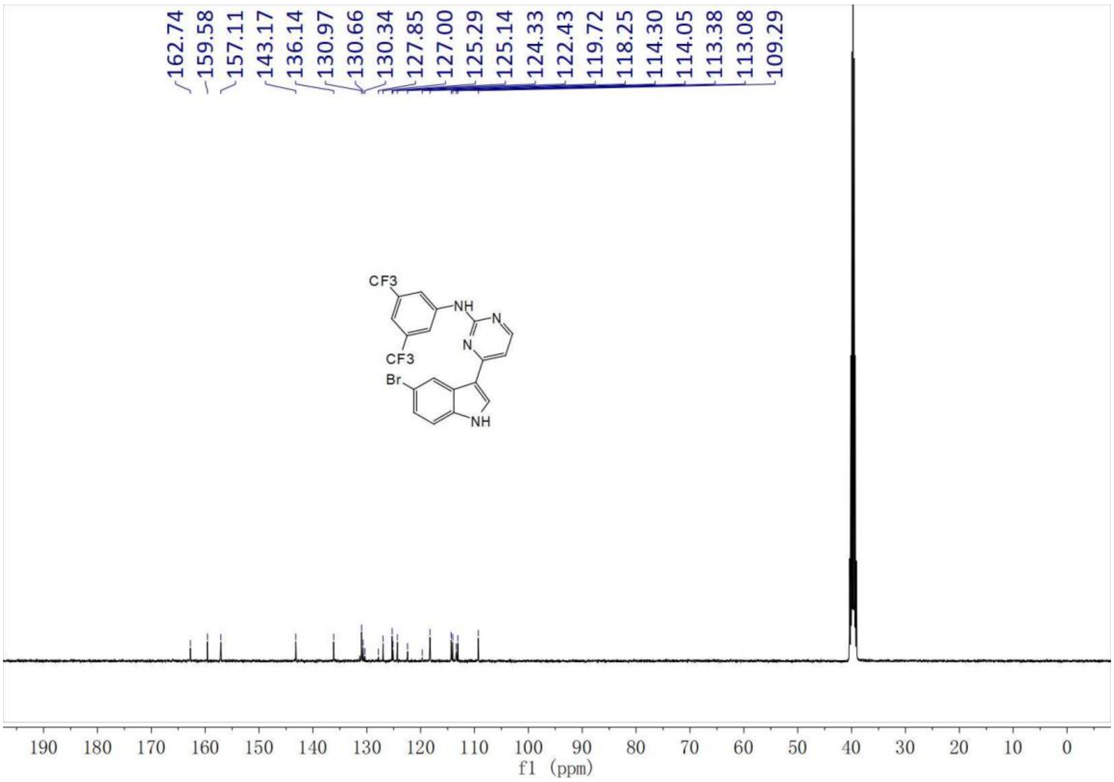

Display Report

Analysis Info Acquisition Date 12/10/2020 17:38:57 PM

Sample Name TC28
Comment

Acquisition Parameter

Source Type	ESI	Ion Polarity	Positive	Set Nebulizer	2.0 Bar
Focus	Active	Set Capillary	4500 V	Set Dry Heater	200 °C
Scan Begin	50 m/z	Set End Plate Offset	-500 V	Set Dry Gas	8.0 l/min
Scan End	3000 m/z	Set Charging Voltage	2000 V	Set Divert Valve	Waste
		Set Corona	0 nA	Set APCI Heater	0 °C

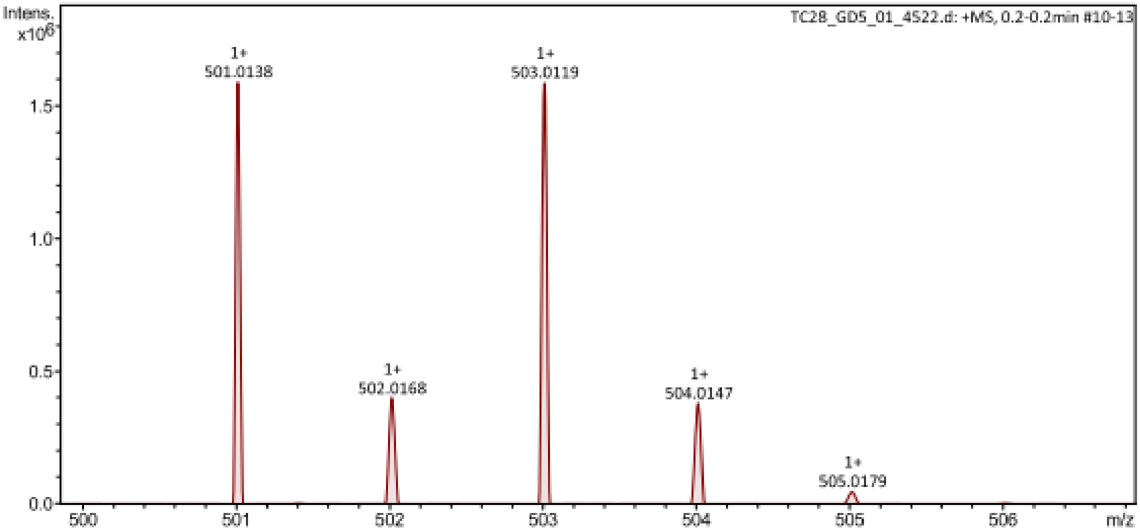

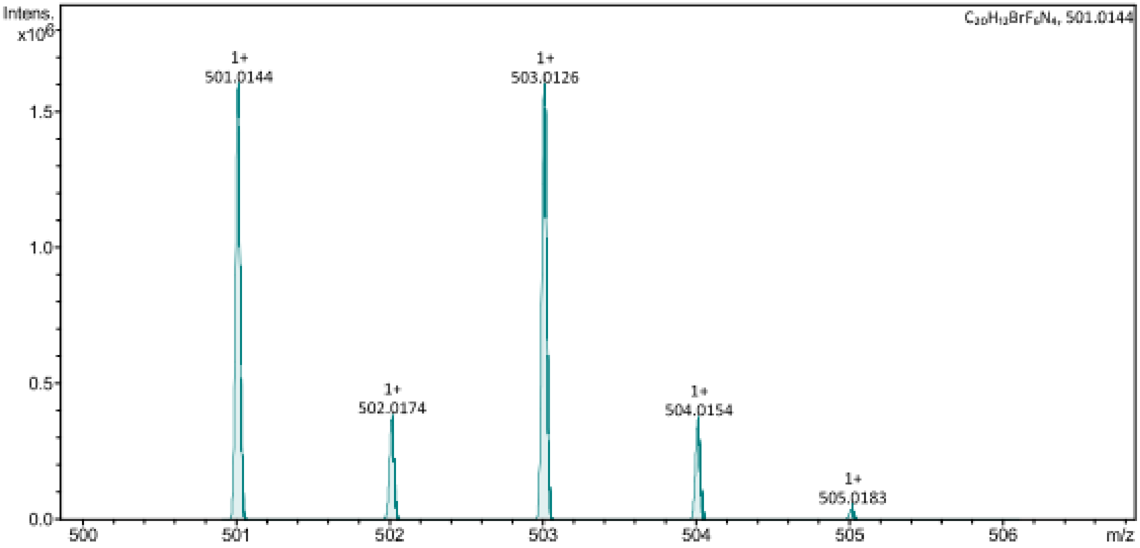

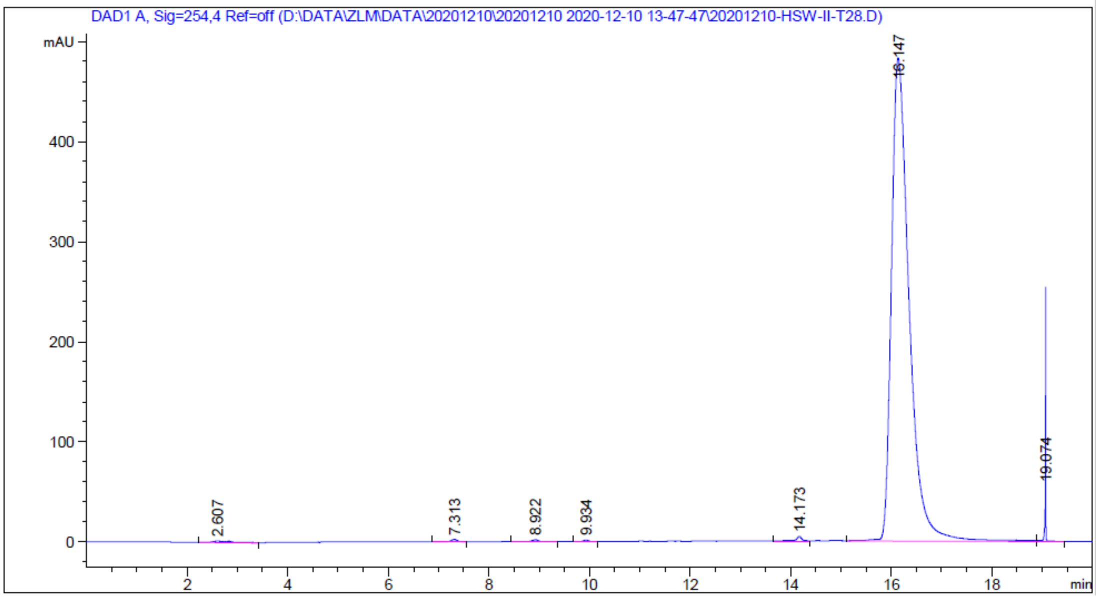

Signal 1: DAD1 A, Sig=254,4 Ref=off

Peak #	RetTime [min]	Type	Width [min]	Area [mAU*s]	Height [mAU]	Area %
1	2.607	BB	0.3416	35.45646	1.40903	0.2941
2	7.313	BB	0.1277	19.44821	2.58632	0.1613
3	8.922	BB	0.1514	18.27141	1.91431	0.1516
4	9.934	BB	0.1141	10.12263	1.59361	0.0840

1260R 12/23/2020 10:51:13 AM BY

Data File D:\DATA\ZLM\DATA\20201210\20201210 2020-12-10 13-47-47\20201[illegible]
Sample Name: 20201210-HSW-II-T28

Peak #	RetTime [min]	Type	Width [min]	Area [mAU*s]	Height [mAU]	Area %
5	14.173	BB	0.1220	30.77377	4.37929	0.2553
6	16.147	BV	0.3739	1.18017e4	481.58182	97.8973
7	19.074	VBA	0.0616	139.40800	54.94632	1.1564

Totals : 1.20552e4 548.41071

^{1}H NMR, ^{13}C NMR, HRMS, and HPLC of compound B27

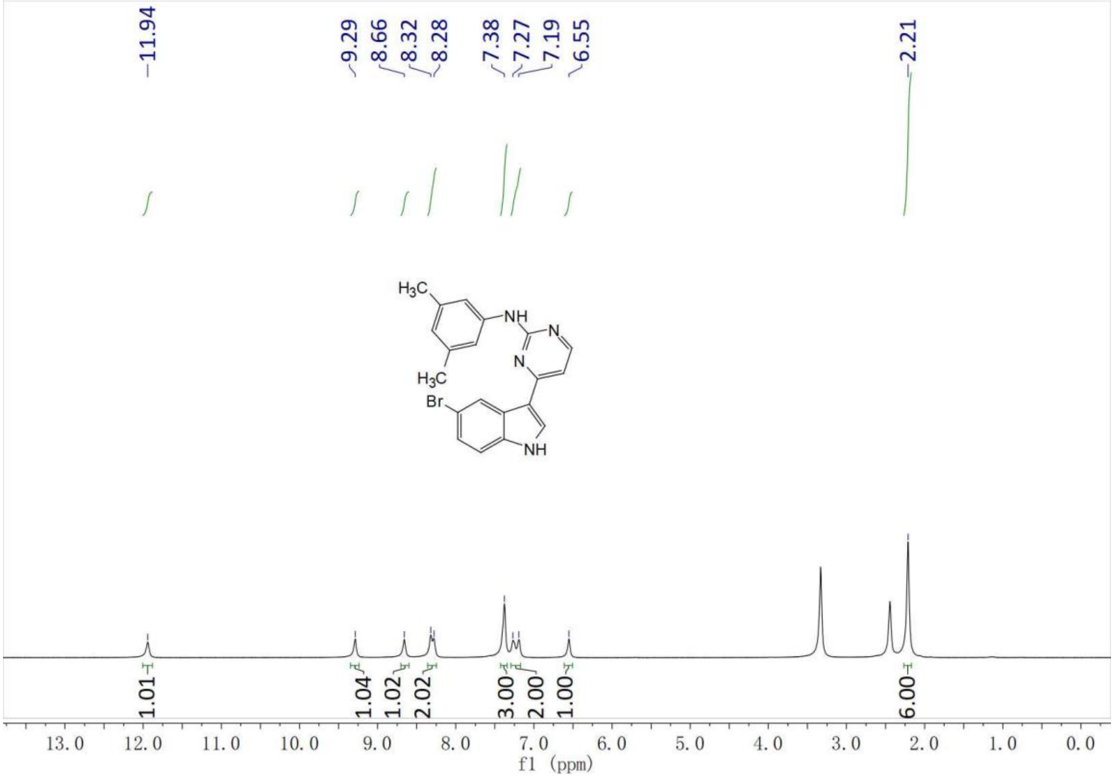

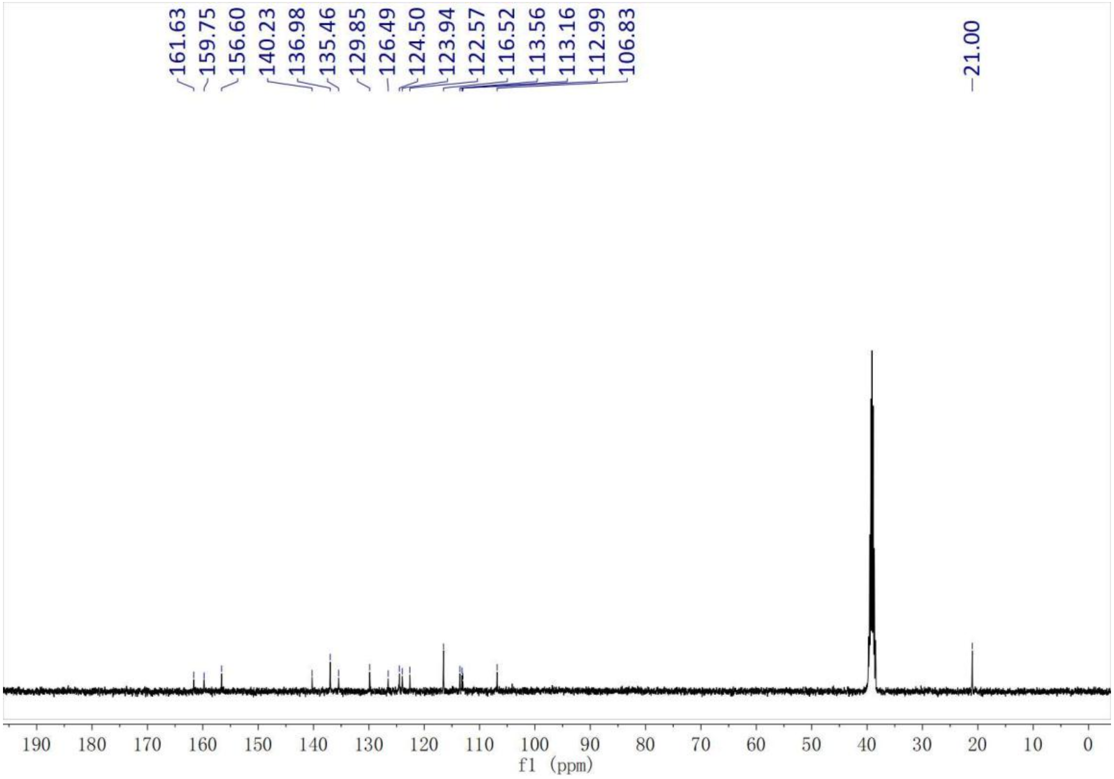

Display Report

Analysis Info

Acquisition Date 12/10/2020 16:50:48 PM

Sample Name TC9
Comment

Acquisition Parameter

Source Type	ESI	Ion Polarity	Positive	Set Nebulizer	2.0 Bar
Focus	Active	Set Capillary	4500 V	Set Dry Heater	200 °C
Scan Begin	50 m/z	Set End Plate Offset	-500 V	Set Dry Gas	8.0 l/min
Scan End	3000 m/z	Set Charging Voltage	2000 V	Set Divert Valve	Waste
		Set Corona	0 nA	Set APCI Heater	0 °C

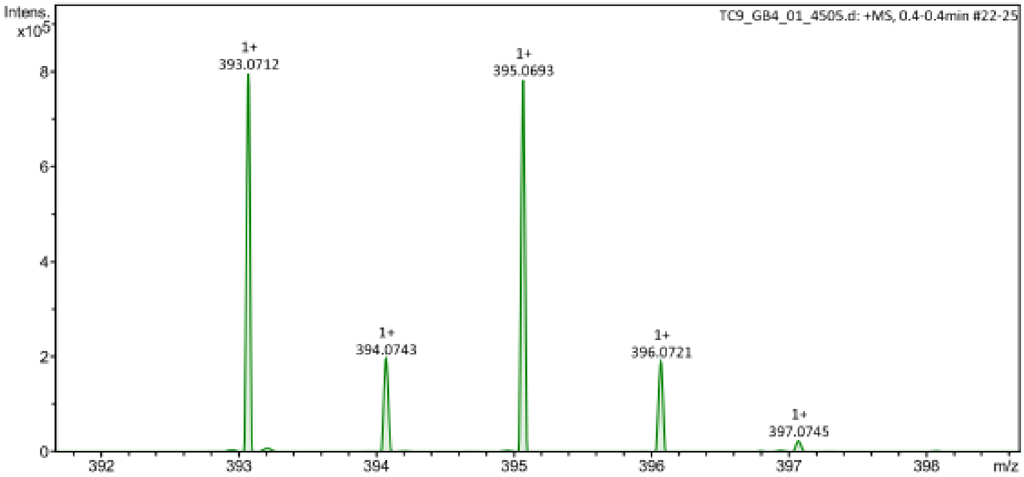

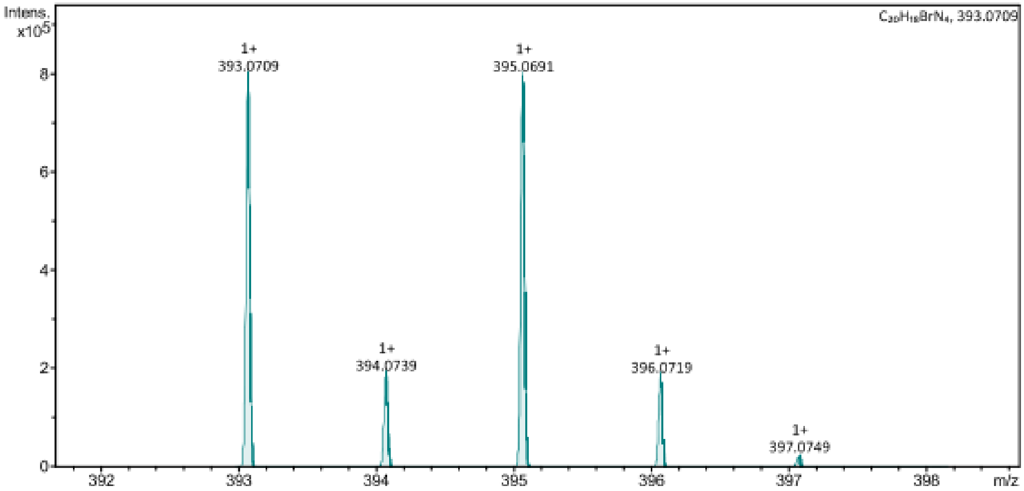

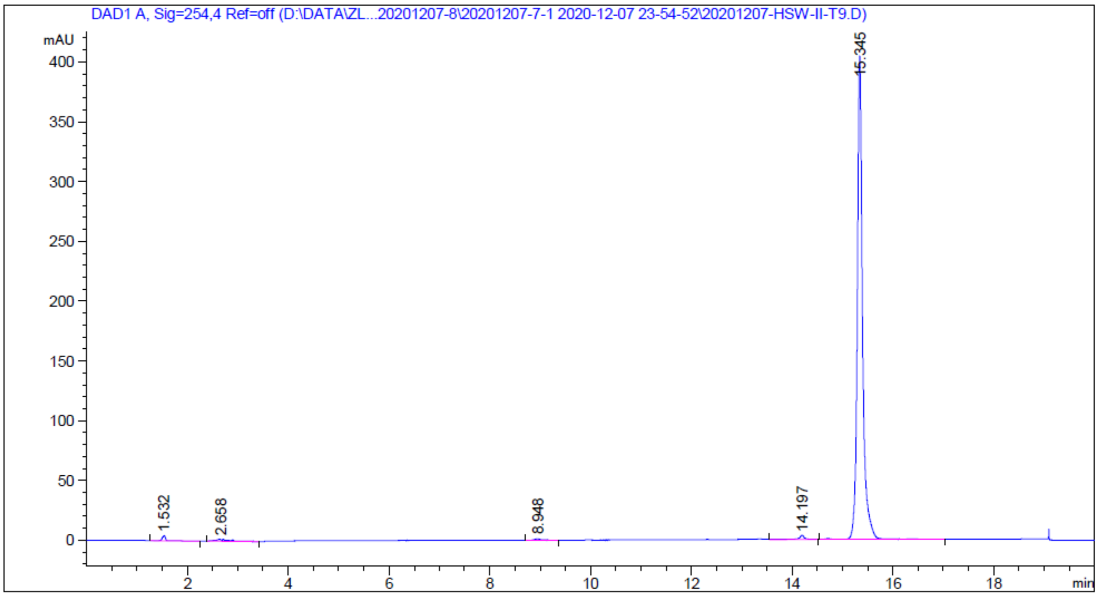

Signal 1: DAD1 A, Sig=254,4 Ref=off

Peak #	RetTime [min]	Type	Width [min]	Area [mAU*s]	Height [mAU]	Area %
1	1.532	BB	0.1006	20.80091	4.00428	0.6840
2	2.658	BB	0.3165	33.23027	1.48812	1.0926
3	8.948	BB	0.1390	9.31774	1.09857	0.3064
4	14.197	VB	0.1308	26.24071	3.36981	0.8628

1260R 12/8/2020 2:25:05 PM BY

Data File D:\DATA\ZL...TA\20201207-8\20201207-7-1 2020-12-07 23-54-52\202
Sample Name: 20201207-HSW-II-T9

Peak #	RetTime [min]	Type	Width [min]	Area [mAU*s]	Height [mAU]	Area %
5	15.345	VV R	0.1278	2951.68823	392.34314	97.0542
Totals :				3041.27786	402.30393	

^{1}H NMR, ^{13}C NMR, HRMS, and HPLC of compound B28

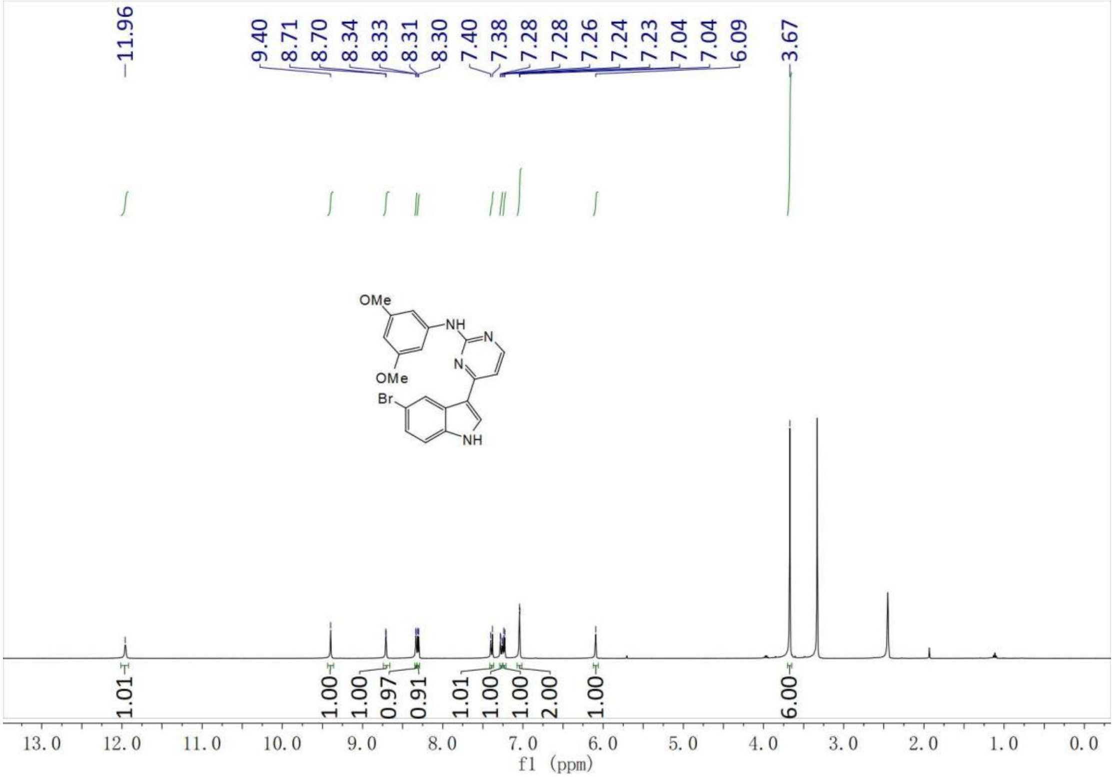

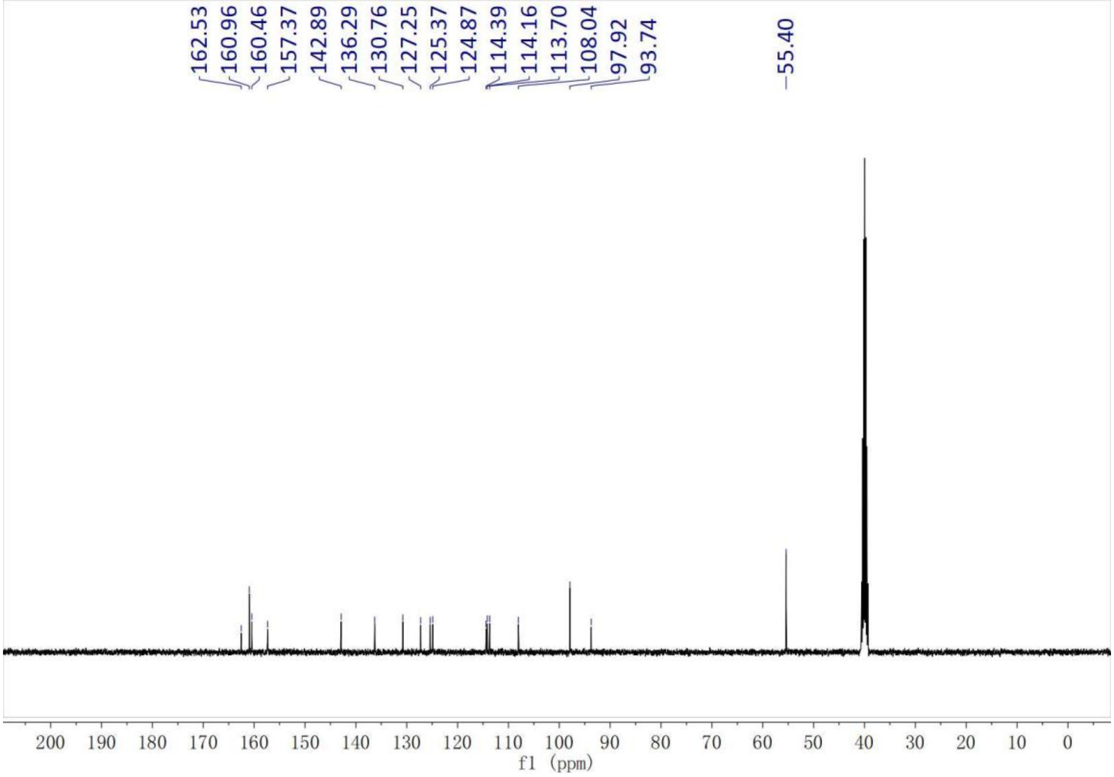

Display Report

Analysis Info Acquisition Date 12/10/2020 16:48:02 PM

Sample Name TC8
Comment

Acquisition Parameter

Source Type	ESI	Ion Polarity	Positive	Set Nebulizer	2.0 Bar
Focus	Active	Set Capillary	4500 V	Set Dry Heater	200 °C
Scan Begin	50 m/z	Set End Plate Offset	-500 V	Set Dry Gas	8.0 l/min
Scan End	3000 m/z	Set Charging Voltage	2000 V	Set Divert Valve	Waste
		Set Corona	0 nA	Set APCI Heater	0 °C

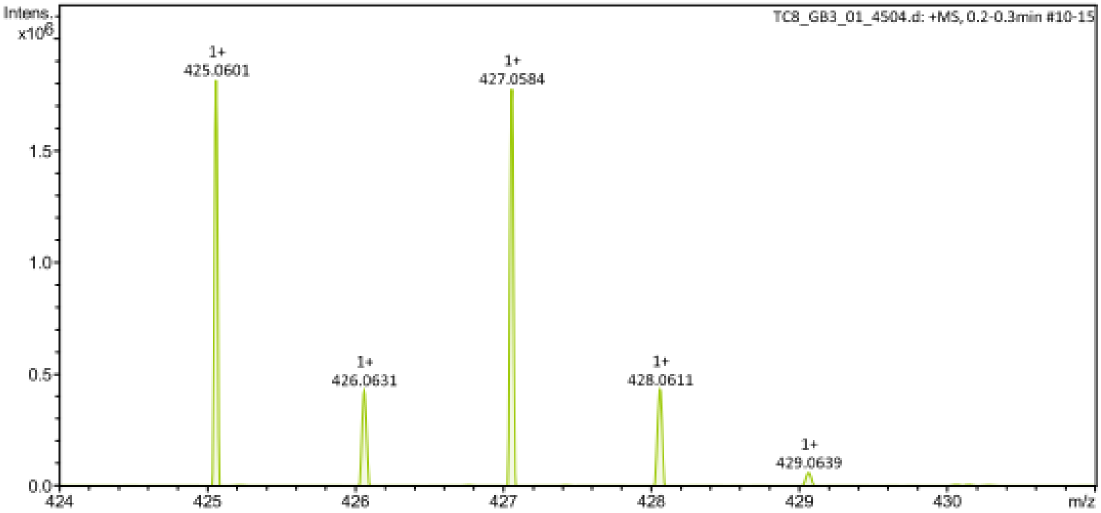

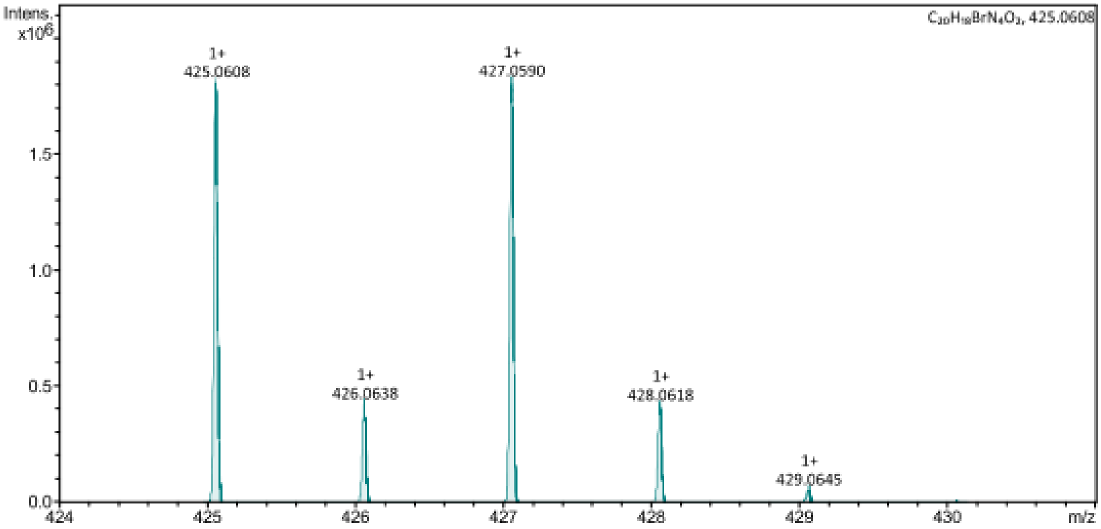

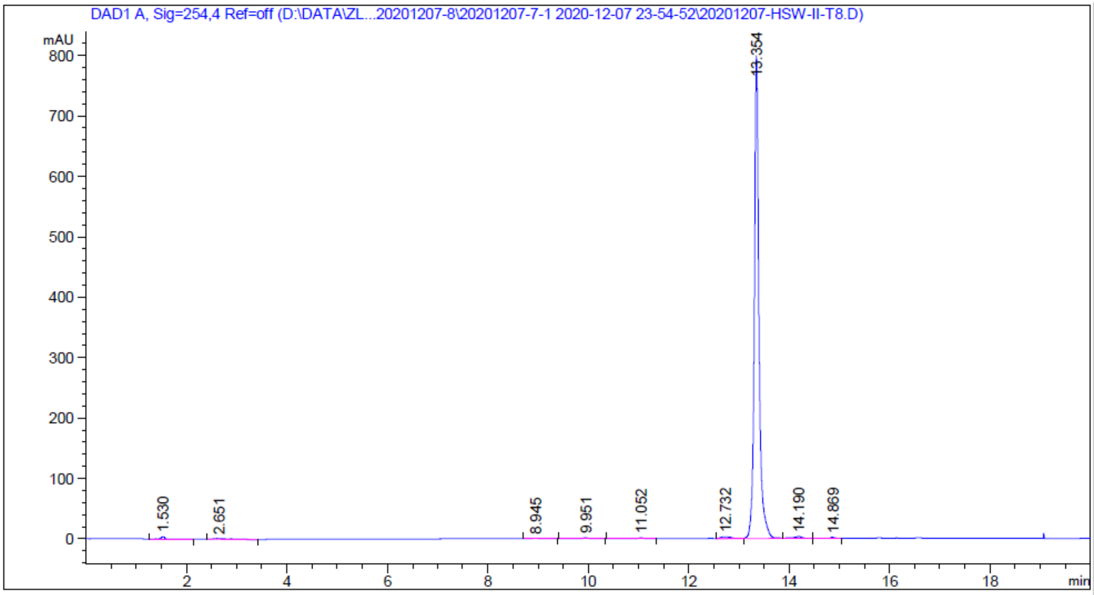

Peak #	RetTime [min]	Type	Width [min]	Area [mAU*s]	Height [mAU]	Area %
1	1.530	BB	0.0941	21.06940	3.95616	0.3931
2	2.651	BB	0.3404	31.74578	1.33981	0.5923
3	8.945	BB	0.1414	11.01046	1.26665	0.2054
4	9.951	BB	0.1272	13.07145	1.74893	0.2439

1260R 12/8/2020 2:23:24 PM BY

Data File D:\DATA\ZL...TA\20201207-8\20201207-7-1 2020-12-07 23-54-52\2
Sample Name: 20201207-HSW-II-T8

Peak #	RetTime [min]	Type	Width [min]	Area [mAU*s]	Height [mAU]	Area %
5	11.052	BB	0.1340	13.03622	1.61823	0.2432
6	12.732	BV E	0.2120	40.65423	3.04037	0.7585
7	13.354	VV R	0.1177	5186.68506	779.06372	96.7693
8	14.190	VB E	0.1529	32.25004	3.33188	0.6017
9	14.869	BB	0.1286	10.32345	1.35966	0.1926

Totals : 5359.84609 796.72540

^{1}H NMR, ^{13}C NMR, HRMS, and HPLC of compound B29

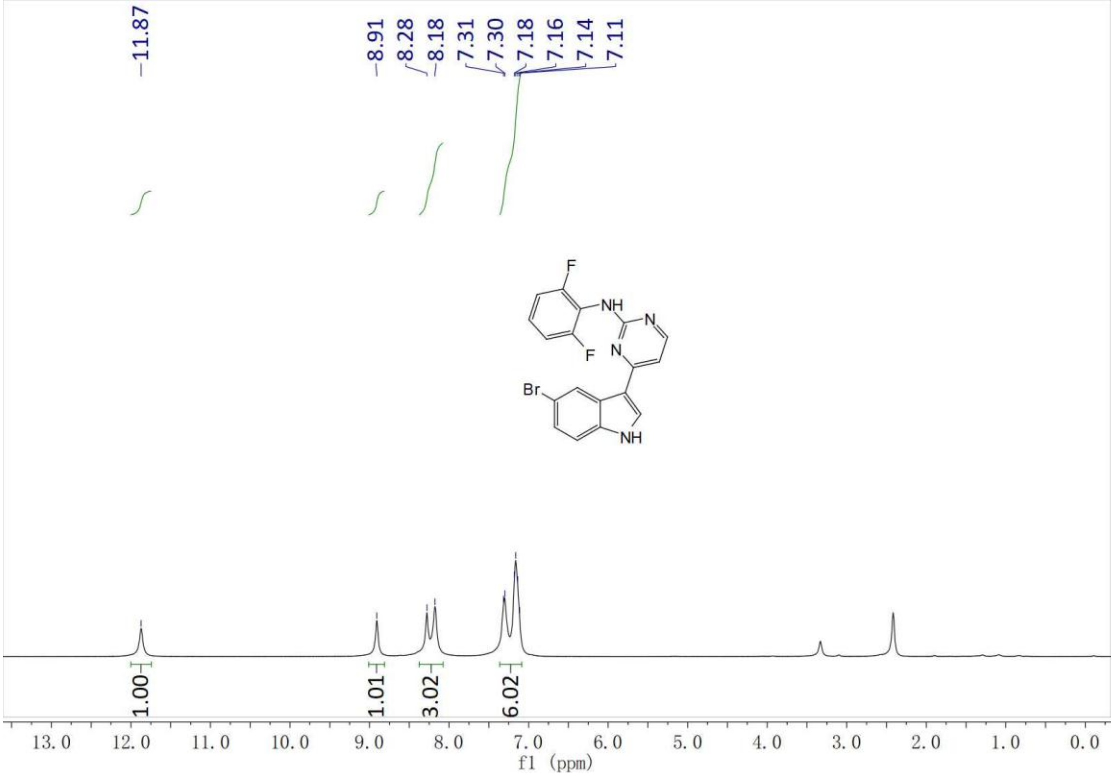

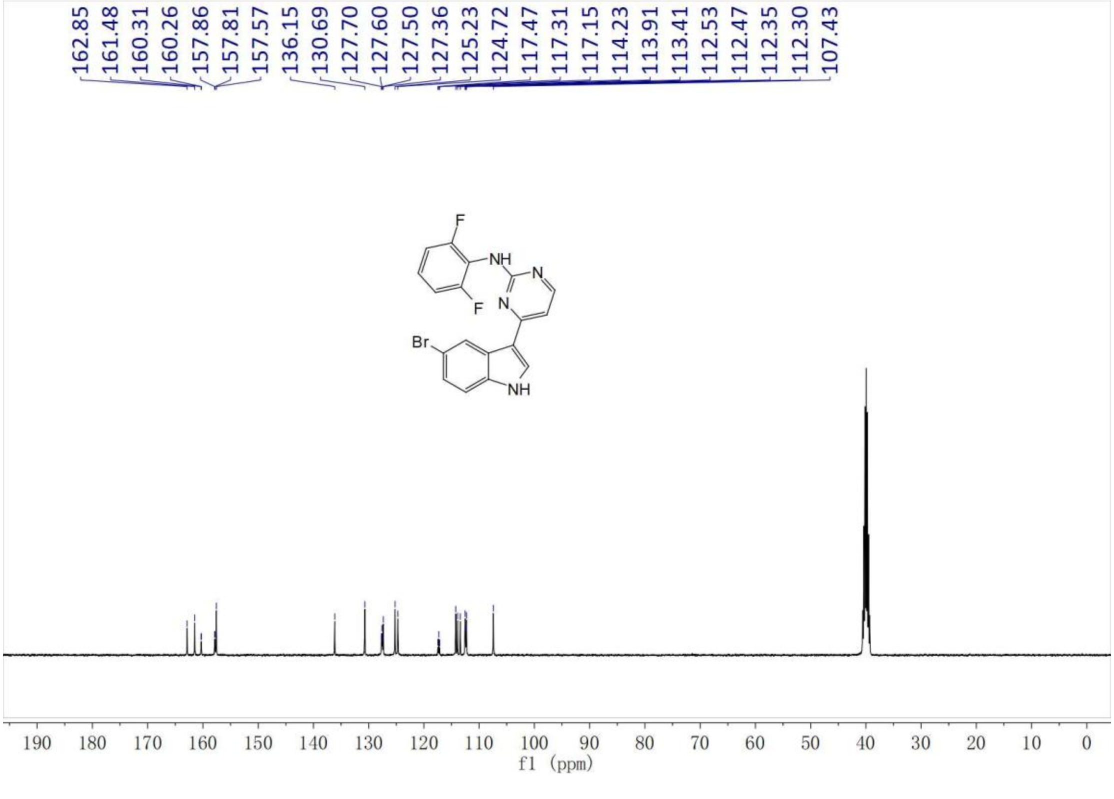

Display Report

Analysis Info Acquisition Date 12/10/2020 17:24:44 PM

Sample Name TC21
Comment

Acquisition Parameter

Source Type	ESI	Ion Polarity	Positive	Set Nebulizer	2.0 Bar
Focus	Active	Set Capillary	4500 V	Set Dry Heater	200 °C
Scan Begin	50 m/z	Set End Plate Offset	-500 V	Set Dry Gas	8.0 l/min
Scan End	3000 m/z	Set Charging Voltage	2000 V	Set Divert Valve	Waste
		Set Corona	0 nA	Set APCI Heater	0 °C

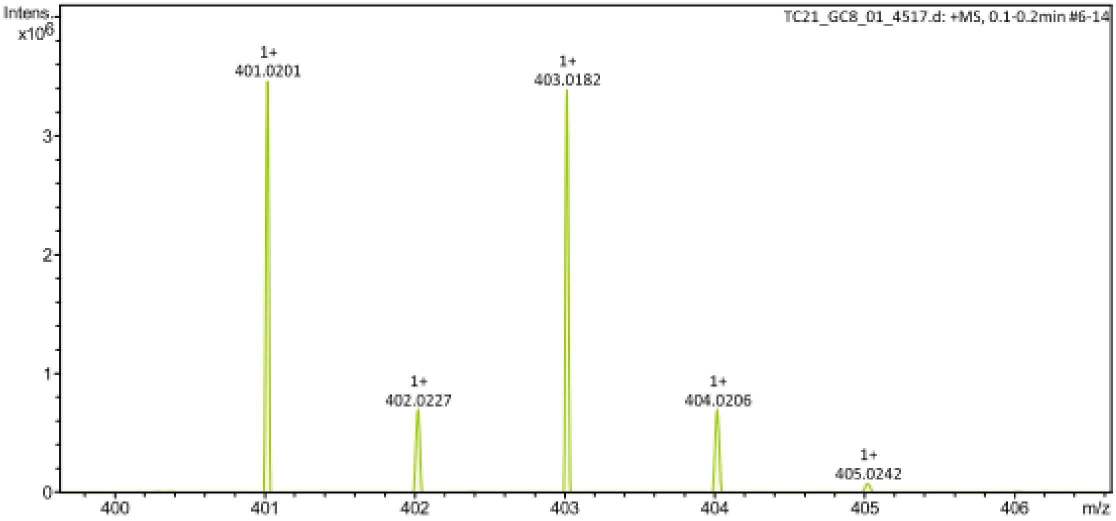

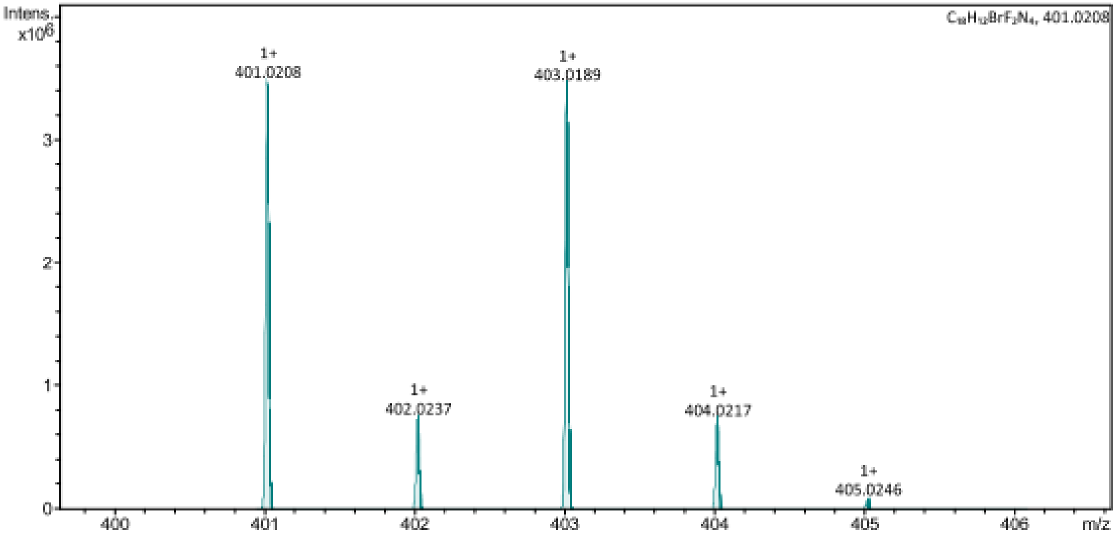

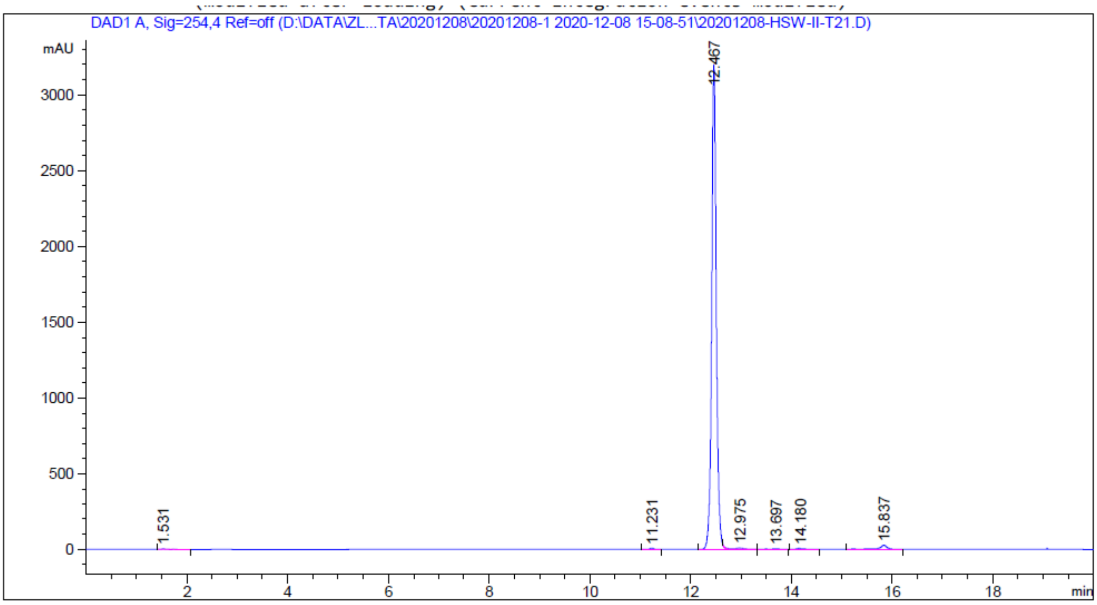

Signal 1: DAD1 A, Sig=254,4 Ref=off

Peak #	RetTime [min]	Type	Width [min]	Area [mAU*s]	Height [mAU]	Area %
1	1.531	BB	0.0888	19.03418	3.90860	0.0907
2	11.231	BB	0.1099	39.37353	6.57168	0.1876
3	12.467	BV R	0.1160	2.04669e4	3140.21143	97.4932
4	12.975	VV E	0.2124	122.42989	7.91835	0.5832
5	13.697	VB E	0.1539	43.17803	4.13182	0.2057

1260R 12/8/2020 9:23:56 PM BY

Data File D:\DATA\ZLM\DATA\20201208\20201208-1 2020-12-08 15-08-51\
Sample Name: 20201208-HSW-II-T21

Peak #	RetTime [min]	Type	Width [min]	Area [mAU*s]	Height [mAU]	Area %
6	14.180	BB	0.1712	46.89018	4.44035	0.2234
7	15.837	BB	0.1667	255.36047	25.09039	1.2164

Totals : 2.09932e4 3192.27262

^{1}H NMR, ^{13}C NMR, HRMS, and HPLC of compound B30

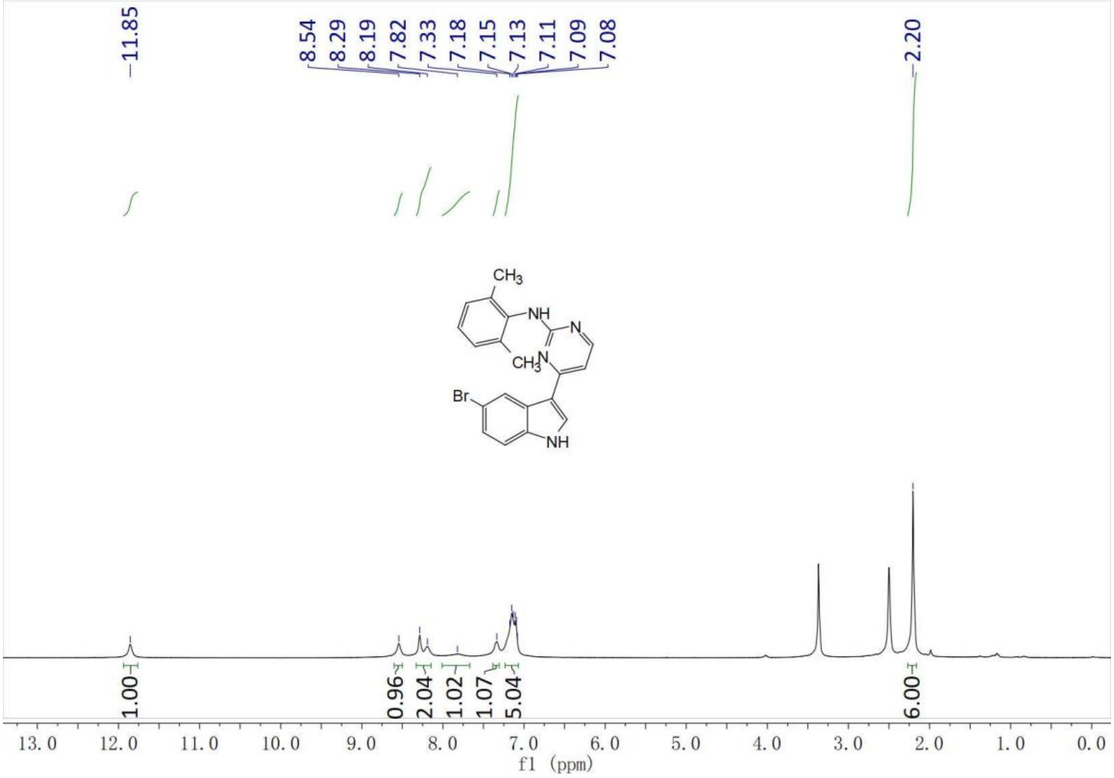

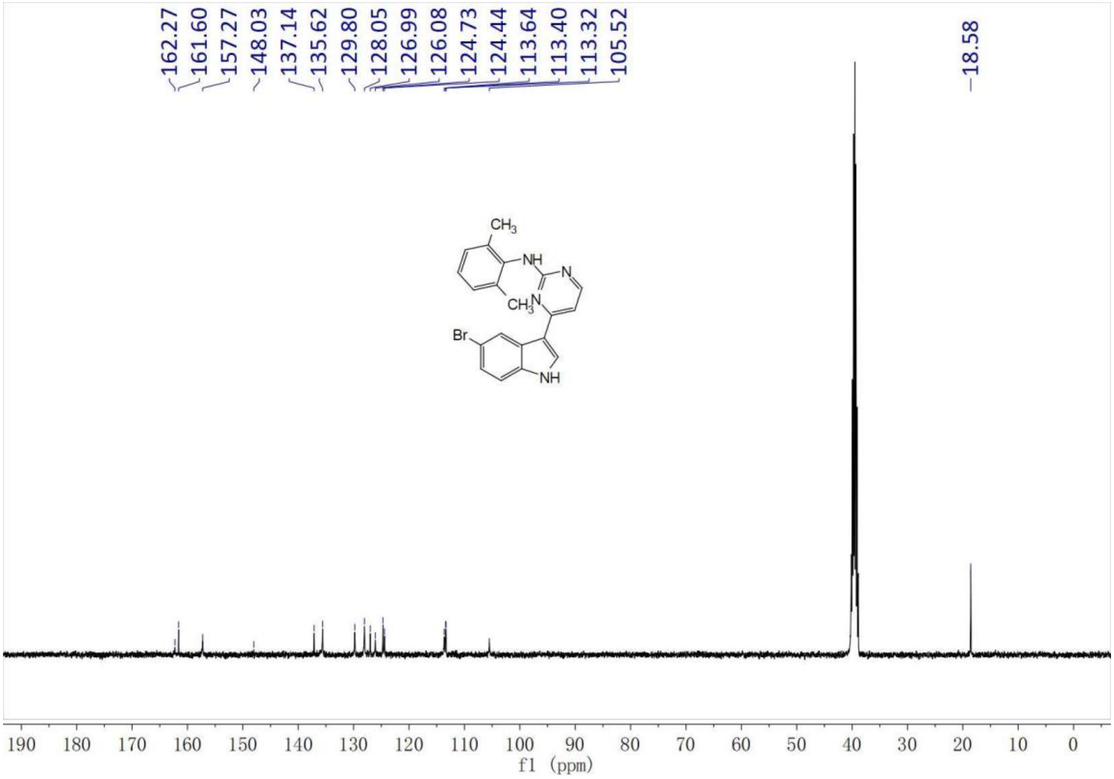

Display Report

Analysis Info

Acquisition Date 12/10/2020 17:53:09 PM

Sample Name TC33
Comment

Acquisition Parameter

Source Type	ESI	Ion Polarity	Positive	Set Nebulizer	2.0 Bar
Focus	Active	Set Capillary	4500 V	Set Dry Heater	200 °C
Scan Begin	50 m/z	Set End Plate Offset	-500 V	Set Dry Gas	8.0 l/min
Scan End	3000 m/z	Set Charging Voltage	2000 V	Set Divert Valve	Waste
		Set Corona	0 nA	Set APCI Heater	0 °C

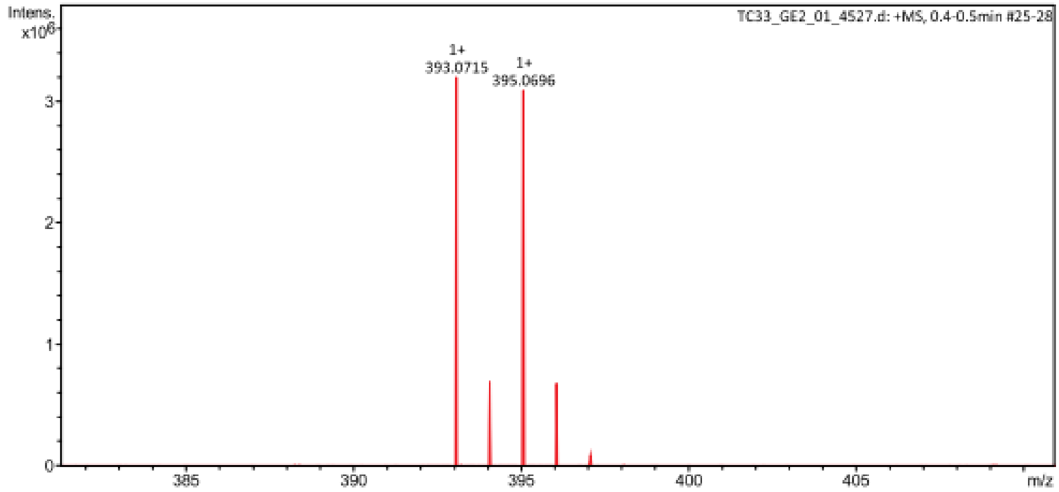

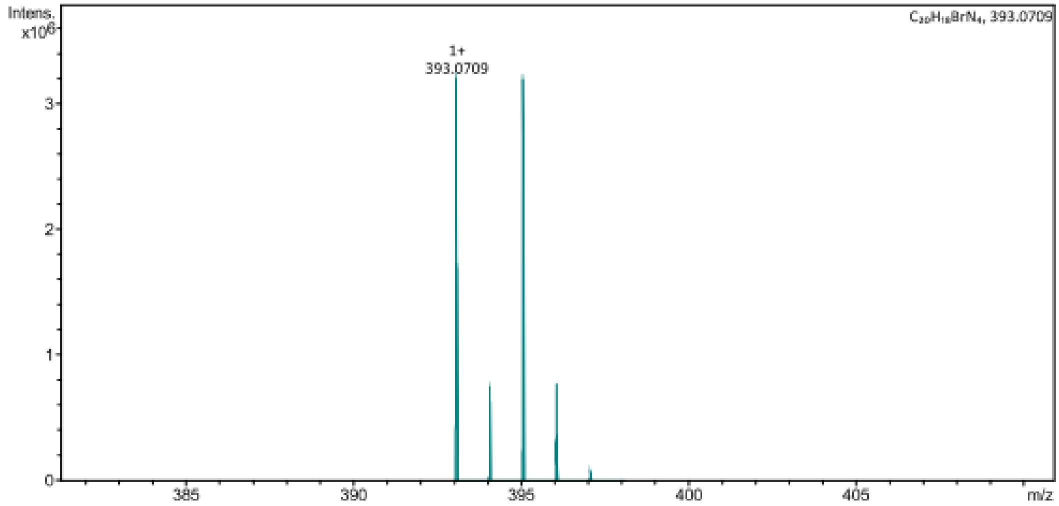

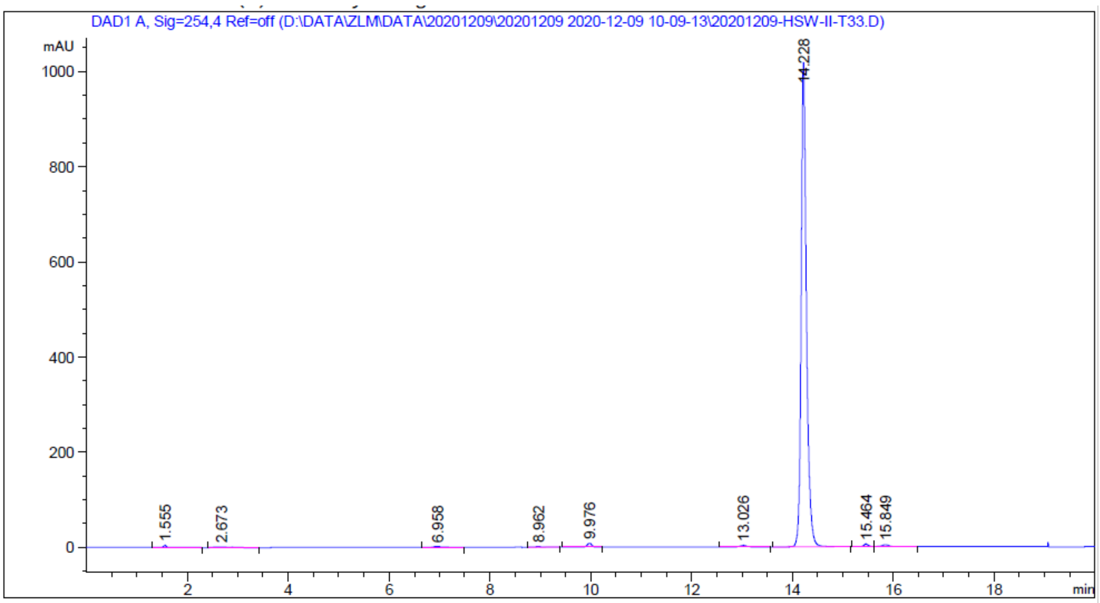

Signal 1: DAD1 A, Sig=254,4 Ref=off

Peak #	RetTime [min]	Type	Width [min]	Area [mAU*s]	Height [mAU]	Area %
1	1.555	BB	0.0849	19.57547	4.32101	0.2550
2	2.673	BB	0.4044	31.56690	1.10808	0.4112
3	6.958	BB	0.1925	20.34305	1.64241	0.2650

1260R 12/9/2020 6:05:57 PM BY

Data File D:\DATA\ZLM\DATA\20201209\20201209 2020-12-09 10-09-13\2020120
Sample Name: 20201209-HSW-II-T33

Peak #	RetTime [min]	Type	Width [min]	Area [mAU*s]	Height [mAU]	Area %
4	8.962	BB	0.1471	13.16242	1.54891	0.1714
5	9.976	BB	0.1191	55.22655	8.14670	0.7193
6	13.026	BB	0.1721	39.09184	3.45292	0.5092
7	14.228	BB	0.1273	7413.85596	990.95563	96.5687
8	15.464	BV	0.1181	36.72093	5.48696	0.4783
9	15.849	VB	0.1685	47.74652	4.33399	0.6219

Totals : 7677.28965 1020.99660

^{1}H NMR, ^{13}C NMR, HRMS, and HPLC of compound B31

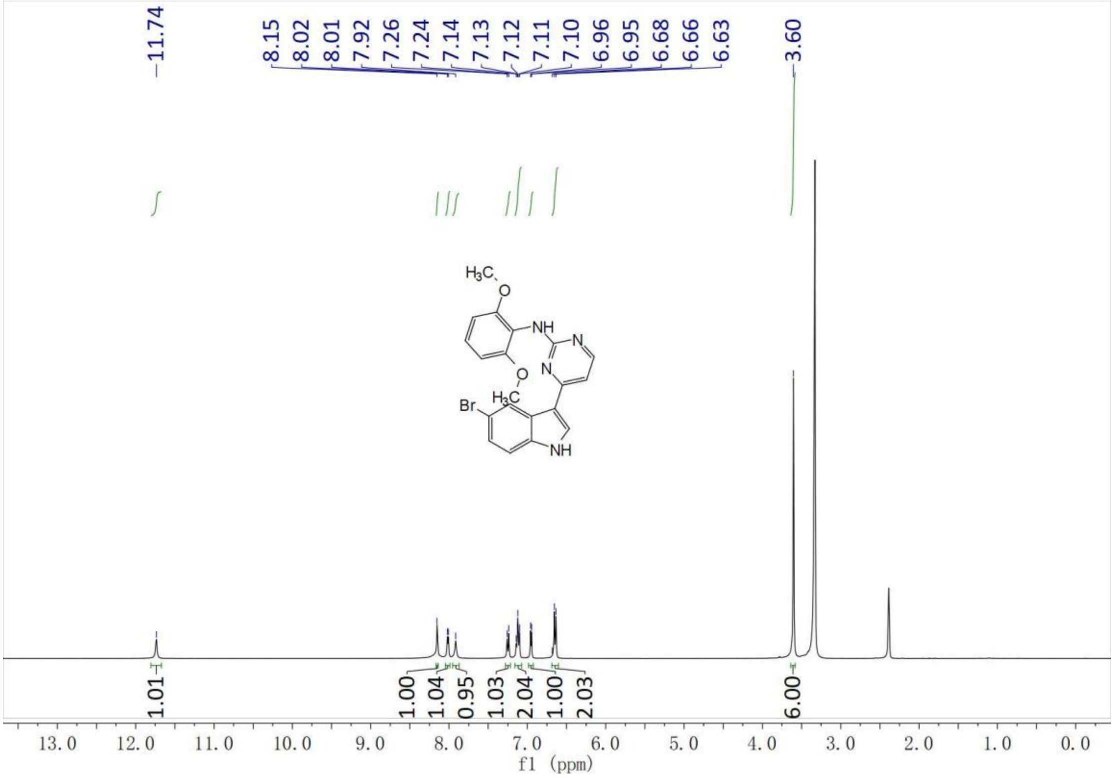

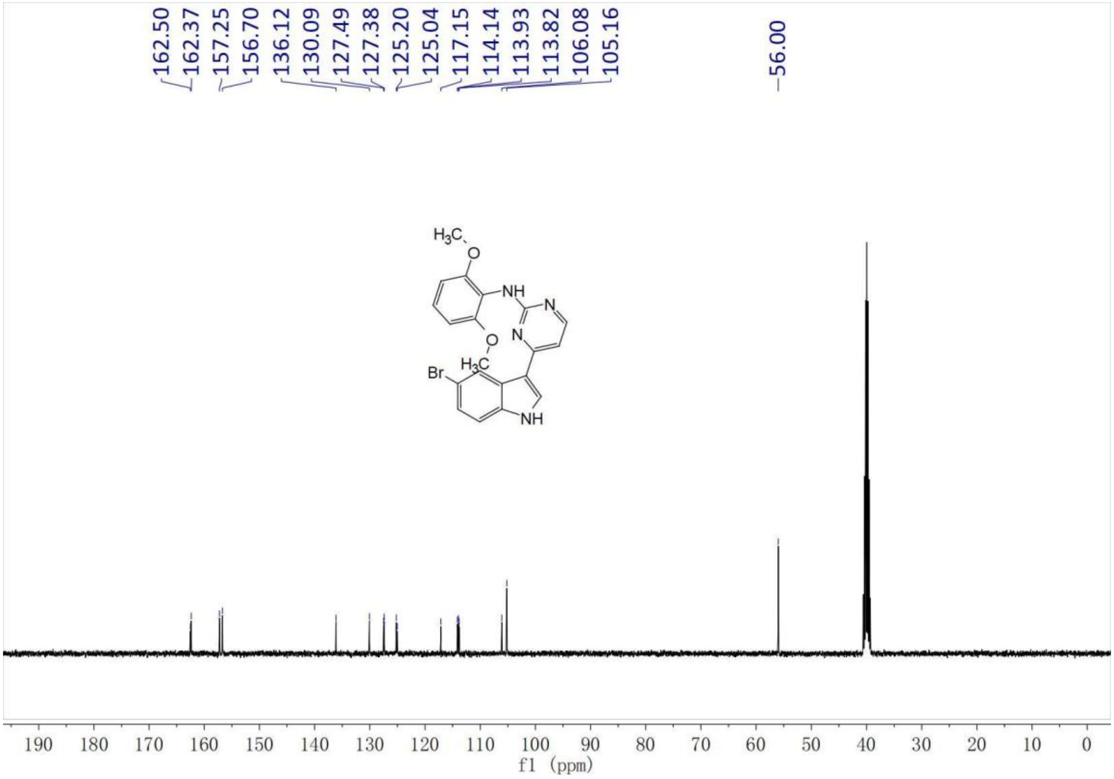

Display Report

Analysis Info

Acquisition Date 12/10/2020 17:27:53 PM

Sample Name TC23
Comment

Acquisition Parameter

Source Type	ESI	Ion Polarity	Positive	Set Nebulizer	2.0 Bar
Focus	Active	Set Capillary	4500 V	Set Dry Heater	200 °C
Scan Begin	50 m/z	Set End Plate Offset	-500 V	Set Dry Gas	8.0 l/min
Scan End	3000 m/z	Set Charging Voltage	2000 V	Set Divert Valve	Waste
		Set Corona	0 nA	Set APCI Heater	0 °C

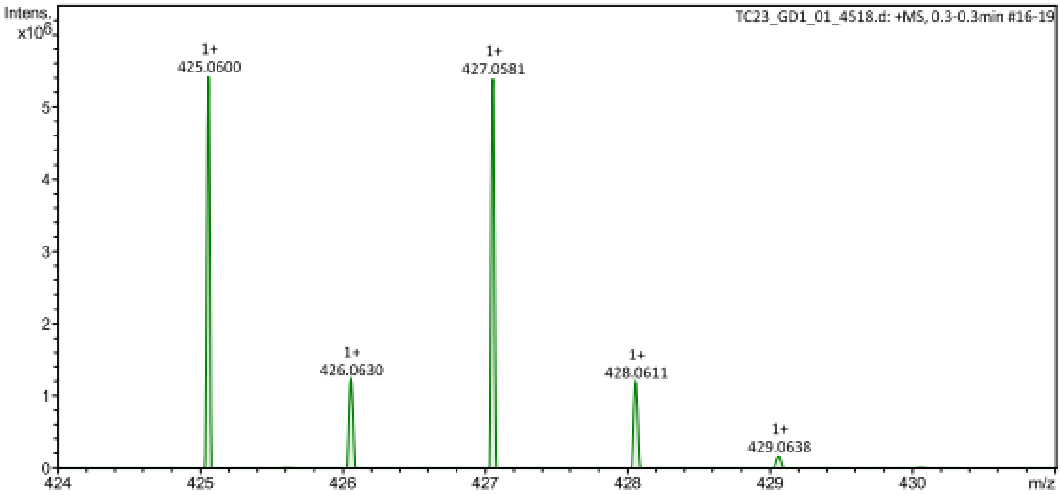

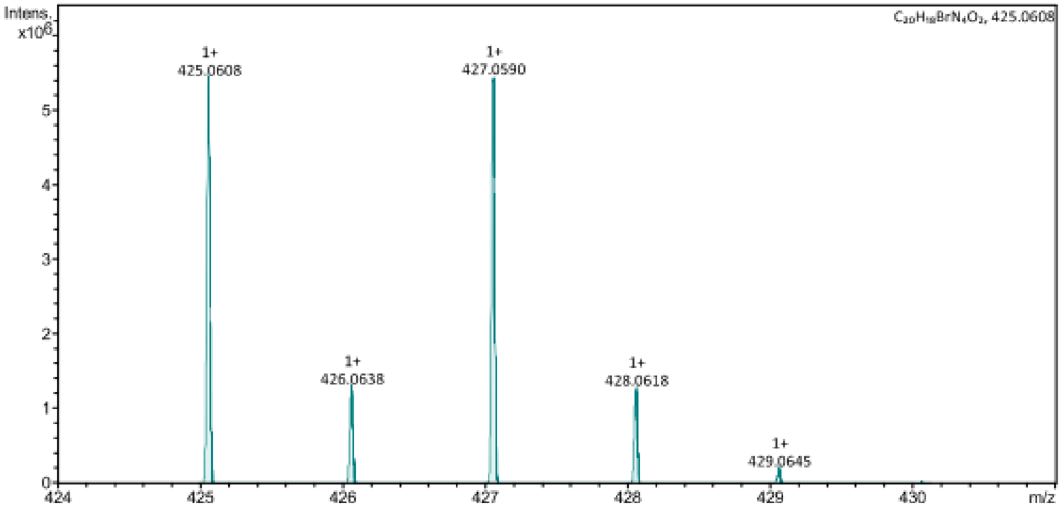

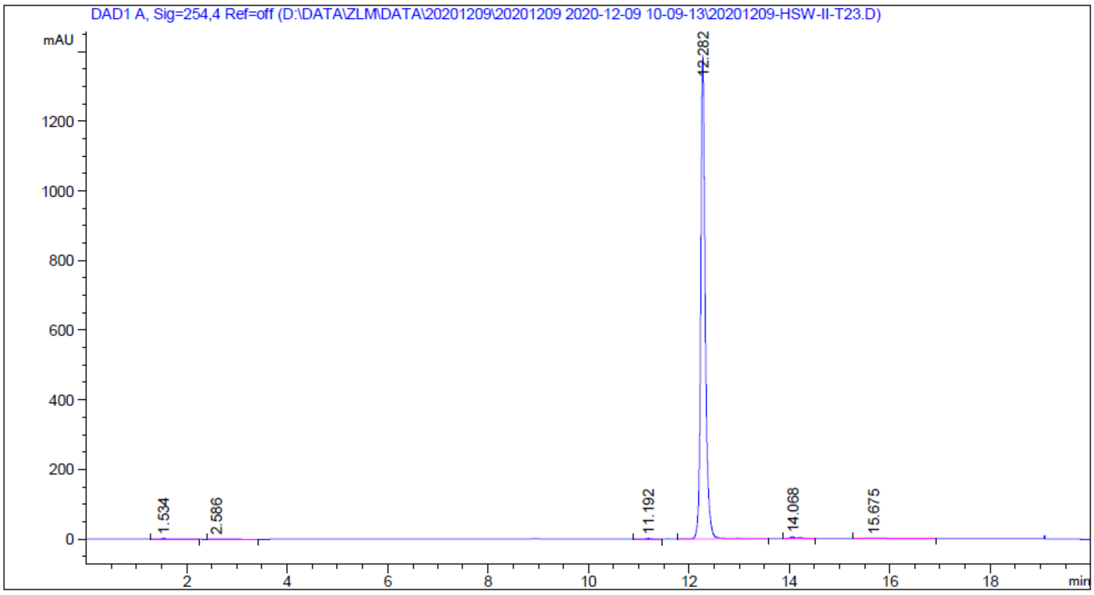

Signal 1: DAD1 A, Sig=254,4 Ref=off

Peak #	RetTime [min]	Type	Width [min]	Area [mAU*s]	Height [mAU]	Area %
1	1.534	BB	0.1034	16.69835	3.07103	0.1867
2	2.586	BB	0.3318	30.85929	1.26880	0.3451
3	11.192	BB	0.1216	11.59501	1.65803	0.1297
4	12.282	BB	0.1156	8749.63770	1351.11157	97.8508
5	14.068	BB	0.1573	60.72800	5.65289	0.6791
6	15.675	BB	0.6593	72.29550	1.68921	0.8085

1260R 12/9/2020 1:26:54 PM BY

Data File D:\DATA\ZLM\DATA\20201209\20201209 2020-12-09 10-09-13\20201
Sample Name: 20201209-HSW-II-T23

Peak #	RetTime [min]	Type	Width [min]	Area [mAU*s]	Height [mAU]	Area %
Totals :				8941.81385	1364.45153	

Dose-response curves of the GSK3β assays

Compound number	Curve
A-13	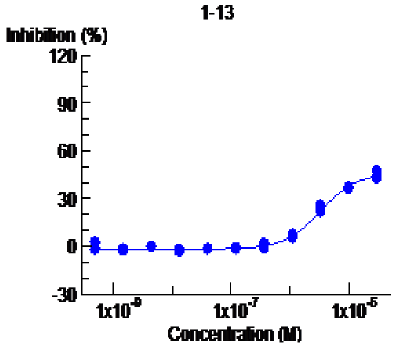
A-26	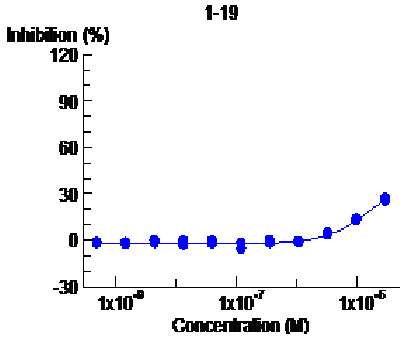
B-29	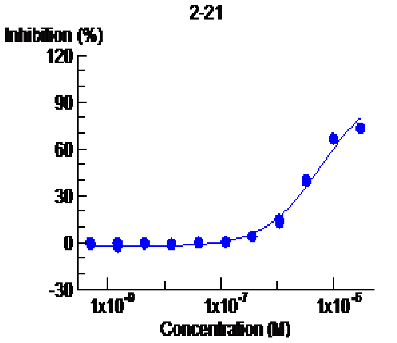
B-30	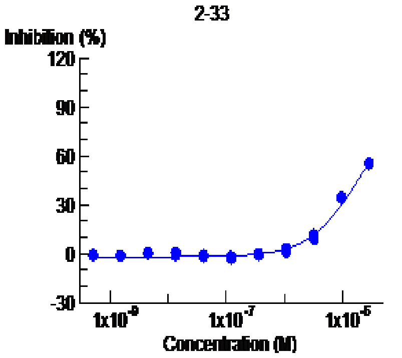
